# Substituent and Ring-Number Effects on the Kinetics of PAH + OH Reactions: A QSAR–DOE Approach with Tunneling Corrections

**DOI:** 10.3390/molecules31020265

**Published:** 2026-01-13

**Authors:** Cezary Parzych, Maciej Baradyn, Artur Ratkiewicz

**Affiliations:** 1 Doctoral School, University of Bialystok, Ciołkowskiego 1K Street, 15-245 Bialystok, Poland; 2 Department of Chemistry, University of Bialystok, Ciołkowskiego 1K Street, 15-245 Bialystok, Poland

**Keywords:** polycyclic aromatic hydrocarbons, kinetics, radical reaction, QSAR model

## Abstract

The reactions of hydrogen transfer by hydroxyl radicals involving polycyclic aromatic hydrocarbons (PAH) are important, because these compounds contribute to environmental pollution and negatively affect human health. Hydroxyl radicals play a key role in atmospheric processes. This study analyzed the influence of the substituent and the number of aromatic rings in the compound on the kinetics of the hydrogen-transfer reaction. This work proposes for the first time a quantitative structure–activity relationship-based statistical framework combining design of experiments and tunneling corrections to predict PAH + ·OH kinetics. The main objective of this research was to identify which molecular features and substituent effects most strongly govern tunneling and reactivity, thereby enabling the rational prediction of PAH behavior in atmospheric and combustion environments. For this purpose, a quantitative structure–activity relationship model was developed using 22 descriptors, and their relationship with the kinetic parameters of the reaction was determined using statistical tools such as design of experiments and partial least squares.

## 1. Introduction

Studying the reaction kinetics of polycyclic aromatic hydrocarbons (PAHs):

PAH + OH∙ → PAH∙ +H_2_O

is important for many reasons. Polycyclic aromatic hydrocarbons are substances that make up environmental pollution—air, water, and soil. These compounds pose a serious threat to human health due to their carcinogenic properties [1,2,3]. PAHs are released into the environment mainly through anthropogenic processes such as incomplete combustion of hydrocarbon fuels, oil refining, and waste incineration [4,5,6]. They are also formed naturally during forest fires or volcanic eruptions [7], although the main cause of the presence of these compounds on Earth is human activity. In the environment, PAHs may exist in the atmosphere as suspended particles or in gaseous form, adsorb onto soil surfaces, migrate into surface waters, and accumulate in living organisms [8,9]. Their reactions with the hydroxyl radical are also important because of the significance of OH∙ in the processes of atmospheric oxidation and combustion [10,11,12]. In addition, the hydroxyl radical contributes to the biodegradation of PAHs, so understanding the kinetics of this reaction could be the starting point for research into the removal of hydrocarbon derivatives from the environment [13]. In addition, the kinetic data can be used to build models of reactions in the atmosphere or to design more effective methods for cleaning the environment of PAHs derivatives, which in turn will help improve air quality and reduce the incidence of diseases caused by particulate pollutants. Although this study focused on gas-phase reactions, outcomes for other matrices (e.g., soil or water) may vary. However, this limitation does not reduce the relevance of the findings, given that PAHs predominantly originate in the atmosphere and are subsequently transferred to other environmental matrices.

The PAH + OH∙ reaction can proceed in two ways: addition of a radical to a hydrocarbon or transfer of a hydrogen atom from a hydrocarbon to a radical with the formation of a water molecule and a hydrocarbon radical. In this research, hydrogen-transfer reactions were studied because of the greater importance of these reactions at high temperatures, which are characteristic of combustion reactions [14,15,16].

**Figure 1:**
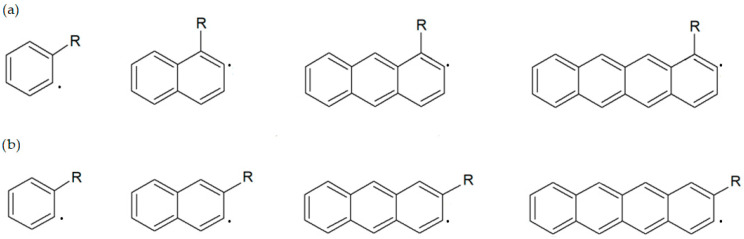
Formulas of the products of the reactions analyzed: (**a**) benzene and alpha derivatives; (**b**) benzene and beta derivatives.

The main objective of this study was to determine the effect of the substituent attached to the hydrocarbon and the number of aromatic rings on the kinetics of the hydrogen-transfer reaction by the hydroxyl radical. The focus was on the tunneling factor [17] and the ratio of the constant rates of the substituted to the unsubstituted compound. For this purpose, derivatives of benzene (which does not belong to the PAH group, but has similar properties), naphthalene, anthracene, and tetracene were analyzed. The focus was on the reactions occurring closest to the substituent: in the case of benzene, it was the ortho position, for the other alpha-substituted acenes, it was position 2, and for beta-substituted acenes, position 3 was studied (Figure 1). The effect of 13 substituents was analyzed:

- halogen atoms: -F, -Cl, -Br
- unbranched alkyl chains: -CH_3_, -C_2_H_5_, -C_3_H_7_, -C_4_H_9_
- nitrogen-containing groups: -NH_2_, -NO_2_
- oxygen-containing groups: -OH, -OOH
- C=O bond-containing groups: -CHO, -COOH

## 2. Materials and Methods

### 2.1. Study of Reaction Kinetics—Transition State Theory

This study employed transition state theory (TST), which postulates that a chemical reaction proceeds through the formation of a transient complex between the reactants and products, referred to as the transition state. The geometry of the transition state is defined as the saddle point of the first degree of the potential energy hypersurface (PES) [18,19]. According to this theory, the rate constant is expressed by the Eyring equation:

$$k\left(T\right)=\;\kappa (T)\sigma \frac{k_{b}T}{h}\frac{Q^{‡}\left(T\right)}{\Phi^{R}\left(T\right)}exp(\frac{{-∆V}^{‡}}{k_{b}T})$$

κ(T) is the transition factor taking into account quantum tunneling, *σ* is the symmetry number of the reaction, $k_{b}\;$ is Boltzman’s constant, h is Planck’s constant, *T* is temperature, $Q^{‡}\left(T\right)$ i $\Phi^{R}\left(T\right)$ denote the total partition functions of the transition state and the reactants, respectively, and ${∆V}^{‡}\;$ is the barrier height of the reaction. In addition, the transition state partition function takes into account the absence of one oscillatory degree of freedom corresponding to the reaction coordinates, and the partition function $\Phi^{R}\left(T\right)$ is the product of the partition functions of the individual reactants.

While anharmonic, variational, and multidimensional tunneling effects can in principle influence absolute rate constants, prior systematic studies of PAH + OH hydrogen abstraction have shown that such corrections yield only minor adjustments to relative trends within this reaction class [20]. For instance, hindered rotor corrections typically deviate by less than 10% from unity, and Eckart tunneling—applied here—has been extensively validated for hydrogen-transfer reactions in aromatic systems [17,21]. Given the large dataset of 91 derivatives, applying higher-level dynamical corrections to each system would be computationally prohibitive without altering the primary QSAR-derived structure–kinetic relationships. Therefore, the present approach provides a consistent and efficient framework for comparative kinetic analysis.

### 2.2. Development of a Quantitative Structure–Activity Relationship (QSAR) Model

The kinetics of the studied reactions were analyzed using a QSAR model. This approach applies statistical techniques, such as multiple linear regression (MLR) or design of experiments (DOE), to establish relationships between molecular descriptors and reaction kinetic parameters. The use of the QSAR approach in the analysis of the reactivity of PAHs towards hydroxyl radicals is justified due to the possibility of quantitatively linking structural features with observed kinetic properties. The reactivity of these compounds largely depends on the nature of substituents, which affect both the distribution of electron density in aromatic rings and the spatial accessibility of OH∙ radical attack sites. A set of 22 descriptors was initially considered based on their known relevance to hydrogen-transfer reactions, tunneling phenomena, and aromatic substitution effects. These include:

- Electronic descriptors: HOMO, LUMO, HOMO–LUMO gap, polarizability (α), dipole moment (μ), natural bond orbital (NBO) values.
- Reactivity indices: Fukui functions *f^+^*, *f^−^*, *f*^0^, and the dual descriptor Δ*f* (to capture site-specific electrophilic/nucleophilic character).
- Thermodynamic descriptors: Specific heat (C_p_) and entropy (S^0^) at 300 K (to account for temperature-dependent effects).
- Topological/ELF descriptors: Local minima/maxima of the electron localization function (ELF) within the reaction center and substituent (to quantify electron localization/delocalization).
- Energetic descriptor: total electronic energy (*E_SCF_*).
- Structural descriptors: Number of aromatic rings (*N_r_*) and substituent parameter (*S*), expressed as the product of dipole moment and specific heat.

These descriptors collectively encode: local reactivity (Fukui indices, ELF extrema)electronic structure (frontier orbitals, charge distribution), steric and conjugation effects (ring number, substituent bulk), and thermodynamic driving forces (entropy, heat capacity). A detailed description of the utilized quantities is provided below.

The Fukui function given by the formula:

$$f\left(r\right)={\left(\frac{\partial \rho \left(r\right)}{\partial N}\right)}_{V}$$

In this equation, r is coordinates, $\rho \left(r\right)$ electron density, N is the number of electrons in the system, and V is external potential [22]. When approximated by the finite difference method, three types of Fukui functions are obtained: $f^{+}\left(r\right),\;f^{-}\left(r\right),\;and\;f^{0}\left(r\right)\;$ [23]. They describe the susceptibility of the system to nucleophilic, electrophilic, and radical attack, respectively, and are expressed as:

$$f^{+}\left(r\right)=\;\rho_{\left(N+1\right)}\left(r\right)-\rho_{N}\left(r\right)$$

$$f^{-}\left(r\right)=\rho_{N}\left(r\right){-\rho }_{\left(N-1\right)}\left(r\right)$$

$$f^{0}\left(r\right)=\frac{f^{+}\left(r\right)+f^{-}\left(r\right)}{2}=\frac{\rho_{\left(N+1\right)}\left(r\right){-\rho }_{\left(N-1\right)}\left(r\right)}{2}$$

To calculate these indices, in addition to the geometry of the neutral molecule, the geometries of the cation (N + 1) and the anion (N − 1) are necessary [24].

In addition, a frequently used parameter is the dual descriptor (∆f), for which a positive value suggests greater susceptibility of the system to nucleophilic attack, while a negative value indicates susceptibility to electrophilic attack [25]:

$$∆f=f^{+}\left(r\right)+f^{-}\left(r\right)=\rho_{\left(N+1\right)}\left(r\right)+{-\rho }_{\left(N-1\right)}\left(r\right)-2\rho_{N}\left(r\right)$$

These functions can also depend on the partial charge on the atom that is the center of the reaction q:

$$f^{+}=q_{N}-q_{\left(N+1\right)}$$

$$f^{-}=q_{\left(N-1\right)}-q_{N}$$

$$f^{0}=\frac{q_{\left(N-1\right)}-q_{\left(N+1\right)}}{2}$$

$$∆f=f^{+}-f^{-}=2q_{N}-q_{\left(N+1\right)}-q_{\left(N-1\right)}$$

Partial charges were determined using the Hirshfeld method because of its optimality in determining electrophilicity and nucleophilicity and because it has been used in other studies related to the Fukui function [26,27,28,29,30]. Partial charges on a carbon atom ($q_{C}$) and hydrogen atom ($q_{H}$) being the center of the reaction also served as descriptors.

Further parameters that were also used to build the QSAR model were the descriptors calculated for the product structures: the HOMO and LUMO energies, the difference between these energies (HOMO–LUMO) expressed in Hartree, the polarizability α in the unit bohr^3^, dipole moment (μ) in Debye, and the total energy of the molecule (>E_SCF_) in Hartree.

Specific heat (C_p_) and entropy (S^0^), thermodynamic parameters determined at 300 K, also served as a descriptors.

The next two descriptors were the minimum and maximum value of the electron localization function calculated within the reaction center (*ELF^C^_min_* i *ELF^C^_max_*) and within the substituent attached to the product (*ELF^R^_min_* i *ELF^R^_max_*). In QSAR equations, ELF is expressed as E. The values of this function are between 0 and 1, and are expressed by the formula:

$$ELF=\frac{1}{1+{\left(\frac{D(r)}{D_{0}(r)}\right)}^{2}}$$

D(r) is the kinetic energy dissipation function, and a $D_{0}(r)$ is a reference to the electron gas. They are given by:

$$D\left(r\right)=t\left(r\right)-\frac{1}{4}\frac{{\left|\nabla \rho (r)\right|}^{2}}{\rho (r)}D_{0}\left(r\right)=\frac{3}{5}{\left({6\pi }^{2}\right)}^{2/3}{\rho (r)}^{5/3}$$

$t\left(r\right)$ stands for local kinetic energy density, and ρ(r) is electron density.

An ELF value close to 1 indicates strong localization of electrons, and a value close to 0 strong delocalization [31]. This function is an important part of molecular electron density theory (MEDT), which has been successfully applied in a number of theoretical studies including the Diels–Alder reaction or cycloaddition [32,33,34,35,36].

The last descriptor for reaction products was the natural bond orbital (NBO) value on the carbon atom that is the center of the reaction. The NBO is employed to analyze the electron structure of molecules, and is based on transforming the wave function to a form resembling a Lewis description of chemical bonds. It utilizes ordered orbital bases to do so: natural atomic orbitals (NAOs), natural hybrid orbitals (NHOs), and NBO [37]. This method is widely used and sometimes in combination with ELF [38,39,40,41].

To avoid overfitting and retain only statistically significant descriptors, we applied two sequential filters, as follows.

1. ANOVA (*p*-value < 0.05)

Each descriptor was tested for its individual correlation with the target kinetic parameter (*κ* or *k_s_*/*k_u_*). Descriptors with *p* > 0.05 were excluded from the initial MLR/DOE models.

2. Variable importance in projection (VIP) > 1 and cross-validation (CV)

For the PLS models, VIP scores were computed to assess the contribution of each descriptor to the variance in the response. Only descriptors with VIP > 1 were retained in the final PLS regressions. Cross-validation was also used. This two-step approach ensured that the retained descriptors were both statistically significant and informative for the predictive models.

### 2.3. Computational Details

The optimized geometries of the substrate molecules, as well as their corresponding cations and anions (required for the calculation of Fukui indices), products, and transition states, were obtained at the M06-2X/aug-cc-pVDZ level of theory using the Gaussian 16 program [42]. The aug-cc-pVDZ basis set was selected because the inclusion of diffuse (“aug”) functions is crucial for accurately describing long-range interactions and charge redistribution that occur during hydrogen abstraction and tunneling processes. This augmentation enhances the representation of the electronic density in the reaction barrier region, resulting in more reliable activation energies and tunneling corrections compared with non-augmented double-zeta sets. The choice of the aug-cc-pVDZ basis set is justified by its balance of accuracy and computational cost for identifying relative trends within a reaction class, where systematic errors tend to cancel. Benchmark calculations for the parent benzene + •OH reaction [20] showed minimal sensitivity to increasing the basis set from double to triple zeta (without diffuse functions), and additional single-point tests with aug-cc-pVTZ on a representative subset in this work confirmed that relative barrier differences and tunneling corrections remained consistent (within 1 kcal/mol and 5%, respectively).

The same software was also employed to calculate electronic descriptors such as HOMO, LUMO, the HOMO–LUMO gap, polarizability (*α*), dipole moment (*μ*), and NBO parameters. To calculate reaction rate constants, tunneling coefficients using Eckart’s method and descriptors *C_p_* and *S*^0^, the MSMC program was used [43]. Multiwfn software 3.8 was utilized to calculate *f^+^*, *f^−^*, *f*^0^, Δ*f*, *q_C_*, and *q_H_* [44]. The programs used for statistical analysis were: R 4.4.2 and Statistica 13 (Tibco Software Inc. San Ramon, CA, USA) [45].

## 3. Results and Discussion

The relationship between the number of aromatic rings, the type of substituent, and the values of the kinetic parameters—the tunneling factor and the ratio of the reaction rate constants of substituted acene to unsubstituted ones at 300 K and 1500 K—were investigated. For this purpose, the response surface of a given kinetic parameter was determined depending on the number of aromatic rings and the type of substituent. A QSAR model was also developed, showing the dependence of the kinetic parameter value on the descriptors using DOE. The considered derivatives was divided according to the position of the substituent (*α* vs. *β*). Both chemically and statistically, this separation is essential: α-substituents exhibit both dominant electronic effects (such as resonance and inductive) and primary steric effects when they are in direct conjugation or close to the reactive/active site on the benzene core. On the other hand, *β*-substituents are separated by an extra atom, which leads to different conformational and secondary steric influences and decreased electronic effects that are frequently more inductive.

To construct statistically robust QSAR models, molecular descriptors were filtered based on a significance threshold (ANOVA *p*-value < 0.05). Recognizing the divergent electronic and steric influences of substituent position, the analysis was conducted on two distinct datasets: (i) *α*-substituted and benzene derivatives, and (ii) *β*-substituted and benzene derivatives, yielding two separate predictive equations.

### 3.1. Tunneling Factor

**Figure 2:**
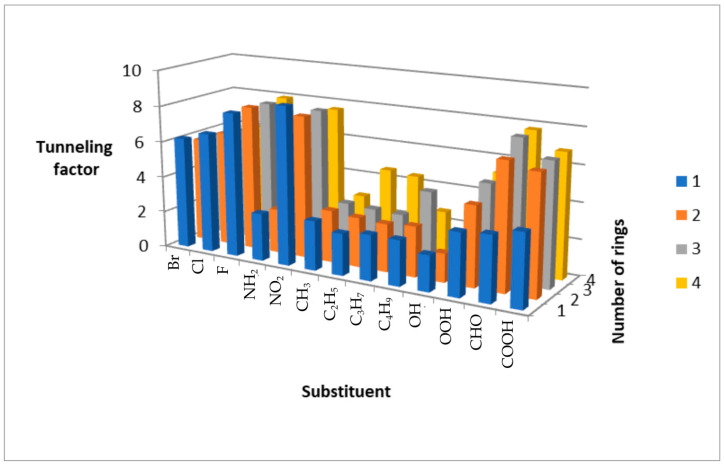
Plot of the dependence of the tunneling factor on the number of aromatic rings.

Our computational results demonstrate that substituent effects on the hydrogen-abstraction pathway in PAH + OH· reactions are governed not only by changes in classical activation barriers (Δ*G^‡^*), but critically by modifications of the barrier shape that control quantum tunneling [21]. In particular, strong electron-withdrawing groups (EWGs) such as –NO_2_ and halogens typically increase the classical barrier height while simultaneously shifting the transition state toward the reactants (an “early” TS). This geometric shift shortens the breaking C–H distance at the barrier apex and steepens the potential-energy surface around the TS, producing a markedly narrower tunneling width. The combined effect of increased C–H polarization and reduced effective mass along the reaction coordinate further enhances the tunneling transmission coefficient *κ*(*T*). Consequently, nitro- and halo-substituted PAHs can show larger tunneling corrections (higher *κ*(*T*)) than electron-rich analogues, despite exhibiting higher Δ*G^‡^* values. These findings underline that barrier width and shape—not height alone—are decisive in the tunneling contribution in hydrogen abstraction by OH·, and therefore that mechanistic interpretations based solely on classical barrier heights may be misleading for substituted PAHs. Consequently, reporting both Δ*G^‡^* and *κ*(*T*) (or tunneling-corrected rate constants) to convey substituent effects on reactivity entirely is recommended. The tunneling factor (*κ*) for *α*-substituted PAHs exhibits clear dependence on both substituent type and the number of aromatic rings. As shown in Figure 2, *κ* is highest for halogenated, nitro, and carbonyl-substituted derivatives, and lowest for amines, alcohols, and alkyl chains. This trend correlates with the electron-withdrawing character of the substituent, which enhances hydrogen abstraction tunneling.

**Figure 3:**
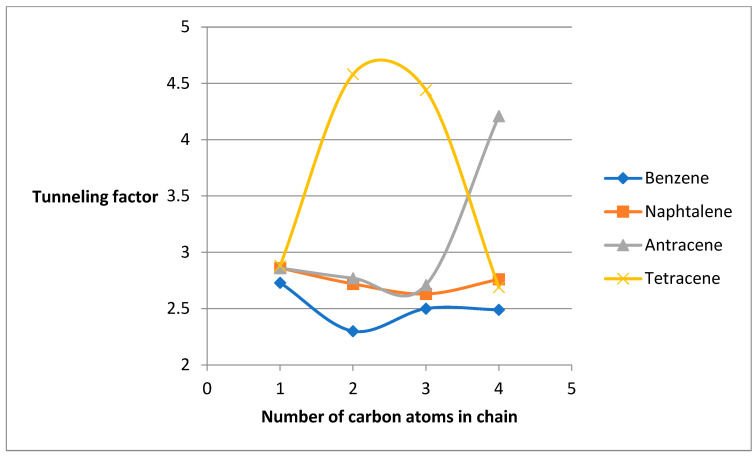

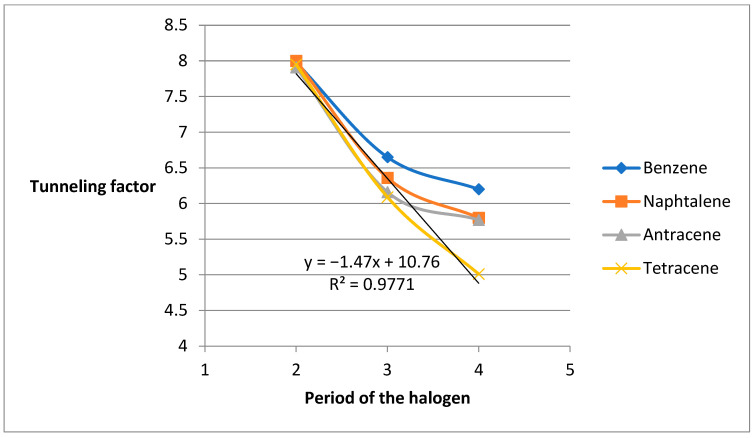
Graphs of the dependence of the tunneling factor on the period number of the attached halogen and on the number of carbon atoms in the attached chain.

A nonlinear decrease in *κ* is observed with increasing halogen period (F > Cl > Br), attributable to reduced electronegativity and polarizability (Figure 3). For alkyl substituents, *κ* generally decreases with chain length, with the lowest value for ethylbenzene. Exceptions occur in tetracene derivatives, where ethyl- and propyl-substituted systems show elevated *κ* values, likely due to enhanced hyperconjugation in larger π systems.

The relationship between *κ* and molecular descriptors is captured by the following QSAR equation:

$$\kappa =1.277N_{r}+24.771G_{ap}+24.865N_{BO}-8.147$$

$$R^{2}=0.874$$

This model takes into account the number of aromatic rings, the HOMO–LUMO gap, and the NBO value. The coefficients indicate that both the number of rings and the NBO have a comparable and dominant effect on the tunneling factor, greater than that of the HOMO–LUMO gap. This simplified model retains strong predictive power while highlighting the primary electronic and structural drivers of tunneling behavior.

**Figure 4:**
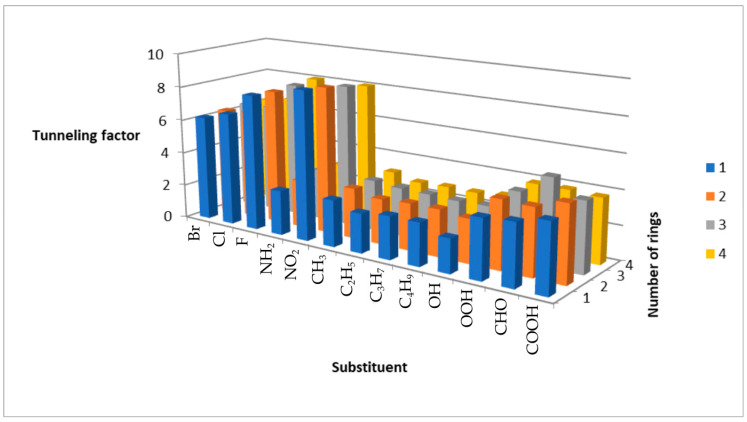
Plot of the dependence of the tunneling factor on the number of aromatic rings, as well as on the type of substituent.

**Figure 5:**
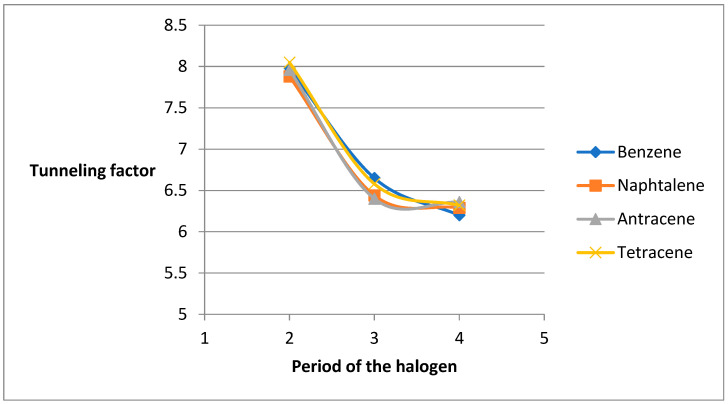

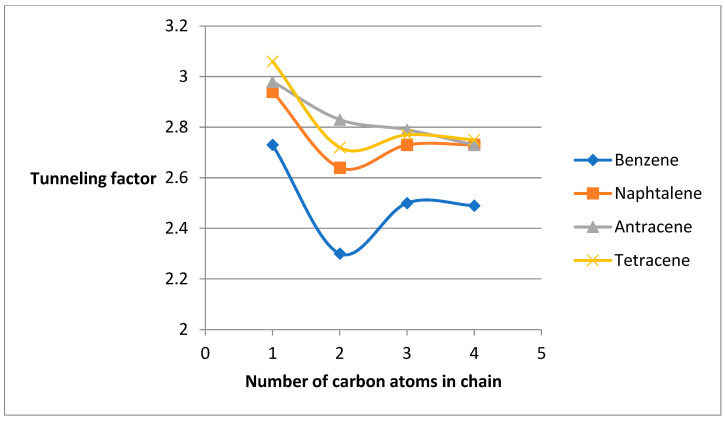
Graphs of the dependence of the tunneling factor on the period number of the attached halogen and on the number of carbon atoms in the attached chain.

For *β*-substituted PAHs, *κ* similarly decreases with increasing ring number and shows a weaker dependence on substituent effects compared to *α* derivatives (Figure 4). Halogen and nitro groups again yield the highest *κ* values, while amines and alkyl chains give the lowest. As with *α* derivatives, *κ* decreases with halogen period, with fluoro derivatives exhibiting the highest values (Figure 5). Alkyl chain-length effects are less pronounced, though methyl derivatives consistently show higher *κ* than ethyl analogues. Notably, nitro derivatives exhibit a marked decrease in *κ* with increasing ring number, contrasting with the behavior of amines.

Figure 5 shows the dependence of the tunneling factor on the period number of the halogen, as well as on the length of the attached chain:

The relationship between *κ* and molecular descriptors for *β*-derivatives is described by the following simplified QSAR equation:

$$\kappa =2.853N_{r}-3.515G_{ap}-0.047\alpha -63.104E_{max}^{R}+69.027$$

$$R^{2}=0.908$$

This model highlights the importance of the number of aromatic rings, the HOMO–LUMO gap, polarizability (*α*), and the local electron localization (*ELF^R^_max_*) in governing tunneling in *β*-substituted systems. The strong predictive power (*R*^2^ = 0.908) and simplicity of this model make it a valuable tool for estimating tunneling effects in substituted PAHs.

Both response surfaces (Figure S1 in Supporting Information—descriptors) have a similar shape: the tunneling factor changes slightly with the change in substituent, while *κ* increases with the number of rings.

### 3.2. Reactions Rate Constants

**Figure 6:**
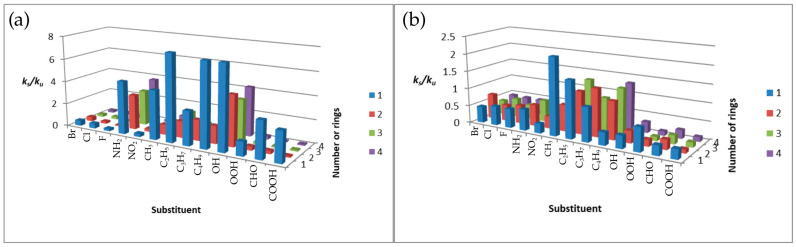
Plots of the dependence of *k_s_*/*k_u_* on the type of substituent and on the number of aromatic rings for: (**a**) 300 K (**b**) 1500 K.

The dependence of the constant rate ratio for alpha derivatives at the two analyzed temperatures is shown in Figure 6 below.

The calculated rate constant for unsubstituted benzene at 300 K $(1.08\times 10^{-14}$ cm^3^ molecule^−1^ s^−1^) agrees well with the experimental value at 298 K ((4.6 ± 2.8) × 10^−14^ cm^3^ molecule^−1^ s^−1^) [46], thus validating the computational approach despite a small 2K difference in temperature. At 1500 K, our value ($4.74\times 10^{-12}$ cm^3^ molecule^−1^ s^−1^) is comparable to the literature estimate ($2.27\times 10^{-12}$ cm^3^ molecule^−1^ s^−1^) [46]. For *α*-naphthalene, the computed rate constant at 300 K ($2.24\times 10^{-14}$ cm^3^ molecule^−1^ s^−1^) aligns with the CVT/SCT result ($1.13\times 10^{-14}$ cm^3^ molecule^−1^ s^−1^ [47]. Less comparable results were obtained for the β position, amounting to 0.19 × 10^−14^ cm^3^ molecule^−1^ s^−1^ in one study [48] and 1.40 × 10^−14^ cm^3^ molecule^−1^ s^−1^ in this work. For toluene at 300 K, our value ($4.58\times 10^{-14}$ cm^3^ molecule^−1^ s^−1^) is similar to the M06-2X/6-311+G(d,p) result ($2.04\times 10^{-14}$ cm^3^ molecule^−1^ s^−1^) [49]. Generally, the kinetic parameter values obtained in this study are typically within a factor of two of those reported in the literature, which supports the reliability of the proposed methodology. The exception is the comparison of the beta-naphthalene reaction, where the difference between the literature value and the calculated value is greater.

At 300 K, for halogen, nitro, methyl, and carbonyl derivatives, the reactions proceeded more slowly than for the unsubstituted compound. In contrast, the *k_s_*/*k_u_* for amines, hydroxyl compounds, and other alkyl derivatives was greater than unity. Exceptions are ethyl- and propyltetracene and propylanthracene. For unsubstituted acenes, the reaction rate at the *α* position decreases in the order naphthalene > tetracene > anthracene at 300 K [20]. This trend is not consistently observed for substituted derivatives, underscoring the strong influence of substituents on kinetics. In contrast, the *k_s_*/*k_u_* ratios at 1500 K are considerably lower than at 300 K. Values greater than unity are observed only for methylbenzene, ethyl derivatives (excluding ethyltetracene), propylnaphthalene, and butyl derivatives other than butylbenzene.

**Figure 7:**
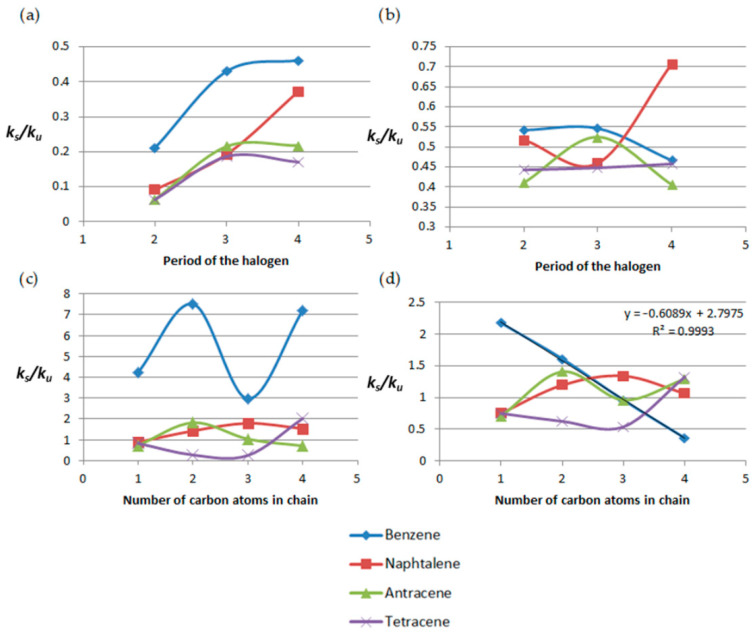
Plots of the dependence of *k_s_*/*k_u_* on the period number of the halogen at (**a**) 300 K and (**b**) 1500 K and the dependence of *k_s_*/*k_u_* on the number of carbon atoms in the attached chain at (**c**) 300 K and (**d**) 1500 K.

Figure 7 shows the dependence of $k_{s}/k_{u}$ on halogen period and alkyl-chain length for *α* derivatives. At 300 K, halogen-substituted benzenes exhibit the highest $k_{s}/k_{u}$ ratios, while other systems show comparable values except for bromonaphthalene. At 1500 K, bromonaphthalene displays the largest enhancement. Alkyl-chain effects are nonlinear at 300 K. At 1500 K, the dependence for benzene derivatives becomes linear, with $k_{s}/k_{u}$ decreasing with increasing chain length.

The QSAR models for *k_s_*/*k_u_* of *α* derivatives are:

300 K:

$$\frac{k_{s}}{k_{u}}=-10.720N_{r}+21.870f^{-}\left(C\right)-101.634f^{0}\left(C\right)+1.836H_{OMO}-118.022G_{ap}\;+0.079\alpha -26.519N_{BO}+50.773$$

$$R^{2}=0.760$$

1500 K:

$$\frac{k_{s}}{k_{u}}=0.045C_{p}-0.035S^{0}+2.695$$

$$R^{2}=0.671$$

The 300 K model is more complex, involving ring number, Fukui indices, frontier orbital energies, polarizability, and NBO. The 1500 K model simplifies to only two thermodynamic descriptors (specific heat and entropy), reflecting the reduced electronic sensitivity at high temperature.

**Figure 8:**
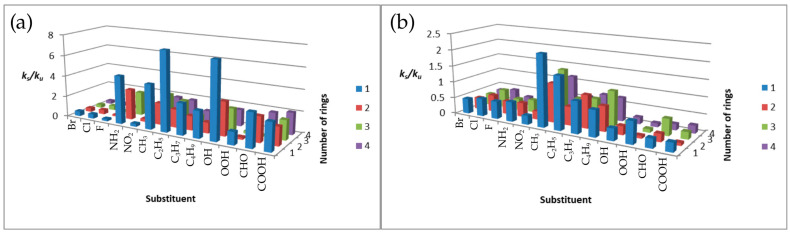
Plots of the dependence of *k_s_*/*k_u_* on the type of substituent and on the number of aromatic rings for (**a**) 300 K and (**b**) 1500 K.

Analogous analyses were performed for beta derivatives, and the results are shown in Figure 8.

At 300 K, the *k_s_*/*k_u_* values are greater than unity in the case of amines, alkyl derivatives, hydroxyl derivatives, and some carbonyl derivatives. Exceptions are propylanthracene and propyl- and butylotetracene. At 1500 K, as in the case of *α* derivatives, the *k_s_*/*k_u_* ratios are lower than those obtained at 300 K. At the higher temperature, the compounds reacting faster than their unsubstituted analogues include methyl derivatives, ethylbenzene, and butylanthracene.

The response surfaces (Figures S2 and S3 in Supporting information—descriptors) for 300 K are similar to each other. They show a peak: the highest *k_s_*/*k_u_* values are observed for a low numerical value describing the substituent and a small number of aromatic rings. As at lower temperatures, the response surfaces for 1500 K are also similar: they show a slight change in the ratio of constant rates with a change in the substituent and an increase in *k_s_*/*k_u_* with a decrease in the number of aromatic rings.

**Figure 9:**
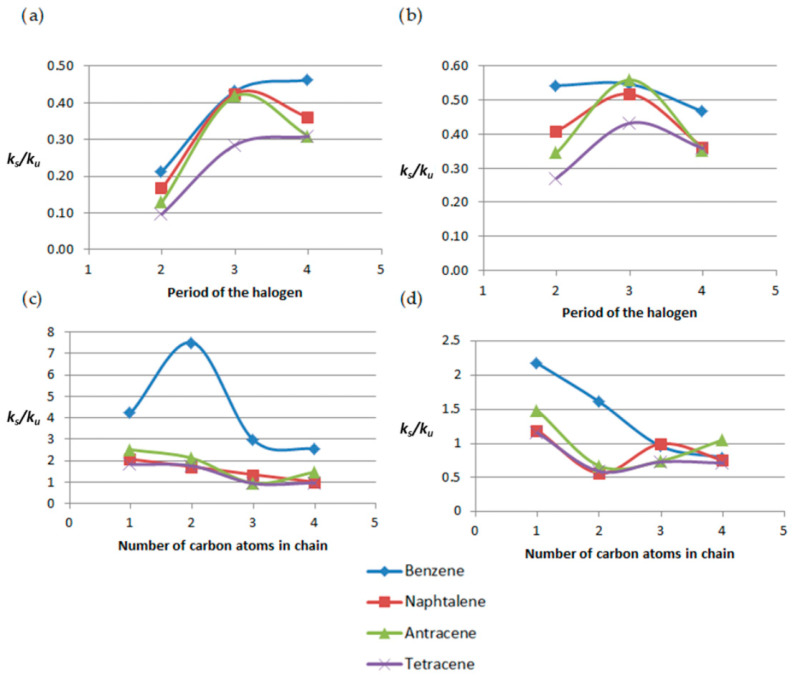
Plots of the dependence of *k_s_*/*k_u_* on the period number of the halogen at (**a**) 300 K (**b**) and 1500 K and the dependence of *k_s_*/*k_u_* on the number of carbon atoms in the attached chain at (**c**) 300 K (**d**) 1500 K.

Figure 9 presents the dependence of *k_s_*/*k_u_* on the halogen period number and the length of the attached chain for both temperature ranges for *β*-derivatives.

At 300 K, the dependence of *k_s_*/*k_u_* on the halogen period number follows a similar trend, except that the lowest values are observed for tetracene derivatives. As in the case of α derivatives, the *k_s_*/*k_u_* ratios for alkyl derivatives of benzene are higher than for the other systems, which in turn display values that are mutually comparable.

The general patterns for the derivatives of naphthalene, anthracene, and tetracene at 1500 K are identical to those at 300 K. The behavior varies for benzene derivatives, though, with only methylbenzene and ethylbenzene showing higher *k_s_*/*k_u_* values than the others. None of these dependences is linear.

Equations describing the dependence of *k_s_*/*k_u_* on the studied descriptors are presented below:

(a) 300 K:

$$\frac{k_{s}}{k_{u}}=-2.878N_{r}-14.887f^{+}\left(C\right)-54.924G_{ap}-16.340N_{BO}+24.701$$

$$R^{2}=0.788$$

(b) 1500 K:

$$\frac{k_{s}}{k_{u}}=-0.086\mu +0.020C_{p}-0.017S^{0}+1.814$$

$$R^{2}=0.731$$

The equation describing the reaction rate ratio at lower temperatures includes: the number of aromatic rings, the Fukui index describing nucleophilic attack, HOMO–LUMO gap, and NBO. As in previous cases, the number of aromatic rings has the greatest impact on the variable. In turn, the equation describing *k_s_*/*k_u_* at 1500 K takes into account the dipole moment, specific heat, and entropy. Thermodynamic parameters have a greater influence on the ratio of rate constants than the dipole moment.

In order to verify the obtained regression equations, calculations were performed for benzene derivatives (in meta position) and naphthalene derivatives (position 1) not included in the training set, and the kinetic parameter values were compared with the results obtained from the regression equations (Table 1).

The values calculated using regression equations are similar to those calculated at the M06-2X/aug-cc-pVDZ level of theory. In the case of the quotient of constant rates, the observed error is greater due to the low values assumed by *k_s_*/*k_u_*.

### 3.3. Electrophilic Properties of Substituents

In order to examine the electrophilic properties of substituents, the global electrophilic index (*ω*) was calculated, which is given by the formula:

$$\omega =\frac{\mu^{2}}{2\eta }$$

where μ denotes chemical electron potential and η denotes chemical hardness [50]:

$$\eta \approx LUMO-HOMO\;\mu \approx \frac{HOMO+LUMO}{2}$$

The results are summarized in Table 2.

Strong electrophiles are those with a *ω* value greater than 0.055 Hartree, while moderate electrophiles have values between 0.03 Hartree and 0.055 Hartree. Weak electrophiles on the other hand are characterized by a *ω* value lower than 0.03 Hartree [51].

The values of the index suggest that there are no strong electrophiles among the compounds studied. On the other hand, all benzene derivatives and some naphthalene derivatives are moderate electrophiles. The electrophilicity of the tested systems decreases with an increase in aromatic rings. A similar trend is observed for most tunneling factors for the alpha position and for *k_s_*/*k_u_* of some alpha and beta derivatives at 300 K.

In addition to the global electrophilicity index, its local equivalent at the reaction center was calculated. It is given by the formula:

$$\omega_{C}=f^{+}(C)\cdot \omega$$

The results are summarized in Table 3.

Aniline and carbonyl derivatives have the highest *ω_C_* values among benzene derivatives. At the two temperatures under investigation, these compounds exhibit moderate *k_s_*/*k_u_* values and comparatively low tunneling factors. The most electrophilic naphthalene derivatives are *α*-propylnaphthalene, *α*-butylnaphthalene, *α*-acid, and *β*-derivatives with –OH, –OOH, and –NH_2_ substituents. With the exception of the acid, all of these substances show comparatively low tunneling factors. Furthermore, compared to unsubstituted naphthalene, the reaction at 1500 K proceeds more slowly for the previously mentioned *β* derivatives. When it comes to anthracene derivatives, *α*-propyl-, *α*-butylanthracene, and *β*-fluoroanthracene exhibit the highest *ω_C_* values. Likewise, the ks/ku ratio at 1500 K is still low for β-naphthalene derivatives with -OH, -OOH, or -NH_2_ groups. The compounds with propyl and butyl substituents that have the highest local electrophilicity among tetracene derivatives are *α*-propyl- and *α*-butyl-tetracene, *α*-nitrotetracene, and *β*-tetracene. Relatively high tunneling factors are another characteristic of the *α* derivatives. At 300 K, the reaction rates for the *β*-derivatives discussed above are similar to those of the unsubstituted analogues, but they are lower at 1500 K. As the number of aromatic rings increases, electrophilicity generally tends to decrease.

### 3.4. PLS Analysis

To gain additional insight into the relationship between descriptors and tunneling factor and rate constants, PLS analysis was performed. Before proceeding, the VIP (variable importance in projection) function was calculated, and descriptors for which VIP was greater than 1 were used for further model building. The number of components was selected based on a permutation test. Regression coefficients, statistical data, and a biplot were determined for each dataset.

The results for the tunneling factor are presented in Table 4 and Table 5.

In both cases, polarizability shows the highest positive correlation. For alpha derivatives, an increase in the tunneling factor is observed with an increase in the local minimum of the ELF function within the base, NBO, and the number describing the base. In turn, a negative correlation occurs for the LUMO energy. In the case of beta derivatives, an increase in the tunneling factor is observed with an increase in the number describing the ligand and the HOMO and LUMO energies. In turn, a negative correlation occurs for the local maximum of the ELF function within the ligand and *E_SCF_*.

For both alpha and beta derivatives, the *R*^2^ coefficients are closer to unity for the MLR used in DOE. However, the *p*-values in each case are significantly less than 0.05.

**Figure 10:**
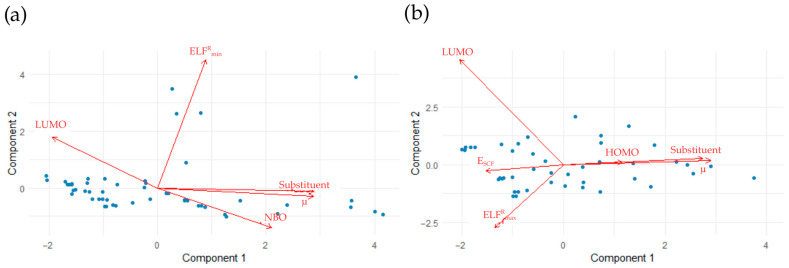
Biplots of PLS analysis in the context of the tunneling factor for (**a**) alpha derivatives and (**b**) beta derivatives. An acute angle between vectors indicates a positive correlation, a right angle indicates no correlation, and a half-angle indicates a negative correlation.

The results of the PLS analysis for the tunneling factor of alpha and beta derivatives are presented in Figure 10.

Since dipole moment was used to calculate the numerical value describing the substituent, a close correlation between them can be observed in all graphs. In the case of alpha derivatives, the substituent correlates positively with NBO and negatively with LUMO energy. On the other hand, there is a weak correlation between the local minimum of the ELF function within the substituent. In the case of beta derivatives, there is a strong positive correlation between the substituent and HOMO energy and a strong negative correlation between the substituent and *E_SCF_*.

Similar studies were conducted for the *k_s_*/*k_u_* dataset for alpha derivatives at temperatures of 300 and 1500 K (Table 6 and Table 7).

At lower temperatures, the strongest positive correlation between the ratio of constant rates and LUMO energy was observed, while a decrease in *k_s_*/*k_u_* occurs with an increase in dipole moment, polarizability, NBO, or the local minimum of the ELF function within the ligand. LUMO energy correlates most strongly with the ratio of constant rates also at 1500 K, while a negative correlation is observed for the number describing the ligand and dipole moment.

For both temperatures, the *R*^2^ and adjusted *R*^2^ coefficients are closer to unity for MLR. The *F*-statistic values are higher for DOE in both cases. The *p*-value is less than 0.05 for MLR, but greater for PLS.

**Figure 11:**
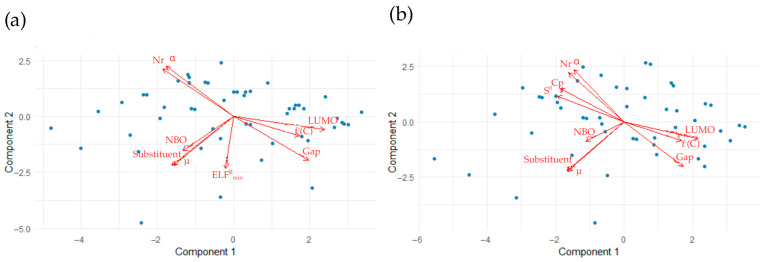
Biplots of PLS analysis for alpha derivatives in the context of ks/ku at (**a**) 300 K and (**b**) 1500 K. An acute angle between vectors indicates a positive correlation, a right angle indicates no correlation, and a half-angle indicates a negative correlation.

The results of the PLS analysis for the ratio of rate constants for alpha derivatives at the two temperatures studied are shown in Figure 11.

In both temperature cases, the variables are divided into three correlated groups. At 300 K, group 1 consists of substituent, NBO, and *ELF^R^_min_*. This group shows weak correlations with the other two groups. Group 2 consists of polarizability and the number of aromatic rings, which correlates negatively with group 3 (LUMO energy, *f^-^*(*C*), and HOMO—LUMO gap). In the case of 1500 K, group 1 consists of the substituent and NBO. As with 300 K, group 1 correlates weakly with the other groups, while groups 2 and 3 correlate negatively with each other. Group 2 consists of polarizability, number of aromatic rings, specific heat, and entropy, while the composition of group 3 is identical to that of 300 K.

Similar analyses were performed for datasets describing the ratio of constant reaction rates of beta derivatives at 300 and 1500 K, and the results are summarized in Table 8 and Table 9.

For lower temperatures, the strongest positive correlation between the ratio of constant rates and descriptors is observed for the maximum of the ELF function within the base and *E_SCF_* and a negative correlation for dipole moment, polarizability, and the number describing the substituent. For higher temperatures, a strong negative correlation between *k_s_*/*k_u_* and dipole moment and the number describing the substituent is also observed. In turn, LUMO energy is a positively correlating descriptor.

Both *R*^2^ and adjusted *R*^2^ values are closer to unity in the case of the DOE method. The *F* statistics values are higher for MLR, and the *p* values are less than 0.05.

**Figure 12:**
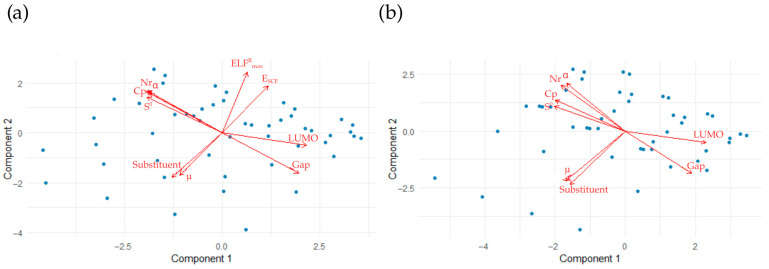
Biplots of PLS analysis for beta derivatives in the context of *k_s_*/*k_u_* at (**a**) 300 K and (**b**) 1500 K. An acute angle between vectors indicates a positive correlation, a right angle indicates no correlation, and a half-angle indicates a negative correlation.

The results of the PLS analysis for the ratio of constant beta derivative rates at the two temperatures studied are shown in Figure 12.

In the case of 300 K, the descriptors are divided into four groups. For group 1, —the substituent correlates weakly with groups 2 and 4 and correlates negatively with group 3. Additionally, groups 2 and 4 correlate negatively with each other. Group 2 consists of polarizability, number of aromatic rings, specific heat, and entropy. Group 3 consists of *ELF^R^_max_* and *E_SCF_*, and group 4 consists of LUMO and HOMO energies and the LUMO gap. For higher temperatures, the formation of three groups of variables was observed. Group 1, consisting of substituents, correlates weakly with the others, which in turn correlate negatively with each other. The composition of group 2 is identical to that at 300 K, while the composition of group 3 at 1500 K is identical to group 4 at 300 K.

For each analyzed system, a more accurate description was obtained for MLR. The *R*^2^ values are closest to unity for the tunneling factor. In turn, they deviate most from unity for *k_s_*/*k_u_* at a temperature of 1500 K, which results from a smaller discrepancy in the ratio of constant rates at a higher temperature.

Although the correlation coefficients (*R*^2^) obtained for certain MLR and PLS models—particularly those describing $k_{s}/k_{u}$ ratios at 1500 K—do not exceed 0.7, the results remain valuable and scientifically meaningful. This is primarily because radical–aromatic systems such as PAH + ·OH reactions exhibit intrinsically high structural and energetic variability, where small changes in substituent polarity, steric hindrance, or conjugation can produce nonlinear kinetic responses. Consequently, even moderate *R*^2^ values in this range capture genuine structure–reactivity trends rather than statistical noise. The lower *R*^2^ values obtained for the PLS models can be partially attributed to the different variable selection strategies applied. While MLR models were constructed using descriptors with statistically significant *p*-values (*p* < 0.05), PLS models were additionally restricted to variables with a VIP > 1. Since the VIP reflects the overall contribution of variables across latent components rather than their direct correlation with the response, this criterion may exclude descriptors that are locally important for explaining the dependent variable, leading to reduced model performance.

Moreover, the statistical significance (*p* < 0.05 for all models except PLS for rate constant ratio in 1500 K) confirms that the identified descriptors—especially those related to electronic distribution (NBO, LUMO, Fukui indices)—are not random, but represent physically interpretable parameters governing the tunneling effect and reaction barriers.

## 4. Conclusions

Despite the fact that the hydroxyl radical is one of the most important radicals present in the atmosphere, the reactions of substituted aromatic hydrocarbons have been poorly studied. Reactions with this radical have been intensively investigated, but for other types of compounds [20,52,53]. This study demonstrates that both the type of substituent attached to the aromatic ring and the number of rings significantly influence the kinetics of the hydrogen atom transfer reaction with the hydroxyl radical. The magnitude and direction of this effect depend on the substitution position (*α* or *β*). For both *α* and *β* derivatives, an increase in the tunneling factor is observed for halogen- and nitro-substituted compounds. Moreover, for both substitution types, the ratio of reaction rate constants (*k_s_*/*k_u_*) for substituted versus unsubstituted acenes decreases with increasing temperature: at higher temperatures, fewer substituents contribute to the acceleration of the reaction relative to the unsubstituted analogues.

At the lower temperature of 300 K, the fastest reactions are observed for *α*-amines, *α*-phenols, several *α*-alkyl derivatives, *β*-amines, *β*-aldehydes, and *β*-carboxylic acids. At 1500 K, the highest *k_s_*/*k_u_* values—and thus the highest rates—are obtained for selected *α*- and *β*-alkyl derivatives.

Another objective of this work was to develop a QSAR model and perform a statistical analysis of how molecular descriptors influence the tunneling factor and the *k_s_*/*k_u_* ratio. The descriptors employed in this model include parameters describing the reactants (Fukui indices), and descriptors related to the products (HOMO and LUMO energies, thermodynamic quantities, NBO values, and local minima or maxima of ELF functions within the reaction center and substituent). These variables enabled the construction of linear relationships between the studied quantities and selected descriptors, characterized by relatively high coefficients of determination (*R*^2^ ranging from 0.671 to 0.908). A more accurate fit was obtained for the tunneling factor, while a slightly weaker correlation was found for *k_s_*/*k_u_* at 1500 K, likely due to lower data variability at this temperature.

Prediction of both the tunneling factor and *k_s_*/*k_u_* proved to be more effective using the MLR-based DOE method than the PLS approach, as indicated by the statistical parameters. Nevertheless, the PLS analysis provided valuable complementary insights into the influence of individual descriptors on the dependent variables and the mutual correlations among descriptors.

Therefore, the findings of this study may contribute to a more comprehensive understanding of their reaction kinetics.

## Figures and Tables

**Table 1:** Kinetic parameters calculated at M06-2X/aug-cc-pVDZ level of theory and via regression equations for reactions from outside of the training set.

Parameter	Reactant	Value Calculated at M06-2X/aug-cc-pVDZ Level of Theory	Value Calculated from Regression Equations	Relative Error (%)
*κ*	Nitrobenzene	5.92	5.46	7.77
*k_s_*/*k_u_* (300 K)	Ethylbenzene	2.27	2.58	13.95
*k_s_*/*k_u_* (1500 K)	Naphthalene-2-carboaldehyde	0.24	0.26	10.07

**Table 2:** Values of the global electrophilicity index expressed in Hartree for the tested compounds.

Substituent	Benzene	Naphthalene Alpha	Naphthalene Beta	Anthracene Alpha	Anthracene Beta	Tetracene Alpha	Tetracene Beta
Br	0.0376	−0.0241	0.0310	0.0250	0.0250	0.0209	0.0209
Cl	0.0382	0.0309	0.0311	0.0250	0.0251	0.0209	−0.0113
F	0.0389	0.0314	−0.0257	0.0253	0.0254	0.0210	0.0211
NH_2_	0.0327	0.0296	0.0298	0.0245	0.0246	0.0207	0.0207
NO_2_	0.0352	0.0282	0.0290	0.0233	−0.0133	0.0197	0.0203
CH_3_	0.0370	−0.0266	0.0315	0.0252	0.0254	0.0210	0.0211
C_2_H_5_	0.0368	0.0311	0.0315	0.0252	0.0253	0.0211	0.0211
C_3_H_7_	0.0371	0.0311	0.0313	0.0251	0.0252	0.0195	0.0193
C_4_H_9_	0.0368	0.0311	0.0313	0.0251	0.0252	0.0195	0.0193
OH	0.0358	−0.0271	0.0306	0.0251	0.0249	0.0210	0.0208
OOH	0.0361	0.0310	0.0313	0.0250	0.0254	0.0209	0.0212
CHO	0.0346	0.0289	0.0299	0.0239	0.0239	0.0202	0.0201
COOH	0.0358	0.0294	0.0303	0.0241	0.0246	−0.0106	0.0205

**Table 3:** Values of the local electrophilicity index expressed in Hartree for the tested compounds.

Substituent	Benzene	Naphthalene Alpha	Naphthalene Beta	Anthracene Alpha	Anthracene Beta	Tetracene Alpha	Tetracene Beta
Br	0.0011	−0.0016	0.0019	0.0014	0.0013	0.0010	0.0009
Cl	0.0011	0.0021	0.0021	0.0014	0.0014	0.0010	−0.0005
F	0.0009	0.0021	−0.0022	0.0014	0.0017	0.0010	0.0011
NH_2_	0.0035	0.0013	0.0024	0.0011	0.0016	0.0008	0.0011
NO_2_	0.0019	0.0019	0.0006	0.0016	−0.0003	0.0012	0.0005
CH_3_	0.0010	−0.0016	0.0022	0.0013	0.0014	0.0009	0.0010
C_2_H_5_	0.0008	0.0019	0.0019	0.0013	0.0013	0.0009	0.0009
C_3_H_7_	0.0021	0.0024	0.0018	0.0020	0.0014	0.0014	0.0015
C_4_H_9_	0.0021	0.0024	0.0018	0.0020	0.0014	0.0014	0.0015
OH	0.0013	−0.0017	0.0026	0.0012	0.0016	0.0009	0.0011
OOH	0.0010	0.0018	0.0027	0.0012	0.0017	0.0008	0.0011
CHO	0.0024	0.0020	0.0007	0.0016	0.0006	0.0011	0.0006
COOH	0.0024	0.0022	0.0008	0.0016	0.0008	−0.0006	0.0007

**Table 4:** Regression coefficient values obtained using the PLS method for the alpha- and beta-derivative tunneling factor.

Alpha		Beta	
Descriptor	Coefficient	Descriptor	Coefficient
μ	0.731	μ	0.736
ELF^R^_min_	0.729	S	0.722
S	0.573	ELF^R^_max_	−0.644
NBO	0.356	HOMO	0.351
LUMO	−0.241	LUMO	0.190
-	-	E_SCF_	−0.141

**Table 5:** Statistical parameter values for MLR analysis included in DOE and PLS for alpha and beta derivatives in the context of the tunneling factor.

	Alpha				Beta			
	*R* ^2^	Adjusted *R*^2^	*F*—Statistics Value	*p* Value	*R* ^2^	Adjusted *R*^2^	*F*—Statistics Value	*p* Value
DOE (MLR)	0.874	0.787	9.95	1.93 × 10^−8^	0.908	0.844	14.12	2.50 × 10^−10^
PLS	0.711	0.680	22.64	2.20 × 10^−11^	0.682	0.639	16.07	8.97 × 10^−10^

**Table 6:** Regression coefficient values obtained using the PLS method for *k_s_*/*k_u_* alpha derivatives at temperatures of 300 K and 1500 K.

300 K		1500 K	
Descriptor	Coefficient	Descriptor	Coefficient
μ	−0.338	S	−0.097
NBO	−0.307	μ	−0.095
ELF^R^_min_	−0.298	LUMO	0.042
S	−0.263	S^0^	−0.038
LUMO	0.253	α	0.028
N_r_	−0.172	C_p_	−0.021
Gap	0.138	f^-^(C)	0.019
α	−0.117	N_r_	0.006
f^-^(C)	0.097	Gap	0.002
-		NBO	−0.0002

**Table 7:** Statistical parameters values for MLR analysis included in DOE and PLS for alpha derivatives for 300 and 1500 K in the context of constant rate ratios.

	300 K				1500 K			
	*R* ^2^	Adjusted *R*^2^	*F*—Statistics Value	*p* Value	*R* ^2^	Adjusted *R*^2^	*F*—Statistics Value	*p* Value
DOE (MLR)	0.760	0.591	4.52	9.64 × 10^−5^	0.671	0.440	2.91	3.75 × 10^−3^
PLS	0.394	0.265	3.04	6.88 × 10^−3^	0.324	0.159	1.96	0.064

**Table 8:** Regression coefficient values obtained using the PLS method for *k_s_*/*k_u_* beta derivatives at temperatures of 300 K and 1500 K.

300 K		1500 K	
Descriptor	Coefficient	Descriptor	Coefficient
ELF^R^max	0.298	μ	−0.088
μ	−0.263	S	−0.086
E_SCF_	0.252	LUMO	0.044
S	−0.240	S^0^	−0.031
LUMO	0.190	Cp	−0.019
Nr	−0.132	Gap	0.013
α	−0.109	α	0.011
Gap	0.086	Nr	−0.005
Cp	0.035	-	-
S^0^	0.023	-	-

**Table 9:** Statistical parameters values for MLR analysis included in DOE and PLS for beta derivatives for 300 and 1500 K in the context of constant rate ratios.

	300 K				1500 K			
	*R* ^2^	Adjusted *R*^2^	*F*—Statistics Value	*p* Value	*R* ^2^	Adjusted *R*^2^	*F*—Statistics Value	*p* Value
DOE (MLR)	0.787	0.639	5.30	2.06 × 10^−5^	0.731	0.542	3.87	3.82 × 10^−4^
PLS	0.361	0.205	2.32	0.029	0.304	0.175	2.35	0.034

## Data Availability

All data generated or analyzed during this study are included in this published article and its Supplementary Information files. Additional datasets (raw output files, input structures, etc.) are available from the corresponding author upon reasonable request.

## Supplementary Materials

The following supporting information can be downloaded at https://www.mdpi.com/article/10.3390/molecules31020265/s1. The supplementary information consists of four files: reactants, transition states, products, and descriptors. The first three contain visualizations of the reactants, transition states, and products of the reaction studied, along with geometries and vibration frequency values. All calculations were performed at the M06-2X/aug-cc-pVDZ theory level. The last file contains the values of the descriptors used to construct the QSAR model and response surfaces.
