# Supplementary material for: Substituent and Ring-Number Effects on the Kinetics of PAH + OH Reactions: A QSAR–DOE Approach with Tunneling Corrections
Source: Molecules. 2026 Jan 13;31(2):265. doi: 10.3390/molecules31020265 (PMC12843667; doi:10.3390/molecules31020265)
Supplement: Supplementary file 1 [file molecules-31-00265-s001.zip › Supporting Information - descriptors.pdf]

Supporting information - descriptors for

Substituent and Ring-Number Effects on the Kinetics of PAH + OH Reactions: A QSAR–DOE Approach with Tunneling Corrections

Cezary Parzych[1], Maciej Baradyn[2], Artur Ratkiewicz[2]*

Address: Institute of Chemistry, University of Bialystok, ul. Ciolkowskiego 1K, 15-245 Bialystok, Poland

[1] Doctoral School of University of Bialystok; 15-245 Bialystok, Ciołkowskiego 1K Street; Poland ; c.parzych@uwb.edu.pl
[2] Department of Physical Chemistry, University of Bialystok, Bialystok, Ciołkowskiego 1K Street; Poland

*Correspondence: artrat@uwb.edu.pl

Table S1 Characteristics of descriptors 3
Table S2 Values of descriptors for benzene derivatives 4
Table S3 Values of descriptors for benzene derivatives - continuation 4
Table S4 Values of descriptors for α-naphtalene derivatives 5
Table S5 Values of descriptors for α-naphtalene - continuation 5
Table S6 Values of descriptors for β-naphtalene derivatives 6
Table S7 Values of descriptors for β-naphtalene - continuation 6
Table S8 Values of descriptors for α-anthracene derivatives 7
Table S9 Values of descriptors for α-anthracene - continuation 8
Table S10 Values of descriptors for β-anthracene derivatives 8
Table S11 Values of descriptors for β-anthracene - continuation 9
Table S12 Values of descriptors for α-tetracene derivatives 9
Table S13 Values of descriptors for α-tetracene - continuation 10
Table S14 Values of descriptors for β-tetracene derivatives 11
Table S15 Values of descriptors for β-tetracene - continuation 11

Table S1 Characteristics of descriptors

Descriptor	Symbol	Unit
**Partial charge on carbon atom**	q(C)	-
**Fukui index for electrophilic attack**	f^-(C)	-
**Fukui index for nucleophilic attack**	f^+(C)	-
**Fukui index for radical attack**	f^0(C)	-
**Dual descriptor**	Δf(C)	-
**Partial charge on hydrogen atom**	q(H)	-
**Reaction energy**	ΔE	kcal/mol
**HOMO energy**	HOMO	Hartree
**LUMO energy**	LUMO	Hartree
**HOMO and LUMO gap**	HOMO - LUMO	Hartree
**Polarizability**	α	$Bohr^3$
**Dipole moment**	μ	D
**Total energy**	E_{SFC}	Hartree
**Heat capacity**	Cp	-
**Entropy**	S^0	-
**Local ELF maximum within reaction center**	$ELFC_{max}$	-
**Local ELF minimum within reaction center**	$ELFC_{min}$	-
**Local ELF maximum within substituent**	$ELFR_{max}$	-
**Local ELF minimum within substituent**	$ELFR_{min}$	-
**NBO value on reaction center**	NBO	-

Table S2 Values of descriptors for benzene derivatives

Substituent	q(C)	$f^-(C)$	$f^+(C)$	$f^0(C)$	Δf(C)	q(H)	ΔE	HOMO	LUMO	HOMO - LUMO
**-Br**	-0.044	0.064	0.029	0.046	-0.036	0.049	-4.94	-0.307	-0.0061	0.3009
**-Cl**	-0.045	0.07	0.03	0.05	-0.04	0.05	-4.52	-0.3113	-0.0054	0.3059
**-F**	-0.054	0.084	0.023	0.053	-0.06	0.054	-3.71	-0.3155	-0.0046	0.3109
**$-NH_2$**	-0.067	0.079	0.106	0.093	0.026	0.038	-5.69	-0.2677	-0.0064	0.2613
**$-NO_2$**	-0.028	0.134	0.055	0.094	-0.079	0.055	-3.48	-0.3455	-0.0643	0.2812
**$-CH_3$**	-0.049	0.073	0.026	0.05	-0.048	0.038	-6.5	-0.2985	-0.0025	0.296
**$-C_2H_5$**	0.049	0.068	0.021	0.045	-0.047	0.037	-7.28	-0.2974	-0.0029	0.2945
**$-C_3H_7$**	-0.0479	0.0886	0.0565	0.0726	-0.0321	0.0424	-6.48	-0.3001	-0.0036	0.2964
**$-C_4H_9$**	-0.0479	0.0886	0.0565	0.0726	-0.0321	0.0424	-6.48	-0.2990	-0.0044	0.2947
**-OH**	-0.07	0.081	0.036	0.059	-0.044	0.04	-5.6	-0.29	-0.004	0.2861
**-OOH**	0.058	0.085	0.029	0.057	-0.056	0.049	-4.84	-0.2955	-0.007	0.2885
**-CHO**	-0.022	0.032	0.069	0.051	0.038	0.051	-5.59	-0.3215	-0.0447	0.2768
**-COOH**	-0.024	0.071	0.068	0.07	-0.003	0.05	-5.26	-0.3186	-0.0325	0.2861

Table S3 Values of descriptors for benzene derivatives - continuation

Substituent	α	μ	E_{SFC}	Cp	S^0	$ELFC_{max}$	$ELFC_{min}$	$ELFR_{max}$	$ELFR_{min}$	NBO
**-Br**	89.03	2.29	-2805.0686	46.32	123.98	0.999994	1.36E-06	0.957755	2.27E-07	0.177
**-Cl**	81.35	2.28	-691.0802	46.24	120.92	0.999968	5.82E-07	0.966325	6.15E-07	0.176
**-F**	66.73	2.17	-330.7095	45.97	117.42	0.999924	1.62E-08	0.995233	2.13E-05	0.123
**$-NH_2$**	79.03	1.39	-286.8366	53.28	125.88	0.999967	9.31E-06	0.999727	1.33E-07	0.118
**$-NO_2$**	83.96	5.15	-435.9563	56.16	136.81	0.999986	5.96E-06	0.999988	1.65E-06	0.228
**$-CH_3$**	80.88	0.62	-270.7891	56.97	130.84	0.999888	9.06E-07	0.99998	3.85E-11	0.166
**$-C_2H_5$**	93.1	0.63	-310.0872	69.59	147.52	0.999924	2.39E-05	0.99996	2.27E-13	0.157
**$-C_3H_7$**	106.94	0.62	-349.3844	34.60	95.32	0.999830	1.15E-05	0.999982	4.62E-14	0.168
**$-C_4H_9$**	119.84	0.63	-388.6819	39.49	102.37	0.999992	2.41E-05	0.999959	1.65E-14	0.168
**-OH**	72.63	0.82	-306.7033	49.66	121.71	0.999985	5.01E-06	0.99916	8.45E-08	0.087

**-OOH**	78.35	1.78	-381.8129	55.17	135.05	0.999917	4.31E-06	0.999603	2.57E-11	0.114
**-CHO**	83.48	3.34	-344.7919	54.64	131.04	0.999973	1.70E-06	0.999979	1.08E-09	0.207
**-COOH**	86.7	2.04	-420.0398	59.88	141.14	0.999986	2.68E-05	0.999994	9.51E-09	0.224

Table S4 Values of descriptors for α-naphtalene derivatives

Substituent	q(C)	f^-(C)	f^+(C)	f^0(C)	Δf(C)	q(H)	ΔE	HOMO	LUMO	HOMO - LUMO
**-Br**	-0.046	0.061	0.067	0.064	0.007	0.049	-5.39	0.2795	-0.0337	0.2458
**-Cl**	-0.047	0.063	0.068	0.066	0.005	0.05	-4.79	-0.2797	-0.0328	0.2469
**-F**	-0.059	0.072	0.068	0.07	-0.004	0.054	-3.57	-0.2788	-0.0274	0.2514
**$-NH_2$**	0.074	0.081	0.045	0.063	-0.036	0.037	-5.67	-0.2535	-0.0171	0.2364
**$-NO_2$**	-0.027	0.051	0.068	0.059	0.017	0.055	-4.22	-0.2988	-0.0732	0.2256
**$-CH_3$**	-0.05	0.066	0.061	0.063	-0.005	0.039	-6.47	0.27	-0.0212	0.2487
**$-C_2H_5$**	-0.048	0.065	0.06	0.063	-0.005	0.039	-6.56	-0.2693	-0.0209	0.2484
**$-C_3H_7$**	-0.04	0.0706	0.0779	0.0743	0.0073	0.0426	-7.18	-0.2697	-0.0205	0.2492
**$-C_4H_9$**	-0.04	0.0706	0.0779	0.0743	0.0073	0.0426	-7.13	-0.2695	-0.0203	0.2492
**-OH**	-0.077	0.077	0.062	0.069	-0.015	0.039	-5.38	0.2636	-0.0171	0.2466
**-OOH**	-0.073	0.073	0.058	0.066	-0.015	0.038	-4.73	-0.2691	-0.0213	0.2479
**-CHO**	-0.026	0.049	0.071	0.06	0.022	0.045	-5.96	-0.2874	-0.0565	0.2309
**-COOH**	-0.027	0.051	0.075	0.063	0.025	0.044	-6.29	-0.2847	-0.0494	0.2354

Table S5 Values of descriptors for α-naphtalene - continuation

Substituent	α	μ	E_{SFC}	Cp	S^0	$ELFC_{max}$	$ELFC_{min}$	$ELFR_{max}$	$ELFR_{min}$	NBO
**-Br**	138.13	2.42	-2958.6744	74.13	160.56	0.943595	4.87E-07	0.957847	6.05E-09	0.18
**-Cl**	130.41	2.38	-844.6858	74.03	157.5	0.999976	6.28E-07	0.994089	2.81E-08	0.178
**-F**	115.97	2.21	-484.3147	73.73	153.97	0.999798	9.74E-07	0.998623	1.71E-05	0.118
**$-NH_2$**	128.23	1.17	-440.4418	81.03	161.99	0.999993	5.34E-06	0.999988	5.90E-09	0.111
**$-NO_2$**	134.12	5.47	-589.5569	83.99	177.88	0.999966	1.13E-07	0.996511	4.86E-09	0.205

**-CH$_3$**	129.86	0.64	-424.3952	84.78	165.14	0.999908	1.50E-06	0.999984	8.86E-14	0.166
**-C$_2$H$_5$**	142.24	0.61	-463.6917	97.41	182.39	0.999874	1.16E-06	0.999992	4.07E-13	0.17
**-C$_3$H$_7$**	155.86	0.46	-502.9907	46.10	104.76	0.999999	9.32E-07	0.999989	2.73E-14	0.161
**-C$_4$H$_9$**	169.09	0.51	-542.2881	51.00	112.20	0.999775	2.36E-06	0.999958	3.78E-15	0.161
**-OH**	122	0.6	-460.3091	77.41	158.03	0.999867	2.84E-06	0.999425	1.35E-08	0.078
**-OOH**	128.05	1.79	-535.4184	82.92	171.89	0.999900	1.88E-04	0.999352	4.38E-08	0.118
**-CHO**	133.01	3.49	-498.3963	82.45	167.67	0.999984	3.51E-05	0.999996	3.16E-05	0.256
**-COOH**	136.18	2.09	-573.6426	87.71	178.43	0.999972	4.89E-06	0.999993	6.47E-12	0.241

Table S6 Values of descriptors for β-naphtalene derivatives

**Substituent**	**q(C)**	**f^-(C)**	**f^+(C)**	**f^0(C)**	**Δf(C)**	**q(H)**	**ΔE**	**HOMO**	**LUMO**	**HOMO - LUMO**
**-Br**	-0.046	0.04	0.063	0.052	0.023	0.049	-4.85	-0.2811	-0.0335	0.2476
**-Cl**	-0.046	0.044	0.067	0.055	0.023	0.05	-4.46	-0.2814	-0.0324	0.249
**-F**	-0.052	0.051	0.086	0.068	0.034	0.055	-3.78	0.281	-0.0287	0.2523
**-NH$_2$**	-0.058	0.027	0.082	0.054	0.056	0.041	-5.79	-0.2524	-0.0143	0.2381
**-NO$_2$**	-0.038	0.072	0.021	0.046	-0.051	0.053	-3.45	-0.3008	-0.0688	0.232
**-CH$_3$**	-0.047	0.049	0.07	0.06	0.021	0.041	-6.41	-0.2704	-0.018	0.2523
**-C$_2$H$_5$**	-0.045	0.047	0.061	0.054	0.013	0.041	-6.68	-0.27	-0.0183	0.2517
**-C$_3$H$_7$**	-0.0479	0.0886	0.0565	0.0726	-0.0321	0.0589	-6.62	-0.2691	-0.0189	0.2502
**-C$_4$H$_9$**	-0.0479	0.0886	0.0565	0.0726	-0.0321	0.0589	-6.26	-0.2688	-0.0187	0.2501
**-OH**	-0.052	0.034	0.086	0.06	0.052	0.05	-5.24	-0.2659	-0.0206	0.2452
**-OOH**	-0.053	0.034	0.087	0.061	0.054	0.05	-4.17	-0.2713	-0.0209	0.2504
**-CHO**	-0.031	0.069	0.024	0.047	-0.044	0.05	-3.97	-0.2908	-0.0516	0.2392
**-COOH**	-0.032	0.07	0.027	0.049	-0.043	0.049	-4.1	-0.2874	-0.0454	0.2421

Table S7 Values of descriptors for β-naphtalene - continuation

**Substituent**	**α**	**μ**	**E_{SFC}**	**Cp**	**S^0**	**$ELFC_{max}$**	**$ELFC_{min}$**	**$ELFR_{max}$**	**$ELFR_{min}$**	**NBO**

**-Br**	141.63	2.52	-2958.6741	74.17	160.91	0.999962	4.26E-06	0.957737	2.02E-08	0.18
**-Cl**	132.98	2.49	-844.6857	74.08	157.86	0.999994	3.22E-06	0.966331	3.83E-08	0.179
**-F**	115.94	2.31	-484.3151	73.83	154.29	0.999961	3.49E-05	0.999214	3.68E-05	0.134
**$-NH_2$**	130.81	1.49	-440.4428	81.15	162.72	0.999909	3.02E-05	0.999829	7.28E-10	0.141
**$-NO_2$**	136.32	5.85	-589.5623	84.01	173.63	0.999963	3.50E-05	0.999929	4.08E-08	0.214
**$-CH_3$**	131.8	0.61	-424.3953	84.82	166.33	0.999965	7.27E-06	0.999995	6.19E-16	0.174
**$-C_2H_5$**	145.39	0.57	-463.6929	97.46	184.28	0.999965	7.47E-06	0.999995	5.47E-16	0.171
**$-C_3H_7$**	159.46	0.54	-502.9907	46.21	106.80	0.999649	1.03E-05	0.999999	1.86E-16	0.172
**$-C_4H_9$**	172.15	0.54	-542.2876	51.04	114.56	0.999669	2.17E-06	0.999958	9.64E-18	0.173
**-OH**	123.08	0.92	-460.3088	77.51	158.54	0.99998	4.43E-06	0.999607	1.99E-03	0.107
**-OOH**	128.76	1.86	-535.4184	82.99	171.79	0.999894	3.03E-06	0.999863	7.39E-13	0.155
**-CHO**	136.37	4.59	-498.396	82.5	167.9	0.999977	3.66E-06	0.999965	9.17E-12	0.225
**-COOH**	139.02	3.29	-573.644	87.75	178.27	0.999931	3.52E-05	0.999987	7.33E-10	0.215

Table S8 Values of descriptors for α-anthracene derivatives

Substituent	q(C)	$f^-(C)$	$f^+(C)$	$f^0(C)$	Δf(C)	q(H)	ΔE	HOMO	LUMO	HOMO - LUMO
**-Br**	-0.046	0.05	0.056	0.053	0.006	0.049	-5.46	-0.2559	-0.0558	0.2001
**-Cl**	-0.048	0.052	0.056	0.054	0.005	0.05	-4.89	-0.2555	-0.0552	0.2003
**-F**	-0.061	0.059	0.056	0.058	-0.003	0.054	-3.52	-0.2542	-0.0522	0.202
**$-NH_2$**	-0.076	0.069	0.046	0.058	-0.023	0.036	-5.6	-0.2393	-0.0431	0.1962
**$-NO_2$**	-0.025	0.041	0.068	0.055	0.027	0.055	-4.55	-0.2676	-0.0816	0.186
**$-CH_3$**	-0.05	0.053	0.051	0.052	-0.002	0.039	-6.5	-0.2472	-0.0456	0.2017
**$-C_2H_5$**	-0.048	0.053	0.05	0.052	-0.002	0.039	-6.72	-0.2466	-0.0454	0.2012
**$-C_3H_7$**	-0.04	0.0706	0.0779	0.0743	0.0073	0.0503	-6.72	-0.2463	-0.0458	0.2005
**$-C_4H_9$**	-0.04	0.0706	0.0779	0.0743	0.0073	0.0503	-7.52	-0.2464	-0.0455	0.2009
**-OH**	-0.08	0.063	0.049	0.056	-0.014	0.039	-5.27	-0.2441	-0.0435	0.2006
**-OOH**	0.076	0.06	0.048	0.054	-0.011	0.038	-2.6	-0.2526	-0.0523	0.2003
**-CHO**	-0.023	0.041	0.067	0.054	0.026	0.046	-6.06	-0.2587	-0.0676	0.1911

**-COOH**	-0.024	0.042	0.068	0.055	0.026	0.045	-6.5	-0.2561	-0.0631	0.193

Table S9 Values of descriptors for α-anthracene - continuation

Substituent	α	μ	E_{SFC}	Cp	S^0	$ELFC_{max}$	$ELFC_{min}$	$ELFR_{max}$	$ELFR_{min}$	NBO
**-Br**	198.15	2.57	-3112.2733	102.02	197.67	0.999867	1.21E-07	0.957851	5.46E-09	0.179
**-Cl**	190.22	2.52	-998.2847	101.94	194.78	0.999976	4.67E-07	0.966626	3.49E-09	0.177
**-F**	175.45	2.33	-637.9134	101.66	191.43	0.999947	9.05E-07	0.997032	1.92E-05	0.114
**$-NH_2$**	188.14	1.14	-594.0408	108.94	199.27	0.999983	2.20E-06	0.99983	7.65E-09	0.105
**$-NO_2$**	195	5.76	-743.1562	111.93	215.28	0.999483	8.51E-07	0.999416	1.71E-08	0.253
**$-CH_3$**	189.55	0.66	-577.9945	112.66	202.16	0.999979	3.43E-07	0.999961	4.16E-13	0.165
**$-C_2H_5$**	202.08	0.61	-617.2908	125.32	219.58	0.999992	4.90E-07	0.999988	2.45E-14	0.169
**$-C_3H_7$**	215.58	0.63	-656.5888	57.94	117.98	0.999890	1.64E-07	0.999995	7.81E-16	0.17
**$-C_4H_9$**	229.75	0.49	-695.8872	62.89	125.56	0.999925	1.97E-05	0.999942	7.36E-14	0.16
**-OH**	181.79	0.5	-613.9079	105.35	195.53	0.999989	2.51E-06	0.999624	1.91E-06	0.071
**-OOH**	187.2	1.97	-689.0141	110.95	208.76	0.999959	1.02E-05	0.999157	1.60E-06	0.112
**-CHO**	193.23	3.49	-651.9961	110.33	204.38	0.999850	1.70E-06	0.999981	2.07E-06	0.26
**-COOH**	198.15	2.57	-3112.2733	102.02	197.67	0.999867	1.21E-07	0.957851	5.46E-09	0.179

Table S10 Values of descriptors for β-anthracene derivatives

Substituent	q(C)	$f^-(C)$	$f^+(C)$	$f^0(C)$	Δf(C)	q(H)	ΔE	HOMO	LUMO	HOMO - LUMO
**-Br**	-0.046	0.045	0.053	0.049	0.008	0.048	-4.84	-0.2561	-0.0558	0.2003
**-Cl**	-0.046	0.046	0.055	0.051	0.009	0.05	-4.48	-0.2559	-0.055	0.2009
**-F**	-0.051	0.049	0.066	0.058	0.017	0.055	-3.86	-0.2548	-0.052	0.2028
**$-NH_2$**	-0.055	0.026	0.065	0.046	0.038	0.042	-5.89	-0.2357	-0.039	0.1966
**$-NO_2$**	-0.041	0.058	0.02	0.039	-0.038	0.052	-3.52	0.2706	-0.0782	0.1924
**$-CH_3$**	-0.046	0.047	0.056	0.052	0.009	0.041	-6.45	-0.2462	-0.0434	0.2028
**$-C_2H_5$**	-0.044	0.046	0.053	0.049	0.006	0.041	-6	-0.246	-0.0434	0.2026

**-C_3H_7**	-0.0479	0.0886	0.0565	0.0726	-0.0321	-0.1223	-6.28	-0.2452	-0.0433	0.2019
**-C_4H_9**	-0.0479	0.0886	0.0565	0.0726	-0.0321	-0.1223	-5.83	-0.2450	-0.0431	0.2019
**-OH**	-0.05	0.039	0.066	0.053	0.027	0.051	-5.11	-0.2442	-0.0449	0.1993
**-OOH**	-0.051	0.04	0.066	0.053	0.026	0.051	-4.22	-0.2483	-0.0453	0.203
**-CHO**	-0.034	0.056	0.026	0.041	-0.03	0.05	-4.3	-0.2601	-0.069	0.1911
**-COOH**	-0.035	0.057	0.031	0.044	-0.026	0.049	-4.99	-0.2588	-0.0624	0.1964

Table S11 Values of descriptors for β-anthracene - continuation

Substituent	α	μ	E_{SFC}	Cp	S^0	$ELFC_{max}$	$ELFC_{min}$	$ELFR_{max}$	$ELFR_{min}$	NBO
**-Br**	204.41	2.73	-3112.2727	102.13	198.3	0.999878	4.23E-06	0.957911	7.18E-09	0.178
**-Cl**	194.87	2.68	-998.2844	102.02	195.14	0.999563	8.25E-07	0.966464	1.21E-11	0.15
**-F**	175.4	2.45	-637.9139	101.74	191.76	0.99997	3.99E-07	0.998324	7.53E-06	0.135
**-NH_2**	193.15	1.63	-594.042	109.07	200.13	0.999028	4.65E-06	0.999694	7.81E-09	0.154
**-NO_2**	199.58	6.46	-743.1612	111.95	210.98	0.99997	7.97E-05	0.999958	1.32E-07	0.205
**-CH_3**	193.07	0.61	-577.9944	112.71	203.39	0.999506	6.27E-06	0.999945	1.05E-13	0.174
**-C_2H_5**	207.57	0.58	-617.2919	125.36	221.53	0.999985	1.35E-04	0.999979	2.21E-16	0.171
**-C_3H_7**	220.53	0.77	-656.5889	58.08	118.29	0.999935	9.00E-05	0.999984	3.73E-17	0.179
**-C_4H_9**	233.71	0.83	-695.8856	62.87	125.58	0.998164	7.76E-07	0.999956	1.39E-16	0.179
**-OH**	183.91	0.99	-613.9075	105.44	196.2	0.999959	5.41E-06	0.999004	2.08E-06	0.111
**-OOH**	189.48	1.82	-689.0176	110.94	208.77	0.999945	3.25E-05	0.999988	2.23E-11	0.158
**-CHO**	198.93	3.84	-651.9956	110.46	205.49	0.999996	9.68E-05	0.999972	3.64E-07	0.192
**-COOH**	201.93	2.51	-727.2441	115.68	215.29	0.999885	3.01E-04	0.999986	1.78E-08	0.208

Table S12 Values of descriptors for α-tetracene derivatives

Substituent	q(C)	$f^-(C)$	$f^+(C)$	$f^0(C)$	Δf(C)	q(H)	ΔE	HOMO	LUMO	HOMO - LUMO
**-Br**	-0.046	0.041	0.046	0.043	0.005	0.049	-5.55	-0.2384	-0.0713	0.1671
**-Cl**	-0.048	0.042	0.046	0.044	0.004	0.05	-4.91	-0.2381	-0.0709	0.1671

**-F**	-0.061	0.049	0.047	0.048	-0.002	0.054	-3.54	-0.237	-0.0691	0.1679
**-NH_2**	0.077	0.055	0.038	0.047	-0.017	0.036	-5.6	-0.227	-0.0613	0.1657
**-NO_2**	-0.023	0.034	0.059	0.047	0.025	0.055	-4.78	-0.2469	-0.0893	0.1576
**-CH_3**	-0.049	0.044	0.042	0.043	-0.001	0.039	-6.55	-0.2312	-0.063	0.1682
**-C_2H_5**	-0.05	0.042	0.041	0.041	-0.001	0.038	-7.36	-0.2308	-0.0624	0.1684
**-C_3H_7**	-0.0435	0.0831	0.0743	0.0787	-0.0088	0.1679	-7.52	-0.2222	-0.0664	0.1557
**-C_4H_9**	-0.0435	0.0831	0.0743	0.0787	-0.0088	0.1679	-7.49	-0.2220	-0.0663	0.1557
**-OH**	-0.081	0.051	0.041	0.046	-0.011	0.039	-5.25	-0.2295	-0.0617	0.1677
**-OOH**	0.077	0.048	0.04	0.044	-0.008	0.038	-2.78	-0.2349	-0.0678	0.1671
**-CHO**	-0.021	0.033	0.056	0.045	0.022	0.046	-6.17	-0.2394	-0.0776	0.1618
**-COOH**	-0.023	0.035	0.056	0.045	0.021	0.045	-6.49	0.2372	-0.0746	0.1627

Table S13 Values of descriptors for α-tetracene - continuation

Substituent	α	μ	E_{SFC}	Cp	S^0	$ELFC_{max}$	$ELFC_{min}$	$ELFR_{max}$	$ELFR_{min}$	NBO
**-Br**	268.61	2.71	-3265.869	129.97	235.07	0.999527	1.41E-06	0.957749	9.02E-09	0.178
**-Cl**	260.38	2.65	-1151.8803	129.91	232.29	0.999985	1.21E-06	0.966541	2.52E-08	0.176
**-F**	245.11	2.45	-791.5089	129.65	229.13	0.999807	3.96E-06	0.999828	8.66E-06	0.112
**-NH_2**	258.31	1.13	-747.6365	136.92	236.84	0.999959	3.75E-05	0.999755	2.11E-08	0.146
**-NO_2**	266.28	6	-896.752	139.88	250.09	0.999804	1.18E-07	0.999811	2.62E-12	0.207
**-CH_3**	259.57	0.68	-731.5902	140.65	239.85	0.999979	2.37E-07	0.999989	1.78E-13	0.165
**-C_2H_5**	273.15	0.55	-770.8882	153.25	256.81	0.999984	8.23E-06	0.999951	2.28E-14	0.158
**-C_3H_7**	286.95	0.44	-810.1855	69.83	130.97	0.999998	5.81E-06	0.999991	1.02E-13	0.159
**-C_4H_9**	300.96	0.48	-849.4830	74.70	138.93	0.972031	3.39E-05	0.999951	2.02E-14	0.159
**-OH**	251.75	0.44	-767.5036	133.33	233.21	0.999958	8.88E-06	0.999956	3.37E-07	0.068
**-OOH**	257.13	1.99	-842.61	138.87	245.87	0.999967	1.18E-05	0.999182	1.34E-08	0.109
**-CHO**	263.86	3.53	-805.592	138.28	241.87	0.999600	3.16E-06	0.999964	1.63E-10	0.231
**-COOH**	266.99	2.12	-880.8377	143.59	252.59	0.999937	1.56E-06	0.999977	8.59E-09	0.248

Table S14 Values of descriptors for β-tetracene derivatives

Substituent	q(C)	f⁻(C)	f⁺(C)	f⁰(C)	Δf(C)	q(H)	ΔE	HOMO	LUMO	HOMO - LUMO
**-Br**	-0.046	0.04	0.045	0.043	0.005	0.048	-4.84	-0.2383	-0.0715	0.1668
**-Cl**	-0.046	0.041	0.046	0.044	0.006	0.049	-4.5	0.2381	-0.0709	0.1672
**-F**	-0.05	0.043	0.053	0.048	0.011	0.055	-3.92	-0.2369	-0.0685	0.1685
**$-NH_2$**	-0.053	0.029	0.052	0.04	0.023	0.042	-5.92	-0.2224	-0.057	0.1654
**$-NO_2$**	-0.043	0.047	0.025	0.036	-0.022	0.052	-3.61	-0.2502	-0.0882	0.162
**$-CH_3$**	-0.045	0.041	0.046	0.044	0.005	0.041	-6.48	-0.2299	-0.0613	0.1686
**$-C_2H_5$**	-0.044	0.041	0.044	0.042	0.003	0.041	-6.87	-0.2297	-0.0612	0.1685
**$-C_3H_7$**	-0.0419	0.0448	0.079	0.0619	0.0342	0.0503	-6.83	-0.2201	-0.0657	0.1544
**$-C_4H_9$**	-0.0419	0.0448	0.079	0.0619	0.0342	0.0503	-7.17	-0.2199	-0.0655	0.1543
**-OH**	-0.049	0.037	0.053	0.045	0.016	0.051	-5.09	-0.2288	-0.0622	0.1666
**-OOH**	-0.05	0.038	0.053	0.045	0.015	0.051	-4.27	-0.2318	-0.0628	0.1691
**-CHO**	-0.035	0.047	0.029	0.038	-0.017	0.049	-4.3	-0.2416	-0.0807	0.1609
**-COOH**	-0.036	0.047	0.032	0.039	-0.015	0.048	-5.02	-0.2404	-0.0761	0.1643

Table S15 Values of descriptors for β-tetracene - continuation

Substituent	α	μ	E_{SFC}	Cp	S^0	$ELFC_{max}$	$ELFC_{min}$	$ELFR_{max}$	$ELFR_{min}$	NBO
**-Br**	277.13	2.9	-3265.8684	130.04	235.63	0.999986	1.82E-06	0.95801	2.23E-10	0.177
**-Cl**	266.69	2.84	-1151.8801	129.97	232.67	0.999606	2.47E-06	0.966371	1.80E-09	0.177
**-F**	245.01	2.57	-791.5095	129.74	229.51	0.999996	2.29E-07	0.999462	1.00E-05	0.136
**$-NH_2$**	265.6	1.78	-747.6379	137.04	237.79	0.999949	1.08E-06	0.999493	6.94E-12	0.154
**$-NO_2$**	273.23	6.98	-896.757	139.88	248.38	0.991576	1.97E-07	0.999995	2.93E-09	0.183
**$-CH_3$**	264.31	0.61	-731.59	140.71	240.95	0.999327	9.48E-07	0.999942	4.44E-13	0.174
**$-C_2H_5$**	279.63	0.59	-770.8875	153.35	259.01	0.99987	5.68E-06	0.999992	5.05E-17	0.171
**$-C_3H_7$**	295.04	0.57	-810.1853	69.96	132.76	0.999961	3.32E-06	0.99995	1.59E-17	0.173
**$-C_4H_9$**	309.10	0.62	-849.4828	74.86	140.50	0.999877	2.58E-06	0.999961	1.61E-19	0.173
**-OH**	254.83	1.04	-767.5033	133.42	233.88	0.999354	3.54E-06	0.99948	1.80E-08	0.113

**-OOH**	260.32	1.8	-842.6136	138.85	246.42	0.999878	1.94E-05	0.999936	2.04E-12	0.138
**-CHO**	272.24	4.09	-805.5913	138.36	242.78	0.999933	4.37E-05	0.999967	2.27E-13	0.213
**-COOH**	274.97	2.75	-880.8398	143.59	252.65	0.999995	1.59E-03	0.999995	1.21E-09	0.22
