# Supplementary material for: Substituent and Ring-Number Effects on the Kinetics of PAH + OH Reactions: A QSAR–DOE Approach with Tunneling Corrections
Source: Molecules. 2026 Jan 13;31(2):265. doi: 10.3390/molecules31020265 (PMC12843667; doi:10.3390/molecules31020265)
Supplement: Supplementary file 1 [file molecules-31-00265-s001.zip › Supporting Information - products.pdf]

Supporting information - products for

Substituent and Ring-Number Effects on the Kinetics of PAH + OH Reactions: A QSAR–DOE Approach with Tunneling Corrections

Cezary Parzych[1], Maciej Baradyn[2], Artur Ratkiewicz[2*]

Address: Institute of Chemistry, University of Bialystok, ul. Ciolkowskiego 1K, 15-245 Bialystok, Poland

[1] Doctoral School of University of Bialystok; 15-245 Bialystok, Ciołkowskiego 1K Street; Poland ; c.parzych@uwb.edu.pl
[2] Department of Physical Chemistry, University of Bialystok, Bialystok, Ciołkowskiego 1K Street; Poland

*Correspondence: artrat@uwb.edu.pl

Figure S1 Visualization of the optimized structure of benzene radical, calculated at the M06-2X/cc-pVTZ level of theory. 18
Table S1 Geometry (Å) of benzene radical, calculated at the M06-2X/cc-pVDZ level of theory 18
Table S2 Frequencies (cm^{-1}) of benzene radical, calculated at the M06-2X/ccpVDZ level of theory. 18
Figure S2 Visualization of the optimized structure of bromobenzene radical, calculated at the M06-2X/cc-pVDZ level of theory. 19
Table S3 Geometry (Å) of bromobenzene radical, calculated at the M06-2X/cc-pVDZ level of theory 19
Table S4 Frequencies (cm^{-1}) of bromobenzene radical, calculated at the M06-2X/ccpVDZ level of theory. 19
Figure S3 Visualization of the optimized structure of chlorobenzene radical, calculated at the M06-2X/cc-pVDZ level of theory. 20
Table S5 Geometry (Å) of chlorobenzene radical, calculated at the M06-2X/cc-pVDZ level of theory 20
Table S6 Frequencies (cm^{-1}) of chlorobenzene radical, calculated at the M06-2X/ccpVDZ level of theory. 20
Figure S4 Visualization of the optimized structure of fluorobenzene radical, calculated at the M06-2X/cc-pVDZ level of theory. 21
Table S7 Geometry (Å) of chlorobenzene radical, calculated at the M06-2X/cc-pVDZ level of theory 21
Table S8 Frequencies (cm^{-1}) of chlorobenzene radical, calculated at the M06-2X/ccpVDZ level of theory. 21
Figure S5 Visualization of the optimized structure of aminobenzene radical, calculated at the M06-2X/cc-pVDZ level of theory. 22
Table S9 Geometry (Å) of aminobenzene radical, calculated at the M06-2X/cc-pVDZ level of theory 22
Table S10 Frequencies (cm^{-1}) of aminobenzene radical, calculated at the M06-2X/ccpVDZ level of theory. 22
Figure S6 Visualization of the optimized structure of nitrobenzene radical, calculated at the M06-2X/cc-pVDZ level of theory 23
Table S11 Geometry (Å) of nitrobenzene radical, calculated at the M06-2X/cc-pVDZ level of theory 23
Table S12 Frequencies (cm^{-1}) of nitrobenzene radical, calculated at the M06-2X/ccpVDZ level of theory. 23

Figure S7 Visualization of the optimized structure of methylbenzene radical, calculated at the M06-2X/cc-pVDZ level of theory. 24

Table S13 Geometry (Å) of methylbenzene radical, calculated at the M06-2X/cc-pVDZ level of theory 24

Table S14 Frequencies (cm^{-1}) of methylbenzene radical, calculated at the M06-2X/ccpVDZ level of theory. 24

Figure S8 Visualization of the optimized structure of ethylbenzene radical, calculated at the M06-2X/cc-pVDZ level of theory. 25

Table S15 Geometry (Å) of ethylbenzene radical, calculated at the M06-2X/cc-pVDZ level of theory 25

Table S16 Frequencies (cm^{-1}) of ethylbenzene radical, calculated at the M06-2X/ccpVDZ level of theory. 25

Figure S9 Visualization of the optimized structure of propylbenzene radical, calculated at the M06-2X/cc-pVDZ level of theory. 26

Table S17 Geometry (Å) of propylbenzene radical, calculated at the M06-2X/cc-pVDZ level of theory 26

Table S18 Frequencies (cm^{-1}) of propylbenzene radical, calculated at the M06-2X/ccpVDZ level of theory. 26

Figure S10 Visualization of the optimized structure of butylbenzene radical, calculated at the M06-2X/cc-pVDZ level of theory. 27

Table S19 Geometry (Å) of butylbenzene radical, calculated at the M06-2X/cc-pVDZ level of theory 27

Table S20 Frequencies (cm^{-1}) of butylbenzene radical, calculated at the M06-2X/ccpVDZ level of theory. 28

Figure S11 Visualization of the optimized structure of hydroxybenzene radical, calculated at the M06-2X/cc-pVDZ level of theory. 28

Table S21 Geometry (Å) of hydroxybenzene radical, calculated at the M06-2X/cc-pVDZ level of theory 28

Table S22 Frequencies (cm^{-1}) of hydroxybenzene radical, calculated at the M06-2X/ccpVDZ level of theory. 28

Figure S12 Visualization of the optimized structure of peroxybenzene radical, calculated at the M06-2X/cc-pVDZ level of theory. 29

Table S23 Geometry (Å) of peroxybenzene radical, calculated at the M06-2X/cc-pVDZ level of theory 29

Table S24 Frequencies (cm^{-1}) of peroxybenzene radical, calculated at the M06-2X/ccpVDZ level of theory. 29

Figure S13 Visualization of the optimized structure of benzaldehyde radical, calculated at the M06-2X/cc-pVDZ level of theory. 30

Table S25 Geometry (Å) of benzaldehyde radical, calculated at the M06-2X/cc-pVDZ level of theory 30

Table S26 Frequencies (cm^{-1}) of benzaldehyde radical, calculated at the M06-2X/ccpVDZ level of theory. 30

Figure S14 Visualization of the optimized structure of benzoic acid radical, calculated at the M06-2X/cc-pVDZ level of theory. 31

Table S27 Geometry (Å) of benzoic acid radical, calculated at the M06-2X/cc-pVDZ level of theory 31

Table S28 Frequencies (cm^{-1}) of benzaldehyde radical, calculated at the M06-2X/ccpVDZ level of theory. 31

Figure S15 Visualization of the optimized structure of naphtalene radical, calculated at the M06-2X/cc-pVDZ level of theory. 32

Table S29 Geometry (Å) of naphtalene radical, calculated at the M06-2X/cc-pVDZ level of theory 32

Table S30 Frequencies (cm^{-1}) of naphtalene radical, calculated at the M06-2X/ccpVDZ level of theory. 32

Figure S16 Visualization of the optimized structure of α-bromonaphtalene radical, calculated at the M06-2X/cc-pVDZ level of theory. 33

Table S31 Geometry (Å) of α-bromonaphtalene radical, calculated at the M06-2X/cc-pVDZ level of theory 33

Table S32 Frequencies (cm^{-1}) of α-bromonaphtalene radical, calculated at the M06-2X/ccpVDZ level of theory. 33

Figure S17 Visualization of the optimized structure of α-chloronaphtalene radical, calculated at the M06-2X/cc-pVDZ level of theory. 34

Table S33 Geometry (Å) of α-chloronaphtalene radical, calculated at the M06-2X/cc-pVDZ level of theory 34

Table S34 Frequencies (cm^{-1}) of α-chloronaphtalene radical, calculated at the M06-2X/ccpVDZ level of theory. 34

Figure S18 Visualization of the optimized structure of α-fluoronaphtalene radical, calculated at the M06-2X/cc-pVDZ level of theory. 35

Table S35 Geometry (Å) of α-fluoronaphtalene radical, calculated at the M06-2X/cc-pVDZ level of theory 35

Table S36 Frequencies (cm^{-1}) of α-fluoronaphtalene radical, calculated at the M06-2X/ccpVDZ level of theory. 35

Figure S19 Visualization of the optimized structure of α-aminonaphtalene radical, calculated at the M06-2X/cc-pVDZ level of theory. 36

Table S37 Geometry (Å) of α-aminonaphtalene radical, calculated at the M06-2X/cc-pVDZ level of theory 36

Table S38 Frequencies (cm^{-1}) of α-aminonaphtalene radical, calculated at the M06-2X/ccpVDZ level of theory. 36

Figure S20 Visualization of the optimized structure of α-nitronaphtalene radical, calculated at the M06-2X/cc-pVDZ level of theory. 37

Table S39 Geometry (Å) of α-nitronaphtalene radical, calculated at the M06-2X/cc-pVDZ level of theory 37

Table S40 Frequencies (cm^{-1}) of α-nitronaphtalene radical, calculated at the M06-2X/ccpVDZ level of theory. 37

Figure S21 Visualization of the optimized structure of α-methylnaphtalene radical, calculated at the M06-2X/cc-pVDZ level of theory. 38

Table S41 Geometry (Å) of α-methylnaphtalene radical, calculated at the M06-2X/cc-pVDZ level of theory 38

Table S42 Frequencies (cm^{-1}) of α-methylnaphtalene radical, calculated at the M06-2X/ccpVDZ level of theory. 38

Figure S22 Visualization of the optimized structure of α-ethylnaphtalene radical, calculated at the M06-2X/cc-pVDZ level of theory. 39

Table S43 Geometry (Å) of α-ethylnaphtalene radical, calculated at the M06-2X/cc-pVDZ level of theory 39

Table S44 Frequencies (cm^{-1}) of α-ethylnaphtalene radical, calculated at the M06-2X/ccpVDZ level of theory. 40

Figure S23 Visualization of the optimized structure of α-propylnaphtalene radical, calculated at the M06-2X/cc-pVDZ level of theory. 40

Table S45 Geometry (Å) of α-propylnaphtalene radical, calculated at the M06-2X/cc-pVDZ level of theory 40

Table S46 Frequencies (cm^{-1}) of α-propylnaphtalene radical, calculated at the M06-2X/ccpVDZ level of theory. 41

Figure S24 Visualization of the optimized structure of α-butylnaphtalene radical, calculated at the M06-2X/cc-pVDZ level of theory. 41

Table S47 Geometry (Å) of α-butylnaphtalene radical, calculated at the M06-2X/cc-pVDZ level of theory 41

Table S48 Frequencies (cm^{-1}) of α-butylnaphtalene radical, calculated at the M06-2X/ccpVDZ level of theory. 42

Figure S25 Visualization of the optimized structure of α-hydroxynaphtalene radical, calculated at the M06-2X/cc-pVDZ level of theory. 43

Table S49 Geometry (Å) of α-hydroxynaphtalene radical, calculated at the M06-2X/cc-pVDZ level of theory 43

Table S50 Frequencies (cm^{-1}) of α-hydroxynaphtalene radical, calculated at the M06-2X/ccpVDZ level of theory. 43

Figure S26 Visualization of the optimized structure of α-peroxynaphtalene radical, calculated at the M06-2X/cc-pVDZ level of theory. 44

Table S51 Geometry (Å) of α-peroxynaphtalene radical, calculated at the M06-2X/cc-pVDZ level of theory 44

Table S52 Frequencies (cm^{-1}) of α-peroxynaphtalene radical, calculated at the M06-2X/ccpVDZ level of theory. 44

Figure S27 Visualization of the optimized structure of α-naphtaldehyde radical, calculated at the M06-2X/cc-pVDZ level of theory. 45

Table S53 Geometry (Å) of α-naphtaldehyde radical, calculated at the M06-2X/cc-pVDZ level of theory 45

Table S54 Frequencies (cm^{-1}) of α-naphtaldehyde radical, calculated at the M06-2X/ccpVDZ level of theory. 45

Figure S28 Visualization of the optimized structure of α-naphtalenic acid radical, calculated at the M06-2X/cc-pVDZ level of theory. 46

Table S55 Geometry (Å) of α-naphtalenic aicd radical, calculated at the M06-2X/cc-pVDZ level of theory 46

Table S56 Frequencies (cm^{-1}) of α-naphtalenic acid radical, calculated at the M06-2X/ccpVDZ level of theory. 46

Figure S29 Visualization of the optimized structure of β-bromonaphtalene radical, calculated at the M06-2X/cc-pVDZ level of theory. 47

Table S57 Geometry (Å) of β-bromonaphtalene radical, calculated at the M06-2X/cc-pVDZ level of theory 47

Table S58 Frequencies (cm^{-1}) of β-bromonaphtalene radical, calculated at the M06-2X/ccpVDZ level of theory. 47

Figure S30 Visualization of the optimized structure of β-chloronaphtalene radical, calculated at the M06-2X/cc-pVDZ level of theory. 48

Table S59 Geometry (Å) of β-chloronaphtalene radical, calculated at the M06-2X/cc-pVDZ level of theory 48

Table S60 Frequencies (cm^{-1}) of β-chloronaphtalene radical, calculated at the M06-2X/ccpVDZ level of theory. 48

Figure S31 Visualization of the optimized structure of β-fluoronaphtalene radical, calculated at the M06-2X/cc-pVDZ level of theory. 49
Table S61 Geometry (Å) of β-fluoronaphtalene radical, calculated at the M06-2X/cc-pVDZ level of theory 49
Table S62 Frequencies (cm^{-1}) of β-fluoronaphtalene radical, calculated at the M06-2X/ccpVDZ level of theory. 49
Figure S32 Visualization of the optimized structure of β-aminonaphtalene radical, calculated at the M06-2X/cc-pVDZ level of theory. 50
Table S63 Geometry (Å) of β-aminonaphtalene radical, calculated at the M06-2X/cc-pVDZ level of theory 50
Table S64 Frequencies (cm^{-1}) of β-aminonaphtalene radical, calculated at the M06-2X/ccpVDZ level of theory. 50
Figure S33 Visualization of the optimized structure of β-nitronaphtalene radical, calculated at the M06-2X/cc-pVDZ level of theory. 51
Table S65 Geometry (Å) of β-nitronaphtalene radical, calculated at the M06-2X/cc-pVDZ level of theory 51
Table S66 Frequencies (cm^{-1}) of β-nitronaphtalene radical, calculated at the M06-2X/ccpVDZ level of theory. 51
Figure S34 Visualization of the optimized structure of β-methylnaphtalene radical, calculated at the M06-2X/cc-pVDZ level of theory. 52
Table S67 Geometry (Å) of β-methylnaphtalene radical, calculated at the M06-2X/cc-pVDZ level of theory 52
Table S68 Frequencies (cm^{-1}) of β-methylnaphtalene radical, calculated at the M06-2X/ccpVDZ level of theory. 52
Figure S35 Visualization of the optimized structure of β-ethylnaphtalene radical, calculated at the M06-2X/cc-pVDZ level of theory. 53
Table S69 Geometry (Å) of β-ethylnaphtalene radical, calculated at the M06-2X/cc-pVDZ level of theory 53
Table S70 Frequencies (cm^{-1}) of β-ethylnaphtalene radical, calculated at the M06-2X/ccpVDZ level of theory. 53
Figure S36 Visualization of the optimized structure of β-propylnaphtalene radical, calculated at the M06-2X/cc-pVDZ level of theory. 54
Table S71 Geometry (Å) of β-propylnaphtalene radical, calculated at the M06-2X/cc-pVDZ level of theory 54
Table S72 Frequencies (cm^{-1}) of β-propylnaphtalene radical, calculated at the M06-2X/ccpVDZ level of theory. 55

Figure S37 Visualization of the optimized structure of β-butylnaphtalene radical, calculated at the M06-2X/cc-pVDZ level of theory. 55
Table S73 Geometry (Å) of β-butylnaphtalene radical, calculated at the M06-2X/cc-pVDZ level of theory 55
Table S74 Frequencies (cm^{-1}) of β-butylnaphtalene radical, calculated at the M06-2X/ccpVDZ level of theory. 56
Figure S38 Visualization of the optimized structure of β-hydroxynaphtalene radical, calculated at the M06-2X/cc-pVDZ level of theory. 56
Table S75 Geometry (Å) of β-hydroxynaphtalene radical, calculated at the M06-2X/cc-pVDZ level of theory 56
Table S76 Frequencies (cm^{-1}) of β-hydroxynaphtalene radical, calculated at the M06-2X/ccpVDZ level of theory. 57
Figure S39 Visualization of the optimized structure of β-peroxynaphtalene radical, calculated at the M06-2X/cc-pVDZ level of theory. 57
Table S77 Geometry (Å) of β-peroxynaphtalene radical, calculated at the M06-2X/cc-pVDZ level of theory 57
Table S78 Frequencies (cm^{-1}) of β-peroxynaphtalene radical, calculated at the M06-2X/ccpVDZ level of theory. 58
Figure S40 Visualization of the optimized structure of β-naphtaldehyde radical, calculated at the M06-2X/cc-pVDZ level of theory. 58
Table S79 Geometry (Å) of β-naphtaldehyde radical, calculated at the M06-2X/cc-pVDZ level of theory 58
Table S80 Frequencies (cm^{-1}) of β-naphtaldehyde radical, calculated at the M06-2X/ccpVDZ level of theory. 59
Figure S41 Visualization of the optimized structure of β-naphtalenic acid radical, calculated at the M06-2X/cc-pVDZ level of theory. 59
Table S81 Geometry (Å) of β-naphtalenic aicd radical, calculated at the M06-2X/cc-pVDZ level of theory 59
Table S82 Frequencies (cm^{-1}) of β-naphtalenic acid radical, calculated at the M06-2X/ccpVDZ level of theory. 60
Figure S42 Visualization of the optimized structure of antracene radical, calculated at the M06-2X/cc-pVDZ level of theory 60
Table S83 Geometry (Å) of antracene radical, calculated at the M06-2X/cc-pVDZ level of theory 60
Table S84 Frequencies (cm^{-1}) of antracene radical, calculated at the M06-2X/ccpVDZ level of theory. 61

Figure S43 Visualization of the optimized structure of α-bromoantracene radical, calculated at the M06-2X/cc-pVDZ level of theory. 61
Table S85 Geometry (Å) of α-bromoantracene radical, calculated at the M06-2X/cc-pVDZ level of theory 62
Table S86 Frequencies (cm^{-1}) of α-bromoantracene radical, calculated at the M06-2X/ccpVDZ level of theory. 62
Figure S44 Visualization of the optimized structure of α-chloroantracene radical, calculated at the M06-2X/cc-pVDZ level of theory. 63
Table S87 Geometry (Å) of α-chloroantracene radical, calculated at the M06-2X/cc-pVDZ level of theory 63
Table S88 Frequencies (cm^{-1}) of α-chloroantracene radical, calculated at the M06-2X/ccpVDZ level of theory. 63
Figure S45 Visualization of the optimized structure of α-fluoroantracene radical, calculated at the M06-2X/cc-pVDZ level of theory. 64
Table S89 Geometry (Å) of α-fluoroantracene radical, calculated at the M06-2X/cc-pVDZ level of theory 64
Table S90 Frequencies (cm^{-1}) of α-fluoroantracene radical, calculated at the M06-2X/ccpVDZ level of theory. 64
Figure S46 Visualization of the optimized structure of α-aminoantracene radical, calculated at the M06-2X/cc-pVDZ level of theory. 65
Table S91 Geometry (Å) of α-aminoantracene radical, calculated at the M06-2X/cc-pVDZ level of theory 65
Table S92 Frequencies (cm^{-1}) of α-aminoantracene radical, calculated at the M06-2X/ccpVDZ level of theory. 66
Figure S47 Visualization of the optimized structure of α-nitroantracene radical, calculated at the M06-2X/cc-pVDZ level of theory. 66
Table S93 Geometry (Å) of α-nitroantracene radical, calculated at the M06-2X/cc-pVDZ level of theory 66
Table S94 Frequencies (cm^{-1}) of α-nitroantracene radical, calculated at the M06-2X/ccpVDZ level of theory. 67
Figure S48 Visualization of the optimized structure of α-methylantracene radical, calculated at the M06-2X/cc-pVDZ level of theory. 67
Table S95 Geometry (Å) of α-methylantracene radical, calculated at the M06-2X/cc-pVDZ level of theory 67
Table S96 Frequencies (cm^{-1}) of α-methylantracene radical, calculated at the M06-2X/ccpVDZ level of theory. 68

Figure S49 Visualization of the optimized structure of α-ethylantracene radical, calculated at the M06-2X/cc-pVDZ level of theory. 69

Table S97 Geometry (Å) of α-ethylantracene radical, calculated at the M06-2X/cc-pVDZ level of theory 69

Table S98 Frequencies (cm^{-1}) of α-ethylantracene radical, calculated at the M06-2X/ccpVDZ level of theory. 69

Figure S50 Visualization of the optimized structure of α-propylantracene radical, calculated at the M06-2X/cc-pVDZ level of theory. 70

Table S99 Geometry (Å) of α-propylantracene radical, calculated at the M06-2X/cc-pVDZ level of theory 70

Table S100 Frequencies (cm^{-1}) of α-propylantracene radical, calculated at the M06-2X/ccpVDZ level of theory. 71

Figure S51 Visualization of the optimized structure of α-butylantracene radical, calculated at the M06-2X/cc-pVDZ level of theory. 71

Table S101 Geometry (Å) of α-butylantracene radical, calculated at the M06-2X/cc-pVDZ level of theory 71

Table S102 Frequencies (cm^{-1}) of α-butylantracene radical, calculated at the M06-2X/ccpVDZ level of theory. 72

Figure S52 Visualization of the optimized structure of α-hydroxyantracene radical, calculated at the M06-2X/cc-pVDZ level of theory. 73

Table S103 Geometry (Å) of α-hydroxyantracene radical, calculated at the M06-2X/cc-pVDZ level of theory 73

Table S104 Frequencies (cm^{-1}) of α-hydroxyantracene radical, calculated at the M06-2X/ccpVDZ level of theory. 73

Figure S53 Visualization of the optimized structure of α-peroxyantracene radical, calculated at the M06-2X/cc-pVDZ level of theory. 74

Table S105 Geometry (Å) of α-peroxyantracene radical, calculated at the M06-2X/cc-pVDZ level of theory 74

Table S106 Frequencies (cm^{-1}) of α-peroxyantracene radical, calculated at the M06-2X/ccpVDZ level of theory. 75

Figure S54 Visualization of the optimized structure of α-antraldehyde radical, calculated at the M06-2X/cc-pVDZ level of theory. 75

Table S107 Geometry (Å) of α-antraldehyde radical, calculated at the M06-2X/cc-pVDZ level of theory 75

Table S108 Frequencies (cm^{-1}) of α-antraldehyde radical, calculated at the M06-2X/ccpVDZ level of theory. 76

Figure S55 Visualization of the optimized structure of α-antracenic acid radical, calculated at the M06-2X/cc-pVDZ level of theory. 76

Table S109 Geometry (Å) of α-antracenic acid radical, calculated at the M06-2X/cc-pVDZ level of theory 76

Table S110 Frequencies (cm^{-1}) of α-antracenic acid radical, calculated at the M06-2X/ccpVDZ level of theory. 77

Figure S56 Visualization of the optimized structure of β-bromoantracene radical, calculated at the M06-2X/cc-pVDZ level of theory. 78

Table S111 Geometry (Å) of β-bromoantracene radical, calculated at the M06-2X/cc-pVDZ level of theory 78

Table S112 Frequencies (cm^{-1}) of β-bromoantracene radical, calculated at the M06-2X/ccpVDZ level of theory. 78

Figure S57 Visualization of the optimized structure of β-chloroantracene radical, calculated at the M06-2X/cc-pVDZ level of theory. 79

Table S113 Geometry (Å) of β-chloroantracene radical, calculated at the M06-2X/cc-pVDZ level of theory 79

Table S114 Frequencies (cm^{-1}) of β-chloroantracene radical, calculated at the M06-2X/ccpVDZ level of theory. 79

Figure S58 Visualization of the optimized structure of β-fluoroantracene radical, calculated at the M06-2X/cc-pVDZ level of theory. 80

Table S115 Geometry (Å) of β-fluoroantracene radical, calculated at the M06-2X/cc-pVDZ level of theory 80

Table S116 Frequencies (cm^{-1}) of β-fluoroantracene radical, calculated at the M06-2X/ccpVDZ level of theory. 80

Figure S59 Visualization of the optimized structure of β-aminoantracene radical, calculated at the M06-2X/cc-pVDZ level of theory. 81

Table S117 Geometry (Å) of β-aminoantracene radical, calculated at the M06-2X/cc-pVDZ level of theory 81

Table S118 Frequencies (cm^{-1}) of β-aminoantracene radical, calculated at the M06-2X/ccpVDZ level of theory. 82

Figure S60 Visualization of the optimized structure of β-nitroantracene radical, calculated at the M06-2X/cc-pVDZ level of theory. 82

Table S119 Geometry (Å) of β-nitroantracene radical, calculated at the M06-2X/cc-pVDZ level of theory 82

Table S120 Frequencies (cm^{-1}) of β-nitroantracene radical, calculated at the M06-2X/ccpVDZ level of theory. 83

Figure S61 Visualization of the optimized structure of α β-methylantracene radical, calculated at the M06-2X/cc-pVDZ level of theory. ... 83
Table S121 Geometry (Å) of β-methylantracene radical, calculated at the M06-2X/cc-pVDZ level of theory ... 83
Table S122 Frequencies (cm^{-1}) of β-methylantracene radical, calculated at the M06-2X/ccpVDZ level of theory. ... 84
Figure S62 Visualization of the optimized structure of β-ethylantracene radical, calculated at the M06-2X/cc-pVDZ level of theory. ... 84
Table S123 Geometry (Å) of β-ethylantracene radical, calculated at the M06-2X/cc-pVDZ level of theory ... 84
Table S124 Frequencies (cm^{-1}) of β-ethylantracene radical, calculated at the M06-2X/ccpVDZ level of theory. ... 85
Figure S63 Visualization of the optimized structure of β-propylantracene radical, calculated at the M06-2X/cc-pVDZ level of theory. ... 86
Table S125 Geometry (Å) of β-propylantracene radical, calculated at the M06-2X/cc-pVDZ level of theory ... 86
Table S126 Frequencies (cm^{-1}) of β-propylantracene radical, calculated at the M06-2X/ccpVDZ level of theory. ... 87
Figure S64 Visualization of the optimized structure of β-butylantracene radical, calculated at the M06-2X/cc-pVDZ level of theory. ... 87
Table S127 Geometry (Å) of β-butylantracene radical, calculated at the M06-2X/cc-pVDZ level of theory ... 87
Table S128 Frequencies (cm^{-1}) of β-butylantracene radical, calculated at the M06-2X/ccpVDZ level of theory. ... 88
Figure S65 Visualization of the optimized structure of β-hydroxyantracene radical, calculated at the M06-2X/cc-pVDZ level of theory. ... 89
Table S129 Geometry (Å) of β-hydroxyantracene radical, calculated at the M06-2X/cc-pVDZ level of theory ... 89
Table S130 Frequencies (cm^{-1}) of β-hydroxyantracene radical, calculated at the M06-2X/ccpVDZ level of theory. ... 89
Figure S66 Visualization of the optimized structure of β-peroxyantracene radical, calculated at the M06-2X/cc-pVDZ level of theory. ... 90
Table S131 Geometry (Å) of β-peroxyantracene radical, calculated at the M06-2X/cc-pVDZ level of theory ... 90
Table S132 Frequencies (cm^{-1}) of β-peroxyantracene radical, calculated at the M06-2X/ccpVDZ level of theory. ... 91

Figure S67 Visualization of the optimized structure of β-antraldehyde radical, calculated at the M06-2X/cc-pVDZ level of theory. 91
Table S133 Geometry (Å) of β-antraldehyde radical, calculated at the M06-2X/cc-pVDZ level of theory 91
Table S134 Frequencies (cm^{-1}) of β-antraldehyde radical, calculated at the M06-2X/ccpVDZ level of theory. 92
Figure S68 Visualization of the optimized structure of β-antracenic acid radical, calculated at the M06-2X/cc-pVDZ level of theory. 92
Table S135 Geometry (Å) of β-antracenic acid radical, calculated at the M06-2X/cc-pVDZ level of theory 92
Table S136 Frequencies (cm^{-1}) of β-antracenic acid radical, calculated at the M06-2X/ccpVDZ level of theory. 93
Figure S69 Visualization of the optimized structure of tetracene radical, calculated at the M06-2X/cc-pVDZ level of theory. 93
Table S137 Geometry (Å) of tetracene radical, calculated at the M06-2X/cc-pVDZ level of theory 94
Table S138 Frequencies (cm^{-1}) of tetracene radical, calculated at the M06-2X/ccpVDZ level of theory. 94
Figure S70 Visualization of the optimized structure of α-bromotetracene radical, calculated at the M06-2X/cc-pVDZ level of theory. 95
Table S139 Geometry (Å) of α-bromotetracene radical, calculated at the M06-2X/cc-pVDZ level of theory 95
Table S140 Frequencies (cm^{-1}) of α-bromotetracene radical, calculated at the M06-2X/ccpVDZ level of theory. 96
Figure S71 Visualization of the optimized structure of α-chlorotetracene acid radical, calculated at the M06-2X/cc-pVDZ level of theory. 96
Table S141 Geometry (Å) of α-chlorotetracene radical, calculated at the M06-2X/cc-pVDZ level of theory 96
Table S142 Frequencies (cm^{-1}) of α-chlorotetracene radical, calculated at the M06-2X/ccpVDZ level of theory. 97
Figure S72 Visualization of the optimized structure of α-fluorotetracene radical, calculated at the M06-2X/cc-pVDZ level of theory. 97
Table S143 Geometry (Å) of α-fluorotetracene radical, calculated at the M06-2X/cc-pVDZ level of theory 97
Table S144 Frequencies (cm^{-1}) of α-fluorotetracene radical, calculated at the M06-2X/ccpVDZ level of theory. 98

Figure S73 Visualization of the optimized structure of α-aminotetracene radical, calculated at the M06-2X/cc-pVDZ level of theory. 99

Table S145 Geometry (Å) of α-aminotetracene radical, calculated at the M06-2X/cc-pVDZ level of theory 99

Table S146 Frequencies (cm^{-1}) of α-aminotetracene radical, calculated at the M06-2X/ccpVDZ level of theory. 100

Figure S74 Visualization of the optimized structure of α-nitrotetracene radical, calculated at the M06-2X/cc-pVDZ level of theory. 100

Table S147 Geometry (Å) of α-nitrotetracene radical, calculated at the M06-2X/cc-pVDZ level of theory 100

Table S148 Frequencies (cm^{-1}) of α-nitrotetracene radical, calculated at the M06-2X/ccpVDZ level of theory. 101

Figure S75 Visualization of the optimized structure of α-methyltetracene radical, calculated at the M06-2X/cc-pVDZ level of theory. 101

Table S149 Geometry (Å) of α-methyltetracene radical, calculated at the M06-2X/cc-pVDZ level of theory 101

Table S150 Frequencies (cm^{-1}) of α-methyltetracene radical, calculated at the M06-2X/ccpVDZ level of theory. 102

Figure S76 Visualization of the optimized structure of α-ethyltetracene radical, calculated at the M06-2X/cc-pVDZ level of theory. 103

Table S151 Geometry (Å) of α-ethyltetracene radical, calculated at the M06-2X/cc-pVDZ level of theory 103

Table S152 Frequencies (cm^{-1}) of α-ethyltetracene radical, calculated at the M06-2X/ccpVDZ level of theory. 104

Figure S77 Visualization of the optimized structure of α-propyltetracene radical, calculated at the M06-2X/cc-pVDZ level of theory. 104

Table S153 Geometry (Å) of α-propyltetracene radical, calculated at the M06-2X/cc-pVDZ level of theory 104

Table S154 Frequencies (cm^{-1}) of α-propyltetracene radical, calculated at the M06-2X/ccpVDZ level of theory. 105

Figure S78 Visualization of the optimized structure of α-butyltetracene radical, calculated at the M06-2X/cc-pVDZ level of theory. 106

Table S155 Geometry (Å) of α-butyltetracene radical, calculated at the M06-2X/cc-pVDZ level of theory 106

Table S156 Frequencies (cm^{-1}) of α-butyltetracene radical, calculated at the M06-2X/ccpVDZ level of theory. 107

Figure S79 Visualization of the optimized structure of α-hydroxytetracene radical, calculated at the M06-2X/cc-pVDZ level of theory. 108

Table S157 Geometry (Å) of α-hydroxytetracene radical, calculated at the M06-2X/cc-pVDZ level of theory 108

Table S158 Frequencies (cm^{-1}) of α-hydroxytetracene radical, calculated at the M06-2X/ccpVDZ level of theory. 108

Figure S80 Visualization of the optimized structure of α-peroxytetracene radical, calculated at the M06-2X/cc-pVDZ level of theory. 109

Table S159 Geometry (Å) of α-peroxytetracene radical, calculated at the M06-2X/cc-pVDZ level of theory 109

Table S160 Frequencies (cm^{-1}) of α-peroxytetracene radical, calculated at the M06-2X/ccpVDZ level of theory. 110

Figure S81 Visualization of the optimized structure of α-tetraldehyde radical, calculated at the M06-2X/cc-pVDZ level of theory. 110

Table S161 Geometry (Å) of α-tetraldehyde radical, calculated at the M06-2X/cc-pVDZ level of theory 110

Table S162 Frequencies (cm^{-1}) of α-tetraldehyde radical, calculated at the M06-2X/ccpVDZ level of theory. 111

Figure S82 Visualization of the optimized structure of α-tetracenic acid radical, calculated at the M06-2X/cc-pVDZ level of theory. 112

Table S163 Geometry (Å) of α-tetracenic acid radical, calculated at the M06-2X/cc-pVDZ level of theory 112

Table S164 Frequencies (cm^{-1}) of α-tetracenic acid radical, calculated at the M06-2X/ccpVDZ level of theory. 112

Figure S83 Visualization of the optimized structure of β-bromotetracene radical, calculated at the M06-2X/cc-pVDZ level of theory. 113

Table S165 Geometry (Å) of β-bromotetracene radical, calculated at the M06-2X/cc-pVDZ level of theory 113

Table S166 Frequencies (cm^{-1}) of β-bromotetracene radical, calculated at the M06-2X/ccpVDZ level of theory. 114

Figure S84 Visualization of the optimized structure of β-chlorotetracene acid radical, calculated at the M06-2X/cc-pVDZ level of theory. 114

Table S167 Geometry (Å) of β-chlorotetracene radical, calculated at the M06-2X/cc-pVDZ level of theory 114

Table S168 Frequencies (cm^{-1}) of β-chlorotetracene radical, calculated at the M06-2X/ccpVDZ level of theory. 115

Figure S85 Visualization of the optimized structure of β-fluorotetracene radical, calculated at the M06-2X/cc-pVDZ level of theory. 115

Table S169 Geometry (Å) of β-fluorotetracene radical, calculated at the M06-2X/cc-pVDZ level of theory 115

Table S170 Frequencies (cm^{-1}) of β-fluorotetracene radical, calculated at the M06-2X/ccpVDZ level of theory. 116

Figure S86 Visualization of the optimized structure of β-aminotetracene radical, calculated at the M06-2X/cc-pVDZ level of theory. 117

Table S171 Geometry (Å) of β-aminotetracene radical, calculated at the M06-2X/cc-pVDZ level of theory 117

Table S172 Frequencies (cm^{-1}) of β-aminotetracene radical, calculated at the M06-2X/ccpVDZ level of theory. 118

Figure S87 Visualization of the optimized structure of β-nitrotetracene radical, calculated at the M06-2X/cc-pVDZ level of theory. 118

Table S173 Geometry (Å) of β-nitrotetracene radical, calculated at the M06-2X/cc-pVDZ level of theory 118

Table S174 Frequencies (cm^{-1}) of β-nitrotetracene radical, calculated at the M06-2X/ccpVDZ level of theory. 119

Figure S88 Visualization of the optimized structure of β-methyltetracene radical, calculated at the M06-2X/cc-pVDZ level of theory. 119

Table S175 Geometry (Å) of β-methyltetracene radical, calculated at the M06-2X/cc-pVDZ level of theory 119

Table S176 Frequencies (cm^{-1}) of β-methyltetracene radical, calculated at the M06-2X/ccpVDZ level of theory. 120

Figure S89 Visualization of the optimized structure of β-ethyltetracene radical, calculated at the M06-2X/cc-pVDZ level of theory. 121

Table S177 Geometry (Å) of β-ethyltetracene radical, calculated at the M06-2X/cc-pVDZ level of theory 121

Table S178 Frequencies (cm^{-1}) of β-ethyltetracene radical, calculated at the M06-2X/ccpVDZ level of theory. 122

Figure S90 Visualization of the optimized structure of β-propyltetracene radical, calculated at the M06-2X/cc-pVDZ level of theory. 122

Table S179 Geometry (Å) of β-propyltetracene radical, calculated at the M06-2X/cc-pVDZ level of theory 122

Table S180 Frequencies (cm^{-1}) of β-propyltetracene radical, calculated at the M06-2X/ccpVDZ level of theory. 123

Figure S91 Visualization of the optimized structure of β-butyltetracene radical, calculated at the M06-2X/cc-pVDZ level of theory. 124
Table S181 Geometry (Å) of β-butyltetracene radical, calculated at the M06-2X/cc-pVDZ level of theory 124
Table S182 Frequencies (cm^{-1}) of β-butyltetracene radical, calculated at the M06-2X/ccpVDZ level of theory. 125
Figure S92 Visualization of the optimized structure of β-hydroxytetracene radical, calculated at the M06-2X/cc-pVDZ level of theory. 125
Table S183 Geometry (Å) of β-hydroxytetracene radical, calculated at the M06-2X/cc-pVDZ level of theory 125
Table S184 Frequencies (cm^{-1}) of β-hydroxytetracene radical, calculated at the M06-2X/ccpVDZ level of theory. 126
Figure S93 Visualization of the optimized structure of α-peroxytetracene radical, calculated at the M06-2X/cc-pVDZ level of theory. 127
Table S185 Geometry (Å) of β-peroxytetracene radical, calculated at the M06-2X/cc-pVDZ level of theory 127
Table S186 Frequencies (cm^{-1}) of β-peroxytetracene radical, calculated at the M06-2X/ccpVDZ level of theory. 128
Figure S94 Visualization of the optimized structure of β-tetraldehyde radical, calculated at the M06-2X/cc-pVDZ level of theory. 128
Table S187 Geometry (Å) of β-tetraldehyde radical, calculated at the M06-2X/cc-pVDZ level of theory 128
Table S188 Frequencies (cm^{-1}) of β-tetraldehyde radical, calculated at the M06-2X/ccpVDZ level of theory. 129
Figure S95 Visualization of the optimized structure of α-tetracenic acid radical, calculated at the M06-2X/cc-pVDZ level of theory. 129
Table S189 Geometry (Å) of β-tetracenic acid radical, calculated at the M06-2X/cc-pVDZ level of theory 129
Table S190 Frequencies (cm^{-1}) of β-tetracenic acid radical, calculated at the M06-2X/ccpVDZ level of theory. 130

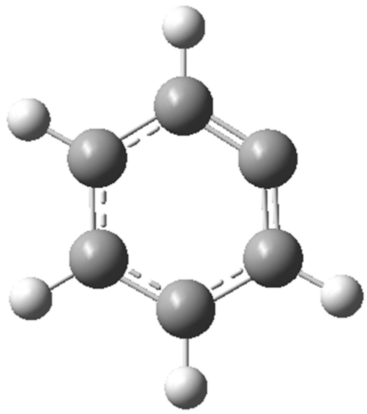

Figure S1 Visualization of the optimized structure of benzene radical, calculated at the M06-2X/cc-pVTZ level of theory.

Table S1 Geometry (Å) of benzene radical, calculated at the M06-2X/cc-pVDZ level of theory

Atom	x	y	z
C	-4.842	-1.548	0.444
C	-3.779	-1.823	-0.389
C	-3.010	-0.883	-1.040
C	-3.344	0.461	-0.831
C	-4.412	0.798	0.003
C	-5.159	-0.196	0.638
H	-2.179	-1.163	-1.687
H	-2.767	1.244	-1.323
H	-5.415	-2.335	0.932
H	-4.665	1.845	0.160
H	-5.991	0.075	1.287

Table S2 Frequencies (cm^{-1}) of benzene radical, calculated at the M06-2X/ccpVDZ level of theory.

401	424	594	610	682	726	834	904
976	979	1006	1036	1058	1080	1163	1169
1295	1331	1462	1477	1615	1668	3201	3206
3217	3219	3230					

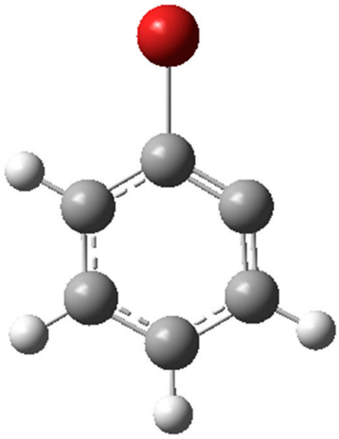

Figure S2 Visualization of the optimized structure of bromobenzene radical, calculated at the M06-2X/cc-pVDZ level of theory.

Table S3 Geometry (Å) of bromobenzene radical, calculated at the M06-2X/cc-pVDZ level of theory

Atom	x	y	z
**C**	-4.744	-1.479	0.493
**C**	-3.702	-1.884	-0.311
**C**	-3.026	-0.886	-1.025
**C**	-3.417	0.449	-0.904
**C**	-4.480	0.816	-0.078
**C**	-5.155	-0.177	0.635
**H**	-3.414	-2.931	-0.390
**H**	-2.194	-1.156	-1.674
**H**	-2.887	1.220	-1.461
**H**	-4.786	1.857	0.017
**Br**	-6.608	0.256	1.775

Table S4 Frequencies (cm^{-1}) of bromobenzene radical, calculated at the M06-2X/ccpVDZ level of theory.

167	243	321	414	480	613	673	686
754	863	959	967	1006	1049	1105	1130
1164	1245	1329	1450	1464	1606	1663	3210
3219	3226	3235					

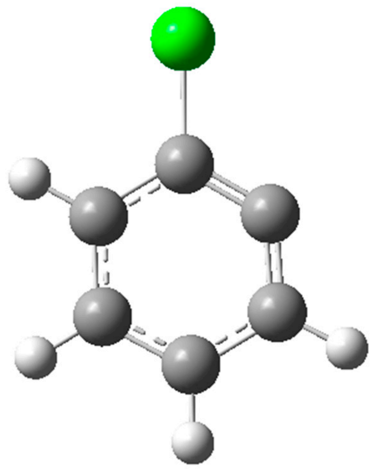

Figure S3 Visualization of the optimized structure of chlorobenzene radical, calculated at the M06-2X/cc-pVDZ level of theory.

Table S5 Geometry (Å) of chlorobenzene radical, calculated at the M06-2X/cc-pVDZ level of theory

Atom	x	y	z
C	-4.744	-1.482	0.493
C	-3.701	-1.884	-0.312
C	-3.025	-0.886	-1.025
C	-3.417	0.449	-0.904
C	-4.480	0.814	-0.077
C	-5.156	-0.179	0.635
H	-3.412	-2.931	-0.392
H	-2.194	-1.156	-1.675
H	-2.888	1.220	-1.461
H	-4.790	1.853	0.021
Cl	-6.491	0.228	1.682

Table S6 Frequencies (cm^{-1}) of chlorobenzene radical, calculated at the M06-2X/ccpVDZ level of theory.

186	286	416	416	491	614	690	709
754	862	957	975	1004	1050	1118	1137
1165	1244	1330	1453	1466	1610	1666	3210
3218	3226	3235					

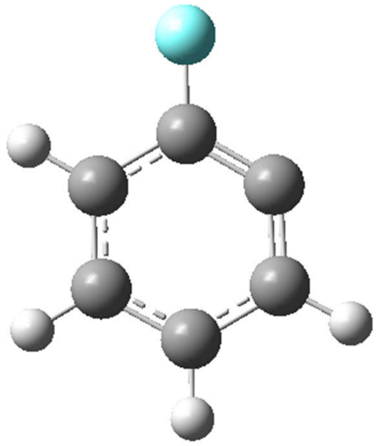

Figure S4 Visualization of the optimized structure of fluorobenzene radical, calculated at the M06-2X/cc-pVDZ level of theory.

Table S7 Geometry (Å) of chlorobenzene radical, calculated at the M06-2X/cc-pVDZ level of theory

Atom	x	y	z
C	-4.744	-1.490	0.494
C	-3.701	-1.884	-0.312
C	-3.024	-0.885	-1.026
C	-3.417	0.448	-0.904
C	-4.481	0.812	-0.076
C	-5.149	-0.183	0.631
H	-3.410	-2.930	-0.394
H	-2.193	-1.155	-1.675
H	-2.889	1.220	-1.460
H	-4.800	1.847	0.031
F	-6.177	0.145	1.434

Table S8 Frequencies (cm^{-1}) of chlorobenzene radical, calculated at the M06-2X/ccpVDZ level of theory.

235	399	423	511	522	614	690	760
827	860	952	987	998	1046	1121	1157
1239	1284	1339	1464	1494	1628	1682	3209
3219	3226	3237					

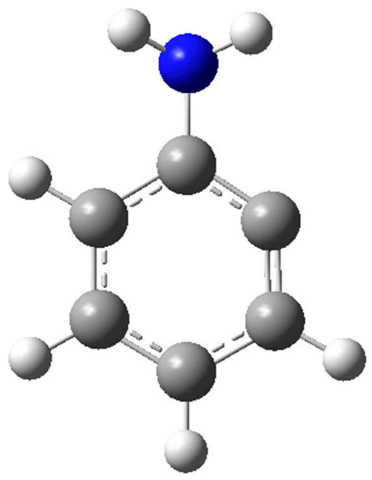

Figure S5 Visualization of the optimized structure of aminobenzene radical, calculated at the M06-2X/cc-pVDZ level of theory.

Table S9 Geometry (Å) of aminobenzene radical, calculated at the M06-2X/cc-pVDZ level of theory

Atom	x	y	z
C	-4.770	-1.479	0.448
C	-3.708	-1.878	-0.325
C	-3.002	-0.886	-1.022
C	-3.406	0.444	-0.902
C	-4.492	0.802	-0.104
C	-5.215	-0.174	0.605
H	-3.421	-2.927	-0.397
H	-2.152	-1.155	-1.647
H	-2.866	1.222	-1.440
H	-4.787	1.849	-0.017
N	-6.266	0.163	1.460
H	-6.758	1.010	1.207
H	-6.900	-0.601	1.655

Table S10 Frequencies (cm^{-1}) of aminobenzene radical, calculated at the M06-2X/ccpVDZ level of theory.

217	278	367	411	512	522	597	623
700	750	835	849	937	981	988	1052
1070	1144	1160	1269	1320	1347	1474	1501
1611	1628	1686	3189	3205	3217	3232	3584
3694							

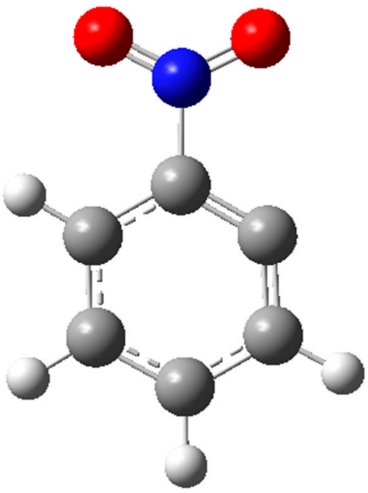

Figure S6 Visualization of the optimized structure of nitrobenzene radical, calculated at the M06-2X/cc-pVDZ level of theory.

Table S11 Geometry (Å) of nitrobenzene radical, calculated at the M06-2X/cc-pVDZ level of theory

Atom	x	y	z
C	-4.702	-1.583	0.480
C	-3.661	-1.921	-0.352
C	-3.030	-0.872	-1.036
C	-3.452	0.449	-0.867
C	-4.512	0.755	-0.015
C	-5.136	-0.290	0.662
H	-3.338	-2.953	-0.478
H	-2.200	-1.094	-1.706
H	-2.950	1.250	-1.406
N	-6.258	-0.021	1.573
H	-4.863	1.774	0.137
O	-6.756	-0.977	2.132
O	-6.609	1.135	1.707

Table S12 Frequencies (cm^{-1}) of nitrobenzene radical, calculated at the M06-2X/ccpVDZ level of theory.

66	168	230	391	417	458	538	610
678	692	742	811	878	883	980	989
1017	1048	1124	1158	1188	1255	1351	1466
1474	1479	1624	1677	1713	3214	3226	3234
3245							

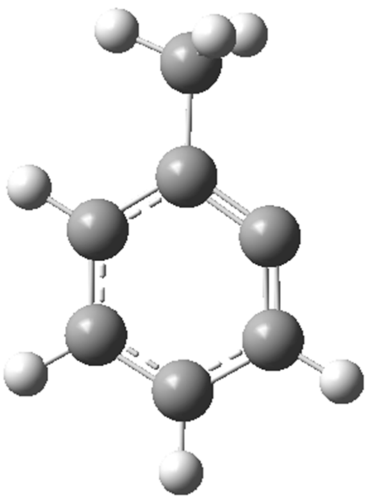

Figure S7 Visualization of the optimized structure of methylbenzene radical, calculated at the M06-2X/cc-pVDZ level of theory.

Table S13 Geometry (Å) of methylbenzene radical, calculated at the M06-2X/cc-pVDZ level of theory

Atom	x	y	z
C	-4.857	-1.477	0.330
C	-3.823	-1.889	-0.478
C	-3.020	-0.886	-1.042
C	-3.295	0.453	-0.768
C	-4.362	0.806	0.062
C	-5.185	-0.170	0.644
H	-3.630	-2.942	-0.676
H	-2.188	-1.156	-1.690
H	-2.674	1.233	-1.205
C	-6.342	0.180	1.543
H	-6.426	1.266	1.661
H	-7.284	-0.199	1.128
H	-6.212	-0.269	2.535
H	-4.564	1.858	0.267

Table S14 Frequencies (cm^{-1}) of methylbenzene radical, calculated at the M06-2X/ccpVDZ level of theory.

58	207	331	416	484	515	624	691
747	803	862	948	982	999	999	1047
1056	1134	1160	1230	1276	1331	1394	1444
1462	1480	1490	1615	1677	3069	3139	3158
3190	3204	3216	3228				

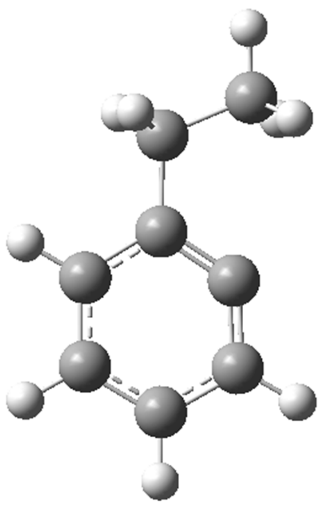

Figure S8 Visualization of the optimized structure of ethylbenzene radical, calculated at the M06-2X/cc-pVDZ level of theory.

Table S15 Geometry (Å) of ethylbenzene radical, calculated at the M06-2X/cc-pVDZ level of theory

Atom	x	y	z
C	-4.909	-1.471	0.225
C	-3.819	-1.884	-0.512
C	-2.978	-0.884	-1.011
C	-3.267	0.458	-0.750
C	-4.385	0.811	0.003
C	-5.253	-0.167	0.520
H	-3.617	-2.937	-0.701
H	-2.102	-1.154	-1.600
H	-2.612	1.237	-1.138
H	-4.598	1.863	0.199
C	-6.469	0.201	1.338
H	-6.140	0.789	2.206
H	-7.103	0.869	0.738
C	-7.277	-1.006	1.803
H	-6.662	-1.669	2.424
H	-8.146	-0.689	2.390
H	-7.632	-1.589	0.944

Table S16 Frequencies (cm^{-1}) of ethylbenzene radical, calculated at the M06-2X/ccpVDZ level of theory.

40	178	198	268	406	418	488	536
624	693	740	779	789	866	955	980
996	1002	1053	1083	1097	1137	1158	1222
1247	1276	1331	1358	1394	1455	1467	1473
1481	1494	1614	1678	3064	3068	3097	3143
3153	3186	3205	3216	3228			

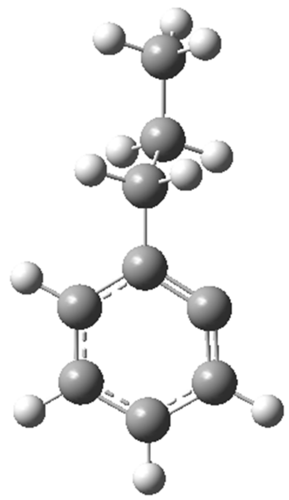

Figure S9 Visualization of the optimized structure of propylbenzene radical, calculated at the M06-2X/cc-pVDZ level of theory.

Table S17 Geometry (Å) of propylbenzene radical, calculated at the M06-2X/cc-pVDZ level of theory

Atom	x	y	z
C	0.474	0.543	0.360
C	1.846	0.445	0.398
C	2.574	1.583	0.020
C	1.897	2.737	-0.372
C	0.501	2.772	-0.391
C	-0.259	1.653	-0.020
H	2.352	-0.470	0.706
H	3.663	1.559	0.031
H	2.461	3.620	-0.668
H	-0.016	3.682	-0.703
C	-1.766	1.677	0.017
H	-2.133	2.467	-0.653
H	-2.157	0.721	-0.355
C	-2.300	1.921	1.433
H	-1.898	2.874	1.805
H	-1.911	1.135	2.095
C	-3.825	1.939	1.474
H	-4.193	2.114	2.492
H	-4.224	2.733	0.829
H	-4.235	0.983	1.124

Table S18 Frequencies (cm^{-1}) of propylbenzene radical, calculated at the M06-2X/ccpVDZ level of theory.

22	88	103	243	277	313	335	418
510	586	625	716	732	753	826	863
870	911	955	986	1001	1052	1069	1074
1116	1138	1159	1221	1234	1272	1298	1310
1337	1372	1392	1456	1465	1469	1476	1484

1487	1615	1675	3058	3058	3068	3094	3116
3133	3144	3187	3204	3216	3228		

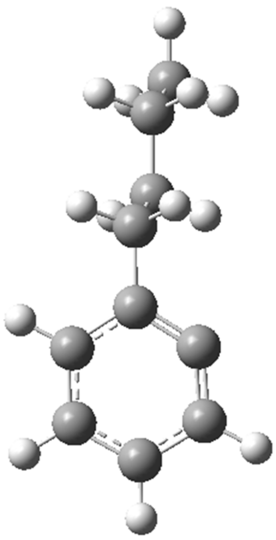

Figure S10 Visualization of the optimized structure of butylbenzene radical, calculated at the M06-2X/cc-pVDZ level of theory.

Table S19 Geometry (Å) of butylbenzene radical, calculated at the M06-2X/cc-pVDZ level of theory

Atom	x	y	z
C	0.708	-0.147	0.458
C	2.080	-0.244	0.514
C	2.813	0.852	0.036
C	2.141	1.967	-0.465
C	0.746	2.003	-0.496
C	-0.019	0.924	-0.027
H	2.581	-1.128	0.908
H	3.902	0.827	0.056
H	2.710	2.817	-0.839
H	0.233	2.880	-0.895
C	-1.526	0.957	-0.002
H	-1.887	1.662	-0.764
H	-1.918	-0.036	-0.261
C	-2.068	1.369	1.371
H	-1.668	2.360	1.632
H	-1.686	0.668	2.128
C	-3.592	1.398	1.414
H	-3.961	2.093	0.645
H	-3.978	0.404	1.148
C	-4.130	1.811	2.781
H	-3.774	2.814	3.053
H	-5.226	1.825	2.794
H	-3.791	1.113	3.559

Table S20 Frequencies (cm-1) of butylbenzene radical, calculated at the M06-2X/ccpVDZ level of theory.

23	78	80	116	214	243	286	333
391	420	523	581	625	717	721	755
785	830	868	914	924	957	986	1002
1043	1054	1077	1089	1128	1140	1158	1215
1228	1255	1274	1301	1313	1336	1348	1389
1395	1456	1462	1467	1476	1477	1486	1488
1614	1675	3047	3055	3059	3068	3080	3098
3120	3133	3142	3185	3203	3215	3229	

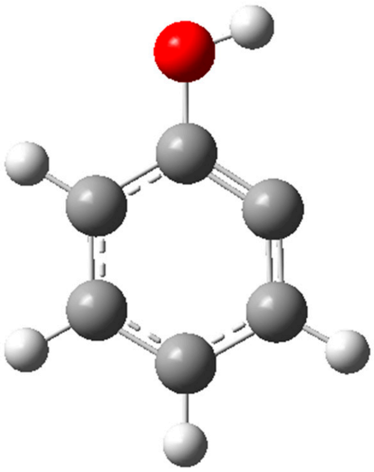

Figure S11 Visualization of the optimized structure of hydroxybenzene radical, calculated at the M06-2X/cc-pVDZ level of theory.

Table S21 Geometry (Å) of hydroxybenzene radical, calculated at the M06-2X/cc-pVDZ level of theory

Atom	x	y	z
C	-4.852	-1.637	0.390
C	-3.709	-1.894	-0.367
C	-3.005	-0.863	-0.989
C	-3.451	0.459	-0.853
C	-4.581	0.667	-0.099
C	-5.309	-0.323	0.534
H	-3.364	-2.922	-0.471
H	-2.114	-1.078	-1.577
H	-2.922	1.284	-1.328
H	-5.405	-2.439	0.878
O	-6.427	-0.092	1.277
H	-6.609	0.855	1.279

Table S22 Frequencies (cm-1) of hydroxybenzene radical, calculated at the M06-2X/ccpVDZ level of theory.

230	369	390	417	520	528	621	695
754	835	858	946	983	996	1048	1116

1156	1183	1275	1320	1359	1483	1495	1628
1681	3208	3213	3223	3235	3868		

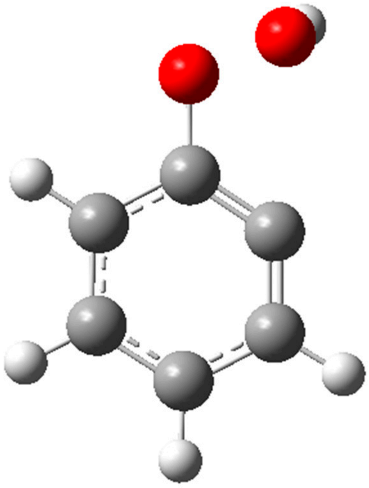

Figure S12 Visualization of the optimized structure of peroxybenzene radical, calculated at the M06-2X/cc-pVDZ level of theory.

Table S23 Geometry (Å) of peroxybenzene radical, calculated at the M06-2X/cc-pVDZ level of theory

Atom	x	y	z
C	-5.209	-1.210	0.063
C	-4.096	-1.777	-0.518
C	-3.055	-0.922	-0.901
C	-3.176	0.453	-0.690
C	-4.318	0.996	-0.106
C	-5.358	0.143	0.278
H	-4.026	-2.853	-0.676
H	-2.158	-1.332	-1.362
H	-2.368	1.119	-0.991
H	-4.422	2.069	0.056
O	-6.455	0.722	0.865
O	-7.462	-0.248	1.066
H	-7.307	-0.513	1.986

Table S24 Frequencies (cm^{-1}) of peroxybenzene radical, calculated at the M06-2X/ccpVDZ level of theory.

89	213	222	264	416	442	522	606
618	691	754	829	854	947	980	996
1041	1057	1127	1157	1250	1276	1343	1433
1465	1492	1622	1682	3209	3216	3225	3235
3794							

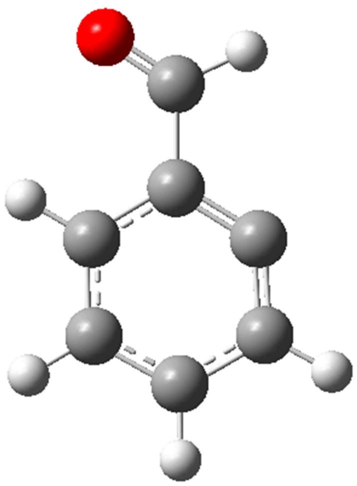

Figure S13 Visualization of the optimized structure of benzaldehyde radical, calculated at the M06-2X/cc-pVDZ level of theory.

Table S25 Geometry (Å) of benzaldehyde radical, calculated at the M06-2X/cc-pVDZ level of theory

Atom	x	y	z
C	-4.806	-1.589	0.416
C	-3.668	-1.873	-0.331
C	-2.967	-0.849	-0.979
C	-3.398	0.481	-0.886
C	-4.529	0.707	-0.134
C	-5.260	-0.262	0.525
H	-3.318	-2.900	-0.415
H	-2.077	-1.084	-1.561
H	-2.860	1.285	-1.385
H	-5.367	-2.371	0.927
O	-7.134	-0.747	1.904
C	-6.472	0.075	1.316
H	-6.741	1.153	1.335

Table S26 Frequencies (cm^{-1}) of benzaldehyde radical, calculated at the M06-2X/ccpVDZ level of theory.

117	212	226	426	434	474	617	648
703	757	836	879	977	990	1018	1036
1048	1124	1158	1216	1269	1338	1396	1459
1483	1614	1672	1828	2985	3204	3217	3222
3232							

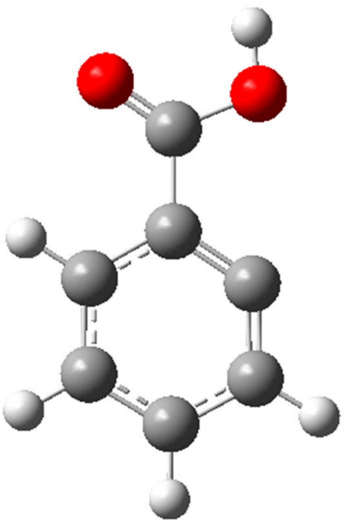

Figure S14 Visualization of the optimized structure of benzoic acid radical, calculated at the M06-2X/cc-pVDZ level of theory.

Table S27 Geometry (Å) of benzoic acid radical, calculated at the M06-2X/cc-pVDZ level of theory

Atom	x	y	z
C	-4.826	-1.565	0.425
C	-3.687	-1.856	-0.321
C	-2.983	-0.836	-0.968
C	-3.411	0.495	-0.876
C	-4.542	0.731	-0.127
C	-5.272	-0.239	0.530
H	-3.342	-2.885	-0.402
H	-2.093	-1.075	-1.550
H	-2.871	1.297	-1.376
H	-5.389	-2.344	0.937
O	-7.148	-0.762	1.914
C	-6.489	0.063	1.329
O	-6.789	1.377	1.335
H	-7.587	1.477	1.875

Table S28 Frequencies (cm^{-1}) of benzaldehyde radical, calculated at the M06-2X/ccpVDZ level of theory.

72	161	194	376	417	451	502	612
617	636	698	745	784	819	884	983
989	1019	1048	1125	1142	1166	1226	1260
1341	1396	1458	1492	1620	1676	1850	3207
3218	3224	3234	3817				

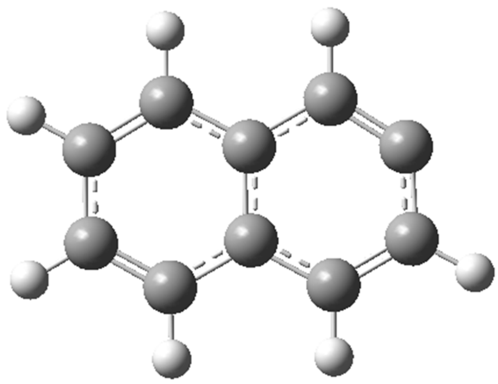

Figure S15 Visualization of the optimized structure of naphtalene radical, calculated at the M06-2X/cc-pVDZ level of theory.

Table S29 Geometry (Å) of naphtalene radical, calculated at the M06-2X/cc-pVDZ level of theory

Atom	x	y	z
C	2.380	0.677	0.000
C	1.246	1.420	0.000
C	-0.002	0.722	0.000
C	0.006	-0.705	0.000
C	1.246	-1.403	0.000
C	2.444	-0.722	0.000
H	-1.246	2.496	0.000
H	1.257	2.510	0.000
C	-1.247	1.407	0.000
C	-1.237	-1.397	0.000
H	1.234	-2.494	0.000
C	-2.425	-0.710	0.000
C	-2.430	0.709	0.000
H	-1.228	-2.487	0.000
H	3.395	-1.251	0.000
H	-3.378	1.244	0.000
H	-3.370	-1.251	0.000

Table S30 Frequencies (cm^{-1}) of naphtalene radical, calculated at the M06-2X/ccpVDZ level of theory.

179	192	376	396	480	495	510	519
621	633	753	779	785	792	826	860
921	926	981	997	1021	1048	1060	1136
1154	1161	1203	1238	1276	1344	1398	1433
1460	1475	1550	1638	1666	1699	3197	3199
3200	3202	3217	3222	3229			

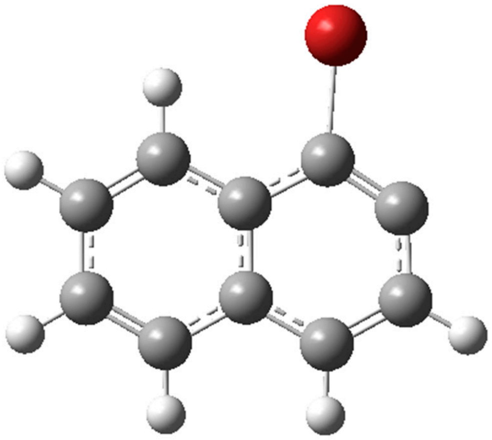

Figure S16 Visualization of the optimized structure of α-bromonaphtalene radical, calculated at the M06-2X/cc-pVDZ level of theory.

Table S31 Geometry (Å) of α-bromonaphtalene radical, calculated at the M06-2X/cc-pVDZ level of theory

Atom	x	y	z
C	-1.988	-2.130	0.000
C	-0.731	-1.576	0.000
C	-0.567	-0.166	0.000
C	-1.726	0.671	0.000
C	-3.009	0.059	0.000
C	-3.140	-1.307	0.000
H	0.152	-2.211	0.000
C	0.708	0.485	0.000
C	-1.595	2.089	0.000
H	-3.890	0.700	0.000
C	-0.355	2.687	0.000
C	0.754	1.835	0.000
H	-2.499	2.698	0.000
H	-2.100	-3.214	0.000
H	-4.129	-1.762	0.000
H	-0.242	3.770	0.000
Br	2.331	-0.503	0.000

Table S32 Frequencies (cm^{-1}) of α-bromonaphtalene radical, calculated at the M06-2X/ccpVDZ level of theory.

105	169	185	229	299	419	421	487
517	534	552	637	657	759	782	799
829	830	908	943	981	1000	1027	1058
1122	1139	1160	1172	1226	1270	1324	1393
1426	1451	1472	1547	1632	1657	1696	3203
3204	3214	3226	3228	3242			

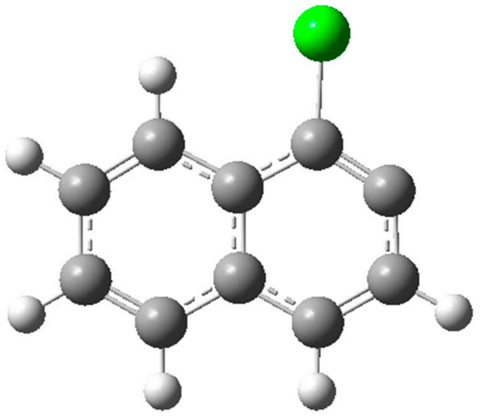

Figure S17 Visualization of the optimized structure of α-chloronaphtalene radical, calculated at the M06-2X/cc-pVDZ level of theory.

Table S33 Geometry (Å) of α-chloronaphtalene radical, calculated at the M06-2X/cc-pVDZ level of theory

Atom	x	y	z
C	-2.057	-1.730	0.000
C	-0.693	-1.565	0.000
C	-0.129	-0.262	0.000
C	-0.993	0.876	0.000
C	-2.398	0.663	0.000
C	-2.920	-0.607	0.000
H	-0.032	-2.428	0.000
C	1.280	-0.016	0.000
C	-0.452	2.193	0.000
H	-3.056	1.532	0.000
C	0.909	2.400	0.000
C	1.723	1.263	0.000
H	-1.137	3.041	0.000
H	-2.480	-2.733	0.000
H	-3.999	-0.755	0.000
H	1.335	3.402	0.000
Cl	2.421	-1.339	0.000

Table S34 Frequencies (cm^{-1}) of α-chloronaphtalene radical, calculated at the M06-2X/ccpVDZ level of theory.

115	184	214	236	389	425	439	485
520	544	561	640	674	758	784	800
830	842	908	964	980	1000	1026	1058
1129	1143	1161	1172	1226	1272	1329	1396
1429	1453	1474	1549	1634	1662	1699	3202
3206	3214	3226	3226	3243			

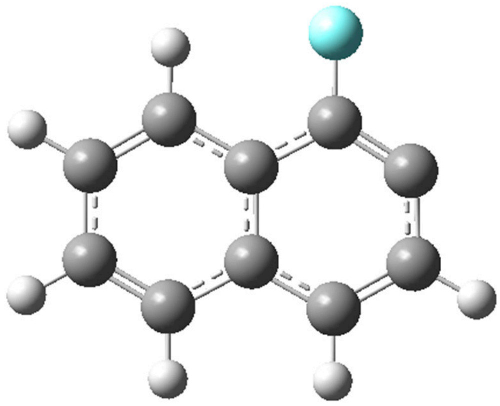

Figure S18 Visualization of the optimized structure of α-fluoronaphtalene radical, calculated at the M06-2X/cc-pVDZ level of theory.

Table S35 Geometry (Å) of α-fluoronaphtalene radical, calculated at the M06-2X/cc-pVDZ level of theory

Atom	x	y	z
C	-2.384	-1.019	0.000
C	-1.071	-1.420	0.000
C	-0.035	-0.449	0.000
C	-0.356	0.941	0.000
C	-1.728	1.313	0.000
C	-2.716	0.359	0.000
H	-0.805	-2.475	0.000
C	1.340	-0.809	0.000
C	0.684	1.913	0.000
H	-1.982	2.372	0.000
C	2.008	1.530	0.000
C	2.287	0.159	0.000
H	0.419	2.970	0.000
H	-3.179	-1.763	0.000
H	-3.763	0.660	0.000
H	2.810	2.266	0.000
F	1.658	-2.117	0.001

Table S36 Frequencies (cm^{-1}) of α-fluoronaphtalene radical, calculated at the M06-2X/ccpVDZ level of theory.

148	187	264	269	433	468	475	484
534	578	585	650	726	756	786	801
829	878	910	974	997	1026	1046	1061
1138	1156	1166	1219	1229	1279	1358	1423
1437	1458	1486	1556	1640	1683	1706	3204
3207	3216	3225	3229	3240			

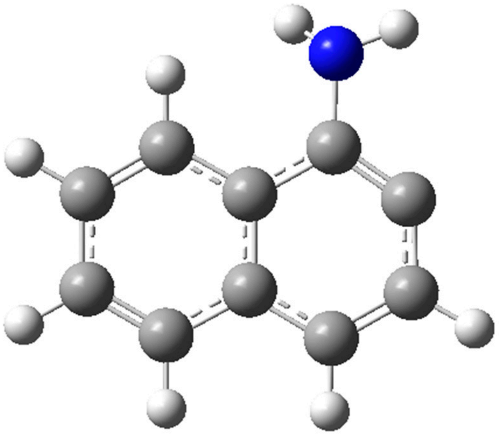

Figure S19 Visualization of the optimized structure of α-aminonaphtalene radical, calculated at the M06-2X/cc-pVDZ level of theory.

Table S37 Geometry (Å) of α-aminonaphtalene radical, calculated at the M06-2X/cc-pVDZ level of theory

Atom	x	y	z
C	-2.313	-1.141	-0.017
C	-0.976	-1.458	0.000
C	0.013	-0.438	0.030
C	-0.399	0.928	0.013
C	-1.791	1.217	0.001
C	-2.727	0.213	-0.008
H	-0.674	-2.503	-0.039
C	1.418	-0.745	0.068
C	0.567	1.973	0.001
H	-2.103	2.262	-0.004
C	1.911	1.669	0.007
C	2.270	0.322	0.045
H	0.230	3.008	-0.018
H	-3.059	-1.934	-0.047
H	-3.789	0.454	-0.018
H	2.667	2.453	-0.007
N	1.857	-2.075	0.065
H	2.846	-2.156	0.267
H	1.316	-2.688	0.662

Table S38 Frequencies (cm^{-1}) of α-aminonaphtalene radical, calculated at the M06-2X/ccpVDZ level of theory.

135	185	250	280	321	432	456	476
483	519	576	579	631	703	732	747
787	800	822	881	900	964	988	1019
1028	1058	1119	1154	1160	1185	1233	1237
1285	1369	1430	1435	1461	1482	1555	1626
1633	1677	1699	3201	3204	3209	3220	3221
3230	3570	3674					

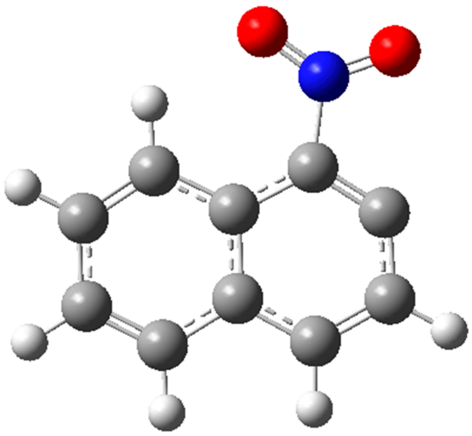

Figure S20 Visualization of the optimized structure of α-nitronaphtalene radical, calculated at the M06-2X/cc-pVDZ level of theory.

Table S39 Geometry (Å) of α-nitronaphtalene radical, calculated at the M06-2X/cc-pVDZ level of theory

Atom	x	y	z
C	-2.242	-1.685	-0.003
C	-0.869	-1.617	-0.016
C	-0.211	-0.355	-0.012
C	-1.017	0.831	0.005
C	-2.433	0.713	0.018
C	-3.039	-0.517	0.014
H	-0.282	-2.527	-0.028
C	1.209	-0.139	-0.024
C	-0.425	2.125	0.009
H	-3.028	1.626	0.031
C	0.942	2.296	-0.004
C	1.699	1.129	-0.020
H	-1.084	2.994	0.022
H	-2.724	-2.662	-0.006
H	-4.124	-0.597	0.024
H	1.402	3.281	-0.001
N	2.223	-1.207	-0.042
O	3.385	-0.850	-0.054
O	1.860	-2.367	-0.043

Table S40 Frequencies (cm^{-1}) of α-nitronaphtalene radical, calculated at the M06-2X/ccpVDZ level of theory.

6	95	187	223	227	349	361	413
472	489	529	532	597	628	666	759
780	782	814	819	837	888	910	989
1009	1016	1032	1065	1143	1151	1172	1181
1230	1280	1344	1399	1421	1459	1460	1475
1555	1631	1667	1692	1707	3202	3207	3218
3232	3234	3306					

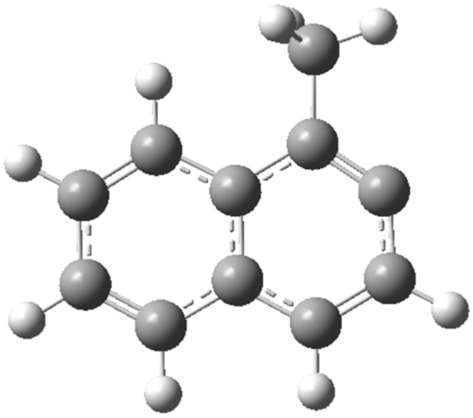

Figure S21 Visualization of the optimized structure of α-methylnaphtalene radical, calculated at the M06-2X/cc-pVDZ level of theory.

Table S41 Geometry (Å) of α-methylnaphtalene radical, calculated at the M06-2X/cc-pVDZ level of theory

Atom	x	y	z
C	-8.063	-0.998	-1.040
C	-7.028	-0.464	-0.308
C	-6.925	0.939	-0.105
C	-7.920	1.788	-0.677
C	-8.976	1.204	-1.427
C	-9.049	-0.156	-1.607
H	-6.275	-1.122	0.123
C	-5.851	1.519	0.656
C	-7.846	3.198	-0.492
H	-9.732	1.860	-1.860
C	-6.819	3.754	0.236
C	-5.873	2.873	0.773
H	-8.616	3.829	-0.937
H	-8.126	-2.075	-1.185
H	-9.864	-0.590	-2.184
H	-6.748	4.830	0.387
C	-4.776	0.669	1.280
H	-5.207	-0.047	1.993
H	-4.236	0.096	0.516
H	-4.059	1.300	1.813

Table S42 Frequencies (cm^{-1}) of α-methylnaphtalene radical, calculated at the M06-2X/ccpVDZ level of theory.

133	180	211	247	265	423	431	472
484	517	546	572	635	717	753	786
788	819	868	892	975	976	993	1019
1043	1051	1076	1136	1151	1170	1194	1227
1276	1342	1391	1417	1432	1448	1468	1473
1489	1556	1634	1673	1694	3060	3124	3167
3198	3199	3210	3221	3222	3232		

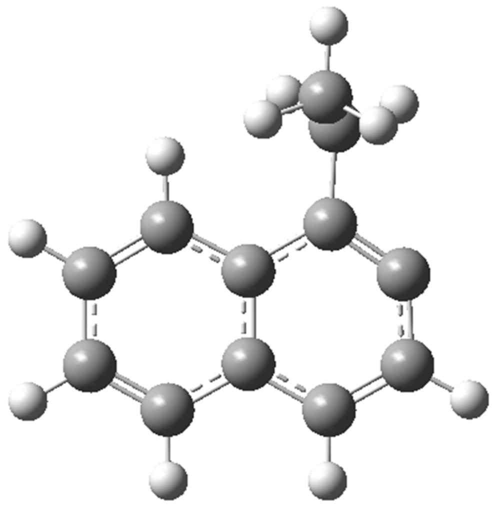

Figure S22 Visualization of the optimized structure of α-ethylnaphtalene radical, calculated at the M06-2X/cc-pVDZ level of theory.

Table S43 Geometry (Å) of α-ethylnaphtalene radical, calculated at the M06-2X/cc-pVDZ level of theory

Atom	x	y	z
C	-8.244	-1.037	-0.939
C	-7.171	-0.526	-0.247
C	-6.992	0.879	-0.107
C	-7.956	1.749	-0.700
C	-9.053	1.189	-1.409
C	-9.197	-0.172	-1.529
H	-6.449	-1.206	0.199
C	-5.883	1.442	0.617
C	-7.816	3.161	-0.575
H	-9.783	1.863	-1.857
C	-6.757	3.701	0.119
C	-5.845	2.799	0.679
H	-8.563	3.807	-1.036
H	-8.363	-2.115	-1.035
H	-10.041	-0.588	-2.075
H	-6.637	4.777	0.227
C	-4.802	0.585	1.233
H	-5.257	-0.259	1.770
H	-4.271	1.187	1.979
C	-3.806	0.073	0.187
H	-3.039	-0.555	0.656
H	-4.312	-0.516	-0.587
H	-3.310	0.918	-0.305

Table S44 Frequencies (cm^{-1}) of α-ethylnaphtalene radical, calculated at the M06-2X/ccpVDZ level of theory.

93	108	176	192	209	294	326	433
436	475	489	517	562	588	660	710
753	778	792	798	819	862	892	963
975	992	1016	1025	1056	1083	1095	1139
1152	1174	1192	1226	1265	1277	1336	1352
1385	1416	1431	1454	1470	1473	1481	1489
1557	1635	1670	1695	3066	3079	3123	3148
3149	3198	3199	3209	3220	3223	3232	

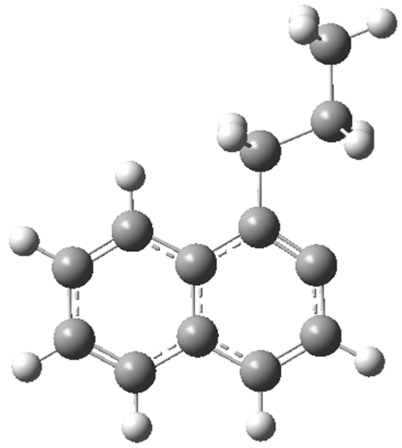

Figure S23 Visualization of the optimized structure of α-propylnaphtalene radical, calculated at the M06-2X/cc-pVDZ level of theory.

Table S45 Geometry (Å) of α-propylnaphtalene radical, calculated at the M06-2X/cc-pVDZ level of theory

Atom	x	y	z
C	-1.511	-1.779	0.000
C	-0.135	-1.765	0.000
C	0.574	-0.530	0.000
C	-0.140	0.705	0.000
C	-1.579	0.693	-0.001
C	-2.158	-0.537	-0.001
H	2.532	-1.456	0.001
H	-2.070	-2.713	-0.001
H	0.429	-2.698	0.000
C	1.995	-0.507	0.001
C	0.601	1.919	0.000
C	1.976	1.907	0.001
C	2.684	0.681	0.001
H	0.071	2.869	0.000
H	2.527	2.847	0.001
H	3.773	0.685	0.002
C	-2.382	1.971	-0.001
H	-2.099	2.571	0.878

H	-2.099	2.571	-0.880
C	-3.891	1.752	-0.001
H	-4.167	1.154	0.878
H	-4.167	1.154	-0.881
C	-4.660	3.069	-0.002
H	-4.412	3.666	-0.889
H	-5.742	2.898	-0.002
H	-4.412	3.667	0.886

Table S46 Frequencies (cm^{-1}) of α-propylnaphtalene radical, calculated at the M06-2X/ccpVDZ level of theory.

64	90	118	153	189	244	249	281
318	424	449	488	505	529	552	618
640	741	746	757	790	793	822	863
871	901	918	985	999	1019	1020	1059
1076	1108	1120	1140	1151	1171	1195	1224
1249	1267	1288	1311	1340	1389	1400	1418
1436	1452	1463	1470	1477	1484	1487	1558
1636	1672	1695	3036	3057	3066	3070	3105
3133	3142	3196	3199	3207	3216	3223	3232

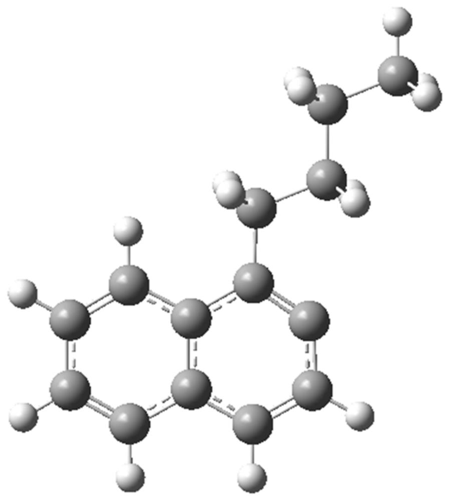

Figure S24 Visualization of the optimized structure of α-butylnaphtalene radical, calculated at the M06-2X/cc-pVDZ level of theory.

Table S47 Geometry (Å) of α-butylnaphtalene radical, calculated at the M06-2X/cc-pVDZ level of theory

Atom	x	y	z
C	-2.751	-2.571	-0.001
C	-1.375	-2.554	0.000
C	-0.668	-1.317	0.000
C	-1.386	-0.084	0.000
C	-2.825	-0.100	-0.001
C	-3.401	-1.331	-0.001

**H**	1.291	-2.239	0.002
**H**	-3.307	-3.507	-0.001
**H**	-0.809	-3.486	0.001
**C**	0.753	-1.291	0.001
**C**	-0.648	1.131	0.000
**C**	0.727	1.124	0.001
**C**	1.438	-0.101	0.002
**H**	-1.180	2.080	0.000
**H**	1.276	2.064	0.001
**H**	2.527	-0.095	0.002
**C**	-3.631	1.177	-0.002
**H**	-3.350	1.777	0.878
**H**	-3.349	1.776	-0.881
**C**	-5.139	0.954	-0.002
**H**	-5.419	0.356	0.878
**H**	-5.418	0.356	-0.883
**C**	-5.921	2.264	-0.003
**H**	-5.633	2.856	-0.884
**H**	-5.634	2.856	0.879
**C**	-7.431	2.043	-0.003
**H**	-7.741	1.475	0.884
**H**	-7.975	2.995	-0.004
**H**	-7.740	1.475	-0.891

Table S48 Frequencies (cm^{-1}) of α-butylnaphtalene radical, calculated at the M06-2X/ccpVDZ level of theory.

46	77	87	122	160	190	216	240
255	274	395	424	472	488	505	532
551	614	640	726	744	756	779	792
804	822	870	894	914	933	985	1000
1008	1020	1059	1062	1095	1115	1122	1142
1151	1170	1195	1223	1229	1249	1282	1303
1312	1319	1366	1394	1404	1418	1436	1452
1463	1466	1474	1477	1485	1488	1558	1636
1672	1695	3036	3046	3055	3058	3069	3080
3103	3131	3142	3196	3198	3207	3216	3223
3232							

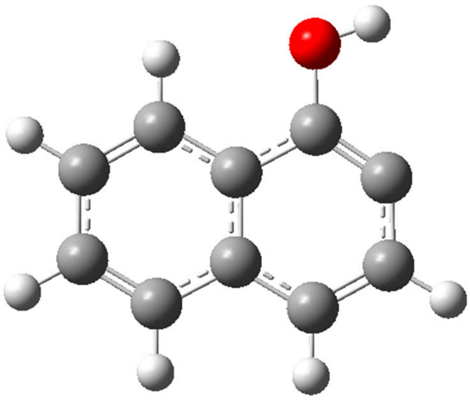

Figure S25 Visualization of the optimized structure of α-hydroxynaphtalene radical, calculated at the M06-2X/cc-pVDZ level of theory.

Table S49 Geometry (Å) of α-hydroxynaphtalene radical, calculated at the M06-2X/cc-pVDZ level of theory

Atom	x	y	z
C	-2.371	-1.068	0.000
C	-1.046	-1.436	0.000
C	-0.032	-0.443	0.000
C	-0.387	0.938	0.000
C	-1.768	1.277	0.000
C	-2.735	0.301	0.000
H	-0.755	-2.484	0.000
C	1.356	-0.792	0.000
C	0.624	1.940	0.000
H	-2.046	2.330	0.000
C	1.956	1.586	0.000
C	2.262	0.223	0.000
H	0.332	2.990	0.000
H	-3.147	-1.832	0.000
H	-3.788	0.578	0.000
H	2.742	2.340	0.000
O	1.671	-2.118	0.000
H	2.631	-2.205	0.000

Table S50 Frequencies (cm^{-1}) of α-hydroxynaphtalene radical, calculated at the M06-2X/ccpVDZ level of theory.

145	187	257	281	404	439	470	477
486	527	580	589	656	729	749	785
799	829	881	910	967	998	1027	1045
1060	1132	1156	1161	1213	1232	1255	1286
1386	1427	1439	1467	1483	1560	1638	1675
1703	3203	3207	3214	3223	3227	3242	3866

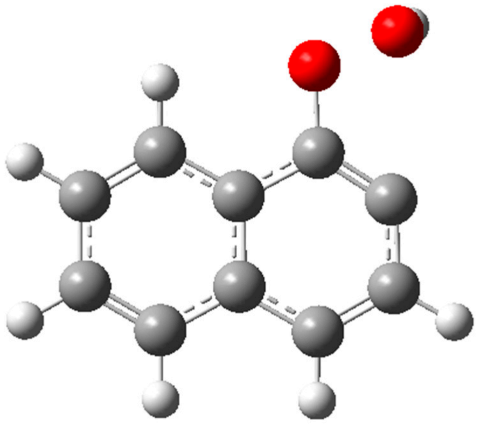

Figure S26 Visualization of the optimized structure of α-peroxynaphtalene radical, calculated at the M06-2X/cc-pVDZ level of theory.

Table S51 Geometry (Å) of α-peroxynaphtalene radical, calculated at the M06-2X/cc-pVDZ level of theory

Atom	x	y	z
C	-2.239	-1.697	-0.010
C	-0.868	-1.608	-0.013
C	-0.238	-0.335	0.000
C	-1.029	0.850	0.012
C	-2.444	0.714	0.014
C	-3.036	-0.526	0.004
H	-0.252	-2.504	-0.027
C	1.185	-0.190	-0.002
C	-0.406	2.131	0.018
H	-3.054	1.617	0.023
C	0.966	2.240	0.014
C	1.717	1.060	0.010
H	-1.032	3.023	0.024
H	-2.719	-2.675	-0.021
H	-4.121	-0.612	0.006
H	1.455	3.214	0.016
O	1.908	-1.355	0.000
O	3.285	-1.066	-0.105
H	3.552	-1.006	0.825

Table S52 Frequencies (cm^{-1}) of α-peroxynaphtalene radical, calculated at the M06-2X/ccpVDZ level of theory.

79	139	181	192	208	269	332	433
452	483	500	537	585	640	649	755
756	800	801	829	872	907	973	996
1003	1023	1055	1096	1137	1156	1171	1225
1230	1288	1354	1419	1437	1443	1458	1482
1557	1639	1677	1703	3202	3205	3213	3221
3226	3238	3785					

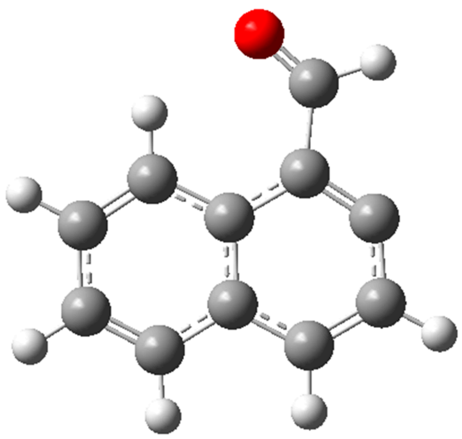

Figure S27 Visualization of the optimized structure of α-naphtaldehyde radical, calculated at the M06-2X/cc-pVDZ level of theory.

Table S53 Geometry (Å) of α-naphtaldehyde radical, calculated at the M06-2X/cc-pVDZ level of theory

Atom	x	y	z
C	-1.945	-1.746	0.000
C	-0.582	-1.556	0.000
C	-0.043	-0.241	0.000
C	-0.941	0.869	0.000
C	-2.342	0.633	0.000
C	-2.836	-0.648	0.000
H	0.097	-2.403	0.000
C	1.374	0.034	0.000
C	-0.441	2.204	0.000
H	-3.016	1.489	0.000
C	0.912	2.465	0.000
C	1.747	1.347	0.000
H	-1.157	3.027	0.000
H	-2.344	-2.760	0.000
H	-3.911	-0.822	0.000
H	1.301	3.481	0.000
C	2.456	-0.981	0.000
O	2.310	-2.182	0.000
H	3.474	-0.535	0.000

Table S54 Frequencies (cm^{-1}) of α-naphtaldehyde radical, calculated at the M06-2X/ccpVDZ level of theory.

84	154	186	211	265	356	422	428
491	503	541	554	645	649	723	760
790	792	827	885	910	989	1007	1029
1044	1052	1070	1135	1155	1172	1183	1231
1285	1356	1394	1418	1430	1457	1477	1555
1631	1658	1692	1816	2974	3197	3203	3215
3229	3229	3272					

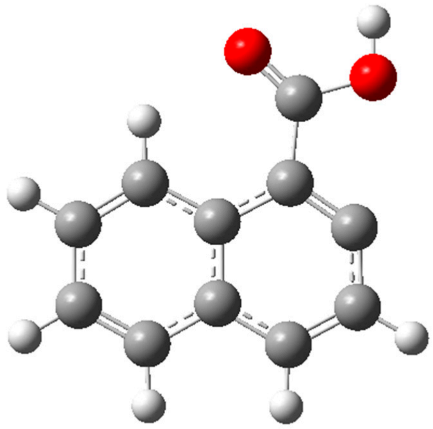

Figure S28 Visualization of the optimized structure of α-naphtalenic acid radical, calculated at the M06-2X/cc-pVDZ level of theory.

Table S55 Geometry (Å) of α-naphtalenic aicd radical, calculated at the M06-2X/cc-pVDZ level of theory

Atom	x	y	z
C	-2.109	-1.900	0.000
C	-0.770	-1.586	0.000
C	-0.349	-0.227	0.000
C	-1.348	0.795	0.000
C	-2.722	0.430	0.000
C	-3.099	-0.889	0.000
H	-0.022	-2.371	0.000
C	1.032	0.189	0.000
C	-0.980	2.170	0.000
H	-3.470	1.222	0.000
C	0.341	2.558	0.000
C	1.282	1.529	0.000
H	-1.772	2.920	0.000
H	-2.412	-2.946	0.001
H	-4.154	-1.160	0.000
H	0.633	3.606	0.000
C	2.182	-0.758	0.000
O	2.126	-1.964	0.000
O	3.364	-0.104	0.001
H	4.051	-0.786	0.001

Table S56 Frequencies (cm^{-1}) of α-naphtalenic acid radical, calculated at the M06-2X/ccpVDZ level of theory.

44	97	186	195	223	329	363	411
467	487	518	532	580	599	637	649
727	760	775	790	823	836	863	911
990	1000	1008	1041	1059	1137	1145	1164
1177	1211	1231	1282	1327	1385	1420	1428
1458	1479	1557	1634	1663	1694	1836	3199
3202	3215	3228	3230	3278	3824		

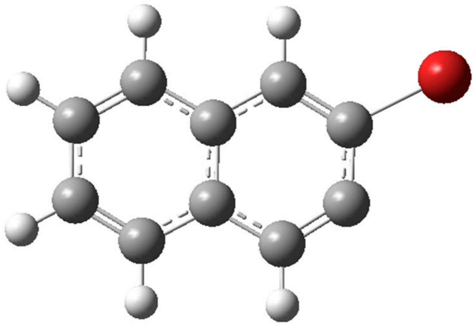

Figure S29 Visualization of the optimized structure of β-bromonaphtalene radical, calculated at the M06-2X/cc-pVDZ level of theory.

Table S57 Geometry (Å) of β-bromonaphtalene radical, calculated at the M06-2X/cc-pVDZ level of theory

Atom	x	y	z
C	3.993	0.099	0.000
C	3.063	1.109	0.000
C	1.674	0.811	0.000
C	1.254	-0.552	0.000
C	2.243	-1.574	0.000
C	3.579	-1.257	0.000
H	0.985	2.890	0.000
H	3.377	2.152	0.000
C	0.683	1.843	0.000
C	-0.134	-0.870	0.000
H	1.921	-2.615	0.000
C	-1.063	0.141	0.000
C	-0.618	1.463	0.000
H	-0.451	-1.913	0.000
H	5.056	0.336	0.000
H	4.327	-2.048	0.000
Br	-2.924	-0.221	0.000

Table S58 Frequencies (cm^{-1}) of β-bromonaphtalene radical, calculated at the M06-2X/ccpVDZ level of theory.

91	169	194	261	272	399	403	488
524	529	587	632	637	764	788	789
829	858	900	923	934	993	1023	1053
1098	1155	1158	1206	1220	1276	1328	1394
1431	1442	1470	1550	1629	1662	1694	3201
3203	3205	3214	3218	3229			

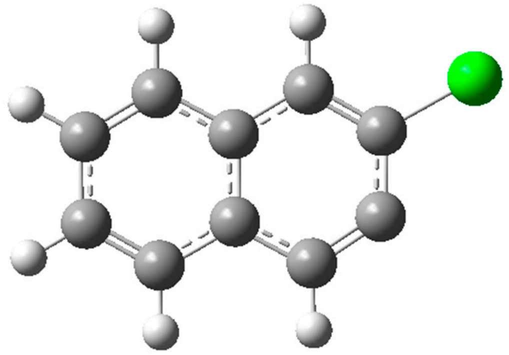

Figure S30 Visualization of the optimized structure of β-chloronaphtalene radical, calculated at the M06-2X/cc-pVDZ level of theory.

Table S59 Geometry (Å) of β-chloronaphtalene radical, calculated at the M06-2X/cc-pVDZ level of theory

Atom	x	y	z
C	-8.035	-0.994	-1.021
C	-7.006	-0.448	-0.293
C	-6.923	0.957	-0.102
C	-7.921	1.798	-0.677
C	-8.973	1.201	-1.425
C	-9.029	-0.160	-1.593
H	-5.092	0.927	1.098
H	-6.240	-1.084	0.149
C	-5.864	1.554	0.651
C	-7.854	3.209	-0.496
H	-9.735	1.845	-1.864
C	-6.826	3.755	0.232
C	-5.864	2.902	0.781
H	-8.617	3.851	-0.935
H	-8.091	-2.072	-1.162
H	-9.839	-0.606	-2.169
Cl	-6.703	5.476	0.479

Table S60 Frequencies (cm^{-1}) of β-chloronaphtalene radical, calculated at the M06-2X/ccpVDZ level of theory.

99	197	210	272	355	401	422	488
524	536	612	642	645	762	787	788
850	857	899	927	932	993	1022	1052
1112	1155	1159	1207	1219	1276	1329	1400
1431	1444	1472	1550	1638	1665	1698	3204
3204	3207	3215	3221	3232			

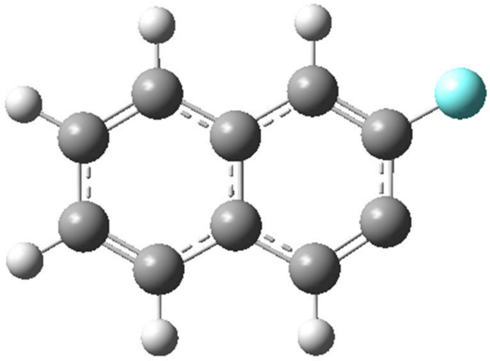

Figure S31 Visualization of the optimized structure of β-fluoronaphtalene radical, calculated at the M06-2X/cc-pVDZ level of theory.

Table S61 Geometry (Å) of β-fluoronaphtalene radical, calculated at the M06-2X/cc-pVDZ level of theory

Atom	x	y	z
C	-8.035	-0.995	-1.021
C	-7.007	-0.448	-0.293
C	-6.924	0.957	-0.103
C	-7.922	1.799	-0.677
C	-8.972	1.201	-1.425
C	-9.028	-0.161	-1.593
H	-5.093	0.923	1.097
H	-6.240	-1.083	0.149
C	-5.864	1.553	0.651
C	-7.853	3.210	-0.495
H	-9.735	1.844	-1.864
C	-6.825	3.746	0.232
C	-5.858	2.900	0.785
H	-8.609	3.864	-0.929
H	-8.091	-2.073	-1.162
H	-9.838	-0.605	-2.168
F	-6.736	5.074	0.418

Table S62 Frequencies (cm^{-1}) of β-fluoronaphtalene radical, calculated at the M06-2X/ccpVDZ level of theory.

126	197	292	309	407	421	465	488
531	557	624	649	709	762	786	789
854	896	907	928	961	991	1021	1052
1146	1156	1174	1200	1261	1274	1342	1414
1435	1462	1484	1555	1659	1673	1700	3197
3200	3202	3214	3219	3232			

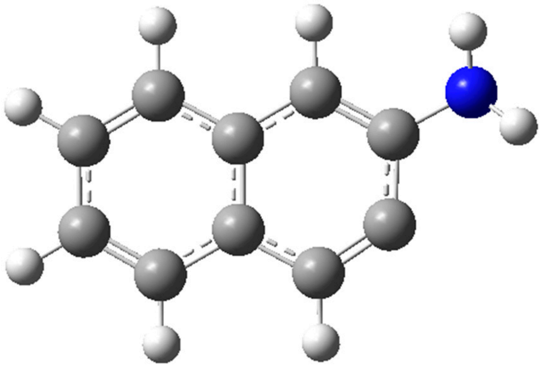

Figure S32 Visualization of the optimized structure of β-aminonaphtalene radical, calculated at the M06-2X/cc-pVDZ level of theory.

Table S63 Geometry (Å) of β-aminonaphtalene radical, calculated at the M06-2X/cc-pVDZ level of theory

Atom	x	y	z
C	-7.958	-0.971	-1.078
C	-6.948	-0.390	-0.350
C	-6.931	1.012	-0.113
C	-7.989	1.802	-0.652
C	-9.019	1.177	-1.400
C	-9.007	-0.181	-1.611
H	-6.143	-1.001	0.059
C	-5.891	1.626	0.638
C	-7.969	3.213	-0.415
H	-9.821	1.794	-1.806
C	-6.948	3.719	0.311
C	-5.877	2.989	0.869
H	-8.763	3.843	-0.817
H	-7.955	-2.047	-1.249
H	-9.801	-0.653	-2.187
H	-5.093	1.001	1.043
N	-4.917	3.624	1.654
H	-4.034	3.136	1.726
H	-4.800	4.609	1.457

Table S64 Frequencies (cm^{-1}) of β-aminonaphtalene radical, calculated at the M06-2X/ccpVDZ level of theory.

121	195	277	288	316	399	420	457
482	531	540	580	625	666	724	759
781	789	845	878	907	913	951	983
1012	1052	1104	1149	1157	1188	1214	1275
1284	1355	1416	1439	1477	1491	1554	1624
1649	1680	1697	3179	3192	3195	3198	3216
3229	3587	3699					

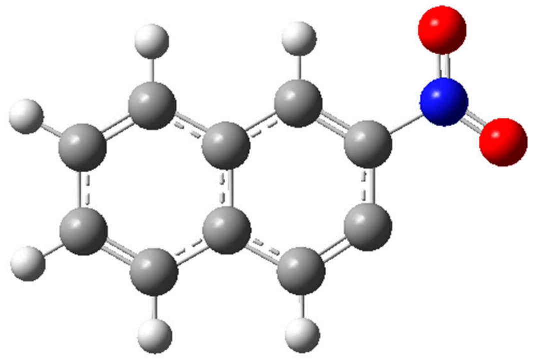

Figure S33 Visualization of the optimized structure of β-nitronaphtalene radical, calculated at the M06-2X/cc-pVDZ level of theory.

Table S65 Geometry (Å) of β-nitronaphtalene radical, calculated at the M06-2X/cc-pVDZ level of theory

Atom	x	y	z
C	-8.035	-0.986	-1.025
C	-7.033	-0.448	-0.254
C	-6.940	0.956	-0.071
C	-7.903	1.802	-0.701
C	-8.928	1.214	-1.492
C	-8.993	-0.148	-1.651
H	-5.167	0.905	1.212
H	-6.298	-1.092	0.227
C	-5.906	1.542	0.728
C	-7.827	3.210	-0.530
H	-9.659	1.865	-1.970
C	-6.822	3.735	0.243
C	-5.889	2.890	0.851
H	-8.550	3.875	-1.000
N	-6.715	5.186	0.440
O	-5.800	5.578	1.136
O	-7.541	5.894	-0.102
H	-8.098	-2.064	-1.160
H	-9.780	-0.590	-2.259

Table S66 Frequencies (cm^{-1}) of β-nitronaphtalene radical, calculated at the M06-2X/ccpVDZ level of theory.

62	94	174	202	254	330	373	401
491	513	521	557	608	621	637	760
784	789	803	815	861	907	914	954
954	998	1023	1052	1129	1158	1164	1208
1233	1277	1338	1404	1442	1458	1469	1478
1563	1645	1672	1698	1711	3208	3209	3212
3224	3229	3234					

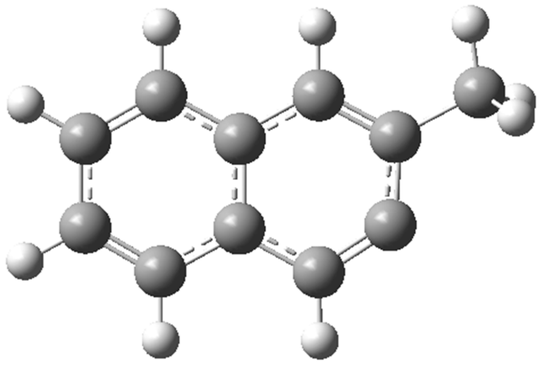

Figure S34 Visualization of the optimized structure of β-methylnaphtalene radical, calculated at the M06-2X/cc-pVDZ level of theory.

Table S67 Geometry (Å) of β-methylnaphtalene radical, calculated at the M06-2X/cc-pVDZ level of theory

Atom	x	y	z
C	-8.038	-1.006	-1.022
C	-6.988	-0.454	-0.327
C	-6.910	0.950	-0.131
C	-7.934	1.786	-0.662
C	-9.005	1.185	-1.376
C	-9.058	-0.177	-1.552
H	-5.038	0.941	1.003
H	-6.201	-1.087	0.083
C	-5.833	1.559	0.587
C	-7.865	3.197	-0.471
H	-9.789	1.824	-1.783
C	-6.829	3.790	0.220
C	-5.853	2.906	0.718
H	-8.661	3.818	-0.887
H	-8.089	-2.084	-1.168
H	-9.884	-0.625	-2.101
C	-6.730	5.275	0.438
H	-5.803	5.668	0.004
H	-7.579	5.793	-0.021
H	-6.716	5.508	1.510

Table S68 Frequencies (cm^{-1}) of β-methylnaphtalene radical, calculated at the M06-2X/cc-pVDZ level of theory.

109	128	198	258	280	402	415	446
490	529	531	625	637	706	760	790
791	855	890	892	926	945	988	1015
1017	1051	1052	1150	1155	1180	1214	1242
1278	1337	1394	1412	1425	1451	1460	1476
1486	1549	1646	1673	1700	3065	3134	3159
3179	3194	3197	3200	3217	3229		

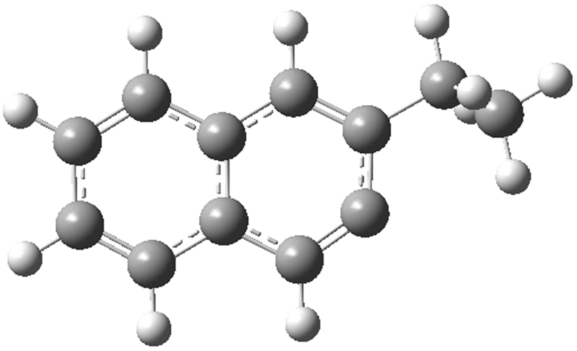

Figure S35 Visualization of the optimized structure of β-ethylnaphtalene radical, calculated at the M06-2X/cc-pVDZ level of theory.

Table S69 Geometry (Å) of β-ethylnaphtalene radical, calculated at the M06-2X/cc-pVDZ level of theory

Atom	x	y	z
C	-8.038	-1.051	-0.963
C	-6.945	-0.434	-0.403
C	-6.914	0.977	-0.248
C	-8.029	1.752	-0.679
C	-9.143	1.083	-1.255
C	-9.149	-0.284	-1.395
H	-4.926	1.084	0.663
H	-6.088	-1.019	-0.070
C	-5.792	1.654	0.328
C	-8.007	3.169	-0.528
H	-9.996	1.675	-1.586
C	-6.931	3.827	0.030
C	-5.863	3.002	0.431
H	-8.873	3.742	-0.867
H	-8.053	-2.134	-1.079
H	-10.009	-0.784	-1.837
C	-6.875	5.323	0.207
H	-7.763	5.768	-0.258
H	-6.924	5.554	1.281
C	-5.603	5.931	-0.385
H	-5.537	5.717	-1.459
H	-5.588	7.018	-0.245
H	-4.714	5.509	0.100

Table S70 Frequencies (cm^{-1}) of β-ethylnaphtalene radical, calculated at the M06-2X/cc-pVDZ level of theory.

40	98	186	196	213	290	336	401
408	481	489	528	558	623	655	707
759	781	790	792	853	878	893	924
937	988	995	1015	1052	1080	1094	1149
1154	1180	1211	1239	1272	1278	1320	1356

1388	1410	1434	1453	1467	1472	1477	1487
1548	1645	1673	1698	3063	3065	3113	3144
3149	3178	3193	3195	3198	3215	3228	

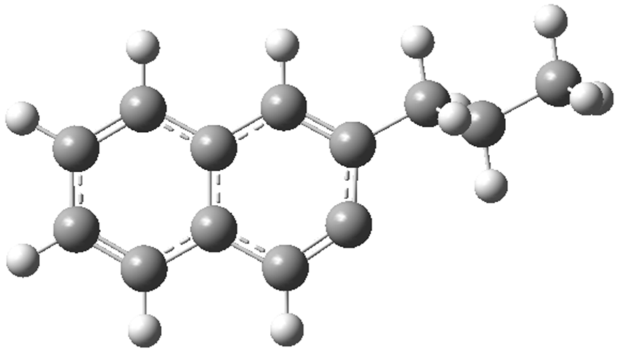

Figure S36 Visualization of the optimized structure of β-propylnaphtalene radical, calculated at the M06-2X/cc-pVDZ level of theory.

Table S71 Geometry (Å) of β-propylnaphtalene radical, calculated at the M06-2X/cc-pVDZ level of theory

Atom	x	y	z
C	-3.083	-1.546	0.170
C	-1.736	-1.679	0.162
C	-0.956	-0.483	0.059
C	-1.633	0.767	-0.025
C	-3.058	0.803	-0.008
C	-3.815	-0.346	0.087
H	0.974	-1.468	0.105
H	-1.240	-2.647	0.229
C	0.464	-0.507	0.041
C	-0.862	1.957	-0.124
H	-3.556	1.772	-0.075
C	0.511	1.906	-0.140
C	1.182	0.661	-0.056
H	-1.380	2.914	-0.188
H	1.090	2.825	-0.216
H	2.270	0.632	-0.070
C	-5.321	-0.346	0.119
H	-5.701	-0.892	-0.757
H	-5.683	0.688	0.037
C	-5.880	-0.994	1.388
H	-5.494	-2.020	1.460
H	-5.496	-0.451	2.263
C	-7.405	-1.005	1.402
H	-7.797	-1.566	0.543
H	-7.792	-1.472	2.316
H	-7.805	0.016	1.348

Table S72 Frequencies (cm⁻¹) of β-propylnaphtalene radical, calculated at the M06-2X/cc-pVDZ level of theory.

33	73	85	165	195	244	265	293
323	403	421	489	504	528	560	626
658	734	742	763	790	790	855	862
879	901	921	928	953	990	1016	1052
1073	1095	1120	1150	1156	1181	1214	1231
1254	1277	1287	1309	1340	1376	1395	1411
1434	1455	1462	1468	1476	1481	1485	1548
1645	1672	1698	3052	3058	3066	3093	3112
3134	3143	3182	3197	3198	3202	3215	3227

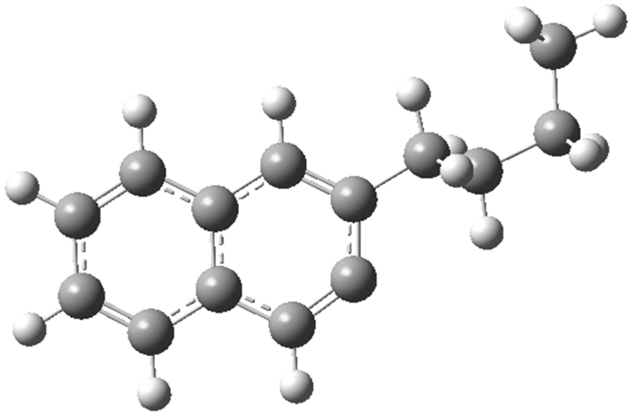

Figure S37 Visualization of the optimized structure of β-butylnaphtalene radical, calculated at the M06-2X/cc-pVDZ level of theory.

Table S73 Geometry (Å) of β-butylnaphtalene radical, calculated at the M06-2X/cc-pVDZ level of theory

Atom	x	y	z
C	-3.565	-2.001	0.172
C	-2.223	-2.176	0.160
C	-1.406	-1.006	0.046
C	-2.045	0.264	-0.045
C	-3.468	0.344	-0.024
C	-4.260	-0.780	0.084
H	0.492	-2.051	0.094
H	-1.757	-3.159	0.232
C	0.012	-1.075	0.024
C	-1.237	1.428	-0.156
H	-3.936	1.328	-0.096
C	0.134	1.335	-0.175
C	0.765	0.070	-0.084
H	-1.725	2.400	-0.225
H	0.741	2.235	-0.260
H	1.852	0.007	-0.100
C	-5.765	-0.730	0.120
H	-6.167	-1.282	-0.744

**H**	-6.086	0.314	0.018
**C**	-6.333	-1.343	1.405
**H**	-5.910	-2.350	1.527
**H**	-5.989	-0.749	2.264
**C**	-7.859	-1.428	1.411
**H**	-8.185	-2.071	0.580
**H**	-8.179	-1.926	2.336
**C**	-8.547	-0.068	1.307
**H**	-8.358	0.407	0.337
**H**	-9.634	-0.167	1.419
**H**	-8.187	0.610	2.093

Table S74 Frequencies (cm^{-1}) of β-butylnaphtalene radical, calculated at the M06-2X/cc-pVDZ level of theory.

24	42	68	126	159	194	220	262
278	326	395	402	434	488	508	528
560	626	657	724	750	763	786	790
790	855	886	897	903	925	941	948
990	993	1014	1052	1096	1111	1122	1149
1154	1181	1208	1214	1250	1260	1277	1299
1311	1341	1358	1384	1397	1411	1433	1454
1459	1467	1474	1481	1482	1486	1548	1644
1672	1697	3047	3056	3057	3060	3094	3100
3124	3134	3142	3178	3194	3196	3200	3215
3228							

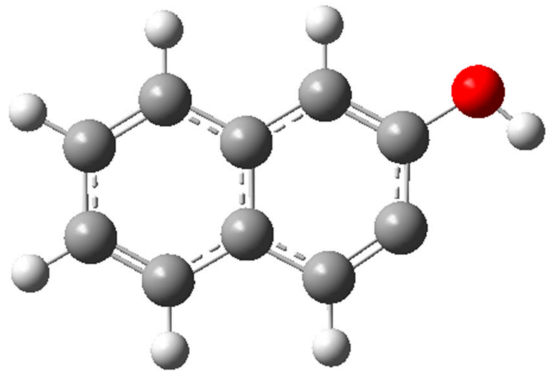

Figure S38 Visualization of the optimized structure of β-hydroxynaphtalene radical, calculated at the M06-2X/cc-pVDZ level of theory.

Table S75 Geometry (Å) of β-hydroxynaphtalene radical, calculated at the M06-2X/cc-pVDZ level of theory

Atom	x	y	z
**C**	-8.054	-1.005	-0.970
**C**	-7.009	-0.437	-0.282
**C**	-6.916	0.973	-0.144

C	-7.918	1.804	-0.727
C	-8.986	1.183	-1.432
C	-9.053	-0.184	-1.550
H	-5.062	0.981	1.020
H	-6.237	-1.061	0.168
C	-5.840	1.594	0.566
C	-7.840	3.218	-0.599
H	-9.755	1.813	-1.879
C	-6.800	3.801	0.087
C	-5.835	2.944	0.644
H	-8.606	3.853	-1.044
H	-8.118	-2.087	-1.071
H	-9.877	-0.643	-2.094
O	-6.732	5.156	0.204
H	-5.945	5.386	0.711

Table S76 Frequencies (cm^{-1}) of β-hydroxynaphtalene radical, calculated at the M06-2X/cc-pVDZ level of theory.

125	197	290	303	383	404	424	461
486	534	561	625	656	719	760	785
788	851	893	908	926	958	989	1018
1051	1144	1156	1178	1190	1228	1273	1288
1359	1415	1443	1472	1489	1559	1659	1675
1698	3196	3198	3200	3207	3217	3230	3866

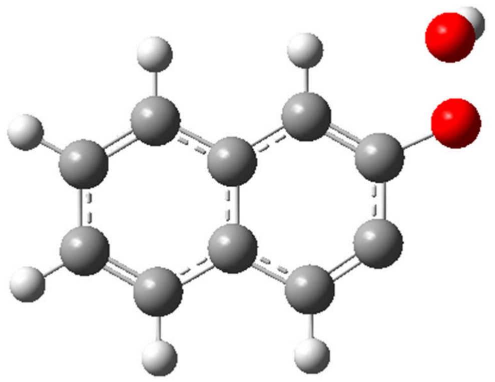

Figure S39 Visualization of the optimized structure of β-peroxynaphtalene radical, calculated at the M06-2X/cc-pVDZ level of theory.

Table S77 Geometry (Å) of β-peroxynaphtalene radical, calculated at the M06-2X/cc-pVDZ level of theory

Atom	x	y	z
C	-3.330	0.468	0.012
C	-2.238	1.303	0.008
C	-0.923	0.771	-0.003
C	-0.737	-0.641	-0.010

C	-1.886	-1.476	-0.007
C	-3.150	-0.936	0.004
H	0.117	2.699	-0.006
H	-2.367	2.385	0.013
C	0.234	1.616	-0.010
C	0.577	-1.199	-0.021
H	-1.748	-2.557	-0.014
C	1.667	-0.365	-0.024
C	1.448	1.025	-0.020
H	0.709	-2.277	-0.037
H	-4.336	0.884	0.021
H	-4.021	-1.591	0.006
O	2.986	-0.748	-0.037
O	3.090	-2.156	-0.031
H	3.400	-2.322	0.871

Table S78 Frequencies (cm^{-1}) of β-peroxynaphtalene radical, calculated at the M06-2X/cc-pVDZ level of theory.

83	132	193	200	206	307	350	404
439	490	524	556	559	630	653	711
763	788	791	855	892	916	924	964
989	1016	1050	1055	1150	1157	1182	1205
1250	1277	1341	1409	1431	1448	1462	1486
1561	1652	1676	1696	3199	3200	3203	3216
3229	3244	3810					

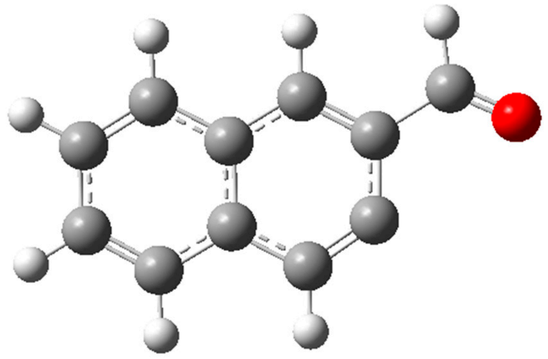

Figure S40 Visualization of the optimized structure of β-naphtaldehyde radical, calculated at the M06-2X/cc-pVDZ level of theory.

Table S79 Geometry (Å) of β-naphtaldehyde radical, calculated at the M06-2X/cc-pVDZ level of theory

Atom	x	y	z
C	-2.000	0.679	0.000
C	-1.863	-0.731	0.000
C	-0.889	1.489	0.000
C	-0.615	-1.303	0.000

C	0.553	-0.494	0.000
C	0.413	0.926	0.000
C	1.590	1.745	0.000
H	1.492	2.831	0.000
C	2.790	1.123	0.000
C	2.984	-0.271	0.000
C	1.855	-1.065	0.000
H	-0.994	2.573	0.000
H	-0.502	-2.387	0.000
H	1.958	-2.153	0.000
O	5.365	-0.260	0.000
C	4.333	-0.886	0.000
H	4.336	-1.999	0.000
H	-2.995	1.122	0.000
H	-2.753	-1.359	0.000

Table S80 Frequencies (cm^{-1}) of β-naphtaldehyde radical, calculated at the M06-2X/cc-pVDZ level of theory.

80	162	168	205	294	349	396	402
492	522	533	610	627	651	763	774
793	796	860	888	904	943	948	994
1022	1034	1052	1149	1155	1179	1207	1258
1278	1326	1396	1410	1444	1456	1479	1551
1642	1671	1697	1834	2961	3178	3197	3200
3203	3220	3232					

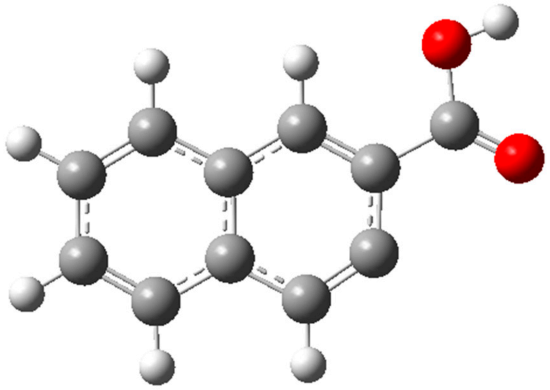

Figure S41 Visualization of the optimized structure of β-naphtalenic acid radical, calculated at the M06-2X/cc-pVDZ level of theory.

Table S81 Geometry (Å) of β-naphtalenic aicd radical, calculated at the M06-2X/cc-pVDZ level of theory

Atom	x	y	z
C	-2.127	-0.740	0.000
C	-2.365	0.658	0.000
C	-0.841	-1.220	0.000
C	-1.316	1.545	0.000

**C**	0.024	1.077	0.000
**C**	0.267	-0.329	0.000
**C**	1.603	-0.814	0.000
**H**	1.784	-1.888	0.000
**C**	2.670	0.062	0.000
**C**	2.380	1.437	0.000
**C**	1.138	1.976	0.000
**H**	-0.650	-2.293	0.000
**H**	-1.498	2.619	0.000
**H**	0.968	3.052	0.000
**C**	4.087	-0.388	0.000
**O**	5.039	0.351	0.000
**O**	4.210	-1.734	0.000
**H**	5.160	-1.922	0.000
**H**	-2.969	-1.430	0.000
**H**	-3.390	1.028	0.000

Table S82 Frequencies (cm^{-1}) of β-naphtalenic acid radical, calculated at the M06-2X/cc-pVDZ level of theory.

61	89	151	202	246	325	354	401
492	507	511	524	592	595	629	652
717	759	789	794	805	860	884	910
940	959	997	1021	1050	1120	1154	1156
1197	1213	1249	1277	1331	1378	1416	1437
1462	1483	1551	1641	1674	1697	1854	3203
3204	3207	3219	3220	3231	3823		

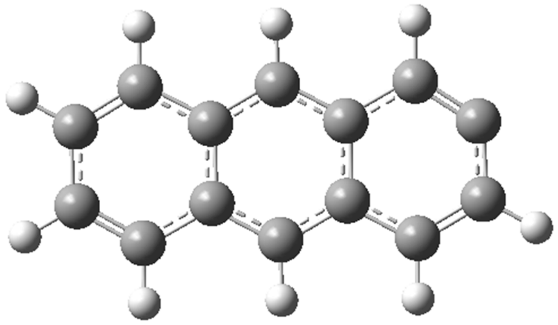

Figure S42 Visualization of the optimized structure of antracene radical, calculated at the M06-2X/cc-pVDZ level of theory.

Table S83 Geometry (Å) of antracene radical, calculated at the M06-2X/cc-pVDZ level of theory

Atom	x	y	z
**C**	-5.897	5.957	-2.865
**C**	-4.549	5.950	-2.645
**C**	-3.892	4.775	-2.155
**C**	-4.676	3.600	-1.898

**C**	-6.087	3.648	-2.142
**C**	-6.678	4.787	-2.609
**C**	-2.513	4.741	-1.921
**C**	-4.047	2.446	-1.420
**C**	-2.669	2.413	-1.186
**C**	-1.881	3.589	-1.443
**C**	-0.466	3.556	-1.204
**H**	0.117	4.456	-1.403
**C**	0.146	2.424	-0.735
**C**	-0.673	1.298	-0.500
**C**	-2.006	1.232	-0.694
**H**	-1.918	5.634	-2.116
**H**	-6.386	6.856	-3.237
**H**	-3.950	6.840	-2.838
**H**	-6.681	2.755	-1.946
**H**	-7.751	4.811	-2.790
**H**	-4.643	1.553	-1.225
**H**	-2.586	0.331	-0.493
**H**	1.218	2.395	-0.552

Table S84 Frequencies (cm^{-1}) of antracene radical, calculated at the M06-2X/cc-pVDZ level of theory.

92	124	242	244	279	390	398	401
477	490	518	528	595	604	631	653
750	758	775	792	805	833	854	881
886	913	923	940	991	998	1018	1035
1047	1127	1146	1162	1177	1194	1256	1283
1290	1316	1364	1371	1443	1451	1478	1484
1519	1607	1636	1651	1686	1713	3194	3196
3200	3200	3202	3203	3218	3224	3229	

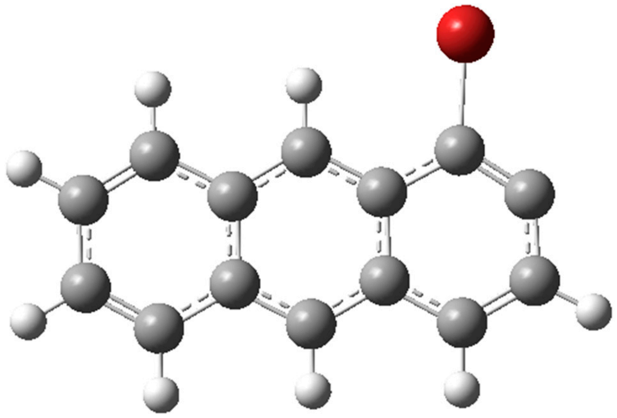

Figure S43 Visualization of the optimized structure of α-bromoantracene radical, calculated at the M06-2X/cc-pVDZ level of theory.

Table S85 Geometry (Å) of α-bromoantracene radical, calculated at the M06-2X/cc-pVDZ level of theory

Atom	x	y	z
C	4.020	-1.655	0.000
C	2.663	-1.801	0.000
C	1.803	-0.655	0.000
C	2.396	0.651	0.000
C	3.824	0.764	0.000
C	4.610	-0.352	0.000
C	0.409	-0.783	0.000
C	1.564	1.775	0.000
C	0.171	1.658	0.000
C	-0.418	0.341	0.000
C	-1.860	0.274	0.000
C	-2.566	1.417	0.000
C	-2.028	2.720	0.000
C	-0.664	2.825	0.000
H	-0.035	-1.777	0.000
H	4.665	-2.532	0.000
H	2.209	-2.792	0.000
H	4.269	1.758	0.000
H	5.695	-0.258	0.000
H	2.011	2.770	0.000
H	-0.186	3.804	0.000
H	-2.671	3.598	0.000
Br	-2.771	-1.393	0.000

Table S86 Frequencies (cm^{-1}) of α-bromoantracene radical, calculated at the M06-2X/cc-pVDZ level of theory.

76	94	123	178	253	263	287	315
409	410	439	490	512	537	558	594
620	648	670	753	772	792	794	815
852	878	904	920	926	942	991	1002
1022	1038	1108	1137	1148	1164	1183	1216
1283	1290	1310	1364	1368	1432	1446	1477
1483	1518	1602	1635	1647	1684	1712	3195
3199	3204	3205	3218	3224	3228	3229	

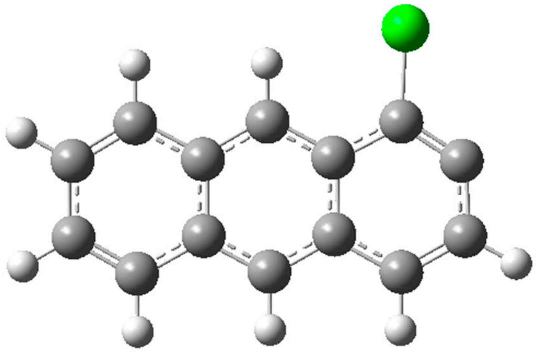

Figure S44 Visualization of the optimized structure of α-chloroantracene radical, calculated at the M06-2X/cc-pVDZ level of theory.

Table S87 Geometry (Å) of α-chloroantracene radical, calculated at the M06-2X/cc-pVDZ level of theory

Atom	x	y	z
C	3.854	-1.194	0.000
C	2.549	-1.594	0.000
C	1.488	-0.632	0.000
C	1.822	0.763	0.000
C	3.203	1.144	0.000
C	4.187	0.197	0.000
C	0.143	-1.022	0.000
C	0.793	1.710	0.000
C	-0.553	1.332	0.000
C	-0.880	-0.072	0.000
C	-2.281	-0.417	0.000
C	-3.196	0.569	0.000
C	-2.915	1.950	0.000
C	-1.595	2.317	0.000
H	-0.106	-2.081	0.000
H	4.653	-1.933	0.000
H	2.291	-2.653	0.000
H	3.452	2.205	0.000
H	5.234	0.495	0.000
H	1.045	2.771	0.000
H	-1.316	3.370	0.000
H	-3.714	2.689	0.000
Cl	-2.782	-2.089	0.000

Table S88 Frequencies (cm^{-1}) of α-chloroantracene radical, calculated at the M06-2X/cc-pVDZ level of theory.

81	98	158	185	256	288	290	371
410	429	452	488	512	539	564	596
622	648	688	750	770	790	798	813
854	874	912	921	936	938	988	1000
1021	1037	1115	1139	1150	1166	1184	1217
1286	1291	1314	1363	1369	1432	1449	1478

1485	1518	1604	1636	1648	1684	1713	3197
3202	3206	3207	3220	3226	3228	3231	

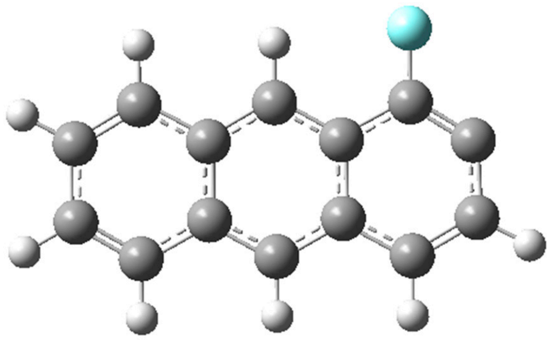

Figure S45 Visualization of the optimized structure of α-fluoroantracene radical, calculated at the M06-2X/cc-pVDZ level of theory.

Table S89 Geometry (Å) of α-fluoroantracene radical, calculated at the M06-2X/cc-pVDZ level of theory

Atom	x	y	z
C	3.771	0.898	0.000
C	2.530	1.467	0.000
C	1.352	0.653	0.000
C	1.498	-0.775	0.000
C	2.818	-1.333	0.000
C	3.918	-0.524	0.000
C	0.070	1.215	0.000
C	0.356	-1.584	0.000
C	-0.930	-1.034	0.000
C	-1.063	0.398	0.000
C	-2.391	0.937	0.000
C	-3.449	0.104	0.000
C	-3.355	-1.302	0.000
C	-2.103	-1.859	0.000
H	-0.049	2.297	0.000
H	4.661	1.526	0.000
H	2.415	2.551	0.000
H	2.925	-2.418	0.000
H	4.916	-0.958	0.000
H	0.471	-2.668	0.000
H	-1.983	-2.942	0.000
H	-4.250	-1.921	0.000
F	-2.527	2.275	0.000

Table S90 Frequencies (cm^{-1}) of α-fluoroantracene radical, calculated at the M06-2X/cc-pVDZ level of theory.

94	100	192	210	261	296	321	391
416	486	490	509	517	549	580	614

624	648	741	751	771	790	809	814
859	874	912	921	938	983	999	1020
1034	1037	1136	1143	1161	1182	1206	1224
1287	1290	1347	1365	1393	1437	1457	1478
1491	1524	1608	1635	1663	1700	1711	3198
3200	3205	3207	3217	3222	3225	3229	

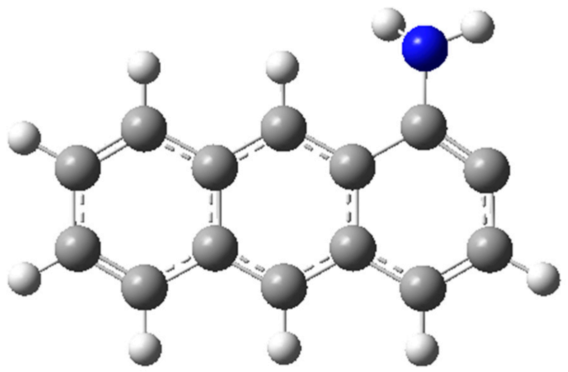

Figure S46 Visualization of the optimized structure of α-aminoantracene radical, calculated at the M06-2X/cc-pVDZ level of theory.

Table S91 Geometry (Å) of α-aminoantracene radical, calculated at the M06-2X/cc-pVDZ level of theory

Atom	x	y	z
C	3.773	0.924	-0.030
C	2.524	1.478	-0.048
C	1.356	0.651	-0.018
C	1.519	-0.773	0.021
C	2.844	-1.316	0.039
C	3.935	-0.495	0.015
C	0.064	1.193	-0.032
C	0.382	-1.589	0.027
C	-0.910	-1.054	0.008
C	-1.069	0.377	-0.005
C	-2.410	0.930	0.005
C	-3.431	0.037	-0.024
C	-3.320	-1.365	-0.038
C	-2.062	-1.907	-0.011
H	-0.041	2.276	-0.094
H	4.655	1.563	-0.051
H	2.397	2.560	-0.083
H	2.964	-2.399	0.070
H	4.939	-0.918	0.029
H	0.506	-2.672	0.041
H	-1.920	-2.987	-0.010
H	-4.208	-1.995	-0.060
N	-2.591	2.319	-0.019

H	-3.554	2.584	0.147
H	-1.968	2.822	0.602

Table S92 Frequencies (cm^{-1}) of α-aminoantracene radical, calculated at the M06-2X/cc-pVDZ level of theory.

92	98	194	204	259	287	320	336
392	414	462	486	508	520	552	574
601	625	647	703	743	751	774	784
812	817	860	869	909	915	933	971
993	1014	1016	1038	1111	1145	1155	1168
1184	1229	1242	1290	1293	1352	1367	1405
1439	1454	1479	1488	1522	1604	1623	1636
1662	1693	1710	3194	3198	3202	3204	3209
3216	3221	3227	3565	3670			

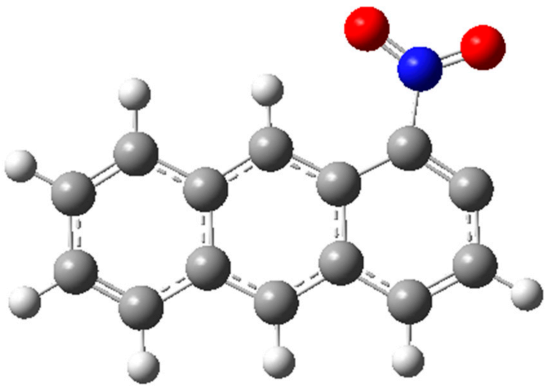

Figure S47 Visualization of the optimized structure of α-nitroantracene radical, calculated at the M06-2X/cc-pVDZ level of theory.

Table S93 Geometry (Å) of α-nitroantracene radical, calculated at the M06-2X/cc-pVDZ level of theory

Atom	x	y	z
C	-3.962	-1.360	-0.033
C	-2.632	-1.672	-0.031
C	-1.640	-0.639	-0.017
C	-2.072	0.726	-0.005
C	-3.474	1.016	-0.008
C	-4.390	0.003	-0.021
C	-0.272	-0.950	-0.015
C	-1.104	1.733	0.009
C	0.262	1.437	0.011
C	0.698	0.057	-0.001
C	2.133	-0.136	0.003
C	2.955	0.938	0.017
C	2.562	2.283	0.029
C	1.211	2.512	0.025

**H**	0.026	-1.992	-0.024
**H**	-4.708	-2.153	-0.044
**H**	-2.302	-2.710	-0.040
**H**	-3.794	2.058	0.001
**H**	-5.455	0.229	-0.023
**H**	-1.418	2.777	0.018
**H**	0.828	3.533	0.034
**H**	3.289	3.092	0.040
**N**	2.802	-1.445	-0.008
**O**	4.017	-1.437	0.000
**O**	2.122	-2.454	-0.023

Table S94 Frequencies (cm^{-1}) of α-nitroantracene radical, calculated at the M06-2X/cc-pVDZ level of theory.

6	67	99	158	174	252	277	287
335	371	402	420	487	514	532	534
559	592	624	655	681	752	762	790
797	798	829	844	866	876	912	927
948	998	999	1006	1019	1036	1136	1146
1153	1168	1186	1222	1288	1294	1319	1366
1368	1433	1447	1460	1477	1483	1516	1600
1630	1649	1678	1702	1710	3198	3202	3203
3209	3220	3230	3235	3287			

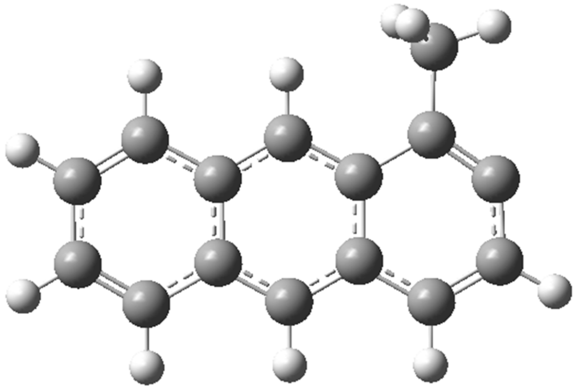

Figure S48 Visualization of the optimized structure of α-methylantracene radical, calculated at the M06-2X/cc-pVDZ level of theory.

Table S95 Geometry (Å) of α-methylantracene radical, calculated at the M06-2X/cc-pVDZ level of theory

Atom	x	y	z
**C**	3.767	0.943	0.000
**C**	2.514	1.486	0.000
**C**	1.352	0.648	0.000
**C**	1.530	-0.775	0.000
**C**	2.859	-1.306	0.000

**C**	3.943	-0.476	0.000
**C**	0.056	1.180	0.000
**C**	0.400	-1.601	0.000
**C**	-0.894	-1.073	0.000
**C**	-1.073	0.357	0.000
**C**	-2.417	0.900	0.000
**C**	-3.420	-0.005	0.000
**C**	-3.306	-1.412	0.000
**C**	-2.042	-1.935	0.000
**H**	-0.062	2.263	0.000
**H**	4.643	1.590	0.000
**H**	2.377	2.567	0.000
**H**	2.988	-2.389	0.000
**H**	4.951	-0.889	0.000
**H**	0.532	-2.683	0.000
**H**	-1.886	-3.014	0.000
**H**	-4.186	-2.052	0.000
**C**	-2.658	2.386	0.000
**H**	-2.211	2.854	0.887
**H**	-2.211	2.854	-0.887
**H**	-3.732	2.593	0.000

Table S96 Frequencies (cm^{-1}) of α-methylantracene radical, calculated at the M06-2X/cc-pVDZ level of theory.

92	96	194	195	222	256	292	316
392	410	439	490	512	513	548	554
594	624	650	733	753	769	790	811
813	856	871	912	921	938	963	987
995	1015	1039	1046	1057	1136	1145	1160
1181	1188	1223	1288	1290	1334	1362	1379
1396	1431	1452	1466	1475	1477	1491	1521
1610	1636	1662	1688	1710	3057	3122	3164
3192	3197	3198	3202	3212	3215	3220	3227

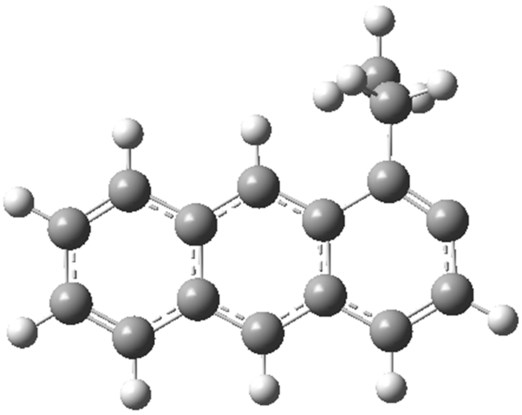

Figure S49 Visualization of the optimized structure of α-ethylantracene radical, calculated at the M06-2X/cc-pVDZ level of theory.

Table S97 Geometry (Å) of α-ethylantracene radical, calculated at the M06-2X/cc-pVDZ level of theory

Atom	x	y	z
C	-5.921	6.019	-2.806
C	-4.569	6.001	-2.608
C	-3.912	4.817	-2.143
C	-4.702	3.647	-1.888
C	-6.117	3.705	-2.107
C	-6.707	4.853	-2.552
C	-2.528	4.772	-1.934
C	-4.068	2.487	-1.431
C	-2.687	2.440	-1.219
C	-1.891	3.614	-1.479
C	-0.458	3.557	-1.250
C	0.020	2.376	-0.798
C	-0.708	1.199	-0.528
C	-2.059	1.241	-0.744
H	-1.947	5.671	-2.135
H	-6.409	6.926	-3.160
H	-3.968	6.889	-2.802
H	-6.712	2.813	-1.911
H	-7.783	4.888	-2.715
H	-4.666	1.597	-1.231
H	-2.680	0.365	-0.555
H	-0.218	0.298	-0.164
C	0.444	4.731	-1.540
H	0.000	5.650	-1.133
H	1.391	4.574	-1.010
C	0.721	4.894	-3.039
H	-0.210	5.023	-3.604
H	1.363	5.763	-3.223
H	1.226	4.001	-3.426

Table S98 Frequencies (cm^{-1}) of α-ethylantracene radical, calculated at the M06-2X/cc-pVDZ level of theory.

72	87	108	149	184	227	237	278
316	346	395	425	444	488	507	517
546	579	623	630	649	725	754	770
786	789	807	816	858	868	913	915
934	949	986	993	1012	1016	1036	1082
1085	1137	1146	1159	1179	1186	1221	1267
1288	1290	1326	1347	1364	1381	1385	1433
1449	1471	1472	1477	1485	1489	1518	1609
1634	1658	1685	1710	3066	3074	3122	3148
3150	3192	3197	3199	3202	3214	3216	3220

3227

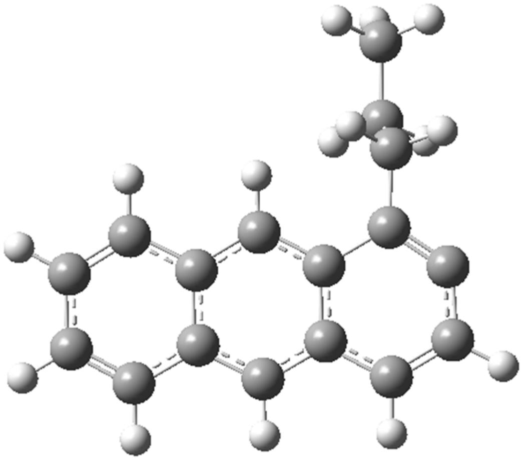

Figure S50 Visualization of the optimized structure of α-propylantracene radical, calculated at the M06-2X/cc-pVDZ level of theory.

Table S99 Geometry (Å) of α-propylantracene radical, calculated at the M06-2X/cc-pVDZ level of theory

Atom	x	y	z
C	-5.335	-2.453	0.067
C	-3.969	-2.399	0.015
C	-3.287	-1.137	-0.002
C	-4.044	0.089	0.040
C	-5.492	0.021	0.097
C	-6.023	-1.222	0.106
C	-1.891	-1.080	-0.063
C	-3.355	1.306	0.017
C	-1.957	1.360	-0.046
C	-1.205	0.138	-0.086
C	0.224	0.207	-0.150
H	0.789	-0.725	-0.180
C	0.865	1.412	-0.172
C	0.117	2.630	-0.133
C	-1.247	2.604	-0.072
H	-1.324	-2.011	-0.097
H	-5.867	-3.402	0.078
H	-3.377	-3.313	-0.016
H	-3.906	2.245	0.048
H	1.952	1.454	-0.221
H	0.645	3.582	-0.152
H	-1.820	3.531	-0.042
C	-6.346	1.261	0.181
H	-5.994	2.008	-0.545
H	-7.372	0.997	-0.106
C	-6.364	1.872	1.588

**H**	-5.335	2.058	1.924
**H**	-6.788	1.132	2.281
**C**	-7.174	3.163	1.636
**H**	-7.197	3.582	2.649
**H**	-6.743	3.919	0.966
**H**	-8.210	2.986	1.319

Table S100 Frequencies (cm^{-1}) of α-propylantracene radical, calculated at the M06-2X/cc-pVDZ level of theory.

51	60	90	108	135	201	227	248
273	292	307	340	401	423	442	488
513	530	555	582	622	636	649	732
744	755	774	789	811	817	854	869
874	904	913	918	935	986	994	1004
1013	1036	1071	1091	1114	1137	1146	1159
1179	1186	1218	1247	1281	1288	1294	1308
1337	1362	1369	1384	1394	1433	1450	1469
1471	1477	1477	1486	1488	1519	1609	1635
1658	1685	1710	3056	3058	3074	3099	3116
3134	3143	3192	3198	3198	3202	3211	3216
3220	3228						

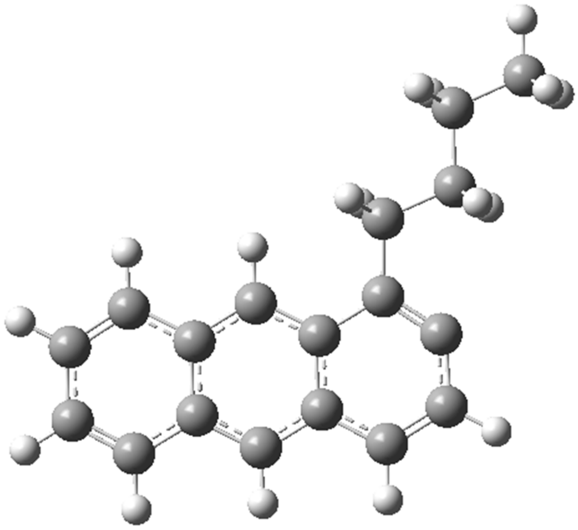

Figure S51 Visualization of the optimized structure of α-butylantracene radical, calculated at the M06-2X/cc-pVDZ level of theory.

Table S101 Geometry (Å) of α-butylantracene radical, calculated at the M06-2X/cc-pVDZ level of theory

Atom	x	y	z
**C**	-5.246	-2.204	-0.014
**C**	-3.878	-2.187	-0.009
**C**	-3.164	-0.943	-0.010
**C**	-3.891	0.302	-0.016
**C**	-5.342	0.272	-0.020

**C**	-5.905	-0.956	-0.019
**C**	-1.766	-0.920	-0.006
**C**	-3.174	1.502	-0.016
**C**	-1.773	1.522	-0.012
**C**	-1.051	0.283	-0.006
**C**	0.381	0.316	-0.002
**H**	0.924	-0.629	0.002
**C**	1.052	1.505	-0.003
**C**	0.333	2.740	-0.008
**C**	-1.033	2.748	-0.012
**H**	-1.220	-1.864	-0.002
**H**	-5.803	-3.139	-0.013
**H**	-3.310	-3.117	-0.005
**H**	-3.706	2.452	-0.020
**H**	2.141	1.520	0.001
**H**	0.885	3.679	-0.009
**H**	-1.583	3.688	-0.016
**C**	-6.150	1.545	-0.026
**H**	-5.871	2.147	0.853
**H**	-5.865	2.143	-0.906
**C**	-7.658	1.319	-0.030
**H**	-7.932	0.719	-0.910
**H**	-7.938	0.723	0.851
**C**	-8.443	2.627	-0.036
**H**	-8.154	3.218	-0.917
**H**	-8.160	3.222	0.845
**C**	-9.952	2.402	-0.040
**H**	-10.499	3.352	-0.044
**H**	-10.257	1.831	-0.926
**H**	-10.263	1.836	0.848

Table S102 Frequencies (cm^{-1}) of α-butylantracene radical, calculated at the M06-2X/cc-pVDZ level of theory.

40	64	70	96	102	143	177	205
246	258	263	288	288	389	407	409
488	491	514	533	557	588	596	629
656	726	754	754	763	789	793	812
813	857	870	912	915	917	930	941
987	988	995	1015	1036	1060	1095	1115
1120	1137	1145	1156	1179	1190	1213	1228
1251	1287	1289	1300	1310	1317	1357	1364
1381	1395	1407	1432	1451	1462	1467	1474
1477	1477	1486	1488	1519	1610	1634	1659
1684	1710	3034	3047	3056	3059	3067	3080
3103	3132	3141	3192	3198	3200	3202	3213
3216	3221	3228					

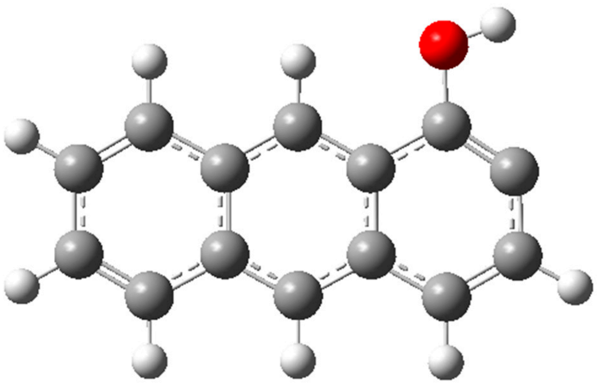

Figure S52 Visualization of the optimized structure of α-hydroxyantracene radical, calculated at the M06-2X/cc-pVDZ level of theory.

Table S103 Geometry (Å) of α-hydroxyantracene radical, calculated at the M06-2X/cc-pVDZ level of theory

Atom	x	y	z
C	3.773	0.916	0.000
C	2.527	1.476	0.000
C	1.354	0.655	0.000
C	1.510	-0.772	0.000
C	2.833	-1.321	0.000
C	3.928	-0.505	0.000
C	0.067	1.207	0.000
C	0.372	-1.586	0.000
C	-0.918	-1.045	0.000
C	-1.062	0.386	0.000
C	-2.393	0.939	0.000
C	-3.434	0.073	0.000
C	-3.336	-1.331	0.000
C	-2.082	-1.883	0.000
H	-0.058	2.288	0.000
H	4.658	1.550	0.000
H	2.404	2.559	0.000
H	2.948	-2.405	0.000
H	4.929	-0.933	0.000
H	0.493	-2.670	0.000
H	-1.954	-2.965	0.000
H	-4.229	-1.954	0.000
O	-2.498	2.297	0.000
H	-3.433	2.532	0.000

Table S104 Frequencies (cm^{-1}) of α-hydroxyantracene radical, calculated at the M06-2X/cc-pVDZ level of theory.

93	100	197	205	262	290	328	392
396	425	478	490	509	522	553	578
624	625	647	743	745	770	787	811

815	858	873	911	922	939	974	995
1015	1034	1038	1131	1143	1159	1180	1192
1226	1271	1288	1293	1355	1370	1412	1444
1460	1478	1488	1525	1608	1635	1657	1694
1710	3196	3198	3203	3206	3216	3223	3224
3228	3866						

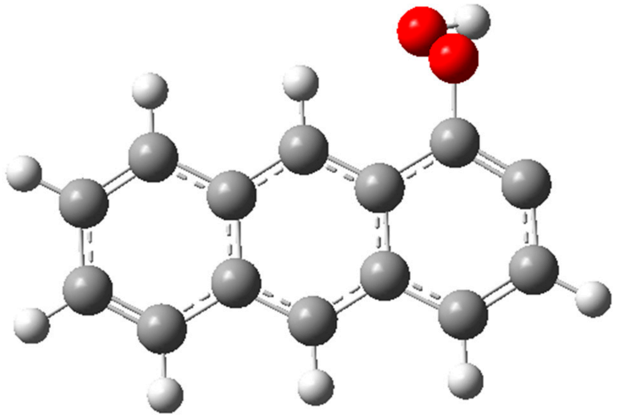

Figure S53 Visualization of the optimized structure of α-peroxyantracene radical, calculated at the M06-2X/cc-pVDZ level of theory.

Table S105 Geometry (Å) of α-peroxyantracene radical, calculated at the M06-2X/cc-pVDZ level of theory

Atom	x	y	z
C	3.982	-1.249	0.068
C	2.675	-1.646	0.069
C	1.617	-0.682	0.037
C	1.955	0.714	0.004
C	3.337	1.089	0.003
C	4.318	0.140	0.033
C	0.271	-1.068	0.035
C	0.930	1.666	-0.022
C	-0.416	1.289	-0.019
C	-0.744	-0.111	0.006
C	-2.139	-0.474	0.011
C	-3.063	0.510	-0.003
C	-2.784	1.892	-0.009
C	-1.467	2.267	-0.025
H	0.009	-2.125	0.053
H	4.779	-1.990	0.092
H	2.414	-2.703	0.094
H	3.589	2.149	-0.023
H	5.366	0.436	0.032
H	1.187	2.726	-0.041
H	-1.196	3.322	-0.037
H	-3.585	2.629	-0.008

**O**	-2.512	-1.800	0.108
**O**	-2.157	-2.453	-1.120
**H**	-2.994	-2.405	-1.606

Table S106 Frequencies (cm-1) of α-peroxyantracene radical, calculated at the M06-2X/cc-pVDZ level of theory.

82	87	102	158	195	238	259	280
319	360	396	433	481	489	510	538
546	582	624	648	668	742	756	778
787	810	822	864	872	912	919	937
961	986	995	1015	1036	1039	1134	1144
1160	1181	1193	1221	1288	1289	1339	1364
1377	1406	1435	1454	1477	1486	1519	1604
1636	1654	1689	1712	3196	3199	3203	3205
3217	3225	3226	3229	3810			

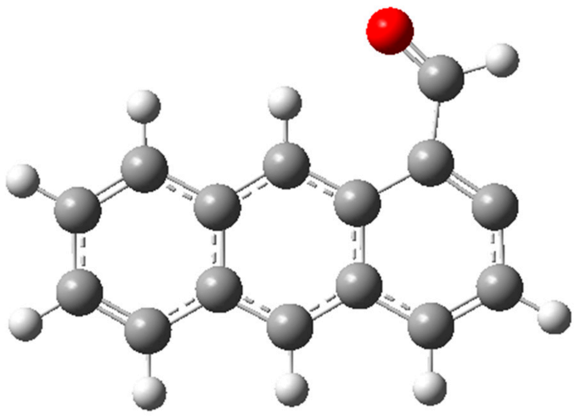

Figure S54 Visualization of the optimized structure of α-antraldehyde radical, calculated at the M06-2X/cc-pVDZ level of theory.

Table S107 Geometry (Å) of α-antraldehyde radical, calculated at the M06-2X/cc-pVDZ level of theory

Atom	x	y	z
**C**	3.804	1.100	0.000
**C**	2.508	1.532	0.000
**C**	1.425	0.596	0.000
**C**	1.728	-0.805	0.000
**C**	3.098	-1.220	0.000
**C**	4.104	-0.297	0.000
**C**	0.089	1.026	0.000
**C**	0.673	-1.724	0.000
**C**	-0.659	-1.303	0.000
**C**	-0.962	0.107	0.000
**C**	-2.363	0.495	0.000
**C**	-3.279	-0.508	0.000
**C**	-3.036	-1.892	0.000

**C**	-1.719	-2.270	0.000
**H**	-0.131	2.090	0.000
**H**	4.620	1.821	0.000
**H**	2.275	2.596	0.000
**H**	3.321	-2.287	0.000
**H**	5.144	-0.620	0.000
**H**	0.894	-2.792	0.000
**H**	-1.453	-3.328	0.000
**H**	-3.845	-2.619	0.000
**C**	-2.874	1.886	0.000
**O**	-2.202	2.893	0.000
**H**	-3.984	1.946	0.000

Table S108 Frequencies (cm^{-1}) of α-antraldehyde radical, calculated at the M06-2X/cc-pVDZ level of theory.

70	97	133	165	218	255	272	308
375	402	410	459	490	521	544	553
605	622	634	662	739	758	773	794
813	818	868	877	913	928	963	997
1003	1018	1031	1038	1043	1132	1148	1160
1170	1185	1228	1288	1296	1350	1364	1376
1407	1440	1454	1475	1481	1517	1602	1634
1646	1678	1712	1811	2980	3192	3199	3200
3206	3219	3230	3231	3252			

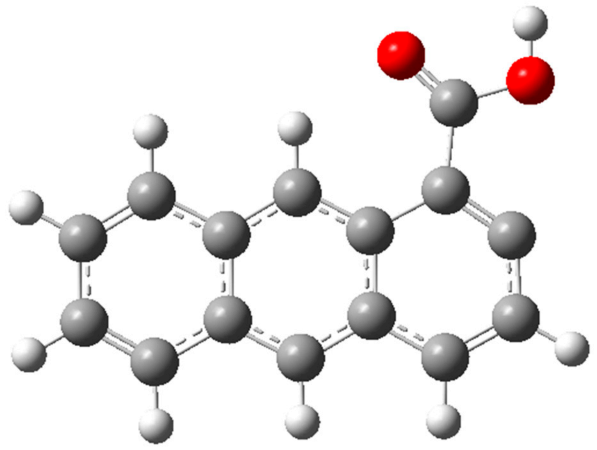

Figure S55 Visualization of the optimized structure of α-antracenic acid radical, calculated at the M06-2X/cc-pVDZ level of theory.

Table S109 Geometry (Å) of α-antracenic acid radical, calculated at the M06-2X/cc-pVDZ level of theory

Atom	x	y	z
**C**	-3.951	-1.436	0.000
**C**	-2.613	-1.711	0.000
**C**	-1.648	-0.654	0.000

**C**	-2.116	0.701	0.000
**C**	-3.525	0.952	0.000
**C**	-4.415	-0.084	0.000
**C**	-0.272	-0.923	0.000
**C**	-1.176	1.736	0.000
**C**	0.198	1.477	0.000
**C**	0.668	0.112	0.000
**C**	2.107	-0.095	0.000
**C**	2.896	1.008	0.000
**C**	2.482	2.350	0.000
**C**	1.130	2.566	0.000
**H**	0.067	-1.954	0.000
**H**	-4.676	-2.249	0.000
**H**	-2.255	-2.741	0.000
**H**	-3.873	1.985	0.000
**H**	-5.486	0.113	0.000
**H**	-1.520	2.771	0.000
**H**	0.734	3.583	0.000
**H**	3.197	3.170	0.000
**C**	2.744	-1.439	0.000
**O**	2.178	-2.507	0.000
**O**	4.093	-1.353	0.000
**H**	4.423	-2.263	0.000

Table S110 Frequencies (cm^{-1}) of α-antracenic acid radical, calculated at the M06-2X/cc-pVDZ level of theory.

**46**	69	100	145	172	252	264	287
**321**	371	402	421	490	512	527	527
**546**	586	622	625	641	657	738	756
**763**	794	804	807	840	861	878	912
**930**	962	978	999	1008	1019	1037	1129
**1139**	1151	1165	1184	1195	1232	1288	1294
**1314**	1362	1364	1414	1436	1450	1476	1483
**1517**	1605	1633	1649	1679	1709	1833	3195
**3198**	3200	3206	3217	3229	3229	3261	3822

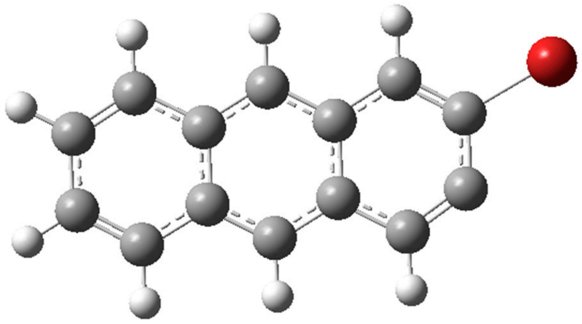

Figure S56 Visualization of the optimized structure of β-bromoantracene radical, calculated at the M06-2X/cc-pVDZ level of theory.

Table S111 Geometry (Å) of β-bromoantracene radical, calculated at the M06-2X/cc-pVDZ level of theory

Atom	x	y	z
C	-4.981	1.285	0.000
C	-3.677	1.688	0.000
C	-2.613	0.729	0.000
C	-2.944	-0.669	0.000
C	-4.324	-1.051	0.000
C	-5.310	-0.106	0.000
C	-1.269	1.116	0.000
C	-1.914	-1.617	0.000
C	-0.572	-1.230	0.000
C	-0.239	0.171	0.000
C	1.138	0.576	0.000
H	1.386	1.637	0.000
C	2.123	-0.370	0.000
C	0.498	-2.195	0.000
H	-1.020	2.177	0.000
H	-5.782	2.023	0.000
H	-3.422	2.748	0.000
H	-4.571	-2.113	-0.001
H	-6.356	-0.408	0.000
H	-2.164	-2.678	0.000
H	0.267	-3.260	0.000
Br	3.959	0.100	0.001
C	1.762	-1.729	0.000

Table S112 Frequencies (cm^{-1}) of β-bromoantracene radical, calculated at the M06-2X/cc-pVDZ level of theory.

56	118	129	165	236	266	294	313
388	419	452	480	491	543	550	606
620	636	695	743	762	779	790	840
851	868	890	895	916	934	944	994
1016	1036	1086	1137	1149	1175	1188	1237
1279	1284	1298	1360	1368	1415	1451	1471
1481	1519	1607	1635	1649	1678	1712	3193
3195	3198	3199	3201	3212	3218	3230	

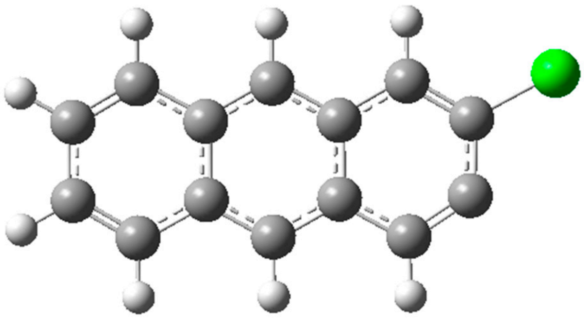

Figure S57 Visualization of the optimized structure of β-chloroantracene radical, calculated at the M06-2X/cc-pVDZ level of theory.

Table S113 Geometry (Å) of β-chloroantracene radical, calculated at the M06-2X/cc-pVDZ level of theory

Atom	x	y	z
C	-5.900	5.973	-2.832
C	-4.555	5.968	-2.598
C	-3.897	4.783	-2.135
C	-4.678	3.598	-1.920
C	-6.086	3.644	-2.178
C	-6.677	4.793	-2.619
C	-2.520	4.751	-1.887
C	-4.047	2.433	-1.468
C	-2.673	2.402	-1.221
C	-1.887	3.589	-1.435
C	-0.475	3.564	-1.183
H	0.119	4.462	-1.345
C	0.116	2.415	-0.742
C	-0.682	1.271	-0.543
C	-2.010	1.210	-0.755
H	-1.928	5.653	-2.050
H	-6.390	6.880	-3.185
H	-3.958	6.866	-2.760
H	-6.676	2.742	-2.014
H	-7.749	4.818	-2.812
H	-4.640	1.532	-1.305
H	-2.587	0.301	-0.586
Cl	1.827	2.334	-0.422

Table S114 Frequencies (cm^{-1}) of β-chloroantracene radical, calculated at the M06-2X/cc-pVDZ level of theory.

66	117	159	174	267	283	321	330
390	424	474	480	491	546	562	614
625	639	717	742	763	780	790	843
852	869	895	902	921	934	944	995
1016	1038	1104	1140	1150	1178	1191	1240
1282	1287	1299	1363	1370	1422	1454	1474

1483	1520	1610	1637	1651	1682	1711	3194
3196	3198	3202	3202	3213	3216	3228	

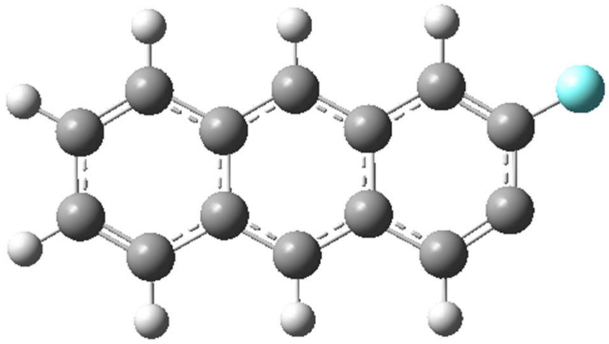

Figure S58 Visualization of the optimized structure of β-fluoroantracene radical, calculated at the M06-2X/cc-pVDZ level of theory.

Table S115 Geometry (Å) of β-fluoroantracene radical, calculated at the M06-2X/cc-pVDZ level of theory

Atom	x	y	z
C	-5.900	5.973	-2.832
C	-4.555	5.968	-2.598
C	-3.897	4.783	-2.135
C	-4.678	3.598	-1.921
C	-6.086	3.644	-2.178
C	-6.678	4.793	-2.619
C	-2.520	4.752	-1.887
C	-4.046	2.434	-1.468
C	-2.672	2.404	-1.221
C	-1.885	3.591	-1.435
C	-0.473	3.563	-1.182
H	0.134	4.453	-1.339
C	0.107	2.413	-0.743
C	-0.685	1.264	-0.541
C	-2.012	1.210	-0.755
H	-1.929	5.654	-2.051
H	-6.390	6.879	-3.184
H	-3.959	6.866	-2.760
H	-6.676	2.742	-2.014
H	-7.750	4.817	-2.812
H	-4.639	1.533	-1.305
H	-2.593	0.303	-0.588
F	1.426	2.353	-0.497

Table S116 Frequencies (cm^{-1}) of β-fluoroantracene radical, calculated at the M06-2X/cc-pVDZ level of theory.

75	117	194	198	271	330	347	373

397	455	481	491	502	556	581	629
634	656	738	765	772	790	796	847
854	871	892	911	934	944	964	998
1019	1037	1138	1146	1173	1182	1220	1236
1280	1298	1310	1365	1374	1441	1464	1480
1485	1522	1621	1648	1658	1693	1711	3196
3197	3199	3202	3203	3217	3219	3229	

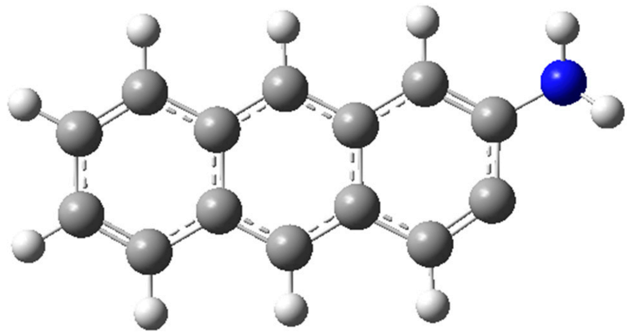

Figure S59 Visualization of the optimized structure of β-aminoantracene radical, calculated at the M06-2X/cc-pVDZ level of theory.

Table S117 Geometry (Å) of β-aminoantracene radical, calculated at the M06-2X/cc-pVDZ level of theory

Atom	x	y	z
C	2.819	0.658	0.002
C	2.733	-0.768	0.001
C	1.684	1.418	0.002
C	1.516	-1.389	0.000
C	0.302	-0.628	-0.001
C	0.391	0.804	0.000
C	-0.790	1.559	0.000
H	-0.728	2.648	0.001
C	-2.041	0.944	-0.001
C	-0.956	-1.241	-0.002
H	1.744	2.507	0.002
H	1.448	-2.477	-0.002
H	-1.015	-2.331	-0.003
C	-2.138	-0.492	-0.002
C	-3.425	-1.121	-0.001
C	-4.585	-0.383	-0.006
H	-3.475	-2.211	-0.003
C	-4.428	1.031	-0.004
C	-3.267	1.706	0.000
H	-3.225	2.796	0.004
N	-5.859	-0.937	-0.073
H	-5.929	-1.895	0.242
H	-6.596	-0.349	0.291

H	3.798	1.135	0.002
H	3.648	-1.359	0.001

Table S118 Frequencies (cm^{-1}) of β-aminoantracene radical, calculated at the M06-2X/cc-pVDZ level of theory.

76	117	188	196	265	309	325	341
373	391	440	476	489	504	537	563
586	630	650	661	738	762	774	790
795	848	851	857	880	910	926	939
952	992	1014	1038	1099	1145	1149	1176
1188	1236	1254	1281	1302	1325	1367	1381
1448	1476	1483	1488	1524	1611	1628	1643
1664	1693	1709	3185	3192	3194	3196	3197
3200	3214	3227	3588	3701			

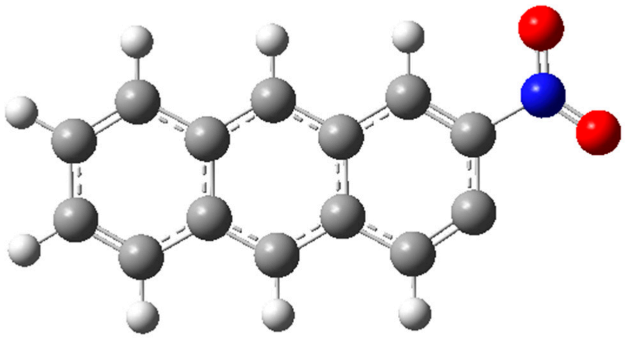

Figure S60 Visualization of the optimized structure of β-nitroantracene radical, calculated at the M06-2X/cc-pVDZ level of theory.

Table S119 Geometry (Å) of β-nitroantracene radical, calculated at the M06-2X/cc-pVDZ level of theory

Atom	x	y	z
C	4.456	-0.833	-0.015
C	3.255	-1.483	0.004
C	2.026	-0.748	0.008
C	2.081	0.687	-0.008
C	3.360	1.331	-0.027
C	4.510	0.596	-0.031
C	0.782	-1.392	0.028
C	0.888	1.416	-0.004
C	-0.355	0.775	0.016
C	-0.409	-0.665	0.032
C	-1.697	-1.316	0.051
H	-1.750	-2.404	0.064
C	-2.796	-0.540	0.054
C	-2.764	0.868	0.038
C	-1.571	1.529	0.019
H	0.745	-2.481	0.040

**H**	5.384	-1.401	-0.018
**H**	3.212	-2.572	0.016
**H**	3.395	2.420	-0.040
**H**	0.926	2.506	-0.016
**H**	-1.555	2.618	0.007
**H**	5.478	1.093	-0.046
**N**	-4.028	1.613	0.043
**O**	-5.049	0.956	0.060
**O**	-3.972	2.828	0.029

Table S120 Frequencies (cm^{-1}) of β-nitroantracene radical, calculated at the M06-2X/cc-pVDZ level of theory.

54	65	120	138	168	263	267	293
305	391	406	464	482	492	531	542
566	593	622	637	711	743	762	779
789	793	839	853	857	877	912	914
940	959	966	999	1022	1038	1127	1144
1153	1179	1191	1238	1282	1297	1301	1368
1371	1435	1457	1468	1479	1484	1527	1622
1640	1658	1679	1710	1712	3197	3198	3200
3203	3204	3219	3225	3230			

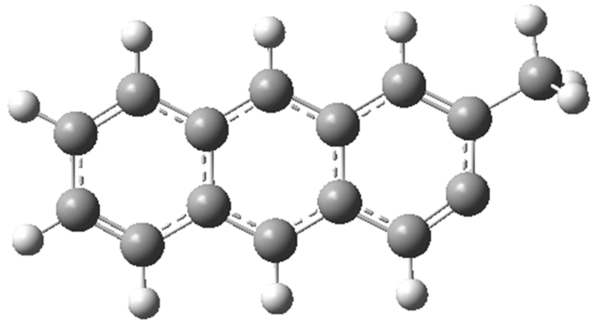

Figure S61 Visualization of the optimized structure of α β-methylantracene radical, calculated at the M06-2X/cc-pVDZ level of theory.

Table S121 Geometry (Å) of β-methylantracene radical, calculated at the M06-2X/cc-pVDZ level of theory

Atom	x	y	z
**C**	4.472	-0.833	-0.015
**C**	3.272	-1.485	0.019
**C**	2.041	-0.755	0.017
**C**	2.088	0.679	-0.021
**C**	3.366	1.326	-0.055
**C**	4.519	0.595	-0.052
**C**	0.797	-1.398	0.051
**C**	0.891	1.405	-0.023
**C**	-0.352	0.765	0.011

C	-0.395	-0.671	0.049
C	-1.686	-1.314	0.083
H	-1.747	-2.401	0.112
C	-2.775	-0.522	0.078
C	-2.803	0.896	0.041
C	-1.579	1.511	0.008
H	0.760	-2.488	0.080
H	5.403	-1.398	-0.013
H	3.233	-2.574	0.048
H	3.398	2.415	-0.084
H	5.485	1.097	-0.079
H	0.929	2.495	-0.052
H	-1.521	2.601	-0.021
C	-4.107	1.645	0.040
H	-4.690	1.406	0.939
H	-4.714	1.361	-0.829
H	-3.935	2.726	0.010

Table S122 Frequencies (cm^{-1}) of β-methylantracene radical, calculated at the M06-2X/cc-pVDZ level of theory.

74	115	134	188	191	267	311	324
370	392	428	484	493	505	544	584
609	629	652	750	764	769	785	793
847	851	866	894	910	934	939	944
995	1016	1018	1039	1054	1142	1149	1176
1192	1202	1248	1282	1300	1301	1366	1371
1396	1432	1459	1460	1472	1482	1486	1522
1612	1641	1658	1692	1710	3065	3133	3161
3184	3192	3195	3197	3197	3202	3215	3227

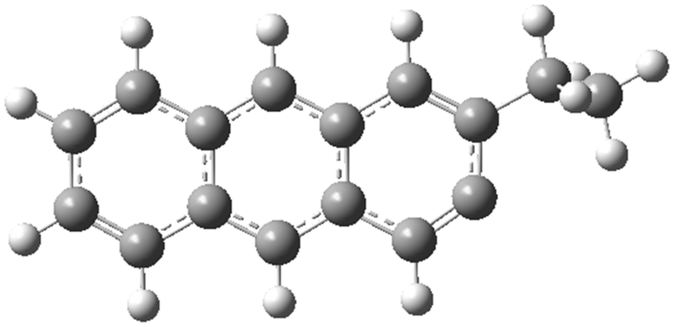

Figure S62 Visualization of the optimized structure of β-ethylantracene radical, calculated at the M06-2X/cc-pVDZ level of theory.

Table S123 Geometry (Å) of β-ethylantracene radical, calculated at the M06-2X/cc-pVDZ level of theory

Atom	x	y	z
C	-5.864	5.931	-2.995

**C**	-4.517	5.903	-2.775
**C**	-3.890	4.748	-2.205
**C**	-4.706	3.615	-1.869
**C**	-6.115	3.684	-2.115
**C**	-6.676	4.804	-2.661
**C**	-2.512	4.694	-1.969
**C**	-4.105	2.480	-1.312
**C**	-2.730	2.429	-1.078
**C**	-1.909	3.562	-1.412
**C**	-0.495	3.508	-1.170
**H**	0.108	4.380	-1.431
**C**	0.108	2.404	-0.624
**C**	-0.759	1.321	-0.321
**C**	-2.091	1.269	-0.507
**H**	-1.894	5.556	-2.225
**H**	-6.330	6.815	-3.429
**H**	-3.894	6.761	-3.029
**H**	-6.731	2.822	-1.859
**H**	-7.749	4.844	-2.843
**H**	-4.724	1.619	-1.056
**H**	-2.690	0.396	-0.247
**C**	1.587	2.306	-0.358
**H**	2.071	3.232	-0.693
**H**	1.748	2.230	0.727
**C**	2.218	1.095	-1.049
**H**	3.293	1.043	-0.840
**H**	1.753	0.167	-0.696
**H**	2.077	1.155	-2.135

Table S124 Frequencies (cm^{-1}) of β-ethylantracene radical, calculated at the M06-2X/cc-pVDZ level of theory.

38	69	114	146	173	234	240	272
314	344	387	411	450	482	491	506
554	583	627	639	665	748	759	762
777	786	792	844	848	868	896	909
931	934	943	992	995	1016	1038	1077
1092	1142	1150	1176	1192	1201	1243	1267
1282	1298	1306	1336	1367	1377	1386	1438
1459	1466	1471	1475	1483	1486	1521	1612
1641	1658	1691	1711	3062	3065	3114	3142
3149	3184	3192	3195	3197	3198	3202	3216
3228							

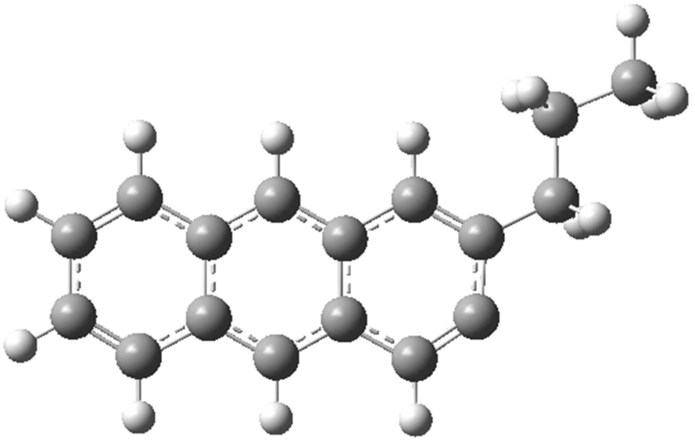

Figure S63 Visualization of the optimized structure of β-propylantracene radical, calculated at the M06-2X/cc-pVDZ level of theory.

Table S125 Geometry (Å) of β-propylantracene radical, calculated at the M06-2X/cc-pVDZ level of theory

Atom	x	y	z
C	-3.983	-2.411	-0.003
C	-2.642	-2.522	-0.016
C	-1.875	-1.300	-0.011
C	-2.588	-0.052	0.007
C	-4.025	-0.052	0.020
C	-4.749	-1.216	0.016
C	-0.479	-1.300	-0.024
C	-1.860	1.142	0.012
C	-0.460	1.147	-0.001
C	0.247	-0.102	-0.019
C	1.679	-0.083	-0.032
H	2.212	-1.033	-0.046
C	2.363	1.100	-0.027
C	1.659	2.343	-0.008
C	0.293	2.365	0.004
H	0.057	-2.249	-0.038
H	-2.128	-3.483	-0.030
H	-4.532	0.913	0.034
H	-2.395	2.092	0.026
H	3.452	1.102	-0.037
H	2.221	3.276	-0.005
H	-0.247	3.312	0.018
C	-6.256	-1.293	0.028
H	-6.576	-1.873	-0.850
H	-6.561	-1.890	0.901
C	-6.985	0.045	0.048
H	-6.674	0.617	0.933
H	-6.690	0.634	-0.832
C	-8.500	-0.137	0.059
H	-9.016	0.830	0.073

**H**	-8.834	-0.686	-0.830
**H**	-8.818	-0.704	0.944

Table S126 Frequencies (cm^{-1}) of β-propylantracene radical, calculated at the M06-2X/cc-pVDZ level of theory.

42	76	92	110	126	190	221	243
270	275	324	355	394	417	439	483
493	533	542	596	610	629	653	738
750	761	774	792	794	846	849	851
875	906	908	921	936	947	964	994
1014	1036	1074	1111	1114	1140	1148	1174
1178	1192	1248	1253	1271	1284	1297	1310
1310	1363	1370	1384	1397	1441	1460	1462
1473	1476	1480	1483	1490	1519	1611	1641
1658	1691	1710	3044	3059	3061	3074	3103
3134	3144	3192	3195	3198	3199	3202	3205
3216	3228						

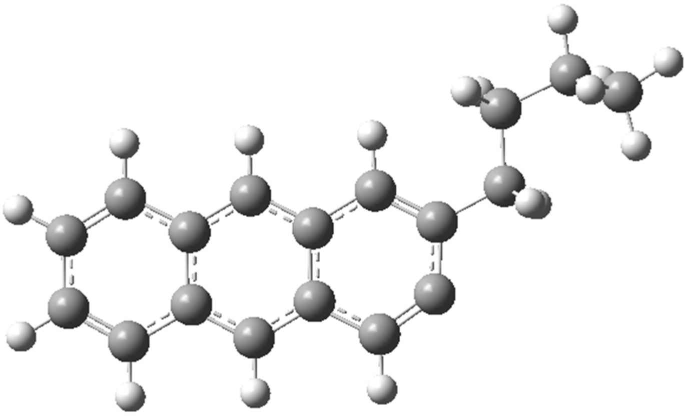

Figure S64 Visualization of the optimized structure of β-butylantracene radical, calculated at the M06-2X/cc-pVDZ level of theory.

Table S127 Geometry (Å) of β-butylantracene radical, calculated at the M06-2X/cc-pVDZ level of theory

Atom	x	y	z
**C**	-3.910	-2.946	0.016
**C**	-2.570	-3.055	-0.026
**C**	-1.803	-1.833	-0.020
**C**	-2.518	-0.586	0.029
**C**	-3.954	-0.588	0.070
**C**	-4.678	-1.752	0.065
**C**	-0.408	-1.830	-0.060
**C**	-1.792	0.609	0.036
**C**	-0.393	0.617	-0.004
**C**	0.316	-0.631	-0.053

**C**	1.747	-0.609	-0.093
**H**	2.282	-1.558	-0.130
**C**	2.429	0.575	-0.085
**C**	1.724	1.817	-0.036
**C**	0.359	1.836	0.003
**H**	0.129	-2.778	-0.097
**H**	-2.056	-4.015	-0.064
**H**	-4.462	0.376	0.109
**H**	-2.328	1.558	0.073
**H**	3.518	0.579	-0.115
**H**	2.285	2.750	-0.031
**H**	-0.183	2.781	0.040
**C**	-6.184	-1.834	0.114
**H**	-6.524	-2.432	-0.745
**H**	-6.456	-2.415	1.006
**C**	-6.911	-0.494	0.119
**H**	-6.633	0.071	1.021
**H**	-6.579	0.098	-0.745
**C**	-8.433	-0.639	0.065
**H**	-8.876	0.364	0.010
**H**	-8.713	-1.155	-0.865
**C**	-9.021	-1.383	1.263
**H**	-10.117	-1.360	1.239
**H**	-8.714	-2.435	1.277
**H**	-8.694	-0.921	2.205

Table S128 Frequencies (cm^{-1}) of β-butylantracene radical, calculated at the M06-2X/cc-pVDZ level of theory.

36	45	81	88	121	133	184	210
222	269	281	324	356	380	394	438
449	483	493	535	542	597	610	629
653	734	748	762	775	782	792	800
848	851	867	890	900	910	932	939
958	965	994	1000	1015	1036	1104	1116
1122	1140	1147	1172	1176	1192	1224	1247
1260	1282	1291	1297	1305	1324	1360	1366
1370	1390	1398	1441	1458	1462	1468	1474
1479	1482	1484	1487	1520	1611	1640	1658
1691	1709	3044	3052	3057	3061	3083	3098
3106	3135	3145	3192	3195	3198	3198	3202
3204	3216	3228					

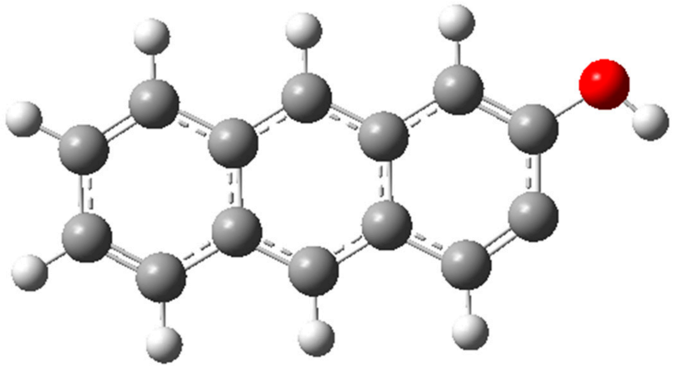

Figure S65 Visualization of the optimized structure of β-hydroxyantracene radical, calculated at the M06-2X/cc-pVDZ level of theory.

Table S129 Geometry (Å) of β-hydroxyantracene radical, calculated at the M06-2X/cc-pVDZ level of theory

Atom	x	y	z
C	-5.917	5.978	-2.800
C	-4.568	5.972	-2.590
C	-3.900	4.785	-2.144
C	-4.679	3.600	-1.922
C	-6.091	3.647	-2.154
C	-6.692	4.797	-2.579
C	-2.519	4.754	-1.922
C	-4.038	2.434	-1.487
C	-2.659	2.406	-1.266
C	-1.873	3.591	-1.487
C	-0.458	3.564	-1.260
H	0.139	4.460	-1.427
C	0.157	2.418	-0.835
C	-0.657	1.276	-0.631
C	-1.987	1.212	-0.818
H	-1.932	5.657	-2.092
H	-6.413	6.887	-3.138
H	-3.975	6.871	-2.758
H	-6.678	2.744	-1.984
H	-7.767	4.823	-2.752
H	-4.626	1.532	-1.317
H	-2.559	0.301	-0.643
O	1.500	2.392	-0.620
H	1.753	1.510	-0.326

Table S130 Frequencies (cm^{-1}) of β-hydroxyantracene radical, calculated at the M06-2X/cc-pVDZ level of theory.

76	117	193	199	269	331	338	351
373	394	450	479	490	506	557	584

630	640	659	736	763	773	791	793
848	851	867	888	910	932	940	961
993	1014	1037	1136	1146	1174	1182	1200
1231	1261	1281	1308	1322	1366	1384	1448
1471	1482	1490	1526	1622	1648	1661	1694
1710	3194	3196	3197	3200	3202	3212	3216
3228	3873						

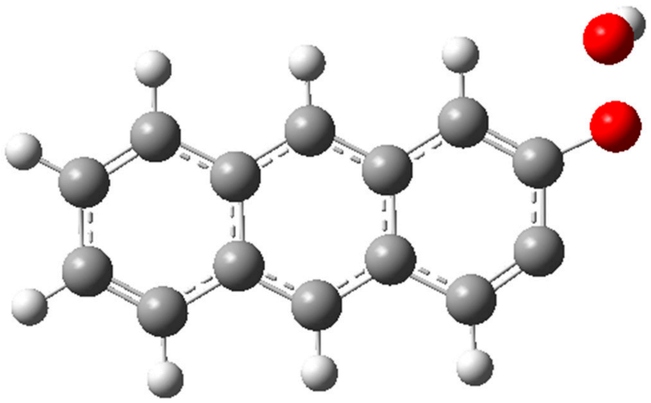

Figure S66 Visualization of the optimized structure of β-peroxyantracene radical, calculated at the M06-2X/cc-pVDZ level of theory.

Table S131 Geometry (Å) of β-peroxyantracene radical, calculated at the M06-2X/cc-pVDZ level of theory

Atom	x	y	z
C	-4.386	-0.903	0.019
C	-3.161	-1.508	0.003
C	-1.959	-0.729	-0.005
C	-2.066	0.702	0.004
C	-3.367	1.298	0.021
C	-4.491	0.521	0.028
C	-0.691	-1.325	-0.021
C	-0.896	1.473	-0.004
C	0.364	0.875	-0.020
C	0.477	-0.559	-0.028
C	1.772	-1.182	-0.044
H	1.850	-2.265	-0.061
C	2.895	-0.405	-0.050
C	2.749	1.005	-0.046
C	1.576	1.659	-0.031
H	-0.616	-2.413	-0.029
H	-5.293	-1.506	0.024
H	-3.078	-2.594	-0.005
H	-3.443	2.385	0.028
H	-5.477	0.985	0.040
H	-0.973	2.561	0.002
H	1.517	2.748	-0.027

**O**	4.195	-0.843	-0.066
**O**	4.240	-2.254	-0.072
**H**	4.548	-2.438	0.828

Table S132 Frequencies (cm^{-1}) of β-peroxyantracene radical, calculated at the M06-2X/cc-pVDZ level of theory.

64	98	137	156	189	218	263	272
346	362	395	414	475	480	490	556
558	599	629	638	648	739	762	773
790	800	849	851	865	884	910	931
941	972	993	1016	1037	1048	1140	1147
1173	1180	1213	1236	1280	1299	1302	1365
1372	1433	1438	1462	1481	1486	1526	1624
1650	1662	1692	1711	3192	3193	3197	3197
3200	3217	3229	3244	3809			

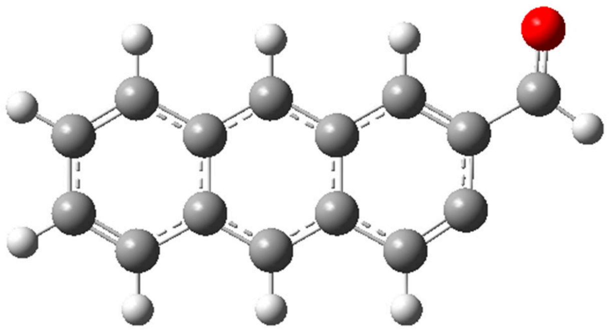

Figure S67 Visualization of the optimized structure of β-antraldehyde radical, calculated at the M06-2X/cc-pVDZ level of theory.

Table S133 Geometry (Å) of β-antraldehyde radical, calculated at the M06-2X/cc-pVDZ level of theory

Atom	x	y	z
**C**	4.458	-0.829	-0.015
**C**	3.259	-1.482	0.020
**C**	2.027	-0.751	0.018
**C**	2.078	0.685	-0.022
**C**	3.357	1.331	-0.057
**C**	4.509	0.599	-0.054
**C**	0.785	-1.396	0.052
**C**	0.884	1.411	-0.024
**C**	-0.360	0.769	0.011
**C**	-0.409	-0.671	0.050
**C**	-1.696	-1.320	0.086
**H**	-1.750	-2.408	0.116
**C**	-2.788	-0.533	0.080
**C**	-2.797	0.882	0.042
**C**	-1.577	1.516	0.008

H	0.750	-2.485	0.082
H	5.388	-1.396	-0.013
H	3.219	-2.571	0.049
H	3.389	2.420	-0.087
H	0.920	2.501	-0.054
H	-1.552	2.606	-0.021
H	5.476	1.099	-0.081
C	-4.066	1.654	0.040
O	-4.115	2.861	0.008
H	-4.993	1.042	0.070

Table S134 Frequencies (cm^{-1}) of β-antraldehyde radical, calculated at the M06-2X/cc-pVDZ level of theory.

63	101	136	146	185	248	277	316
359	396	406	472	482	492	539	565
614	622	638	646	748	763	774	792
808	849	849	872	910	911	937	961
977	996	1018	1036	1037	1137	1147	1168
1179	1194	1237	1280	1293	1301	1363	1372
1392	1443	1456	1475	1483	1518	1604	1636
1655	1679	1712	1826	2985	3194	3196	3198
3199	3201	3202	3219	3231			

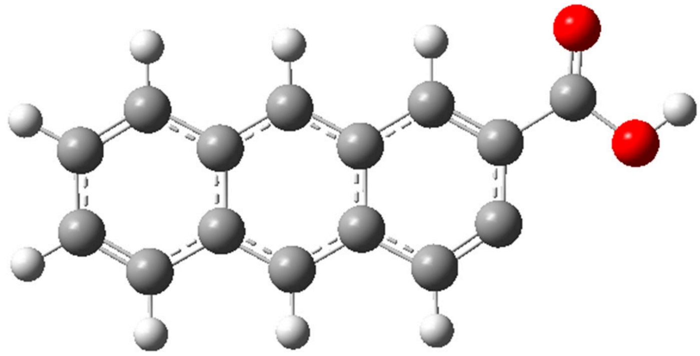

Figure S68 Visualization of the optimized structure of β-antracenic acid radical, calculated at the M06-2X/cc-pVDZ level of theory.

Table S135 Geometry (Å) of β-antracenic acid radical, calculated at the M06-2X/cc-pVDZ level of theory

Atom	x	y	z
C	-4.486	-1.198	0.000
C	-3.201	-1.658	0.000
C	-2.097	-0.744	0.000
C	-2.364	0.667	0.000
C	-3.726	1.108	0.000
C	-4.752	0.207	0.000
C	-0.771	-1.189	0.000

**C**	-1.295	1.571	0.000
**C**	0.029	1.128	0.000
**C**	0.299	-0.287	0.000
**C**	1.653	-0.746	0.000
**H**	1.869	-1.815	0.000
**C**	2.697	0.144	0.000
**C**	2.384	1.524	0.000
**C**	1.143	2.045	0.000
**H**	-0.565	-2.260	0.000
**H**	-5.319	-1.900	0.000
**H**	-2.992	-2.727	0.000
**H**	-3.927	2.179	0.000
**H**	-5.784	0.554	0.000
**H**	-1.501	2.642	0.000
**H**	0.956	3.119	0.000
**C**	4.094	-0.365	0.000
**O**	4.403	-1.531	0.000
**O**	4.999	0.634	0.000
**H**	5.873	0.216	0.000

Table S136 Frequencies (cm^{-1}) of β-antracenic acid radical, calculated at the M06-2X/cc-pVDZ level of theory.

55	67	121	124	164	248	268	284
301	393	398	459	483	493	515	526
560	582	618	634	648	665	750	763
764	780	794	797	843	850	876	910
916	938	940	973	998	1020	1037	1127
1143	1149	1177	1187	1220	1241	1280	1293
1301	1359	1368	1397	1440	1460	1479	1485
1521	1611	1639	1658	1685	1711	1846	3194
3195	3198	3200	3202	3205	3217	3229	3822

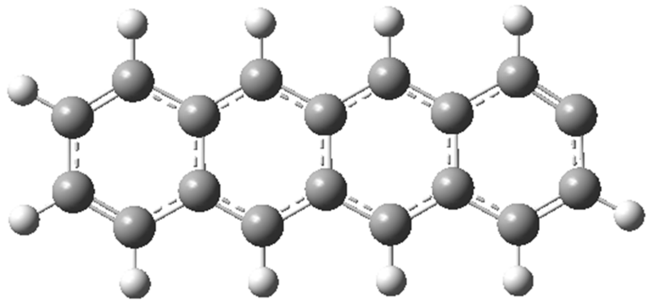

Figure S69 Visualization of the optimized structure of tetracene radical, calculated at the M06-2X/cc-pVDZ level of theory.

Table S137 Geometry (Å) of tetracene radical, calculated at the M06-2X/cc-pVDZ level of theory

Atom	x	y	z
C	-3.649	0.729	0.000
C	-2.473	1.416	0.000
C	-1.214	0.722	0.000
C	-1.220	-0.723	0.000
C	-2.486	-1.404	0.000
C	-3.655	-0.707	0.000
C	-0.001	1.399	0.000
C	-0.014	-1.412	0.000
C	1.223	-0.734	0.000
C	1.228	0.709	0.000
C	2.466	1.387	0.000
H	2.470	2.478	0.000
C	3.673	0.701	0.000
C	2.454	-1.425	0.000
H	0.004	2.490	0.000
H	-4.597	1.266	0.000
H	-2.465	2.506	0.000
H	-2.488	-2.494	0.000
H	-4.608	-1.235	0.000
H	-0.020	-2.503	0.000
H	2.450	-2.515	-0.001
C	3.664	-0.746	0.000
C	4.933	-1.441	0.000
C	6.053	-0.696	0.000
H	4.948	-2.531	-0.001
C	6.117	0.720	0.000
C	4.932	1.400	0.000
H	7.071	1.244	0.000
H	4.919	2.490	0.000

Table S138 Frequencies (cm^{-1}) of tetracene radical, calculated at the M06-2X/cc-pVDZ level of theory.

53	91	154	171	195	279	311	320
327	387	452	468	483	497	498	526
556	569	608	622	636	732	757	764
769	776	780	790	794	846	862	880
882	892	915	922	944	947	990	995
1012	1027	1038	1118	1147	1155	1174	1186
1210	1222	1275	1284	1297	1323	1330	1349
1384	1436	1450	1455	1481	1489	1502	1589
1613	1627	1639	1678	1689	1718	3195	3196
3197	3199	3200	3200	3202	3204	3218	3225
3229							

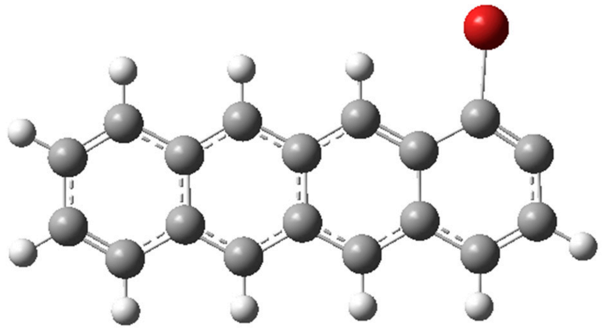

Figure S70 Visualization of the optimized structure of α-bromotetracene radical, calculated at the M06-2X/cc-pVDZ level of theory.

Table S139 Geometry (Å) of α-bromotetracene radical, calculated at the M06-2X/cc-pVDZ level of theory

Atom	x	y	z
C	-5.901	-0.038	0.000
C	-4.939	0.926	0.000
C	-3.545	0.575	0.000
C	-3.184	-0.824	0.000
C	-4.234	-1.805	0.000
C	-5.543	-1.427	0.000
C	-2.545	1.539	0.000
C	-1.842	-1.185	0.000
C	-0.820	-0.214	0.000
C	-1.181	1.181	0.000
C	-0.154	2.149	0.000
H	-0.424	3.206	0.000
C	1.187	1.793	0.000
C	0.546	-0.575	0.000
H	-2.817	2.595	0.000
H	-6.954	0.240	0.000
H	-5.208	1.982	0.000
H	-3.959	-2.859	0.000
H	-6.330	-2.180	0.000
H	-1.570	-2.241	0.000
H	0.813	-1.630	0.000
C	1.544	0.386	0.000
C	2.959	0.077	0.000
C	3.846	1.081	0.000
C	3.538	2.462	0.000
C	2.216	2.800	0.000
H	4.324	3.215	0.000
H	1.914	3.847	0.000
Br	3.570	-1.722	0.000

Table S140 Frequencies (cm-1) of α-bromotetracene radical, calculated at the M06-2X/cc-pVDZ level of theory.

54	62	99	141	161	192	218	290
300	323	331	356	402	475	482	496
502	522	557	568	569	607	634	666
739	759	765	775	777	788	804	806
862	878	890	911	912	928	943	947
988	997	1015	1027	1096	1130	1149	1157
1177	1198	1222	1249	1283	1295	1321	1330
1350	1383	1428	1451	1452	1482	1488	1501
1589	1608	1629	1635	1674	1688	1718	3197
3199	3200	3201	3205	3206	3220	3227	3229
3230							

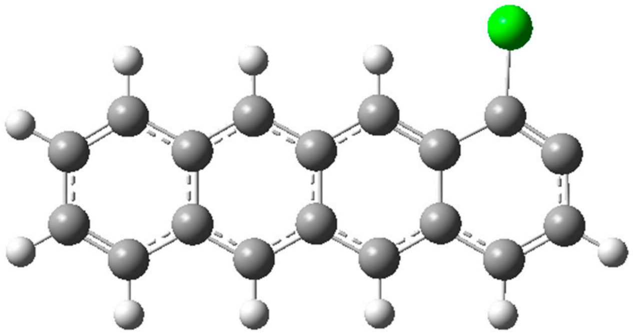

Figure S71 Visualization of the optimized structure of α-chlorotetracene acid radical, calculated at the M06-2X/cc-pVDZ level of theory.

Table S141 Geometry (Å) of α-chlorotetracene radical, calculated at the M06-2X/cc-pVDZ level of theory

Atom	x	y	z
C	5.423	-0.333	0.000
C	4.358	-1.182	0.000
C	3.012	-0.675	0.000
C	2.812	0.756	0.000
C	3.967	1.612	0.000
C	5.224	1.088	0.000
C	1.909	-1.519	0.000
C	1.519	1.266	0.000
C	0.394	0.418	0.000
C	0.595	-1.010	0.000
C	-0.534	-1.856	0.000
H	-0.385	-2.937	0.000
C	-1.827	-1.351	0.000
C	-0.923	0.931	0.000
H	2.060	-2.600	0.000
H	6.438	-0.728	0.000
H	4.505	-2.261	0.000
H	3.812	2.691	0.000

**H**	6.091	1.747	0.000
**H**	1.369	2.346	0.000
**H**	-1.070	2.009	0.000
**C**	-2.021	0.086	0.000
**C**	-3.389	0.559	0.000
**C**	-4.389	-0.337	0.000
**C**	-4.239	-1.744	0.000
**C**	-2.965	-2.233	0.000
**H**	-5.106	-2.401	0.000
**H**	-2.787	-3.308	0.000
**Cl**	-3.731	2.271	0.000

Table S142 Frequencies (cm^{-1}) of α-chlorotetracene radical, calculated at the M06-2X/cc-pVDZ level of theory.

58	62	124	145	162	215	221	291
314	331	365	395	402	480	482	496
504	522	559	572	573	607	634	682
740	760	765	776	777	788	806	813
861	883	888	910	914	942	944	945
985	996	1015	1030	1104	1131	1152	1156
1176	1197	1222	1251	1284	1293	1321	1332
1351	1382	1430	1452	1454	1482	1487	1502
1589	1609	1628	1636	1676	1691	1715	3194
3196	3197	3198	3202	3202	3216	3225	3226
3228							

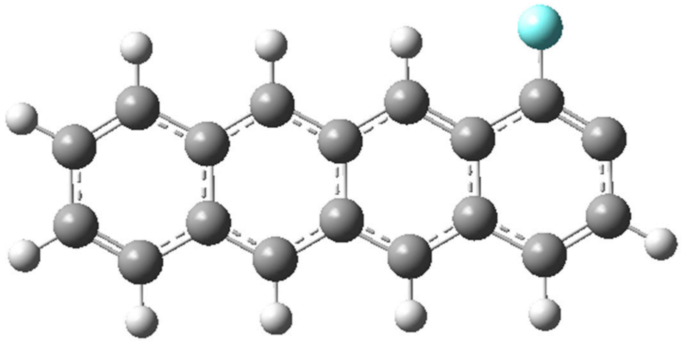

Figure S72 Visualization of the optimized structure of α-fluorotetracene radical, calculated at the M06-2X/cc-pVDZ level of theory.

Table S143 Geometry (Å) of α-fluorotetracene radical, calculated at the M06-2X/cc-pVDZ level of theory

Atom	x	y	z
**C**	5.152	-0.556	0.000
**C**	4.026	-1.322	0.000
**C**	2.722	-0.715	0.000
**C**	2.630	0.727	0.000
**C**	3.847	1.493	0.000

**C**	5.061	0.877	0.000
**C**	1.559	-1.474	0.000
**C**	1.381	1.333	0.000
**C**	0.194	0.571	0.000
**C**	0.285	-0.869	0.000
**C**	-0.901	-1.633	0.000
**H**	-0.829	-2.721	0.000
**C**	-2.154	-1.035	0.000
**C**	-1.080	1.180	0.000
**H**	1.629	-2.563	0.000
**H**	6.134	-1.027	0.000
**H**	4.092	-2.410	0.000
**H**	3.774	2.581	0.000
**H**	5.975	1.468	0.000
**H**	1.311	2.421	0.000
**H**	-1.158	2.266	0.000
**C**	-2.232	0.410	0.000
**C**	-3.545	0.997	0.000
**C**	-4.630	0.206	0.000
**C**	-4.590	-1.209	0.000
**C**	-3.365	-1.814	0.000
**H**	-5.510	-1.792	0.000
**H**	-3.286	-2.900	0.000
**F**	-3.629	2.340	0.000

Table S144 Frequencies (cm^{-1}) of α-fluorotetracene radical, calculated at the M06-2X/cc-pVDZ level of theory.

54	74	141	153	173	237	244	298
314	332	397	407	476	482	496	507
520	520	563	588	603	604	634	731
742	760	765	774	777	791	809	836
860	881	888	912	917	944	949	981
996	1014	1026	1037	1121	1135	1153	1173
1191	1204	1218	1256	1286	1300	1324	1345
1366	1393	1430	1450	1459	1483	1489	1503
1590	1612	1626	1648	1684	1700	1716	3194
3196	3197	3199	3202	3206	3217	3222	3225
3229							

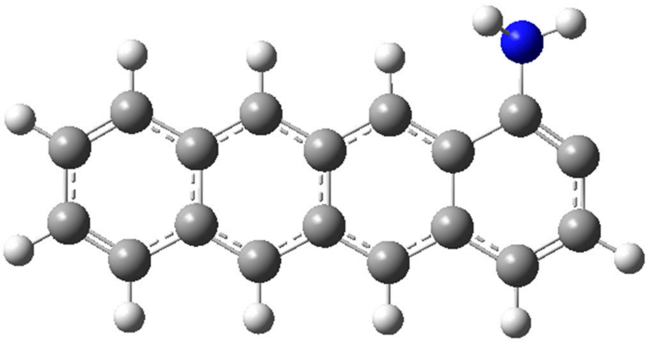

Figure S73 Visualization of the optimized structure of α-aminotetracene radical, calculated at the M06-2X/cc-pVDZ level of theory.

Table S145 Geometry (Å) of α-aminotetracene radical, calculated at the M06-2X/cc-pVDZ level of theory

Atom	x	y	z
C	-5.165	-0.537	0.015
C	-4.043	-1.309	0.036
C	-2.736	-0.712	0.018
C	-2.635	0.729	-0.021
C	-3.846	1.503	-0.042
C	-5.065	0.894	-0.025
C	-1.577	-1.478	0.036
C	-1.380	1.326	-0.038
C	-0.199	0.558	-0.014
C	-0.300	-0.880	0.019
C	0.885	-1.645	0.020
H	0.810	-2.733	0.027
C	2.142	-1.055	0.003
C	1.082	1.155	-0.026
H	-1.653	-2.565	0.062
H	-6.150	-1.001	0.028
H	-4.116	-2.396	0.065
H	-3.767	2.589	-0.073
H	-5.975	1.492	-0.042
H	-1.305	2.414	-0.067
H	1.140	2.241	-0.085
C	2.239	0.391	-0.003
C	3.561	1.000	0.010
C	4.615	0.152	-0.027
C	4.566	-1.258	-0.051
C	3.337	-1.856	-0.024
H	5.481	-1.847	-0.080
H	3.243	-2.941	-0.030
N	3.679	2.395	-0.002
H	3.039	2.862	0.630
H	4.630	2.702	0.160

Table S146 Frequencies (cm^{-1}) of α-aminotetracene radical, calculated at the M06-2X/cc-pVDZ level of theory.

55	72	144	150	169	225	258	296
314	325	337	398	406	471	481	493
495	518	524	562	585	589	609	634
698	729	750	756	764	776	778	789
809	844	859	877	884	905	918	936
941	972	996	1014	1016	1027	1115	1127
1154	1159	1173	1198	1216	1233	1265	1290
1309	1322	1349	1371	1401	1434	1450	1455
1483	1486	1501	1587	1609	1621	1627	1648
1682	1694	1716	3192	3194	3194	3197	3201
3204	3210	3216	3221	3228	3560	3665	

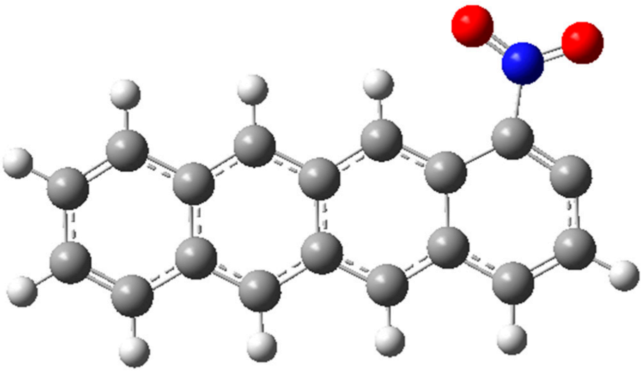

Figure S74 Visualization of the optimized structure of α-nitrotetracene radical, calculated at the M06-2X/cc-pVDZ level of theory.

Table S147 Geometry (Å) of α-nitrotetracene radical, calculated at the M06-2X/cc-pVDZ level of theory

Atom	x	y	z
O	4.955	-1.816	-0.007
O	2.950	-2.598	-0.015
N	3.747	-1.678	-0.008
C	1.832	0.064	0.001
C	1.566	1.495	0.010
C	3.240	-0.300	0.001
C	2.644	2.447	0.018
C	0.757	-0.815	-0.007
C	0.256	1.955	0.011
C	4.181	0.668	0.009
C	3.954	2.056	0.018
C	-0.576	-0.343	-0.006
C	-0.840	1.072	0.003
H	2.388	3.507	0.025
H	0.927	-1.885	-0.014
H	0.072	3.030	0.017
H	4.775	2.769	0.024

**C**	-1.665	-1.238	-0.014
**H**	-1.468	-2.311	-0.021
**C**	-2.978	-0.782	-0.014
**C**	-3.240	0.639	-0.005
**C**	-2.174	1.530	0.003
**H**	-2.368	2.603	0.010
**C**	-4.095	-1.686	-0.022
**H**	-3.894	-2.757	-0.028
**C**	-5.373	-1.217	-0.021
**H**	-6.211	-1.912	-0.027
**C**	-5.633	0.195	-0.012
**H**	-6.664	0.546	-0.011
**C**	-4.605	1.089	-0.004
**H**	-4.799	2.161	0.003

Table S148 Frequencies (cm^{-1}) of α-nitrotetracene radical, calculated at the M06-2X/cc-pVDZ level of theory.

20	52	64	121	136	162	219	223
287	304	318	332	361	398	440	480
493	505	526	531	538	572	593	612
634	674	735	759	766	776	779	788
791	807	823	861	863	884	892	917
919	942	956	998	1002	1003	1016	1028
1120	1145	1155	1162	1176	1195	1221	1257
1292	1296	1322	1334	1349	1381	1430	1443
1456	1463	1479	1484	1498	1586	1606	1628
1636	1673	1684	1702	1717	3192	3195	3197
3199	3202	3204	3219	3230	3236	3283	

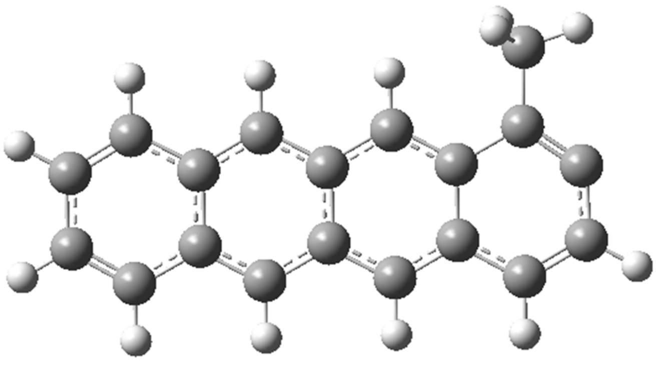

Figure S75 Visualization of the optimized structure of α-methyltetracene radical, calculated at the M06-2X/cc-pVDZ level of theory.

Table S149 Geometry (Å) of α-methyltetracene radical, calculated at the M06-2X/cc-pVDZ level of theory

Atom	x	y	z
**C**	-6.049	-3.034	-0.196

**C**	-4.823	-2.463	-0.356
**C**	-3.622	-3.217	-0.120
**C**	-3.742	-4.597	0.291
**C**	-5.058	-5.156	0.446
**C**	-6.169	-4.405	0.212
**C**	-2.360	-2.657	-0.276
**C**	-2.594	-5.342	0.524
**C**	-1.309	-4.782	0.368
**C**	-1.188	-3.405	-0.042
**C**	0.100	-2.845	-0.197
**H**	0.176	-1.804	-0.507
**C**	1.250	-3.586	0.034
**C**	1.128	-4.970	0.446
**C**	-0.134	-5.527	0.601
**H**	-2.270	-1.615	-0.586
**H**	-6.951	-2.452	-0.378
**H**	-2.684	-6.384	0.834
**C**	2.315	-5.749	0.689
**H**	-5.145	-6.198	0.756
**H**	-7.160	-4.840	0.332
**H**	-0.222	-6.569	0.911
**H**	2.202	-6.788	0.998
**C**	3.553	-5.197	0.536
**C**	3.612	-3.840	0.132
**C**	2.579	-3.014	-0.124
**H**	4.459	-5.773	0.716
**H**	-4.729	-1.422	-0.665
**C**	2.760	-1.582	-0.550
**H**	2.302	-1.406	-1.532
**H**	2.285	-0.899	0.168
**H**	3.824	-1.339	-0.612

Table S150 Frequencies (cm^{-1}) of α-methyltetracene radical, calculated at the M06-2X/cc-pVDZ level of theory.

55	70	142	150	164	219	237	247
291	314	332	376	401	458	481	487
494	522	524	552	570	581	610	634
720	741	758	768	776	776	791	808
833	860	881	884	908	915	942	946
964	988	995	1013	1027	1044	1063	1118
1140	1152	1172	1182	1194	1218	1254	1288
1297	1322	1341	1355	1380	1395	1427	1448
1453	1466	1475	1484	1487	1500	1589	1613
1627	1647	1684	1690	1714	3057	3122	3167
3192	3193	3194	3197	3200	3201	3213	3216
3222	3228						

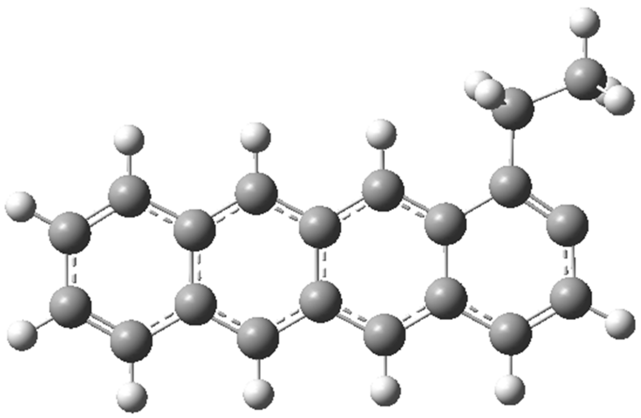

Figure S76 Visualization of the optimized structure of α-ethyltetracene radical, calculated at the M06-2X/cc-pVDZ level of theory.

Table S151 Geometry (Å) of α-ethyltetracene radical, calculated at the M06-2X/cc-pVDZ level of theory

Atom	x	y	z
C	-5.932	-3.108	-0.383
C	-4.762	-2.463	-0.564
C	-3.560	-3.228	-0.257
C	-3.699	-4.593	0.210
C	-5.012	-5.166	0.361
C	-6.127	-4.437	0.069
C	-2.289	-2.689	-0.398
C	-2.564	-5.333	0.506
C	-1.267	-4.794	0.366
C	-1.127	-3.437	-0.098
C	0.166	-2.893	-0.240
H	0.274	-1.866	-0.591
C	1.302	-3.637	0.058
C	1.161	-4.997	0.523
C	-0.110	-5.541	0.666
H	-2.165	-1.665	-0.748
H	-2.673	-6.360	0.857
C	2.349	-5.748	0.825
H	-5.094	-6.194	0.713
H	-7.126	-4.855	0.178
H	-0.217	-6.568	1.017
H	2.238	-6.774	1.175
C	3.583	-5.192	0.677
C	3.724	-3.841	0.215
H	4.721	-3.417	0.104
C	2.625	-3.093	-0.083
H	2.729	-2.066	-0.434
H	4.476	-5.771	0.909
C	-4.677	-1.040	-1.054
H	-4.129	-0.444	-0.309

**H**	-4.068	-1.018	-1.969
**C**	-6.040	-0.411	-1.321
**H**	-6.648	-0.401	-0.408
**H**	-5.927	0.620	-1.673
**H**	-6.588	-0.980	-2.082

Table S152 Frequencies (cm-1) of α-ethyltetracene radical, calculated at the M06-2X/cc-pVDZ level of theory.

50	63	102	114	151	171	206	215
265	275	300	316	331	386	402	480
484	493	504	527	530	550	573	601
612	635	725	738	758	768	777	779
789	792	813	828	860	884	886	907
916	942	942	966	989	995	1009	1016
1028	1094	1099	1121	1140	1154	1173	1186
1194	1219	1252	1278	1283	1290	1321	1330
1353	1371	1388	1399	1431	1452	1455	1470
1475	1483	1484	1487	1502	1591	1616	1628
1647	1683	1690	1715	3051	3067	3086	3143
3150	3189	3190	3192	3196	3197	3199	3214
3216	3219	3228					

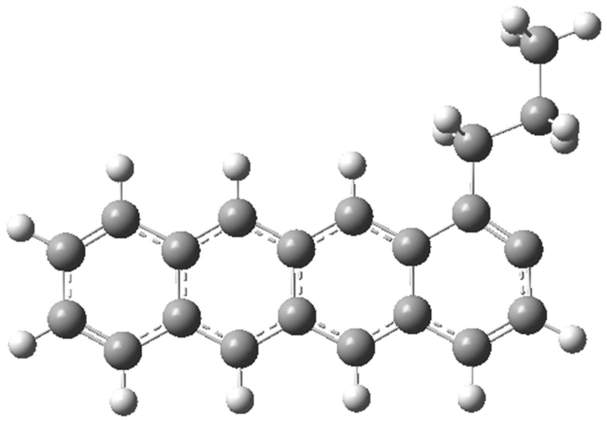

Figure S77 Visualization of the optimized structure of α-propyltetracene radical, calculated at the M06-2X/cc-pVDZ level of theory.

Table S153 Geometry (Å) of α-propyltetracene radical, calculated at the M06-2X/cc-pVDZ level of theory

Atom	x	y	z
**C**	-5.953	-3.064	-0.175
**C**	-4.786	-2.415	-0.357
**C**	-3.580	-3.196	-0.116
**C**	-3.712	-4.581	0.292
**C**	-5.023	-5.156	0.451
**C**	-6.142	-4.411	0.222
**C**	-2.311	-2.656	-0.266

**C**	-2.572	-5.337	0.524
**C**	-1.277	-4.796	0.375
**C**	-1.144	-3.419	-0.031
**C**	0.147	-2.874	-0.181
**H**	0.250	-1.832	-0.488
**C**	1.287	-3.633	0.052
**C**	1.152	-5.012	0.459
**C**	-0.116	-5.559	0.610
**H**	-2.192	-1.617	-0.571
**H**	-2.677	-6.378	0.831
**C**	2.345	-5.780	0.695
**H**	-5.100	-6.199	0.758
**H**	-7.139	-4.831	0.338
**H**	-0.218	-6.601	0.916
**H**	2.239	-6.821	1.001
**C**	3.577	-5.222	0.540
**C**	3.711	-3.851	0.136
**H**	4.707	-3.426	0.019
**C**	2.608	-3.087	-0.098
**H**	2.708	-2.046	-0.405
**H**	4.473	-5.814	0.722
**C**	-4.706	-0.971	-0.783
**H**	-4.135	-0.409	-0.027
**H**	-4.122	-0.907	-1.714
**C**	-6.067	-0.313	-0.988
**H**	-6.642	-0.382	-0.055
**H**	-6.628	-0.880	-1.742
**C**	-5.937	1.145	-1.418
**H**	-5.385	1.227	-2.363
**H**	-6.922	1.605	-1.562
**H**	-5.399	1.730	-0.660

Table S154 Frequencies (cm^{-1}) of α-propyltetracene radical, calculated at the M06-2X/cc-pVDZ level of theory.

42	60	80	86	98	154	174	180
231	245	245	294	314	318	331	388
402	481	486	493	503	526	546	550
573	611	617	636	731	748	750	758
768	776	780	792	805	831	859	870
884	890	910	910	919	943	944	987
996	1003	1015	1027	1075	1106	1119	1122
1143	1154	1174	1185	1196	1220	1244	1248
1272	1292	1308	1309	1323	1342	1356	1378
1392	1404	1431	1450	1456	1463	1470	1477
1484	1487	1488	1502	1591	1615	1628	1647
1682	1688	1716	3033	3056	3066	3067	3103
3131	3142	3194	3195	3196	3199	3201	3204

3218	3218	3221	3229	

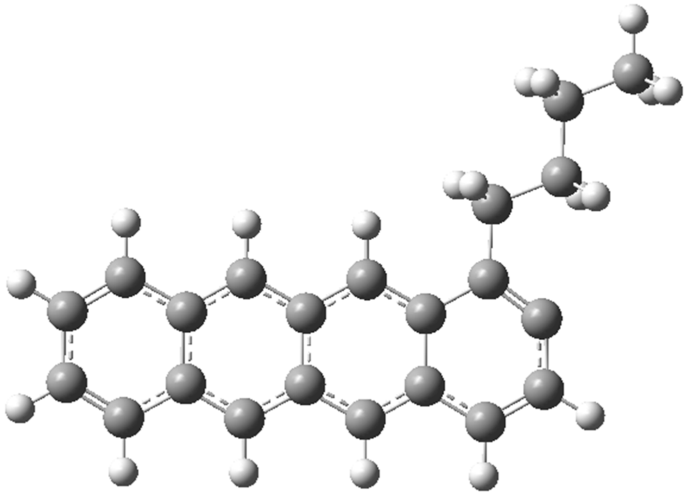

Figure S78 Visualization of the optimized structure of α-butyltetracene radical, calculated at the M06-2X/cc-pVDZ level of theory.

Table S155 Geometry (Å) of α-butyltetracene radical, calculated at the M06-2X/cc-pVDZ level of theory

Atom	x	y	z
C	-5.951	-3.080	-0.170
C	-4.784	-2.429	-0.353
C	-3.577	-3.209	-0.113
C	-3.707	-4.594	0.295
C	-5.016	-5.171	0.455
C	-6.137	-4.427	0.227
C	-2.309	-2.666	-0.263
C	-2.565	-5.348	0.527
C	-1.271	-4.804	0.377
C	-1.141	-3.427	-0.028
C	0.148	-2.879	-0.180
H	0.249	-1.837	-0.487
C	1.291	-3.635	0.053
C	1.160	-5.015	0.459
C	-0.107	-5.565	0.611
H	-2.193	-1.627	-0.568
H	-2.667	-6.389	0.834
C	2.354	-5.780	0.695
H	-5.092	-6.214	0.763
H	-7.134	-4.849	0.343
H	-0.207	-6.607	0.918
H	2.251	-6.821	1.002
C	3.585	-5.219	0.539
C	3.715	-3.848	0.135
H	4.710	-3.420	0.017
C	2.611	-3.086	-0.098

H	2.707	-2.045	-0.405
H	4.482	-5.808	0.721
C	-4.706	-0.985	-0.778
H	-4.135	-0.423	-0.023
H	-4.121	-0.921	-1.710
C	-6.065	-0.327	-0.984
H	-6.645	-0.392	-0.051
H	-6.631	-0.891	-1.740
C	-5.946	1.132	-1.414
H	-5.362	1.188	-2.344
H	-5.376	1.687	-0.654
C	-7.306	1.793	-1.620
H	-7.894	1.770	-0.693
H	-7.200	2.840	-1.928
H	-7.880	1.268	-2.395

Table S156 Frequencies (cm^{-1}) of α-butyltetracene radical, calculated at the M06-2X/cc-pVDZ level of theory.

32	57	57	64	88	131	151	154
181	205	228	248	277	294	316	331
342	402	436	483	495	498	505	525
544	550	572	611	615	636	721	741
745	760	770	776	780	787	794	810
835	863	885	888	908	913	917	930
945	947	988	991	997	1016	1030	1060
1094	1113	1123	1123	1145	1154	1175	1185
1198	1222	1226	1235	1265	1294	1295	1299
1311	1321	1333	1356	1366	1383	1395	1407
1432	1451	1456	1461	1465	1474	1477	1484
1486	1487	1503	1591	1615	1628	1648	1683
1692	1715	3034	3047	3055	3058	3066	3079
3103	3130	3141	3190	3192	3193	3196	3197
3200	3214	3216	3220	3228			

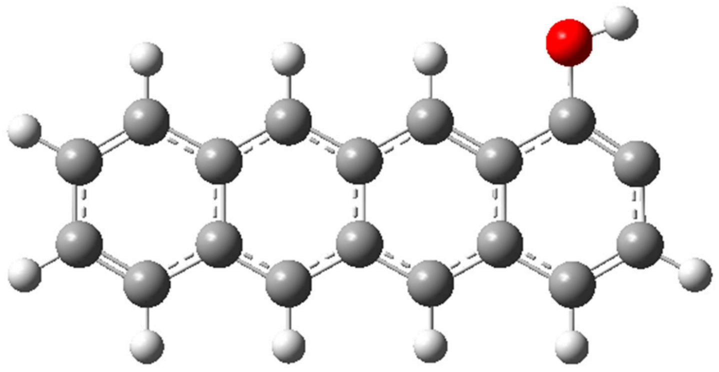

Figure S79 Visualization of the optimized structure of α-hydroxytetracene radical, calculated at the M06-2X/cc-pVDZ level of theory.

Table S157 Geometry (Å) of α-hydroxytetracene radical, calculated at the M06-2X/cc-pVDZ level of theory

Atom	x	y	z
C	5.160	-0.544	0.000
C	4.036	-1.313	0.000
C	2.730	-0.712	0.000
C	2.633	0.730	0.000
C	3.847	1.500	0.000
C	5.063	0.889	0.000
C	1.570	-1.475	0.000
C	1.381	1.331	0.000
C	0.197	0.565	0.000
C	0.294	-0.874	0.000
C	-0.892	-1.641	0.000
H	-0.817	-2.729	0.000
C	-2.147	-1.047	0.000
C	-1.080	1.169	0.000
H	1.643	-2.563	0.000
H	6.143	-1.011	0.000
H	4.106	-2.401	0.000
H	3.770	2.588	0.000
H	5.976	1.483	0.000
H	1.307	2.419	0.000
H	-1.161	2.254	0.000
C	-2.231	0.398	0.000
C	-3.545	1.004	0.000
C	-4.617	0.183	0.000
C	-4.577	-1.229	0.000
C	-3.352	-1.834	0.000
H	-5.496	-1.813	0.000
H	-3.269	-2.920	0.000
O	-3.592	2.365	0.000
H	-4.516	2.640	0.000

Table S158 Frequencies (cm^{-1}) of α-hydroxytetracene radical, calculated at the M06-2X/cc-pVDZ level of theory.

54	74	143	151	173	230	252	298
314	329	395	400	419	476	482	496
504	520	524	563	587	606	615	634
731	738	759	763	775	776	792	811
838	861	881	888	912	917	943	951
974	995	1012	1027	1034	1121	1132	1152
1172	1182	1203	1216	1239	1274	1288	1318
1324	1348	1370	1411	1437	1451	1462	1482
1488	1502	1589	1611	1626	1643	1681	1695

1715	3193	3195	3196	3198	3201	3205	3216
3223	3224	3228	3868				

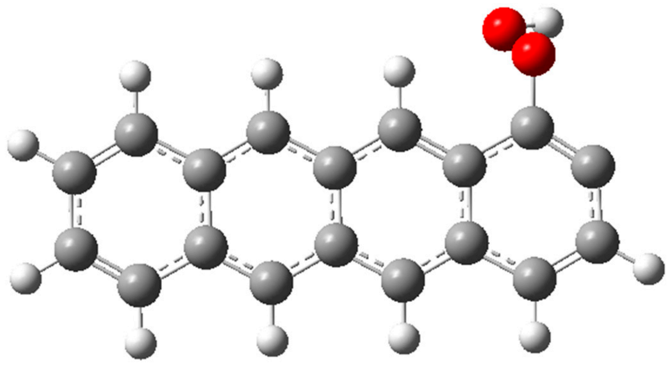

Figure S80 Visualization of the optimized structure of α-peroxytetracene radical, calculated at the M06-2X/cc-pVDZ level of theory.

Table S159 Geometry (Å) of α-peroxytetracene radical, calculated at the M06-2X/cc-pVDZ level of theory

Atom	x	y	z
C	5.537	0.306	0.041
C	4.473	1.156	0.028
C	3.127	0.652	0.016
C	2.924	-0.779	0.017
C	4.078	-1.637	0.030
C	5.336	-1.115	0.041
C	2.026	1.498	0.004
C	1.631	-1.287	0.005
C	0.506	-0.437	-0.007
C	0.709	0.992	-0.007
C	-0.416	1.843	-0.014
H	-0.263	2.923	-0.008
C	-1.709	1.337	-0.023
C	-0.810	-0.948	-0.022
H	2.179	2.578	0.004
H	6.552	0.699	0.050
H	4.623	2.236	0.028
H	3.921	-2.715	0.031
H	6.202	-1.776	0.051
H	1.478	-2.366	0.005
H	-0.970	-2.025	-0.029
C	-1.901	-0.096	-0.032
C	-3.262	-0.589	-0.039
C	-4.272	0.302	-0.033
C	-4.127	1.710	-0.006
C	-2.855	2.210	-0.009
H	-4.996	2.365	0.011

**H**	-2.688	3.287	0.004
**O**	-3.507	-1.946	0.028
**O**	-3.094	-2.536	-1.213
**H**	-3.930	-2.549	-1.701

Table S160 Frequencies (cm^{-1}) of α-peroxytetracene radical, calculated at the M06-2X/cc-pVDZ level of theory.

58	65	89	132	152	158	204	239
268	287	313	329	352	404	428	472
482	496	507	522	540	565	584	606
634	661	731	756	761	769	776	776
792	814	846	862	884	888	913	918
944	951	960	989	996	1014	1029	1042
1124	1137	1155	1175	1190	1200	1221	1261
1293	1299	1324	1345	1359	1384	1406	1432
1453	1460	1482	1489	1504	1590	1613	1629
1641	1680	1693	1716	3193	3196	3197	3198
3202	3203	3217	3224	3226	3228	3807	

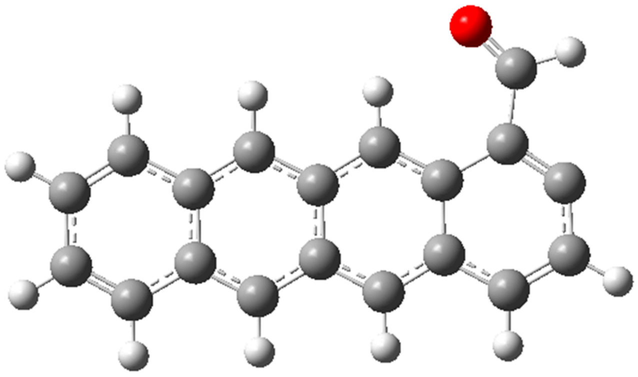

Figure S81 Visualization of the optimized structure of α-tetraldehyde radical, calculated at the M06-2X/cc-pVDZ level of theory.

Table S161 Geometry (Å) of α-tetraldehyde radical, calculated at the M06-2X/cc-pVDZ level of theory

Atom	x	y	z
**C**	5.340	-0.395	0.000
**C**	4.265	-1.231	0.000
**C**	2.926	-0.708	0.000
**C**	2.742	0.724	0.000
**C**	3.906	1.567	0.000
**C**	5.157	1.028	0.000
**C**	1.812	-1.539	0.000
**C**	1.455	1.251	0.000
**C**	0.320	0.417	0.000
**C**	0.505	-1.011	0.000
**C**	-0.637	-1.840	0.000

**H**	-0.507	-2.923	0.001
**C**	-1.919	-1.309	0.000
**C**	-0.990	0.955	0.000
**H**	1.949	-2.621	0.001
**H**	6.350	-0.802	0.000
**H**	4.400	-2.312	0.001
**H**	3.765	2.647	0.000
**H**	6.032	1.677	0.000
**H**	1.316	2.333	0.000
**H**	-1.120	2.034	0.000
**C**	-2.104	0.130	0.000
**C**	-3.474	0.631	0.000
**C**	-4.466	-0.293	0.000
**C**	-4.339	-1.697	0.000
**C**	-3.062	-2.184	0.000
**H**	-5.207	-2.353	0.000
**H**	-2.885	-3.261	0.000
**C**	-3.870	2.058	-0.001
**O**	-3.118	3.007	-0.001
**H**	-4.971	2.209	-0.001

Table S162 Frequencies (cm^{-1}) of α-tetraldehyde radical, calculated at the M06-2X/cc-pVDZ level of theory.

57	60	114	127	162	173	212	252
290	312	320	341	402	430	449	482
496	507	532	549	575	580	608	634
650	720	746	761	771	777	778	793
811	851	863	884	895	918	925	944
970	997	1000	1014	1028	1030	1047	1122
1137	1155	1168	1178	1200	1222	1265	1295
1306	1325	1352	1356	1385	1408	1434	1455
1459	1480	1485	1500	1586	1606	1629	1634
1670	1688	1714	1808	2980	3194	3194	3196
3199	3200	3203	3217	3227	3228	3255	

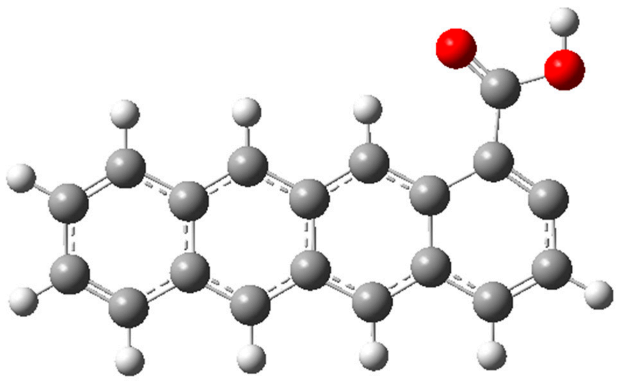

Figure S82 Visualization of the optimized structure of α-tetracenic acid radical, calculated at the M06-2X/cc-pVDZ level of theory.

Table S163 Geometry (Å) of α-tetracenic acid radical, calculated at the M06-2X/cc-pVDZ level of theory

Atom	x	y	z
C	-5.654	0.135	0.000
C	-4.640	1.044	0.000
C	-3.267	0.616	0.000
C	-2.984	-0.801	0.000
C	-4.087	-1.722	0.000
C	-5.373	-1.272	0.000
C	-2.214	1.522	0.000
C	-1.664	-1.237	0.000
C	-0.589	-0.326	0.000
C	-0.874	1.086	0.000
C	0.209	1.989	0.000
H	0.006	3.061	0.000
C	1.525	1.548	0.000
C	0.753	-0.773	0.000
H	-2.426	2.592	0.000
H	-6.690	0.471	0.000
H	-4.850	2.113	0.000
H	-3.870	-2.790	0.000
H	-6.200	-1.980	0.000
H	-1.451	-2.306	0.000
H	0.952	-1.840	0.000
C	1.812	0.125	0.000
C	3.217	-0.268	0.000
C	4.140	0.721	0.000
C	3.908	2.111	0.000
C	2.600	2.505	0.000
H	4.727	2.827	0.000
H	2.343	3.565	0.000
C	3.673	-1.683	0.000
O	2.971	-2.667	0.000
O	5.022	-1.776	-0.001
H	5.228	-2.722	-0.001

Table S164 Frequencies (cm^{-1}) of α-tetracenic acid radical, calculated at the M06-2X/cc-pVDZ level of theory.

43	54	67	115	137	162	212	218
286	289	316	333	359	396	437	481
493	506	513	524	532	570	586	612
615	634	640	729	738	759	769	777
780	791	795	831	833	862	883	892
917	920	941	965	982	996	1004	1017
1027	1115	1140	1154	1155	1174	1190	1203

1220	1263	1292	1296	1322	1330	1350	1378
1413	1433	1454	1455	1481	1484	1500	1588
1610	1629	1638	1675	1687	1717	1832	3191
3192	3197	3198	3200	3201	3218	3230	3231
3256	3821						

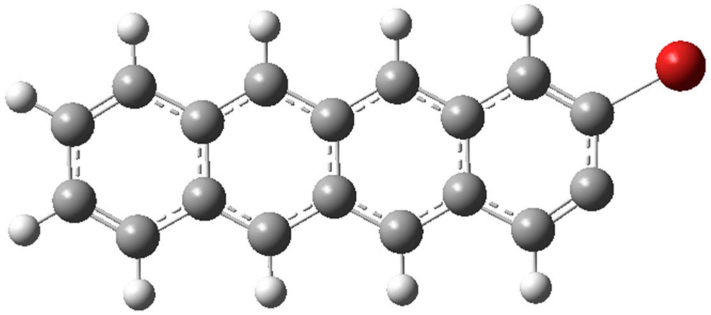

Figure S83 Visualization of the optimized structure of β-bromotetracene radical, calculated at the M06-2X/cc-pVDZ level of theory.

Table S165 Geometry (Å) of β-bromotetracene radical, calculated at the M06-2X/cc-pVDZ level of theory

Atom	x	y	z
C	-3.650	0.723	0.000
C	-2.476	1.413	0.000
C	-1.215	0.723	0.000
C	-1.218	-0.721	0.000
C	-2.482	-1.407	0.000
C	-3.653	-0.712	0.000
C	-0.005	1.405	0.000
C	-0.010	-1.407	0.000
C	1.224	-0.724	0.000
C	1.226	0.719	0.000
C	2.463	1.401	0.000
H	2.466	2.492	0.000
C	3.667	0.715	0.000
C	2.456	-1.412	0.000
H	-0.004	2.496	0.000
H	-4.599	1.257	0.000
H	-2.472	2.503	0.000
H	-2.481	-2.497	0.000
H	-4.604	-1.243	0.000
H	-0.013	-2.498	0.000
H	2.453	-2.503	0.000
C	3.667	-0.733	0.000
C	4.918	-1.446	0.000
C	6.091	-0.755	0.000
H	4.912	-2.535	-0.001
C	6.056	0.657	0.000

**C**	4.939	1.404	0.000
**H**	4.963	2.494	0.000
**Br**	7.771	-1.631	-0.001

Table S166 Frequencies (cm^{-1}) of β-bromotetracene radical, calculated at the M06-2X/cc-pVDZ level of theory.

39	80	102	116	180	194	223	239
297	334	349	379	385	461	470	484
499	514	544	576	590	628	638	653
723	760	769	769	777	788	796	844
859	876	878	894	907	922	922	947
950	997	1017	1030	1083	1123	1156	1176
1185	1210	1222	1260	1280	1294	1320	1322
1347	1384	1410	1451	1456	1478	1489	1505
1590	1613	1628	1638	1669	1690	1715	3194
3196	3197	3198	3198	3203	3204	3215	3216
3228							

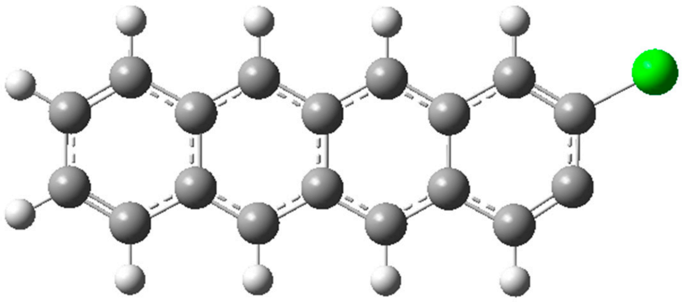

Figure S84 Visualization of the optimized structure of β-chlorotetracene acid radical, calculated at the M06-2X/cc-pVDZ level of theory.

Table S167 Geometry (Å) of β-chlorotetracene radical, calculated at the M06-2X/cc-pVDZ level of theory

Atom	x	y	z
**C**	-3.650	0.723	0.000
**C**	-2.477	1.413	0.000
**C**	-1.215	0.723	0.000
**C**	-1.218	-0.721	0.000
**C**	-2.481	-1.407	0.000
**C**	-3.653	-0.713	0.000
**C**	-0.005	1.405	0.000
**C**	-0.010	-1.407	0.000
**C**	1.224	-0.724	0.000
**C**	1.226	0.719	0.000
**C**	2.462	1.402	0.000
**H**	2.466	2.492	0.000
**C**	3.666	0.715	0.000

C	2.457	-1.412	0.000
H	-0.004	2.496	0.000
H	-4.600	1.256	0.000
H	-2.472	2.503	0.000
H	-2.480	-2.497	0.000
H	-4.604	-1.243	0.000
H	-0.012	-2.498	0.000
H	2.453	-2.502	0.000
C	3.667	-0.733	0.000
C	4.919	-1.444	0.000
C	6.093	-0.755	0.000
H	4.919	-2.534	-0.001
C	6.057	0.660	0.000
C	4.939	1.405	0.000
H	4.961	2.494	0.000
Cl	7.634	-1.565	0.000

Table S168 Frequencies (cm^{-1}) of β-chlorotetracene radical, calculated at the M06-2X/cc-pVDZ level of theory.

44	81	121	121	181	222	228	278
299	343	353	386	420	462	471	484
499	519	545	582	600	628	638	679
722	761	769	769	777	789	803	845
860	876	881	902	907	921	926	946
950	998	1017	1029	1098	1122	1154	1174
1182	1207	1222	1257	1279	1292	1319	1322
1346	1384	1412	1452	1454	1478	1488	1504
1591	1616	1627	1639	1673	1689	1715	3194
3196	3197	3199	3199	3202	3203	3215	3217
3229							

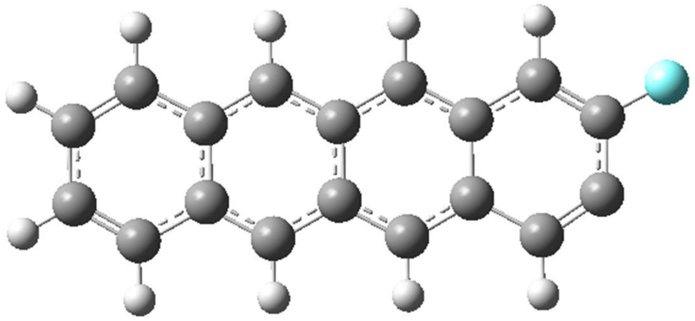

Figure S85 Visualization of the optimized structure of β-fluorotetracene radical, calculated at the M06-2X/cc-pVDZ level of theory.

Table S169 Geometry (Å) of β-fluorotetracene radical, calculated at the M06-2X/cc-pVDZ level of theory

Atom	x	y	z

**C**	-3.651	0.723	0.000
**C**	-2.477	1.414	0.000
**C**	-1.216	0.724	0.000
**C**	-1.218	-0.721	0.000
**C**	-2.482	-1.407	0.000
**C**	-3.653	-0.712	0.000
**C**	-0.005	1.405	0.000
**C**	-0.010	-1.407	0.000
**C**	1.224	-0.725	0.000
**C**	1.226	0.718	0.000
**C**	2.462	1.400	0.000
**H**	2.466	2.490	0.000
**C**	3.666	0.713	0.000
**C**	2.457	-1.413	0.000
**H**	-0.003	2.496	0.000
**H**	-4.600	1.257	0.000
**H**	-2.472	2.504	0.000
**H**	-2.481	-2.496	0.000
**H**	-4.604	-1.243	0.000
**H**	-0.013	-2.498	0.000
**H**	2.453	-2.503	0.000
**C**	3.668	-0.735	0.000
**C**	4.922	-1.444	0.000
**C**	6.085	-0.746	0.000
**H**	4.938	-2.533	-0.001
**C**	6.058	0.669	0.000
**C**	4.937	1.407	0.000
**H**	4.954	2.497	0.000
**F**	7.274	-1.373	0.000

Table S170 Frequencies (cm^{-1}) of β-fluorotetracene radical, calculated at the M06-2X/cc-pVDZ level of theory.

46	84	131	142	185	242	264	300
307	368	386	392	466	472	480	484
498	531	548	592	622	629	639	717
740	759	769	770	777	791	828	844
858	871	881	905	915	919	948	948
959	995	1012	1027	1120	1152	1167	1172
1192	1204	1244	1261	1278	1291	1320	1334
1346	1385	1429	1450	1466	1481	1487	1503
1594	1626	1638	1644	1687	1694	1716	3193
3195	3196	3198	3198	3202	3202	3217	3218
3229							

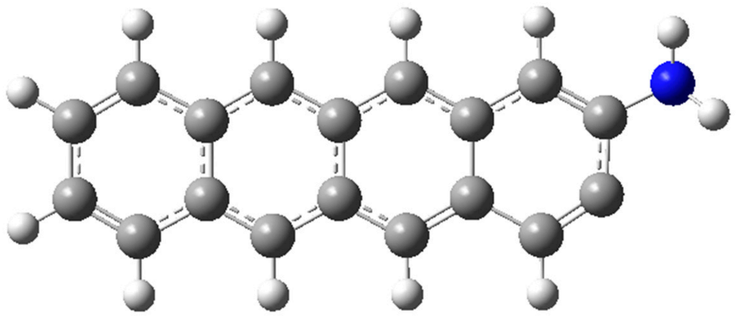

Figure S86 Visualization of the optimized structure of β-aminotetracene radical, calculated at the M06-2X/cc-pVDZ level of theory.

Table S171 Geometry (Å) of β-aminotetracene radical, calculated at the M06-2X/cc-pVDZ level of theory

Atom	x	y	z
C	-3.659	0.731	0.024
C	-2.483	1.418	0.021
C	-1.223	0.726	0.008
C	-1.227	-0.718	-0.001
C	-2.492	-1.401	0.003
C	-3.663	-0.704	0.015
C	-0.010	1.405	0.004
C	-0.019	-1.406	-0.014
C	1.217	-0.727	-0.018
C	1.218	0.715	-0.009
C	2.458	1.394	-0.012
H	2.465	2.485	-0.005
C	3.658	0.703	-0.024
C	2.449	-1.417	-0.031
H	-0.006	2.496	0.011
H	-4.607	1.266	0.034
H	-2.475	2.508	0.028
H	-2.493	-2.490	-0.004
H	-4.614	-1.234	0.018
H	-0.025	-2.497	-0.021
H	2.440	-2.508	-0.037
C	3.665	-0.745	-0.034
C	4.917	-1.450	-0.049
C	6.116	-0.784	-0.048
H	4.901	-2.541	-0.056
C	6.047	0.642	-0.039
C	4.934	1.388	-0.030
H	4.960	2.478	-0.025
N	7.357	-1.407	0.007
H	7.373	-2.369	-0.303
H	8.124	-0.860	-0.360

Table S172 Frequencies (cm^{-1}) of β-aminotetracene radical, calculated at the M06-2X/cc-pVDZ level of theory.

48	84	129	142	183	236	260	295
302	322	361	372	389	466	467	474
483	496	526	538	553	595	630	639
645	717	748	759	768	771	777	789
830	842	846	863	880	898	913	916
940	947	948	995	1014	1027	1098	1127
1152	1171	1179	1204	1206	1258	1274	1280
1298	1321	1344	1350	1389	1439	1450	1480
1484	1488	1505	1592	1616	1626	1636	1648
1686	1695	1715	3186	3191	3192	3193	3194
3196	3198	3200	3216	3228	3587	3702	

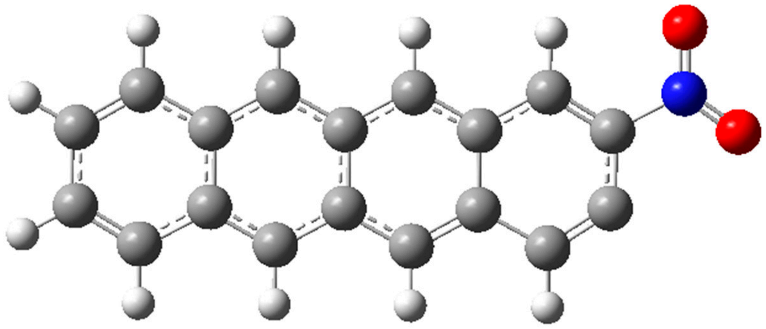

Figure S87 Visualization of the optimized structure of β-nitrotetracene radical, calculated at the M06-2X/cc-pVDZ level of theory.

Table S173 Geometry (Å) of β-nitrotetracene radical, calculated at the M06-2X/cc-pVDZ level of theory

Atom	x	y	z
C	-3.646	0.726	0.000
C	-2.471	1.415	0.000
C	-1.212	0.722	0.000
C	-1.219	-0.722	0.000
C	-2.483	-1.406	0.000
C	-3.653	-0.709	0.000
C	0.000	1.404	0.000
C	-0.012	-1.410	0.000
C	1.221	-0.728	0.000
C	1.229	0.715	0.000
C	2.467	1.398	0.000
H	2.470	2.488	0.000
C	3.670	0.711	0.000
C	2.451	-1.418	0.000
H	0.002	2.494	0.000
H	-4.594	1.262	0.000
H	-2.465	2.505	0.000

**H**	-2.484	-2.496	-0.001
**H**	-4.605	-1.237	0.000
**H**	-0.016	-2.501	0.000
**H**	2.450	-2.508	0.000
**C**	3.662	-0.738	0.000
**C**	4.907	-1.453	0.000
**C**	6.075	-0.756	0.000
**H**	4.924	-2.542	0.000
**C**	6.063	0.658	0.000
**C**	4.945	1.401	0.000
**H**	4.965	2.490	0.000
**N**	7.361	-1.460	-0.001
**O**	7.347	-2.676	0.000
**O**	8.361	-0.769	-0.001

Table S174 Frequencies (cm^{-1}) of β-nitrotetracene radical, calculated at the M06-2X/cc-pVDZ level of theory.

40	58	82	109	122	180	202	224
258	295	320	344	388	406	446	472
485	500	518	536	556	576	579	628
638	667	723	762	770	770	777	787
790	799	847	856	865	884	890	914
916	938	951	957	968	1000	1020	1030
1123	1131	1156	1177	1184	1209	1229	1261
1282	1295	1322	1330	1351	1386	1430	1454
1458	1466	1482	1488	1507	1596	1628	1628
1646	1672	1687	1707	1715	3197	3199	3200
3201	3202	3205	3206	3218	3228	3230	

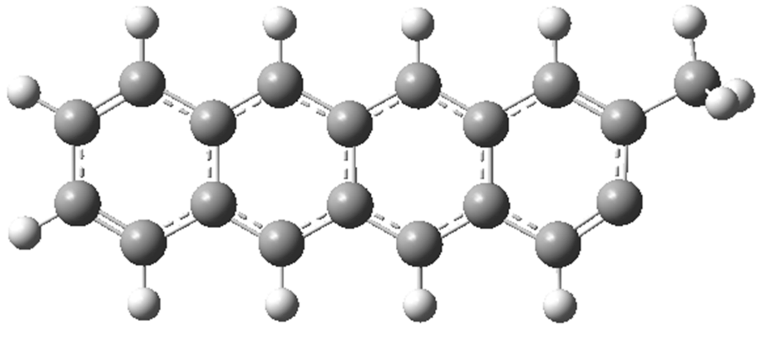

Figure S88 Visualization of the optimized structure of β-methyltetracene radical, calculated at the M06-2X/cc-pVDZ level of theory.

Table S175 Geometry (Å) of β-methyltetracene radical, calculated at the M06-2X/cc-pVDZ level of theory

Atom	x	y	z
**C**	-3.659	0.727	0.000

**C**	-2.484	1.416	0.034
**C**	-1.223	0.725	0.016
**C**	-1.227	-0.718	-0.038
**C**	-2.491	-1.402	-0.072
**C**	-3.662	-0.708	-0.053
**C**	-0.012	1.406	0.049
**C**	-0.019	-1.404	-0.056
**C**	1.215	-0.723	-0.022
**C**	1.219	0.719	0.032
**C**	2.456	1.399	0.065
**H**	2.461	2.489	0.106
**C**	3.659	0.710	0.047
**C**	2.448	-1.411	-0.040
**H**	-0.011	2.496	0.090
**H**	-4.608	1.261	0.014
**H**	-2.479	2.505	0.075
**H**	-2.490	-2.491	-0.112
**H**	-4.614	-1.237	-0.079
**H**	-0.022	-2.494	-0.096
**H**	2.443	-2.501	-0.081
**C**	3.660	-0.735	-0.007
**C**	4.916	-1.442	-0.025
**C**	6.117	-0.791	0.007
**H**	4.891	-2.533	-0.066
**C**	6.045	0.632	0.060
**C**	4.937	1.390	0.081
**H**	4.966	2.479	0.122
**C**	7.445	-1.493	-0.011
**H**	8.024	-1.244	0.887
**H**	7.311	-2.579	-0.053
**H**	8.036	-1.177	-0.880

Table S176 Frequencies (cm^{-1}) of β-methyltetracene radical, calculated at the M06-2X/cc-pVDZ level of theory.

48	84	122	141	151	182	235	250
299	303	353	355	386	463	470	473
483	496	534	545	592	593	630	640
729	734	759	771	771	778	789	822
839	857	873	881	902	912	918	937
944	948	996	1015	1017	1028	1053	1122
1152	1172	1181	1191	1206	1232	1265	1278
1292	1322	1330	1345	1384	1396	1419	1450
1459	1461	1472	1485	1487	1505	1592	1623
1626	1642	1686	1693	1715	3064	3132	3160
3185	3192	3193	3194	3195	3197	3198	3201
3216	3228						

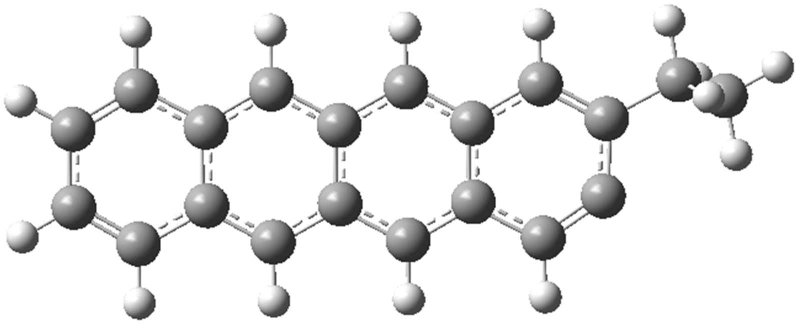

Figure S89 Visualization of the optimized structure of β-ethyltetracene radical, calculated at the M06-2X/cc-pVDZ level of theory.

Table S177 Geometry (Å) of β-ethyltetracene radical, calculated at the M06-2X/cc-pVDZ level of theory

Atom	x	y	z
C	-6.071	-3.124	-0.402
C	-4.830	-2.552	-0.458
C	-3.627	-3.308	-0.215
C	-3.724	-4.717	0.100
C	-5.043	-5.311	0.160
C	-6.095	-4.513	-0.083
C	-2.375	-2.713	-0.276
C	-2.573	-5.451	0.334
C	-1.294	-4.854	0.274
C	-1.193	-3.449	-0.039
C	0.082	-2.851	-0.100
H	0.159	-1.789	-0.336
C	1.239	-3.584	0.134
C	1.138	-4.990	0.447
C	-0.114	-5.589	0.509
H	-2.296	-1.651	-0.512
H	-2.651	-6.513	0.571
C	2.348	-5.729	0.686
H	-5.145	-6.369	0.398
H	-0.189	-6.651	0.746
H	2.269	-6.791	0.922
C	3.564	-5.120	0.620
C	3.664	-3.722	0.309
H	4.648	-3.257	0.262
C	2.544	-2.984	0.076
H	2.617	-1.922	-0.161
H	4.473	-5.691	0.803
C	-7.352	-2.371	-0.644
H	-7.113	-1.320	-0.851
H	-7.838	-2.778	-1.541
C	-8.313	-2.474	0.543
H	-9.239	-1.922	0.344

**H**	-8.572	-3.523	0.737
**H**	-7.851	-2.065	1.450
**H**	-4.732	-1.491	-0.695

Table S178 Frequencies (cm^{-1}) of β-ethyltetracene radical, calculated at the M06-2X/cc-pVDZ level of theory.

35	53	78	113	135	169	200	210
259	266	300	322	360	380	403	462
471	483	487	495	533	552	590	629
637	642	726	731	758	770	770	777
782	790	815	837	858	874	881	903
909	919	932	945	949	989	995	1014
1028	1077	1092	1123	1152	1172	1181	1190
1206	1230	1254	1273	1278	1291	1320	1330
1339	1350	1384	1387	1423	1451	1461	1466
1472	1477	1485	1488	1504	1591	1622	1627
1643	1686	1692	1715	3063	3066	3115	3144
3149	3183	3192	3193	3194	3195	3196	3197
3201	3216	3228					

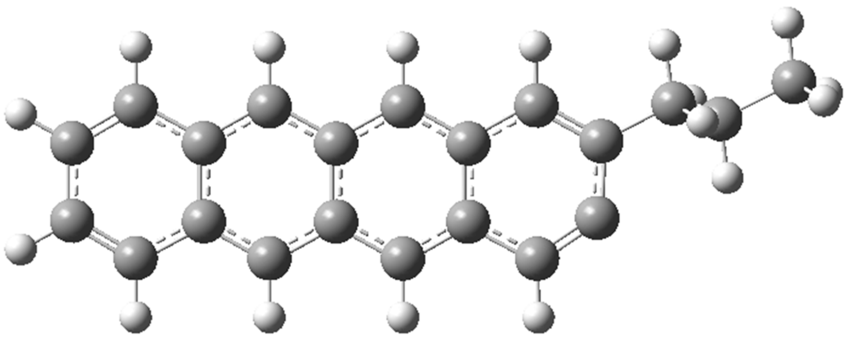

Figure S90 Visualization of the optimized structure of β-propyltetracene radical, calculated at the M06-2X/cc-pVDZ level of theory.

Table S179 Geometry (Å) of β-propyltetracene radical, calculated at the M06-2X/cc-pVDZ level of theory

Atom	x	y	z
**C**	-5.855	-2.609	-0.193
**C**	-4.579	-2.201	-0.467
**C**	-3.432	-3.015	-0.156
**C**	-3.624	-4.306	0.468
**C**	-4.979	-4.727	0.756
**C**	-5.974	-3.888	0.423
**C**	-2.142	-2.587	-0.440
**C**	-2.527	-5.097	0.770
**C**	-1.211	-4.669	0.482
**C**	-1.015	-3.382	-0.138
**C**	0.298	-2.953	-0.425

**H**	0.446	-1.979	-0.893
**C**	1.401	-3.742	-0.123
**C**	1.204	-5.031	0.497
**C**	-0.085	-5.462	0.784
**H**	-1.991	-1.615	-0.910
**H**	-2.677	-6.070	1.239
**C**	2.360	-5.831	0.800
**H**	-5.153	-5.694	1.228
**H**	-0.233	-6.436	1.252
**H**	2.208	-6.804	1.268
**C**	3.614	-5.387	0.510
**C**	3.809	-4.105	-0.107
**H**	4.821	-3.770	-0.329
**C**	2.743	-3.314	-0.411
**H**	2.888	-2.341	-0.879
**H**	4.481	-6.002	0.744
**H**	-4.409	-1.231	-0.940
**C**	-7.078	-1.785	-0.489
**H**	-6.776	-0.860	-0.999
**H**	-7.729	-2.341	-1.180
**C**	-7.873	-1.447	0.776
**H**	-7.222	-0.893	1.467
**H**	-8.146	-2.383	1.283
**C**	-9.126	-0.635	0.466
**H**	-9.683	-0.398	1.380
**H**	-8.866	0.311	-0.027
**H**	-9.793	-1.192	-0.204

Table S180 Frequencies (cm^{-1}) of β-propyltetracene radical, calculated at the M06-2X/cc-pVDZ level of theory.

26	46	72	77	102	143	159	192
234	244	253	277	308	349	358	378
396	465	471	483	495	500	543	555
594	630	639	645	727	735	755	759
770	772	778	790	820	836	856	866
878	882	899	907	917	922	946	951
952	995	1014	1029	1072	1094	1118	1126
1153	1173	1184	1190	1208	1230	1241	1265
1280	1287	1295	1308	1323	1332	1348	1374
1388	1395	1424	1452	1462	1463	1469	1476
1481	1485	1488	1505	1592	1622	1628	1643
1686	1692	1715	3050	3058	3065	3093	3113
3133	3143	3183	3193	3194	3195	3197	3197
3198	3203	3217	3229				

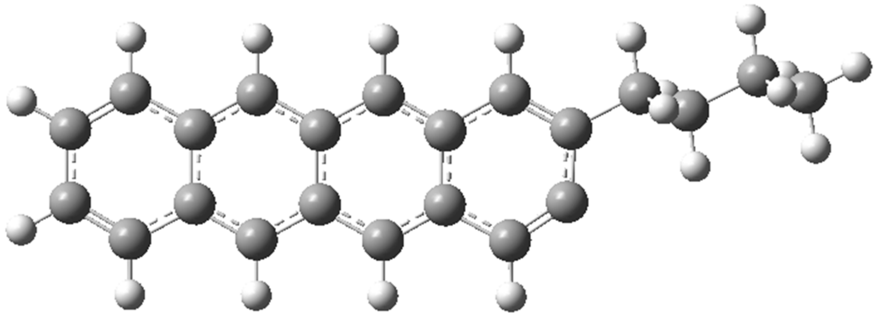

Figure S91 Visualization of the optimized structure of β-butyltetracene radical, calculated at the M06-2X/cc-pVDZ level of theory.

Table S181 Geometry (Å) of β-butyltetracene radical, calculated at the M06-2X/cc-pVDZ level of theory

Atom	x	y	z
C	-5.737	-2.441	-0.195
C	-4.457	-2.118	-0.549
C	-3.332	-2.944	-0.189
C	-3.553	-4.155	0.571
C	-4.912	-4.484	0.944
C	-5.885	-3.643	0.556
C	-2.038	-2.604	-0.557
C	-2.477	-4.956	0.920
C	-1.157	-4.618	0.549
C	-0.932	-3.411	-0.208
C	0.385	-3.071	-0.579
H	0.556	-2.158	-1.152
C	1.466	-3.872	-0.233
C	1.241	-5.080	0.525
C	-0.053	-5.424	0.895
H	-1.865	-1.692	-1.130
H	-2.650	-5.868	1.493
C	2.374	-5.895	0.873
H	-5.108	-5.389	1.520
H	-0.223	-6.337	1.468
H	2.201	-6.806	1.444
C	3.633	-5.537	0.498
C	3.857	-4.335	-0.254
H	4.873	-4.070	-0.543
C	2.813	-3.534	-0.606
H	2.980	-2.621	-1.177
H	4.483	-6.162	0.768
H	-4.265	-1.211	-1.126
C	-6.936	-1.600	-0.538
H	-6.616	-0.748	-1.152
H	-7.633	-2.197	-1.146
C	-7.671	-1.097	0.708

**H**	-6.977	-0.502	1.318
**H**	-7.967	-1.961	1.322
**C**	-8.902	-0.265	0.363
**H**	-8.598	0.587	-0.262
**H**	-9.586	-0.873	-0.247
**C**	-9.631	0.241	1.605
**H**	-8.969	0.870	2.214
**H**	-10.512	0.836	1.338
**H**	-9.964	-0.598	2.229

Table S182 Frequencies (cm^{-1}) of β-butyltetracene radical, calculated at the M06-2X/cc-pVDZ level of theory.

18	37	64	69	95	114	141	148
186	219	234	240	261	297	330	357
363	385	428	469	472	483	496	498
544	554	595	630	638	646	722	728
754	759	770	772	778	784	790	826
837	858	873	882	901	911	912	918
928	947	948	954	996	1014	1029	1047
1090	1098	1126	1128	1153	1173	1184	1191
1208	1220	1234	1252	1267	1280	1292	1297
1311	1321	1332	1342	1352	1384	1391	1396
1425	1452	1460	1464	1466	1473	1475	1482
1486	1488	1505	1592	1622	1628	1643	1686
1692	1715	3046	3052	3058	3060	3078	3096
3115	3133	3143	3183	3192	3193	3194	3196
3197	3197	3201	3216	3228			

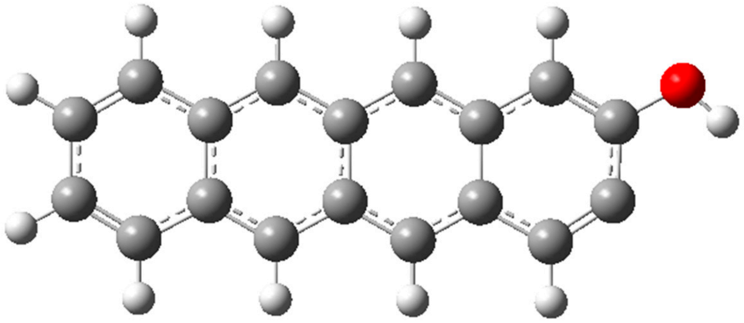

Figure S92 Visualization of the optimized structure of β-hydroxytetracene radical, calculated at the M06-2X/cc-pVDZ level of theory.

Table S183 Geometry (Å) of β-hydroxytetracene radical, calculated at the M06-2X/cc-pVDZ level of theory

Atom	x	y	z
**C**	-3.940	-0.949	0.000
**C**	-2.675	-1.521	0.000
**C**	-1.507	-0.729	0.000

**C**	-1.640	0.708	0.000
**C**	-2.929	1.279	0.000
**C**	-0.217	-1.301	0.000
**C**	-0.471	1.500	0.000
**C**	0.790	0.924	0.000
**C**	0.930	-0.517	0.000
**C**	2.241	-1.107	0.000
**H**	2.350	-2.191	0.000
**C**	3.357	-0.322	0.000
**C**	3.171	1.088	0.000
**C**	1.993	1.729	0.000
**H**	-0.122	-2.387	0.000
**H**	-2.578	-2.607	0.000
**H**	-3.027	2.365	0.000
**H**	-0.567	2.586	0.000
**H**	1.916	2.816	0.000
**O**	4.600	-0.877	0.000
**H**	5.257	-0.172	0.000
**C**	-4.072	0.490	0.000
**C**	-5.391	1.061	-0.001
**C**	-5.136	-1.748	-0.001
**C**	-6.497	0.266	-0.001
**H**	-5.486	2.146	-0.001
**C**	-6.367	-1.164	-0.001
**H**	-5.035	-2.833	-0.001
**H**	-7.491	0.710	-0.001
**H**	-7.265	-1.780	-0.001

Table S184 Frequencies (cm^{-1}) of β-hydroxytetracene radical, calculated at the M06-2X/cc-pVDZ level of theory.

47	85	132	142	185	241	264	302
305	342	367	381	392	468	470	479
483	498	533	550	594	630	633	639
717	745	758	770	770	777	791	830
844	856	869	880	904	915	918	946
947	955	994	1011	1026	1118	1152	1171
1172	1194	1201	1208	1260	1272	1283	1303
1321	1341	1349	1388	1440	1450	1472	1485
1487	1504	1594	1626	1641	1644	1687	1695
1716	3192	3194	3195	3197	3197	3201	3201
3212	3216	3228	3875				

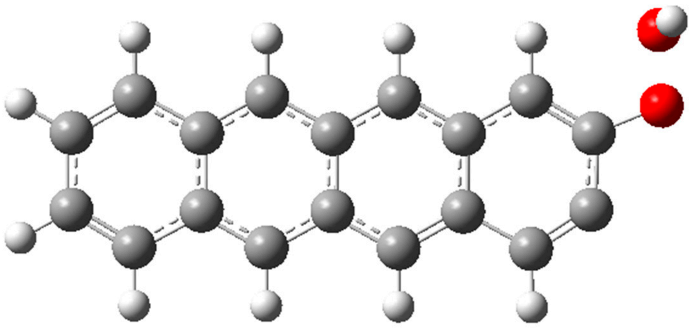

Figure S93 Visualization of the optimized structure of α-peroxytetracene radical, calculated at the M06-2X/cc-pVDZ level of theory.

Table S185 Geometry (Å) of β-peroxytetracene radical, calculated at the M06-2X/cc-pVDZ level of theory

Atom	x	y	z
C	-3.662	0.748	-0.002
C	-2.483	1.430	-0.005
C	-1.227	0.731	-0.002
C	-1.238	-0.713	0.003
C	-2.507	-1.390	0.006
C	-3.674	-0.687	0.004
C	-0.011	1.404	-0.005
C	-0.035	-1.407	0.006
C	1.204	-0.734	0.003
C	1.215	0.707	-0.002
C	2.457	1.380	-0.004
H	2.470	2.471	-0.007
C	3.654	0.683	-0.002
C	2.433	-1.431	0.005
H	-0.001	2.494	-0.009
H	-4.607	1.288	-0.003
H	-2.470	2.519	-0.009
H	-2.513	-2.480	0.011
H	-4.628	-1.212	0.006
H	-0.045	-2.498	0.010
H	2.420	-2.522	0.009
C	3.650	-0.765	0.002
C	4.896	-1.489	0.003
C	6.074	-0.806	0.000
H	4.887	-2.576	0.017
C	6.044	0.616	0.001
C	4.932	1.365	-0.001
H	4.962	2.454	-0.002
O	7.337	-1.342	0.001
O	7.273	-2.752	0.005
H	7.555	-2.959	-0.898

Table S186 Frequencies (cm^{-1}) of β-peroxytetracene radical, calculated at the M06-2X/cc-pVDZ level of theory.

46	71	115	121	135	185	192	208
244	293	309	333	368	393	433	465
471	484	499	519	550	571	597	629
630	639	719	741	760	769	770	778
789	838	846	855	868	883	902	916
917	945	948	971	996	1016	1030	1048
1126	1154	1175	1176	1196	1208	1243	1263
1281	1294	1321	1332	1351	1387	1427	1434
1453	1468	1485	1489	1508	1596	1628	1639
1649	1688	1692	1715	3193	3195	3196	3198
3198	3201	3202	3216	3227	3245	3809	

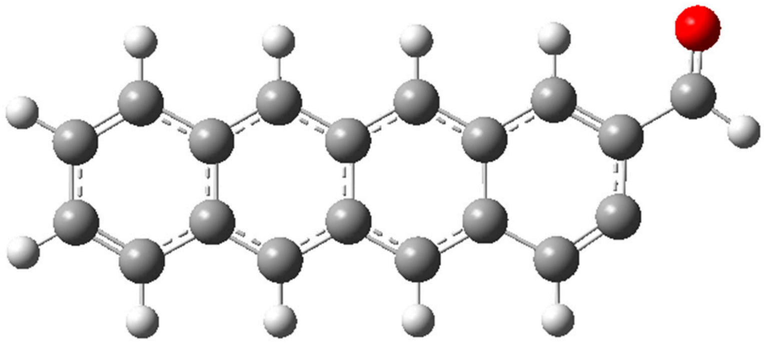

Figure S94 Visualization of the optimized structure of β-tetraldehyde radical, calculated at the M06-2X/cc-pVDZ level of theory.

Table S187 Geometry (Å) of β-tetraldehyde radical, calculated at the M06-2X/cc-pVDZ level of theory

Atom	x	y	z
C	5.607	0.507	0.000
C	4.478	1.268	0.000
C	3.177	0.656	0.000
C	3.093	-0.787	0.000
C	4.312	-1.549	0.000
C	5.523	-0.927	0.000
C	2.011	1.411	0.000
C	1.846	-1.397	0.000
C	0.656	-0.639	0.000
C	0.739	0.802	0.000
C	-0.453	1.560	0.000
H	-0.387	2.649	0.000
C	-1.697	0.950	0.000
C	-0.614	-1.251	0.000
H	2.077	2.500	0.000
H	6.587	0.982	0.000
H	4.540	2.356	0.000

H	4.244	-2.636	0.000
H	6.441	-1.513	0.000
H	1.781	-2.486	0.000
H	-0.680	-2.339	0.000
C	-1.782	-0.497	0.000
C	-4.222	-0.385	0.000
C	-4.083	1.029	0.000
C	-2.927	1.713	0.000
H	-2.884	2.802	0.000
C	-5.557	-1.037	0.000
O	-5.718	-2.235	0.000
H	-6.424	-0.342	0.000
C	-3.069	-1.128	0.000
H	-3.143	-2.217	0.000

Table S188 Frequencies (cm^{-1}) of β-tetraldehyde radical, calculated at the M06-2X/cc-pVDZ level of theory.

45	72	114	118	139	190	195	230
294	308	323	347	391	432	460	472
484	499	522	542	581	598	620	633
640	729	749	761	770	772	777	791
842	843	862	883	886	911	916	934
949	964	976	998	1018	1030	1038	1123
1155	1168	1176	1188	1210	1232	1264	1282
1296	1322	1327	1349	1387	1390	1439	1453
1459	1480	1488	1504	1589	1611	1627	1642
1675	1687	1715	1824	2984	3196	3198	3199
3200	3200	3202	3204	3205	3217	3229	

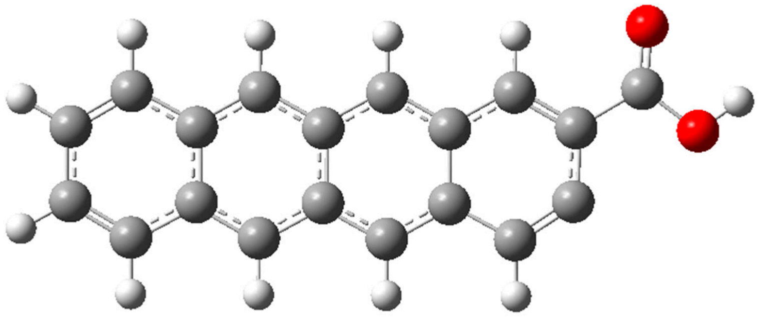

Figure S95 Visualization of the optimized structure of *α*-tetracenic acid radical, calculated at the M06-2X/cc-pVDZ level of theory.

Table S189 Geometry (Å) of β-tetracenic acid radical, calculated at the M06-2X/cc-pVDZ level of theory

Atom	x	y	z
C	5.912	0.325	0.000
C	4.827	1.147	0.000

**C**	3.494	0.608	0.000
**C**	3.331	-0.827	0.000
**C**	4.506	-1.655	0.000
**C**	5.750	-1.101	0.000
**C**	2.371	1.427	0.000
**C**	2.052	-1.368	0.000
**C**	0.906	-0.546	0.000
**C**	1.068	0.888	0.000
**C**	-0.080	1.711	0.000
**H**	0.045	2.794	0.000
**C**	-1.356	1.170	0.000
**C**	-0.397	-1.086	0.000
**H**	2.497	2.510	0.000
**H**	6.917	0.746	0.000
**H**	4.949	2.230	0.000
**H**	4.378	-2.738	0.000
**H**	6.633	-1.738	0.000
**H**	1.926	-2.452	0.000
**H**	-0.523	-2.169	0.000
**C**	-1.521	-0.269	0.000
**C**	-2.842	-0.828	0.000
**C**	-3.946	-0.021	0.000
**H**	-2.976	-1.910	0.000
**C**	-3.738	1.385	0.000
**C**	-2.544	1.999	0.000
**H**	-2.440	3.084	0.000
**C**	-5.301	-0.633	0.000
**O**	-5.523	-1.820	0.000
**O**	-6.279	0.294	0.000
**H**	-7.119	-0.187	0.000

Table S190 Frequencies (cm^{-1}) of β-tetracenic acid radical, calculated at the M06-2X/cc-pVDZ level of theory.

40	60	83	102	120	180	189	220
252	296	311	341	390	398	438	474
485	500	514	532	532	565	576	628
629	639	640	732	736	761	770	773
777	791	794	815	845	864	883	890
912	913	941	941	950	976	999	1020
1030	1123	1132	1155	1176	1183	1208	1211
1232	1263	1281	1295	1322	1326	1348	1378
1401	1431	1454	1462	1484	1488	1505	1592
1620	1627	1643	1682	1688	1715	1845	3195
3197	3199	3199	3200	3202	3204	3209	3217
3229	3822						
