# Supplementary material for: Substituent and Ring-Number Effects on the Kinetics of PAH + OH Reactions: A QSAR–DOE Approach with Tunneling Corrections
Source: Molecules. 2026 Jan 13;31(2):265. doi: 10.3390/molecules31020265 (PMC12843667; doi:10.3390/molecules31020265)
Supplement: Supplementary file 1 [file molecules-31-00265-s001.zip › Supporting Information - reactants.pdf]

Supporting information - reactants for

Substituent and Ring-Number Effects on the Kinetics of PAH + OH Reactions: A QSAR–DOE Approach with Tunneling Corrections

Cezary Parzych[1], Maciej Baradyn[2], Artur Ratkiewicz[2]*

Address: Institute of Chemistry, University of Bialystok, ul. Ciolkowskiego 1K, 15-245 Bialystok, Poland

[1] Doctoral School of University of Bialystok; 15-245 Bialystok, Ciołkowskiego 1K Street; Poland ; c.parzych@uwb.edu.pl
[2] Department of Physical Chemistry, University of Bialystok, Bialystok, Ciołkowskiego 1K Street; Poland

*Correspondence: artrat@uwb.edu.pl

Figure S1 Visualization of the optimized structure of benzene molecule, calculated at the M06-2X/cc-pVTZ level of theory. 18

Table S1 Geometry (Å) of benzene molecule, calculated at the M06-2X/aug-aug-cc-pVDZ level of theory 18

Table S2 Frequencies (cm^{-1}) of benzene molecule, calculated at the M06-2X/aug-aug-cc-pVDZ level of theory. 18

Figure S2 Visualization of the optimized structure of bromobenzene molecule, calculated at the M06-2X/aug-aug-cc-pVDZ level of theory. 19

Table S3 Geometry (Å) of bromobenzene molecule, calculated at the M06-2X/aug-aug-cc-pVDZ level of theory 19

Table S4 Frequencies (cm^{-1}) of bromobenzene molecule, calculated at the M06-2X/aug-aug-cc-pVDZ level of theory.... 19

Figure S3 Visualization of the optimized structure of chlorobenzene molecule, calculated at the M06-2X/aug-aug-cc-pVDZ level of theory. 20

Table S5 Geometry (Å) of chlorobenzene molecule, calculated at the M06-2X/aug-aug-cc-pVDZ level of theory 20

Table S6 Frequencies (cm^{-1}) of chlorobenzene molecule, calculated at the M06-2X/aug-aug-cc-pVDZ level of theory.... 20

Figure S4 Visualization of the optimized structure of fluorobenzene molecule, calculated at the M06-2X/aug-aug-cc-pVDZ level of theory. 21

Table S7 Geometry (Å) of chlorobenzene molecule, calculated at the M06-2X/aug-aug-cc-pVDZ level of theory 21

Table S8 Frequencies (cm^{-1}) of chlorobenzene molecule, calculated at the M06-2X/aug-aug-cc-pVDZ level of theory.... 21

Figure S5 Visualization of the optimized structure of aminobenzene molecule, calculated at the M06-2X/aug-aug-cc-pVDZ level of theory. 22

Table S9 Geometry (Å) of aminobenzene molecule, calculated at the M06-2X/aug-aug-cc-pVDZ level of theory 22

Table S10 Frequencies (cm^{-1}) of aminobenzene molecule, calculated at the M06-2X/aug-aug-cc-pVDZ level of theory.... 22

Figure S6 Visualization of the optimized structure of nitrobenzene molecule, calculated at the M06-2X/aug-aug-cc-pVDZ level of theory. 23

Table S11 Geometry (Å) of nitrobenzene molecule, calculated at the M06-2X/aug-aug-cc-pVDZ level of theory 23

Table S12 Frequencies (cm^{-1}) of aminobenzene molecule, calculated at the M06-2X/aug-aug-cc-pVDZ level of theory.... 23

Figure S7 Visualization of the optimized structure of methylbenzene molecule, calculated at the M06-2X/aug-aug-cc-pVDZ level of theory. 24

Table S13 Geometry (Å) of methylbenzene molecule, calculated at the M06-2X/aug-aug-cc-pVDZ level of theory 24

Table S14 Frequencies (cm^{-1}) of methylbenzene molecule, calculated at the M06-2X/aug-aug-cc-pVDZ level of theory. 24

Figure S8 Visualization of the optimized structure of ethylbenzene molecule, calculated at the M06-2X/aug-aug-cc-pVDZ level of theory. 25

Table S15 Geometry (Å) of ethylbenzene molecule, calculated at the M06-2X/aug-aug-cc-pVDZ level of theory 25

Table S16 Frequencies (cm^{-1}) of ethylbenzene molecule, calculated at the M06-2X/aug-aug-cc-pVDZ level of theory. 25

Figure S9 Visualization of the optimized structure of propylbenzene molecule, calculated at the M06-2X/aug-aug-cc-pVDZ level of theory. 26

Table S17 Geometry (Å) of propylbenzene molecule, calculated at the M06-2X/aug-aug-cc-pVDZ level of theory 26

Table S18 Frequencies (cm^{-1}) of propylbenzene molecule, calculated at the M06-2X/aug-aug-cc-pVDZ level of theory. 27

Figure S10 Visualization of the optimized structure of butylbenzene molecule, calculated at the M06-2X/aug-aug-cc-pVDZ level of theory. 27

Table S19 Geometry (Å) of butylbenzene molecule, calculated at the M06-2X/aug-aug-cc-pVDZ level of theory 27

Table S20 Frequencies (cm^{-1}) of butylbenzene molecule, calculated at the M06-2X/aug-aug-cc-pVDZ level of theory. 28

Figure S11 Visualization of the optimized structure of hydroxybenzene molecule, calculated at the M06-2X/aug-aug-cc-pVDZ level of theory. 28

Table S21 Geometry (Å) of hydroxybenzene molecule, calculated at the M06-2X/aug-aug-cc-pVDZ level of theory 28

Table S22 Frequencies (cm^{-1}) of hydroxybenzene molecule, calculated at the M06-2X/aug-aug-cc-pVDZ level of theory. 29

Figure S12 Visualization of the optimized structure of peroxybenzene molecule, calculated at the M06-2X/aug-aug-cc-pVDZ level of theory. 29

Table S23 Geometry (Å) of peroxybenzene molecule, calculated at the M06-2X/aug-aug-cc-pVDZ level of theory 29

Table S24 Frequencies (cm^{-1}) of peroxybenzene molecule, calculated at the M06-2X/aug-aug-cc-pVDZ level of theory. 30

Figure S13 Visualization of the optimized structure of benzaldehyde molecule, calculated at the M06-2X/aug-aug-cc-pVDZ level of theory. 30
Table S25 Geometry (Å) of benzaldehyde molecule, calculated at the M06-2X/aug-aug-cc-pVDZ level of theory 30
Table S26 Frequencies (cm^{-1}) of benzaldehyde molecule, calculated at the M06-2X/aug-aug-cc-pVDZ level of theory 31
Figure S14 Visualization of the optimized structure of benzoic acid molecule, calculated at the M06-2X/aug-aug-cc-pVDZ level of theory. 31
Table S27 Geometry (Å) of benzoic acid molecule, calculated at the M06-2X/aug-aug-cc-pVDZ level of theory 31
Table S28 Frequencies (cm^{-1}) of benzaldehyde molecule, calculated at the M06-2X/aug-aug-cc-pVDZ level of theory 32
Figure S15 Visualization of the optimized structure of naphtalene molecule, calculated at the M06-2X/aug-aug-cc-pVDZ level of theory. 32
Table S29 Geometry (Å) of naphtalene molecule, calculated at the M06-2X/aug-aug-cc-pVDZ level of theory 32
Table S30 Frequencies (cm^{-1}) of naphtalene molecule, calculated at the M06-2X/aug-aug-cc-pVDZ level of theory. 33
Figure S16 Visualization of the optimized structure of α-bromonaphtalene molecule, calculated at the M06-2X/aug-aug-cc-pVDZ level of theory. 33
Table S31 Geometry (Å) of α-bromonaphtalene molecule, calculated at the M06-2X/aug-aug-cc-pVDZ level of theory 33
Table S32 Frequencies (cm^{-1}) of α-bromonaphtalene molecule, calculated at the M06-2X/aug-aug-cc-pVDZ level of theory. 34
Figure S17 Visualization of the optimized structure of α-chloronaphtalene molecule, calculated at the M06-2X/aug-aug-cc-pVDZ level of theory. 34
Table S33 Geometry (Å) of α-chloronaphtalene molecule, calculated at the M06-2X/aug-aug-cc-pVDZ level of theory 34
Table S34 Frequencies (cm^{-1}) of α-chloronaphtalene molecule, calculated at the M06-2X/aug-aug-cc-pVDZ level of theory. 35
Figure S18 Visualization of the optimized structure of α-fluoronaphtalene molecule, calculated at the M06-2X/aug-aug-cc-pVDZ level of theory. 35
Table S35 Geometry (Å) of α-fluoronaphtalene molecule, calculated at the M06-2X/aug-aug-cc-pVDZ level of theory 35
Table S36 Frequencies (cm^{-1}) of α-fluoronaphtalene molecule, calculated at the M06-2X/aug-aug-cc-pVDZ level of theory. 36

Figure S19 Visualization of the optimized structure of α-aminonaphtalene molecule, calculated at the M06-2X/aug-aug-cc-pVDZ level of theory. 36

Table S37 Geometry (Å) of α-aminonaphtalene molecule, calculated at the M06-2X/aug-aug-cc-pVDZ level of theory 36

Table S38 Frequencies (cm^{-1}) of α-aminonaphtalene molecule, calculated at the M06-2X/aug-aug-cc-pVDZ level of theory. 37

Figure S20 Visualization of the optimized structure of α-nitronaphtalene molecule, calculated at the M06-2X/aug-aug-cc-pVDZ level of theory. 37

Table S39 Geometry (Å) of α-nitronaphtalene molecule, calculated at the M06-2X/aug-aug-cc-pVDZ level of theory 37

Table S40 Frequencies (cm^{-1}) of α-nitronaphtalene molecule, calculated at the M06-2X/aug-aug-cc-pVDZ level of theory. 38

Figure S21 Visualization of the optimized structure of α-methylnaphtalene molecule, calculated at the M06-2X/aug-aug-cc-pVDZ level of theory. 38

Table S41 Geometry (Å) of α-methylnaphtalene molecule, calculated at the M06-2X/aug-aug-cc-pVDZ level of theory 38

Table S42 Frequencies (cm^{-1}) of α-methylnaphtalene molecule, calculated at the M06-2X/aug-aug-cc-pVDZ level of theory. 39

Figure S22 Visualization of the optimized structure of α-ethylnaphtalene molecule, calculated at the M06-2X/aug-aug-cc-pVDZ level of theory. 39

Table S43 Geometry (Å) of α-ethylnaphtalene molecule, calculated at the M06-2X/aug-aug-cc-pVDZ level of theory 39

Table S44 Frequencies (cm^{-1}) of α-ethylnaphtalene molecule, calculated at the M06-2X/aug-aug-cc-pVDZ level of theory. 40

Figure S23 Visualization of the optimized structure of α-propylnaphtalene molecule, calculated at the M06-2X/aug-aug-cc-pVDZ level of theory. 40

Table S45 Geometry (Å) of α-propylnaphtalene molecule, calculated at the M06-2X/aug-aug-cc-pVDZ level of theory 41

Table S46 Frequencies (cm^{-1}) of α-propylnaphtalene molecule, calculated at the M06-2X/aug-aug-cc-pVDZ level of theory. 41

Figure S24 Visualization of the optimized structure of α-butylnaphtalene molecule, calculated at the M06-2X/aug-aug-cc-pVDZ level of theory. 42

Table S47 Geometry (Å) of α-butylnaphtalene molecule, calculated at the M06-2X/aug-aug-cc-pVDZ level of theory 42

Table S48 Frequencies (cm^{-1}) of α-butylnaphtalene molecule, calculated at the M06-2X/aug-aug-cc-pVDZ level of theory. 43

Figure S25 Visualization of the optimized structure of α-hydroxynaphtalene molecule, calculated at the M06-2X/aug-aug-cc-pVDZ level of theory. 43

Table S49 Geometry (Å) of α-hydroxynaphtalene molecule, calculated at the M06-2X/aug-aug-cc-pVDZ level of theory 43

Table S50 Frequencies (cm^{-1}) of α-hydroxynaphtalene molecule, calculated at the M06-2X/aug-aug-cc-pVDZ level of theory. 44

Figure S26 Visualization of the optimized structure of α-peroxynaphtalene molecule, calculated at the M06-2X/aug-aug-cc-pVDZ level of theory. 44

Table S51 Geometry (Å) of α-peroxynaphtalene molecule, calculated at the M06-2X/aug-aug-cc-pVDZ level of theory 44

Table S52 Frequencies (cm^{-1}) of α-peroxynaphtalene molecule, calculated at the M06-2X/aug-aug-cc-pVDZ level of theory. 45

Figure S27 Visualization of the optimized structure of α-naphtaldehyde molecule, calculated at the M06-2X/aug-aug-cc-pVDZ level of theory. 45

Table S53 Geometry (Å) of α-naphtaldehyde molecule, calculated at the M06-2X/aug-aug-cc-pVDZ level of theory 45

Table S54 Frequencies (cm^{-1}) of α-naphtaldehyde molecule, calculated at the M06-2X/aug-aug-cc-pVDZ level of theory. 46

Figure S28 Visualization of the optimized structure of α-naphtalenic acid molecule, calculated at the M06-2X/aug-aug-cc-pVDZ level of theory. 46

Table S55 Geometry (Å) of α-naphtalenic aicd molecule, calculated at the M06-2X/aug-aug-cc-pVDZ level of theory 47

Table S56 Frequencies (cm^{-1}) of α-naphtalenic acid molecule, calculated at the M06-2X/aug-aug-cc-pVDZ level of theory. 47

Figure S29 Visualization of the optimized structure of β-bromonaphtalene molecule, calculated at the M06-2X/aug-aug-cc-pVDZ level of theory. 48

Table S57 Geometry (Å) of β-bromonaphtalene molecule, calculated at the M06-2X/aug-aug-cc-pVDZ level of theory 48

Table S58 Frequencies (cm^{-1}) of β-bromonaphtalene molecule, calculated at the M06-2X/aug-aug-cc-pVDZ level of theory. 48

Figure S30 Visualization of the optimized structure of β-chloronaphtalene molecule, calculated at the M06-2X/aug-aug-cc-pVDZ level of theory. 48

Table S59 Geometry (Å) of β-chloronaphtalene molecule, calculated at the M06-2X/aug-aug-cc-pVDZ level of theory 49

Table S60 Frequencies (cm^{-1}) of β-chloronaphtalene molecule, calculated at the M06-2X/aug-aug-cc-pVDZ level of theory. 49

Figure S31 Visualization of the optimized structure of β-fluoronaphtalene molecule, calculated at the M06-2X/aug-aug-cc-pVDZ level of theory. 49

Table S61 Geometry (Å) of β-fluoronaphtalene molecule, calculated at the M06-2X/aug-aug-cc-pVDZ level of theory 50

Table S62 Frequencies (cm^{-1}) of β-fluoronaphtalene molecule, calculated at the M06-2X/aug-aug-cc-pVDZ level of theory. 50

Figure S32 Visualization of the optimized structure of β-aminonaphtalene molecule, calculated at the M06-2X/aug-aug-cc-pVDZ level of theory. 50

Table S63 Geometry (Å) of β-aminonaphtalene molecule, calculated at the M06-2X/aug-aug-cc-pVDZ level of theory 51

Table S64 Frequencies (cm^{-1}) of β-aminonaphtalene molecule, calculated at the M06-2X/aug-aug-cc-pVDZ level of theory. 51

Figure S33 Visualization of the optimized structure of β-nitronaphtalene molecule, calculated at the M06-2X/aug-aug-cc-pVDZ level of theory. 52

Table S65 Geometry (Å) of β-nitronaphtalene molecule, calculated at the M06-2X/aug-aug-cc-pVDZ level of theory 52

Table S66 Frequencies (cm^{-1}) of β-nitronaphtalene molecule, calculated at the M06-2X/aug-aug-cc-pVDZ level of theory. 52

Figure S34 Visualization of the optimized structure of β-methylnaphtalene molecule, calculated at the M06-2X/aug-aug-cc-pVDZ level of theory. 53

Table S67 Geometry (Å) of β-methylnaphtalene molecule, calculated at the M06-2X/aug-aug-cc-pVDZ level of theory 53

Table S68 Frequencies (cm^{-1}) of β-methylnaphtalene molecule, calculated at the M06-2X/aug-aug-cc-pVDZ level of theory. 53

Figure S35 Visualization of the optimized structure of β-ethylnaphtalene molecule, calculated at the M06-2X/aug-aug-cc-pVDZ level of theory. 54

Table S69 Geometry (Å) of β-ethylnaphtalene molecule, calculated at the M06-2X/aug-aug-cc-pVDZ level of theory 54

Table S70 Frequencies (cm^{-1}) of β-ethylnaphtalene molecule, calculated at the M06-2X/aug-aug-cc-pVDZ level of theory. 55

Figure S36 Visualization of the optimized structure of β-propylnaphtalene molecule, calculated at the M06-2X/aug-aug-cc-pVDZ level of theory. 55

Table S71 Geometry (Å) of β-propylnaphtalene molecule, calculated at the M06-2X/aug-aug-cc-pVDZ level of theory 55

Table S72 Frequencies (cm^{-1}) of β-propylnaphtalene molecule, calculated at the M06-2X/aug-aug-cc-pVDZ level of theory. 56

Figure S37 Visualization of the optimized structure of β-butylnaphtalene molecule, calculated at the M06-2X/aug-aug-cc-pVDZ level of theory. 56

Table S73 Geometry (Å) of β-butylnaphtalene molecule, calculated at the M06-2X/aug-aug-cc-pVDZ level of theory 56

Table S74 Frequencies (cm^{-1}) of β-butylnaphtalene molecule, calculated at the M06-2X/aug-aug-cc-pVDZ level of theory. 57

Figure S38 Visualization of the optimized structure of β-hydroxynaphtalene molecule, calculated at the M06-2X/aug-aug-cc-pVDZ level of theory. 58

Table S75 Geometry (Å) of β-hydroxynaphtalene molecule, calculated at the M06-2X/aug-aug-cc-pVDZ level of theory 58

Table S76 Frequencies (cm^{-1}) of β-hydroxynaphtalene molecule, calculated at the M06-2X/aug-aug-cc-pVDZ level of theory. 58

Figure S39 Visualization of the optimized structure of β-peroxynaphtalene molecule, calculated at the M06-2X/aug-aug-cc-pVDZ level of theory. 59

Table S77 Geometry (Å) of β-peroxynaphtalene molecule, calculated at the M06-2X/aug-aug-cc-pVDZ level of theory 59

Table S78 Frequencies (cm^{-1}) of β-peroxynaphtalene molecule, calculated at the M06-2X/aug-aug-cc-pVDZ level of theory. 59

Figure S40 Visualization of the optimized structure of β-naphtaldehyde molecule, calculated at the M06-2X/aug-aug-cc-pVDZ level of theory. 60

Table S79 Geometry (Å) of β-naphtaldehyde molecule, calculated at the M06-2X/aug-aug-cc-pVDZ level of theory 60

Table S80 Frequencies (cm^{-1}) of β-naphtaldehyde molecule, calculated at the M06-2X/aug-aug-cc-pVDZ level of theory. 60

Figure S41 Visualization of the optimized structure of β-naphtalenic acid molecule, calculated at the M06-2X/aug-aug-cc-pVDZ level of theory. 61

Table S81 Geometry (Å) of β-naphtalenic aicd molecule, calculated at the M06-2X/aug-aug-cc-pVDZ level of theory 61

Table S82 Frequencies (cm^{-1}) of β-naphtalenic acid molecule, calculated at the M06-2X/aug-aug-cc-pVDZ level of theory. 62

Figure S42 Visualization of the optimized structure of antracene molecule, calculated at the M06-2X/aug-aug-cc-pVDZ level of theory. 62

Table S83 Geometry (Å) of antracene molecule, calculated at the M06-2X/aug-aug-cc-pVDZ level of theory 62

Table S84 Frequencies (cm^{-1}) of antracene molecule, calculated at the M06-2X/aug-aug-cc-pVDZ level of theory. 63

Figure S43 Visualization of the optimized structure of α-bromoantracene molecule, calculated at the M06-2X/aug-aug-cc-pVDZ level of theory. 63
Table S85 Geometry (Å) of α-bromoantracene molecule, calculated at the M06-2X/aug-aug-cc-pVDZ level of theory 63
Table S86 Frequencies (cm^{-1}) of α-bromoantracene molecule, calculated at the M06-2X/aug-aug-cc-pVDZ level of theory. 64
Figure S44 Visualization of the optimized structure of α-chloroantracene molecule, calculated at the M06-2X/aug-aug-cc-pVDZ level of theory. 64
Table S87 Geometry (Å) of α-chloroantracene molecule, calculated at the M06-2X/aug-aug-cc-pVDZ level of theory 64
Table S88 Frequencies (cm^{-1}) of α-chloroantracene molecule, calculated at the M06-2X/aug-aug-cc-pVDZ level of theory. 65
Figure S45 Visualization of the optimized structure of α-fluoroantracene molecule, calculated at the M06-2X/aug-aug-cc-pVDZ level of theory. 65
Table S89 Geometry (Å) of α-fluoroantracene molecule, calculated at the M06-2X/aug-aug-cc-pVDZ level of theory 66
Table S90 Frequencies (cm^{-1}) of α-fluoroantracene molecule, calculated at the M06-2X/aug-aug-cc-pVDZ level of theory. 66
Figure S46 Visualization of the optimized structure of α-aminoantracene molecule, calculated at the M06-2X/aug-aug-cc-pVDZ level of theory. 67
Table S91 Geometry (Å) of α-aminoantracene molecule, calculated at the M06-2X/aug-aug-cc-pVDZ level of theory 67
Table S92 Frequencies (cm^{-1}) of α-aminoantracene molecule, calculated at the M06-2X/aug-aug-cc-pVDZ level of theory. 67
Figure S47 Visualization of the optimized structure of α-nitroantracene molecule, calculated at the M06-2X/aug-aug-cc-pVDZ level of theory. 68
Table S93 Geometry (Å) of α-nitroantracene molecule, calculated at the M06-2X/aug-aug-cc-pVDZ level of theory 68
Table S94 Frequencies (cm^{-1}) of α-nitroantracene molecule, calculated at the M06-2X/aug-aug-cc-pVDZ level of theory. 69
Figure S48 Visualization of the optimized structure of α-methylantracene molecule, calculated at the M06-2X/aug-aug-cc-pVDZ level of theory. 69
Table S95 Geometry (Å) of α-methylantracene molecule, calculated at the M06-2X/aug-aug-cc-pVDZ level of theory 69
Table S96 Frequencies (cm^{-1}) of α-methylantracene molecule, calculated at the M06-2X/aug-aug-cc-pVDZ level of theory. 70

Figure S49 Visualization of the optimized structure of α-ethylantracene molecule, calculated at the M06-2X/aug-aug-cc-pVDZ level of theory. 70

Table S97 Geometry (Å) of α-ethylantracene molecule, calculated at the M06-2X/aug-aug-cc-pVDZ level of theory 71

Table S98 Frequencies (cm^{-1}) of α-ethylantracene molecule, calculated at the M06-2X/aug-aug-cc-pVDZ level of theory. 71

Figure S50 Visualization of the optimized structure of α-propylantracene molecule, calculated at the M06-2X/aug-aug-cc-pVDZ level of theory. 72

Table S99 Geometry (Å) of α-propylantracene molecule, calculated at the M06-2X/aug-aug-cc-pVDZ level of theory 72

Table S100 Frequencies (cm^{-1}) of α-propylantracene molecule, calculated at the M06-2X/aug-aug-cc-pVDZ level of theory. 73

Figure S51 Visualization of the optimized structure of α-butylantracene molecule, calculated at the M06-2X/aug-aug-cc-pVDZ level of theory. 73

Table S101 Geometry (Å) of α-butylantracene molecule, calculated at the M06-2X/aug-aug-cc-pVDZ level of theory 73

Table S102 Frequencies (cm^{-1}) of α-butylantracene molecule, calculated at the M06-2X/aug-aug-cc-pVDZ level of theory. 74

Figure S52 Visualization of the optimized structure of α-hydroxyantracene molecule, calculated at the M06-2X/aug-aug-cc-pVDZ level of theory. 75

Table S103 Geometry (Å) of α-hydroxyantracene molecule, calculated at the M06-2X/aug-aug-cc-pVDZ level of theory 75

Table S104 Frequencies (cm^{-1}) of α-hydroxyantracene molecule, calculated at the M06-2X/aug-aug-cc-pVDZ level of theory. 76

Figure S53 Visualization of the optimized structure of α-peroxyantracene molecule, calculated at the M06-2X/aug-aug-cc-pVDZ level of theory. 76

Table S105 Geometry (Å) of α-peroxyantracene molecule, calculated at the M06-2X/aug-aug-cc-pVDZ level of theory 76

Table S106 Frequencies (cm^{-1}) of α-peroxyantracene molecule, calculated at the M06-2X/aug-aug-cc-pVDZ level of theory. 77

Figure S54 Visualization of the optimized structure of α-antraldehyde molecule, calculated at the M06-2X/aug-aug-cc-pVDZ level of theory. 77

Table S107 Geometry (Å) of α-antraldehyde molecule, calculated at the M06-2X/aug-aug-cc-pVDZ level of theory 77

Table S108 Frequencies (cm^{-1}) of α-antraldehyde molecule, calculated at the M06-2X/aug-aug-cc-pVDZ level of theory. 78

Figure S55 Visualization of the optimized structure of α-antracenic acid molecule, calculated at the M06-2X/aug-aug-cc-pVDZ level of theory. ... 78

Table S109 Geometry (Å) of α-antracenic acid molecule, calculated at the M06-2X/aug-aug-cc-pVDZ level of theory ... 79

Table S110 Frequencies (cm^{-1}) of α-antracenic acid molecule, calculated at the M06-2X/aug-aug-cc-pVDZ level of theory. ... 79

Figure S56 Visualization of the optimized structure of β-bromoantracene molecule, calculated at the M06-2X/aug-aug-cc-pVDZ level of theory. ... 80

Table S111 Geometry (Å) of β-bromoantracene molecule, calculated at the M06-2X/aug-aug-cc-pVDZ level of theory ... 80

Table S112 Frequencies (cm^{-1}) of β-bromoantracene molecule, calculated at the M06-2X/aug-aug-cc-pVDZ level of theory. ... 80

Figure S57 Visualization of the optimized structure of β-chloroantracene molecule, calculated at the M06-2X/aug-aug-cc-pVDZ level of theory. ... 81

Table S113 Geometry (Å) of β-chloroantracene molecule, calculated at the M06-2X/aug-aug-cc-pVDZ level of theory ... 81

Table S114 Frequencies (cm^{-1}) of β-chloroantracene molecule, calculated at the M06-2X/aug-aug-cc-pVDZ level of theory. ... 82

Figure S58 Visualization of the optimized structure of β-fluoroantracene molecule, calculated at the M06-2X/aug-aug-cc-pVDZ level of theory. ... 82

Table S115 Geometry (Å) of β-fluoroantracene molecule, calculated at the M06-2X/aug-aug-cc-pVDZ level of theory ... 82

Table S116 Frequencies (cm^{-1}) of β-fluoroantracene molecule, calculated at the M06-2X/aug-aug-cc-pVDZ level of theory. ... 83

Figure S59 Visualization of the optimized structure of β-aminoantracene molecule, calculated at the M06-2X/aug-aug-cc-pVDZ level of theory. ... 83

Table S117 Geometry (Å) of β-aminoantracene molecule, calculated at the M06-2X/aug-aug-cc-pVDZ level of theory ... 83

Table S118 Frequencies (cm^{-1}) of β-aminoantracene molecule, calculated at the M06-2X/aug-aug-cc-pVDZ level of theory. ... 84

Figure S60 Visualization of the optimized structure of β-nitroantracene molecule, calculated at the M06-2X/aug-aug-cc-pVDZ level of theory. ... 84

Table S119 Geometry (Å) of β-nitroantracene molecule, calculated at the M06-2X/aug-aug-cc-pVDZ level of theory ... 85

Table S120 Frequencies (cm^{-1}) of β-nitroantracene molecule, calculated at the M06-2X/aug-aug-cc-pVDZ level of theory. ... 85

Figure S61 Visualization of the optimized structure of α β-methylantracene molecule, calculated at the M06-2X/aug-aug-cc-pVDZ level of theory. 86
Table S121 Geometry (Å) of β-methylantracene molecule, calculated at the M06-2X/aug-aug-cc-pVDZ level of theory 86
Table S122 Frequencies (cm^{-1}) of β-methylantracene molecule, calculated at the M06-2X/aug-aug-cc-pVDZ level of theory. 87
Figure S62 Visualization of the optimized structure of β-ethylantracene molecule, calculated at the M06-2X/aug-aug-cc-pVDZ level of theory. 87
Table S123 Geometry (Å) of β-ethylantracene molecule, calculated at the M06-2X/aug-aug-cc-pVDZ level of theory 87
Table S124 Frequencies (cm^{-1}) of β-ethylantracene molecule, calculated at the M06-2X/aug-aug-cc-pVDZ level of theory. 88
Figure S63 Visualization of the optimized structure of β-propylantracene molecule, calculated at the M06-2X/aug-aug-cc-pVDZ level of theory. 88
Table S125 Geometry (Å) of β-propylantracene molecule, calculated at the M06-2X/aug-aug-cc-pVDZ level of theory 88
Table S126 Frequencies (cm^{-1}) of β-propylantracene molecule, calculated at the M06-2X/aug-aug-cc-pVDZ level of theory. 89
Figure S64 Visualization of the optimized structure of β-butylantracene molecule, calculated at the M06-2X/aug-aug-cc-pVDZ level of theory. 90
Table S127 Geometry (Å) of β-butylantracene molecule, calculated at the M06-2X/aug-aug-cc-pVDZ level of theory 90
Table S128 Frequencies (cm^{-1}) of β-butylantracene molecule, calculated at the M06-2X/aug-aug-cc-pVDZ level of theory. 91
Figure S65 Visualization of the optimized structure of β-hydroxyantracene molecule, calculated at the M06-2X/aug-aug-cc-pVDZ level of theory. 91
Table S129 Geometry (Å) of β-hydroxyantracene molecule, calculated at the M06-2X/aug-aug-cc-pVDZ level of theory 91
Table S130 Frequencies (cm^{-1}) of β-hydroxyantracene molecule, calculated at the M06-2X/aug-aug-cc-pVDZ level of theory. 92
Figure S66 Visualization of the optimized structure of β-peroxyantracene molecule, calculated at the M06-2X/aug-aug-cc-pVDZ level of theory. 92
Table S131 Geometry (Å) of β-peroxyantracene molecule, calculated at the M06-2X/aug-aug-cc-pVDZ level of theory 93
Table S132 Frequencies (cm^{-1}) of β-peroxyantracene molecule, calculated at the M06-2X/aug-aug-cc-pVDZ level of theory. 93

Figure S67 Visualization of the optimized structure of β-antraldehyde molecule, calculated at the M06-2X/aug-aug-cc-pVDZ level of theory. ... 94

Table S133 Geometry (Å) of β-antraldehyde molecule, calculated at the M06-2X/aug-aug-cc-pVDZ level of theory ... 94

Table S134 Frequencies (cm^{-1}) of β-antraldehyde molecule, calculated at the M06-2X/aug-aug-cc-pVDZ level of theory. ... 94

Figure S68 Visualization of the optimized structure of β-antracenic acid molecule, calculated at the M06-2X/aug-aug-cc-pVDZ level of theory. ... 95

Table S135 Geometry (Å) of β-antracenic acid molecule, calculated at the M06-2X/aug-aug-cc-pVDZ level of theory ... 95

Table S136 Frequencies (cm^{-1}) of β-antracenic acid molecule, calculated at the M06-2X/aug-aug-cc-pVDZ level of theory. ... 96

Figure S69 Visualization of the optimized structure of tetracene molecule, calculated at the M06-2X/aug-aug-cc-pVDZ level of theory. ... 96

Table S137 Geometry (Å) of tetracene molecule, calculated at the M06-2X/aug-aug-cc-pVDZ level of theory ... 96

Table S138 Frequencies (cm^{-1}) of tetracene molecule, calculated at the M06-2X/aug-aug-cc-pVDZ level of theory. ... 97

Figure S70 Visualization of the optimized structure of α-bromotetracene molecule, calculated at the M06-2X/aug-aug-cc-pVDZ level of theory. ... 97

Table S139 Geometry (Å) of α-bromotetracene molecule, calculated at the M06-2X/aug-aug-cc-pVDZ level of theory ... 98

Table S140 Frequencies (cm^{-1}) of α-bromotetracene molecule, calculated at the M06-2X/aug-aug-cc-pVDZ level of theory. ... 98

Figure S71 Visualization of the optimized structure of α-chlorotetracene acid molecule, calculated at the M06-2X/aug-aug-cc-pVDZ level of theory. ... 99

Table S141 Geometry (Å) of α-chlorotetracene molecule, calculated at the M06-2X/aug-aug-cc-pVDZ level of theory ... 99

Table S142 Frequencies (cm^{-1}) of α-chlorotetracene molecule, calculated at the M06-2X/aug-aug-cc-pVDZ level of theory. ... 100

Figure S72 Visualization of the optimized structure of α-fluorotetracene molecule, calculated at the M06-2X/aug-aug-cc-pVDZ level of theory. ... 100

Table S143 Geometry (Å) of α-fluorotetracene molecule, calculated at the M06-2X/aug-aug-cc-pVDZ level of theory ... 100

Table S144 Frequencies (cm^{-1}) of α-fluorotetracene molecule, calculated at the M06-2X/aug-aug-cc-pVDZ level of theory. ... 101

Figure S73 Visualization of the optimized structure of α-aminotetracene molecule, calculated at the M06-2X/aug-aug-cc-pVDZ level of theory. 101
Table S145 Geometry (Å) of α-aminotetracene molecule, calculated at the M06-2X/aug-aug-cc-pVDZ level of theory 102
Table S146 Frequencies (cm^{-1}) of α-aminotetracene molecule, calculated at the M06-2X/aug-aug-cc-pVDZ level of theory. 102
Figure S74 Visualization of the optimized structure of α-nitrotetracene molecule, calculated at the M06-2X/aug-aug-cc-pVDZ level of theory. 103
Table S147 Geometry (Å) of α-nitrotetracene molecule, calculated at the M06-2X/aug-aug-cc-pVDZ level of theory 103
Table S148 Frequencies (cm^{-1}) of α-nitrotetracene molecule, calculated at the M06-2X/aug-aug-cc-pVDZ level of theory. 104
Figure S75 Visualization of the optimized structure of α-methyltetracene molecule, calculated at the M06-2X/aug-aug-cc-pVDZ level of theory. 104
Table S149 Geometry (Å) of α-methyltetracene molecule, calculated at the M06-2X/aug-aug-cc-pVDZ level of theory 104
Table S150 Frequencies (cm^{-1}) of α-methyltetracene molecule, calculated at the M06-2X/aug-aug-cc-pVDZ level of theory. 105
Figure S76 Visualization of the optimized structure of α-ethyltetracene molecule, calculated at the M06-2X/aug-aug-cc-pVDZ level of theory. 106
Table S151 Geometry (Å) of α-ethyltetracene molecule, calculated at the M06-2X/aug-aug-cc-pVDZ level of theory 106
Table S152 Frequencies (cm^{-1}) of α-ethyltetracene molecule, calculated at the M06-2X/aug-aug-cc-pVDZ level of theory. 107
Figure S77 Visualization of the optimized structure of α-propyltetracene molecule, calculated at the M06-2X/aug-aug-cc-pVDZ level of theory. 107
Table S153 Geometry (Å) of α-propyltetracene molecule, calculated at the M06-2X/aug-aug-cc-pVDZ level of theory 107
Table S154 Frequencies (cm^{-1}) of α-propyltetracene molecule, calculated at the M06-2X/aug-aug-cc-pVDZ level of theory. 108
Figure S78 Visualization of the optimized structure of α-butyltetracene molecule, calculated at the M06-2X/aug-aug-cc-pVDZ level of theory. 109
Table S155 Geometry (Å) of α-butyltetracene molecule, calculated at the M06-2X/aug-aug-cc-pVDZ level of theory 109
Table S156 Frequencies (cm^{-1}) of α-butyltetracene molecule, calculated at the M06-2X/aug-aug-cc-pVDZ level of theory. 110

Figure S79 Visualization of the optimized structure of α-hydroxytetracene molecule, calculated at the M06-2X/aug-aug-cc-pVDZ level of theory. 111

Table S157 Geometry (Å) of α-hydroxytetracene molecule, calculated at the M06-2X/aug-aug-cc-pVDZ level of theory 111

Table S158 Frequencies (cm^{-1}) of α-hydroxytetracene molecule, calculated at the M06-2X/aug-aug-cc-pVDZ level of theory. 112

Figure S80 Visualization of the optimized structure of α-peroxytetracene molecule, calculated at the M06-2X/aug-aug-cc-pVDZ level of theory. 112

Table S159 Geometry (Å) of α-peroxytetracene molecule, calculated at the M06-2X/aug-aug-cc-pVDZ level of theory 112

Table S160 Frequencies (cm^{-1}) of α-peroxytetracene molecule, calculated at the M06-2X/aug-aug-cc-pVDZ level of theory. 113

Figure S81 Visualization of the optimized structure of α-tetraldehyde molecule, calculated at the M06-2X/aug-aug-cc-pVDZ level of theory. 113

Table S161 Geometry (Å) of α-tetraldehyde molecule, calculated at the M06-2X/aug-aug-cc-pVDZ level of theory 114

Table S162 Frequencies (cm^{-1}) of α-tetraldehyde molecule, calculated at the M06-2X/aug-aug-cc-pVDZ level of theory. 114

Figure S82 Visualization of the optimized structure of α-tetracenic acid molecule, calculated at the M06-2X/aug-aug-cc-pVDZ level of theory. 115

Table S163 Geometry (Å) of α-tetracenic acid molecule, calculated at the M06-2X/aug-aug-cc-pVDZ level of theory 115

Table S164 Frequencies (cm^{-1}) of α-tetracenic acid molecule, calculated at the M06-2X/aug-aug-cc-pVDZ level of theory. 116

Figure S83 Visualization of the optimized structure of β-bromotetracene molecule, calculated at the M06-2X/aug-aug-cc-pVDZ level of theory. 116

Table S165 Geometry (Å) of β-bromotetracene molecule, calculated at the M06-2X/aug-aug-cc-pVDZ level of theory 116

Table S166 Frequencies (cm^{-1}) of β-bromotetracene molecule, calculated at the M06-2X/aug-aug-cc-pVDZ level of theory. 117

Figure S84 Visualization of the optimized structure of β-chlorotetracene acid molecule, calculated at the M06-2X/aug-aug-cc-pVDZ level of theory. 118

Table S167 Geometry (Å) of β-chlorotetracene molecule, calculated at the M06-2X/aug-aug-cc-pVDZ level of theory 118

Table S168 Frequencies (cm^{-1}) of β-chlorotetracene molecule, calculated at the M06-2X/aug-aug-cc-pVDZ level of theory. 119

Figure S85 Visualization of the optimized structure of β-fluorotetracene molecule, calculated at the M06-2X/aug-aug-cc-pVDZ level of theory. 119

Table S169 Geometry (Å) of β-fluorotetracene molecule, calculated at the M06-2X/aug-aug-cc-pVDZ level of theory 119

Table S170 Frequencies (cm^{-1}) of β-fluorotetracene molecule, calculated at the M06-2X/aug-aug-cc-pVDZ level of theory. 120

Figure S86 Visualization of the optimized structure of β-aminotetracene molecule, calculated at the M06-2X/aug-aug-cc-pVDZ level of theory. 120

Table S171 Geometry (Å) of β-aminotetracene molecule, calculated at the M06-2X/aug-aug-cc-pVDZ level of theory 120

Table S172 Frequencies (cm^{-1}) of β-aminotetracene molecule, calculated at the M06-2X/aug-aug-cc-pVDZ level of theory. 121

Figure S87 Visualization of the optimized structure of β-nitrotetracene molecule, calculated at the M06-2X/aug-aug-cc-pVDZ level of theory. 122

Table S173 Geometry (Å) of β-nitrotetracene molecule, calculated at the M06-2X/aug-aug-cc-pVDZ level of theory 122

Table S174 Frequencies (cm^{-1}) of β-nitrotetracene molecule, calculated at the M06-2X/aug-aug-cc-pVDZ level of theory. 123

Figure S88 Visualization of the optimized structure of β-methyltetracene molecule, calculated at the M06-2X/aug-aug-cc-pVDZ level of theory. 123

Table S175 Geometry (Å) of β-methyltetracene molecule, calculated at the M06-2X/aug-aug-cc-pVDZ level of theory 123

Table S176 Frequencies (cm^{-1}) of β-methyltetracene molecule, calculated at the M06-2X/aug-aug-cc-pVDZ level of theory. 124

Figure S89 Visualization of the optimized structure of β-ethyltetracene molecule, calculated at the M06-2X/aug-aug-cc-pVDZ level of theory. 125

Table S177 Geometry (Å) of β-ethyltetracene molecule, calculated at the M06-2X/aug-aug-cc-pVDZ level of theory 125

Table S178 Frequencies (cm^{-1}) of β-ethyltetracene molecule, calculated at the M06-2X/aug-aug-cc-pVDZ level of theory. 125

Figure S90 Visualization of the optimized structure of β-propyltetracene molecule, calculated at the M06-2X/aug-aug-cc-pVDZ level of theory. 126

Table S179 Geometry (Å) of β-propyltetracene molecule, calculated at the M06-2X/aug-aug-cc-pVDZ level of theory 126

Table S180 Frequencies (cm^{-1}) of β-propyltetracene molecule, calculated at the M06-2X/aug-aug-cc-pVDZ level of theory. 127

Figure S91 Visualization of the optimized structure of β-butyltetracene molecule, calculated at the M06-2X/aug-aug-cc-pVDZ level of theory. 128

Table S181 Geometry (Å) of β-butyltetracene molecule, calculated at the M06-2X/aug-aug-cc-pVDZ level of theory 128

Table S182 Frequencies (cm^{-1}) of β-butyltetracene molecule, calculated at the M06-2X/aug-aug-cc-pVDZ level of theory. 129

Figure S92 Visualization of the optimized structure of β-hydroxytetracene molecule, calculated at the M06-2X/aug-aug-cc-pVDZ level of theory. 129

Table S183 Geometry (Å) of β-hydroxytetracene molecule, calculated at the M06-2X/aug-aug-cc-pVDZ level of theory 129

Table S184 Frequencies (cm^{-1}) of β-hydroxytetracene molecule, calculated at the M06-2X/aug-aug-cc-pVDZ level of theory. 130

Figure S93 Visualization of the optimized structure of α-peroxytetracene molecule, calculated at the M06-2X/aug-aug-cc-pVDZ level of theory. 131

Table S185 Geometry (Å) of β-peroxytetracene molecule, calculated at the M06-2X/aug-aug-cc-pVDZ level of theory 131

Table S186 Frequencies (cm^{-1}) of β-peroxytetracene molecule, calculated at the M06-2X/aug-aug-cc-pVDZ level of theory. 132

Figure S94 Visualization of the optimized structure of β-tetraldehyde molecule, calculated at the M06-2X/aug-aug-cc-pVDZ level of theory. 132

Table S187 Geometry (Å) of β-tetraldehyde molecule, calculated at the M06-2X/aug-aug-cc-pVDZ level of theory 132

Table S188 Frequencies (cm^{-1}) of β-tetraldehyde molecule, calculated at the M06-2X/aug-aug-cc-pVDZ level of theory. 133

Figure S95 Visualization of the optimized structure of α-tetracenic acid molecule, calculated at the M06-2X/aug-aug-cc-pVDZ level of theory. 134

Table S189 Geometry (Å) of β-tetracenic acid molecule, calculated at the M06-2X/aug-aug-cc-pVDZ level of theory 134

Table S190 Frequencies (cm^{-1}) of β-tetracenic acid molecule, calculated at the M06-2X/aug-aug-cc-pVDZ level of theory. 134

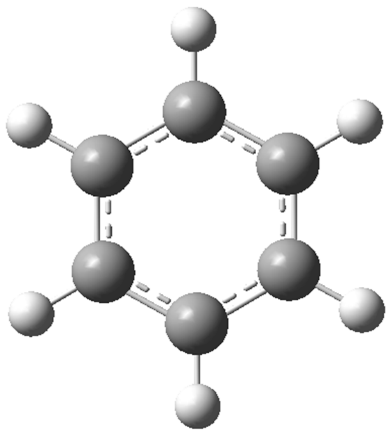

Figure S1 Visualization of the optimized structure of benzene molecule, calculated at the M06-2X/cc-pVTZ level of theory.

Table S1 Geometry (Å) of benzene molecule, calculated at the M06-2X/aug-aug-cc-pVDZ level of theory

Atom	x	y	z
**C**	-4.832	-1.535	0.435
**C**	-3.766	-1.879	-0.397
**C**	-3.024	-0.880	-1.028
**C**	-3.348	0.462	-0.828
**C**	-4.414	0.806	0.004
**C**	-5.156	-0.193	0.636
**H**	-3.513	-2.927	-0.554
**H**	-2.192	-1.148	-1.678
**H**	-2.769	1.242	-1.321
**H**	-5.411	-2.315	0.928
**H**	-4.667	1.853	0.161
**H**	-5.988	0.075	1.285

Table S2 Frequencies (cm^{-1}) of benzene molecule, calculated at the M06-2X/aug-aug-cc-pVDZ level of theory.

411	412	613	613	691	733	873	875
1001	1003	1007	1032	1032	1068	1069	1156
1189	1190	1345	1353	1507	1508	1671	1671
3200	3207	3209	3221	3223	3231		

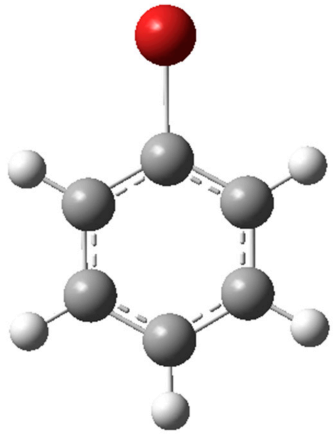

Figure S2 Visualization of the optimized structure of bromobenzene molecule, calculated at the M06-2X/aug-aug-cc-pVDZ level of theory.

Table S3 Geometry (Å) of bromobenzene molecule, calculated at the M06-2X/aug-aug-cc-pVDZ level of theory

Atom	x	y	z
C	-4.770	-1.528	0.521
C	-3.704	-1.867	-0.312
C	-3.024	-0.880	-1.027
C	-3.413	0.453	-0.908
C	-4.477	0.808	-0.079
C	-5.142	-0.191	0.626
H	-3.406	-2.910	-0.400
H	-2.193	-1.151	-1.676
H	-2.887	1.229	-1.463
H	-4.787	1.846	0.020
H	-5.305	-2.291	1.082
Br	-6.593	0.281	1.759

Table S4 Frequencies (cm^{-1}) of bromobenzene molecule, calculated at the M06-2X/aug-aug-cc-pVDZ level of theory.

172	258	325	414	473	621	683	707
759	854	933	996	1005	1019	1054	1102
1104	1167	1191	1313	1336	1475	1506	1657
1661	3212	3219	3231	3238	3240		

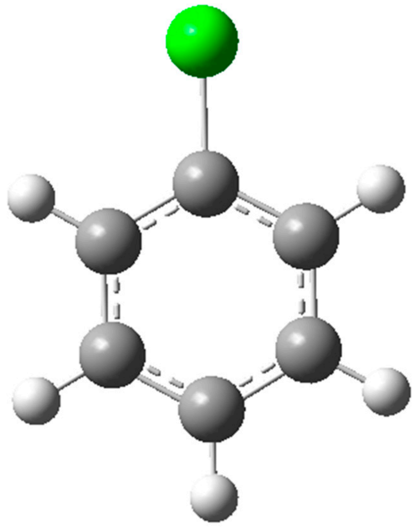

Figure S3 Visualization of the optimized structure of chlorobenzene molecule, calculated at the M06-2X/aug-aug-cc-pVDZ level of theory.

Table S5 Geometry (Å) of chlorobenzene molecule, calculated at the M06-2X/aug-aug-cc-pVDZ level of theory

Atom	x	y	z
C	-4.769	-1.528	0.521
C	-3.704	-1.867	-0.312
C	-3.024	-0.881	-1.027
C	-3.413	0.453	-0.908
C	-4.477	0.808	-0.079
C	-5.142	-0.191	0.626
H	-3.405	-2.910	-0.401
H	-2.193	-1.151	-1.676
H	-2.887	1.229	-1.463
H	-4.790	1.845	0.022
H	-5.307	-2.288	1.083
Cl	-6.477	0.243	1.668

Table S6 Frequencies (cm^{-1}) of chlorobenzene molecule, calculated at the M06-2X/aug-aug-cc-pVDZ level of theory.

190	300	417	422	483	621	708	716
762	852	931	994	1010	1017	1055	1100
1124	1167	1187	1311	1338	1478	1509	1662
1663	3211	3218	3229	3235	3238		

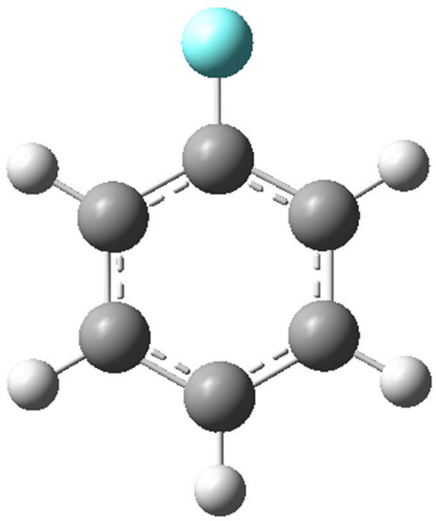

Figure S4 Visualization of the optimized structure of fluorobenzene molecule, calculated at the M06-2X/aug-aug-cc-pVDZ level of theory.

Table S7 Geometry (Å) of chlorobenzene molecule, calculated at the M06-2X/aug-aug-cc-pVDZ level of theory

Atom	x	y	z
C	-4.769	-1.531	0.521
C	-3.703	-1.868	-0.313
C	-3.024	-0.881	-1.027
C	-3.412	0.454	-0.909
C	-4.476	0.811	-0.080
C	-5.130	-0.195	0.617
H	-3.403	-2.911	-0.402
H	-2.193	-1.151	-1.676
H	-2.885	1.228	-1.465
H	-4.801	1.843	0.032
H	-5.317	-2.279	1.090
F	-6.161	0.141	1.421

Table S8 Frequencies (cm^{-1}) of chlorobenzene molecule, calculated at the M06-2X/aug-aug-cc-pVDZ level of theory.

239	405	424	509	521	621	707	778
830	844	925	986	1008	1017	1047	1089
1165	1165	1274	1308	1348	1488	1533	1678
1684	3213	3220	3233	3240	3242		

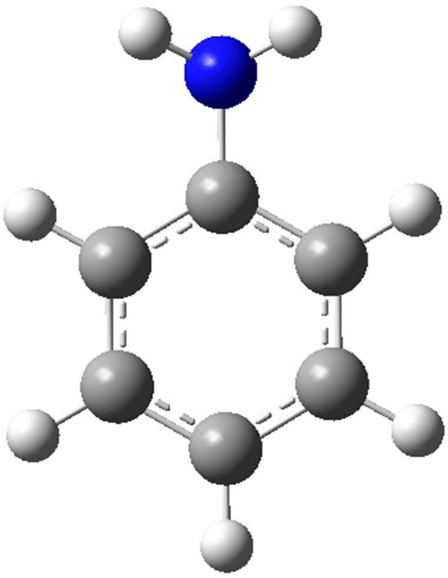

Figure S5 Visualization of the optimized structure of aminobenzene molecule, calculated at the M06-2X/aug-aug-cc-pVDZ level of theory.

Table S9 Geometry (Å) of aminobenzene molecule, calculated at the M06-2X/aug-aug-cc-pVDZ level of theory

Atom	x	y	z
C	-4.787	-1.526	0.489
C	-3.701	-1.862	-0.314
C	-3.004	-0.881	-1.019
C	-3.412	0.448	-0.907
C	-4.497	0.795	-0.107
C	-5.199	-0.190	0.601
H	-3.398	-2.906	-0.389
H	-2.156	-1.148	-1.646
H	-2.880	1.229	-1.450
H	-4.803	1.838	-0.022
H	-5.321	-2.300	1.041
N	-6.250	0.160	1.454
H	-6.712	1.029	1.222
H	-6.914	-0.581	1.635

Table S10 Frequencies (cm^{-1}) of aminobenzene molecule, calculated at the M06-2X/aug-aug-cc-pVDZ level of theory.

223	298	382	418	505	533	591	628
709	770	838	839	900	986	1002	1007
1059	1073	1130	1163	1189	1312	1345	1353
1502	1536	1635	1664	1687	3196	3196	3212
3217	3236	3589	3696				

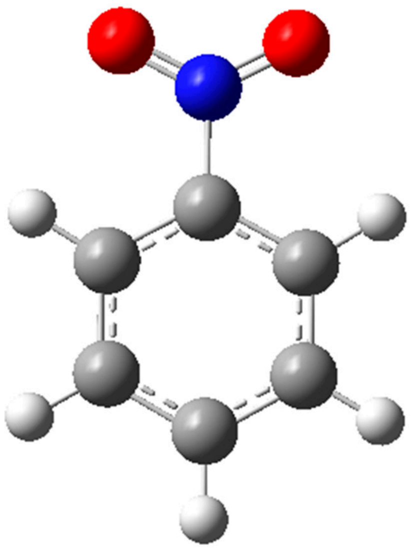

Figure S6 Visualization of the optimized structure of nitrobenzene molecule, calculated at the M06-2X/aug-aug-cc-pVDZ level of theory.

Table S11 Geometry (Å) of nitrobenzene molecule, calculated at the M06-2X/aug-aug-cc-pVDZ level of theory

Atom	x	y	z
C	-4.719	-1.636	0.503
C	-3.658	-1.905	-0.358
C	-3.027	-0.864	-1.040
C	-3.452	0.454	-0.868
C	-4.512	0.742	-0.012
C	-5.122	-0.314	0.655
H	-3.323	-2.932	-0.495
H	-2.197	-1.082	-1.711
H	-2.957	1.263	-1.403
H	-5.233	-2.422	1.050
N	-6.247	-0.019	1.565
H	-4.868	1.756	0.145
O	-6.768	-0.956	2.138
O	-6.585	1.142	1.683

Table S12 Frequencies (cm^{-1}) of aminobenzene molecule, calculated at the M06-2X/aug-aug-cc-pVDZ level of theory.

57	174	264	405	418	448	532	619
699	699	730	830	864	886	971	1009
1017	1030	1053	1101	1143	1170	1192	1319
1360	1475	1492	1514	1667	1668	1722	3217
3228	3236	3257	3257				

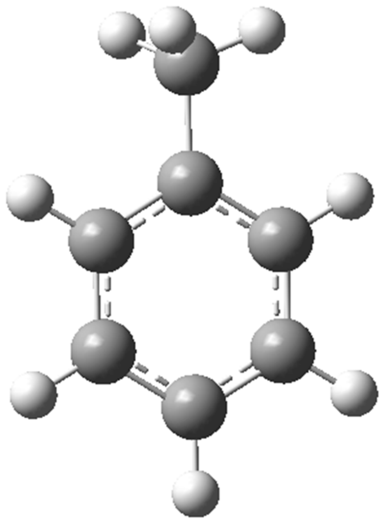

Figure S7 Visualization of the optimized structure of methylbenzene molecule, calculated at the M06-2X/aug-aug-cc-pVDZ level of theory.

Table S13 Geometry (Å) of methylbenzene molecule, calculated at the M06-2X/aug-aug-cc-pVDZ level of theory

Atom	x	y	z
C	0.201	-1.198	-0.012
C	-1.191	-1.208	-0.012
C	-1.901	-0.006	0.000
C	-1.206	1.200	0.012
C	0.191	1.204	0.011
C	0.912	0.009	-0.001
H	-1.726	-2.157	-0.021
H	-2.990	-0.012	0.001
H	-1.749	2.144	0.021
H	0.749	-2.141	-0.022
C	2.420	0.003	-0.001
H	2.818	1.023	0.009
H	2.809	-0.525	0.879
H	2.808	-0.508	-0.892
H	0.727	2.153	0.020

Table S14 Frequencies (cm^{-1}) of methylbenzene molecule, calculated at the M06-2X/aug-aug-cc-pVDZ level of theory.

66	214	345	414	477	525	630	714
744	805	863	918	996	998	1012	1015
1052	1061	1112	1165	1194	1246	1328	1345
1396	1460	1464	1495	1533	1662	1686	3062
3128	3155	3191	3196	3209	3216	3230	

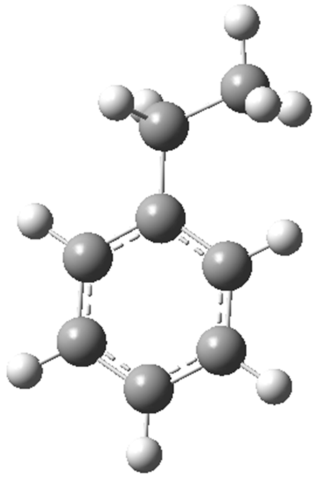

Figure S8 Visualization of the optimized structure of ethylbenzene molecule, calculated at the M06-2X/aug-aug-cc-pVDZ level of theory.

Table S15 Geometry (Å) of ethylbenzene molecule, calculated at the M06-2X/aug-aug-cc-pVDZ level of theory

Atom	x	y	z
C	-4.880	-1.551	0.283
C	-3.741	-1.871	-0.462
C	-2.933	-0.864	-0.980
C	-3.270	0.472	-0.748
C	-4.404	0.785	-0.006
C	-5.227	-0.219	0.522
H	-3.489	-2.916	-0.636
H	-2.046	-1.114	-1.561
H	-2.646	1.270	-1.149
H	-4.662	1.830	0.171
H	-5.496	-2.356	0.678
C	-6.450	0.178	1.323
H	-6.120	0.808	2.161
H	-7.078	0.823	0.692
C	-7.290	-0.976	1.859
H	-6.704	-1.615	2.532
H	-8.148	-0.591	2.422
H	-7.674	-1.601	1.043

Table S16 Frequencies (cm^{-1}) of ethylbenzene molecule, calculated at the M06-2X/aug-aug-cc-pVDZ level of theory.

57	188	236	302	410	417	481	544
628	719	723	778	801	867	934	993
1002	1015	1018	1062	1076	1101	1123	1165
1199	1219	1285	1321	1348	1362	1396	1467
1479	1483	1488	1534	1661	1684	3059	3064
3092	3139	3149	3189	3205	3214	3226	3232

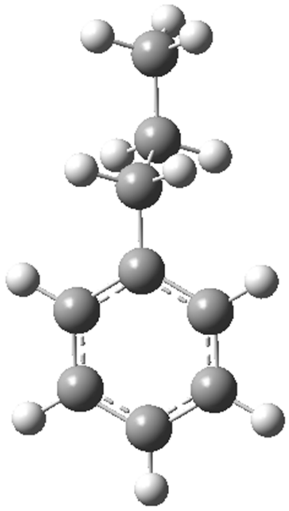

Figure S9 Visualization of the optimized structure of propylbenzene molecule, calculated at the M06-2X/aug-aug-cc-pVDZ level of theory.

Table S17 Geometry (Å) of propylbenzene molecule, calculated at the M06-2X/aug-aug-cc-pVDZ level of theory

Atom	x	y	z
C	0.175	-0.891	-0.029
C	1.573	-0.873	-0.020
C	2.274	0.333	0.002
C	1.582	1.544	0.016
C	0.188	1.539	0.007
C	-0.507	0.330	-0.015
H	2.120	-1.817	-0.034
H	3.364	0.327	0.005
H	2.127	2.486	0.030
H	-0.361	2.480	0.013
H	-1.598	0.333	-0.026
C	-0.579	-2.196	0.003
H	-0.006	-2.968	-0.529
H	-1.538	-2.083	-0.523
C	-0.849	-2.671	1.436
H	-1.413	-1.892	1.967
H	0.111	-2.773	1.962
C	-1.611	-3.991	1.472
H	-1.045	-4.782	0.961
H	-1.797	-4.318	2.502
H	-2.582	-3.894	0.967

Table S18 Frequencies (cm^{-1}) of propylbenzene molecule, calculated at the M06-2X/aug-aug-cc-pVDZ level of theory.

43	91	105	244	281	314	350	415
512	596	628	719	734	770	833	862
870	909	936	997	1013	1016	1060	1070
1073	1117	1119	1164	1192	1234	1236	1299
1304	1339	1347	1372	1393	1465	1470	1476
1484	1485	1532	1662	1684	3055	3058	3065
3090	3111	3133	3142	3188	3189	3207	3214
3228							

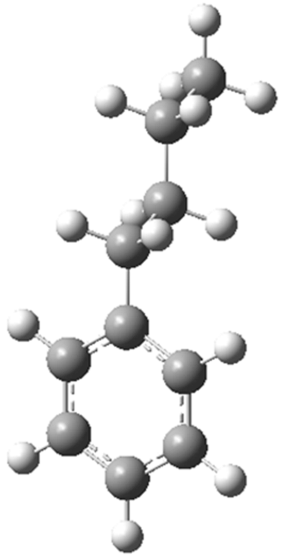

Figure S10 Visualization of the optimized structure of butylbenzene molecule, calculated at the M06-2X/aug-aug-cc-pVDZ level of theory.

Table S19 Geometry (Å) of butylbenzene molecule, calculated at the M06-2X/aug-aug-cc-pVDZ level of theory

Atom	x	y	z
C	1.216	0.088	-0.034
C	2.615	0.106	-0.026
C	3.316	1.311	-0.003
C	2.624	2.522	0.013
C	1.229	2.517	0.005
C	0.534	1.309	-0.018
H	3.161	-0.838	-0.041
H	4.405	1.306	-0.001
H	3.169	3.465	0.028
H	0.681	3.459	0.013
H	-0.557	1.311	-0.028
C	0.462	-1.217	0.000
H	1.035	-1.990	-0.533
H	-0.497	-1.104	-0.525
C	0.195	-1.692	1.433

**H**	-0.370	-0.914	1.968
**H**	1.155	-1.796	1.960
**C**	-0.567	-3.012	1.483
**H**	0.005	-3.778	0.940
**H**	-1.521	-2.897	0.948
**C**	-0.831	-3.481	2.911
**H**	-1.423	-2.737	3.461
**H**	-1.380	-4.430	2.928
**H**	0.112	-3.625	3.454

Table S20 Frequencies (cm^{-1}) of butylbenzene molecule, calculated at the M06-2X/aug-aug-cc-pVDZ level of theory.

31	76	79	118	216	238	286	350
391	413	525	590	628	717	720	772
783	833	862	907	923	936	993	1012
1014	1043	1060	1074	1088	1120	1128	1164
1191	1219	1231	1264	1290	1310	1338	1346
1347	1388	1395	1462	1467	1475	1476	1484
1486	1530	1661	1684	3047	3054	3058	3064
3077	3094	3114	3132	3141	3190	3190	3206
3216	3228						

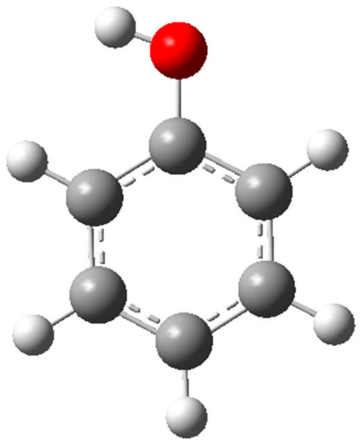

Figure S11 Visualization of the optimized structure of hydroxybenzene molecule, calculated at the M06-2X/aug-aug-cc-pVDZ level of theory.

Table S21 Geometry (Å) of hydroxybenzene molecule, calculated at the M06-2X/aug-aug-cc-pVDZ level of theory

Atom	x	y	z
**C**	-4.828	-1.607	0.484
**C**	-3.759	-1.892	-0.360
**C**	-3.070	-0.866	-1.011
**C**	-3.464	0.455	-0.809
**C**	-4.534	0.754	0.034

**C**	-5.214	-0.280	0.679
**H**	-3.460	-2.929	-0.511
**H**	-2.235	-1.096	-1.669
**H**	-2.936	1.265	-1.310
**H**	-5.375	-2.395	0.998
**H**	-4.840	1.790	0.191
**O**	-6.269	-0.053	1.517
**H**	-6.438	0.893	1.570

Table S22 Frequencies (cm^{-1}) of hydroxybenzene molecule, calculated at the M06-2X/aug-aug-cc-pVDZ level of theory.

232	366	408	422	518	532	626	711
777	837	840	915	987	1009	1011	1054
1098	1161	1180	1203	1304	1345	1364	1506
1537	1677	1686	3191	3210	3218	3230	3237
3880							

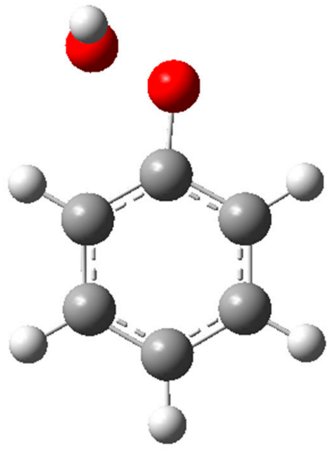

Figure S12 Visualization of the optimized structure of peroxybenzene molecule, calculated at the M06-2X/aug-aug-cc-pVDZ level of theory.

Table S23 Geometry (Å) of peroxybenzene molecule, calculated at the M06-2X/aug-aug-cc-pVDZ level of theory

Atom	x	y	z
**C**	-0.067	1.069	0.028
**C**	1.301	1.353	0.005
**C**	2.247	0.333	-0.021
**C**	1.821	-0.997	-0.021
**C**	0.465	-1.304	0.002
**C**	-0.467	-0.264	0.024
**H**	1.622	2.394	0.010
**H**	3.309	0.569	-0.038
**H**	2.550	-1.805	-0.038

**H**	-0.805	1.863	0.059
**H**	0.113	-2.334	0.004
**O**	-1.780	-0.684	0.049
**O**	-2.669	0.412	0.051
**H**	-3.005	0.391	-0.857

Table S24 Frequencies (cm^{-1}) of peroxybenzene molecule, calculated at the M06-2X/aug-aug-cc-pVDZ level of theory.

76	216	258	276	420	449	514	608
620	711	771	824	845	917	991	1009
1010	1047	1059	1104	1163	1175	1257	1322
1355	1436	1489	1523	1674	1678	3211	3218
3229	3237	3270	3808				

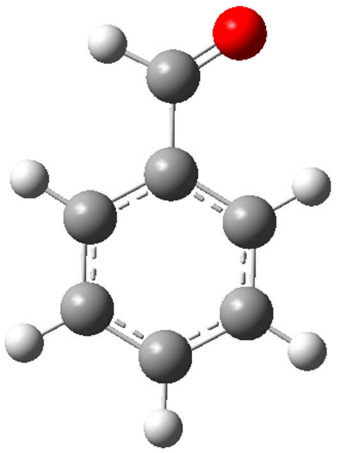

Figure S13 Visualization of the optimized structure of benzaldehyde molecule, calculated at the M06-2X/aug-aug-cc-pVDZ level of theory.

Table S25 Geometry (Å) of benzaldehyde molecule, calculated at the M06-2X/aug-aug-cc-pVDZ level of theory

Atom	x	y	z
**C**	-4.803	-1.586	0.413
**C**	-3.667	-1.880	-0.331
**C**	-2.969	-0.854	-0.977
**C**	-3.405	0.467	-0.879
**C**	-4.544	0.763	-0.133
**C**	-5.242	-0.260	0.512
**H**	-3.319	-2.908	-0.413
**H**	-2.079	-1.088	-1.559
**H**	-2.858	1.262	-1.383
**H**	-4.897	1.792	-0.048
**H**	-5.367	-2.364	0.926
**O**	-7.122	-0.745	1.896
**C**	-6.454	0.070	1.304

**H**	-6.726	1.150	1.324

Table S26 Frequencies (cm^{-1}) of benzaldehyde molecule, calculated at the M06-2X/aug-aug-cc-pVDZ level of theory.

120	224	238	419	443	472	623	659
714	769	843	876	953	1012	1016	1027
1046	1054	1102	1167	1180	1236	1322	1355
1411	1486	1526	1664	1679	1833	2955	3196
3208	3220	3228	3234				

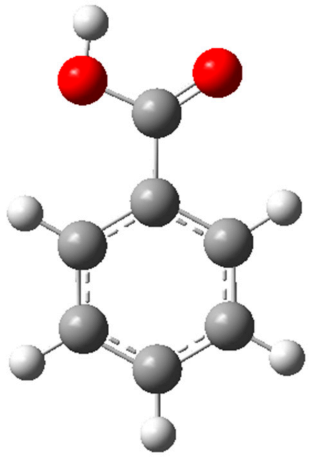

Figure S14 Visualization of the optimized structure of benzoic acid molecule, calculated at the M06-2X/aug-aug-cc-pVDZ level of theory.

Table S27 Geometry (Å) of benzoic acid molecule, calculated at the M06-2X/aug-aug-cc-pVDZ level of theory

Atom	x	y	z
**C**	-4.825	-1.551	0.423
**C**	-3.689	-1.861	-0.319
**C**	-2.982	-0.848	-0.968
**C**	-3.410	0.477	-0.877
**C**	-4.546	0.794	-0.136
**C**	-5.253	-0.223	0.514
**H**	-3.353	-2.894	-0.392
**H**	-2.093	-1.092	-1.548
**H**	-2.856	1.266	-1.384
**H**	-4.889	1.823	-0.058
**H**	-5.395	-2.321	0.939
**O**	-7.124	-0.774	1.900
**C**	-6.473	0.061	1.318
**O**	-6.800	1.372	1.342
**H**	-7.597	1.444	1.886

Table S28 Frequencies (cm^{-1}) of benzaldehyde molecule, calculated at the M06-2X/aug-aug-cc-pVDZ level of theory.

68	165	220	387	416	441	500	606
624	641	715	731	784	838	874	973
1013	1015	1031	1056	1103	1137	1168	1188
1231	1327	1353	1397	1483	1530	1662	1682
1849	3208	3220	3228	3236	3243	3821	

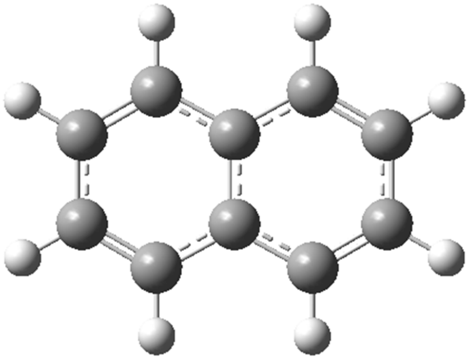

Figure S15 Visualization of the optimized structure of naphtalene molecule, calculated at the M06-2X/aug-aug-cc-pVDZ level of theory.

Table S29 Geometry (Å) of naphtalene molecule, calculated at the M06-2X/aug-aug-cc-pVDZ level of theory

Atom	x	y	z
C	2.430	0.709	0.000
C	1.244	1.402	0.000
C	0.000	0.712	0.000
C	0.000	-0.712	0.000
C	1.244	-1.402	0.000
C	2.430	-0.709	0.000
H	-1.239	2.492	0.000
H	1.239	2.492	0.000
C	-1.244	1.402	0.000
C	-1.244	-1.402	0.000
H	1.239	-2.492	0.000
C	-2.430	-0.709	0.000
C	-2.430	0.709	0.000
H	-1.239	-2.492	0.000
H	3.376	1.248	0.000
H	3.376	-1.248	0.000
H	-3.376	1.248	0.000
H	-3.376	-1.248	0.000

Table S30 Frequencies (cm^{-1}) of naphtalene molecule, calculated at the M06-2X/aug-aug-cc-pVDZ level of theory.

177	187	374	403	488	496	512	522
628	650	735	785	803	809	813	861
929	940	978	1002	1015	1023	1044	1057
1151	1155	1169	1172	1231	1260	1287	1406
1410	1439	1490	1496	1566	1661	1679	1714
3198	3198	3201	3202	3216	3216	3227	3228

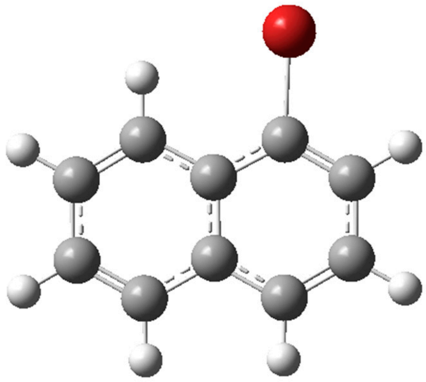

Figure S16 Visualization of the optimized structure of α-bromonaphtalene molecule, calculated at the M06-2X/aug-aug-cc-pVDZ level of theory.

Table S31 Geometry (Å) of α-bromonaphtalene molecule, calculated at the M06-2X/aug-aug-cc-pVDZ level of theory

Atom	x	y	z
C	2.707	-1.019	0.005
C	1.427	-1.518	-0.002
C	0.309	-0.640	-0.006
C	0.539	0.770	-0.003
C	1.877	1.248	0.005
C	2.938	0.378	0.008
H	1.258	-2.592	-0.005
C	-1.048	-1.080	-0.013
C	-0.562	1.668	-0.006
H	2.043	2.326	0.007
C	-1.849	1.196	-0.013
C	-2.099	-0.199	-0.016
H	-0.363	2.739	-0.004
H	3.553	-1.705	0.008
H	3.958	0.758	0.014
H	-2.692	1.884	-0.016
H	-3.121	-0.572	-0.022
Br	-1.447	-2.941	-0.018

Table S32 Frequencies (cm^{-1}) of α-bromonaphtalene molecule, calculated at the M06-2X/aug-aug-cc-pVDZ level of theory.

107	178	180	233	308	422	424	485
517	536	558	647	663	756	800	801
828	834	903	938	961	994	1010	1028
1056	1091	1157	1160	1178	1224	1234	1275
1378	1407	1427	1470	1492	1559	1648	1672
1706	3201	3207	3214	3225	3227	3238	3244

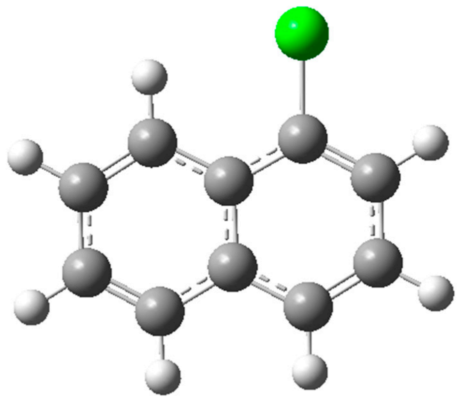

Figure S17 Visualization of the optimized structure of α-chloronaphtalene molecule, calculated at the M06-2X/aug-aug-cc-pVDZ level of theory.

Table S33 Geometry (Å) of α-chloronaphtalene molecule, calculated at the M06-2X/aug-aug-cc-pVDZ level of theory

Atom	x	y	z
C	2.703	-1.023	0.005
C	1.422	-1.518	-0.002
C	0.308	-0.635	-0.006
C	0.538	0.773	-0.002
C	1.878	1.249	0.005
C	2.936	0.375	0.008
H	1.247	-2.591	-0.005
C	-1.047	-1.077	-0.013
C	-0.564	1.671	-0.006
H	2.047	2.325	0.008
C	-1.850	1.196	-0.013
C	-2.100	-0.198	-0.016
H	-0.367	2.742	-0.004
H	3.548	-1.710	0.007
H	3.958	0.752	0.014
H	-2.693	1.885	-0.016
H	-3.120	-0.577	-0.021
Cl	-1.394	-2.791	-0.018

Table S34 Frequencies (cm^{-1}) of α-chloronaphtalene molecule, calculated at the M06-2X/aug-aug-cc-pVDZ level of theory.

119	178	226	243	398	425	437	484
521	546	566	650	680	756	801	802
828	850	902	933	981	994	1008	1028
1058	1094	1158	1165	1178	1227	1236	1277
1380	1411	1434	1473	1496	1562	1654	1675
1710	3200	3205	3212	3222	3226	3236	3242

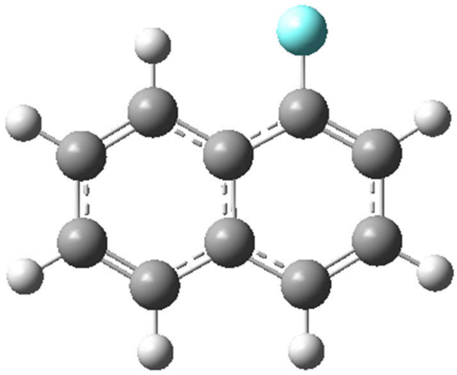

Figure S18 Visualization of the optimized structure of α-fluoronaphtalene molecule, calculated at the M06-2X/aug-aug-cc-pVDZ level of theory.

Table S35 Geometry (Å) of α-fluoronaphtalene molecule, calculated at the M06-2X/aug-aug-cc-pVDZ level of theory

Atom	x	y	z
C	2.692	-1.033	0.005
C	1.407	-1.518	-0.003
C	0.310	-0.616	-0.006
C	0.539	0.789	-0.002
C	1.884	1.252	0.005
C	2.932	0.365	0.009
H	1.211	-2.588	-0.006
C	-1.037	-1.055	-0.014
C	-0.570	1.680	-0.006
H	2.067	2.326	0.008
C	-1.854	1.195	-0.013
C	-2.103	-0.201	-0.017
H	-0.383	2.753	-0.003
H	3.534	-1.723	0.007
H	3.957	0.733	0.014
H	-2.699	1.881	-0.016
H	-3.114	-0.601	-0.022
F	-1.253	-2.387	-0.017

Table S36 Frequencies (cm^{-1}) of α-fluoronaphtalene molecule, calculated at the M06-2X/aug-aug-cc-pVDZ level of theory.

149	180	273	274	432	470	475	485
537	576	592	655	729	756	797	799
836	886	906	923	990	1004	1023	1043
1061	1103	1160	1170	1181	1236	1258	1287
1392	1428	1444	1479	1508	1568	1666	1685
1723	3204	3210	3215	3227	3228	3240	3242

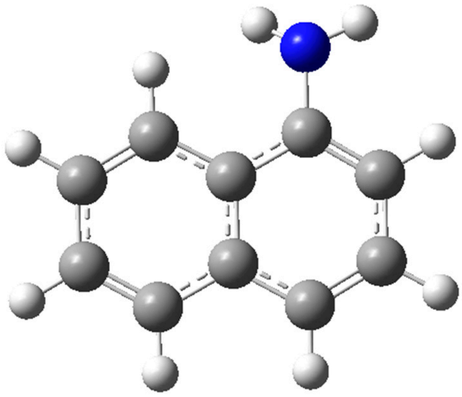

Figure S19 Visualization of the optimized structure of α-aminonaphtalene molecule, calculated at the M06-2X/aug-aug-cc-pVDZ level of theory.

Table S37 Geometry (Å) of α-aminonaphtalene molecule, calculated at the M06-2X/aug-aug-cc-pVDZ level of theory

Atom	x	y	z
C	-8.044	-0.999	-1.030
C	-7.017	-0.454	-0.296
C	-6.936	0.948	-0.081
C	-7.924	1.792	-0.665
C	-8.976	1.197	-1.413
C	-9.042	-0.164	-1.589
H	-6.242	-1.110	0.097
C	-5.886	1.543	0.696
C	-7.840	3.202	-0.503
H	-9.734	1.847	-1.851
C	-6.807	3.744	0.220
C	-5.835	2.917	0.827
H	-8.598	3.835	-0.960
H	-8.087	-2.075	-1.191
H	-9.854	-0.603	-2.166
H	-5.034	3.368	1.414
H	-6.733	4.823	0.345
N	-4.899	0.726	1.256
H	-4.322	1.203	1.936
H	-5.237	-0.154	1.625

Table S38 Frequencies (cm^{-1}) of α-aminonaphtalene molecule, calculated at the M06-2X/aug-aug-cc-pVDZ level of theory.

135	177	260	285	334	433	462	481
485	520	578	581	635	678	731	749
796	797	827	886	894	911	983	997
1018	1042	1060	1116	1138	1160	1179	1198
1240	1270	1313	1401	1435	1441	1484	1504
1571	1635	1669	1677	1709	3195	3200	3209
3209	3220	3225	3230	3581	3683		

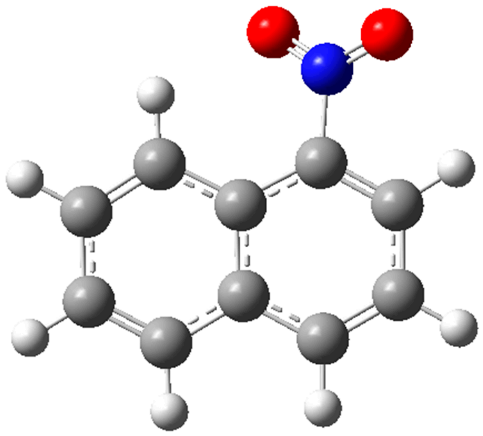

Figure S20 Visualization of the optimized structure of α-nitronaphtalene molecule, calculated at the M06-2X/aug-aug-cc-pVDZ level of theory.

Table S39 Geometry (Å) of α-nitronaphtalene molecule, calculated at the M06-2X/aug-aug-cc-pVDZ level of theory

Atom	x	y	z
O	3.336	-0.284	-0.001
O	1.982	-1.942	0.001
N	2.218	-0.751	0.000
C	-0.293	-0.226	0.000
C	-1.269	0.823	0.000
C	1.078	0.201	0.000
C	-0.870	2.186	0.000
C	-0.768	-1.571	0.000
C	-2.653	0.502	0.000
C	1.437	1.527	0.000
C	0.454	2.537	0.000
C	-2.117	-1.838	0.000
C	-3.076	-0.801	0.000
H	-1.648	2.951	0.000
H	-0.060	-2.389	0.000
H	-3.372	1.324	0.000

H	2.496	1.775	0.000
H	0.758	3.583	0.000
H	-2.448	-2.877	0.000
H	-4.140	-1.038	0.000

Table S40 Frequencies (cm^{-1}) of α-nitronaphtalene molecule, calculated at the M06-2X/aug-aug-cc-pVDZ level of theory.

60	100	179	218	228	367	369	412
471	482	522	535	603	650	671	748
785	799	802	820	842	896	908	963
1000	1017	1025	1030	1062	1107	1163	1175
1184	1230	1239	1284	1398	1411	1428	1475
1480	1497	1568	1653	1671	1696	1721	3206
3207	3215	3231	3234	3252	3275		

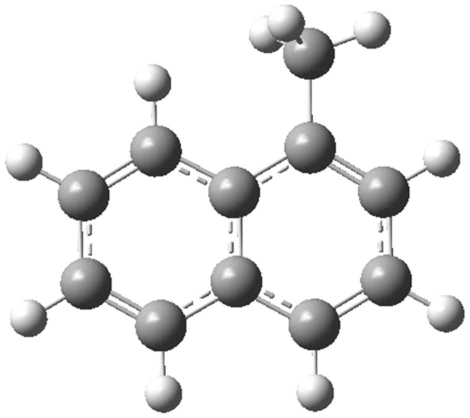

Figure S21 Visualization of the optimized structure of α-methylnaphtalene molecule, calculated at the M06-2X/aug-aug-cc-pVDZ level of theory.

Table S41 Geometry (Å) of α-methylnaphtalene molecule, calculated at the M06-2X/aug-aug-cc-pVDZ level of theory

Atom	x	y	z
C	-8.055	-0.996	-1.034
C	-7.023	-0.455	-0.304
C	-6.924	0.950	-0.104
C	-7.923	1.789	-0.679
C	-8.977	1.201	-1.428
C	-9.045	-0.160	-1.604
H	-6.270	-1.112	0.127
C	-5.859	1.537	0.653
C	-7.846	3.197	-0.492
H	-9.735	1.853	-1.863
C	-6.819	3.741	0.235
C	-5.827	2.904	0.806
H	-8.614	3.830	-0.936

**H**	-8.113	-2.074	-1.177
**H**	-9.858	-0.598	-2.181
**H**	-5.019	3.357	1.380
**H**	-6.759	4.819	0.379
**C**	-4.795	0.667	1.268
**H**	-5.230	-0.052	1.976
**H**	-4.260	0.091	0.501
**H**	-4.064	1.278	1.807

Table S42 Frequencies (cm^{-1}) of α-methylnaphtalene molecule, calculated at the M06-2X/aug-aug-cc-pVDZ level of theory.

137	175	216	257	282	425	438	480
482	517	553	574	649	719	748	796
799	817	872	886	927	988	992	1006
1020	1051	1056	1080	1101	1156	1173	1181
1234	1259	1283	1384	1396	1425	1437	1461
1466	1493	1503	1569	1664	1675	1704	3057
3120	3158	3195	3198	3203	3210	3222	3224
3233							

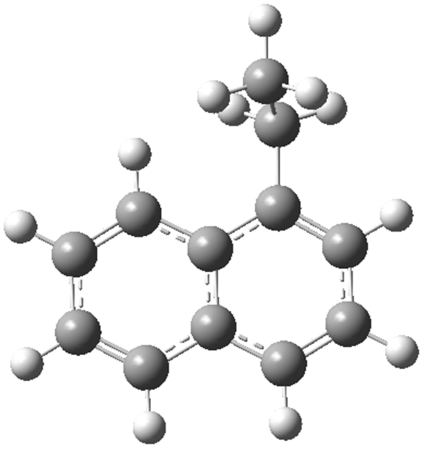

Figure S22 Visualization of the optimized structure of α-ethylnaphtalene molecule, calculated at the M06-2X/aug-aug-cc-pVDZ level of theory.

Table S43 Geometry (Å) of α-ethylnaphtalene molecule, calculated at the M06-2X/aug-aug-cc-pVDZ level of theory

Atom	x	y	z
**C**	-8.230	-1.035	-0.933
**C**	-7.163	-0.515	-0.240
**C**	-6.992	0.891	-0.098
**C**	-7.960	1.751	-0.695
**C**	-9.053	1.184	-1.406
**C**	-9.188	-0.177	-1.526
**H**	-6.439	-1.193	0.208

**C**	-5.893	1.463	0.624
**C**	-7.821	3.160	-0.567
**H**	-9.786	1.855	-1.856
**C**	-6.765	3.690	0.129
**C**	-5.805	2.834	0.724
**H**	-8.567	3.807	-1.029
**H**	-8.342	-2.114	-1.027
**H**	-10.029	-0.600	-2.073
**H**	-4.974	3.273	1.275
**H**	-6.659	4.769	0.230
**C**	-4.821	0.590	1.230
**H**	-5.276	-0.258	1.758
**H**	-4.282	1.175	1.986
**C**	-3.823	0.082	0.182
**H**	-3.061	-0.556	0.645
**H**	-4.329	-0.495	-0.601
**H**	-3.320	0.928	-0.301

Table S44 Frequencies (cm^{-1}) of α-ethylnaphtalene molecule, calculated at the M06-2X/aug-aug-cc-pVDZ level of theory.

96	112	174	192	211	302	333	440
443	479	487	518	565	597	667	712
750	782	799	804	823	867	892	932
969	988	1006	1019	1032	1056	1086	1102
1107	1158	1177	1183	1234	1254	1270	1281
1346	1385	1393	1424	1436	1468	1472	1480
1490	1504	1569	1662	1674	1705	3063	3076
3115	3144	3147	3190	3196	3201	3207	3219
3221	3232						

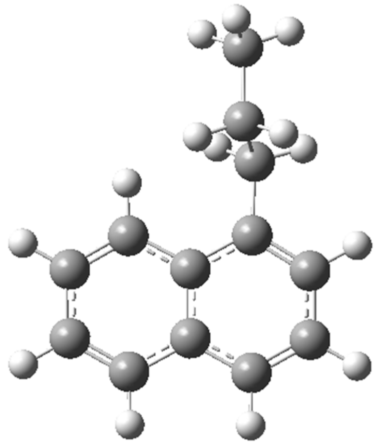

Figure S23 Visualization of the optimized structure of α-propylnaphtalene molecule, calculated at the M06-2X/aug-aug-cc-pVDZ level of theory.

Table S45 Geometry (Å) of α-propylnaphtalene molecule, calculated at the M06-2X/aug-aug-cc-pVDZ level of theory

Atom	x	y	z
C	-2.633	-1.400	0.161
C	-1.260	-1.489	0.103
C	-0.493	-0.283	-0.007
C	-1.166	0.973	-0.051
C	-2.586	1.011	0.017
C	-3.304	-0.153	0.121
H	1.467	-1.232	-0.048
H	-3.221	-2.316	0.240
C	0.929	-0.287	-0.079
C	-0.406	2.169	-0.164
H	-3.089	1.977	-0.016
H	-4.391	-0.126	0.170
C	0.964	2.131	-0.232
C	1.638	0.885	-0.189
H	-0.936	3.121	-0.198
H	1.536	3.053	-0.318
H	2.725	0.859	-0.245
C	-0.583	-2.834	0.195
H	0.224	-2.909	-0.548
H	-1.313	-3.616	-0.058
C	-0.021	-3.120	1.594
H	0.648	-2.303	1.895
H	-0.854	-3.117	2.310
C	0.719	-4.453	1.650
H	1.572	-4.453	0.959
H	1.098	-4.656	2.658
H	0.056	-5.280	1.363

Table S46 Frequencies (cm^{-1}) of α-propylnaphtalene molecule, calculated at the M06-2X/aug-aug-cc-pVDZ level of theory.

61	82	107	171	188	243	293	298
328	439	446	482	507	521	571	611
667	728	741	753	798	802	822	867
883	896	912	935	990	1006	1020	1034
1059	1072	1105	1113	1116	1158	1179	1184
1230	1245	1257	1276	1305	1310	1373	1394
1395	1427	1438	1468	1473	1477	1483	1492
1504	1570	1666	1677	1707	3056	3058	3072
3095	3113	3133	3142	3193	3197	3201	3208
3221	3222	3230					

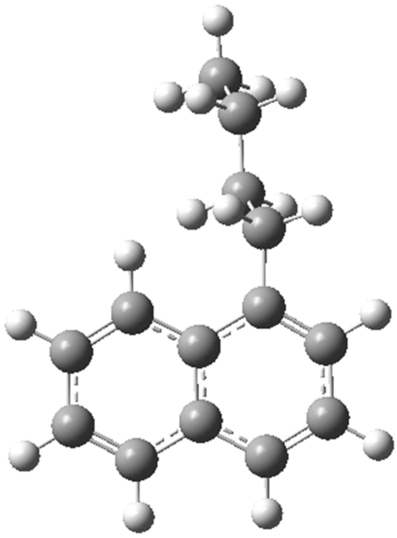

Figure S24 Visualization of the optimized structure of α-butylnaphtalene molecule, calculated at the M06-2X/aug-aug-cc-pVDZ level of theory.

Table S47 Geometry (Å) of α-butylnaphtalene molecule, calculated at the M06-2X/aug-aug-cc-pVDZ level of theory

Atom	x	y	z
C	-1.432	-1.207	-0.227
C	-0.062	-1.297	-0.119
C	0.701	-0.093	0.031
C	0.027	1.163	0.062
C	-1.389	1.203	-0.059
C	-2.103	0.041	-0.200
H	2.658	-1.043	0.136
H	-2.016	-2.121	-0.337
C	2.120	-0.098	0.157
C	0.783	2.358	0.216
H	-1.893	2.169	-0.034
H	-3.188	0.069	-0.289
C	2.150	2.318	0.334
C	2.824	1.072	0.305
H	0.253	3.310	0.239
H	2.718	3.239	0.451
H	3.909	1.045	0.401
C	0.618	-2.641	-0.201
H	1.396	-2.725	0.571
H	-0.120	-3.425	0.016
C	1.233	-2.910	-1.580
H	1.911	-2.088	-1.851
H	0.429	-2.906	-2.330
C	1.985	-4.236	-1.634
H	2.789	-4.223	-0.883
H	1.303	-5.050	-1.347

**C**	2.573	-4.519	-3.014
**H**	3.275	-3.727	-3.306
**H**	3.112	-5.474	-3.033
**H**	1.781	-4.561	-3.773

Table S48 Frequencies (cm^{-1}) of α-butylnaphtalene molecule, calculated at the M06-2X/aug-aug-cc-pVDZ level of theory.

44	64	74	132	162	183	233	244
283	314	390	442	451	481	506	520
577	607	668	720	733	750	787	801
803	823	880	891	913	926	936	988
1005	1018	1031	1044	1060	1088	1104	1114
1129	1158	1177	1183	1220	1236	1248	1262
1287	1295	1313	1347	1389	1395	1396	1425
1436	1466	1468	1476	1476	1483	1490	1503
1569	1665	1676	1705	3049	3055	3059	3067
3078	3095	3114	3132	3141	3194	3198	3202
3210	3223	3223	3232				

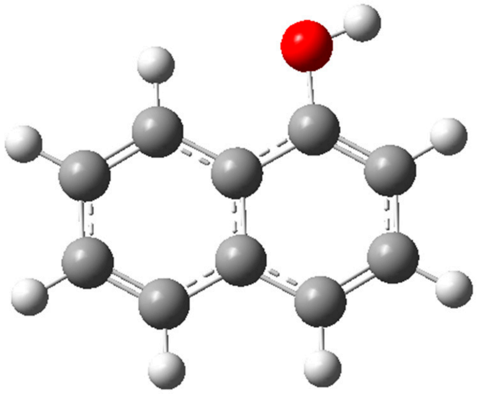

Figure S25 Visualization of the optimized structure of α-hydroxynaphtalene molecule, calculated at the M06-2X/aug-aug-cc-pVDZ level of theory.

Table S49 Geometry (Å) of α-hydroxynaphtalene molecule, calculated at the M06-2X/aug-aug-cc-pVDZ level of theory

Atom	x	y	z
**C**	2.685	-1.037	0.006
**C**	1.398	-1.519	0.002
**C**	0.299	-0.621	-0.002
**C**	0.531	0.782	-0.002
**C**	1.875	1.245	0.002
**C**	2.926	0.359	0.006
**H**	1.202	-2.589	0.002
**C**	-1.051	-1.086	-0.007
**C**	-0.572	1.681	-0.006

H	2.057	2.319	0.003
C	-1.855	1.195	-0.011
C	-2.103	-0.200	-0.011
H	-0.382	2.753	-0.006
H	3.526	-1.729	0.009
H	3.950	0.729	0.010
H	-2.700	1.881	-0.014
H	-3.128	-0.572	-0.015
O	-1.219	-2.439	-0.007
H	-2.160	-2.642	-0.009

Table S50 Frequencies (cm^{-1}) of α-hydroxynaphtalene molecule, calculated at the M06-2X/aug-aug-cc-pVDZ level of theory.

146	180	268	293	390	438	476	483
486	532	582	598	662	731	751	797
798	837	888	895	919	990	1002	1024
1048	1070	1111	1158	1169	1178	1223	1256
1268	1311	1409	1434	1446	1488	1510	1575
1667	1680	1719	3196	3202	3210	3213	3226
3227	3243	3880					

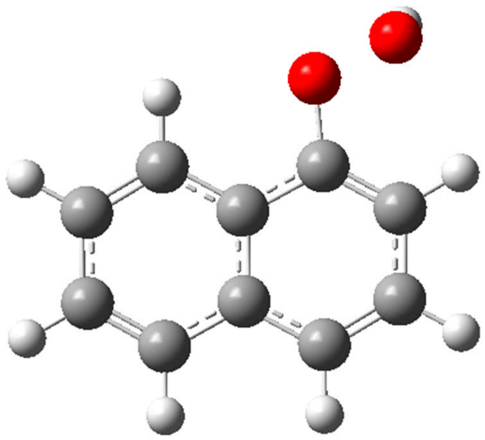

Figure S26 Visualization of the optimized structure of α-peroxynaphtalene molecule, calculated at the M06-2X/aug-aug-cc-pVDZ level of theory.

Table S51 Geometry (Å) of α-peroxynaphtalene molecule, calculated at the M06-2X/aug-aug-cc-pVDZ level of theory

Atom	x	y	z
C	2.654	-1.055	0.004
C	1.362	-1.524	0.010
C	0.271	-0.614	0.004
C	0.521	0.786	-0.006
C	1.870	1.233	-0.012
C	2.911	0.338	-0.007

**H**	1.163	-2.593	0.022
**C**	-1.089	-1.051	0.007
**C**	-0.572	1.697	-0.008
**H**	2.062	2.306	-0.019
**C**	-1.859	1.227	0.001
**C**	-2.136	-0.165	0.010
**H**	-0.368	2.767	-0.014
**H**	3.486	-1.757	0.010
**H**	3.940	0.696	-0.011
**H**	-2.695	1.924	0.004
**H**	-3.158	-0.526	0.029
**O**	-1.224	-2.422	0.012
**O**	-2.581	-2.803	0.015
**H**	-2.709	-3.072	-0.907

Table S52 Frequencies (cm^{-1}) of α-peroxynaphtalene molecule, calculated at the M06-2X/aug-aug-cc-pVDZ level of theory.

84	138	184	208	219	274	356	434
453	487	502	538	591	638	658	757
758	802	803	836	876	904	924	994
1007	1011	1026	1056	1091	1119	1160	1175
1189	1236	1255	1292	1392	1421	1436	1453
1478	1498	1566	1666	1679	1718	3201	3207
3212	3224	3225	3240	3270	3802		

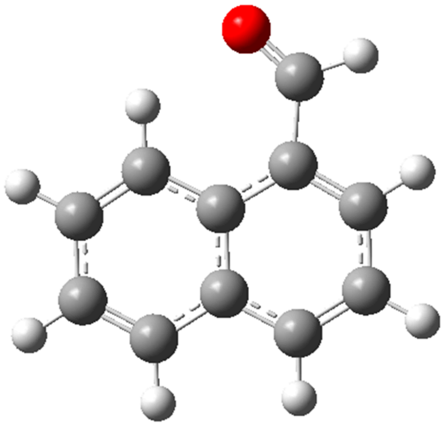

Figure S27 Visualization of the optimized structure of α-naphtaldehyde molecule, calculated at the M06-2X/aug-aug-cc-pVDZ level of theory.

Table S53 Geometry (Å) of α-naphtaldehyde molecule, calculated at the M06-2X/aug-aug-cc-pVDZ level of theory

Atom	x	y	z
**C**	2.752	-0.982	0.005
**C**	1.482	-1.513	-0.001

**C**	0.346	-0.658	-0.004
**C**	0.555	0.753	-0.002
**C**	1.880	1.266	0.004
**C**	2.959	0.417	0.007
**H**	1.335	-2.588	-0.002
**C**	-1.009	-1.136	-0.010
**C**	-0.560	1.636	-0.006
**H**	2.022	2.347	0.005
**C**	-1.845	1.156	-0.012
**C**	-2.060	-0.240	-0.014
**H**	-0.372	2.710	-0.004
**H**	3.612	-1.651	0.008
**H**	3.972	0.816	0.011
**H**	-2.694	1.838	-0.015
**H**	-3.081	-0.625	-0.019
**C**	-1.397	-2.569	-0.013
**O**	-0.657	-3.526	-0.010
**H**	-2.500	-2.723	-0.018

Table S54 Frequencies (cm^{-1}) of α-naphtaldehyde molecule, calculated at the M06-2X/aug-aug-cc-pVDZ level of theory.

84	155	187	227	278	365	422	443
487	502	550	556	655	656	728	758
799	804	833	894	910	956	1000	1018
1044	1048	1054	1076	1109	1159	1178	1188
1236	1243	1288	1387	1421	1432	1441	1482
1501	1570	1657	1670	1703	1822	2958	3194
3199	3204	3212	3225	3230	3272		

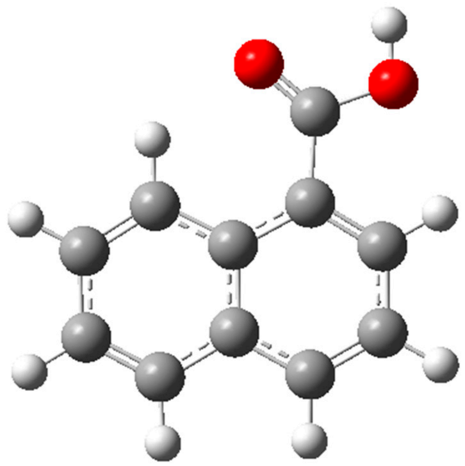

Figure S28 Visualization of the optimized structure of α-naphtalenic acid molecule, calculated at the M06-2X/aug-aug-cc-pVDZ level of theory.

Table S55 Geometry (Å) of α-naphtalenic aicd molecule, calculated at the M06-2X/aug-aug-cc-pVDZ level of theory

Atom	x	y	z
C	2.737	-0.995	0.005
C	1.468	-1.524	0.000
C	0.325	-0.673	-0.004
C	0.544	0.739	-0.002
C	1.870	1.251	0.003
C	2.949	0.404	0.006
H	1.329	-2.598	-0.001
C	-1.037	-1.138	-0.009
C	-0.560	1.633	-0.006
H	2.009	2.332	0.004
C	-1.847	1.162	-0.011
C	-2.080	-0.231	-0.013
H	-0.361	2.705	-0.005
H	3.594	-1.667	0.008
H	3.963	0.802	0.010
H	-2.691	1.848	-0.014
H	-3.102	-0.599	-0.017
C	-1.376	-2.592	-0.011
O	-0.599	-3.518	-0.006
O	-2.712	-2.817	-0.018
H	-2.816	-3.780	-0.018

Table S56 Frequencies (cm^{-1}) of α-naphtalenic acid molecule, calculated at the M06-2X/aug-aug-cc-pVDZ level of theory.

21	97	181	223	224	348	372	412
463	481	521	533	579	600	643	656
729	750	786	802	811	847	866	912
972	1003	1004	1025	1043	1060	1112	1156
1162	1183	1211	1238	1245	1284	1374	1393
1424	1431	1477	1503	1570	1656	1671	1702
1834	3201	3202	3214	3229	3229	3258	3290
3829							

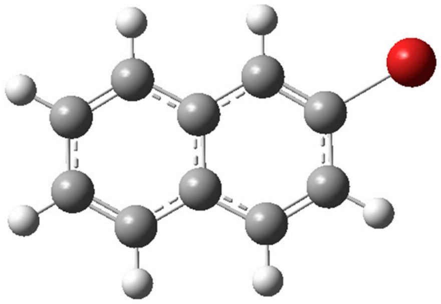

Figure S29 Visualization of the optimized structure of β-bromonaphtalene molecule, calculated at the M06-2X/aug-aug-cc-pVDZ level of theory.

Table S57 Geometry (Å) of β-bromonaphtalene molecule, calculated at the M06-2X/aug-aug-cc-pVDZ level of theory

Atom	x	y	z
C	-8.036	-0.995	-1.022
C	-7.009	-0.445	-0.294
C	-6.928	0.961	-0.105
C	-7.927	1.796	-0.681
C	-8.978	1.199	-1.429
C	-9.031	-0.163	-1.595
H	-5.113	0.923	1.084
H	-6.242	-1.080	0.149
C	-5.878	1.561	0.643
C	-7.851	3.204	-0.494
H	-9.741	1.841	-1.868
C	-6.819	3.734	0.234
C	-5.816	2.920	0.815
H	-8.611	3.848	-0.932
H	-8.090	-2.074	-1.161
H	-9.841	-0.610	-2.170
H	-5.013	3.379	1.387
Br	-6.711	5.614	0.487

Table S58 Frequencies (cm^{-1}) of β-bromonaphtalene molecule, calculated at the M06-2X/aug-aug-cc-pVDZ level of theory.

92	179	187	268	277	405	411	491
520	524	591	641	652	765	790	800
841	846	887	933	943	989	1009	1021
1050	1096	1153	1158	1171	1218	1251	1283
1376	1402	1436	1473	1490	1557	1656	1668
1706	3200	3204	3207	3218	3224	3229	3239

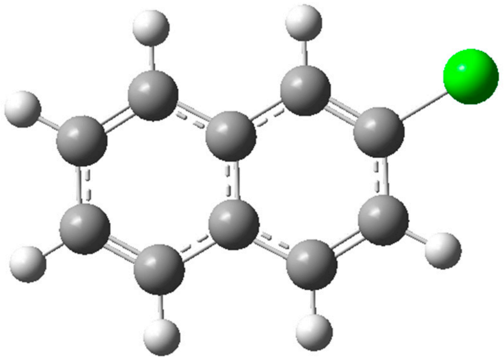

Figure S30 Visualization of the optimized structure of β-chloronaphtalene molecule, calculated at the M06-2X/aug-aug-cc-pVDZ level of theory.

Table S59 Geometry (Å) of β-chloronaphtalene molecule, calculated at the M06-2X/aug-aug-cc-pVDZ level of theory

Atom	x	y	z
C	-8.036	-0.995	-1.022
C	-7.010	-0.445	-0.294
C	-6.928	0.960	-0.105
C	-7.927	1.796	-0.681
C	-8.978	1.199	-1.429
C	-9.031	-0.163	-1.595
H	-5.113	0.921	1.084
H	-6.242	-1.080	0.149
C	-5.878	1.560	0.643
C	-7.850	3.204	-0.494
H	-9.741	1.841	-1.868
C	-6.819	3.734	0.234
C	-5.816	2.920	0.815
H	-8.609	3.852	-0.930
H	-8.090	-2.074	-1.162
H	-9.841	-0.610	-2.170
H	-5.015	3.383	1.386
Cl	-6.718	5.463	0.468

Table S60 Frequencies (cm^{-1}) of β-chloronaphtalene molecule, calculated at the M06-2X/aug-aug-cc-pVDZ level of theory.

101	187	220	277	360	412	424	491
522	531	612	654	655	765	791	800
841	863	886	931	950	988	1008	1020
1051	1108	1154	1160	1170	1220	1253	1285
1378	1406	1437	1476	1494	1559	1660	1675
1711	3200	3204	3208	3218	3224	3230	3239

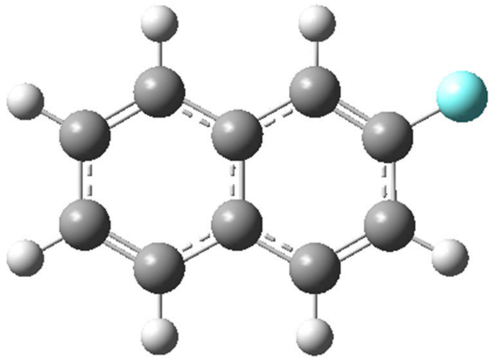

Figure S31 Visualization of the optimized structure of β-fluoronaphtalene molecule, calculated at the M06-2X/aug-aug-cc-pVDZ level of theory.

Table S61 Geometry (Å) of β-fluoronaphtalene molecule, calculated at the M06-2X/aug-aug-cc-pVDZ level of theory

Atom	x	y	z
C	-8.035	-0.997	-1.022
C	-7.010	-0.444	-0.295
C	-6.929	0.962	-0.106
C	-7.929	1.798	-0.682
C	-8.979	1.198	-1.430
C	-9.030	-0.164	-1.595
H	-5.114	0.916	1.082
H	-6.242	-1.077	0.149
C	-5.877	1.558	0.643
C	-7.853	3.205	-0.496
H	-9.743	1.838	-1.870
C	-6.819	3.719	0.233
C	-5.814	2.918	0.816
H	-8.603	3.868	-0.925
H	-8.089	-2.075	-1.161
H	-9.840	-0.612	-2.170
H	-5.019	3.395	1.384
F	-6.734	5.054	0.418

Table S62 Frequencies (cm^{-1}) of β-fluoronaphtalene molecule, calculated at the M06-2X/aug-aug-cc-pVDZ level of theory.

128	187	293	312	417	426	467	492
528	550	628	661	719	771	791	797
839	886	908	924	983	987	999	1020
1052	1132	1150	1162	1175	1241	1274	1285
1381	1414	1442	1481	1512	1572	1666	1686
1719	3198	3201	3206	3216	3224	3229	3236

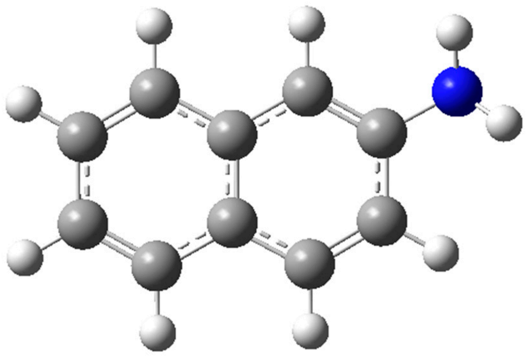

Figure S32 Visualization of the optimized structure of β-aminonaphtalene molecule, calculated at the M06-2X/aug-aug-cc-pVDZ level of theory.

Table S63 Geometry (Å) of β-aminonaphtalene molecule, calculated at the M06-2X/aug-aug-cc-pVDZ level of theory

Atom	x	y	z
C	-7.958	-0.973	-1.075
C	-6.947	-0.397	-0.345
C	-6.924	1.006	-0.111
C	-7.974	1.802	-0.652
C	-9.007	1.180	-1.402
C	-9.003	-0.178	-1.612
H	-6.146	-1.011	0.067
C	-5.889	1.626	0.636
C	-7.949	3.204	-0.416
H	-9.804	1.801	-1.810
C	-6.944	3.781	0.313
C	-5.888	2.989	0.852
H	-8.749	3.821	-0.825
H	-7.962	-2.049	-1.244
H	-9.798	-0.648	-2.188
H	-6.943	4.856	0.495
H	-5.090	1.008	1.045
N	-4.917	3.612	1.639
H	-4.055	3.092	1.743
H	-4.747	4.583	1.415

Table S64 Frequencies (cm^{-1}) of β-aminonaphtalene molecule, calculated at the M06-2X/aug-aug-cc-pVDZ level of theory.

123	186	284	290	300	414	425	466
488	528	537	584	630	671	732	767
792	799	836	867	909	915	975	979
1000	1014	1051	1110	1151	1155	1169	1200
1246	1285	1310	1397	1418	1444	1489	1515
1571	1634	1662	1686	1716	3187	3191	3193
3196	3209	3213	3228	3587	3695		

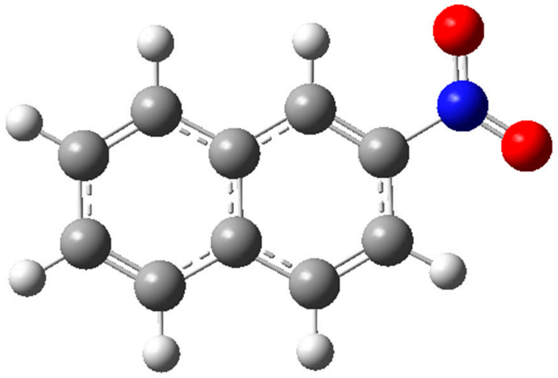

Figure S33 Visualization of the optimized structure of β-nitronaphtalene molecule, calculated at the M06-2X/aug-aug-cc-pVDZ level of theory.

Table S65 Geometry (Å) of β-nitronaphtalene molecule, calculated at the M06-2X/aug-aug-cc-pVDZ level of theory

Atom	x	y	z
C	-8.039	-0.985	-1.028
C	-7.037	-0.447	-0.257
C	-6.943	0.958	-0.074
C	-7.905	1.801	-0.702
C	-8.931	1.216	-1.494
C	-8.996	-0.145	-1.653
H	-5.185	0.894	1.197
H	-6.302	-1.091	0.223
C	-5.916	1.544	0.719
C	-7.816	3.205	-0.523
H	-9.662	1.868	-1.971
C	-6.807	3.714	0.251
C	-5.839	2.903	0.886
H	-8.535	3.875	-0.989
N	-6.725	5.175	0.431
O	-5.820	5.603	1.122
O	-7.561	5.864	-0.120
H	-8.103	-2.064	-1.163
H	-9.784	-0.587	-2.261
H	-5.065	3.370	1.487

Table S66 Frequencies (cm^{-1}) of β-nitronaphtalene molecule, calculated at the M06-2X/aug-aug-cc-pVDZ level of theory.

53	96	192	196	261	344	382	411
488	513	522	548	611	639	648	753
791	797	810	826	851	909	911	958
974	997	1017	1025	1051	1116	1151	1165
1167	1232	1250	1285	1381	1414	1448	1470
1482	1496	1568	1662	1675	1704	1724	3206
3210	3216	3222	3233	3243	3261		

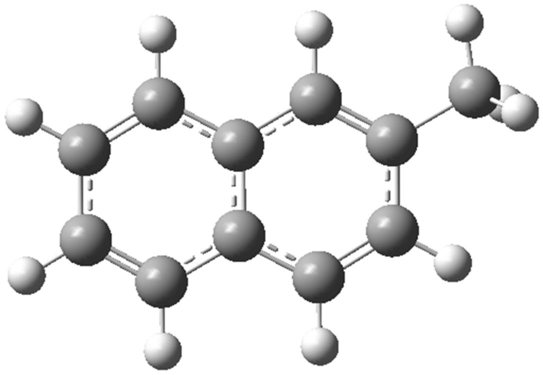

Figure S34 Visualization of the optimized structure of β-methylnaphtalene molecule, calculated at the M06-2X/aug-aug-cc-pVDZ level of theory.

Table S67 Geometry (Å) of β-methylnaphtalene molecule, calculated at the M06-2X/aug-aug-cc-pVDZ level of theory

Atom	x	y	z
C	-8.037	-1.004	-1.021
C	-6.993	-0.448	-0.323
C	-6.918	0.957	-0.127
C	-7.940	1.788	-0.665
C	-9.008	1.185	-1.382
C	-9.056	-0.177	-1.557
H	-5.066	0.933	1.003
H	-6.206	-1.078	0.092
C	-5.851	1.567	0.590
C	-7.863	3.196	-0.468
H	-9.791	1.823	-1.794
C	-6.826	3.770	0.228
C	-5.809	2.926	0.761
H	-8.653	3.823	-0.884
H	-8.087	-2.082	-1.166
H	-9.880	-0.627	-2.109
H	-4.985	3.379	1.313
C	-6.737	5.258	0.441
H	-5.809	5.657	0.010
H	-7.583	5.776	-0.021
H	-6.729	5.497	1.513

Table S68 Frequencies (cm^{-1}) of β-methylnaphtalene molecule, calculated at the M06-2X/aug-aug-cc-pVDZ level of theory.

111	134	188	267	287	410	416	453
493	525	526	629	653	712	760	793
805	841	880	895	932	965	985	1008
1019	1020	1051	1056	1149	1154	1169	1192
1238	1265	1284	1382	1395	1413	1438	1463

1464	1486	1512	1564	1660	1684	1718	3059
3124	3156	3185	3190	3194	3197	3207	3215
3228							

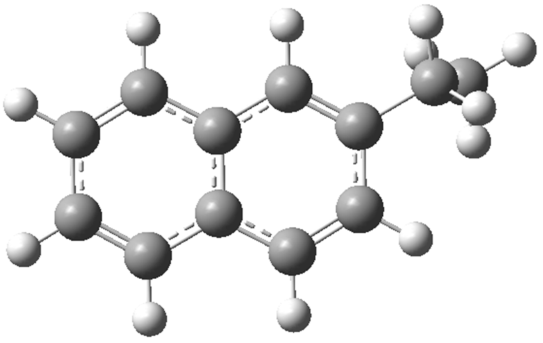

Figure S35 Visualization of the optimized structure of β-ethylnaphtalene molecule, calculated at the M06-2X/aug-aug-cc-pVDZ level of theory.

Table S69 Geometry (Å) of β-ethylnaphtalene molecule, calculated at the M06-2X/aug-aug-cc-pVDZ level of theory

Atom	x	y	z
C	-8.030	-1.020	-1.013
C	-6.982	-0.442	-0.340
C	-6.929	0.966	-0.152
C	-7.980	1.774	-0.669
C	-9.052	1.148	-1.361
C	-9.077	-0.215	-1.530
H	-5.058	0.981	0.945
H	-6.174	-1.055	0.061
C	-5.860	1.598	0.542
C	-7.926	3.183	-0.478
H	-9.856	1.769	-1.757
C	-6.885	3.781	0.193
C	-5.840	2.959	0.705
H	-8.738	3.794	-0.876
H	-8.061	-2.100	-1.153
H	-9.903	-0.684	-2.062
H	-5.016	3.429	1.243
C	-6.802	5.277	0.359
H	-7.788	5.719	0.171
H	-6.534	5.513	1.399
C	-5.769	5.899	-0.587
H	-6.037	5.694	-1.631
H	-5.714	6.985	-0.447
H	-4.773	5.478	-0.407

Table S70 Frequencies (cm⁻¹) of β-ethylnaphtalene molecule, calculated at the M06-2X/aug-aug-cc-pVDZ level of theory.

46	90	186	199	210	292	344	410
418	470	497	524	560	628	672	709
767	780	794	809	844	884	887	933
954	986	998	1005	1018	1051	1076	1090
1151	1154	1170	1190	1237	1255	1275	1286
1343	1382	1389	1413	1440	1465	1472	1478
1483	1512	1563	1659	1684	1717	3061	3066
3112	3143	3145	3186	3190	3197	3200	3209
3215	3227						

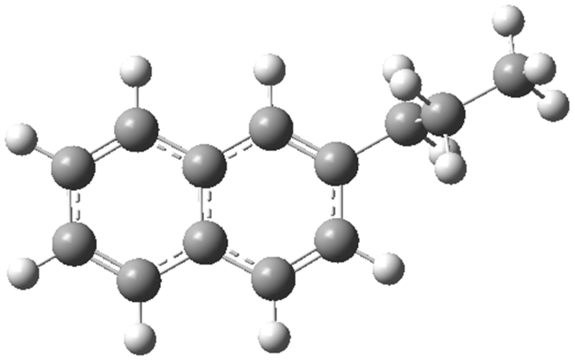

Figure S36 Visualization of the optimized structure of β-propylnaphtalene molecule, calculated at the M06-2X/aug-aug-cc-pVDZ level of theory.

Table S71 Geometry (Å) of β-propylnaphtalene molecule, calculated at the M06-2X/aug-aug-cc-pVDZ level of theory

Atom	x	y	z
C	-1.680	-2.596	-0.056
C	-0.305	-2.556	-0.034
C	0.405	-1.323	-0.032
C	-0.327	-0.103	-0.052
C	-1.748	-0.160	-0.076
C	-2.399	-1.367	-0.077
H	2.385	-2.208	0.005
H	0.265	-3.486	-0.021
C	1.826	-1.272	-0.010
C	0.381	1.129	-0.051
H	-2.311	0.773	-0.098
H	-3.490	-1.395	-0.100
C	1.754	1.149	-0.030
C	2.484	-0.066	-0.009
H	-0.187	2.060	-0.068
H	2.288	2.098	-0.028
H	3.573	-0.039	0.008
C	-2.436	-3.898	-0.009

**H**	-3.267	-3.868	-0.729
**H**	-1.773	-4.720	-0.314
**C**	-2.996	-4.194	1.387
**H**	-3.636	-3.357	1.701
**H**	-2.160	-4.231	2.100
**C**	-3.784	-5.499	1.427
**H**	-4.172	-5.700	2.432
**H**	-4.635	-5.461	0.735
**H**	-3.150	-6.346	1.133

Table S72 Frequencies (cm^{-1}) of β-propylnaphtalene molecule, calculated at the M06-2X/aug-aug-cc-pVDZ level of theory.

43	73	82	173	189	242	271	294
332	413	419	492	505	524	562	632
672	732	740	770	792	810	844	863
878	899	920	933	974	986	1005	1017
1051	1072	1092	1117	1151	1155	1171	1192
1230	1245	1266	1284	1298	1310	1371	1388
1393	1414	1441	1465	1471	1476	1479	1486
1511	1564	1659	1683	1716	3051	3058	3065
3091	3112	3134	3142	3182	3187	3194	3197
3206	3214	3227					

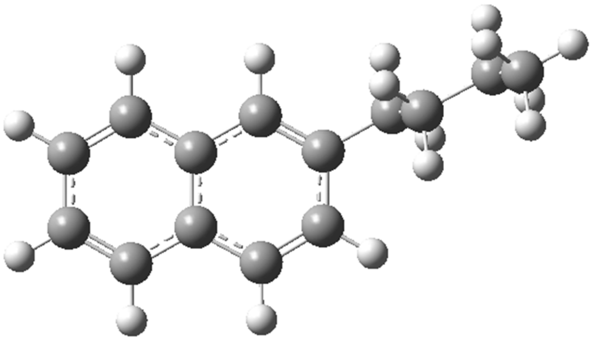

Figure S37 Visualization of the optimized structure of β-butylnaphtalene molecule, calculated at the M06-2X/aug-aug-cc-pVDZ level of theory.

Table S73 Geometry (Å) of β-butylnaphtalene molecule, calculated at the M06-2X/aug-aug-cc-pVDZ level of theory

Atom	x	y	z
**C**	0.401	-2.000	-0.031
**C**	1.775	-1.960	0.007
**C**	2.485	-0.727	0.009
**C**	1.753	0.493	-0.030
**C**	0.333	0.435	-0.071
**C**	-0.319	-0.771	-0.070

**H**	4.464	-1.611	0.075
**H**	2.345	-2.890	0.035
**C**	3.905	-0.675	0.046
**C**	2.460	1.725	-0.031
**H**	-0.230	1.368	-0.107
**H**	-1.409	-0.800	-0.106
**C**	3.833	1.746	0.007
**C**	4.563	0.530	0.046
**H**	1.893	2.656	-0.062
**H**	4.367	2.695	0.007
**H**	5.652	0.558	0.076
**C**	-0.356	-3.302	0.016
**H**	-1.180	-3.276	-0.712
**H**	0.309	-4.126	-0.276
**C**	-0.930	-3.589	1.408
**H**	-1.575	-2.752	1.715
**H**	-0.103	-3.624	2.133
**C**	-1.721	-4.891	1.461
**H**	-2.542	-4.842	0.732
**H**	-1.071	-5.720	1.144
**C**	-2.282	-5.179	2.851
**H**	-2.955	-4.373	3.173
**H**	-2.846	-6.119	2.870
**H**	-1.473	-5.254	3.589

Table S74 Frequencies (cm^{-1}) of β-butylnaphtalene molecule, calculated at the M06-2X/aug-aug-cc-pVDZ level of theory.

28	57	74	118	145	185	237	242
272	301	392	417	430	493	504	524
561	632	672	721	739	768	784	793
809	843	882	896	914	927	936	972
984	1002	1016	1045	1050	1089	1098	1128
1150	1154	1170	1191	1219	1240	1252	1270
1285	1298	1312	1345	1386	1389	1395	1413
1439	1462	1467	1475	1476	1478	1487	1510
1564	1658	1682	1716	3045	3052	3058	3060
3076	3094	3112	3133	3142	3184	3190	3196
3200	3209	3215	3227				

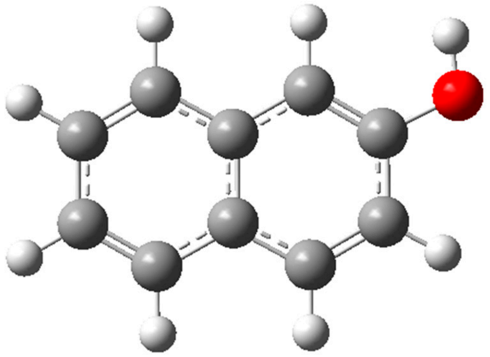

Figure S38 Visualization of the optimized structure of β-hydroxynaphtalene molecule, calculated at the M06-2X/aug-aug-cc-pVDZ level of theory.

Table S75 Geometry (Å) of β-hydroxynaphtalene molecule, calculated at the M06-2X/aug-aug-cc-pVDZ level of theory

Atom	x	y	z
C	-2.928	0.406	0.000
C	-1.858	1.267	0.000
C	-0.527	0.772	0.000
C	-0.306	-0.635	0.000
C	-1.433	-1.503	0.000
C	-2.710	-0.995	0.000
H	0.436	2.716	0.000
H	-2.014	2.345	0.000
C	0.601	1.639	0.000
C	1.025	-1.133	0.000
H	-1.268	-2.580	0.000
C	2.088	-0.262	0.000
C	1.878	1.142	0.000
H	1.192	-2.211	0.000
H	-3.563	-1.672	0.000
H	2.748	1.796	0.000
O	3.391	-0.665	0.000
H	3.430	-1.628	0.001
H	-3.945	0.794	0.000

Table S76 Frequencies (cm^{-1}) of β-hydroxynaphtalene molecule, calculated at the M06-2X/aug-aug-cc-pVDZ level of theory.

126	187	295	307	395	418	432	471
490	529	556	630	671	729	769	793
800	841	876	912	923	983	984	1008
1018	1052	1140	1152	1162	1188	1213	1244
1287	1314	1398	1417	1447	1494	1514	1580
1669	1685	1716	3182	3195	3198	3201	3215
3228	3230	3876					

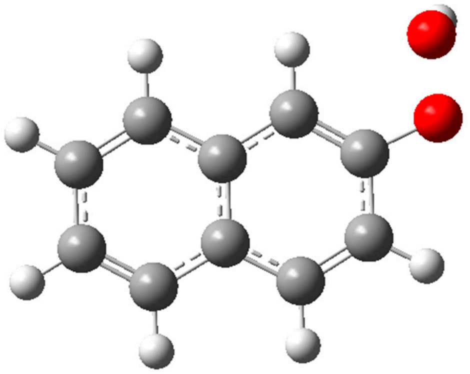

Figure S39 Visualization of the optimized structure of β-peroxynaphtalene molecule, calculated at the M06-2X/aug-aug-cc-pVDZ level of theory.

Table S77 Geometry (Å) of β-peroxynaphtalene molecule, calculated at the M06-2X/aug-aug-cc-pVDZ level of theory

Atom	x	y	z
C	3.321	0.009	-0.016
C	2.427	1.052	-0.020
C	1.029	0.809	-0.006
C	0.553	-0.531	0.011
C	1.500	-1.591	0.016
C	2.849	-1.327	0.003
H	0.439	2.900	-0.017
H	2.780	2.084	-0.033
C	0.079	1.871	-0.007
C	-0.848	-0.788	0.023
H	1.138	-2.619	0.031
C	-1.718	0.270	0.019
C	-1.265	1.616	0.006
H	-1.214	-1.810	0.049
H	4.392	0.203	-0.026
H	3.563	-2.149	0.007
H	-2.001	2.418	0.009
O	-3.091	0.168	0.028
O	-3.502	-1.181	0.049
H	-3.793	-1.310	-0.867

Table S78 Frequencies (cm^{-1}) of β-peroxynaphtalene molecule, calculated at the M06-2X/aug-aug-cc-pVDZ level of theory.

83	134	189	195	207	309	352	416
441	494	520	551	557	635	664	726
769	792	803	840	881	918	924	984
984	1004	1017	1050	1054	1137	1152	1164
1182	1237	1268	1282	1384	1412	1434	1449
1481	1507	1567	1668	1683	1713	3198	3202

3207	3216	3228	3232	3256	3805		

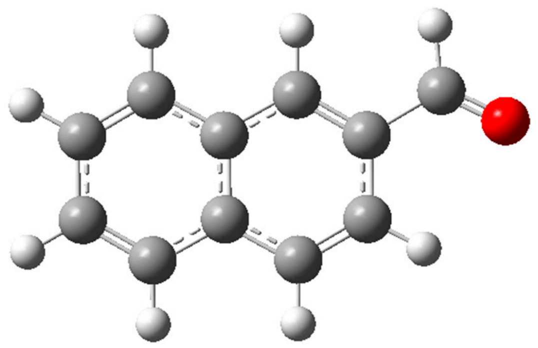

Figure S40 Visualization of the optimized structure of β-naphtaldehyde molecule, calculated at the M06-2X/aug-aug-cc-pVDZ level of theory.

Table S79 Geometry (Å) of β-naphtaldehyde molecule, calculated at the M06-2X/aug-aug-cc-pVDZ level of theory

Atom	x	y	z
C	-2.909	0.394	0.000
C	-1.846	1.264	0.000
C	-0.512	0.777	0.000
C	-0.294	-0.631	0.000
C	-1.413	-1.508	0.000
C	-2.692	-1.007	0.000
H	0.430	2.732	0.000
H	-2.012	2.341	0.000
C	0.611	1.657	0.000
C	1.041	-1.113	0.000
H	-1.238	-2.583	0.000
C	2.105	-0.240	0.001
C	1.888	1.166	0.000
H	1.219	-2.190	0.001
H	-3.544	-1.684	0.000
H	2.756	1.823	0.001
H	-3.928	0.778	0.000
C	3.486	-0.778	0.001
O	4.481	-0.093	0.001
H	3.560	-1.889	0.000

Table S80 Frequencies (cm^{-1}) of β-naphtaldehyde molecule, calculated at the M06-2X/aug-aug-cc-pVDZ level of theory.

83	164	184	198	304	355	398	410
496	521	527	613	639	660	767	770
797	812	852	892	897	946	973	992
1021	1025	1043	1052	1141	1156	1162	1183

1240	1275	1284	1374	1402	1415	1450	1480
1506	1566	1662	1679	1712	1828	2961	3182
3198	3201	3202	3218	3230	3230		

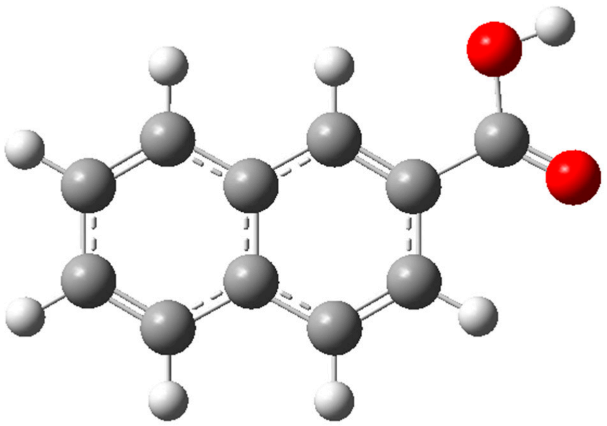

Figure S41 Visualization of the optimized structure of β-naphtalenic acid molecule, calculated at the M06-2X/aug-aug-cc-pVDZ level of theory.

Table S81 Geometry (Å) of β-naphtalenic aicd molecule, calculated at the M06-2X/aug-aug-cc-pVDZ level of theory

Atom	x	y	z
C	-2.909	0.415	0.000
C	-1.834	1.270	0.000
C	-0.507	0.763	0.000
C	-0.307	-0.647	0.000
C	-1.439	-1.508	0.000
C	-2.711	-0.990	0.000
H	0.469	2.701	0.000
H	-1.985	2.349	-0.001
C	0.628	1.623	0.000
C	1.017	-1.157	0.001
H	-1.279	-2.586	0.001
C	2.093	-0.298	0.001
C	1.897	1.109	0.001
H	1.177	-2.234	0.001
H	-3.573	-1.655	0.000
H	2.774	1.754	0.001
H	-3.923	0.813	-0.001
C	3.494	-0.800	0.001
O	4.474	-0.095	0.001
O	3.579	-2.149	0.002
H	4.524	-2.362	0.002

Table S82 Frequencies (cm^{-1}) of β-naphtalenic acid molecule, calculated at the M06-2X/aug-aug-cc-pVDZ level of theory.

63	92	166	194	252	334	360	410
490	507	512	520	597	602	637	658
721	753	792	799	819	858	888	908
960	965	995	1020	1027	1050	1117	1151
1158	1166	1204	1240	1261	1286	1378	1393
1418	1444	1478	1509	1566	1660	1681	1712
1845	3202	3206	3209	3219	3228	3230	3237
3823							

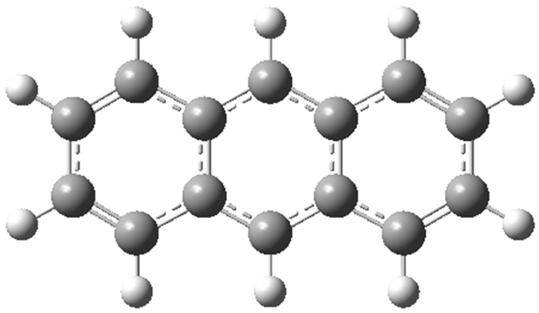

Figure S42 Visualization of the optimized structure of antracene molecule, calculated at the M06-2X/aug-aug-cc-pVDZ level of theory.

Table S83 Geometry (Å) of antracene molecule, calculated at the M06-2X/aug-aug-cc-pVDZ level of theory

Atom	x	y	z
C	-5.903	5.957	-2.866
C	-4.555	5.954	-2.647
C	-3.893	4.781	-2.157
C	-4.676	3.604	-1.899
C	-6.087	3.649	-2.142
C	-6.681	4.786	-2.609
C	-2.515	4.751	-1.924
C	-4.042	2.453	-1.421
C	-2.664	2.422	-1.188
C	-1.881	3.600	-1.446
C	-0.470	3.555	-1.204
H	0.121	4.449	-1.400
C	0.124	2.418	-0.736
C	-0.654	1.246	-0.480
C	-2.002	1.249	-0.699
H	-1.920	5.645	-2.120
H	-6.394	6.855	-3.238
H	-3.959	6.846	-2.842
H	-6.678	2.754	-1.946
H	-7.755	4.808	-2.790

H	-4.637	1.559	-1.225
H	-2.598	0.358	-0.504
H	-0.163	0.349	-0.107
H	1.198	2.396	-0.556

Table S84 Frequencies (cm^{-1}) of antracene molecule, calculated at the M06-2X/aug-aug-cc-pVDZ level of theory.

89	122	240	243	274	395	399	400
486	496	516	530	601	611	634	656
747	778	778	779	799	834	857	883
906	917	920	942	991	996	1013	1014
1033	1040	1137	1138	1170	1174	1183	1201
1282	1288	1301	1349	1369	1403	1452	1480
1487	1493	1528	1616	1645	1658	1714	1714
3194	3195	3199	3199	3203	3204	3217	3217
3228	3228						

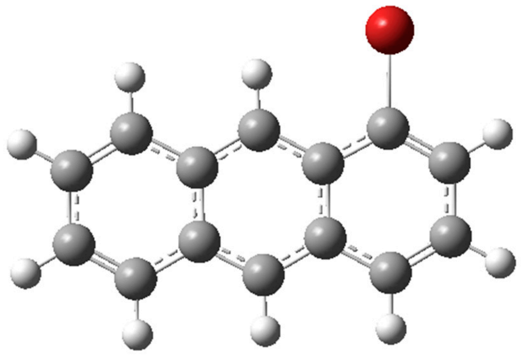

Figure S43 Visualization of the optimized structure of α-bromoantracene molecule, calculated at the M06-2X/aug-aug-cc-pVDZ level of theory.

Table S85 Geometry (Å) of α-bromoantracene molecule, calculated at the M06-2X/aug-aug-cc-pVDZ level of theory

Atom	x	y	z
C	3.823	1.220	-0.089
C	2.513	1.588	-0.202
C	1.469	0.610	-0.128
C	1.826	-0.766	0.068
C	3.211	-1.113	0.181
C	4.178	-0.152	0.105
C	0.121	0.966	-0.241
C	0.812	-1.725	0.141
C	-0.538	-1.376	0.029
C	-0.893	0.006	-0.167
C	-2.294	0.310	-0.276
C	-3.254	-0.656	-0.199
C	-2.880	-2.020	-0.004

**C**	-1.567	-2.369	0.106
**H**	-0.137	2.013	-0.389
**H**	4.609	1.971	-0.147
**H**	2.239	2.632	-0.350
**H**	3.477	-2.159	0.330
**H**	5.228	-0.424	0.193
**H**	1.076	-2.773	0.290
**H**	-1.276	-3.408	0.255
**H**	-3.662	-2.775	0.054
**H**	-4.304	-0.387	-0.286
**Br**	-2.851	2.110	-0.537

Table S86 Frequencies (cm^{-1}) of α-bromoantracene molecule, calculated at the M06-2X/aug-aug-cc-pVDZ level of theory.

77	92	130	182	252	264	287	322
410	411	439	490	512	539	566	600
621	651	677	756	775	795	796	822
858	878	916	918	933	936	949	1000
1011	1021	1038	1075	1140	1149	1172	1185
1192	1253	1293	1299	1344	1369	1390	1443
1460	1480	1492	1521	1607	1638	1658	1704
1714	3193	3199	3203	3206	3217	3225	3227
3228	3239						

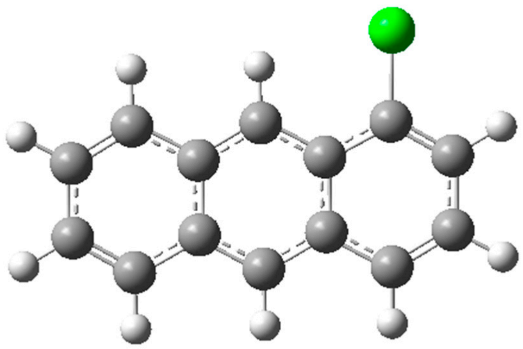

Figure S44 Visualization of the optimized structure of α-chloroantracene molecule, calculated at the M06-2X/aug-aug-cc-pVDZ level of theory.

Table S87 Geometry (Å) of α-chloroantracene molecule, calculated at the M06-2X/aug-aug-cc-pVDZ level of theory

Atom	x	y	z
**C**	3.821	1.222	-0.090
**C**	2.510	1.588	-0.203
**C**	1.467	0.609	-0.128
**C**	1.825	-0.767	0.068
**C**	3.211	-1.111	0.182

**C**	4.176	-0.149	0.105
**C**	0.118	0.964	-0.241
**C**	0.813	-1.728	0.142
**C**	-0.537	-1.381	0.030
**C**	-0.891	0.000	-0.167
**C**	-2.291	0.307	-0.277
**C**	-3.254	-0.655	-0.200
**C**	-2.882	-2.019	-0.004
**C**	-1.569	-2.372	0.107
**H**	-0.145	2.009	-0.390
**H**	4.606	1.974	-0.148
**H**	2.235	2.632	-0.352
**H**	3.478	-2.157	0.330
**H**	5.227	-0.420	0.193
**H**	1.080	-2.775	0.291
**H**	-1.282	-3.412	0.255
**H**	-3.664	-2.774	0.054
**H**	-4.302	-0.378	-0.288
**Cl**	-2.781	1.968	-0.516

Table S88 Frequencies (cm^{-1}) of α-chloroantracene molecule, calculated at the M06-2X/aug-aug-cc-pVDZ level of theory.

82	96	165	189	254	289	294	378
411	433	454	488	512	540	572	602
623	652	694	751	773	791	800	820
861	872	912	916	930	944	955	995
1003	1016	1036	1076	1144	1150	1174	1184
1191	1253	1290	1299	1346	1366	1393	1444
1464	1481	1495	1522	1611	1642	1658	1708
1714	3196	3201	3205	3209	3219	3227	3228
3231	3240						

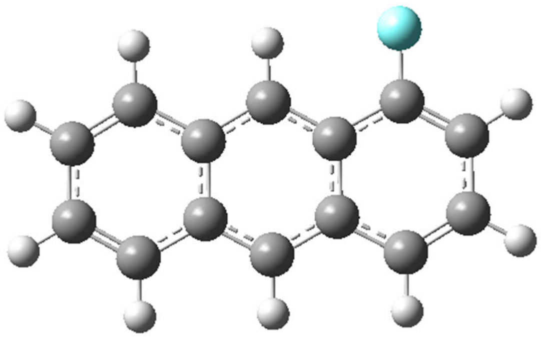

Figure S45 Visualization of the optimized structure of α-fluoroantracene molecule, calculated at the M06-2X/aug-aug-cc-pVDZ level of theory.

Table S89 Geometry (Å) of α-fluoroantracene molecule, calculated at the M06-2X/aug-aug-cc-pVDZ level of theory

Atom	x	y	z
C	3.813	1.229	-0.092
C	2.502	1.591	-0.205
C	1.462	0.609	-0.129
C	1.823	-0.768	0.068
C	3.210	-1.105	0.182
C	4.173	-0.141	0.105
C	0.111	0.957	-0.243
C	0.818	-1.738	0.144
C	-0.534	-1.400	0.032
C	-0.883	-0.021	-0.165
C	-2.272	0.289	-0.275
C	-3.255	-0.646	-0.203
C	-2.887	-2.014	-0.006
C	-1.577	-2.380	0.108
H	-0.170	1.998	-0.392
H	4.596	1.984	-0.151
H	2.223	2.634	-0.355
H	3.481	-2.150	0.332
H	5.225	-0.409	0.193
H	1.095	-2.783	0.295
H	-1.304	-3.424	0.257
H	-3.674	-2.764	0.052
H	-4.295	-0.345	-0.294
F	-2.593	1.586	-0.461

Table S90 Frequencies (cm^{-1}) of α-fluoroantracene molecule, calculated at the M06-2X/aug-aug-cc-pVDZ level of theory.

93	101	194	214	260	301	324	393
417	487	490	512	524	548	587	619
624	650	744	753	773	793	811	826
872	876	908	914	931	943	998	1003
1020	1037	1045	1079	1148	1162	1175	1184
1225	1269	1287	1300	1353	1374	1410	1448
1480	1482	1502	1528	1620	1648	1662	1711
1726	3197	3199	3204	3209	3217	3222	3227
3228	3240						

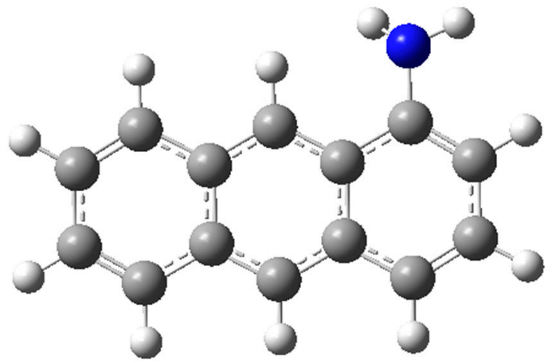

Figure S46 Visualization of the optimized structure of α-aminoantracene molecule, calculated at the M06-2X/aug-aug-cc-pVDZ level of theory.

Table S91 Geometry (Å) of α-aminoantracene molecule, calculated at the M06-2X/aug-aug-cc-pVDZ level of theory

Atom	x	y	z
C	3.820	1.221	-0.110
C	2.508	1.586	-0.218
C	1.466	0.609	-0.120
C	1.824	-0.765	0.084
C	3.210	-1.107	0.194
C	4.177	-0.147	0.101
C	0.113	0.956	-0.224
C	0.812	-1.727	0.156
C	-0.540	-1.386	0.047
C	-0.898	-0.004	-0.129
C	-2.299	0.345	-0.213
C	-3.247	-0.647	-0.170
C	-2.877	-2.015	-0.027
C	-1.568	-2.382	0.092
H	-0.133	2.000	-0.413
H	4.605	1.973	-0.183
H	2.233	2.628	-0.379
H	3.478	-2.152	0.351
H	5.228	-0.418	0.185
H	1.082	-2.775	0.295
H	-1.285	-3.425	0.217
H	-3.661	-2.770	0.002
H	-4.303	-0.382	-0.235
N	-2.648	1.687	-0.399
H	-3.637	1.862	-0.274
H	-2.096	2.342	0.140

Table S92 Frequencies (cm^{-1}) of α-aminoantracene molecule, calculated at the M06-2X/aug-aug-cc-pVDZ level of theory.

92	98	197	208	260	294	322	352

394	416	469	487	512	528	553	581
604	625	649	678	746	748	772	789
816	823	868	873	889	911	918	938
991	994	1015	1034	1039	1099	1132	1153
1167	1182	1187	1236	1286	1294	1316	1354
1381	1422	1453	1475	1483	1500	1530	1614
1637	1654	1661	1708	1712	3194	3194	3197
3201	3207	3209	3215	3224	3227	3577	3679

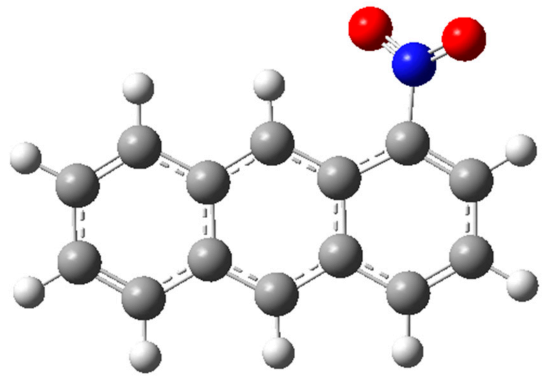

Figure S47 Visualization of the optimized structure of α-nitroantracene molecule, calculated at the M06-2X/aug-aug-cc-pVDZ level of theory.

Table S93 Geometry (Å) of α-nitroantracene molecule, calculated at the M06-2X/aug-aug-cc-pVDZ level of theory

Atom	x	y	z
C	3.849	1.194	-0.138
C	2.542	1.576	-0.239
C	1.489	0.610	-0.136
C	1.835	-0.765	0.075
C	3.217	-1.127	0.177
C	4.192	-0.178	0.074
C	0.145	0.991	-0.234
C	0.810	-1.709	0.166
C	-0.535	-1.340	0.061
C	-0.884	0.046	-0.134
C	-2.292	0.322	-0.257
C	-3.249	-0.651	-0.211
C	-2.878	-2.007	0.000
C	-1.561	-2.335	0.135
H	-0.088	2.040	-0.381
H	4.642	1.936	-0.217
H	2.277	2.621	-0.398
H	3.472	-2.174	0.338
H	5.241	-0.460	0.152
H	1.059	-2.760	0.316
H	-1.261	-3.370	0.297

**H**	-3.653	-2.769	0.054
**H**	-4.292	-0.368	-0.328
**N**	-2.794	1.698	-0.426
**O**	-3.836	1.845	-1.036
**O**	-2.158	2.607	0.074

Table S94 Frequencies (cm^{-1}) of α-nitroantracene molecule, calculated at the M06-2X/aug-aug-cc-pVDZ level of theory.

50	70	97	162	173	248	278	283
358	372	403	426	486	498	513	538
579	604	626	661	685	752	763	778
792	798	837	845	875	883	916	923
942	964	998	1008	1012	1018	1036	1094
1149	1153	1178	1186	1193	1256	1295	1300
1347	1371	1398	1442	1466	1478	1484	1493
1524	1611	1640	1654	1683	1712	1722	3198
3201	3207	3208	3219	3230	3235	3251	3266

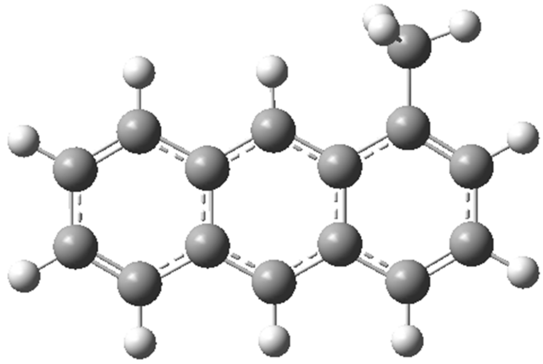

Figure S48 Visualization of the optimized structure of α-methylantracene molecule, calculated at the M06-2X/aug-aug-cc-pVDZ level of theory.

Table S95 Geometry (Å) of α-methylantracene molecule, calculated at the M06-2X/aug-aug-cc-pVDZ level of theory

Atom	x	y	z
**C**	3.811	1.227	-0.087
**C**	2.498	1.589	-0.195
**C**	1.458	0.607	-0.122
**C**	1.821	-0.768	0.067
**C**	3.208	-1.108	0.175
**C**	4.172	-0.143	0.100
**C**	0.105	0.953	-0.229
**C**	0.810	-1.732	0.139
**C**	-0.540	-1.387	0.032
**C**	-0.907	-0.010	-0.158
**C**	-2.305	0.339	-0.267

**C**	-3.243	-0.653	-0.188
**C**	-2.881	-2.022	0.000
**C**	-1.570	-2.381	0.107
**H**	-0.150	2.002	-0.372
**H**	4.594	1.982	-0.144
**H**	2.220	2.633	-0.338
**H**	3.478	-2.154	0.318
**H**	5.224	-0.412	0.184
**H**	1.081	-2.779	0.282
**H**	-1.283	-3.422	0.250
**H**	-3.664	-2.777	0.057
**H**	-4.299	-0.395	-0.270
**C**	-2.706	1.776	-0.465
**H**	-2.356	2.402	0.366
**H**	-2.273	2.183	-1.389
**H**	-3.795	1.864	-0.528

Table S96 Frequencies (cm^{-1}) of α-methylantracene molecule, calculated at the M06-2X/aug-aug-cc-pVDZ level of theory.

93	96	200	201	233	257	295	326
395	411	448	490	515	522	550	563
602	625	652	736	756	777	793	813
814	863	870	914	914	927	946	986
995	1005	1016	1039	1061	1065	1091	1150
1161	1179	1186	1219	1278	1292	1302	1349
1373	1396	1412	1445	1462	1465	1481	1494
1498	1526	1623	1651	1661	1708	1711	3055
3118	3157	3192	3196	3196	3201	3203	3214
3215	3222	3226					

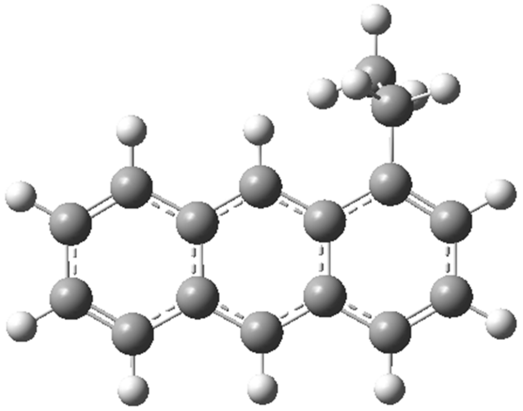

Figure S49 Visualization of the optimized structure of α-ethylantracene molecule, calculated at the M06-2X/aug-aug-cc-pVDZ level of theory.

Table S97 Geometry (Å) of α-ethylantracene molecule, calculated at the M06-2X/aug-aug-cc-pVDZ level of theory

Atom	x	y	z
C	-5.911	6.026	-2.805
C	-4.560	6.003	-2.605
C	-3.907	4.815	-2.141
C	-4.701	3.647	-1.890
C	-6.115	3.710	-2.111
C	-6.701	4.861	-2.554
C	-2.524	4.767	-1.930
C	-4.069	2.485	-1.435
C	-2.688	2.437	-1.222
C	-1.887	3.606	-1.476
C	-0.461	3.544	-1.247
C	0.086	2.375	-0.788
C	-0.708	1.214	-0.539
C	-2.054	1.242	-0.751
H	-1.944	5.666	-2.129
H	-6.396	6.935	-3.158
H	-3.955	6.890	-2.795
H	-6.713	2.820	-1.918
H	-7.777	4.899	-2.720
H	-4.668	1.595	-1.238
H	-2.670	0.363	-0.564
H	-0.226	0.307	-0.177
C	0.420	4.731	-1.546
H	-0.032	5.648	-1.144
H	1.374	4.597	-1.021
C	0.692	4.893	-3.047
H	-0.240	5.007	-3.612
H	1.322	5.771	-3.236
H	1.210	4.007	-3.433
H	1.160	2.326	-0.608

Table S98 Frequencies (cm^{-1}) of α-ethylantracene molecule, calculated at the M06-2X/aug-aug-cc-pVDZ level of theory.

73	86	110	152	188	227	234	274
322	353	396	428	455	486	510	519
547	585	623	636	652	729	752	774
784	788	809	819	864	868	912	915
921	936	960	993	1001	1014	1026	1035
1084	1088	1101	1150	1159	1178	1184	1216
1260	1274	1290	1299	1341	1354	1374	1384
1408	1444	1469	1472	1473	1481	1492	1498
1524	1620	1648	1658	1706	1711	3065	3074
3116	3145	3148	3192	3193	3197	3201	3203

3215	3216	3222	3227			

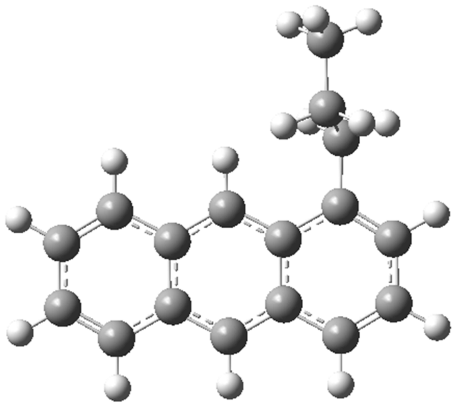

Figure S50 Visualization of the optimized structure of α-propylantracene molecule, calculated at the M06-2X/aug-aug-cc-pVDZ level of theory.

Table S99 Geometry (Å) of α-propylantracene molecule, calculated at the M06-2X/aug-aug-cc-pVDZ level of theory

Atom	x	y	z
C	-1.357	-3.701	0.089
C	0.010	-3.776	0.090
C	0.778	-2.553	0.033
C	0.084	-1.293	-0.018
C	-1.348	-1.282	-0.010
C	-2.047	-2.451	0.042
C	2.178	-2.545	0.020
C	0.815	-0.102	-0.079
C	2.213	-0.100	-0.092
C	2.909	-1.353	-0.042
C	4.342	-1.345	-0.057
H	4.869	-2.298	-0.019
C	5.034	-0.169	-0.117
C	4.339	1.080	-0.167
C	2.973	1.112	-0.154
H	2.730	-3.483	0.059
H	-1.939	-4.622	0.126
H	-1.863	-0.322	-0.047
H	-3.136	-2.443	0.045
H	0.279	0.847	-0.120
H	6.123	-0.175	-0.128
H	4.908	2.007	-0.214
H	2.439	2.061	-0.192
C	0.700	-5.113	0.189
H	1.534	-5.166	-0.525
H	-0.009	-5.899	-0.101
C	1.215	-5.414	1.604

**H**	1.858	-4.592	1.945
**H**	0.356	-5.438	2.287
**C**	1.975	-6.735	1.664
**H**	2.854	-6.709	1.007
**H**	2.319	-6.951	2.683
**H**	1.337	-7.567	1.338

Table S100 Frequencies (cm^{-1}) of α-propylantracene molecule, calculated at the M06-2X/aug-aug-cc-pVDZ level of theory.

52	62	88	108	137	206	225	247
271	295	313	352	402	426	454	487
510	534	561	589	622	641	652	734
747	753	777	787	811	820	858	871
880	904	915	916	926	938	994	1000
1014	1021	1035	1069	1089	1108	1115	1150
1159	1178	1184	1215	1240	1266	1287	1294
1303	1311	1351	1366	1380	1393	1409	1445
1468	1469	1476	1478	1482	1491	1497	1524
1621	1648	1658	1705	1712	3056	3058	3073
3095	3113	3133	3142	3192	3194	3198	3202
3204	3213	3216	3223	3228			

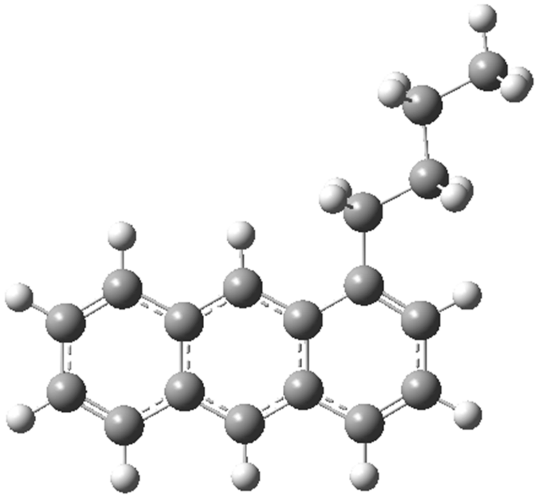

Figure S51 Visualization of the optimized structure of α-butylantracene molecule, calculated at the M06-2X/aug-aug-cc-pVDZ level of theory.

Table S101 Geometry (Å) of α-butylantracene molecule, calculated at the M06-2X/aug-aug-cc-pVDZ level of theory

Atom	x	y	z
**C**	-2.086	-2.660	-0.018
**C**	-0.720	-2.733	-0.013
**C**	0.042	-1.502	-0.013
**C**	-0.653	-0.243	-0.016

**C**	-2.086	-0.235	-0.020
**C**	-2.777	-1.408	-0.021
**C**	1.441	-1.491	-0.008
**C**	0.075	0.950	-0.015
**C**	1.474	0.957	-0.011
**C**	2.172	-0.296	-0.008
**C**	3.604	-0.284	-0.003
**H**	4.133	-1.238	-0.001
**C**	4.294	0.894	-0.003
**C**	3.597	2.142	-0.006
**C**	2.232	2.172	-0.010
**H**	1.995	-2.429	-0.006
**H**	-2.679	-3.572	-0.018
**H**	-2.604	0.723	-0.023
**H**	-3.866	-1.404	-0.024
**H**	-0.463	1.900	-0.018
**H**	5.383	0.891	0.001
**H**	4.165	3.071	-0.005
**H**	1.696	3.121	-0.013
**C**	0.019	-4.051	-0.010
**H**	0.686	-4.080	-0.885
**H**	0.681	-4.078	0.870
**C**	-0.844	-5.308	-0.011
**H**	-1.497	-5.310	-0.896
**H**	-1.502	-5.308	0.870
**C**	-0.001	-6.580	-0.007
**H**	0.654	-6.576	0.876
**H**	0.659	-6.578	-0.886
**C**	-0.854	-7.845	-0.008
**H**	-1.498	-7.880	-0.897
**H**	-0.232	-8.748	-0.005
**H**	-1.503	-7.878	0.877

Table S102 Frequencies (cm^{-1}) of α-butylantracene molecule, calculated at the M06-2X/aug-aug-cc-pVDZ level of theory.

42	73	76	96	112	158	186	208
248	260	263	290	305	390	411	411
489	496	512	537	566	594	602	631
661	736	755	756	772	786	794	816
821	865	872	911	914	918	924	942
963	996	1006	1008	1017	1037	1065	1094
1100	1122	1130	1150	1158	1180	1189	1215
1235	1256	1283	1296	1302	1310	1318	1338
1360	1376	1391	1398	1422	1447	1465	1469
1473	1477	1478	1480	1490	1499	1525	1623
1651	1661	1708	1711	3032	3046	3056	3059

3065	3082	3104	3133	3141	3192	3197	3200
3202	3216	3218	3220	3228	3231		

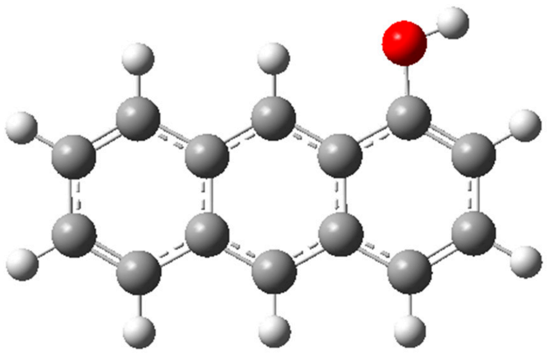

Figure S52 Visualization of the optimized structure of α-hydroxyantracene molecule, calculated at the M06-2X/aug-aug-cc-pVDZ level of theory.

Table S103 Geometry (Å) of α-hydroxyantracene molecule, calculated at the M06-2X/aug-aug-cc-pVDZ level of theory

Atom	x	y	z
C	3.803	1.234	-0.093
C	2.490	1.593	-0.206
C	1.452	0.610	-0.130
C	1.815	-0.765	0.068
C	3.202	-1.101	0.181
C	4.164	-0.135	0.104
C	0.100	0.954	-0.243
C	0.809	-1.735	0.144
C	-0.543	-1.398	0.032
C	-0.897	-0.021	-0.166
C	-2.290	0.314	-0.280
C	-3.256	-0.651	-0.202
C	-2.889	-2.017	-0.006
C	-1.580	-2.385	0.108
H	-0.181	1.995	-0.393
H	4.585	1.990	-0.152
H	2.209	2.635	-0.356
H	3.475	-2.146	0.332
H	5.216	-0.402	0.192
H	1.086	-2.779	0.294
H	-1.304	-3.428	0.258
H	-3.676	-2.767	0.052
H	-4.308	-0.380	-0.291
O	-2.564	1.637	-0.466
H	-3.517	1.757	-0.530

Table S104 Frequencies (cm^{-1}) of α-hydroxyantracene molecule, calculated at the M06-2X/aug-aug-cc-pVDZ level of theory.

93	101	201	208	262	298	334	388
394	424	486	490	513	531	554	587
625	630	650	746	749	773	792	814
826	870	874	886	913	928	943	992
996	1017	1038	1052	1092	1146	1160	1178
1182	1209	1251	1286	1290	1314	1355	1380
1423	1454	1481	1491	1504	1535	1620	1651
1658	1710	1719	3192	3195	3198	3202	3209
3215	3225	3227	3227	3882			

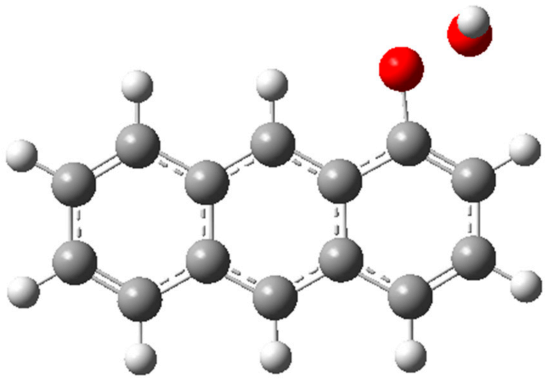

Figure S53 Visualization of the optimized structure of α-peroxyantracene molecule, calculated at the M06-2X/aug-aug-cc-pVDZ level of theory.

Table S105 Geometry (Å) of α-peroxyantracene molecule, calculated at the M06-2X/aug-aug-cc-pVDZ level of theory

Atom	x	y	z
C	3.779	1.248	-0.097
C	2.464	1.600	-0.207
C	1.432	0.610	-0.132
C	1.803	-0.762	0.062
C	3.193	-1.092	0.172
C	4.149	-0.120	0.095
C	0.077	0.948	-0.243
C	0.802	-1.737	0.139
C	-0.552	-1.408	0.030
C	-0.914	-0.033	-0.166
C	-2.316	0.280	-0.277
C	-3.281	-0.681	-0.193
C	-2.892	-2.043	0.005
C	-1.582	-2.402	0.112
H	-0.201	1.990	-0.387
H	4.556	2.008	-0.155
H	2.177	2.641	-0.353
H	3.471	-2.135	0.320

**H**	5.203	-0.381	0.181
**H**	1.084	-2.780	0.288
**H**	-1.296	-3.442	0.262
**H**	-3.673	-2.799	0.071
**H**	-4.329	-0.412	-0.264
**O**	-2.554	1.620	-0.470
**O**	-3.935	1.886	-0.567
**H**	-4.045	1.996	-1.524

Table S106 Frequencies (cm^{-1}) of α-peroxyantracene molecule, calculated at the M06-2X/aug-aug-cc-pVDZ level of theory.

73	95	118	160	189	217	265	301
304	346	395	420	486	489	513	535
587	606	623	633	670	751	769	772
791	815	821	864	873	900	914	928
942	995	999	1002	1017	1037	1083	1106
1150	1163	1182	1185	1224	1274	1293	1300
1352	1374	1404	1436	1450	1480	1485	1498
1525	1619	1650	1660	1713	1723	3197	3200
3204	3209	3218	3226	3227	3230	3271	3803

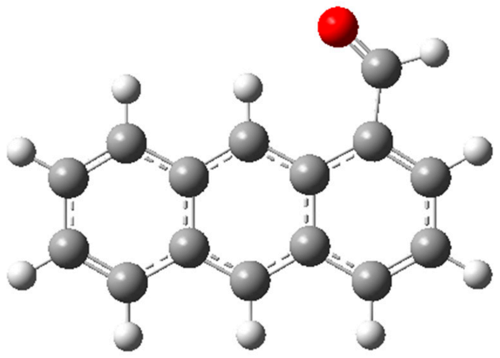

Figure S54 Visualization of the optimized structure of α-antraldehyde molecule, calculated at the M06-2X/aug-aug-cc-pVDZ level of theory.

Table S107 Geometry (Å) of α-antraldehyde molecule, calculated at the M06-2X/aug-aug-cc-pVDZ level of theory

Atom	x	y	z
**C**	3.854	1.197	-0.084
**C**	2.547	1.577	-0.194
**C**	1.494	0.609	-0.122
**C**	1.840	-0.769	0.068
**C**	3.221	-1.131	0.179
**C**	4.197	-0.178	0.105
**C**	0.148	0.987	-0.234
**C**	0.815	-1.718	0.138

**C**	-0.528	-1.347	0.028
**C**	-0.876	0.038	-0.163
**C**	-2.281	0.367	-0.271
**C**	-3.231	-0.623	-0.192
**C**	-2.880	-1.989	-0.003
**C**	-1.563	-2.334	0.103
**H**	-0.100	2.034	-0.377
**H**	4.647	1.941	-0.139
**H**	2.281	2.624	-0.338
**H**	3.477	-2.180	0.323
**H**	5.245	-0.461	0.191
**H**	1.066	-2.769	0.282
**H**	-1.273	-3.375	0.247
**H**	-3.660	-2.746	0.055
**H**	-4.284	-0.351	-0.276
**C**	-2.808	1.739	-0.469
**O**	-2.164	2.759	-0.566
**H**	-3.919	1.777	-0.527

Table S108 Frequencies (cm^{-1}) of α-antraldehyde molecule, calculated at the M06-2X/aug-aug-cc-pVDZ level of theory.

70	96	132	172	227	254	276	312
376	405	410	470	490	520	543	561
609	623	638	668	745	757	777	796
815	826	877	882	916	927	955	967
999	1016	1019	1037	1044	1052	1095	1151
1160	1182	1186	1217	1265	1292	1299	1355
1372	1408	1428	1446	1478	1480	1495	1524
1615	1643	1656	1700	1713	1818	2955	3191
3197	3199	3204	3205	3219	3230	3232	3255

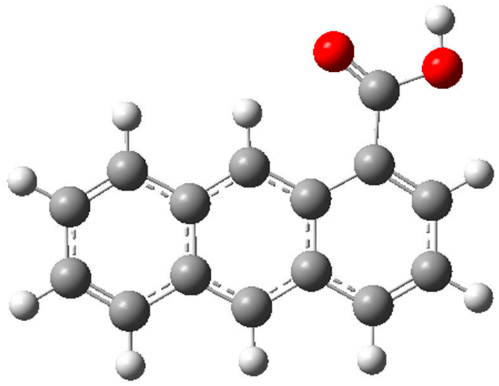

Figure S55 Visualization of the optimized structure of α-antracenic acid molecule, calculated at the M06-2X/aug-aug-cc-pVDZ level of theory.

Table S109 Geometry (Å) of α-antracenic acid molecule, calculated at the M06-2X/aug-aug-cc-pVDZ level of theory

Atom	x	y	z
C	3.840	1.206	-0.084
C	2.532	1.584	-0.193
C	1.480	0.614	-0.122
C	1.830	-0.763	0.066
C	3.212	-1.124	0.176
C	4.185	-0.169	0.103
C	0.135	0.991	-0.232
C	0.804	-1.709	0.136
C	-0.540	-1.338	0.026
C	-0.897	0.046	-0.163
C	-2.308	0.362	-0.269
C	-3.248	-0.637	-0.189
C	-2.881	-1.998	-0.003
C	-1.563	-2.334	0.102
H	-0.103	2.039	-0.374
H	4.631	1.952	-0.140
H	2.264	2.630	-0.336
H	3.469	-2.173	0.319
H	5.234	-0.449	0.188
H	1.053	-2.761	0.279
H	-1.264	-3.373	0.245
H	-3.657	-2.758	0.055
H	-4.301	-0.381	-0.271
C	-2.787	1.761	-0.466
O	-2.103	2.755	-0.558
O	-4.137	1.849	-0.541
H	-4.333	2.788	-0.667

Table S110 Frequencies (cm^{-1}) of α-antracenic acid molecule, calculated at the M06-2X/aug-aug-cc-pVDZ level of theory.

27	69	97	155	174	251	278	281
339	374	402	422	488	509	528	528
543	584	622	624	646	660	740	756
768	787	794	809	848	869	878	915
928	962	972	985	999	1019	1020	1036
1101	1140	1151	1170	1187	1190	1222	1262
1294	1300	1344	1368	1377	1418	1444	1466
1483	1495	1525	1616	1641	1654	1697	1710
1832	3194	3199	3201	3206	3217	3228	3229
3251	3272	3831					

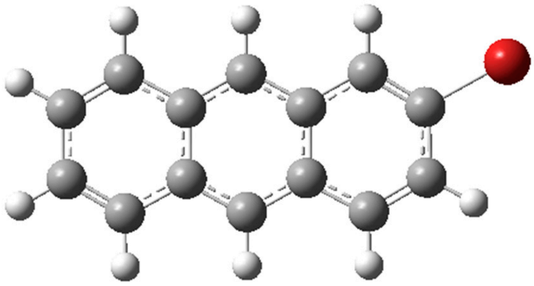

Figure S56 Visualization of the optimized structure of β-bromoantracene molecule, calculated at the M06-2X/aug-aug-cc-pVDZ level of theory.

Table S111 Geometry (Å) of β-bromoantracene molecule, calculated at the M06-2X/aug-aug-cc-pVDZ level of theory

Atom	x	y	z
C	-5.905	5.975	-2.834
C	-4.559	5.972	-2.600
C	-3.898	4.789	-2.137
C	-4.679	3.602	-1.922
C	-6.087	3.646	-2.179
C	-6.681	4.794	-2.620
C	-2.522	4.759	-1.890
C	-4.045	2.439	-1.469
C	-2.670	2.410	-1.223
C	-1.890	3.597	-1.438
C	-0.480	3.559	-1.182
H	0.117	4.454	-1.343
C	0.094	2.405	-0.743
C	-0.665	1.215	-0.523
C	-2.008	1.227	-0.759
H	-1.930	5.661	-2.053
H	-6.396	6.881	-3.186
H	-3.965	6.871	-2.762
H	-6.676	2.744	-2.015
H	-7.752	4.817	-2.812
H	-4.637	1.537	-1.306
H	-2.598	0.326	-0.596
H	-0.161	0.317	-0.171
Br	1.962	2.344	-0.401

Table S112 Frequencies (cm^{-1}) of β-bromoantracene molecule, calculated at the M06-2X/aug-aug-cc-pVDZ level of theory.

58	114	136	168	238	259	301	318
400	417	452	484	494	541	550	616
623	642	698	761	770	782	794	828
842	865	895	909	930	934	945	994

1009	1015	1036	1085	1137	1146	1170	1178
1196	1270	1284	1295	1333	1363	1380	1449
1473	1478	1489	1521	1612	1645	1653	1702
1712	3191	3192	3197	3200	3204	3217	3221
3229	3238						

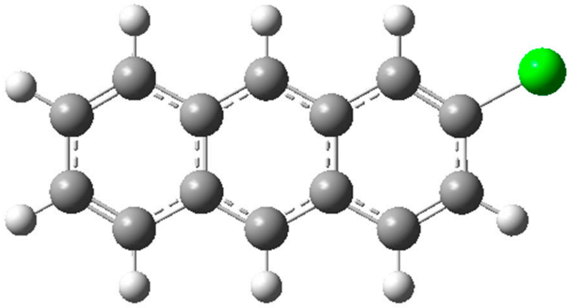

Figure S57 Visualization of the optimized structure of β-chloroantracene molecule, calculated at the M06-2X/aug-aug-cc-pVDZ level of theory.

Table S113 Geometry (Å) of β-chloroantracene molecule, calculated at the M06-2X/aug-aug-cc-pVDZ level of theory

Atom	x	y	z
C	-5.905	5.975	-2.834
C	-4.560	5.972	-2.600
C	-3.899	4.789	-2.137
C	-4.679	3.602	-1.922
C	-6.088	3.647	-2.179
C	-6.681	4.794	-2.620
C	-2.522	4.759	-1.890
C	-4.045	2.440	-1.470
C	-2.670	2.410	-1.223
C	-1.890	3.597	-1.438
C	-0.480	3.559	-1.182
H	0.122	4.451	-1.341
C	0.093	2.405	-0.743
C	-0.665	1.215	-0.523
C	-2.008	1.227	-0.759
H	-1.931	5.661	-2.053
H	-6.396	6.881	-3.186
H	-3.966	6.872	-2.762
H	-6.676	2.744	-2.015
H	-7.753	4.817	-2.812
H	-4.636	1.538	-1.306
H	-2.599	0.326	-0.596
Cl	1.811	2.345	-0.428
H	-0.158	0.320	-0.171

Table S114 Frequencies (cm^{-1}) of β-chloroantracene molecule, calculated at the M06-2X/aug-aug-cc-pVDZ level of theory.

67	114	164	177	259	285	326	337
402	424	476	485	494	544	561	622
626	646	719	763	771	782	794	829
846	866	895	910	930	945	948	995
1008	1016	1039	1100	1139	1147	1171	1181
1200	1272	1287	1297	1333	1367	1383	1452
1477	1480	1492	1524	1614	1645	1656	1705
1712	3193	3195	3198	3202	3207	3216	3222
3227	3237						

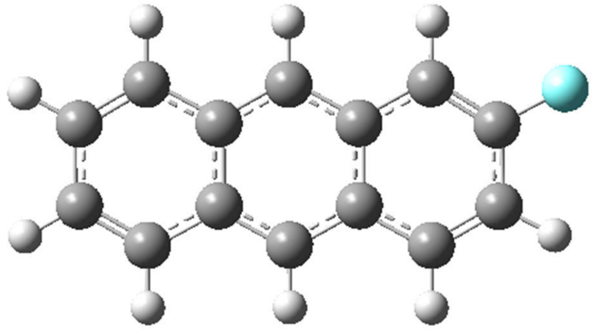

Figure S58 Visualization of the optimized structure of β-fluoroantracene molecule, calculated at the M06-2X/aug-aug-cc-pVDZ level of theory.

Table S115 Geometry (Å) of β-fluoroantracene molecule, calculated at the M06-2X/aug-aug-cc-pVDZ level of theory

Atom	x	y	z
C	-5.906	5.974	-2.834
C	-4.561	5.972	-2.600
C	-3.899	4.790	-2.137
C	-4.679	3.603	-1.922
C	-6.088	3.646	-2.179
C	-6.683	4.793	-2.620
C	-2.522	4.761	-1.890
C	-4.044	2.442	-1.470
C	-2.668	2.413	-1.223
C	-1.887	3.601	-1.438
C	-0.478	3.561	-1.182
H	0.140	4.444	-1.336
C	0.079	2.403	-0.745
C	-0.667	1.213	-0.523
C	-2.011	1.227	-0.760
H	-1.932	5.664	-2.054
H	-6.398	6.880	-3.186

H	-3.968	6.872	-2.763
H	-6.675	2.743	-2.014
H	-7.754	4.815	-2.812
H	-4.634	1.539	-1.306
H	-2.606	0.328	-0.598
H	-0.149	0.323	-0.171
F	1.404	2.346	-0.498

Table S116 Frequencies (cm^{-1}) of β-fluoroantracene molecule, calculated at the M06-2X/aug-aug-cc-pVDZ level of theory.

74	114	197	198	262	333	350	374
410	454	485	493	505	555	582	629
637	658	764	769	776	789	799	832
852	869	892	911	929	943	990	996
1001	1019	1036	1125	1142	1153	1172	1186
1223	1270	1284	1311	1332	1370	1383	1449
1480	1484	1505	1531	1620	1648	1665	1712
1724	3192	3195	3198	3202	3209	3216	3228
3229	3238						

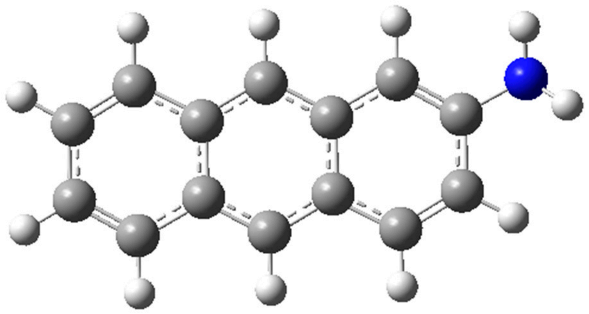

Figure S59 Visualization of the optimized structure of β-aminoantracene molecule, calculated at the M06-2X/aug-aug-cc-pVDZ level of theory.

Table S117 Geometry (Å) of β-aminoantracene molecule, calculated at the M06-2X/aug-aug-cc-pVDZ level of theory

Atom	x	y	z
C	2.856	0.665	0.002
C	2.782	-0.762	0.000
C	1.713	1.414	0.002
C	1.571	-1.395	-0.002
C	0.350	-0.645	-0.002
C	0.426	0.789	0.001
C	-0.761	1.531	0.001
H	-0.710	2.621	0.002
C	-2.009	0.905	-0.001

**C**	-0.903	-1.270	-0.004
**H**	1.763	2.503	0.004
**H**	1.514	-2.483	-0.004
**H**	-0.953	-2.360	-0.006
**C**	-2.090	-0.530	-0.004
**C**	-3.375	-1.155	0.000
**C**	-4.526	-0.408	-0.003
**H**	-3.429	-2.244	0.000
**C**	-4.440	1.027	-0.007
**C**	-3.232	1.653	-0.002
**H**	-5.363	1.607	-0.021
**H**	-3.179	2.742	-0.003
**N**	-5.794	-0.987	-0.069
**H**	-5.836	-1.959	0.204
**H**	-6.536	-0.441	0.349
**H**	3.702	-1.344	0.000
**H**	3.830	1.151	0.004

Table S118 Frequencies (cm^{-1}) of β-aminoantracene molecule, calculated at the M06-2X/aug-aug-cc-pVDZ level of theory.

75	113	190	197	258	297	329	339
376	408	444	481	491	507	542	568
591	630	648	661	762	770	779	794
794	830	854	857	879	910	922	937
976	991	1001	1012	1036	1107	1139	1150
1168	1178	1193	1247	1280	1284	1326	1344
1375	1396	1452	1479	1493	1508	1536	1616
1633	1646	1666	1708	1722	3189	3191	3192
3194	3195	3200	3213	3214	3227	3587	3696

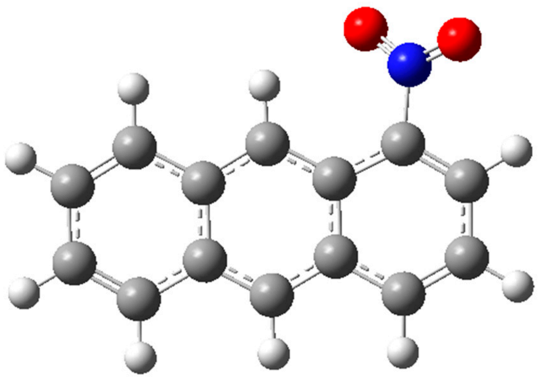

Figure S60 Visualization of the optimized structure of β-nitroantracene molecule, calculated at the M06-2X/aug-aug-cc-pVDZ level of theory.

Table S119 Geometry (Å) of β-nitroantracene molecule, calculated at the M06-2X/aug-aug-cc-pVDZ level of theory

Atom	x	y	z
C	-4.939	-1.462	0.040
C	-3.657	-1.935	0.047
C	-2.545	-1.033	0.013
C	-2.804	0.380	-0.027
C	-4.162	0.835	-0.033
C	-5.195	-0.056	0.000
C	-1.223	-1.491	0.019
C	-1.727	1.272	-0.060
C	-0.406	0.811	-0.054
C	-0.146	-0.602	-0.014
C	1.214	-1.057	-0.009
H	1.405	-2.129	0.022
C	2.254	-0.177	-0.041
C	1.966	1.218	-0.081
C	0.698	1.717	-0.088
H	-1.030	-2.564	0.049
H	-3.460	-3.006	0.077
H	-4.353	1.908	-0.063
H	-1.919	2.345	-0.091
H	0.535	2.792	-0.119
H	3.290	-0.503	-0.038
H	-5.777	-2.157	0.066
H	-6.225	0.298	-0.004
N	3.095	2.164	-0.116
O	2.843	3.353	-0.149
O	4.217	1.694	-0.109

Table S120 Frequencies (cm^{-1}) of β-nitroantracene molecule, calculated at the M06-2X/aug-aug-cc-pVDZ level of theory.

49	64	116	149	171	260	270	310
310	400	411	468	484	495	530	540
565	602	625	644	710	757	768	783
788	798	842	845	868	874	911	914
939	970	981	999	1019	1021	1038	1114
1137	1148	1170	1182	1200	1271	1286	1302
1338	1371	1383	1456	1466	1480	1482	1496
1530	1621	1649	1657	1692	1711	1724	3195
3197	3200	3204	3211	3218	3230	3238	3260

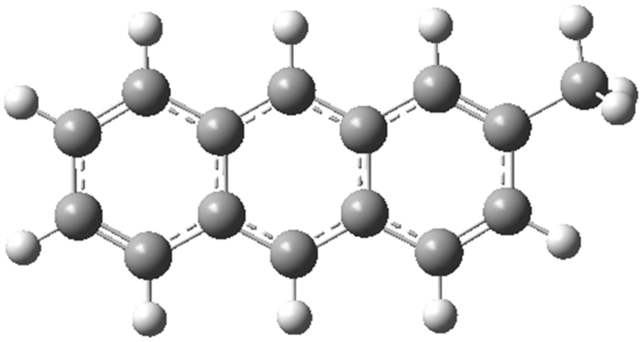

Figure S61 Visualization of the optimized structure of α β-methylantracene molecule, calculated at the M06-2X/aug-aug-cc-pVDZ level of theory.

Table S121 Geometry (Å) of β-methylantracene molecule, calculated at the M06-2X/aug-aug-cc-pVDZ level of theory

Atom	x	y	z
C	-4.365	-0.281	0.026
C	-3.348	-1.192	0.059
C	-1.981	-0.766	0.023
C	-1.696	0.640	-0.048
C	-2.791	1.563	-0.080
C	-4.082	1.118	-0.045
C	-0.919	-1.677	0.056
C	-0.364	1.070	-0.083
C	0.696	0.160	-0.050
C	0.410	-1.246	0.021
C	1.511	-2.164	0.053
H	1.298	-3.231	0.107
C	2.796	-1.713	0.018
C	3.097	-0.310	-0.053
C	2.065	0.586	-0.085
H	-1.133	-2.745	0.109
H	-3.559	-2.260	0.112
H	-2.573	2.629	-0.134
H	-0.150	2.138	-0.136
H	2.271	1.656	-0.139
H	3.623	-2.423	0.043
H	-5.401	-0.617	0.053
H	-4.907	1.829	-0.070
C	4.537	0.124	-0.089
H	5.068	-0.221	0.808
H	5.050	-0.308	-0.958
H	4.621	1.214	-0.144

Table S122 Frequencies (cm^{-1}) of β-methylantracene molecule, calculated at the M06-2X/aug-aug-cc-pVDZ level of theory.

74	112	144	192	194	258	316	330
373	401	428	487	497	506	542	586
613	629	655	762	772	775	785	797
825	850	864	896	910	929	945	961
994	1009	1014	1025	1037	1057	1138	1147
1171	1179	1194	1210	1278	1284	1306	1337
1370	1385	1398	1447	1462	1466	1481	1486
1509	1529	1614	1645	1663	1710	1724	3058
3122	3157	3190	3191	3192	3193	3196	3201
3211	3215	3227					

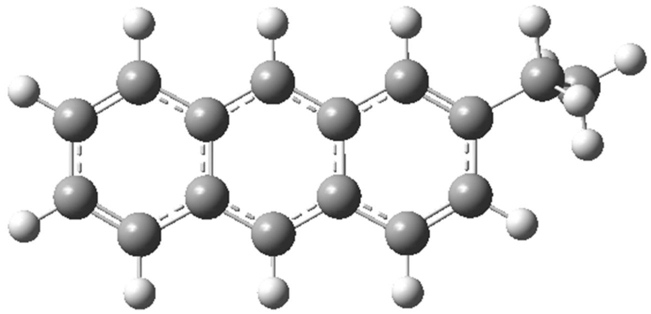

Figure S62 Visualization of the optimized structure of β-ethylantracene molecule, calculated at the M06-2X/aug-aug-cc-pVDZ level of theory.

Table S123 Geometry (Å) of β-ethylantracene molecule, calculated at the M06-2X/aug-aug-cc-pVDZ level of theory

Atom	x	y	z
C	-5.810	5.913	-3.065
C	-4.465	5.873	-2.830
C	-3.859	4.725	-2.224
C	-4.694	3.613	-1.865
C	-6.100	3.694	-2.127
C	-6.641	4.806	-2.708
C	-2.484	4.660	-1.972
C	-4.114	2.487	-1.271
C	-2.741	2.422	-1.022
C	-1.904	3.533	-1.380
C	-0.498	3.452	-1.116
H	0.129	4.303	-1.388
C	0.062	2.347	-0.537
C	-0.786	1.243	-0.184
C	-2.129	1.278	-0.414
H	-1.851	5.505	-2.244
H	-6.259	6.791	-3.527

**H**	-3.829	6.715	-3.101
**H**	-6.730	2.848	-1.854
**H**	-7.711	4.856	-2.903
**H**	-4.747	1.641	-0.998
**H**	-2.762	0.435	-0.134
**H**	-0.336	0.367	0.284
**C**	1.545	2.236	-0.293
**H**	2.005	3.228	-0.393
**H**	1.719	1.900	0.739
**C**	2.213	1.259	-1.266
**H**	3.289	1.183	-1.068
**H**	1.776	0.256	-1.176
**H**	2.072	1.595	-2.301

Table S124 Frequencies (cm^{-1}) of β-ethylantracene molecule, calculated at the M06-2X/aug-aug-cc-pVDZ level of theory.

48	68	111	149	178	233	239	266
320	354	391	419	442	486	496	508
556	586	628	645	660	760	762	776
780	788	797	833	847	865	897	910
931	944	953	994	996	1006	1013	1038
1076	1090	1139	1148	1172	1180	1195	1209
1260	1282	1286	1307	1338	1345	1373	1383
1390	1452	1464	1472	1478	1482	1483	1509
1529	1615	1645	1663	1711	1722	3062	3066
3114	3142	3147	3188	3189	3192	3194	3198
3202	3210	3216	3228				

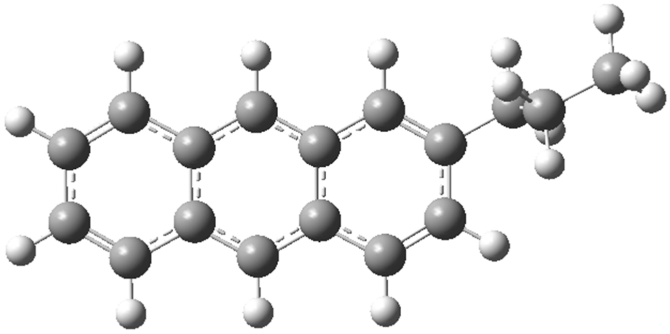

Figure S63 Visualization of the optimized structure of β-propylantracene molecule, calculated at the M06-2X/aug-aug-cc-pVDZ level of theory.

Table S125 Geometry (Å) of β-propylantracene molecule, calculated at the M06-2X/aug-aug-cc-pVDZ level of theory

Atom	x	y	z
**C**	-1.552	-3.064	-0.049

**C**	-0.186	-3.041	0.006
**C**	0.548	-1.810	-0.008
**C**	-0.177	-0.573	-0.083
**C**	-1.608	-0.624	-0.142
**C**	-2.264	-1.818	-0.124
**C**	1.945	-1.779	0.047
**C**	0.524	0.636	-0.098
**C**	1.923	0.667	-0.043
**C**	2.649	-0.570	0.032
**C**	4.080	-0.524	0.087
**H**	4.629	-1.464	0.143
**C**	4.743	0.669	0.071
**C**	4.020	1.900	-0.002
**C**	2.655	1.898	-0.057
**H**	2.499	-2.717	0.102
**H**	0.373	-3.976	0.060
**H**	-2.161	0.313	-0.206
**H**	-3.353	-1.841	-0.175
**H**	-0.029	1.574	-0.156
**H**	5.831	0.691	0.115
**H**	4.567	2.842	-0.013
**H**	2.100	2.834	-0.113
**C**	-2.334	-4.349	0.011
**H**	-3.110	-4.344	-0.769
**H**	-1.667	-5.196	-0.202
**C**	-3.001	-4.561	1.375
**H**	-3.642	-3.698	1.601
**H**	-2.220	-4.581	2.148
**C**	-3.821	-5.846	1.420
**H**	-4.286	-5.989	2.403
**H**	-4.620	-5.824	0.667
**H**	-3.189	-6.720	1.214

Table S126 Frequencies (cm^{-1}) of β-propylantracene molecule, calculated at the M06-2X/aug-aug-cc-pVDZ level of theory.

38	56	83	107	133	198	224	244
263	270	333	353	400	420	442	486
496	522	554	599	628	647	669	734
762	772	781	790	798	832	848	864
872	895	910	912	933	945	977	994
1001	1014	1036	1072	1093	1117	1138	1148
1171	1178	1192	1207	1240	1279	1283	1291
1307	1312	1342	1367	1375	1388	1394	1452
1465	1470	1476	1478	1482	1486	1507	1528
1614	1645	1662	1711	1721	3048	3058	3065
3092	3112	3134	3143	3186	3190	3191	3193

3197	3201	3210	3216	3228	

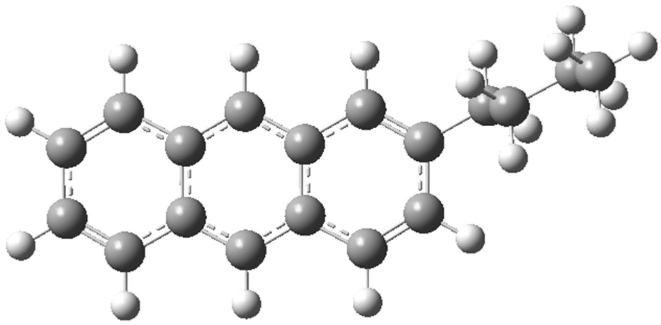

Figure S64 Visualization of the optimized structure of β-butylantracene molecule, calculated at the M06-2X/aug-aug-cc-pVDZ level of theory.

Table S127 Geometry (Å) of β-butylantracene molecule, calculated at the M06-2X/aug-aug-cc-pVDZ level of theory

Atom	x	y	z
C	-1.873	-2.687	-0.091
C	-0.510	-2.671	0.003
C	0.233	-1.445	-0.012
C	-0.482	-0.205	-0.131
C	-1.911	-0.249	-0.231
C	-2.575	-1.440	-0.210
C	1.627	-1.421	0.083
C	0.227	0.999	-0.149
C	1.623	1.023	-0.053
C	2.339	-0.216	0.066
C	3.768	-0.178	0.164
H	4.309	-1.120	0.254
C	4.439	1.011	0.145
C	3.726	2.245	0.026
C	2.364	2.249	-0.069
H	2.173	-2.361	0.173
H	0.042	-3.608	0.091
H	-2.456	0.690	-0.328
H	-3.663	-1.457	-0.291
H	-0.318	1.939	-0.241
H	5.525	1.028	0.220
H	4.279	3.183	0.013
H	1.816	3.187	-0.160
C	-2.664	-3.968	-0.030
H	-3.425	-3.968	-0.824
H	-1.997	-4.820	-0.218
C	-3.357	-4.158	1.324
H	-4.002	-3.290	1.529
H	-2.593	-4.170	2.115

**C**	-4.185	-5.437	1.385
**H**	-4.945	-5.411	0.590
**H**	-3.535	-6.297	1.167
**C**	-4.862	-5.632	2.738
**H**	-5.535	-4.794	2.961
**H**	-5.452	-6.556	2.763
**H**	-4.115	-5.685	3.542

Table S128 Frequencies (cm^{-1}) of β-butylantracene molecule, calculated at the M06-2X/aug-aug-cc-pVDZ level of theory.

27	45	71	96	117	127	196	199
238	244	260	308	338	391	414	423
445	487	497	522	555	600	629	647
667	721	762	773	780	784	793	798
831	852	866	893	910	915	926	933
945	976	994	999	1014	1036	1044	1089
1100	1128	1138	1148	1171	1178	1193	1207
1223	1257	1278	1285	1294	1309	1313	1340
1346	1370	1386	1391	1396	1451	1463	1467
1476	1477	1479	1482	1488	1507	1528	1614
1645	1662	1712	1722	3045	3052	3058	3060
3077	3095	3113	3132	3141	3186	3190	3191
3193	3197	3201	3210	3216	3228		

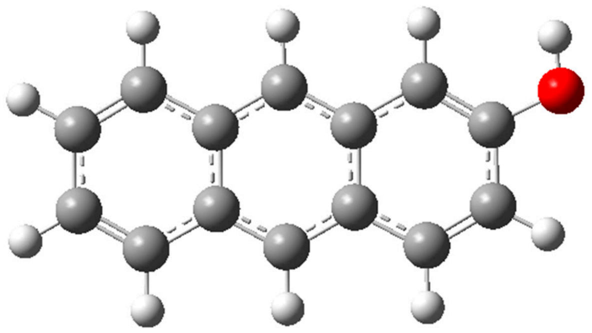

Figure S65 Visualization of the optimized structure of β-hydroxyantracene molecule, calculated at the M06-2X/aug-aug-cc-pVDZ level of theory.

Table S129 Geometry (Å) of β-hydroxyantracene molecule, calculated at the M06-2X/aug-aug-cc-pVDZ level of theory

Atom	x	y	z
**C**	-3.993	-0.905	0.000
**C**	-2.770	-1.511	0.000
**C**	-1.565	-0.735	0.000
**C**	-1.671	0.697	0.000
**C**	-2.973	1.295	0.000

C	-4.098	0.521	0.000
C	-0.300	-1.332	0.000
C	-0.502	1.465	0.000
C	0.761	0.868	0.000
C	0.870	-0.565	0.000
C	2.171	-1.162	0.000
H	2.255	-2.250	0.000
C	3.289	-0.377	0.000
C	3.190	1.050	0.000
C	1.965	1.646	0.000
H	-0.226	-2.421	0.000
H	-4.901	-1.506	0.000
H	-2.689	-2.598	0.000
H	-3.046	2.382	0.000
H	-5.083	0.985	0.000
H	-0.577	2.554	0.000
H	1.885	2.733	0.000
H	4.112	1.629	0.000
O	4.561	-0.867	0.000
H	4.535	-1.830	0.000

Table S130 Frequencies (cm^{-1}) of β-hydroxyantracene molecule, calculated at the M06-2X/aug-aug-cc-pVDZ level of theory.

76	114	195	199	261	337	345	377
406	420	458	483	492	508	557	586
630	646	662	762	771	779	794	796
838	856	859	879	910	926	939	986
993	1011	1014	1036	1131	1142	1159	1176
1191	1207	1242	1281	1284	1326	1350	1373
1396	1454	1481	1494	1508	1542	1622	1650
1664	1710	1721	3188	3191	3193	3196	3200
3206	3215	3227	3233	3873			

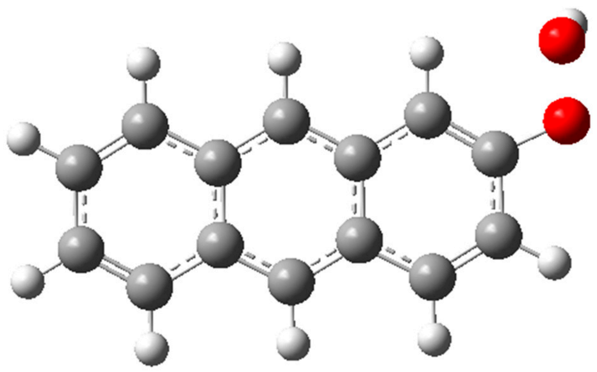

Figure S66 Visualization of the optimized structure of β-peroxyantracene molecule, calculated at the M06-2X/aug-aug-cc-pVDZ level of theory.

Table S131 Geometry (Å) of β-peroxyantracene molecule, calculated at the M06-2X/aug-aug-cc-pVDZ level of theory

Atom	x	y	z
C	-4.417	-0.913	-0.084
C	-3.192	-1.515	-0.031
C	-1.992	-0.734	0.004
C	-2.102	0.697	-0.018
C	-3.404	1.290	-0.073
C	-4.526	0.511	-0.105
C	-0.724	-1.327	0.059
C	-0.935	1.470	0.015
C	0.327	0.876	0.069
C	0.441	-0.556	0.093
C	1.741	-1.160	0.151
H	1.834	-2.242	0.157
C	2.844	-0.361	0.182
C	2.752	1.067	0.154
C	1.529	1.660	0.100
H	-0.645	-2.414	0.075
H	-5.322	-1.518	-0.110
H	-3.107	-2.601	-0.015
H	-3.481	2.377	-0.090
H	-5.512	0.972	-0.148
H	-1.014	2.558	-0.003
H	1.446	2.746	0.079
H	3.673	1.648	0.175
O	4.147	-0.798	0.240
O	4.211	-2.207	0.254
H	4.410	-2.384	1.185

Table S132 Frequencies (cm^{-1}) of β-peroxyantracene molecule, calculated at the M06-2X/aug-aug-cc-pVDZ level of theory.

65	97	135	158	184	211	263	264
348	365	409	414	473	485	494	557
558	606	631	639	653	761	772	779
794	802	832	857	862	884	910	926
940	993	994	1006	1015	1037	1050	1132
1145	1156	1174	1184	1218	1272	1283	1306
1337	1371	1384	1434	1451	1478	1486	1506
1528	1623	1654	1666	1711	1723	3189	3192
3196	3199	3204	3216	3228	3229	3254	3806

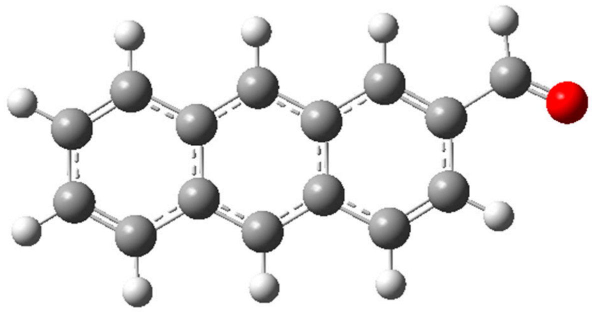

Figure S67 Visualization of the optimized structure of β-antraldehyde molecule, calculated at the M06-2X/aug-aug-cc-pVDZ level of theory.

Table S133 Geometry (Å) of β-antraldehyde molecule, calculated at the M06-2X/aug-aug-cc-pVDZ level of theory

Atom	x	y	z
C	4.414	0.240	0.000
C	3.372	1.122	0.000
C	2.018	0.656	0.000
C	1.776	-0.760	0.000
C	2.896	-1.653	0.000
C	4.173	-1.169	0.000
C	0.931	1.540	0.000
C	0.457	-1.227	0.000
C	-0.625	-0.343	0.000
C	-0.385	1.074	0.000
C	-1.509	1.970	0.000
H	-1.315	3.042	0.000
C	-2.785	1.496	0.000
C	-3.023	0.082	0.000
C	-1.979	-0.804	0.000
H	1.118	2.615	0.000
H	5.440	0.605	0.000
H	3.553	2.197	0.000
H	2.707	-2.726	0.000
H	5.018	-1.856	0.000
H	0.270	-2.302	0.000
H	-2.173	-1.879	0.000
O	-5.396	0.265	-0.001
C	-4.410	-0.436	0.000
H	-3.646	2.161	0.000
H	-4.500	-1.546	-0.001

Table S134 Frequencies (cm^{-1}) of β-antraldehyde molecule, calculated at the M06-2X/aug-aug-cc-pVDZ level of theory.

58	112	130	147	226	259	266	317
339	400	406	486	488	500	541	554

621	621	638	705	764	780	782	797
801	841	848	870	908	911	937	956
977	997	1018	1024	1038	1042	1134	1147
1162	1179	1193	1214	1280	1287	1313	1340
1369	1380	1408	1455	1480	1483	1505	1530
1617	1646	1660	1710	1711	1826	2960	3186
3195	3197	3200	3204	3206	3218	3229	3234

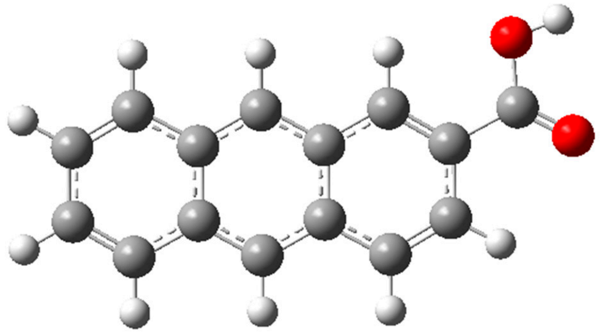

Figure S68 Visualization of the optimized structure of β-antracenic acid molecule, calculated at the M06-2X/aug-aug-cc-pVDZ level of theory.

Table S135 Geometry (Å) of β-antracenic acid molecule, calculated at the M06-2X/aug-aug-cc-pVDZ level of theory

Atom	x	y	z
C	-5.762	5.792	-3.339
C	-4.405	5.663	-3.252
C	-3.817	4.627	-2.457
C	-4.680	3.721	-1.750
C	-6.098	3.890	-1.868
C	-6.621	4.891	-2.635
C	-2.431	4.472	-2.351
C	-4.119	2.704	-0.970
C	-2.735	2.549	-0.864
C	-1.872	3.455	-1.571
C	-0.456	3.290	-1.457
H	0.202	3.974	-1.991
C	0.067	2.287	-0.687
C	-0.790	1.382	0.019
C	-2.143	1.512	-0.069
H	-1.774	5.158	-2.887
H	-6.199	6.583	-3.945
H	-3.746	6.347	-3.786
H	-6.751	3.202	-1.331
H	-7.700	5.010	-2.717
H	-4.776	2.018	-0.434
H	-2.801	0.827	0.466
H	-0.329	0.600	0.619

**C**	1.534	2.092	-0.551
**O**	2.050	1.225	0.113
**O**	2.264	2.990	-1.250
**H**	3.192	2.770	-1.089

Table S136 Frequencies (cm^{-1}) of β-antracenic acid molecule, calculated at the M06-2X/aug-aug-cc-pVDZ level of theory.

55	64	118	134	166	253	260	301
303	398	399	453	485	496	519	523
552	580	622	627	639	671	757	765
770	783	790	801	847	850	873	911
912	938	964	971	995	1017	1019	1037
1109	1136	1145	1166	1178	1196	1220	1276
1284	1302	1335	1367	1382	1397	1456	1477
1482	1506	1530	1616	1646	1660	1711	1711
1842	3192	3193	3197	3201	3204	3217	3222
3229	3236	3823					

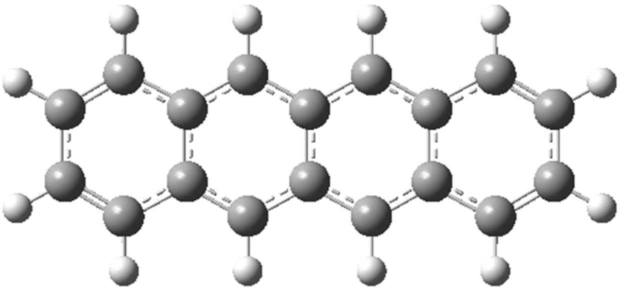

Figure S69 Visualization of the optimized structure of tetracene molecule, calculated at the M06-2X/aug-aug-cc-pVDZ level of theory.

Table S137 Geometry (Å) of tetracene molecule, calculated at the M06-2X/aug-aug-cc-pVDZ level of theory

Atom	x	y	z
**C**	-3.652	0.725	0.000
**C**	-2.477	1.414	0.000
**C**	-1.216	0.723	0.000
**C**	-1.220	-0.722	0.000
**C**	-2.485	-1.406	0.000
**C**	-3.656	-0.711	0.000
**C**	-0.005	1.403	0.000
**C**	-0.013	-1.409	0.000
**C**	1.223	-0.728	0.000
**C**	1.227	0.715	0.000
**C**	2.463	1.396	0.000
**H**	2.466	2.486	0.000
**C**	3.669	0.709	0.000

**C**	2.455	-1.416	0.000
**H**	-0.003	2.493	0.000
**H**	-4.600	1.260	0.000
**H**	-2.471	2.504	0.000
**H**	-2.485	-2.496	0.000
**H**	-4.607	-1.240	0.000
**H**	-0.017	-2.500	0.000
**H**	2.452	-2.507	0.000
**C**	3.665	-0.736	0.000
**C**	4.926	-1.427	0.000
**C**	6.101	-0.739	0.000
**H**	4.920	-2.517	-0.001
**C**	6.105	0.697	0.000
**H**	7.050	-1.273	0.000
**C**	4.934	1.393	0.000
**H**	7.057	1.227	0.000
**H**	4.934	2.483	0.000

Table S138 Frequencies (cm^{-1}) of tetracene molecule, calculated at the M06-2X/aug-aug-cc-pVDZ level of theory.

52	90	153	169	193	279	309	319
326	392	450	478	487	498	501	524
560	573	610	627	637	751	751	765
767	777	780	786	792	854	867	880
888	898	918	930	943	948	994	996
1012	1012	1024	1030	1121	1154	1155	1171
1177	1194	1219	1227	1283	1290	1326	1331
1351	1361	1407	1444	1453	1488	1489	1492
1507	1591	1626	1628	1641	1687	1711	1722
3195	3196	3196	3197	3199	3200	3204	3204
3218	3218	3229	3229				

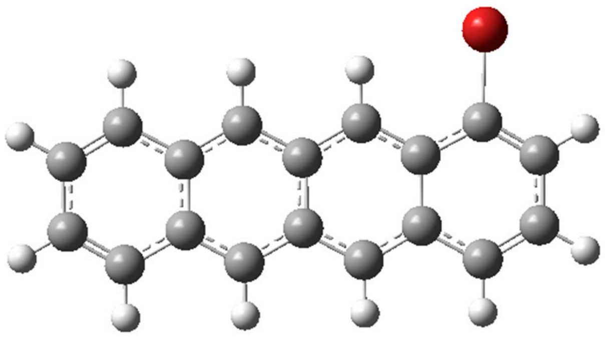

Figure S70 Visualization of the optimized structure of α-bromotetracene molecule, calculated at the M06-2X/aug-aug-cc-pVDZ level of theory.

Table S139 Geometry (Å) of α-bromotetracene molecule, calculated at the M06-2X/aug-aug-cc-pVDZ level of theory

Atom	x	y	z
C	5.295	0.562	0.000
C	4.155	1.307	0.000
C	2.862	0.678	0.000
C	2.796	-0.765	0.000
C	4.026	-1.511	0.000
C	5.229	-0.873	0.000
C	1.686	1.416	0.000
C	1.558	-1.394	0.000
C	0.358	-0.653	0.000
C	0.424	0.786	0.000
C	-0.780	1.520	0.000
H	-0.738	2.609	0.000
C	-2.018	0.892	0.000
C	-0.904	-1.288	0.000
H	1.736	2.506	0.000
H	6.268	1.050	0.000
H	4.202	2.396	0.000
H	3.973	-2.599	0.000
H	6.154	-1.448	0.000
H	1.508	-2.483	0.000
H	-0.941	-2.375	0.000
C	-2.082	-0.556	0.000
C	-3.398	-1.149	0.000
C	-4.532	-0.398	0.000
C	-4.446	1.033	0.000
C	-3.237	1.654	0.000
H	-5.368	1.612	0.000
H	-3.167	2.741	0.000
H	-5.506	-0.883	0.000
Br	-3.572	-3.044	0.000

Table S140 Frequencies (cm^{-1}) of α-bromotetracene molecule, calculated at the M06-2X/aug-aug-cc-pVDZ level of theory.

55	62	103	143	162	195	219	292
305	326	330	361	403	475	481	496
502	522	563	570	573	611	634	672
745	758	768	775	781	790	808	812
861	887	889	910	913	926	945	946
950	996	1006	1014	1027	1066	1130	1156
1164	1177	1186	1208	1227	1272	1294	1318
1328	1350	1355	1397	1440	1453	1468	1487
1492	1501	1590	1614	1629	1642	1686	1705
1720	3197	3198	3200	3201	3205	3209	3219

3226	3230	3230	3240

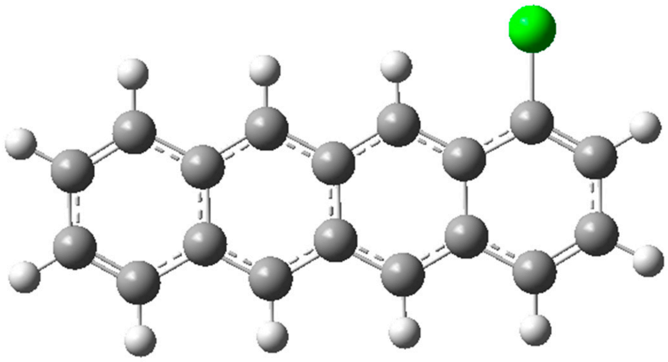

Figure S71 Visualization of the optimized structure of α-chlorotetracene acid molecule, calculated at the M06-2X/aug-aug-cc-pVDZ level of theory.

Table S141 Geometry (Å) of α-chlorotetracene molecule, calculated at the M06-2X/aug-aug-cc-pVDZ level of theory

Atom	x	y	z
C	-5.900	-0.045	0.000
C	-4.941	0.921	0.000
C	-3.545	0.575	0.000
C	-3.180	-0.823	0.000
C	-4.228	-1.808	0.000
C	-5.537	-1.434	0.000
C	-2.548	1.542	0.000
C	-1.838	-1.180	0.000
C	-0.818	-0.206	0.000
C	-1.183	1.189	0.000
C	-0.159	2.159	0.000
H	-0.429	3.215	0.000
C	1.183	1.804	0.000
C	0.548	-0.563	0.000
H	-2.824	2.597	0.000
H	-6.954	0.230	0.000
H	-5.213	1.976	0.000
H	-3.949	-2.861	0.000
H	-6.322	-2.189	0.000
H	-1.562	-2.235	0.000
H	0.815	-1.618	0.000
C	1.545	0.402	0.000
C	2.954	0.092	0.000
C	3.910	1.058	0.000
C	3.531	2.440	0.000
C	2.219	2.800	0.000
H	4.313	3.198	0.000
H	1.928	3.850	0.000

H	4.961	0.778	0.000
Cl	3.450	-1.584	0.000

Table S142 Frequencies (cm^{-1}) of α-chlorotetracene molecule, calculated at the M06-2X/aug-aug-cc-pVDZ level of theory.

58	62	126	146	164	219	224	293
314	330	366	403	406	480	481	496
504	524	565	573	579	611	634	688
745	759	769	776	780	790	813	818
860	888	890	910	914	923	946	950
963	996	1004	1014	1029	1069	1130	1155
1167	1176	1186	1207	1227	1271	1293	1318
1328	1350	1356	1398	1442	1454	1471	1487
1491	1502	1591	1618	1629	1641	1688	1705
1718	3193	3195	3196	3198	3202	3204	3216
3223	3228	3229	3237				

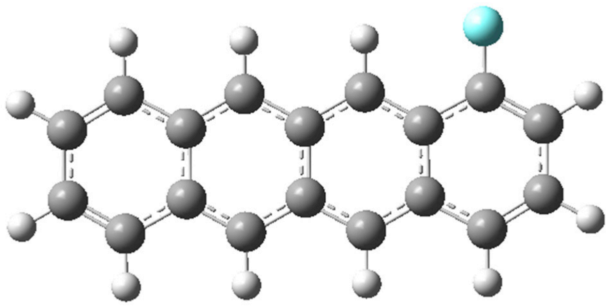

Figure S72 Visualization of the optimized structure of α-fluorotetracene molecule, calculated at the M06-2X/aug-aug-cc-pVDZ level of theory.

Table S143 Geometry (Å) of α-fluorotetracene molecule, calculated at the M06-2X/aug-aug-cc-pVDZ level of theory

Atom	x	y	z
C	5.292	0.551	0.000
C	4.154	1.300	0.000
C	2.860	0.674	0.000
C	2.790	-0.769	0.000
C	4.018	-1.518	0.000
C	5.222	-0.883	0.000
C	1.685	1.415	0.000
C	1.549	-1.394	0.000
C	0.351	-0.650	0.000
C	0.420	0.792	0.000
C	-0.778	1.537	0.000
H	-0.724	2.627	0.000
C	-2.020	0.918	0.000
C	-0.913	-1.278	0.000

**H**	1.739	2.505	0.000
**H**	6.266	1.037	0.000
**H**	4.204	2.388	0.000
**H**	3.962	-2.606	0.000
**H**	6.146	-1.460	0.000
**H**	1.496	-2.483	0.000
**H**	-0.973	-2.365	0.000
**C**	-2.076	-0.525	0.000
**C**	-3.378	-1.126	0.000
**C**	-4.529	-0.411	0.000
**C**	-4.452	1.022	0.000
**C**	-3.251	1.663	0.000
**H**	-5.379	1.594	0.000
**H**	-3.200	2.751	0.000
**H**	-5.486	-0.927	0.000
**F**	-3.421	-2.474	0.000

Table S144 Frequencies (cm^{-1}) of α-fluorotetracene molecule, calculated at the M06-2X/aug-aug-cc-pVDZ level of theory.

54	74	143	151	176	242	246	302
314	333	399	409	479	482	497	507
522	526	567	586	607	610	634	734
746	760	769	774	778	791	821	846
862	886	887	910	917	918	948	952
996	999	1014	1026	1043	1083	1126	1153
1171	1174	1196	1218	1250	1272	1286	1318
1327	1350	1368	1413	1441	1452	1486	1489
1494	1504	1594	1626	1633	1646	1687	1712
1733	3194	3196	3197	3198	3202	3208	3217
3222	3226	3229	3242				

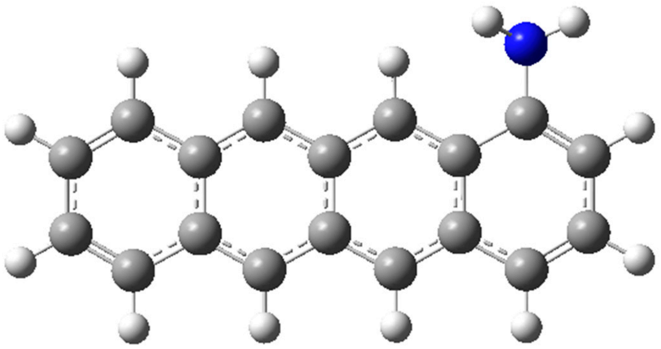

Figure S73 Visualization of the optimized structure of α-aminotetracene molecule, calculated at the M06-2X/aug-aug-cc-pVDZ level of theory.

Table S145 Geometry (Å) of α-aminotetracene molecule, calculated at the M06-2X/aug-aug-cc-pVDZ level of theory

Atom	x	y	z
C	5.298	0.560	-0.004
C	4.159	1.306	0.011
C	2.865	0.678	0.010
C	2.798	-0.765	-0.008
C	4.027	-1.510	-0.025
C	5.231	-0.874	-0.023
C	1.689	1.417	0.023
C	1.558	-1.391	-0.009
C	0.359	-0.650	0.009
C	0.426	0.790	0.021
C	-0.778	1.525	0.015
H	-0.732	2.614	0.004
C	-2.019	0.902	0.014
C	-0.907	-1.278	0.014
H	1.739	2.507	0.032
H	6.272	1.048	-0.003
H	4.206	2.395	0.025
H	3.973	-2.599	-0.039
H	6.156	-1.449	-0.036
H	1.508	-2.481	-0.023
H	-0.933	-2.366	-0.030
C	-2.084	-0.544	0.034
C	-3.388	-1.180	0.061
C	-4.518	-0.407	0.019
C	-4.440	1.019	-0.036
C	-3.239	1.660	-0.025
H	-5.365	1.592	-0.070
H	-3.180	2.747	-0.049
H	-5.497	-0.887	0.039
N	-3.448	-2.576	0.069
H	-2.764	-3.021	0.668
H	-4.376	-2.935	0.255

Table S146 Frequencies (cm^{-1}) of α-aminotetracene molecule, calculated at the M06-2X/aug-aug-cc-pVDZ level of theory.

55	72	144	150	172	231	261	301
314	329	342	405	408	477	481	494
498	523	528	568	586	593	611	634
677	731	746	758	765	776	779	790
818	852	860	878	884	895	908	919
939	943	993	995	1012	1027	1034	1099
1121	1152	1153	1173	1178	1198	1220	1261
1287	1294	1323	1335	1354	1371	1422	1446

1453	1484	1487	1492	1504	1590	1618	1628
1639	1652	1685	1708	1720	3191	3193	3194
3195	3197	3201	3208	3210	3216	3225	3228
3574	3676						

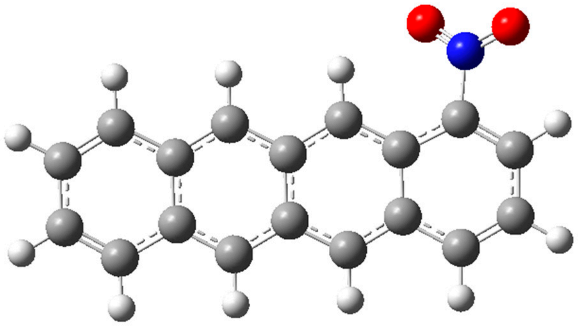

Figure S74 Visualization of the optimized structure of α-nitrotetracene molecule, calculated at the M06-2X/aug-aug-cc-pVDZ level of theory.

Table S147 Geometry (Å) of α-nitrotetracene molecule, calculated at the M06-2X/aug-aug-cc-pVDZ level of theory

Atom	x	y	z
O	4.769	-1.924	-0.439
O	2.936	-2.496	0.522
N	3.676	-1.673	0.023
C	1.836	0.097	0.027
C	1.559	1.522	0.026
C	3.236	-0.263	0.001
C	2.642	2.464	0.017
C	0.765	-0.790	0.015
C	0.246	1.973	0.026
C	4.238	0.659	-0.029
C	3.939	2.054	-0.012
C	-0.570	-0.331	0.007
C	-0.844	1.083	0.017
H	2.395	3.527	0.031
H	0.941	-1.861	0.018
H	0.054	3.049	0.026
H	5.267	0.307	-0.065
H	4.756	2.774	-0.021
C	-1.653	-1.235	-0.008
H	-1.446	-2.307	-0.014
C	-2.969	-0.791	-0.013
C	-3.241	0.629	-0.001
C	-2.183	1.527	0.013
H	-2.386	2.600	0.022
C	-4.079	-1.703	-0.028
H	-3.868	-2.774	-0.036

**C**	-5.361	-1.245	-0.032
**H**	-6.195	-1.947	-0.044
**C**	-5.631	0.165	-0.021
**H**	-6.666	0.509	-0.024
**C**	-4.611	1.066	-0.006
**H**	-4.812	2.139	0.003

Table S148 Frequencies (cm^{-1}) of α-nitrotetracene molecule, calculated at the M06-2X/aug-aug-cc-pVDZ level of theory.

47	56	65	124	138	160	214	224
282	313	326	333	375	392	444	479
489	500	508	524	556	570	605	622
635	679	739	757	766	772	776	784
789	809	833	863	872	888	891	912
921	938	951	966	998	1010	1015	1017
1027	1087	1134	1155	1173	1178	1186	1207
1226	1273	1297	1321	1328	1351	1359	1403
1439	1456	1464	1482	1486	1492	1504	1590
1620	1629	1640	1679	1688	1714	1728	3193
3195	3197	3199	3202	3208	3219	3230	3236
3252	3264						

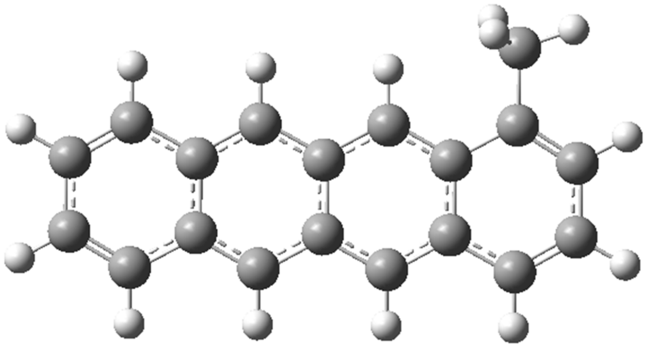

Figure S75 Visualization of the optimized structure of α-methyltetracene molecule, calculated at the M06-2X/aug-aug-cc-pVDZ level of theory.

Table S149 Geometry (Å) of α-methyltetracene molecule, calculated at the M06-2X/aug-aug-cc-pVDZ level of theory

Atom	x	y	z
**C**	-6.045	-3.027	-0.199
**C**	-4.818	-2.459	-0.357
**C**	-3.618	-3.216	-0.120
**C**	-3.742	-4.596	0.290
**C**	-5.059	-5.152	0.444
**C**	-6.168	-4.398	0.209
**C**	-2.355	-2.659	-0.275

**C**	-2.596	-5.344	0.525
**C**	-1.309	-4.787	0.370
**C**	-1.185	-3.410	-0.040
**C**	0.104	-2.853	-0.194
**H**	0.177	-1.811	-0.504
**C**	1.255	-3.594	0.038
**C**	1.127	-4.975	0.450
**C**	-0.135	-5.533	0.604
**H**	-2.263	-1.618	-0.585
**H**	-6.946	-2.443	-0.382
**H**	-2.689	-6.386	0.835
**C**	2.317	-5.744	0.690
**H**	-5.149	-6.193	0.754
**H**	-7.160	-4.830	0.329
**H**	-0.223	-6.575	0.915
**H**	2.212	-6.784	1.000
**C**	3.544	-5.180	0.533
**C**	3.669	-3.810	0.125
**H**	4.667	-3.388	0.008
**C**	2.580	-3.026	-0.119
**H**	4.446	-5.763	0.716
**H**	-4.721	-1.419	-0.667
**C**	2.730	-1.592	-0.546
**H**	2.263	-1.421	-1.526
**H**	2.248	-0.914	0.171
**H**	3.788	-1.323	-0.617

Table S150 Frequencies (cm^{-1}) of α-methyltetracene molecule, calculated at the M06-2X/aug-aug-cc-pVDZ level of theory.

55	71	146	150	169	222	249	257
294	315	332	387	403	469	482	492
495	527	528	562	578	583	613	634
723	750	759	773	776	780	791	808
840	860	884	888	901	916	923	944
945	987	996	1006	1017	1027	1061	1072
1084	1131	1154	1173	1179	1196	1219	1245
1278	1290	1322	1328	1350	1364	1397	1413
1441	1452	1464	1465	1486	1492	1495	1504
1592	1627	1637	1645	1686	1708	1718	3055
3118	3160	3192	3192	3194	3196	3197	3201
3204	3215	3216	3224	3228			

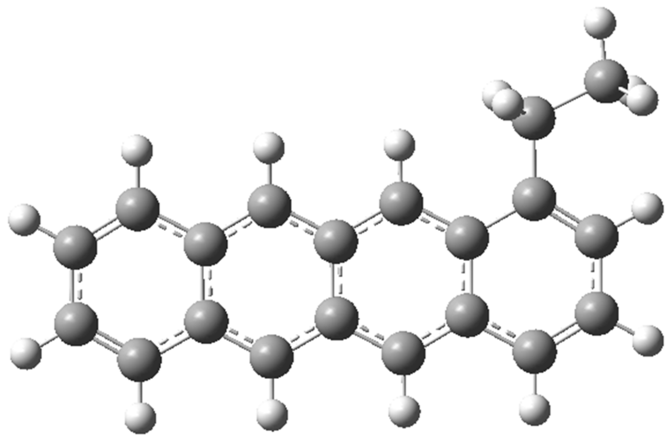

Figure S76 Visualization of the optimized structure of α-ethyltetracene molecule, calculated at the M06-2X/aug-aug-cc-pVDZ level of theory.

Table S151 Geometry (Å) of α-ethyltetracene molecule, calculated at the M06-2X/aug-aug-cc-pVDZ level of theory

Atom	x	y	z
C	-6.003	-3.098	-0.364
C	-4.790	-2.497	-0.538
C	-3.585	-3.251	-0.236
C	-3.707	-4.613	0.240
C	-5.018	-5.181	0.400
C	-6.124	-4.448	0.108
C	-2.315	-2.710	-0.387
C	-2.567	-5.347	0.534
C	-1.274	-4.804	0.382
C	-1.147	-3.449	-0.091
C	0.141	-2.897	-0.246
H	0.240	-1.872	-0.603
C	1.285	-3.631	0.048
C	1.156	-4.989	0.522
C	-0.110	-5.541	0.677
H	-2.192	-1.689	-0.743
H	-6.917	-2.551	-0.585
H	-2.672	-6.373	0.891
C	2.352	-5.730	0.819
H	-5.101	-6.207	0.758
H	-7.117	-4.878	0.229
H	-0.208	-6.567	1.035
H	2.251	-6.755	1.177
C	3.581	-5.167	0.659
C	3.709	-3.817	0.188
H	4.702	-3.387	0.067
C	2.603	-3.078	-0.106
H	2.697	-2.053	-0.464
H	4.479	-5.738	0.888

**C**	-4.656	-1.076	-1.034
**H**	-4.086	-0.502	-0.289
**H**	-4.036	-1.084	-1.943
**C**	-5.966	-0.355	-1.329
**H**	-6.592	-0.282	-0.430
**H**	-5.762	0.662	-1.681
**H**	-6.540	-0.872	-2.107

Table S152 Frequencies (cm^{-1}) of α-ethyltetracene molecule, calculated at the M06-2X/aug-aug-cc-pVDZ level of theory.

52	64	112	127	150	184	221	222
291	294	313	318	333	390	403	481
486	495	506	528	536	564	582	603
614	635	726	750	756	774	777	783
784	794	825	831	860	888	889	910
917	933	947	948	977	995	1014	1016
1027	1028	1096	1103	1103	1135	1154	1172
1183	1196	1218	1242	1279	1292	1294	1324
1327	1337	1357	1373	1392	1420	1445	1454
1476	1478	1482	1486	1492	1493	1503	1594
1629	1640	1646	1688	1709	1719	3047	3066
3082	3141	3149	3189	3189	3191	3195	3198
3198	3216	3217	3219	3228	3233		

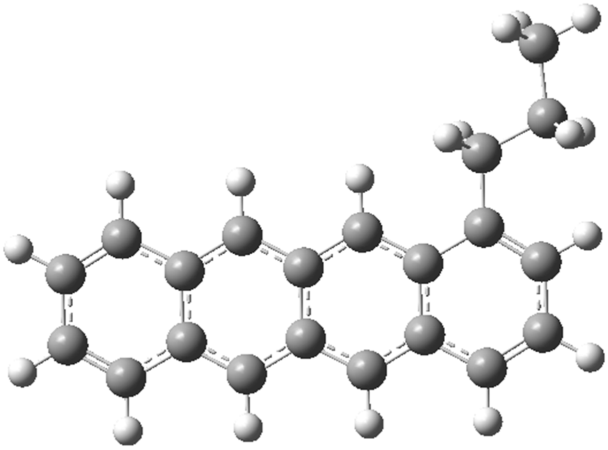

Figure S77 Visualization of the optimized structure of α-propyltetracene molecule, calculated at the M06-2X/aug-aug-cc-pVDZ level of theory.

Table S153 Geometry (Å) of α-propyltetracene molecule, calculated at the M06-2X/aug-aug-cc-pVDZ level of theory

Atom	x	y	z
**C**	-6.009	-2.910	-0.221
**C**	-4.773	-2.367	-0.371

**C**	-3.598	-3.160	-0.127
**C**	-3.754	-4.540	0.279
**C**	-5.092	-5.087	0.430
**C**	-6.164	-4.278	0.182
**C**	-2.328	-2.622	-0.276
**C**	-2.614	-5.298	0.512
**C**	-1.315	-4.761	0.364
**C**	-1.166	-3.387	-0.040
**C**	0.130	-2.850	-0.188
**H**	0.241	-1.810	-0.495
**C**	1.263	-3.619	0.048
**C**	1.114	-4.997	0.454
**C**	-0.159	-5.533	0.602
**H**	-2.223	-1.580	-0.583
**H**	-6.899	-2.310	-0.405
**H**	-2.700	-6.339	0.819
**C**	2.299	-5.775	0.693
**H**	-7.174	-4.668	0.289
**H**	-0.270	-6.574	0.908
**H**	2.184	-6.814	0.999
**C**	3.537	-5.227	0.541
**C**	3.685	-3.858	0.138
**H**	4.684	-3.441	0.023
**C**	2.589	-3.084	-0.099
**H**	2.698	-2.044	-0.406
**H**	4.427	-5.827	0.726
**H**	-4.647	-1.329	-0.677
**C**	-5.247	-6.528	0.854
**H**	-4.712	-7.164	0.131
**H**	-4.726	-6.667	1.814
**C**	-6.677	-7.039	0.993
**H**	-7.203	-6.926	0.036
**H**	-7.216	-6.426	1.728
**C**	-6.709	-8.502	1.425
**H**	-7.739	-8.864	1.524
**H**	-6.196	-9.137	0.692
**H**	-6.210	-8.635	2.394

Table S154 Frequencies (cm^{-1}) of α-propyltetracene molecule, calculated at the M06-2X/aug-aug-cc-pVDZ level of theory.

43	60	88	100	121	157	178	182
235	251	256	297	316	324	330	393
404	482	487	494	503	525	550	563
580	613	620	637	745	749	755	763
774	776	783	793	810	836	859	872
887	889	914	914	926	936	945	949

996	1004	1016	1023	1027	1077	1092	1116
1121	1140	1155	1174	1183	1198	1219	1239
1261	1276	1291	1303	1318	1324	1328	1351
1363	1384	1397	1423	1444	1453	1468	1473
1477	1478	1488	1493	1495	1503	1593	1628
1640	1645	1686	1708	1719	3033	3057	3064
3067	3106	3133	3141	3194	3194	3196	3199
3202	3203	3217	3219	3222	3228	3230	

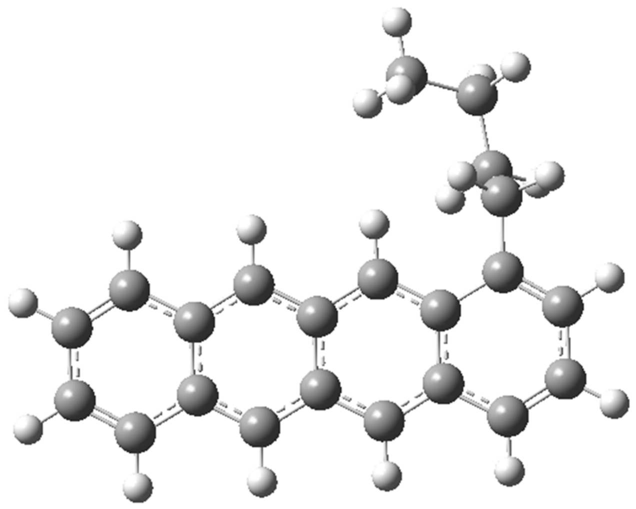

Figure S78 Visualization of the optimized structure of α-butyltetracene molecule, calculated at the M06-2X/aug-aug-cc-pVDZ level of theory.

Table S155 Geometry (Å) of α-butyltetracene molecule, calculated at the M06-2X/aug-aug-cc-pVDZ level of theory

Atom	x	y	z
C	-6.040	-2.700	-0.400
C	-4.768	-2.231	-0.502
C	-3.651	-3.088	-0.211
C	-3.900	-4.457	0.188
C	-5.271	-4.922	0.284
C	-6.286	-4.055	-0.002
C	-2.346	-2.622	-0.303
C	-2.815	-5.278	0.471
C	-1.484	-4.814	0.382
C	-1.241	-3.450	-0.017
C	0.089	-2.988	-0.106
H	0.273	-1.956	-0.408
C	1.165	-3.819	0.180
C	0.922	-5.186	0.579
C	-0.385	-5.649	0.670
H	-2.169	-1.588	-0.602
H	-6.885	-2.049	-0.618
H	-2.973	-6.313	0.771

**C**	2.050	-6.028	0.870
**H**	-7.317	-4.401	0.075
**H**	-0.568	-6.681	0.971
**H**	1.863	-7.059	1.171
**C**	3.322	-5.552	0.773
**C**	3.564	-4.193	0.377
**H**	4.589	-3.834	0.306
**C**	2.525	-3.360	0.092
**H**	2.706	-2.328	-0.210
**H**	4.168	-6.200	0.997
**H**	-4.571	-1.203	-0.803
**C**	-5.557	-6.353	0.657
**H**	-4.943	-6.639	1.521
**H**	-6.604	-6.436	0.976
**C**	-5.320	-7.325	-0.511
**H**	-4.356	-7.103	-0.989
**H**	-6.092	-7.136	-1.269
**C**	-5.350	-8.793	-0.086
**H**	-5.401	-9.423	-0.984
**H**	-6.268	-8.990	0.488
**C**	-4.128	-9.201	0.737
**H**	-4.061	-8.635	1.676
**H**	-4.162	-10.265	0.999
**H**	-3.206	-9.024	0.167

Table S156 Frequencies (cm^{-1}) of α-butyltetracene molecule, calculated at the M06-2X/aug-aug-cc-pVDZ level of theory.

23	50	53	74	111	128	153	160
198	225	254	276	295	315	322	335
390	411	428	473	481	493	500	524
547	569	584	612	632	637	728	740
751	759	771	776	778	790	791	813
852	861	884	886	893	909	916	925
940	943	949	979	997	1000	1017	1027
1034	1084	1101	1109	1120	1137	1155	1173
1179	1198	1210	1220	1242	1260	1277	1294
1304	1312	1325	1332	1353	1353	1366	1384
1394	1413	1443	1451	1468	1470	1476	1481
1485	1485	1492	1493	1503	1592	1627	1636
1644	1686	1707	1719	3054	3057	3062	3074
3099	3100	3121	3131	3142	3191	3194	3194
3195	3199	3203	3204	3213	3218	3224	3229

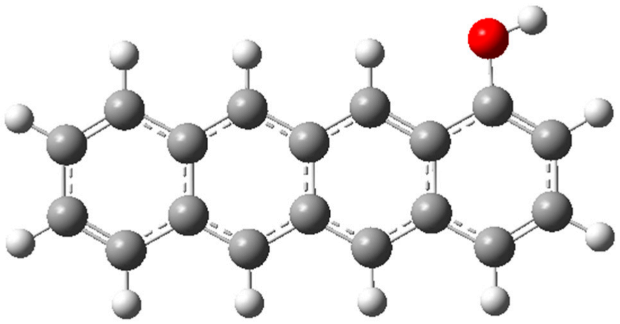

Figure S79 Visualization of the optimized structure of α-hydroxytetracene molecule, calculated at the M06-2X/aug-aug-cc-pVDZ level of theory.

Table S157 Geometry (Å) of α-hydroxytetracene molecule, calculated at the M06-2X/aug-aug-cc-pVDZ level of theory

Atom	x	y	z
C	5.287	0.548	0.000
C	4.150	1.296	0.000
C	2.855	0.672	0.000
C	2.785	-0.771	0.000
C	4.012	-1.520	0.000
C	5.218	-0.886	0.000
C	1.681	1.414	0.000
C	1.543	-1.395	0.000
C	0.346	-0.650	0.000
C	0.415	0.790	0.000
C	-0.785	1.533	0.000
H	-0.732	2.623	0.000
C	-2.027	0.914	0.000
C	-0.919	-1.278	0.000
H	1.735	2.503	0.000
H	6.262	1.033	0.000
H	4.200	2.385	0.000
H	3.955	-2.609	0.000
H	6.140	-1.464	0.000
H	1.489	-2.484	0.000
H	-0.978	-2.365	0.000
C	-2.086	-0.530	0.000
C	-3.386	-1.156	0.000
C	-4.528	-0.410	0.000
C	-4.453	1.021	0.000
C	-3.253	1.664	0.000
H	-5.380	1.592	0.000
H	-3.201	2.752	0.000
H	-5.502	-0.900	0.000
O	-3.376	-2.519	0.000
H	-4.283	-2.843	0.000

Table S158 Frequencies (cm^{-1}) of α-hydroxytetracene molecule, calculated at the M06-2X/aug-aug-cc-pVDZ level of theory.

54	74	144	150	176	237	256	304
314	331	390	404	417	482	483	497
508	523	529	567	588	608	622	634
734	745	760	767	775	777	793	822
848	862	878	884	897	917	917	947
955	991	996	1014	1027	1051	1093	1125
1152	1171	1175	1193	1218	1220	1262	1285
1290	1325	1335	1353	1371	1424	1447	1453
1487	1491	1500	1511	1593	1626	1634	1640
1686	1710	1725	3192	3195	3195	3196	3197
3201	3208	3216	3225	3227	3228	3881	

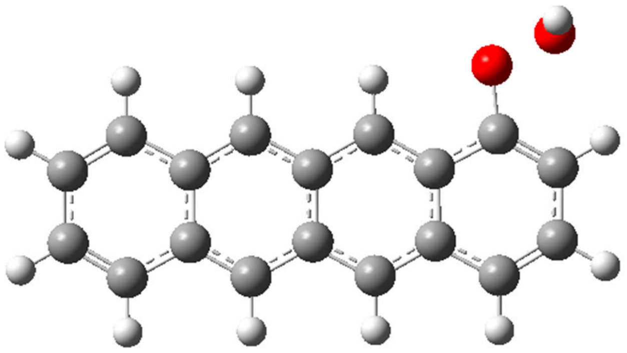

Figure S80 Visualization of the optimized structure of α-peroxytetracene molecule, calculated at the M06-2X/aug-aug-cc-pVDZ level of theory.

Table S159 Geometry (Å) of α-peroxytetracene molecule, calculated at the M06-2X/aug-aug-cc-pVDZ level of theory

Atom	x	y	z
C	5.278	0.544	0.006
C	4.141	1.292	0.010
C	2.846	0.667	0.006
C	2.776	-0.776	-0.003
C	4.004	-1.525	-0.008
C	5.209	-0.890	-0.003
C	1.671	1.408	0.009
C	1.535	-1.400	-0.008
C	0.338	-0.656	-0.003
C	0.407	0.784	0.005
C	-0.794	1.525	0.006
H	-0.744	2.615	0.010
C	-2.035	0.905	0.002
C	-0.927	-1.285	-0.007
H	1.725	2.498	0.015

H	6.253	1.029	0.010
H	4.191	2.381	0.017
H	3.948	-2.613	-0.015
H	6.132	-1.468	-0.006
H	1.482	-2.490	-0.015
H	-0.976	-2.372	-0.016
C	-2.096	-0.538	-0.003
C	-3.404	-1.152	-0.004
C	-4.549	-0.416	-0.010
C	-4.460	1.017	-0.007
C	-3.261	1.657	0.000
H	-5.386	1.590	-0.013
H	-3.207	2.745	0.002
H	-5.516	-0.906	-0.028
O	-3.348	-2.525	-0.001
O	-4.640	-3.090	-0.010
H	-4.745	-3.350	0.917

Table S160 Frequencies (cm^{-1}) of α-peroxytetracene molecule, calculated at the M06-2X/aug-aug-cc-pVDZ level of theory.

54	65	103	124	149	178	194	224
241	308	314	329	333	397	409	481
490	494	510	525	551	570	609	612
631	646	745	759	763	767	776	786
788	814	839	860	884	888	901	911
917	941	946	995	1001	1006	1017	1029
1078	1112	1131	1155	1174	1179	1199	1221
1247	1278	1292	1322	1328	1353	1366	1408
1436	1445	1456	1484	1489	1495	1506	1593
1628	1635	1642	1688	1712	1727	3192	3194
3194	3196	3200	3205	3216	3223	3225	3228
3270	3802						

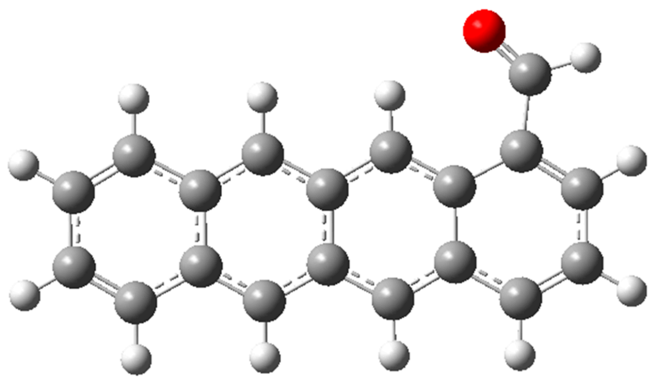

Figure S81 Visualization of the optimized structure of α-tetraldehyde molecule, calculated at the M06-2X/aug-aug-cc-pVDZ level of theory.

Table S161 Geometry (Å) of α-tetraldehyde molecule, calculated at the M06-2X/aug-aug-cc-pVDZ level of theory

Atom	x	y	z
C	5.311	0.591	0.000
C	4.167	1.329	0.000
C	2.878	0.691	0.000
C	2.821	-0.752	0.000
C	4.055	-1.489	0.000
C	5.254	-0.844	0.000
C	1.697	1.422	0.000
C	1.586	-1.389	0.000
C	0.381	-0.657	0.000
C	0.440	0.782	0.000
C	-0.770	1.506	0.000
H	-0.738	2.596	-0.001
C	-1.999	0.862	0.000
C	-0.875	-1.308	0.000
H	1.738	2.512	-0.001
H	6.282	1.085	0.000
H	4.207	2.418	-0.001
H	4.008	-2.578	0.000
H	6.183	-1.413	0.000
H	1.542	-2.479	0.000
H	-0.906	-2.394	0.001
C	-2.060	-0.586	0.000
C	-3.376	-1.201	0.000
C	-4.502	-0.420	0.000
C	-4.436	1.006	0.000
C	-3.220	1.620	0.000
H	-5.354	1.590	0.000
H	-3.148	2.708	0.000
H	-5.479	-0.906	0.000
C	-3.615	-2.663	0.001
O	-2.778	-3.538	0.001
H	-4.697	-2.929	0.001

Table S162 Frequencies (cm^{-1}) of α-tetraldehyde molecule, calculated at the M06-2X/aug-aug-cc-pVDZ level of theory.

55	60	111	130	162	175	221	262
292	312	323	341	403	442	454	482
496	507	532	558	576	583	611	634
659	725	748	762	775	777	782	792
818	862	863	890	894	920	922	938
956	971	998	1015	1019	1030	1043	1058
1092	1136	1156	1176	1181	1200	1220	1244
1278	1296	1319	1328	1355	1369	1414	1430

1445	1457	1483	1488	1493	1505	1591	1621
1629	1640	1686	1699	1716	1816	2957	3194
3195	3195	3198	3199	3200	3203	3216	3228
3228	3258						

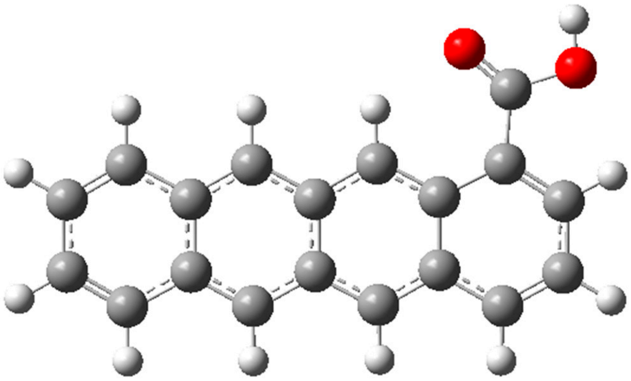

Figure S82 Visualization of the optimized structure of α-tetracenic acid molecule, calculated at the M06-2X/aug-aug-cc-pVDZ level of theory.

Table S163 Geometry (Å) of α-tetracenic acid molecule, calculated at the M06-2X/aug-aug-cc-pVDZ level of theory

Atom	x	y	z
C	5.307	0.594	0.000
C	4.161	1.330	0.000
C	2.874	0.690	0.000
C	2.820	-0.754	0.000
C	4.056	-1.489	0.000
C	5.253	-0.841	0.000
C	1.691	1.417	0.000
C	1.587	-1.394	0.000
C	0.379	-0.666	0.000
C	0.436	0.773	0.000
C	-0.778	1.488	0.000
H	-0.754	2.578	0.000
C	-2.005	0.838	0.000
C	-0.873	-1.322	0.000
H	1.729	2.507	0.000
H	6.276	1.091	0.000
H	4.199	2.419	0.000
H	4.011	-2.577	0.000
H	6.183	-1.407	0.000
H	1.546	-2.484	0.000
H	-0.889	-2.406	0.000
C	-2.068	-0.611	0.000
C	-3.390	-1.220	0.000
C	-4.512	-0.433	0.000

**C**	-4.435	0.992	0.000
**C**	-3.220	1.603	0.000
**H**	-5.353	1.576	0.000
**H**	-3.142	2.690	0.000
**H**	-5.490	-0.907	0.000
**C**	-3.570	-2.700	0.000
**O**	-2.696	-3.536	0.000
**O**	-4.874	-3.071	0.000
**H**	-4.872	-4.039	0.001

Table S164 Frequencies (cm^{-1}) of α-tetracenic acid molecule, calculated at the M06-2X/aug-aug-cc-pVDZ level of theory.

25	54	64	120	139	162	217	223
288	302	323	329	374	397	437	481
494	506	520	522	530	570	587	616
616	634	646	731	746	758	774	777
782	784	792	838	843	863	889	893
918	922	940	967	977	992	997	1017
1024	1027	1093	1133	1154	1163	1175	1187
1202	1219	1238	1275	1296	1321	1328	1350
1357	1383	1422	1441	1456	1476	1485	1492
1503	1591	1624	1629	1640	1685	1701	1718
1830	3191	3192	3196	3197	3201	3203	3218
3230	3230	3255	3267	3827			

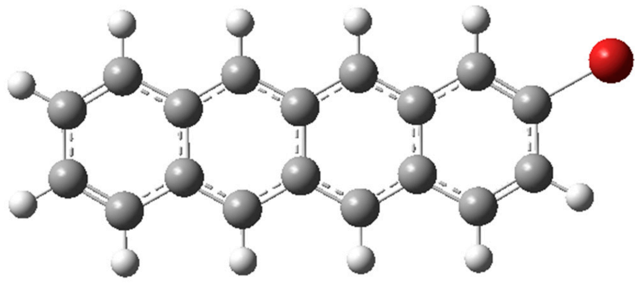

Figure S83 Visualization of the optimized structure of β-bromotetracene molecule, calculated at the M06-2X/aug-aug-cc-pVDZ level of theory.

Table S165 Geometry (Å) of β-bromotetracene molecule, calculated at the M06-2X/aug-aug-cc-pVDZ level of theory

Atom	x	y	z
**C**	-3.650	0.727	0.000
**C**	-2.475	1.415	0.000
**C**	-1.215	0.723	0.000
**C**	-1.220	-0.722	0.000
**C**	-2.485	-1.405	0.000

**C**	-3.655	-0.709	0.000
**C**	-0.003	1.402	0.000
**C**	-0.013	-1.410	0.000
**C**	1.223	-0.730	0.000
**C**	1.227	0.713	0.000
**C**	2.464	1.393	0.000
**H**	2.469	2.484	0.000
**C**	3.668	0.705	0.000
**C**	2.454	-1.420	0.000
**H**	0.000	2.493	0.000
**H**	-4.598	1.262	0.000
**H**	-2.468	2.505	0.000
**H**	-2.486	-2.495	0.000
**H**	-4.606	-1.238	0.000
**H**	-0.018	-2.501	0.000
**H**	2.451	-2.511	0.000
**C**	3.664	-0.740	0.000
**C**	4.921	-1.438	0.000
**C**	6.083	-0.734	0.000
**H**	4.926	-2.526	-0.001
**C**	6.108	0.701	0.000
**C**	4.933	1.388	0.000
**H**	7.065	1.216	0.000
**H**	4.938	2.477	0.000
**Br**	7.750	-1.642	-0.001

Table S166 Frequencies (cm^{-1}) of β-bromotetracene molecule, calculated at the M06-2X/aug-aug-cc-pVDZ level of theory.

40	78	103	118	176	194	225	245
295	336	352	378	395	460	476	486
502	514	543	577	598	629	640	657
746	760	770	778	779	791	798	820
858	875	885	902	906	924	939	948
952	996	1007	1014	1030	1079	1125	1154
1164	1176	1194	1219	1226	1281	1289	1318
1324	1340	1352	1389	1441	1456	1478	1488
1490	1504	1591	1623	1628	1637	1686	1700
1715	3194	3195	3196	3198	3198	3202	3207
3216	3223	3228	3240				

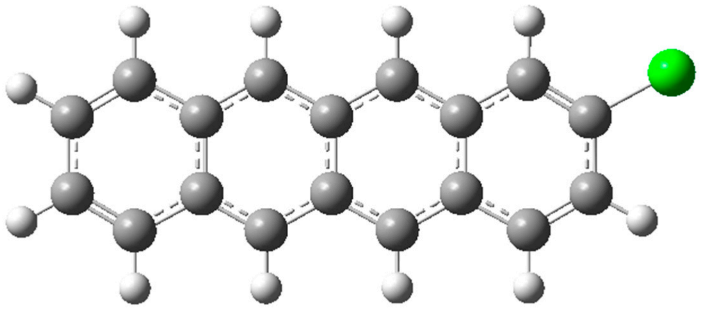

Figure S84 Visualization of the optimized structure of β-chlorotetracene acid molecule, calculated at the M06-2X/aug-aug-cc-pVDZ level of theory.

Table S167 Geometry (Å) of β-chlorotetracene molecule, calculated at the M06-2X/aug-aug-cc-pVDZ level of theory

Atom	x	y	z
C	-3.650	0.727	0.000
C	-2.475	1.415	0.000
C	-1.215	0.723	0.000
C	-1.220	-0.722	0.000
C	-2.485	-1.405	0.000
C	-3.655	-0.709	0.000
C	-0.004	1.402	0.000
C	-0.013	-1.410	0.000
C	1.223	-0.730	0.000
C	1.227	0.713	0.000
C	2.463	1.393	0.000
H	2.469	2.484	0.000
C	3.668	0.705	0.000
C	2.454	-1.420	0.000
H	0.000	2.493	0.000
H	-4.598	1.262	0.000
H	-2.468	2.505	0.000
H	-2.485	-2.495	0.000
H	-4.607	-1.238	0.000
H	-0.018	-2.501	0.000
H	2.450	-2.511	0.000
C	3.664	-0.740	0.000
C	4.922	-1.438	0.000
C	6.082	-0.733	0.000
H	4.933	-2.526	-0.001
C	6.107	0.702	0.000
C	4.933	1.389	0.000
H	7.067	1.212	0.000
H	4.936	2.478	0.000
Cl	7.618	-1.566	0.000

Table S168 Frequencies (cm^{-1}) of β-chlorotetracene molecule, calculated at the M06-2X/aug-aug-cc-pVDZ level of theory.

44	79	120	122	177	224	230	280
296	345	356	397	422	460	477	486
501	518	544	582	605	629	639	682
747	760	770	778	780	792	804	822
859	875	884	902	910	923	947	949
951	996	1006	1013	1028	1092	1123	1153
1159	1174	1190	1216	1224	1278	1287	1317
1323	1338	1350	1389	1440	1453	1479	1487
1491	1503	1591	1624	1627	1638	1686	1703
1717	3194	3195	3196	3198	3198	3203	3208
3217	3224	3228	3239				

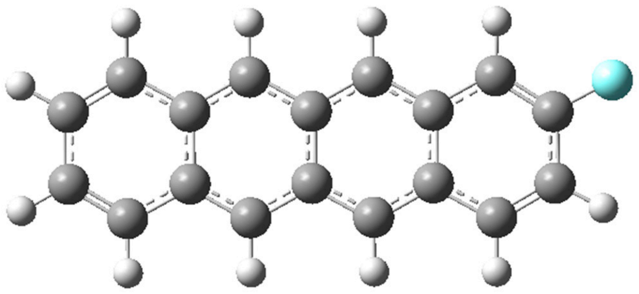

Figure S85 Visualization of the optimized structure of β-fluorotetracene molecule, calculated at the M06-2X/aug-aug-cc-pVDZ level of theory.

Table S169 Geometry (Å) of β-fluorotetracene molecule, calculated at the M06-2X/aug-aug-cc-pVDZ level of theory

Atom	x	y	z
C	-3.650	0.729	0.000
C	-2.475	1.416	0.000
C	-1.215	0.723	0.000
C	-1.220	-0.722	0.000
C	-2.486	-1.404	0.000
C	-3.656	-0.707	0.000
C	-0.003	1.401	0.000
C	-0.014	-1.411	0.000
C	1.222	-0.732	0.000
C	1.226	0.711	0.000
C	2.463	1.390	0.000
H	2.470	2.480	0.000
C	3.668	0.701	0.000
C	2.454	-1.423	0.000
H	0.001	2.492	0.000
H	-4.598	1.265	0.000
H	-2.467	2.506	0.000

**H**	-2.488	-2.494	0.000
**H**	-4.608	-1.236	0.000
**H**	-0.020	-2.501	0.000
**H**	2.448	-2.513	0.000
**C**	3.665	-0.745	0.000
**C**	4.923	-1.441	0.000
**C**	6.070	-0.722	0.000
**H**	4.955	-2.528	-0.001
**C**	6.106	0.706	0.000
**C**	4.931	1.391	0.000
**H**	7.072	1.206	0.000
**H**	4.928	2.481	0.000
**F**	7.263	-1.353	0.000

Table S170 Frequencies (cm^{-1}) of β-fluorotetracene molecule, calculated at the M06-2X/aug-aug-cc-pVDZ level of theory.

45	83	132	142	181	244	267	300
303	369	390	406	470	476	478	485
500	532	548	592	623	630	641	744
745	758	769	778	778	791	824	829
857	869	884	900	915	918	945	948
985	995	997	1012	1027	1117	1137	1153
1168	1174	1195	1218	1252	1275	1286	1320
1327	1342	1351	1391	1444	1452	1483	1488
1501	1513	1594	1626	1634	1647	1689	1713
1731	3193	3195	3195	3198	3198	3202	3209
3217	3229	3229	3238				

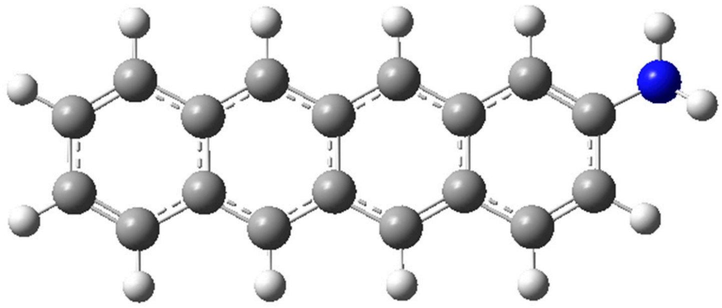

Figure S86 Visualization of the optimized structure of β-aminotetracene molecule, calculated at the M06-2X/aug-aug-cc-pVDZ level of theory.

Table S171 Geometry (Å) of β-aminotetracene molecule, calculated at the M06-2X/aug-aug-cc-pVDZ level of theory

Atom	x	y	z
**C**	-3.656	0.734	0.022
**C**	-2.479	1.419	0.019

**C**	-1.220	0.725	0.007
**C**	-1.226	-0.719	-0.002
**C**	-2.493	-1.399	0.000
**C**	-3.662	-0.701	0.012
**C**	-0.007	1.401	0.005
**C**	-0.020	-1.409	-0.014
**C**	1.218	-0.734	-0.016
**C**	1.221	0.709	-0.007
**C**	2.461	1.386	-0.009
**H**	2.470	2.477	-0.001
**C**	3.662	0.694	-0.021
**C**	2.449	-1.425	-0.028
**H**	0.000	2.492	0.013
**H**	-4.603	1.271	0.031
**H**	-2.470	2.509	0.027
**H**	-2.496	-2.489	-0.007
**H**	-4.615	-1.229	0.014
**H**	-0.028	-2.500	-0.021
**H**	2.440	-2.516	-0.035
**C**	3.664	-0.752	-0.030
**C**	4.921	-1.442	-0.048
**C**	6.106	-0.758	-0.047
**H**	4.917	-2.533	-0.059
**C**	6.098	0.685	-0.030
**C**	4.929	1.376	-0.022
**H**	7.052	1.213	-0.017
**H**	4.934	2.465	-0.012
**N**	7.345	-1.395	0.007
**H**	7.340	-2.369	-0.264
**H**	8.110	-0.884	-0.413

Table S172 Frequencies (cm^{-1}) of β-aminotetracene molecule, calculated at the M06-2X/aug-aug-cc-pVDZ level of theory.

47	82	130	142	179	237	264	296
302	309	361	379	405	468	474	478
484	498	532	544	558	596	631	639
642	745	751	758	771	778	779	788
824	832	848	862	885	894	913	916
939	944	970	995	1000	1013	1027	1106
1128	1152	1162	1172	1187	1208	1220	1273
1280	1287	1322	1344	1347	1362	1401	1446
1452	1487	1490	1502	1521	1592	1625	1628
1636	1650	1688	1711	1729	3190	3191	3192
3192	3194	3194	3196	3200	3213	3216	3228
3587	3697						

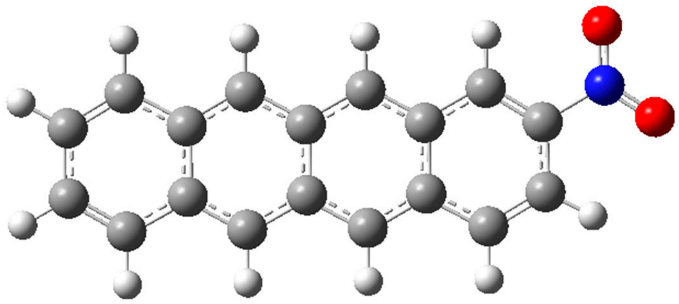

Figure S87 Visualization of the optimized structure of β-nitrotetracene molecule, calculated at the M06-2X/aug-aug-cc-pVDZ level of theory.

Table S173 Geometry (Å) of β-nitrotetracene molecule, calculated at the M06-2X/aug-aug-cc-pVDZ level of theory

Atom	x	y	z
C	-3.646	0.726	0.000
C	-2.471	1.415	0.000
C	-1.212	0.721	0.000
C	-1.219	-0.723	0.000
C	-2.484	-1.407	0.000
C	-3.653	-0.709	0.000
C	0.000	1.401	0.000
C	-0.013	-1.412	0.000
C	1.222	-0.731	0.000
C	1.230	0.713	0.000
C	2.467	1.394	0.000
H	2.470	2.485	0.000
C	3.671	0.707	0.000
C	2.452	-1.422	0.000
H	0.003	2.492	0.000
H	-4.594	1.262	0.000
H	-2.464	2.504	0.000
H	-2.485	-2.497	0.000
H	-4.605	-1.237	0.000
H	-0.017	-2.503	0.000
H	2.451	-2.512	0.000
C	3.661	-0.739	0.000
C	4.912	-1.438	0.000
C	6.070	-0.727	0.000
H	4.939	-2.525	0.000
C	6.112	0.703	0.000
C	4.939	1.389	0.000
H	7.077	1.201	0.000
H	4.941	2.478	0.000
N	7.346	-1.463	0.000
O	7.306	-2.678	0.000
O	8.368	-0.804	0.000

Table S174 Frequencies (cm^{-1}) of β-nitrotetracene molecule, calculated at the M06-2X/aug-aug-cc-pVDZ level of theory.

40	53	80	114	122	176	211	226
268	294	330	346	395	415	447	477
487	503	518	535	549	581	582	630
640	671	744	761	772	772	778	786
790	801	840	864	865	887	888	913
916	939	950	971	979	1000	1016	1019
1029	1112	1126	1155	1158	1177	1194	1221
1231	1281	1290	1322	1329	1344	1353	1394
1448	1456	1464	1484	1489	1496	1508	1595
1627	1634	1642	1685	1692	1714	1726	3196
3198	3199	3200	3201	3205	3214	3218	3230
3241	3263						

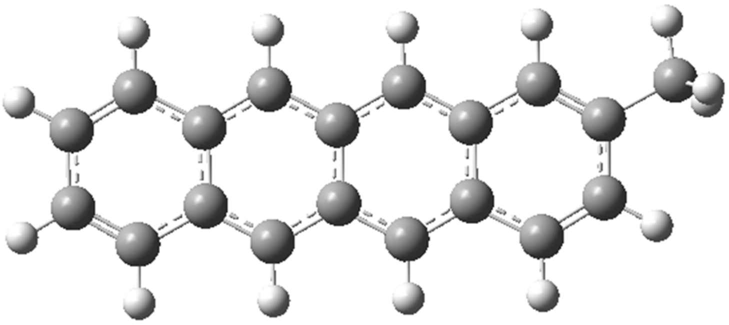

Figure S88 Visualization of the optimized structure of β-methyltetracene molecule, calculated at the M06-2X/aug-aug-cc-pVDZ level of theory.

Table S175 Geometry (Å) of β-methyltetracene molecule, calculated at the M06-2X/aug-aug-cc-pVDZ level of theory

Atom	x	y	z
C	-3.657	0.732	0.002
C	-2.480	1.418	0.033
C	-1.221	0.724	0.015
C	-1.227	-0.719	-0.037
C	-2.493	-1.400	-0.068
C	-3.663	-0.703	-0.049
C	-0.008	1.401	0.046
C	-0.021	-1.408	-0.054
C	1.215	-0.731	-0.024
C	1.221	0.711	0.028
C	2.460	1.388	0.059
H	2.467	2.478	0.097
C	3.663	0.698	0.041
C	2.447	-1.421	-0.041

**H**	-0.004	2.491	0.084
**H**	-4.604	1.268	0.016
**H**	-2.472	2.507	0.071
**H**	-2.495	-2.489	-0.107
**H**	-4.615	-1.230	-0.074
**H**	-0.027	-2.498	-0.093
**H**	2.441	-2.512	-0.080
**C**	3.659	-0.745	-0.011
**C**	4.920	-1.439	-0.028
**C**	6.107	-0.770	0.002
**H**	4.905	-2.529	-0.067
**C**	6.096	0.672	0.053
**C**	4.932	1.374	0.072
**H**	7.051	1.198	0.077
**H**	4.940	2.463	0.110
**C**	7.432	-1.481	-0.016
**H**	8.015	-1.234	0.881
**H**	7.299	-2.567	-0.056
**H**	8.026	-1.171	-0.886

Table S176 Frequencies (cm^{-1}) of β-methyltetracene molecule, calculated at the M06-2X/aug-aug-cc-pVDZ level of theory.

47	83	124	140	163	179	239	254
296	303	356	360	396	462	473	478
486	498	535	543	591	594	631	642
737	749	758	771	779	782	789	816
822	858	875	884	898	913	921	944
946	958	995	1008	1014	1024	1028	1058
1122	1153	1164	1172	1188	1197	1218	1234
1279	1285	1324	1332	1340	1354	1392	1398
1440	1452	1462	1467	1486	1488	1504	1514
1591	1625	1629	1645	1688	1712	1730	3057
3121	3156	3190	3191	3191	3192	3193	3194
3197	3201	3210	3216	3228			

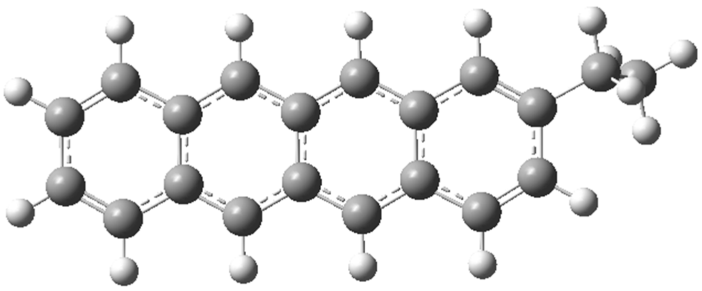

Figure S89 Visualization of the optimized structure of β-ethyltetracene molecule, calculated at the M06-2X/aug-aug-cc-pVDZ level of theory.

Table S177 Geometry (Å) of β-ethyltetracene molecule, calculated at the M06-2X/aug-aug-cc-pVDZ level of theory

Atom	x	y	z
C	-6.086	-3.217	-0.440
C	-4.865	-2.613	-0.484
C	-3.649	-3.341	-0.240
C	-3.735	-4.752	0.057
C	-5.038	-5.362	0.092
C	-6.158	-4.627	-0.142
C	-2.403	-2.730	-0.281
C	-2.576	-5.477	0.294
C	-1.304	-4.866	0.255
C	-1.216	-3.456	-0.040
C	0.054	-2.844	-0.079
H	0.122	-1.778	-0.302
C	1.216	-3.567	0.159
C	1.127	-4.978	0.454
C	-0.119	-5.591	0.496
H	-2.335	-1.665	-0.505
H	-2.645	-6.543	0.517
C	2.342	-5.708	0.698
H	-5.107	-6.428	0.308
H	-7.138	-5.106	-0.116
H	-0.186	-6.656	0.718
H	2.272	-6.773	0.920
C	3.552	-5.085	0.652
C	3.640	-3.683	0.359
H	4.619	-3.206	0.328
C	2.515	-2.953	0.122
H	2.579	-1.888	-0.101
H	4.466	-5.649	0.839
C	-7.368	-2.456	-0.656
H	-7.137	-1.472	-1.084
H	-7.988	-2.991	-1.388
C	-8.156	-2.280	0.647
H	-9.091	-1.735	0.468
H	-8.406	-3.253	1.088
H	-7.561	-1.722	1.380
H	-4.790	-1.548	-0.710

Table S178 Frequencies (cm^{-1}) of β-ethyltetracene molecule, calculated at the M06-2X/aug-aug-cc-pVDZ level of theory.

39	58	77	114	138	165	200	210
260	270	297	320	367	380	414	463

476	482	486	496	534	553	590	631
636	641	730	750	758	771	778	780
783	789	817	822	857	874	884	898
911	919	941	946	953	992	995	1000
1015	1027	1075	1090	1123	1152	1165	1173
1188	1195	1219	1233	1263	1280	1285	1322
1332	1340	1347	1356	1384	1395	1442	1453
1464	1472	1481	1483	1488	1503	1514	1591
1625	1628	1645	1688	1711	1728	3061	3063
3113	3142	3147	3187	3190	3191	3192	3193
3194	3197	3201	3210	3216	3228		

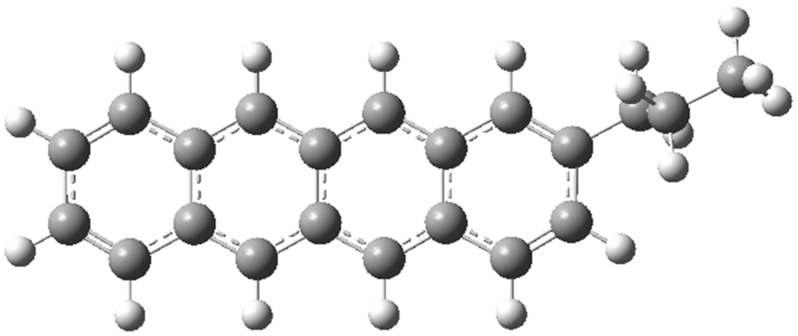

Figure S90 Visualization of the optimized structure of β-propyltetracene molecule, calculated at the M06-2X/aug-aug-cc-pVDZ level of theory.

Table S179 Geometry (Å) of β-propyltetracene molecule, calculated at the M06-2X/aug-aug-cc-pVDZ level of theory

Atom	x	y	z
C	-5.974	-2.995	-0.934
C	-4.727	-2.462	-1.039
C	-3.572	-3.195	-0.594
C	-3.772	-4.513	-0.038
C	-5.112	-5.030	0.046
C	-6.188	-4.310	-0.381
C	-2.289	-2.676	-0.687
C	-2.673	-5.242	0.397
C	-1.363	-4.725	0.309
C	-1.164	-3.408	-0.248
C	0.145	-2.892	-0.337
H	0.295	-1.897	-0.758
C	1.244	-3.622	0.099
C	1.046	-4.939	0.656
C	-0.240	-5.457	0.748
H	-2.141	-1.681	-1.109
H	-6.839	-2.428	-1.280
H	-2.822	-6.238	0.817
C	2.197	-5.677	1.101

**H**	-5.253	-6.028	0.464
**H**	-0.388	-6.452	1.169
**H**	2.044	-6.671	1.521
**C**	3.448	-5.149	1.001
**C**	3.645	-3.839	0.447
**H**	4.654	-3.437	0.376
**C**	2.583	-3.105	0.013
**H**	2.729	-2.110	-0.408
**H**	4.312	-5.718	1.341
**H**	-4.580	-1.470	-1.465
**C**	-7.597	-4.826	-0.257
**H**	-8.131	-4.668	-1.206
**H**	-7.577	-5.908	-0.072
**C**	-8.372	-4.135	0.871
**H**	-7.841	-4.306	1.817
**H**	-8.366	-3.049	0.703
**C**	-9.808	-4.638	0.973
**H**	-9.829	-5.720	1.160
**H**	-10.348	-4.143	1.789
**H**	-10.354	-4.448	0.039

Table S180 Frequencies (cm^{-1}) of β-propyltetracene molecule, calculated at the M06-2X/aug-aug-cc-pVDZ level of theory.

30	46	71	76	105	145	157	191
235	245	257	277	304	348	365	378
408	465	477	485	496	501	543	557
595	631	640	642	733	746	755	759
772	779	782	790	816	829	858	868
878	885	898	908	917	923	943	949
974	996	999	1015	1029	1071	1093	1116
1126	1153	1164	1174	1191	1197	1221	1232
1243	1282	1287	1293	1308	1325	1337	1343
1356	1373	1393	1396	1444	1454	1464	1469
1476	1483	1486	1488	1504	1514	1592	1626
1628	1645	1689	1712	1727	3049	3058	3066
3093	3112	3134	3143	3186	3189	3191	3192
3193	3194	3197	3201	3210	3216	3228	

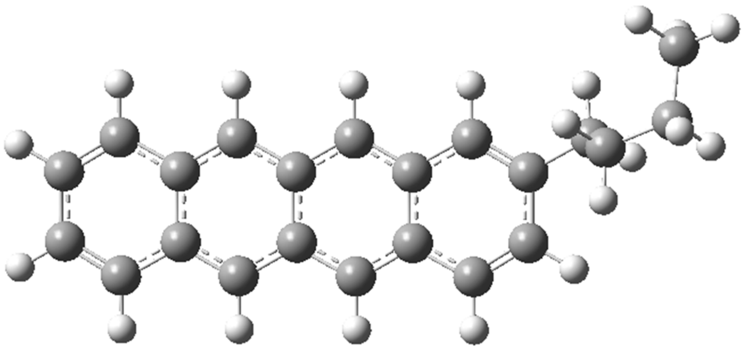

Figure S91 Visualization of the optimized structure of β-butyltetracene molecule, calculated at the M06-2X/aug-aug-cc-pVDZ level of theory.

Table S181 Geometry (Å) of β-butyltetracene molecule, calculated at the M06-2X/aug-aug-cc-pVDZ level of theory

Atom	x	y	z
C	-5.971	-3.110	0.076
C	-4.759	-2.496	0.022
C	-3.542	-3.260	0.091
C	-3.642	-4.695	0.213
C	-4.949	-5.294	0.263
C	-6.086	-4.543	0.200
C	-2.293	-2.659	0.041
C	-2.482	-5.455	0.279
C	-1.204	-4.856	0.229
C	-1.106	-3.422	0.108
C	0.170	-2.823	0.059
H	0.244	-1.739	-0.032
C	1.331	-3.583	0.125
C	1.234	-5.019	0.246
C	-0.019	-5.618	0.295
H	-2.221	-1.575	-0.052
H	-6.885	-2.517	0.018
H	-2.554	-6.539	0.370
C	2.448	-5.787	0.312
H	-5.015	-6.379	0.353
H	-0.091	-6.703	0.386
H	2.372	-6.870	0.403
C	3.664	-5.177	0.263
C	3.761	-3.750	0.143
H	4.744	-3.284	0.106
C	2.637	-2.984	0.077
H	2.706	-1.900	-0.014
H	4.577	-5.770	0.314
H	-4.688	-1.413	-0.078
C	-7.458	-5.156	0.294
H	-7.383	-6.236	0.118

H	-8.102	-4.739	-0.496
C	-8.110	-4.891	1.658
H	-7.515	-5.394	2.434
H	-8.060	-3.815	1.874
C	-9.564	-5.355	1.736
H	-9.966	-5.087	2.722
H	-10.158	-4.801	0.994
C	-9.737	-6.857	1.511
H	-10.777	-7.162	1.677
H	-9.467	-7.145	0.488
H	-9.102	-7.428	2.203

Table S182 Frequencies (cm^{-1}) of β-butyltetracene molecule, calculated at the M06-2X/aug-aug-cc-pVDZ level of theory.

28	40	41	71	100	122	147	157
190	206	252	263	281	305	324	363
377	407	426	466	477	485	496	503
545	558	595	631	639	642	729	748
758	761	772	779	782	786	790	819
835	859	875	885	893	906	914	922
936	944	950	969	992	996	1000	1016
1028	1094	1108	1118	1126	1153	1164	1174
1189	1196	1211	1220	1236	1265	1280	1287
1296	1314	1327	1338	1342	1355	1358	1380
1395	1397	1443	1454	1459	1467	1477	1482
1483	1487	1488	1504	1514	1591	1625	1629
1645	1688	1712	1727	3044	3055	3056	3059
3094	3099	3123	3134	3144	3186	3189	3192
3192	3193	3194	3197	3201	3210	3216	3228

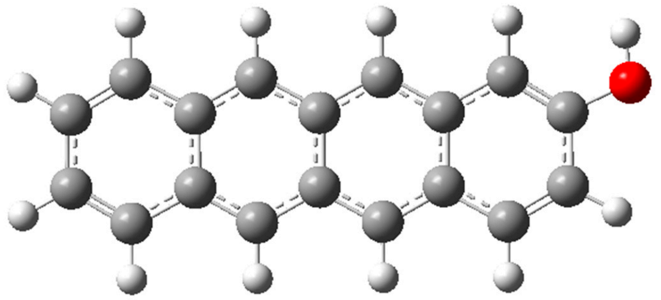

Figure S92 Visualization of the optimized structure of β-hydroxytetracene molecule, calculated at the M06-2X/aug-aug-cc-pVDZ level of theory.

Table S183 Geometry (Å) of β-hydroxytetracene molecule, calculated at the M06-2X/aug-aug-cc-pVDZ level of theory

Atom	x	y	z

C	-3.653	0.736	0.000
C	-2.475	1.420	0.000
C	-1.217	0.724	0.000
C	-1.225	-0.720	0.000
C	-2.493	-1.399	0.000
C	-3.661	-0.699	0.000
C	-0.003	1.398	0.000
C	-0.020	-1.413	0.000
C	1.218	-0.738	0.000
C	1.224	0.705	0.000
C	2.465	1.379	0.000
H	2.475	2.470	0.000
C	3.666	0.687	0.000
C	2.449	-1.431	0.000
H	0.005	2.489	0.000
H	-4.599	1.275	0.000
H	-2.464	2.510	0.000
H	-2.498	-2.489	0.000
H	-4.614	-1.226	0.000
H	-0.030	-2.503	0.000
H	2.438	-2.522	0.000
C	3.662	-0.758	0.000
C	4.921	-1.452	0.000
C	6.092	-0.755	0.000
H	4.922	-2.543	0.000
C	6.103	0.682	0.000
C	4.933	1.371	0.000
H	7.069	1.184	0.000
H	4.935	2.460	0.000
O	7.326	-1.333	0.000
H	7.232	-2.292	0.000

Table S184 Frequencies (cm^{-1}) of β-hydroxytetracene molecule, calculated at the M06-2X/aug-aug-cc-pVDZ level of theory.

47	84	132	143	181	242	268	302
302	365	392	404	425	473	475	482
485	499	535	550	594	632	635	643
746	751	759	770	779	779	789	833
834	850	863	885	897	914	916	940
944	980	995	1011	1013	1027	1120	1146
1153	1172	1185	1201	1208	1220	1269	1281
1287	1323	1344	1350	1362	1401	1448	1453
1488	1491	1503	1526	1595	1626	1639	1647
1689	1712	1729	3188	3192	3192	3193	3194
3197	3201	3205	3216	3228	3232	3873	

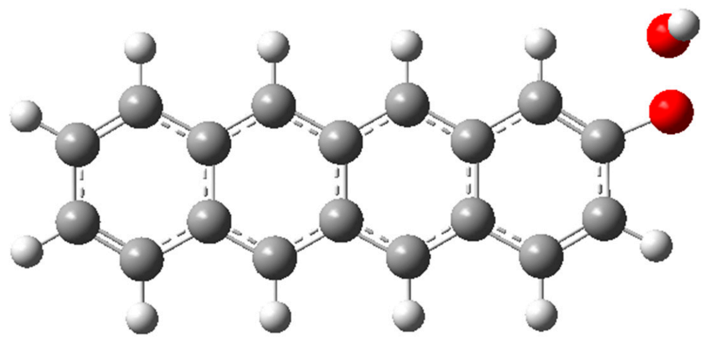

Figure S93 Visualization of the optimized structure of α-peroxytetracene molecule, calculated at the M06-2X/aug-aug-cc-pVDZ level of theory.

Table S185 Geometry (Å) of β-peroxytetracene molecule, calculated at the M06-2X/aug-aug-cc-pVDZ level of theory

Atom	x	y	z
C	-3.659	0.751	-0.003
C	-2.479	1.430	-0.006
C	-1.224	0.730	-0.003
C	-1.238	-0.714	0.003
C	-2.507	-1.389	0.007
C	-3.673	-0.685	0.003
C	-0.007	1.400	-0.006
C	-0.035	-1.411	0.007
C	1.205	-0.741	0.004
C	1.217	0.702	-0.003
C	2.461	1.371	-0.004
H	2.475	2.462	-0.007
C	3.658	0.673	-0.001
C	2.433	-1.439	0.006
H	0.005	2.491	-0.010
H	-4.604	1.292	-0.006
H	-2.465	2.520	-0.011
H	-2.515	-2.479	0.012
H	-4.628	-1.208	0.006
H	-0.048	-2.502	0.012
H	2.420	-2.530	0.011
C	3.650	-0.772	0.003
C	4.901	-1.483	0.004
C	6.064	-0.780	0.000
H	4.904	-2.568	0.019
C	6.094	0.657	0.002
C	4.928	1.351	0.000
H	7.062	1.156	0.005
H	4.938	2.441	0.000
O	7.328	-1.320	-0.003
O	7.279	-2.730	0.012

H	7.488	-2.943	-0.910

Table S186 Frequencies (cm^{-1}) of β-peroxytetracene molecule, calculated at the M06-2X/aug-aug-cc-pVDZ level of theory.

46	70	116	122	136	186	198	213
247	293	303	336	368	408	434	464
477	486	500	519	550	571	603	627
631	642	747	748	761	771	779	779
789	825	842	856	866	887	898	916
917	945	946	992	997	1004	1016	1030
1049	1125	1147	1154	1175	1180	1199	1222
1247	1280	1289	1322	1330	1343	1357	1394
1435	1447	1454	1486	1489	1505	1508	1596
1628	1641	1649	1692	1711	1728	3193	3194
3195	3197	3198	3202	3208	3216	3227	3232
3255	3803						

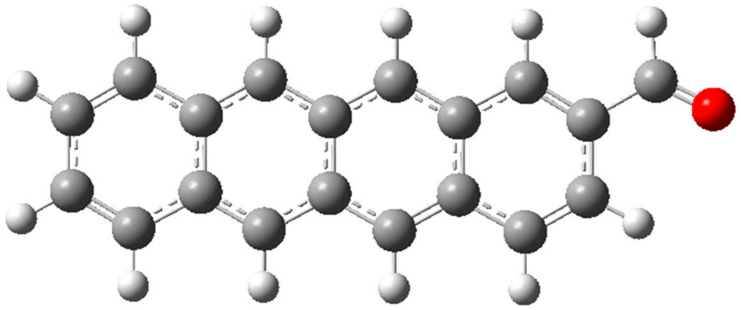

Figure S94 Visualization of the optimized structure of β-tetraldehyde molecule, calculated at the M06-2X/aug-aug-cc-pVDZ level of theory.

Table S187 Geometry (Å) of β-tetraldehyde molecule, calculated at the M06-2X/aug-aug-cc-pVDZ level of theory

Atom	x	y	z
C	-3.645	0.723	0.000
C	-2.470	1.412	0.000
C	-1.211	0.719	0.000
C	-1.217	-0.725	0.000
C	-2.481	-1.409	0.000
C	-3.651	-0.712	0.000
C	0.001	1.400	0.000
C	-0.009	-1.413	0.000
C	1.224	-0.731	0.000
C	1.231	0.712	0.000
C	2.469	1.394	0.000
H	2.472	2.485	0.000

**C**	3.674	0.709	0.000
**C**	2.457	-1.420	0.000
**H**	0.003	2.491	0.000
**H**	-4.593	1.259	0.000
**H**	-2.464	2.502	0.000
**H**	-2.481	-2.499	0.000
**H**	-4.603	-1.241	0.000
**H**	-0.013	-2.503	0.000
**H**	2.455	-2.510	0.000
**C**	3.665	-0.737	0.000
**C**	4.924	-1.424	0.000
**C**	6.102	-0.733	0.000
**H**	4.929	-2.516	0.000
**C**	6.113	0.706	0.000
**C**	4.942	1.395	0.000
**H**	7.078	1.210	0.000
**H**	4.938	2.484	0.000
**C**	7.379	-1.482	0.000
**O**	8.470	-0.961	0.000
**H**	7.276	-2.591	-0.001

Table S188 Frequencies (cm^{-1}) of β-tetraldehyde molecule, calculated at the M06-2X/aug-aug-cc-pVDZ level of theory.

40	81	99	117	170	180	209	264
270	294	321	362	394	420	460	479
488	500	520	542	579	602	629	639
662	751	760	770	774	786	786	791
819	831	861	882	884	907	913	929
948	958	972	998	1016	1022	1028	1041
1123	1148	1154	1173	1184	1196	1219	1241
1279	1286	1323	1339	1342	1351	1386	1407
1446	1453	1483	1487	1503	1510	1592	1626
1631	1642	1686	1708	1720	1823	2960	3185
3194	3194	3196	3197	3199	3203	3204	3218
3229	3232						

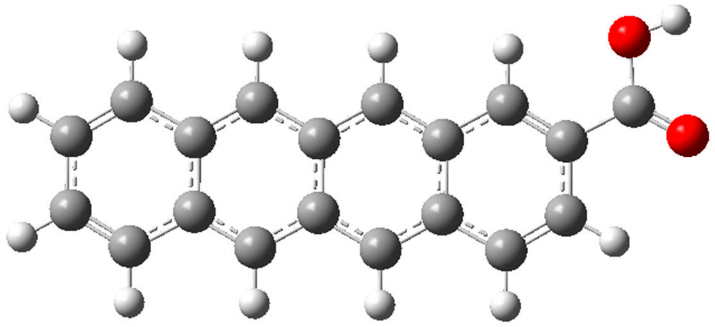

Figure S95 Visualization of the optimized structure of α-tetracenic acid molecule, calculated at the M06-2X/aug-aug-cc-pVDZ level of theory.

Table S189 Geometry (Å) of β-tetracenic acid molecule, calculated at the M06-2X/aug-aug-cc-pVDZ level of theory

Atom	x	y	z
C	-3.646	0.742	0.000
C	-2.467	1.424	0.000
C	-1.211	0.725	0.000
C	-1.225	-0.720	0.000
C	-2.494	-1.396	0.000
C	-3.660	-0.693	0.000
C	0.004	1.398	0.000
C	-0.023	-1.415	0.000
C	1.216	-0.740	0.000
C	1.231	0.703	0.000
C	2.472	1.377	0.000
H	2.482	2.468	0.000
C	3.673	0.684	0.000
C	2.443	-1.436	0.000
H	0.013	2.489	0.000
H	-4.591	1.284	0.000
H	-2.454	2.514	0.000
H	-2.501	-2.486	0.000
H	-4.615	-1.216	0.000
H	-0.033	-2.505	0.000
H	2.436	-2.527	0.000
C	3.657	-0.761	0.000
C	4.907	-1.465	0.000
C	6.089	-0.782	0.000
H	4.901	-2.554	0.000
C	6.110	0.656	0.000
C	4.946	1.357	0.000
H	7.077	1.154	0.000
H	4.955	2.446	0.000
C	7.397	-1.488	0.000
O	8.471	-0.936	0.000
O	7.282	-2.835	0.000
H	8.184	-3.185	0.000

Table S190 Frequencies (cm^{-1}) of β-tetracenic acid molecule, calculated at the M06-2X/aug-aug-cc-pVDZ level of theory.

41	58	83	108	121	176	196	221
264	291	316	340	393	404	437	477
486	499	516	520	530	566	580	618
630	640	641	738	748	760	771	775
779	790	795	816	848	863	886	886

911	913	937	950	969	971	998	1019
1021	1030	1107	1128	1155	1157	1176	1194
1216	1223	1240	1284	1290	1325	1329	1343
1353	1393	1399	1448	1457	1483	1489	1504
1516	1592	1627	1630	1643	1689	1708	1719
1842	3195	3196	3197	3198	3199	3203	3208
3217	3227	3228	3240	3826			
