# Supplementary material for: Substituent and Ring-Number Effects on the Kinetics of PAH + OH Reactions: A QSAR–DOE Approach with Tunneling Corrections
Source: Molecules. 2026 Jan 13;31(2):265. doi: 10.3390/molecules31020265 (PMC12843667; doi:10.3390/molecules31020265)
Supplement: Supplementary file 1 [file molecules-31-00265-s001.zip › Supporting Information - transition states.pdf]

Supporting information – transition states for

Substituent and Ring-Number Effects on the Kinetics of PAH + OH Reactions: A QSAR–DOE Approach with Tunneling Corrections

Cezary Parzych[1], Maciej Baradyn[2], Artur Ratkiewicz[2]*

Address: Institute of Chemistry, University of Bialystok, ul. Ciolkowskiego 1K, 15-245 Bialystok, Poland

[1] Doctoral School of University of Bialystok; 15-245 Bialystok, Ciołkowskiego 1K Street; Poland ; c.parzych@uwb.edu.pl
[2] Department of Physical Chemistry, University of Bialystok, Bialystok, Ciołkowskiego 1K Street; Poland

*Correspondence: artrat@uwb.edu.pl

Figure S1 Visualization of hydrogen abstraction transition state between hydroxyl radical and benzene molecule, calculated at the M06-2X/cc-pVTZ level of theory. 22

Table S1 Geometry (Å) of hydrogen abstraction transition state between hydroxyl radical and benzene molecule, calculated at the M06-2X/cc-pVDZ level of theory.......................... 22

Table S2 Frequencies (cm^{-1}) of hydrogen abstraction transition state between hydroxyl radical and benzene molecule, calculated at the M06-2X/cc-pVDZ level of theory............. 22

Figure S2 Visualization of hydrogen abstraction transition state between hydroxyl radical and bromobenzene molecule, calculated at the M06-2X/cc-pVDZ level of theory. 23

Table S3 Geometry (Å) of hydrogen abstraction transition state between hydroxyl radical and bromobenzene molecule, calculated at the M06-2X/cc-pVDZ level of theory 23

Table S4 Frequencies (cm^{-1}) of hydrogen abstraction transition state between hydroxyl radical and bromobenzene molecule, calculated at the M06-2X/cc-pVDZ level of theory.. 23

Figure S3 Visualization of hydrogen abstraction transition state between hydroxyl radical and chlorobenzene molecule, calculated at the M06-2X/cc-pVDZ level of theory. 24

Table S5 Geometry (Å) of hydrogen abstraction transition state between hydroxyl radical and chlorobenzene molecule, calculated at the M06-2X/cc-pVDZ level of theory 24

Table S6 Frequencies (cm^{-1}) of hydrogen abstraction transition state between hydroxyl radical and chlorobenzene molecule, calculated at the M06-2X/cc-pVDZ level of theory. . 24

Figure S4 Visualization of hydrogen abstraction transition state between hydroxyl radical and fluorobenzene molecule, calculated at the M06-2X/cc-pVDZ level of theory. 25

Table S7 Geometry (Å) of hydrogen abstraction transition state between hydroxyl radical and fluorobenzene molecule, calculated at the M06-2X/cc-pVDZ level of theory............... 25

Table S8 Frequencies (cm^{-1}) of hydrogen abstraction transition state between hydroxyl radical and fluorobenzene molecule, calculated at the M06-2X/cc-pVDZ level of theory... 25

Figure S5 Visualization of hydrogen abstraction transition state between hydroxyl radical and aminobenzene molecule, calculated at the M06-2X/cc-pVDZ level of theory. 26

Table S9 Geometry (Å) of hydrogen abstraction transition state between hydroxyl radical and aminobenzene molecule, calculated at the M06-2X/cc-pVDZ level of theory 26

Table S10 Frequencies (cm^{-1}) of hydrogen abstraction transition state between hydroxyl radical and aminobenzene molecule, calculated at the M06-2X/cc-pVDZ level of theory. . 26

Figure S6 Visualization of hydrogen abstraction transition state between hydroxyl radical and nitrobenzene molecule, calculated at the M06-2X/cc-pVDZ level of theory. 27

Table S11 Geometry (Å) of hydrogen abstraction transition state between hydroxyl radical and nitrobenzene molecule, calculated at the M06-2X/cc-pVDZ level of theory 27

Table S12 Frequencies (cm^{-1}) of hydrogen abstraction transition state between hydroxyl radical and aminobenzene molecule, calculated at the M06-2X/cc-pVDZ level of theory. . 27

Figure S7 Visualization of hydrogen abstraction transition state between hydroxyl radical and methylbenzene molecule, calculated at the M06-2X/cc-pVDZ level of theory. 28

Table S13 Geometry (Å) of hydrogen abstraction transition state between hydroxyl radical and methylbenzene molecule, calculated at the M06-2X/cc-pVDZ level of theory 28

Table S14 Frequencies (cm^{-1}) of hydrogen abstraction transition state between hydroxyl radical and methylbenzene molecule, calculated at the M06-2X/cc-pVDZ level of theory. 28

Figure S8 Visualization of hydrogen abstraction transition state between hydroxyl radical and ethylbenzene molecule, calculated at the M06-2X/cc-pVDZ level of theory................. 29

Table S15 Geometry (Å) of hydrogen abstraction transition state between hydroxyl radical and ethylbenzene molecule, calculated at the M06-2X/cc-pVDZ level of theory................. 29

Table S16 Frequencies (cm^{-1}) of hydrogen abstraction transition state between hydroxyl radical and ethylbenzene molecule, calculated at the M06-2X/cc-pVDZ level of theory..... 29

Figure S9 Visualization of hydrogen abstraction transition state between hydroxyl radical and propylbenzene molecule, calculated at the M06-2X/cc-pVDZ level of theory.............. 30

Table S17 Geometry (Å) of hydrogen abstraction transition state between hydroxyl radical and propylbenzene molecule, calculated at the M06-2X/cc-pVDZ level of theory.............. 30

Table S18 Frequencies (cm^{-1}) of hydrogen abstraction transition state between hydroxyl radical and propylbenzene molecule, calculated at the M06-2X/cc-pVDZ level of theory.. 31

Figure S10 Visualization of hydrogen abstraction transition state between hydroxyl radical and butylbenzene molecule, calculated at the M06-2X/cc-pVDZ level of theory. 31

Table S19 Geometry (Å) of hydrogen abstraction transition state between hydroxyl radical and butylbenzene molecule, calculated at the M06-2X/cc-pVDZ level of theory 31

Table S20 Frequencies (cm^{-1}) of hydrogen abstraction transition state between hydroxyl radical and butylbenzene molecule, calculated at the M06-2X/cc-pVDZ level of theory. ... 32

Figure S11 Visualization of hydrogen abstraction transition state between hydroxyl radical and hydroxybenzene molecule, calculated at the M06-2X/cc-pVDZ level of theory........... 32

Table S21 Geometry (Å) of hydrogen abstraction transition state between hydroxyl radical and hydroxybenzene molecule, calculated at the M06-2X/cc-pVDZ level of theory........... 32

Table S22 Frequencies (cm^{-1}) of hydrogen abstraction transition state between hydroxyl radical and hydroxybenzene molecule, calculated at the M06-2X/cc-pVDZ level of theory. 33

Figure S12 Visualization of hydrogen abstraction transition state between hydroxyl radical and peroxybenzene molecule, calculated at the M06-2X/cc-pVDZ level of theory. 33

Table S23 Geometry (Å) of hydrogen abstraction transition state between hydroxyl radical and peroxybenzene molecule, calculated at the M06-2X/cc-pVDZ level of theory 33

Table S24 Frequencies (cm^{-1}) of hydrogen abstraction transition state between hydroxyl radical and peroxybenzene molecule, calculated at the M06-2X/cc-pVDZ level of theory. 34

Figure S13 Visualization of hydrogen abstraction transition state between hydroxyl radical and benzaldehyde molecule, calculated at the M06-2X/cc-pVDZ level of theory. 34

Table S25 Geometry (Å) of hydrogen abstraction transition state between hydroxyl radical and benzaldehyde molecule, calculated at the M06-2X/cc-pVDZ level of theory 34

Table S26 Frequencies (cm^{-1}) of hydrogen abstraction transition state between hydroxyl radical and benzaldehyde molecule, calculated at the M06-2X/cc-pVDZ level of theory. .. 35

Figure S14 Visualization of hydrogen abstraction transition state between hydroxyl radical and benzoic acid molecule, calculated at the M06-2X/cc-pVDZ level of theory. 35

Table S27 Geometry (Å) of hydrogen abstraction transition state between hydroxyl radical and benzoic acid molecule, calculated at the M06-2X/cc-pVDZ level of theory 35

Table S28 Frequencies (cm^{-1}) of hydrogen abstraction transition state between hydroxyl radical and benzaldehyde molecule, calculated at the M06-2X/cc-pVDZ level of theory. .. 36

Figure S15 Visualization of hydrogen abstraction transition state between hydroxyl radical and naphtalene molecule, calculated at the M06-2X/cc-pVDZ level of theory..................... 36

Table S29 Geometry (Å) of hydrogen abstraction transition state between hydroxyl radical and naphtalene molecule, calculated at the M06-2X/cc-pVDZ level of theory..................... 36

Table S30 Frequencies (cm^{-1}) of hydrogen abstraction transition state between hydroxyl radical and naphtalene molecule, calculated at the M06-2X/cc-pVDZ level of theory. 37

Figure S16 Visualization of hydrogen abstraction transition state between hydroxyl radical and α-bromonaphtalene molecule, calculated at the M06-2X/cc-pVDZ level of theory...... 37

Table S31 Geometry (Å) of hydrogen abstraction transition state between hydroxyl radical and α-bromonaphtalene molecule, calculated at the M06-2X/cc-pVDZ level of theory...... 37

Table S32 Frequencies (cm^{-1}) of hydrogen abstraction transition state between hydroxyl radical and α-bromonaphtalene molecule, calculated at the M06-2X/cc-pVDZ level of theory. 38

Figure S17 Visualization of hydrogen abstraction transition state between hydroxyl radical and α-chloronaphtalene molecule, calculated at the M06-2X/cc-pVDZ level of theory. 38

Table S33 Geometry (Å) of hydrogen abstraction transition state between hydroxyl radical and α-chloronaphtalene molecule, calculated at the M06-2X/cc-pVDZ level of theory 38

Table S34 Frequencies (cm^{-1}) of hydrogen abstraction transition state between hydroxyl radical and α-chloronaphtalene molecule, calculated at the M06-2X/cc-pVDZ level of theory. 39

Figure S18 Visualization of hydrogen abstraction transition state between hydroxyl radical and α-fluoronaphtalene molecule, calculated at the M06-2X/cc-pVDZ level of theory....... 39

Table S35 Geometry (Å) of hydrogen abstraction transition state between hydroxyl radical and α-fluoronaphtalene molecule, calculated at the M06-2X/cc-pVDZ level of theory 40

Table S36 Frequencies (cm^{-1}) of hydrogen abstraction transition state between hydroxyl radical and α-fluoronaphtalene molecule, calculated at the M06-2X/cc-pVDZ level of theory. 40

Figure S19 Visualization of hydrogen abstraction transition state between hydroxyl radical and α-aminonaphtalene molecule, calculated at the M06-2X/cc-pVDZ level of theory. 41

Table S37 Geometry (Å) of hydrogen abstraction transition state between hydroxyl radical and α-aminonaphtalene molecule, calculated at the M06-2X/cc-pVDZ level of theory 41

Table S38 Frequencies (cm-1) of hydrogen abstraction transition state between hydroxyl radical and α-aminonaphtalene molecule, calculated at the M06-2X/cc-pVDZ level of theory. 41

Figure S20 Visualization of hydrogen abstraction transition state between hydroxyl radical and α-nitronaphtalene molecule, calculated at the M06-2X/cc-pVDZ level of theory. 42

Table S39 Geometry (Å) of hydrogen abstraction transition state between hydroxyl radical and α-nitronaphtalene molecule, calculated at the M06-2X/cc-pVDZ level of theory 42

Table S40 Frequencies (cm^{-1}) of hydrogen abstraction transition state between hydroxyl radical and α-nitronaphtalene molecule, calculated at the M06-2X/cc-pVDZ level of theory. 42

Figure S21 Visualization of hydrogen abstraction transition state between hydroxyl radical and α-methylnaphtalene molecule, calculated at the M06-2X/cc-pVDZ level of theory. 43

Table S41 Geometry (Å) of hydrogen abstraction transition state between hydroxyl radical and α-methylnaphtalene molecule, calculated at the M06-2X/cc-pVDZ level of theory 43

Table S42 Frequencies (cm^{-1}) of hydrogen abstraction transition state between hydroxyl radical and α-methylnaphtalene molecule, calculated at the M06-2X/cc-pVDZ level of theory. 44

Figure S22 Visualization of hydrogen abstraction transition state between hydroxyl radical and α-ethylnaphtalene molecule, calculated at the M06-2X/cc-pVDZ level of theory......... 44

Table S43 Geometry (Å) of hydrogen abstraction transition state between hydroxyl radical and α-ethylnaphtalene molecule, calculated at the M06-2X/cc-pVDZ level of theory......... 44

Table S44 Frequencies (cm^{-1}) of hydrogen abstraction transition state between hydroxyl radical and α-ethylnaphtalene molecule, calculated at the M06-2X/cc-pVDZ level of theory. 45

Figure S23 Visualization of hydrogen abstraction transition state between hydroxyl radical and α-propylnaphtalene molecule, calculated at the M06-2X/cc-pVDZ level of theory...... 45

Table S45 Geometry (Å) of hydrogen abstraction transition state between hydroxyl radical and α-propylnaphtalene molecule, calculated at the M06-2X/cc-pVDZ level of theory...... 45

Table S46 Frequencies (cm^{-1}) of hydrogen abstraction transition state between hydroxyl radical and α-propylnaphtalene molecule, calculated at the M06-2X/cc-pVDZ level of theory. 46

Figure S24 Visualization of hydrogen abstraction transition state between hydroxyl radical and α-butylnaphtalene molecule, calculated at the M06-2X/cc-pVDZ level of theory. 47

Table S47 Geometry (Å) of hydrogen abstraction transition state between hydroxyl radical and α-butylnaphtalene molecule, calculated at the M06-2X/cc-pVDZ level of theory 47

Table S48 Frequencies (cm^{-1}) of hydrogen abstraction transition state between hydroxyl radical and α-butylnaphtalene molecule, calculated at the M06-2X/cc-pVDZ level of theory. 48

Figure S25 Visualization of hydrogen abstraction transition state between hydroxyl radical and α-hydroxynaphtalene molecule, calculated at the M06-2X/cc-pVDZ level of theory... 48

Table S49 Geometry (Å) of hydrogen abstraction transition state between hydroxyl radical and α-hydroxynaphtalene molecule, calculated at the M06-2X/cc-pVDZ level of theory... 48

Table S50 Frequencies (cm^{-1}) of hydrogen abstraction transition state between hydroxyl radical and α-hydroxynaphtalene molecule, calculated at the M06-2X/cc-pVDZ level of theory. 49

Figure S26 Visualization of hydrogen abstraction transition state between hydroxyl radical and α-peroxynaphtalene molecule, calculated at the M06-2X/cc-pVDZ level of theory. 49

Table S51 Geometry (Å) of hydrogen abstraction transition state between hydroxyl radical and α-peroxynaphtalene molecule, calculated at the M06-2X/cc-pVDZ level of theory 49

Table S52 Frequencies (cm^{-1}) of hydrogen abstraction transition state between hydroxyl radical and α-peroxynaphtalene molecule, calculated at the M06-2X/cc-pVDZ level of theory. 50

Figure S27 Visualization of hydrogen abstraction transition state between hydroxyl radical and α-naphtaldehyde molecule, calculated at the M06-2X/cc-pVDZ level of theory........... 50

Table S53 Geometry (Å) of hydrogen abstraction transition state between hydroxyl radical and α-naphtaldehyde molecule, calculated at the M06-2X/cc-pVDZ level of theory........... 51

Table S54 Frequencies (cm^{-1}) of hydrogen abstraction transition state between hydroxyl radical and α-naphtaldehyde molecule, calculated at the M06-2X/cc-pVDZ level of theory. 51

Figure S28 Visualization of hydrogen abstraction transition state between hydroxyl radical and α-naphtalenic acid molecule, calculated at the M06-2X/cc-pVDZ level of theory. 52

Table S55 Geometry (Å) of hydrogen abstraction transition state between hydroxyl radical and α-naphtalenic aicd molecule, calculated at the M06-2X/cc-pVDZ level of theory 52

Table S56 Frequencies (cm^{-1}) of hydrogen abstraction transition state between hydroxyl radical and α-naphtalenic acid molecule, calculated at the M06-2X/cc-pVDZ level of theory. 52

Figure S29 Visualization of hydrogen abstraction transition state between hydroxyl radical and β-bromonaphtalene molecule, calculated at the M06-2X/cc-pVDZ level of theory. 53

Table S57 Geometry (Å) of hydrogen abstraction transition state between hydroxyl radical and β-bromonaphtalene molecule, calculated at the M06-2X/cc-pVDZ level of theory 53

Table S58 Frequencies (cm^{-1}) of hydrogen abstraction transition state between hydroxyl radical and β-bromonaphtalene molecule, calculated at the M06-2X/cc-pVDZ level of theory. 54

Figure S30 Visualization of hydrogen abstraction transition state between hydroxyl radical and β-chloronaphtalene molecule, calculated at the M06-2X/cc-pVDZ level of theory....... 54

Table S59 Geometry (Å) of hydrogen abstraction transition state between hydroxyl radical and β-chloronaphtalene molecule, calculated at the M06-2X/cc-pVDZ level of theory....... 54

Table S60 Frequencies (cm^{-1}) of hydrogen abstraction transition state between hydroxyl radical and β-chloronaphtalene molecule, calculated at the M06-2X/cc-pVDZ level of theory. 55

Figure S31 Visualization of hydrogen abstraction transition state between hydroxyl radical and β-fluoronaphtalene molecule, calculated at the M06-2X/cc-pVDZ level of theory. 55

Table S61 Geometry (Å) of hydrogen abstraction transition state between hydroxyl radical and β-fluoronaphtalene molecule, calculated at the M06-2X/cc-pVDZ level of theory 55

Table S62 Frequencies (cm^{-1}) of hydrogen abstraction transition state between hydroxyl radical and β-fluoronaphtalene molecule, calculated at the M06-2X/cc-pVDZ level of theory. 56

Figure S32 Visualization of hydrogen abstraction transition state between hydroxyl radical and β-aminonaphtalene molecule, calculated 56

at the M06-2X/cc-pVDZ level of theory. 56

Table S63 Geometry (Å) of hydrogen abstraction transition state between hydroxyl radical and β-aminonaphtalene molecule, calculated at the M06-2X/cc-pVDZ level of theory....... 56

Table S64 Frequencies (cm^{-1}) of hydrogen abstraction transition state between hydroxyl radical and β-aminonaphtalene molecule, calculated at the M06-2X/cc-pVDZ level of theory. 57

Figure S33 Visualization of hydrogen abstraction transition state between hydroxyl radical and β-nitronaphtalene molecule, calculated at the M06-2X/cc-pVDZ level of theory. 57

Table S65 Geometry (Å) of hydrogen abstraction transition state between hydroxyl radical and β-nitronaphtalene molecule, calculated at the M06-2X/cc-pVDZ level of theory 57

Table S66 Frequencies (cm^{-1}) of hydrogen abstraction transition state between hydroxyl radical and β-nitronaphtalene molecule, calculated at the M06-2X/cc-pVDZ level of theory. 58

Figure S34 Visualization of hydrogen abstraction transition state between hydroxyl radical and β-methylnaphtalene molecule, calculated at the M06-2X/cc-pVDZ level of theory. 58

Table S67 Geometry (Å) of hydrogen abstraction transition state between hydroxyl radical and β-methylnaphtalene molecule, calculated at the M06-2X/cc-pVDZ level of theory 59

Table S68 Frequencies (cm^{-1}) of hydrogen abstraction transition state between hydroxyl radical and β-methylnaphtalene molecule, calculated at the M06-2X/cc-pVDZ level of theory. 59

Figure S35 Visualization of hydrogen abstraction transition state between hydroxyl radical and β-ethylnaphtalene molecule, calculated at the M06-2X/cc-pVDZ level of theory. 60

Table S69 Geometry (Å) of hydrogen abstraction transition state between hydroxyl radical and β-ethylnaphtalene molecule, calculated at the M06-2X/cc-pVDZ level of theory 60

Table S70 Frequencies (cm^{-1}) of hydrogen abstraction transition state between hydroxyl radical and β-ethylnaphtalene molecule, calculated at the M06-2X/cc-pVDZ level of theory. 61

Figure S36 Visualization of hydrogen abstraction transition state between hydroxyl radical and β-propylnaphtalene molecule, calculated at the M06-2X/cc-pVDZ level of theory. 61

Table S71 Geometry (Å) of hydrogen abstraction transition state between hydroxyl radical and β-propylnaphtalene molecule, calculated at the M06-2X/cc-pVDZ level of theory 61

Table S72 Frequencies (cm^{-1}) of hydrogen abstraction transition state between hydroxyl radical and β-propylnaphtalene molecule, calculated at the M06-2X/cc-pVDZ level of theory. 62

Figure S37 Visualization of hydrogen abstraction transition state between hydroxyl radical and β-butylnaphtalene molecule, calculated at the M06-2X/cc-pVDZ level of theory......... 62

Table S73 Geometry (Å) of hydrogen abstraction transition state between hydroxyl radical and β-butylnaphtalene molecule, calculated at the M06-2X/cc-pVDZ level of theory......... 63

Table S74 Frequencies (cm^{-1}) of hydrogen abstraction transition state between hydroxyl radical and β-butylnaphtalene molecule, calculated at the M06-2X/cc-pVDZ level of theory. 63

Figure S38 Visualization of hydrogen abstraction transition state between hydroxyl radical and β-hydroxynaphtalene molecule, calculated at the M06-2X/cc-pVDZ level of theory... 64

Table S75 Geometry (Å) of hydrogen abstraction transition state between hydroxyl radical and β-hydroxynaphtalene molecule, calculated at the M06-2X/cc-pVDZ level of theory ... 64

Table S76 Frequencies (cm^{-1}) of hydrogen abstraction transition state between hydroxyl radical and β-hydroxynaphtalene molecule, calculated at the M06-2X/cc-pVDZ level of theory. 65

Figure S39 Visualization of hydrogen abstraction transition state between hydroxyl radical and β-peroxynaphtalene molecule, calculated at the M06-2X/cc-pVDZ level of theory...... 65

Table S77 Geometry (Å) of hydrogen abstraction transition state between hydroxyl radical and β-peroxynaphtalene molecule, calculated at the M06-2X/cc-pVDZ level of theory...... 65

Table S78 Frequencies (cm^{-1}) of hydrogen abstraction transition state between hydroxyl radical and β-peroxynaphtalene molecule, calculated at the M06-2X/cc-pVDZ level of theory. 66

Figure S40 Visualization of hydrogen abstraction transition state between hydroxyl radical and β-naphtaldehyde molecule, calculated at the M06-2X/cc-pVDZ level of theory. 66

Table S79 Geometry (Å) of hydrogen abstraction transition state between hydroxyl radical and β-naphtaldehyde molecule, calculated at the M06-2X/cc-pVDZ level of theory 66

Table S80 Frequencies (cm^{-1}) of hydrogen abstraction transition state between hydroxyl radical and β-naphtaldehyde molecule, calculated at the M06-2X/cc-pVDZ level of theory. 67

Figure S41 Visualization of hydrogen abstraction transition state between hydroxyl radical and β-naphtalenic acid molecule, calculated at the M06-2X/cc-pVDZ level of theory......... 67

Table S81 Geometry (Å) of hydrogen abstraction transition state between hydroxyl radical and β-naphtalenic aicd molecule, calculated at the M06-2X/cc-pVDZ level of theory......... 67

Table S82 Frequencies (cm^{-1}) of hydrogen abstraction transition state between hydroxyl radical and β-naphtalenic acid molecule, calculated at the M06-2X/cc-pVDZ level of theory. 68

Figure S42 Visualization of hydrogen abstraction transition state between hydroxyl radical and antracene molecule, calculated at the M06-2X/cc-pVDZ level of theory. 68

Table S83 Geometry (Å) of hydrogen abstraction transition state between hydroxyl radical and antracene molecule, calculated at the M06-2X/cc-pVDZ level of theory 68

Table S84 Frequencies (cm^{-1}) of hydrogen abstraction transition state between hydroxyl radical and antracene molecule, calculated at the M06-2X/cc-pVDZ level of theory. 69

Figure S43 Visualization of hydrogen abstraction transition state between hydroxyl radical and α-bromoantracene molecule, calculated at the M06-2X/cc-pVDZ level of theory......... 70

Table S85 Geometry (Å) of hydrogen abstraction transition state between hydroxyl radical and α-bromoantracene molecule, calculated at the M06-2X/cc-pVDZ level of theory......... 70

Table S86 Frequencies (cm^{-1}) of hydrogen abstraction transition state between hydroxyl radical and α-bromoantracene molecule, calculated at the M06-2X/cc-pVDZ level of theory. 71

Figure S44 Visualization of hydrogen abstraction transition state between hydroxyl radical and α-chloroantracene molecule, calculated at the M06-2X/cc-pVDZ level of theory. 71

Table S87 Geometry (Å) of hydrogen abstraction transition state between hydroxyl radical and α-chloroantracene molecule, calculated at the M06-2X/cc-pVDZ level of theory 71

Table S88 Frequencies (cm^{-1}) of hydrogen abstraction transition state between hydroxyl radical and α-chloroantracene molecule, calculated at the M06-2X/cc-pVDZ level of theory. 72

Figure S45 Visualization of hydrogen abstraction transition state between hydroxyl radical and α-fluoroantracene molecule, calculated at the M06-2X/cc-pVDZ level of theory. 72

Table S89 Geometry (Å) of hydrogen abstraction transition state between hydroxyl radical and α-fluoroantracene molecule, calculated at the M06-2X/cc-pVDZ level of theory 72

Table S90 Frequencies (cm^{-1}) of hydrogen abstraction transition state between hydroxyl radical and α-fluoroantracene molecule, calculated at the M06-2X/cc-pVDZ level of theory. 73

Figure S46 Visualization of hydrogen abstraction transition state between hydroxyl radical and α-aminoantracene molecule, calculated at the M06-2X/cc-pVDZ level of theory. 73

Table S91 Geometry (Å) of hydrogen abstraction transition state between hydroxyl radical and α-aminoantracene molecule, calculated at the M06-2X/cc-pVDZ level of theory 74

Table S92 Frequencies (cm^{-1}) of hydrogen abstraction transition state between hydroxyl radical and α-aminoantracene molecule, calculated at the M06-2X/cc-pVDZ level of theory. 74

Figure S47 Visualization of hydrogen abstraction transition state between hydroxyl radical and α-nitroantracene molecule, calculated at the M06-2X/cc-pVDZ level of theory. 75

Table S93 Geometry (Å) of hydrogen abstraction transition state between hydroxyl radical and α-nitroantracene molecule, calculated at the M06-2X/cc-pVDZ level of theory 75

Table S94 Frequencies (cm^{-1}) of hydrogen abstraction transition state between hydroxyl radical and α-nitroantracene molecule, calculated at the M06-2X/cc-pVDZ level of theory. 76

Figure S48 Visualization of hydrogen abstraction transition state between hydroxyl radical and α-methylantracene molecule, calculated at the M06-2X/cc-pVDZ level of theory. 76

Table S95 Geometry (Å) of hydrogen abstraction transition state between hydroxyl radical and α-methylantracene molecule, calculated at the M06-2X/cc-pVDZ level of theory 76

Table S96 Frequencies (cm^{-1}) of hydrogen abstraction transition state between hydroxyl radical and α-methylantracene molecule, calculated at the M06-2X/cc-pVDZ level of theory. 77

Figure S49 Visualization of hydrogen abstraction transition state between hydroxyl radical and α-ethylantracene molecule, calculated at the M06-2X/cc-pVDZ level of theory. 77

Table S97 Geometry (Å) of hydrogen abstraction transition state between hydroxyl radical and α-ethylantracene molecule, calculated at the M06-2X/cc-pVDZ level of theory 77

Table S98 Frequencies (cm^{-1}) of hydrogen abstraction transition state between hydroxyl radical and α-ethylantracene molecule, calculated at the M06-2X/cc-pVDZ level of theory. 78

Figure S50 Visualization of hydrogen abstraction transition state between hydroxyl radical and α-propylantracene molecule, calculated at the M06-2X/cc-pVDZ level of theory. 79

Table S99 Geometry (Å) of hydrogen abstraction transition state between hydroxyl radical and α-propylantracene molecule, calculated at the M06-2X/cc-pVDZ level of theory 79

Table S100 Frequencies (cm^{-1}) of hydrogen abstraction transition state between hydroxyl radical and α-propylantracene molecule, calculated at the M06-2X/cc-pVDZ level of theory. 80

Figure S51 Visualization of hydrogen abstraction transition state between hydroxyl radical and α-butylantracene molecule, calculated at the M06-2X/cc-pVDZ level of theory. 80

Table S101 Geometry (Å) of hydrogen abstraction transition state between hydroxyl radical and α-butylantracene molecule, calculated at the M06-2X/cc-pVDZ level of theory 81

Table S102 Frequencies (cm^{-1}) of hydrogen abstraction transition state between hydroxyl radical and α-butylantracene molecule, calculated at the M06-2X/cc-pVDZ level of theory. 81

Figure S52 Visualization of hydrogen abstraction transition state between hydroxyl radical and α-hydroxyantracene molecule, calculated at the M06-2X/cc-pVDZ level of theory. 82

Table S103 Geometry (Å) of hydrogen abstraction transition state between hydroxyl radical and α-hydroxyantracene molecule, calculated at the M06-2X/cc-pVDZ level of theory 82

Table S104 Frequencies (cm^{-1}) of hydrogen abstraction transition state between hydroxyl radical and α-hydroxyantracene molecule, calculated at the M06-2X/cc-pVDZ level of theory. 83

Figure S53 Visualization of hydrogen abstraction transition state between hydroxyl radical and α-peroxyantracene molecule, calculated at the M06-2X/cc-pVDZ level of theory. 83

Table S105 Geometry (Å) of hydrogen abstraction transition state between hydroxyl radical and α-peroxyantracene molecule, calculated at the M06-2X/cc-pVDZ level of theory 83
Table S106 Frequencies (cm^{-1}) of hydrogen abstraction transition state between hydroxyl radical and α-peroxyantracene molecule, calculated at the M06-2X/cc-pVDZ level of theory. 84
Figure S54 Visualization of hydrogen abstraction transition state between hydroxyl radical and α-antraldehyde molecule, calculated at the M06-2X/cc-pVDZ level of theory. 85
Table S107 Geometry (Å) of hydrogen abstraction transition state between hydroxyl radical and α-antraldehyde molecule, calculated at the M06-2X/cc-pVDZ level of theory. 85
Table S108 Frequencies (cm^{-1}) of hydrogen abstraction transition state between hydroxyl radical and α-antraldehyde molecule, calculated at the M06-2X/cc-pVDZ level of theory. 86
Figure S55 Visualization of hydrogen abstraction transition state between hydroxyl radical and α-antracenic acid molecule, calculated at the M06-2X/cc-pVDZ level of theory. 86
Table S109 Geometry (Å) of hydrogen abstraction transition state between hydroxyl radical and α-antracenic acid molecule, calculated at the M06-2X/cc-pVDZ level of theory 86
Table S110 Frequencies (cm^{-1}) of hydrogen abstraction transition state between hydroxyl radical and α-antracenic acid molecule, calculated at the M06-2X/cc-pVDZ level of theory. 87
Figure S56 Visualization of hydrogen abstraction transition state between hydroxyl radical and β-bromoantracene molecule, calculated at the M06-2X/cc-pVDZ level of theory. 87
Table S111 Geometry (Å) of hydrogen abstraction transition state between hydroxyl radical and β-bromoantracene molecule, calculated at the M06-2X/cc-pVDZ level of theory 87
Table S112 Frequencies (cm^{-1}) of hydrogen abstraction transition state between hydroxyl radical and β-bromoantracene molecule, calculated at the M06-2X/cc-pVDZ level of theory. 88
Figure S57 Visualization of hydrogen abstraction transition state between hydroxyl radical and β-chloroantracene molecule, calculated at the M06-2X/cc-pVDZ level of theory. 89
Table S113 Geometry (Å) of hydrogen abstraction transition state between hydroxyl radical and β-chloroantracene molecule, calculated at the M06-2X/cc-pVDZ level of theory 89
Table S114 Frequencies (cm^{-1}) of hydrogen abstraction transition state between hydroxyl radical and β-chloroantracene molecule, calculated at the M06-2X/cc-pVDZ level of theory. 89

Figure S58 Visualization of hydrogen abstraction transition state between hydroxyl radical and β-fluoroantracene molecule, calculated at the M06-2X/cc-pVDZ level of theory.......... 90
Table S115 Geometry (Å) of hydrogen abstraction transition state between hydroxyl radical and β-fluoroantracene molecule, calculated at the M06-2X/cc-pVDZ level of theory 90
Table S116 Frequencies (cm^{-1}) of hydrogen abstraction transition state between hydroxyl radical and β-fluoroantracene molecule, calculated at the M06-2X/cc-pVDZ level of theory. 91
Figure S59 Visualization of hydrogen abstraction transition state between hydroxyl radical and β-aminoantracene molecule, calculated at the M06-2X/cc-pVDZ level of theory. 91
Table S117 Geometry (Å) of hydrogen abstraction transition state between hydroxyl radical and β-aminoantracene molecule, calculated at the M06-2X/cc-pVDZ level of theory 91
Table S118 Frequencies (cm^{-1}) of hydrogen abstraction transition state between hydroxyl radical and β-aminoantracene molecule, calculated at the M06-2X/cc-pVDZ level of theory. 92
Figure S60 Visualization of hydrogen abstraction transition state between hydroxyl radical and β-nitroantracene molecule, calculated at the M06-2X/cc-pVDZ level of theory. 92
Table S119 Geometry (Å) of hydrogen abstraction transition state between hydroxyl radical and β-nitroantracene molecule, calculated at the M06-2X/cc-pVDZ level of theory93
Table S120 Frequencies (cm^{-1}) of hydrogen abstraction transition state between hydroxyl radical and β-nitroantracene molecule, calculated at the M06-2X/cc-pVDZ level of theory. 93
Figure S61 Visualization of hydrogen abstraction transition state between hydroxyl radical and β-methylantracene molecule, calculated at the M06-2X/cc-pVDZ level of theory. 94
Table S121 Geometry (Å) of hydrogen abstraction transition state between hydroxyl radical and β-methylantracene molecule, calculated at the M06-2X/cc-pVDZ level of theory 94
Table S122 Frequencies (cm^{-1}) of hydrogen abstraction transition state between hydroxyl radical and β-methylantracene molecule, calculated at the M06-2X/cc-pVDZ level of theory. 95
Figure S62 Visualization of hydrogen abstraction transition state between hydroxyl radical and β-ethylantracene molecule, calculated at the M06-2X/cc-pVDZ level of theory. 95
Table S123 Geometry (Å) of hydrogen abstraction transition state between hydroxyl radical and β-ethylantracene molecule, calculated at the M06-2X/cc-pVDZ level of theory 95

Table S124 Frequencies (cm^{-1}) of hydrogen abstraction transition state between hydroxyl radical and β-ethylantracene molecule, calculated at the M06-2X/cc-pVDZ level of theory. 96

Figure S63 Visualization of hydrogen abstraction transition state between hydroxyl radical and β-propylantracene molecule, calculated at the M06-2X/cc-pVDZ level of theory. 96

Table S125 Geometry (Å) of hydrogen abstraction transition state between hydroxyl radical and β-propylantracene molecule, calculated at the M06-2X/cc-pVDZ level of theory 97

Table S126 Frequencies (cm^{-1}) of hydrogen abstraction transition state between hydroxyl radical and β-propylantracene molecule, calculated at the M06-2X/cc-pVDZ level of theory. 97

Figure S64 Visualization of hydrogen abstraction transition state between hydroxyl radical and β-butylantracene molecule, calculated at the M06-2X/cc-pVDZ level of theory. 98

Table S127 Geometry (Å) of hydrogen abstraction transition state between hydroxyl radical and β-butylantracene molecule, calculated at the M06-2X/cc-pVDZ level of theory 98

Table S128 Frequencies (cm^{-1}) of hydrogen abstraction transition state between hydroxyl radical and β-butylantracene molecule, calculated at the M06-2X/cc-pVDZ level of theory. 99

Figure S65 Visualization of hydrogen abstraction transition state between hydroxyl radical and β-hydroxyantracene molecule, calculated at the M06-2X/cc-pVDZ level of theory. .. 100

Table S129 Geometry (Å) of hydrogen abstraction transition state between hydroxyl radical and β-hydroxyantracene molecule, calculated at the M06-2X/cc-pVDZ level of theory 100

Table S130 Frequencies (cm^{-1}) of hydrogen abstraction transition state between hydroxyl radical and β-hydroxyantracene molecule, calculated at the M06-2X/cc-pVDZ level of theory. 101

Figure S66 Visualization of hydrogen abstraction transition state between hydroxyl radical and β-peroxyantracene molecule, calculated at the M06-2X/cc-pVDZ level of theory. 101

Table S131 Geometry (Å) of hydrogen abstraction transition state between hydroxyl radical and β-peroxyantracene molecule, calculated at the M06-2X/cc-pVDZ level of theory 101

Table S132 Frequencies (cm^{-1}) of hydrogen abstraction transition state between hydroxyl radical and β-peroxyantracene molecule, calculated at the M06-2X/cc-pVDZ level of theory. 102

Figure S67 Visualization of hydrogen abstraction transition state between hydroxyl radical and β-antraldehyde molecule, calculated at the M06-2X/cc-pVDZ level of theory. 102

Table S133 Geometry (Å) of hydrogen abstraction transition state between hydroxyl radical and β-antraldehyde molecule, calculated at the M06-2X/cc-pVDZ level of theory102

Table S134 Frequencies (cm^{-1}) of hydrogen abstraction transition state between hydroxyl radical and β-antraldehyde molecule, calculated at the M06-2X/cc-pVDZ level of theory. 103

Figure S68 Visualization of hydrogen abstraction transition state between hydroxyl radical and β-antracenic acid molecule, calculated at the M06-2X/cc-pVDZ level of theory. 104

Table S135 Geometry (Å) of hydrogen abstraction transition state between hydroxyl radical and β-antracenic acid molecule, calculated at the M06-2X/cc-pVDZ level of theory 104

Table S136 Frequencies (cm^{-1}) of hydrogen abstraction transition state between hydroxyl radical and β-antracenic acid molecule, calculated at the M06-2X/cc-pVDZ level of theory. 105

Figure S69 Visualization of hydrogen abstraction transition state between hydroxyl radical and tetracene molecule, calculated at the M06-2X/cc-pVDZ level of theory. 105

Table S137 Geometry (Å) of hydrogen abstraction transition state between hydroxyl radical and tetracene molecule, calculated at the M06-2X/cc-pVDZ level of theory 105

Table S138 Frequencies (cm^{-1}) of hydrogen abstraction transition state between hydroxyl radical and tetracene molecule, calculated at the M06-2X/cc-pVDZ level of theory. 106

Figure S70 Visualization of hydrogen abstraction transition state between hydroxyl radical and α-bromotetracene molecule, calculated at the M06-2X/cc-pVDZ level of theory........ 106

Table S139 Geometry (Å) of hydrogen abstraction transition state between hydroxyl radical and α-bromotetracene molecule, calculated at the M06-2X/cc-pVDZ level of theory 107

Table S140 Frequencies (cm^{-1}) of hydrogen abstraction transition state between hydroxyl radical and α-bromotetracene molecule, calculated at the M06-2X/cc-pVDZ level of theory. 107

Figure S71 Visualization of hydrogen abstraction transition state between hydroxyl radical and α-chlorotetracene acid molecule, calculated at the M06-2X/cc-pVDZ level of theory.108

Table S141 Geometry (Å) of hydrogen abstraction transition state between hydroxyl radical and α-chlorotetracene molecule, calculated at the M06-2X/cc-pVDZ level of theory 108

Table S142 Frequencies (cm^{-1}) of hydrogen abstraction transition state between hydroxyl radical and α-chlorotetracene molecule, calculated at the M06-2X/cc-pVDZ level of theory. 109

Figure S72 Visualization of hydrogen abstraction transition state between hydroxyl radical and α-fluorotetracene molecule, calculated at the M06-2X/cc-pVDZ level of theory. 109

Table S143 Geometry (Å) of hydrogen abstraction transition state between hydroxyl radical and α-fluorotetracene molecule, calculated at the M06-2X/cc-pVDZ level of theory 109

Table S144 Frequencies (cm^{-1}) of hydrogen abstraction transition state between hydroxyl radical and α-fluorotetracene molecule, calculated at the M06-2X/cc-pVDZ level of theory. 110

Figure S73 Visualization of hydrogen abstraction transition state between hydroxyl radical and α-aminotetracene molecule, calculated at the M06-2X/cc-pVDZ level of theory. 111

Table S145 Geometry (Å) of hydrogen abstraction transition state between hydroxyl radical and α-aminotetracene molecule, calculated at the M06-2X/cc-pVDZ level of theory 111

Table S146 Frequencies (cm^{-1}) of hydrogen abstraction transition state between hydroxyl radical and α-aminotetracene molecule, calculated at the M06-2X/cc-pVDZ level of theory. 112

Figure S74 Visualization of hydrogen abstraction transition state between hydroxyl radical and α-nitrotetracene molecule, calculated at the M06-2X/cc-pVDZ level of theory. 112

Table S147 Geometry (Å) of hydrogen abstraction transition state between hydroxyl radical and α-nitrotetracene molecule, calculated at the M06-2X/cc-pVDZ level of theory 112

Table S148 Frequencies (cm^{-1}) of hydrogen abstraction transition state between hydroxyl radical and α-nitrotetracene molecule, calculated at the M06-2X/cc-pVDZ level of theory. 113

Figure S75 Visualization of hydrogen abstraction transition state between hydroxyl radical and α-methyltetracene molecule, calculated at the M06-2X/cc-pVDZ level of theory. 114

Table S149 Geometry (Å) of hydrogen abstraction transition state between hydroxyl radical and α-methyltetracene molecule, calculated at the M06-2X/cc-pVDZ level of theory 114

Table S150 Frequencies (cm^{-1}) of hydrogen abstraction transition state between hydroxyl radical and α-methyltetracene molecule, calculated at the M06-2X/cc-pVDZ level of theory. 115

Figure S76 Visualization of hydrogen abstraction transition state between hydroxyl radical and α-ethyltetracene molecule, calculated at the M06-2X/cc-pVDZ level of theory. 115

Table S151 Geometry (Å) of hydrogen abstraction transition state between hydroxyl radical and α-ethyltetracene molecule, calculated at the M06-2X/cc-pVDZ level of theory 115

Table S152 Frequencies (cm^{-1}) of hydrogen abstraction transition state between hydroxyl radical and α-ethyltetracene molecule, calculated at the M06-2X/cc-pVDZ level of theory. 116

Figure S77 Visualization of hydrogen abstraction transition state between hydroxyl radical and α-propyltetracene molecule, calculated at the M06-2X/cc-pVDZ level of theory. 117

Table S153 Geometry (Å) of hydrogen abstraction transition state between hydroxyl radical and α-propyltetracene molecule, calculated at the M06-2X/cc-pVDZ level of theory 117

Table S154 Frequencies (cm^{-1}) of hydrogen abstraction transition state between hydroxyl radical and α-propyltetracene molecule, calculated at the M06-2X/cc-pVDZ level of theory. 118

Figure S78 Visualization of hydrogen abstraction transition state between hydroxyl radical and α-butyltetracene molecule, calculated at the M06-2X/cc-pVDZ level of theory. 119

Table S155 Geometry (Å) of hydrogen abstraction transition state between hydroxyl radical and α-butyltetracene molecule, calculated at the M06-2X/cc-pVDZ level of theory 119

Table S156 Frequencies (cm^{-1}) of hydrogen abstraction transition state between hydroxyl radical and α-butyltetracene molecule, calculated at the M06-2X/cc-pVDZ level of theory. 120

Figure S79 Visualization of hydrogen abstraction transition state between hydroxyl radical and α-hydroxytetracene molecule, calculated at the M06-2X/cc-pVDZ level of theory. ... 121

Table S157 Geometry (Å) of hydrogen abstraction transition state between hydroxyl radical and α-hydroxytetracene molecule, calculated at the M06-2X/cc-pVDZ level of theory 121

Table S158 Frequencies (cm^{-1}) of hydrogen abstraction transition state between hydroxyl radical and α-hydroxytetracene molecule, calculated at the M06-2X/cc-pVDZ level of theory. 122

Figure S80 Visualization of hydrogen abstraction transition state between hydroxyl radical and α-peroxytetracene molecule, calculated at the M06-2X/cc-pVDZ level of theory. 122

Table S159 Geometry (Å) of hydrogen abstraction transition state between hydroxyl radical and α-peroxytetracene molecule, calculated at the M06-2X/cc-pVDZ level of theory 122

Table S160 Frequencies (cm^{-1}) of hydrogen abstraction transition state between hydroxyl radical and α-peroxytetracene molecule, calculated at the M06-2X/cc-pVDZ level of theory. 123

Figure S81 Visualization of hydrogen abstraction transition state between hydroxyl radical and α-tetraldehyde molecule, calculated at the M06-2X/cc-pVDZ level of theory. 124

Table S161 Geometry (Å) of hydrogen abstraction transition state between hydroxyl radical and α-tetraldehyde molecule, calculated at the M06-2X/cc-pVDZ level of theory 124

Table S162 Frequencies (cm^{-1}) of hydrogen abstraction transition state between hydroxyl radical and α-tetraldehyde molecule, calculated at the M06-2X/cc-pVDZ level of theory.125

Figure S82 Visualization of hydrogen abstraction transition state between hydroxyl radical and α-tetracenic acid molecule, calculated at the M06-2X/cc-pVDZ level of theory. 125

Table S163 Geometry (Å) of hydrogen abstraction transition state between hydroxyl radical and α-tetracenic acid molecule, calculated at the M06-2X/cc-pVDZ level of theory 125

Table S164 Frequencies (cm^{-1}) of hydrogen abstraction transition state between hydroxyl radical and α-tetracenic acid molecule, calculated at the M06-2X/cc-pVDZ level of theory. 126

Figure S83 Visualization of hydrogen abstraction transition state between hydroxyl radical and β-bromotetracene molecule, calculated at the M06-2X/cc-pVDZ level of theory. 127

Table S165 Geometry (Å) of hydrogen abstraction transition state between hydroxyl radical and β-bromotetracene molecule, calculated at the M06-2X/cc-pVDZ level of theory 127

Table S166 Frequencies (cm^{-1}) of hydrogen abstraction transition state between hydroxyl radical and β-bromotetracene molecule, calculated at the M06-2X/cc-pVDZ level of theory. 128

Figure S84 Visualization of hydrogen abstraction transition state between hydroxyl radical and β-chlorotetracene acid molecule, calculated at the M06-2X/cc-pVDZ level of theory. 128

Table S167 Geometry (Å) of hydrogen abstraction transition state between hydroxyl radical and β-chlorotetracene molecule, calculated at the M06-2X/cc-pVDZ level of theory 128

Table S168 Frequencies (cm^{-1}) of hydrogen abstraction transition state between hydroxyl radical and β-chlorotetracene molecule, calculated at the M06-2X/cc-pVDZ level of theory. 129

Figure S85 Visualization of hydrogen abstraction transition state between hydroxyl radical and β-fluorotetracene molecule, calculated at the M06-2X/cc-pVDZ level of theory. 130

Table S169 Geometry (Å) of hydrogen abstraction transition state between hydroxyl radical and β-fluorotetracene molecule, calculated at the M06-2X/cc-pVDZ level of theory 130

Table S170 Frequencies (cm^{-1}) of hydrogen abstraction transition state between hydroxyl radical and β-fluorotetracene molecule, calculated at the M06-2X/cc-pVDZ level of theory. 130

Figure S86 Visualization of hydrogen abstraction transition state between hydroxyl radical and β-aminotetracene molecule, calculated at the M06-2X/cc-pVDZ level of theory. 131

Table S171 Geometry (Å) of hydrogen abstraction transition state between hydroxyl radical and β-aminotetracene molecule, calculated at the M06-2X/cc-pVDZ level of theory 131

Table S172 Frequencies (cm^{-1}) of hydrogen abstraction transition state between hydroxyl radical and β-aminotetracene molecule, calculated at the M06-2X/cc-pVDZ level of theory. 132

Figure S87 Visualization of hydrogen abstraction transition state between hydroxyl radical and β-nitrotetracene molecule, calculated at the M06-2X/cc-pVDZ level of theory. 132

Table S173 Geometry (Å) of hydrogen abstraction transition state between hydroxyl radical and β-nitrotetracene molecule, calculated at the M06-2X/cc-pVDZ level of theory 132

Table S174 Frequencies (cm^{-1}) of hydrogen abstraction transition state between hydroxyl radical and β-nitrotetracene molecule, calculated at the M06-2X/cc-pVDZ level of theory. 133

Figure S88 Visualization of hydrogen abstraction transition state between hydroxyl radical and β-methyltetracene molecule, calculated at the M06-2X/cc-pVDZ level of theory. 134

Table S175 Geometry (Å) of hydrogen abstraction transition state between hydroxyl radical and β-methyltetracene molecule, calculated at the M06-2X/cc-pVDZ level of theory 134

Table S176 Frequencies (cm^{-1}) of hydrogen abstraction transition state between hydroxyl radical and β-methyltetracene molecule, calculated at the M06-2X/cc-pVDZ level of theory. 135

Figure S89 Visualization of hydrogen abstraction transition state between hydroxyl radical and β-ethyltetracene molecule, calculated at the M06-2X/cc-pVDZ level of theory........... 135

Table S177 Geometry (Å) of hydrogen abstraction transition state between hydroxyl radical and β-ethyltetracene molecule, calculated at the M06-2X/cc-pVDZ level of theory 135

Table S178 Frequencies (cm^{-1}) of hydrogen abstraction transition state between hydroxyl radical and β-ethyltetracene molecule, calculated at the M06-2X/cc-pVDZ level of theory. 136

Figure S90 Visualization of hydrogen abstraction transition state between hydroxyl radical and β-propyltetracene molecule, calculated at the M06-2X/cc-pVDZ level of theory........ 137

Table S179 Geometry (Å) of hydrogen abstraction transition state between hydroxyl radical and β-propyltetracene molecule, calculated at the M06-2X/cc-pVDZ level of theory 137

Table S180 Frequencies (cm^{-1}) of hydrogen abstraction transition state between hydroxyl radical and β-propyltetracene molecule, calculated at the M06-2X/cc-pVDZ level of theory. 138

Figure S91 Visualization of hydrogen abstraction transition state between hydroxyl radical and β-butyltetracene molecule, calculated at the M06-2X/cc-pVDZ level of theory. 139
Table S181 Geometry (Å) of hydrogen abstraction transition state between hydroxyl radical and β-butyltetracene molecule, calculated at the M06-2X/cc-pVDZ level of theory 139
Table S182 Frequencies (cm^{-1}) of hydrogen abstraction transition state between hydroxyl radical and β-butyltetracene molecule, calculated at the M06-2X/cc-pVDZ level of theory. 140
Figure S92 Visualization of hydrogen abstraction transition state between hydroxyl radical and β-hydroxytetracene molecule, calculated at the M06-2X/cc-pVDZ level of theory..... 140
Table S183 Geometry (Å) of hydrogen abstraction transition state between hydroxyl radical and β-hydroxytetracene molecule, calculated at the M06-2X/cc-pVDZ level of theory 140
Table S184 Frequencies (cm^{-1}) of hydrogen abstraction transition state between hydroxyl radical and β-hydroxytetracene molecule, calculated at the M06-2X/cc-pVDZ level of theory. 141
Figure S93 Visualization of hydrogen abstraction transition state between hydroxyl radical and β -peroxytetracene molecule, calculated at the M06-2X/cc-pVDZ level of theory. 142
Table S185 Geometry (Å) of hydrogen abstraction transition state between hydroxyl radical and β-peroxytetracene molecule, calculated at the M06-2X/cc-pVDZ level of theory 142
Table S186 Frequencies (cm^{-1}) of hydrogen abstraction transition state between hydroxyl radical and β-peroxytetracene molecule, calculated at the M06-2X/cc-pVDZ level of theory. 143
Figure S94 Visualization of hydrogen abstraction transition state between hydroxyl radical and β-tetraldehyde molecule, calculated at the M06-2X/cc-pVDZ level of theory. 143
Table S187 Geometry (Å) of hydrogen abstraction transition state between hydroxyl radical and β-tetraldehyde molecule, calculated at the M06-2X/cc-pVDZ level of theory 143
Table S188 Frequencies (cm^{-1}) of hydrogen abstraction transition state between hydroxyl radical and β-tetraldehyde molecule, calculated at the M06-2X/cc-pVDZ level of theory. 144
Figure S95 Visualization of hydrogen abstraction transition state between hydroxyl radical and β -tetracenic acid molecule, calculated at the M06-2X/cc-pVDZ level of theory. 145
Table S189 Geometry (Å) of hydrogen abstraction transition state between hydroxyl radical and β-tetracenic acid molecule, calculated at the M06-2X/cc-pVDZ level of theory 145

Table S190 Frequencies (cm^{-1}) of hydrogen abstraction transition state between hydroxyl radical and β-tetracenic acid molecule, calculated at the M06-2X/cc-pVDZ level of theory. 146

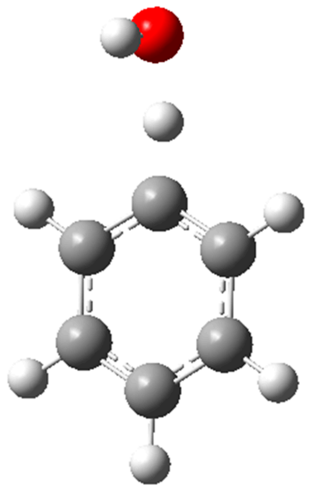

Figure S1 Visualization of hydrogen abstraction transition state between hydroxyl radical and benzene molecule, calculated at the M06-2X/cc-pVTZ level of theory.

Table S1 Geometry (Å) of hydrogen abstraction transition state between hydroxyl radical and benzene molecule, calculated at the M06-2X/cc-pVDZ level of theory

Atom	x	y	z
**C**	-0.014	-0.061	-0.058
**C**	-0.022	1.327	-0.076
**C**	1.139	2.086	-0.025
**C**	2.365	1.419	0.029
**C**	2.403	0.024	0.038
**C**	1.220	-0.715	-0.005
**H**	-1.080	1.896	-0.151
**H**	1.093	3.174	-0.033
**H**	3.292	1.991	0.063
**H**	-0.945	-0.624	-0.093
**H**	3.362	-0.491	0.082
**H**	1.255	-1.804	0.003
**O**	-2.210	2.498	0.108
**H**	-2.177	2.465	1.079

Table S2 Frequencies (cm^{-1}) of hydrogen abstraction transition state between hydroxyl radical and benzene molecule, calculated at the M06-2X/cc-pVDZ level of theory.

-1133	73	100	208	365	409	484	606
629	703	750	826	853	928	995	999
1017	1046	1047	1077	1115	1170	1178	1279
1320	1334	1475	1506	1644	1663	3205	3211
3221	3226	3231	3793				

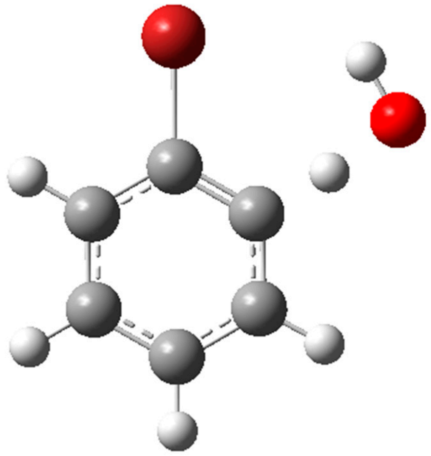

Figure S2 Visualization of hydrogen abstraction transition state between hydroxyl radical and bromobenzene molecule, calculated at the M06-2X/cc-pVDZ level of theory.

Table S3 Geometry (Å) of hydrogen abstraction transition state between hydroxyl radical and bromobenzene molecule, calculated at the M06-2X/cc-pVDZ level of theory

Atom	x	y	z
C	-0.075	0.188	0.103
C	0.009	1.568	-0.005
C	1.272	2.161	-0.080
C	2.418	1.368	-0.045
C	2.323	-0.019	0.063
C	1.059	-0.605	0.138
H	-1.190	-0.314	0.167
H	-0.896	2.172	-0.031
H	1.357	3.243	-0.165
H	3.403	1.829	-0.104
H	3.214	-0.643	0.091
Br	0.906	-2.492	0.286
O	-2.256	-0.986	0.242
H	-1.860	-1.874	0.301

Table S4 Frequencies (cm^{-1}) of hydrogen abstraction transition state between hydroxyl radical and bromobenzene molecule, calculated at the M06-2X/cc-pVDZ level of theory.

-1466	82	110	168	215	261	312	414
455	479	646	679	697	759	816	837
908	984	1011	1020	1059	1129	1140	1168
1254	1320	1372	1465	1498	1635	1655	3212
3221	3230	3236	3769				

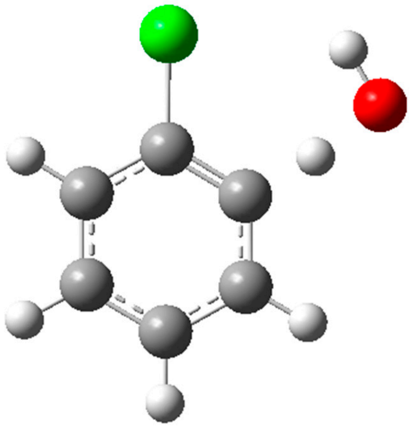

Figure S3 Visualization of hydrogen abstraction transition state between hydroxyl radical and chlorobenzene molecule, calculated at the M06-2X/cc-pVDZ level of theory.

Table S5 Geometry (Å) of hydrogen abstraction transition state between hydroxyl radical and chlorobenzene molecule, calculated at the M06-2X/cc-pVDZ level of theory

Atom	x	y	z
C	-0.086	0.202	0.103
C	0.010	1.581	-0.006
C	1.277	2.164	-0.082
C	2.418	1.361	-0.047
C	2.311	-0.025	0.063
C	1.043	-0.599	0.138
H	-1.200	-0.306	0.170
H	-0.890	2.193	-0.032
H	1.371	3.245	-0.168
H	3.405	1.816	-0.106
H	3.195	-0.659	0.091
Cl	0.885	-2.334	0.277
O	-2.240	-1.010	0.250
H	-1.817	-1.885	0.308

Table S6 Frequencies (cm^{-1}) of hydrogen abstraction transition state between hydroxyl radical and chlorobenzene molecule, calculated at the M06-2X/cc-pVDZ level of theory.

-1483	81	113	175	208	300	386	414
474	489	650	678	716	760	819	831
901	978	1016	1020	1058	1138	1144	1167
1250	1323	1377	1466	1503	1637	1660	3214
3223	3231	3238	3776				

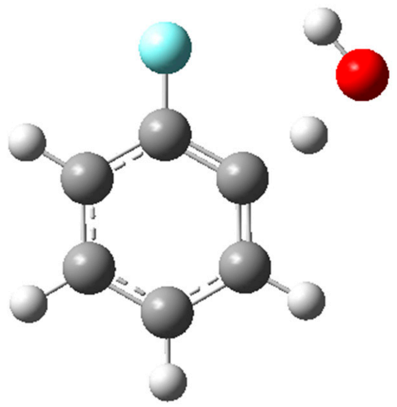

Figure S4 Visualization of hydrogen abstraction transition state between hydroxyl radical and fluorobenzene molecule, calculated at the M06-2X/cc-pVDZ level of theory.

Table S7 Geometry (Å) of hydrogen abstraction transition state between hydroxyl radical and fluorobenzene molecule, calculated at the M06-2X/cc-pVDZ level of theory

Atom	x	y	z
C	-0.125	0.250	0.097
C	0.003	1.623	-0.032
C	1.287	2.174	-0.099
C	2.409	1.347	-0.037
C	2.272	-0.035	0.092
C	0.990	-0.564	0.157
H	-1.233	-0.289	0.161
H	-0.880	2.259	-0.079
H	1.407	3.251	-0.200
H	3.406	1.781	-0.090
H	3.132	-0.701	0.142
F	0.823	-1.898	0.282
O	-2.168	-1.114	0.249
H	-1.641	-1.930	0.317

Table S8 Frequencies (cm^{-1}) of hydrogen abstraction transition state between hydroxyl radical and fluorobenzene molecule, calculated at the M06-2X/cc-pVDZ level of theory.

-1512	86	89	203	249	383	418	426
517	540	648	673	768	802	821	855
899	967	1006	1043	1050	1131	1161	1254
1274	1335	1404	1478	1536	1653	1680	3216
3223	3231	3239	3774				

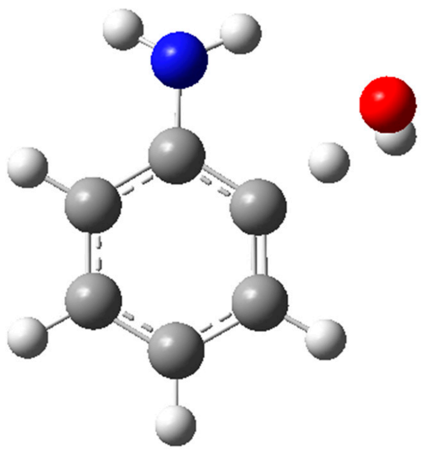

Figure S5 Visualization of hydrogen abstraction transition state between hydroxyl radical and aminobenzene molecule, calculated at the M06-2X/cc-pVDZ level of theory.

Table S9 Geometry (Å) of hydrogen abstraction transition state between hydroxyl radical and aminobenzene molecule, calculated at the M06-2X/cc-pVDZ level of theory

Atom	x	y	z
C	0.012	0.098	-0.079
C	0.000	1.481	-0.078
C	-1.230	2.144	-0.023
C	-2.402	1.391	0.055
C	-2.370	0.000	0.075
C	-1.143	-0.685	0.019
H	0.932	2.041	-0.136
H	-1.268	3.231	-0.043
H	-3.366	1.897	0.104
H	-3.296	-0.572	0.139
H	1.052	-0.519	-0.153
N	-1.067	-2.067	-0.006
H	-1.847	-2.566	0.396
H	-0.158	-2.451	0.219
O	1.945	-1.378	0.262
H	2.095	-1.017	1.152

Table S10 Frequencies (cm^{-1}) of hydrogen abstraction transition state between hydroxyl radical and aminobenzene molecule, calculated at the M06-2X/cc-pVDZ level of theory.

-1168	105	121	207	250	331	371	394
441	513	544	560	636	720	758	786
841	858	954	988	998	1055	1079	1146
1160	1196	1300	1316	1345	1377	1489	1538
1626	1637	1678	3198	3212	3217	3236	3576
3704	3793						

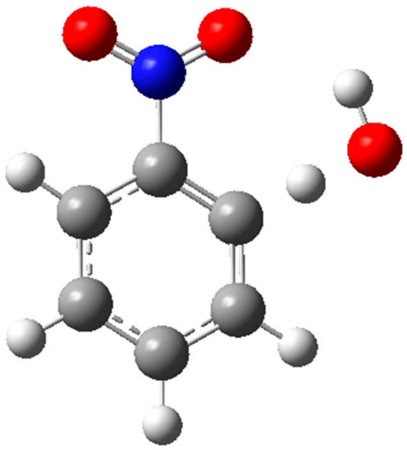

Figure S6 Visualization of hydrogen abstraction transition state between hydroxyl radical and nitrobenzene molecule, calculated at the M06-2X/cc-pVDZ level of theory.

Table S11 Geometry (Å) of hydrogen abstraction transition state between hydroxyl radical and nitrobenzene molecule, calculated at the M06-2X/cc-pVDZ level of theory

Atom	x	y	z
C	0.028	0.116	0.088
C	-0.021	1.499	0.031
C	-1.267	2.128	-0.043
C	-2.441	1.373	-0.060
C	-2.385	-0.017	-0.003
C	-1.137	-0.633	0.071
H	1.164	-0.382	0.153
H	0.900	2.080	0.045
H	-1.319	3.215	-0.088
H	-3.408	1.868	-0.119
H	-3.282	-0.632	-0.015
N	-1.085	-2.106	0.131
O	0.008	-2.638	0.196
O	-2.139	-2.705	0.113
O	2.285	-0.872	0.217
H	1.992	-1.800	0.242

Table S12 Frequencies (cm^{-1}) of hydrogen abstraction transition state between hydroxyl radical and aminobenzene molecule, calculated at the M06-2X/cc-pVDZ level of theory.

-1620	47	84	156	172	261	326	368
424	442	458	541	649	674	713	734
774	820	829	891	922	996	1029	1042
1058	1137	1161	1184	1258	1351	1373	1474
1479	1499	1645	1663	1717	3216	3228	3237
3254	3775						

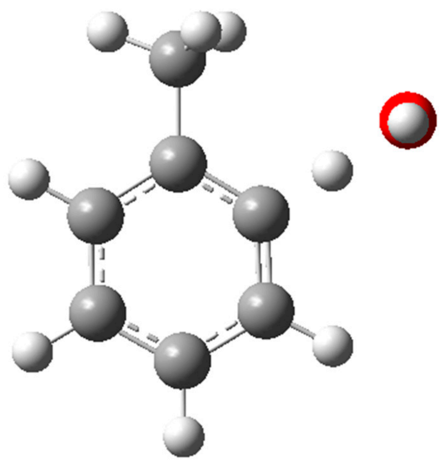

Figure S7 Visualization of hydrogen abstraction transition state between hydroxyl radical and methylbenzene molecule, calculated at the M06-2X/cc-pVDZ level of theory.

Table S13 Geometry (Å) of hydrogen abstraction transition state between hydroxyl radical and methylbenzene molecule, calculated at the M06-2X/cc-pVDZ level of theory

Atom	x	y	z
C	0.033	0.071	-0.166
C	0.024	1.457	-0.169
C	1.235	2.131	0.018
C	2.406	1.399	0.208
C	2.378	0.004	0.215
C	1.178	-0.697	0.036
H	-0.998	-0.509	-0.364
H	-0.903	2.005	-0.325
H	1.259	3.219	0.011
H	3.352	1.919	0.352
H	3.301	-0.557	0.364
C	1.119	-2.201	0.063
H	2.122	-2.633	-0.022
H	0.494	-2.580	-0.753
H	0.674	-2.552	1.004
O	-2.127	-1.183	-0.238
H	-2.224	-1.120	0.728

Table S14 Frequencies (cm^{-1}) of hydrogen abstraction transition state between hydroxyl radical and methylbenzene molecule, calculated at the M06-2X/cc-pVDZ level of theory.

-1040	42	78	95	133	207	334	381
451	511	543	639	713	761	791	850
878	962	1003	1009	1015	1048	1058	1090
1151	1170	1230	1282	1297	1331	1398	1454
1460	1486	1518	1641	1672	3067	3137	3163
3196	3209	3218	3230	3789			

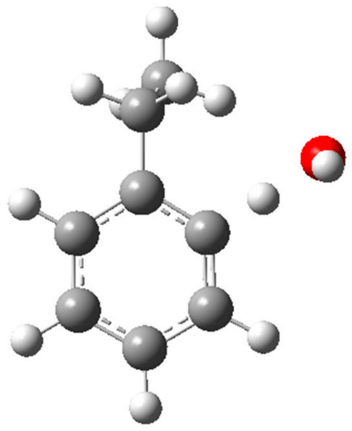

Figure S8 Visualization of hydrogen abstraction transition state between hydroxyl radical and ethylbenzene molecule, calculated at the M06-2X/cc-pVDZ level of theory.

Table S15 Geometry (Å) of hydrogen abstraction transition state between hydroxyl radical and ethylbenzene molecule, calculated at the M06-2X/cc-pVDZ level of theory

Atom	x	y	z
C	-0.056	0.139	0.393
C	-0.061	1.481	0.047
C	1.129	2.053	-0.415
C	2.279	1.271	-0.503
C	2.252	-0.075	-0.134
C	1.075	-0.673	0.334
H	-0.972	2.072	0.125
H	1.153	3.103	-0.701
H	3.209	1.713	-0.860
H	3.159	-0.677	-0.204
H	-1.078	-0.366	0.760
C	1.008	-2.132	0.709
H	2.028	-2.530	0.780
H	0.545	-2.230	1.700
C	0.196	-2.944	-0.305
H	0.628	-2.849	-1.309
H	0.184	-4.005	-0.029
H	-0.839	-2.585	-0.336
O	-2.064	-0.883	1.480
H	-1.845	-0.474	2.335

Table S16 Frequencies (cm^{-1}) of hydrogen abstraction transition state between hydroxyl radical and ethylbenzene molecule, calculated at the M06-2X/cc-pVDZ level of theory.

-1019	70	83	92	130	143	225	301
360	382	461	537	568	638	733	766
782	787	843	882	966	989	1008	1010
1056	1068	1091	1110	1151	1169	1221	1254
1284	1303	1334	1353	1386	1463	1472	1475

1484	1515	1639	1672	3065	3076	3120	3146
3154	3196	3209	3220	3230	3788		

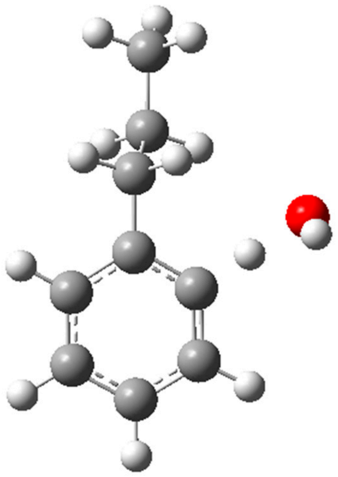

Figure S9 Visualization of hydrogen abstraction transition state between hydroxyl radical and propylbenzene molecule, calculated at the M06-2X/cc-pVDZ level of theory.

Table S17 Geometry (Å) of hydrogen abstraction transition state between hydroxyl radical and propylbenzene molecule, calculated at the M06-2X/cc-pVDZ level of theory

Atom	x	y	z
C	1.096	2.132	-0.011
C	2.304	1.450	-0.132
C	2.418	0.069	-0.101
C	1.251	-0.690	0.034
C	0.020	-0.046	0.143
C	-0.056	1.348	0.120
H	3.292	2.110	-0.283
H	3.391	-0.412	-0.188
H	1.308	-1.777	0.055
H	-0.891	-0.633	0.251
H	-1.025	1.841	0.209
C	1.043	3.637	-0.075
H	1.793	4.059	0.609
H	0.055	3.981	0.262
C	1.318	4.165	-1.487
H	0.578	3.738	-2.178
H	2.304	3.805	-1.809
C	1.278	5.688	-1.542
H	2.039	6.117	-0.877
H	1.470	6.054	-2.557
H	0.297	6.067	-1.225
O	4.338	2.908	-0.114
H	4.464	2.776	0.842

Table S18 Frequencies (cm^{-1}) of hydrogen abstraction transition state between hydroxyl radical and propylbenzene molecule, calculated at the M06-2X/cc-pVDZ level of theory.

-1006	50	72	88	99	114	132	243
271	314	350	382	466	547	593	639
731	734	768	809	850	870	885	915
966	1007	1009	1059	1073	1080	1115	1118
1152	1169	1220	1238	1279	1284	1308	1317
1340	1374	1394	1460	1467	1474	1477	1483
1515	1639	1671	3058	3060	3071	3101	3118
3136	3143	3195	3209	3219	3230	3787	

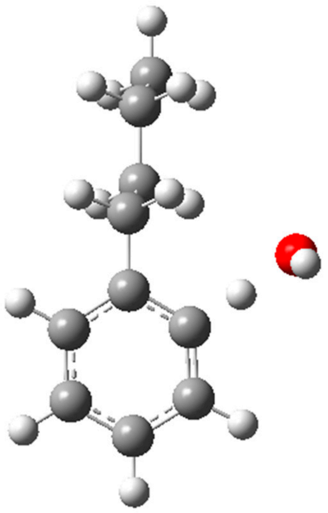

Figure S10 Visualization of hydrogen abstraction transition state between hydroxyl radical and butylbenzene molecule, calculated at the M06-2X/cc-pVDZ level of theory.

Table S19 Geometry (Å) of hydrogen abstraction transition state between hydroxyl radical and butylbenzene molecule, calculated at the M06-2X/cc-pVDZ level of theory

Atom	x	y	z
C	1.076	2.125	-0.003
C	2.288	1.458	-0.167
C	2.420	0.079	-0.134
C	1.269	-0.694	0.050
C	0.035	-0.065	0.204
C	-0.060	1.327	0.176
H	3.259	2.131	-0.360
H	3.395	-0.390	-0.256
H	1.342	-1.780	0.074
H	-0.863	-0.664	0.350
H	-1.031	1.809	0.300
C	1.002	3.629	-0.071
H	1.771	4.062	0.584
H	0.024	3.963	0.299
C	1.223	4.153	-1.494

**H**	0.460	3.722	-2.160
**H**	2.199	3.801	-1.854
**C**	1.174	5.676	-1.565
**H**	1.944	6.087	-0.897
**H**	0.204	6.026	-1.182
**C**	1.388	6.200	-2.982
**H**	0.616	5.813	-3.661
**H**	1.351	7.295	-3.015
**H**	2.365	5.881	-3.369
**O**	4.300	2.945	-0.236
**H**	4.473	2.810	0.712

Table S20 Frequencies (cm^{-1}) of hydrogen abstraction transition state between hydroxyl radical and butylbenzene molecule, calculated at the M06-2X/cc-pVDZ level of theory.

-1001	37	61	76	96	97	126	132
214	241	287	333	383	402	476	554
588	639	719	735	768	784	809	855
881	916	926	967	1006	1008	1044	1061
1085	1090	1120	1129	1153	1170	1217	1225
1258	1280	1286	1310	1317	1337	1351	1389
1395	1458	1465	1471	1476	1478	1485	1515
1639	1672	3046	3053	3059	3066	3082	3098
3119	3133	3141	3192	3208	3217	3230	3783

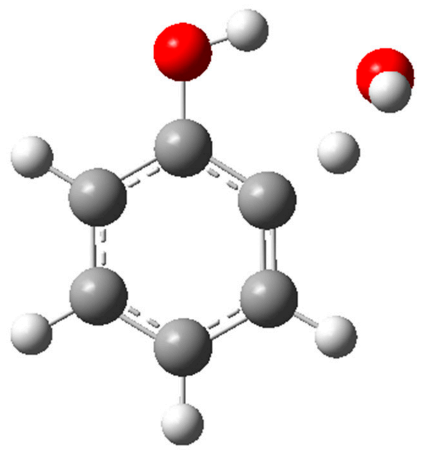

Figure S11 Visualization of hydrogen abstraction transition state between hydroxyl radical and hydroxybenzene molecule, calculated at the M06-2X/cc-pVDZ level of theory.

Table S21 Geometry (Å) of hydrogen abstraction transition state between hydroxyl radical and hydroxybenzene molecule, calculated at the M06-2X/cc-pVDZ level of theory

Atom	x	y	z
**C**	-0.001	0.129	-0.013
**C**	0.018	1.512	-0.085
**C**	1.256	2.160	-0.074

C	2.426	1.404	0.028
C	2.388	0.016	0.121
C	1.153	-0.643	0.112
H	-1.028	-0.514	-0.071
H	-0.909	2.078	-0.168
H	1.302	3.245	-0.148
H	3.392	1.909	0.040
H	3.296	-0.577	0.209
O	1.117	-1.991	0.216
H	0.187	-2.269	0.222
O	-1.760	-1.543	0.276
H	-1.940	-1.293	1.199

Table S22 Frequencies (cm^{-1}) of hydrogen abstraction transition state between hydroxyl radical and hydroxybenzene molecule, calculated at the M06-2X/cc-pVDZ level of theory.

-1186	105	141	221	299	353	425	440
530	547	627	638	699	757	768	841
868	960	993	1005	1053	1124	1158	1168
1226	1301	1317	1365	1438	1501	1557	1652
1672	3211	3218	3229	3236	3738	3785	

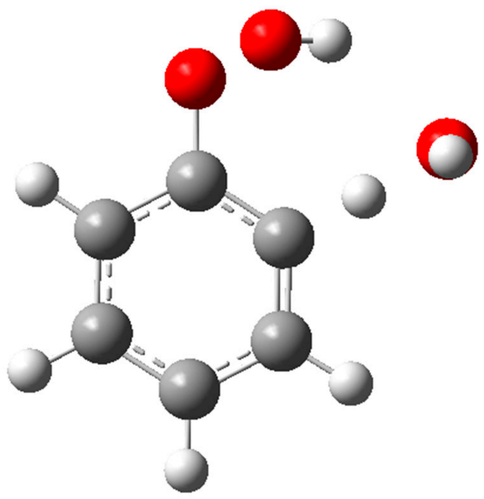

Figure S12 Visualization of hydrogen abstraction transition state between hydroxyl radical and peroxybenzene molecule, calculated at the M06-2X/cc-pVDZ level of theory.

Table S23 Geometry (Å) of hydrogen abstraction transition state between hydroxyl radical and peroxybenzene molecule, calculated at the M06-2X/cc-pVDZ level of theory

Atom	x	y	z
C	0.156	-0.049	0.117
C	0.040	1.329	0.031
C	1.201	2.101	-0.065
C	2.448	1.475	-0.061
C	2.552	0.091	0.055

**C**	1.394	-0.682	0.151
**H**	-0.943	1.797	0.017
**H**	1.128	3.184	-0.146
**H**	3.355	2.073	-0.133
**H**	-0.852	-0.714	0.107
**H**	3.517	-0.412	0.080
**O**	1.559	-2.044	0.187
**O**	0.599	-2.644	1.042
**H**	-0.181	-2.735	0.468
**O**	-1.898	-1.462	0.318
**H**	-1.966	-1.337	1.282

Table S24 Frequencies (cm^{-1}) of hydrogen abstraction transition state between hydroxyl radical and peroxybenzene molecule, calculated at the M06-2X/cc-pVDZ level of theory.

-1148	21	94	146	199	255	301	369
415	472	499	533	608	630	711	759
784	848	880	966	1006	1012	1030	1054
1103	1141	1161	1178	1275	1280	1338	1474
1475	1508	1645	1664	3214	3220	3229	3238
3732	3772						

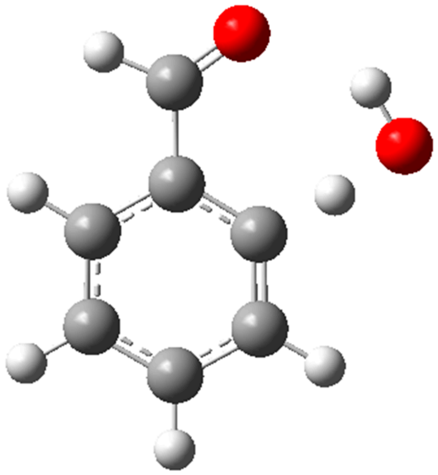

Figure S13 Visualization of hydrogen abstraction transition state between hydroxyl radical and benzaldehyde molecule, calculated at the M06-2X/cc-pVDZ level of theory.

Table S25 Geometry (Å) of hydrogen abstraction transition state between hydroxyl radical and benzaldehyde molecule, calculated at the M06-2X/cc-pVDZ level of theory

Atom	x	y	z
**C**	0.062	0.159	0.085
**C**	-0.013	1.539	0.022
**C**	-1.276	2.139	-0.054
**C**	-2.432	1.359	-0.065
**C**	-2.332	-0.029	0.000

**C**	-1.077	-0.646	0.076
**H**	1.194	-0.345	0.152
**H**	0.891	2.145	0.031
**H**	-1.353	3.225	-0.104
**H**	-3.410	1.833	-0.124
**H**	-3.231	-0.648	-0.008
**C**	-1.015	-2.126	0.144
**H**	-2.005	-2.631	0.127
**O**	0.000	-2.781	0.212
**O**	2.241	-0.998	0.221
**H**	1.812	-1.876	0.243

Table S26 Frequencies (cm^{-1}) of hydrogen abstraction transition state between hydroxyl radical and benzaldehyde molecule, calculated at the M06-2X/cc-pVDZ level of theory.

-1562	87	123	158	240	253	380	406
446	486	500	640	676	698	766	802
853	873	922	988	1025	1047	1053	1060
1142	1167	1236	1262	1344	1397	1417	1476
1516	1642	1665	1823	2985	3198	3211	3227
3234	3706						

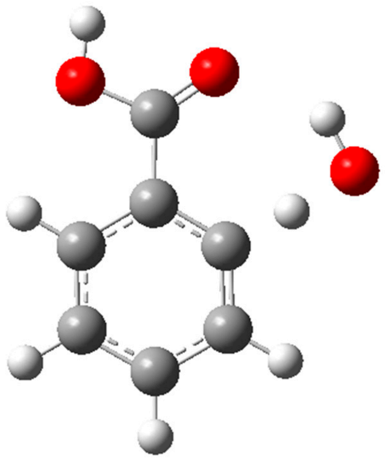

Figure S14 Visualization of hydrogen abstraction transition state between hydroxyl radical and benzoic acid molecule, calculated at the M06-2X/cc-pVDZ level of theory.

Table S27 Geometry (Å) of hydrogen abstraction transition state between hydroxyl radical and benzoic acid molecule, calculated at the M06-2X/cc-pVDZ level of theory

Atom	x	y	z
**C**	-0.046	-0.018	-0.067
**C**	0.020	1.372	-0.073
**C**	-1.148	2.132	0.001
**C**	-2.394	1.504	0.081
**C**	-2.440	0.121	0.085

C	-1.290	-0.661	0.013
H	0.988	1.866	-0.135
H	-1.091	3.220	-0.003
H	-3.312	2.087	0.139
H	-3.565	-0.389	0.158
C	-1.391	-2.146	0.020
H	0.861	-0.616	-0.125
O	-0.192	-2.754	-0.055
H	-0.363	-3.707	-0.044
O	-2.421	-2.779	0.085
O	-4.641	-0.999	0.225
H	-4.243	-1.890	0.200

Table S28 Frequencies (cm^{-1}) of hydrogen abstraction transition state between hydroxyl radical and benzaldehyde molecule, calculated at the M06-2X/cc-pVDZ level of theory.

-1573	62	94	159	165	233	365	402
434	437	469	512	617	638	673	696
739	786	825	829	850	934	997	1031
1042	1062	1136	1159	1172	1232	1267	1341
1389	1403	1471	1520	1640	1671	1834	3210
3226	3232	3244	3725	3818			

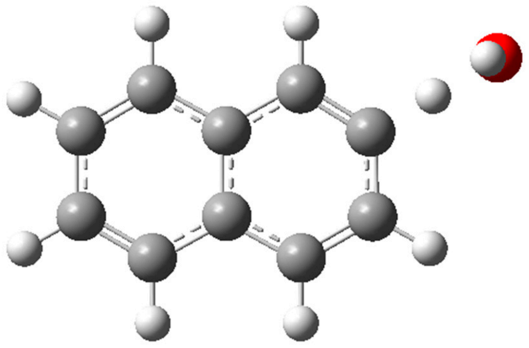

Figure S15 Visualization of hydrogen abstraction transition state between hydroxyl radical and naphtalene molecule, calculated at the M06-2X/cc-pVDZ level of theory.

Table S29 Geometry (Å) of hydrogen abstraction transition state between hydroxyl radical and naphtalene molecule, calculated at the M06-2X/cc-pVDZ level of theory

Atom	x	y	z
C	0.041	-0.062	-0.075
C	1.215	-0.760	-0.064
C	2.446	-0.042	-0.032
C	2.414	1.382	0.001
C	1.160	2.054	-0.001
C	-0.019	1.347	-0.034
H	3.719	-1.797	-0.055

**H**	1.226	-1.850	-0.087
**C**	3.702	-0.708	-0.029
**C**	3.644	2.095	0.036
**H**	1.147	3.143	0.024
**C**	4.843	1.427	0.038
**C**	4.872	0.009	0.005
**H**	3.617	3.185	0.061
**H**	-0.994	-0.677	-0.132
**H**	-0.981	1.857	-0.038
**H**	5.830	-0.509	0.006
**H**	5.779	1.983	0.064
**O**	-2.078	-1.344	0.154
**H**	-1.995	-1.358	1.122

Table S30 Frequencies (cm^{-1}) of hydrogen abstraction transition state between hydroxyl radical and naphtalene molecule, calculated at the M06-2X/cc-pVDZ level of theory.

-1160	61	70	135	186	200	369	380
412	486	501	514	573	624	650	751
776	785	797	838	858	887	934	940
988	1006	1022	1048	1058	1101	1146	1160
1165	1214	1248	1279	1284	1376	1405	1436
1472	1493	1556	1654	1668	1705	3200	3203
3204	3210	3218	3229	3230	3792		

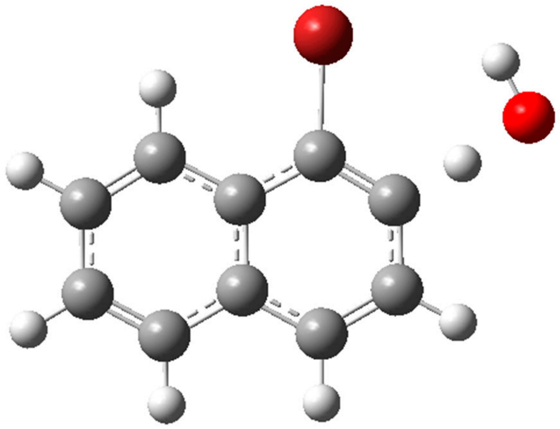

Figure S16 Visualization of hydrogen abstraction transition state between hydroxyl radical and α-bromonaphtalene molecule, calculated at the M06-2X/cc-pVDZ level of theory.

Table S31 Geometry (Å) of hydrogen abstraction transition state between hydroxyl radical and α-bromonaphtalene molecule, calculated at the M06-2X/cc-pVDZ level of theory

Atom	x	y	z
**C**	-2.395	-0.014	-0.001
**C**	-1.202	-0.695	0.000
**C**	0.031	0.011	0.000

C	0.012	1.439	0.000
C	-1.242	2.108	0.000
C	-2.419	1.402	-0.001
H	-1.192	-1.782	-0.001
C	1.311	-0.625	0.000
C	1.230	2.172	0.000
H	-1.249	3.197	0.000
C	2.441	1.526	0.000
C	2.455	0.117	0.000
H	1.186	3.261	0.000
H	-3.332	-0.569	-0.001
H	-3.373	1.926	-0.001
H	3.379	2.078	0.000
H	3.550	-0.422	0.000
Br	1.440	-2.524	0.000
O	4.617	-1.104	0.001
H	4.213	-1.990	0.001

Table S32 Frequencies (cm^{-1}) of hydrogen abstraction transition state between hydroxyl radical and α-bromonaphtalene molecule, calculated at the M06-2X/cc-pVDZ level of theory.

-1450	47	104	111	147	180	203	235
301	394	423	438	476	532	551	598
626	668	744	766	784	818	830	873
882	904	959	991	1011	1025	1061	1149
1151	1162	1183	1231	1273	1315	1375	1410
1430	1463	1496	1554	1645	1665	1702	3202
3209	3214	3227	3230	3242	3770		

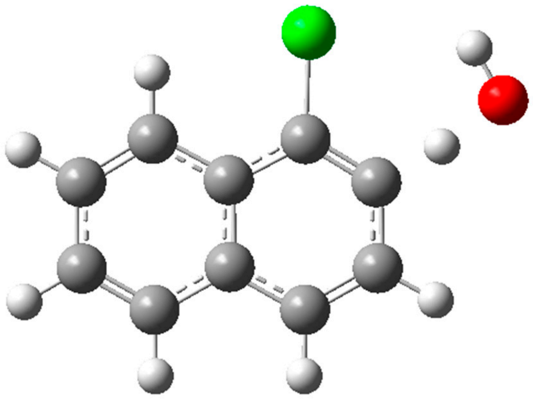

Figure S17 Visualization of hydrogen abstraction transition state between hydroxyl radical and α-chloronaphtalene molecule, calculated at the M06-2X/cc-pVDZ level of theory.

Table S33 Geometry (Å) of hydrogen abstraction transition state between hydroxyl radical and α-chloronaphtalene molecule, calculated at the M06-2X/cc-pVDZ level of theory

Atom	x	y	z

**C**	-2.390	-0.024	-0.001
**C**	-1.195	-0.702	0.000
**C**	0.034	0.010	0.000
**C**	0.014	1.437	-0.001
**C**	-1.242	2.103	-0.001
**C**	-2.417	1.392	-0.001
**H**	-1.181	-1.789	0.000
**C**	1.312	-0.627	0.000
**C**	1.232	2.170	0.000
**H**	-1.253	3.192	-0.001
**C**	2.443	1.523	0.000
**C**	2.457	0.114	0.000
**H**	1.190	3.259	-0.001
**H**	-3.326	-0.580	-0.001
**H**	-3.372	1.914	-0.002
**H**	3.381	2.075	0.000
**H**	3.547	-0.443	0.001
**Cl**	1.414	-2.373	0.001
**O**	4.574	-1.176	0.001
**H**	4.128	-2.042	0.002

Table S34 Frequencies (cm^{-1}) of hydrogen abstraction transition state between hydroxyl radical and α-chloronaphtalene molecule, calculated at the M06-2X/cc-pVDZ level of theory.

-1466	57	110	115	189	222	226	249
367	411	425	446	477	543	558	593
628	678	742	766	784	817	842	877
878	902	976	990	1006	1024	1058	1150
1152	1164	1182	1230	1275	1320	1377	1413
1430	1463	1498	1553	1646	1667	1703	3204
3209	3216	3229	3232	3244	3777		

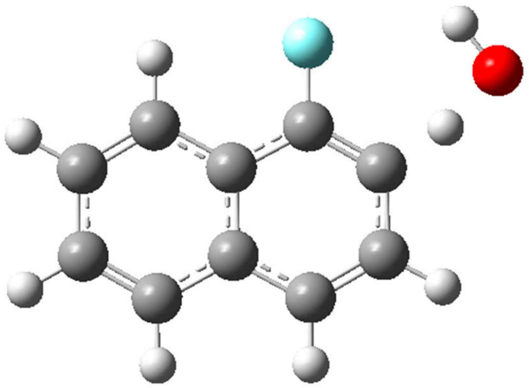

Figure S18 Visualization of hydrogen abstraction transition state between hydroxyl radical and α-fluoronaphtalene molecule, calculated at the M06-2X/cc-pVDZ level of theory.

Table S35 Geometry (Å) of hydrogen abstraction transition state between hydroxyl radical and α-fluoronaphtalene molecule, calculated at the M06-2X/cc-pVDZ level of theory

Atom	x	y	z
C	-2.366	-0.069	-0.002
C	-1.162	-0.730	-0.001
C	0.049	0.010	0.000
C	0.021	1.434	-0.001
C	-1.245	2.081	-0.002
C	-2.408	1.349	-0.002
H	-1.119	-1.817	-0.001
C	1.322	-0.613	0.000
C	1.241	2.167	0.000
H	-1.277	3.170	-0.002
C	2.454	1.519	0.000
C	2.474	0.110	0.001
H	1.201	3.256	-0.001
H	-3.296	-0.634	-0.002
H	-3.371	1.857	-0.003
H	3.388	2.078	0.001
H	3.542	-0.507	0.002
F	1.372	-1.963	0.001
O	4.423	-1.393	0.002
H	3.846	-2.177	0.002

Table S36 Frequencies (cm^{-1}) of hydrogen abstraction transition state between hydroxyl radical and α-fluoronaphtalene molecule, calculated at the M06-2X/cc-pVDZ level of theory.

-1504	64	74	146	182	209	267	270
371	435	477	477	492	570	589	589
636	723	747	768	787	837	876	881
898	914	990	1004	1025	1053	1076	1154
1160	1171	1227	1234	1279	1366	1396	1433
1443	1469	1516	1564	1653	1684	1716	3204
3208	3214	3226	3231	3236	3781		

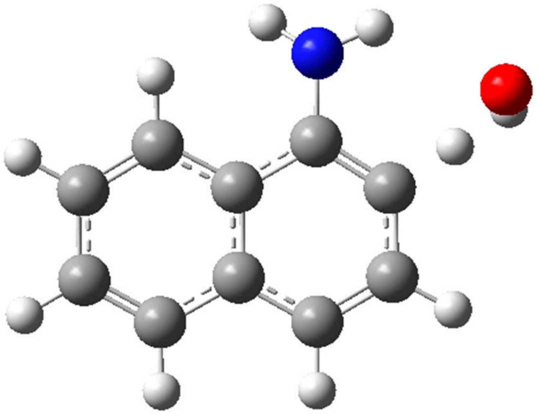

Figure S19 Visualization of hydrogen abstraction transition state between hydroxyl radical and α-aminonaphtalene molecule, calculated at the M06-2X/cc-pVDZ level of theory.

Table S37 Geometry (Å) of hydrogen abstraction transition state between hydroxyl radical and α-aminonaphtalene molecule, calculated at the M06-2X/cc-pVDZ level of theory

Atom	x	y	z
C	-2.077	-0.380	0.037
C	-0.961	0.420	-0.042
C	-1.078	1.834	-0.048
C	-2.375	2.423	0.009
C	-3.507	1.568	0.095
C	-3.365	0.202	0.114
H	0.019	-0.046	-0.132
C	0.075	2.692	-0.104
C	-2.525	3.839	-0.023
H	-4.497	2.021	0.142
C	-1.416	4.646	-0.113
C	-0.145	4.051	-0.179
H	-3.525	4.268	0.010
H	-1.972	-1.464	0.031
H	-4.244	-0.438	0.178
H	0.854	4.735	-0.270
H	-1.515	5.729	-0.152
N	1.357	2.167	-0.148
H	2.083	2.857	0.013
H	1.503	1.306	0.358
O	2.010	5.170	0.140
H	1.770	5.426	1.047

Table S38 Frequencies (cm-1) of hydrogen abstraction transition state between hydroxyl radical and α-aminonaphtalene molecule, calculated at the M06-2X/cc-pVDZ level of theory.

-1194	75	112	130	189	229	264	292
352	365	437	462	479	496	552	582
583	620	686	733	746	777	800	802
821	886	900	974	987	1019	1036	1061
1125	1152	1163	1180	1204	1236	1262	1298
1358	1384	1436	1446	1473	1510	1562	1631
1649	1672	1700	3202	3207	3210	3221	3225
3232	3566	3689	3795				

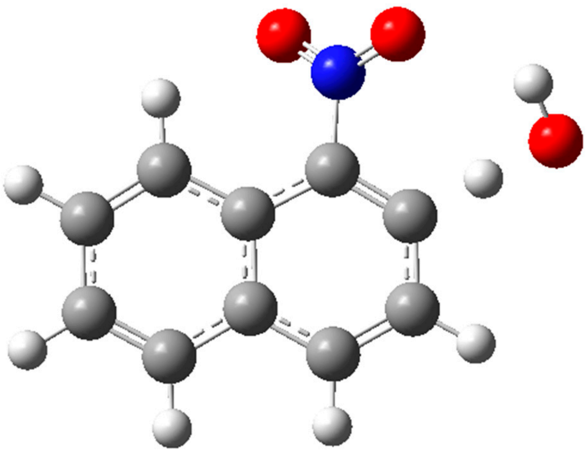

Figure S20 Visualization of hydrogen abstraction transition state between hydroxyl radical and α-nitronaphtalene molecule, calculated at the M06-2X/cc-pVDZ level of theory.

Table S39 Geometry (Å) of hydrogen abstraction transition state between hydroxyl radical and α-nitronaphtalene molecule, calculated at the M06-2X/cc-pVDZ level of theory

Atom	x	y	z
C	-2.397	-0.027	-0.114
C	-1.217	-0.727	-0.041
C	0.023	-0.032	0.031
C	0.013	1.397	0.012
C	-1.231	2.082	-0.053
C	-2.413	1.388	-0.113
H	-1.232	-1.811	-0.032
C	1.311	-0.653	0.073
C	1.231	2.128	0.049
H	-1.224	3.171	-0.060
C	2.448	1.490	0.079
C	2.460	0.085	0.082
H	1.184	3.216	0.047
H	-3.337	-0.574	-0.172
H	-3.361	1.920	-0.164
H	3.384	2.043	0.104
H	3.582	-0.445	0.129
N	1.446	-2.120	0.124
O	2.379	-2.622	-0.479
O	0.633	-2.744	0.772
O	4.690	-0.970	0.040
H	4.389	-1.815	-0.340

Table S40 Frequencies (cm^{-1}) of hydrogen abstraction transition state between hydroxyl radical and α-nitronaphtalene molecule, calculated at the M06-2X/cc-pVDZ level of theory.

-1628	52	56	98	157	186	209	225
295	347	366	383	413	470	493	532

558	619	634	672	738	765	777	804
816	836	870	888	902	909	994	1014
1028	1032	1063	1155	1160	1176	1191	1234
1283	1326	1385	1407	1425	1468	1474	1493
1557	1646	1665	1696	1712	3205	3208	3218
3232	3234	3272	3767				

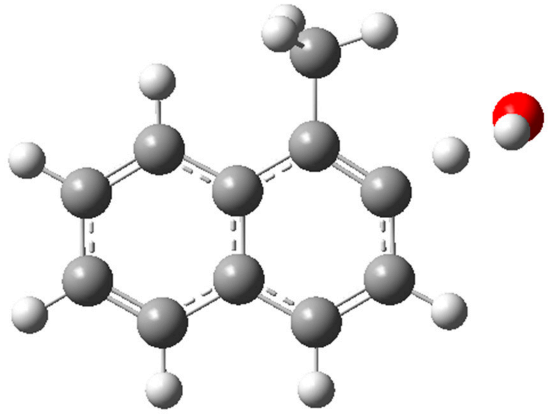

Figure S21 Visualization of hydrogen abstraction transition state between hydroxyl radical and α-methylnaphtalene molecule, calculated at the M06-2X/cc-pVDZ level of theory.

Table S41 Geometry (Å) of hydrogen abstraction transition state between hydroxyl radical and α-methylnaphtalene molecule, calculated at the M06-2X/cc-pVDZ level of theory

Atom	x	y	z
C	-8.996	-0.798	-0.191
C	-7.636	-0.595	-0.195
C	-7.094	0.718	-0.150
C	-7.990	1.826	-0.100
C	-9.390	1.584	-0.097
C	-9.885	0.303	-0.142
H	-9.394	-1.811	-0.226
H	-6.964	-1.451	-0.233
C	-5.676	0.950	-0.158
C	-7.474	3.152	-0.055
H	-10.067	2.437	-0.059
H	-10.960	0.130	-0.139
C	-6.119	3.369	-0.059
C	-5.258	2.252	-0.100
H	-8.171	3.989	-0.016
H	-5.708	4.376	-0.021
O	-2.819	2.687	-0.404
H	-2.936	2.795	-1.364
H	-4.074	2.462	-0.077
C	-4.700	-0.194	-0.216
H	-4.832	-0.863	0.644
H	-4.848	-0.790	-1.126

**H**	-3.674	0.187	-0.208

Table S42 Frequencies (cm^{-1}) of hydrogen abstraction transition state between hydroxyl radical and α-methylnaphtalene molecule, calculated at the M06-2X/cc-pVDZ level of theory.

-1074	66	84	135	185	191	221	250
280	382	430	440	479	489	551	571
576	657	717	759	780	794	830	858
872	896	987	998	1003	1022	1048	1052
1075	1096	1151	1158	1173	1202	1230	1276
1286	1362	1398	1423	1435	1457	1472	1482
1501	1562	1652	1666	1696	3060	3124	3171
3200	3204	3212	3224	3228	3235	3791	

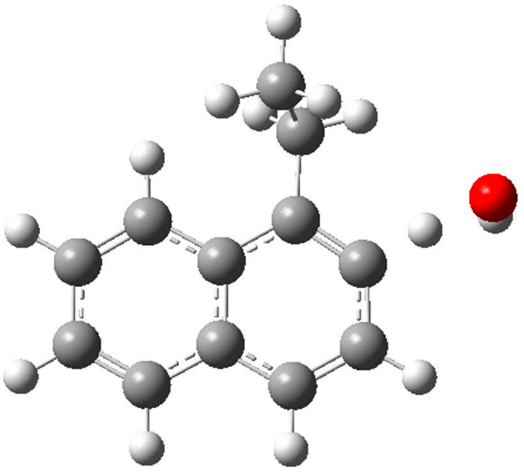

Figure S22 Visualization of hydrogen abstraction transition state between hydroxyl radical and α-ethylnaphtalene molecule, calculated at the M06-2X/cc-pVDZ level of theory.

Table S43 Geometry (Å) of hydrogen abstraction transition state between hydroxyl radical and α-ethylnaphtalene molecule, calculated at the M06-2X/cc-pVDZ level of theory

Atom	x	y	z
**C**	-2.221	-0.444	0.186
**C**	-1.074	0.311	0.127
**C**	-1.135	1.730	0.043
**C**	-2.417	2.354	0.022
**C**	-3.585	1.546	0.084
**C**	-3.493	0.178	0.164
**H**	-0.106	-0.185	0.143
**C**	0.049	2.543	-0.011
**C**	-2.516	3.771	-0.058
**H**	-4.559	2.035	0.069
**C**	-1.382	4.544	-0.113
**C**	-0.129	3.899	-0.096
**H**	-3.503	4.232	-0.076
**H**	-2.153	-1.529	0.250

**H**	-4.394	-0.431	0.211
**H**	0.851	4.588	-0.173
**H**	-1.440	5.629	-0.177
**C**	1.431	1.937	-0.033
**H**	1.501	1.138	0.717
**H**	2.152	2.712	0.251
**C**	1.796	1.395	-1.420
**H**	2.796	0.946	-1.409
**H**	1.078	0.638	-1.756
**H**	1.793	2.211	-2.153
**O**	1.896	5.351	0.092
**H**	1.757	5.458	1.049

Table S44 Frequencies (cm^{-1}) of hydrogen abstraction transition state between hydroxyl radical and α-ethylnaphtalene molecule, calculated at the M06-2X/cc-pVDZ level of theory.

-1046	60	78	94	112	130	185	194
210	299	327	393	440	448	481	495
566	571	602	673	714	758	780	785
807	830	860	873	897	970	986	1000
1019	1041	1057	1084	1090	1104	1151	1158
1177	1196	1228	1270	1277	1290	1348	1374
1385	1422	1434	1464	1471	1475	1488	1498
1562	1654	1664	1699	3065	3082	3128	3148
3151	3199	3203	3211	3224	3226	3234	3786

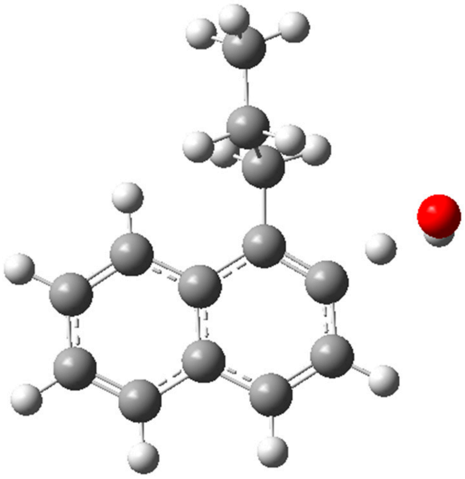

Figure S23 Visualization of hydrogen abstraction transition state between hydroxyl radical and α-propylnaphtalene molecule, calculated at the M06-2X/cc-pVDZ level of theory.

Table S45 Geometry (Å) of hydrogen abstraction transition state between hydroxyl radical and α-propylnaphtalene molecule, calculated at the M06-2X/cc-pVDZ level of theory

Atom	x	y	z
**C**	1.201	2.045	-0.073

**C**	2.429	1.439	-0.031
**C**	2.448	0.002	-0.004
**C**	1.216	-0.717	-0.002
**C**	-0.018	-0.010	-0.035
**C**	-0.032	1.362	-0.067
**H**	4.614	-0.208	0.025
**H**	1.172	3.244	-0.129
**C**	3.663	-0.737	0.029
**C**	1.241	-2.138	0.034
**H**	-0.950	-0.575	-0.036
**H**	-0.968	1.918	-0.096
**C**	2.430	-2.822	0.066
**C**	3.655	-2.111	0.064
**H**	0.293	-2.676	0.036
**H**	2.437	-3.911	0.093
**H**	4.596	-2.659	0.089
**C**	3.699	2.250	-0.070
**H**	4.429	1.845	0.645
**H**	3.470	3.275	0.246
**C**	4.318	2.295	-1.474
**H**	4.474	1.272	-1.843
**H**	3.596	2.767	-2.154
**C**	5.634	3.066	-1.487
**H**	6.372	2.589	-0.828
**H**	6.059	3.110	-2.497
**H**	5.484	4.095	-1.136
**O**	1.128	4.532	0.159
**H**	1.007	4.460	1.121

Table S46 Frequencies (cm^{-1}) of hydrogen abstraction transition state between hydroxyl radical and α-propylnaphtalene molecule, calculated at the M06-2X/cc-pVDZ level of theory.

-1044	50	60	79	85	106	147	175
195	241	283	298	323	391	439	444
488	510	566	572	615	669	729	744
758	781	804	828	853	872	893	896
913	979	993	1011	1037	1057	1073	1090
1106	1115	1151	1158	1176	1196	1225	1248
1272	1286	1300	1312	1371	1374	1394	1422
1433	1464	1469	1476	1477	1489	1498	1562
1653	1663	1698	3057	3063	3074	3101	3124
3135	3144	3199	3203	3211	3223	3227	3232
3786							

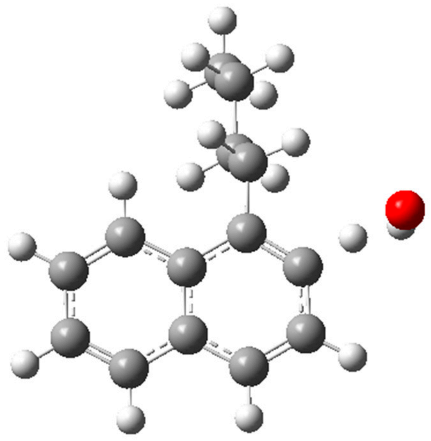

Figure S24 Visualization of hydrogen abstraction transition state between hydroxyl radical and α-butylnaphtalene molecule, calculated at the M06-2X/cc-pVDZ level of theory.

Table S47 Geometry (Å) of hydrogen abstraction transition state between hydroxyl radical and α-butylnaphtalene molecule, calculated at the M06-2X/cc-pVDZ level of theory

Atom	x	y	z
C	1.216	2.039	-0.042
C	2.439	1.423	-0.026
C	2.448	-0.014	-0.023
C	1.211	-0.724	-0.022
C	-0.018	-0.007	-0.024
C	-0.022	1.365	-0.027
H	4.613	-0.240	-0.013
H	1.200	3.241	-0.067
C	3.659	-0.762	-0.017
C	1.226	-2.146	-0.020
H	-0.954	-0.565	-0.024
H	-0.954	1.928	-0.032
C	2.411	-2.839	-0.016
C	3.641	-2.137	-0.014
H	0.274	-2.677	-0.023
H	2.410	-3.928	-0.014
H	4.578	-2.692	-0.011
C	3.714	2.226	0.020
H	4.456	1.797	-0.669
H	3.499	3.243	-0.329
C	4.306	2.303	1.433
H	4.446	1.289	1.836
H	3.578	2.799	2.092
C	5.629	3.060	1.467
H	6.348	2.553	0.807
H	5.478	4.066	1.049
C	6.207	3.165	2.875
H	6.385	2.167	3.298
H	7.159	3.709	2.879

**H**	5.513	3.693	3.541
**O**	1.226	4.512	0.278
**H**	1.193	4.401	1.244

Table S48 Frequencies (cm^{-1}) of hydrogen abstraction transition state between hydroxyl radical and α-butylnaphtalene molecule, calculated at the M06-2X/cc-pVDZ level of theory.

-1080	36	57	68	73	82	135	156
166	194	232	242	272	318	379	400
444	452	490	508	566	576	602	680
720	730	758	780	788	806	827	860
880	894	915	931	979	992	1009	1031
1047	1060	1087	1091	1110	1128	1154	1162
1179	1205	1220	1235	1260	1283	1291	1301
1315	1349	1372	1389	1396	1424	1435	1464
1466	1469	1476	1481	1490	1499	1562	1653
1665	1699	3044	3053	3058	3074	3077	3097
3129	3132	3142	3199	3201	3211	3223	3227
3233	3792						

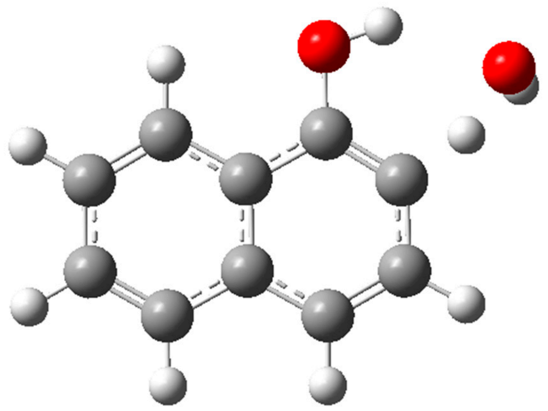

Figure S25 Visualization of hydrogen abstraction transition state between hydroxyl radical and α-hydroxynaphtalene molecule, calculated at the M06-2X/cc-pVDZ level of theory.

Table S49 Geometry (Å) of hydrogen abstraction transition state between hydroxyl radical and α-hydroxynaphtalene molecule, calculated at the M06-2X/cc-pVDZ level of theory

Atom	x	y	z
**C**	-2.383	-0.049	0.015
**C**	-1.184	-0.720	-0.015
**C**	0.036	0.004	-0.026
**C**	0.022	1.427	-0.002
**C**	-1.237	2.086	0.028
**C**	-2.408	1.368	0.036
**H**	-1.147	-1.807	-0.031
**C**	1.299	-0.666	-0.047
**C**	1.246	2.157	-0.001

**H**	-1.256	3.175	0.045
**C**	2.451	1.496	-0.032
**C**	2.446	0.089	-0.088
**H**	1.215	3.245	0.013
**H**	-3.319	-0.605	0.020
**H**	-3.365	1.888	0.058
**H**	3.390	2.044	-0.045
**H**	3.468	-0.570	-0.176
**O**	1.304	-2.016	-0.036
**H**	2.230	-2.309	-0.008
**O**	4.153	-1.625	0.158
**H**	4.312	-1.423	1.096

Table S50 Frequencies (cm^{-1}) of hydrogen abstraction transition state between hydroxyl radical and α-hydroxynaphtalene molecule, calculated at the M06-2X/cc-pVDZ level of theory.

-1241	79	129	144	197	241	295	303
354	438	474	483	510	553	587	595
661	675	734	736	763	798	803	835
887	915	977	1000	1026	1053	1074	1130
1158	1164	1182	1227	1251	1285	1301	1388
1421	1445	1452	1485	1536	1570	1652	1669
1705	3200	3206	3212	3225	3225	3238	3712
3786							

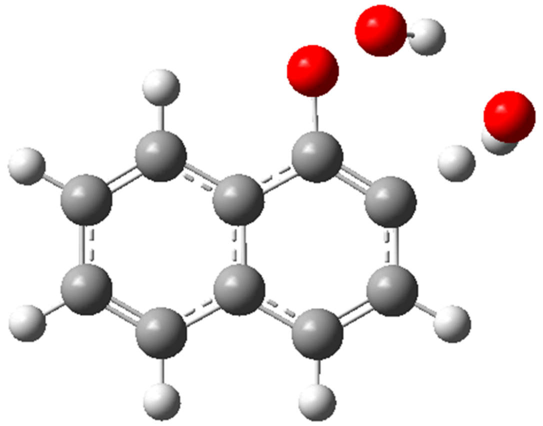

Figure S26 Visualization of hydrogen abstraction transition state between hydroxyl radical and α-peroxynaphtalene molecule, calculated at the M06-2X/cc-pVDZ level of theory.

Table S51 Geometry (Å) of hydrogen abstraction transition state between hydroxyl radical and α-peroxynaphtalene molecule, calculated at the M06-2X/cc-pVDZ level of theory

Atom	x	y	z
**C**	-2.434	0.007	-0.006
**C**	-1.259	-0.700	-0.095
**C**	-0.016	-0.013	-0.097
**C**	0.011	1.408	-0.015

**C**	-1.225	2.104	0.076
**C**	-2.417	1.422	0.084
**H**	-1.262	-1.784	-0.170
**C**	1.229	-0.711	-0.174
**C**	1.255	2.099	-0.034
**H**	-1.209	3.191	0.139
**C**	2.438	1.405	-0.119
**C**	2.398	0.000	-0.201
**H**	1.258	3.187	0.016
**H**	-3.386	-0.520	-0.009
**H**	-3.356	1.967	0.154
**H**	3.394	1.925	-0.135
**H**	3.448	-0.624	-0.235
**O**	1.156	-2.079	-0.161
**O**	2.157	-2.658	-0.981
**H**	2.953	-2.615	-0.418
**O**	4.344	-1.418	0.238
**H**	4.298	-1.188	1.182

Table S52 Frequencies (cm^{-1}) of hydrogen abstraction transition state between hydroxyl radical and α-peroxynaphtalene molecule, calculated at the M06-2X/cc-pVDZ level of theory.

-1328	46	71	129	175	189	220	250
290	345	356	436	456	489	517	549
568	586	626	677	754	760	785	814
830	840	876	911	982	1000	1003	1026
1052	1092	1134	1158	1170	1173	1230	1236
1287	1353	1372	1428	1438	1463	1500	1506
1556	1650	1668	1703	3204	3210	3216	3228
3228	3244	3666	3792				

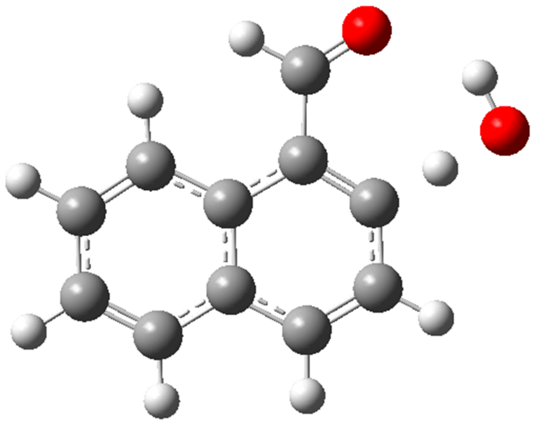

Figure S27 Visualization of hydrogen abstraction transition state between hydroxyl radical and α-naphtaldehyde molecule, calculated at the M06-2X/cc-pVDZ level of theory.

Table S53 Geometry (Å) of hydrogen abstraction transition state between hydroxyl radical and α-naphtaldehyde molecule, calculated at the M06-2X/cc-pVDZ level of theory

Atom	x	y	z
C	-2.434	0.043	0.003
C	-1.259	-0.671	0.003
C	0.001	-0.007	0.001
C	0.004	1.421	-0.001
C	-1.229	2.129	0.000
C	-2.425	1.458	0.001
H	-1.315	-1.756	0.004
C	1.269	-0.692	0.000
C	1.236	2.128	-0.003
H	-1.202	3.218	-0.002
C	2.440	1.462	-0.003
C	2.421	0.059	-0.002
H	1.211	3.218	-0.004
H	-3.384	-0.488	0.004
H	-3.365	2.007	0.002
H	3.386	1.999	-0.005
H	3.536	-0.476	-0.002
C	1.353	-2.175	0.002
O	2.390	-2.804	0.001
H	0.396	-2.728	0.003
O	4.626	-1.067	-0.003
H	4.236	-1.963	-0.001

Table S54 Frequencies (cm^{-1}) of hydrogen abstraction transition state between hydroxyl radical and α-naphtaldehyde molecule, calculated at the M06-2X/cc-pVDZ level of theory.

-1581	61	85	118	148	200	235	253
349	381	417	432	444	504	527	532
596	642	653	754	757	782	790	828
872	887	896	915	995	1021	1029	1042
1043	1063	1152	1158	1188	1205	1234	1283
1338	1388	1416	1422	1453	1470	1496	1564
1649	1654	1699	1809	3040	3202	3203	3215
3230	3231	3248	3725				

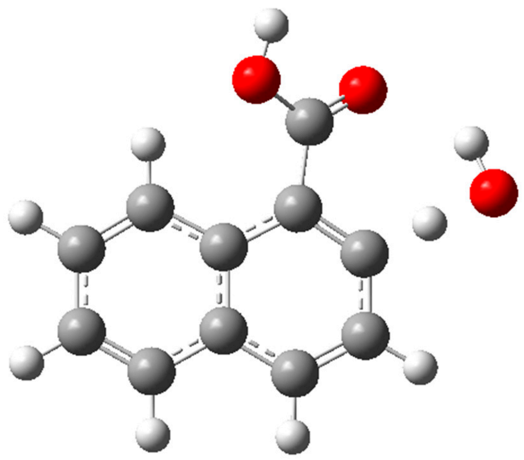

Figure S28 Visualization of hydrogen abstraction transition state between hydroxyl radical and α-naphtalenic acid molecule, calculated at the M06-2X/cc-pVDZ level of theory.

Table S55 Geometry (Å) of hydrogen abstraction transition state between hydroxyl radical and α-naphtalenic aicd molecule, calculated at the M06-2X/cc-pVDZ level of theory

Atom	x	y	z
C	-2.446	0.080	0.139
C	-1.277	-0.642	0.117
C	-0.016	0.011	0.009
C	-0.008	1.439	-0.053
C	-1.237	2.153	-0.038
C	-2.434	1.492	0.053
H	-1.320	-1.723	0.183
C	1.254	-0.669	0.002
C	1.222	2.149	-0.118
H	-1.204	3.241	-0.093
C	2.424	1.486	-0.100
C	2.403	0.083	-0.033
H	1.193	3.237	-0.173
H	-3.397	-0.445	0.225
H	-3.371	2.047	0.067
H	3.372	2.018	-0.139
H	3.517	-0.447	-0.033
C	1.433	-2.152	0.024
O	2.406	-2.717	0.470
O	0.429	-2.845	-0.545
H	0.683	-3.779	-0.498
O	4.629	-0.990	0.104
H	4.276	-1.839	0.431

Table S56 Frequencies (cm^{-1}) of hydrogen abstraction transition state between hydroxyl radical and α-naphtalenic acid molecule, calculated at the M06-2X/cc-pVDZ level of theory.

-1570	47	59	91	160	190	209	225

340	349	371	396	411	467	492	522
543	605	615	640	654	731	750	772
779	812	834	852	885	903	914	995
1016	1021	1030	1065	1149	1160	1163	1187
1224	1238	1284	1333	1385	1391	1426	1431
1468	1498	1564	1651	1660	1699	1823	3201
3203	3213	3230	3231	3283	3744	3802	

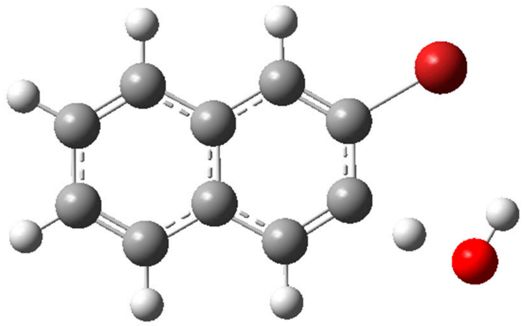

Figure S29 Visualization of hydrogen abstraction transition state between hydroxyl radical and β-bromonaphtalene molecule, calculated at the M06-2X/cc-pVDZ level of theory.

Table S57 Geometry (Å) of hydrogen abstraction transition state between hydroxyl radical and β-bromonaphtalene molecule, calculated at the M06-2X/cc-pVDZ level of theory

Atom	x	y	z
C	-4.578	0.320	1.667
C	-5.318	1.433	1.355
C	-6.416	1.343	0.456
C	-6.738	0.078	-0.115
C	-5.955	-1.057	0.227
C	-4.900	-0.939	1.098
H	-6.949	3.453	0.557
H	-3.739	0.399	2.356
H	-5.073	2.401	1.791
C	-7.194	2.486	0.121
C	-7.839	-0.013	-1.015
H	-6.207	-2.021	-0.214
H	-4.304	-1.813	1.355
C	-8.560	1.107	-1.311
C	-8.246	2.360	-0.750
H	-8.096	-0.974	-1.459
H	-9.500	0.995	-2.088
O	-10.491	1.041	-2.867
H	-10.688	1.991	-2.790
Br	-9.293	3.877	-1.207

Table S58 Frequencies (cm⁻¹) of hydrogen abstraction transition state between hydroxyl radical and β-bromonaphtalene molecule, calculated at the M06-2X/cc-pVDZ level of theory.

-1063	66	73	81	100	129	193	202
209	290	339	372	406	451	473	496
549	577	625	702	704	767	779	783
797	839	870	899	913	930	956	993
1000	1020	1052	1080	1092	1116	1153	1159
1187	1224	1241	1275	1282	1287	1336	1368
1388	1416	1436	1462	1476	1478	1483	1497

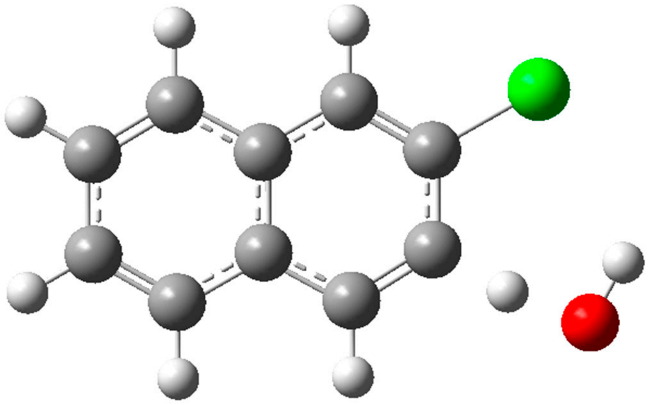

Figure S30 Visualization of hydrogen abstraction transition state between hydroxyl radical and β-chloronaphtalene molecule, calculated at the M06-2X/cc-pVDZ level of theory.

Table S59 Geometry (Å) of hydrogen abstraction transition state between hydroxyl radical and β-chloronaphtalene molecule, calculated at the M06-2X/cc-pVDZ level of theory

Atom	x	y	z
C	-4.571	0.316	1.672
C	-5.242	1.442	1.261
C	-6.364	1.339	0.394
C	-6.782	0.049	-0.043
C	-6.068	-1.097	0.398
C	-4.988	-0.967	1.236
H	-6.757	3.482	0.288
H	-3.713	0.406	2.336
H	-4.924	2.429	1.595
C	-7.071	2.493	-0.042
C	-7.906	-0.056	-0.913
H	-6.393	-2.081	0.060
H	-4.447	-1.851	1.570
C	-8.557	1.076	-1.308
C	-8.148	2.354	-0.879
H	-8.235	-1.037	-1.254
H	-9.522	0.966	-2.056
O	-10.524	1.051	-2.814

H	-10.629	2.018	-2.807
Cl	-9.029	3.760	-1.425

Table S60 Frequencies (cm^{-1}) of hydrogen abstraction transition state between hydroxyl radical and β-chloronaphtalene molecule, calculated at the M06-2X/cc-pVDZ level of theory.

-1477	59	101	105	160	213	217	276
354	385	402	427	492	529	584	614
622	664	765	796	797	802	820	877
879	923	951	959	994	1022	1051	1128
1155	1158	1214	1224	1277	1318	1388	1402
1436	1473	1481	1554	1652	1660	1700	3203
3206	3211	3218	3220	3231	3776		

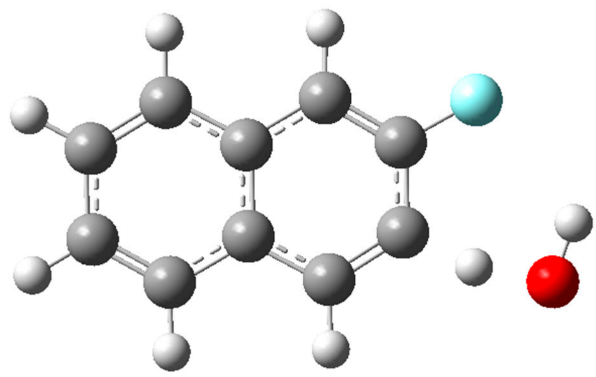

Figure S31 Visualization of hydrogen abstraction transition state between hydroxyl radical and β-fluoronaphtalene molecule, calculated at the M06-2X/cc-pVDZ level of theory.

Table S61 Geometry (Å) of hydrogen abstraction transition state between hydroxyl radical and β-fluoronaphtalene molecule, calculated at the M06-2X/cc-pVDZ level of theory

Atom	x	y	z
C	1.841	3.720	-0.091
C	1.070	2.585	-0.062
C	1.674	1.301	0.010
C	3.096	1.202	0.051
C	3.867	2.396	0.019
C	3.255	3.623	-0.050
H	-0.196	0.178	0.010
H	-0.017	2.651	-0.093
C	0.891	0.109	0.041
C	3.712	-0.079	0.122
H	4.954	2.320	0.050
C	2.920	-1.191	0.150
C	1.519	-1.099	0.110
H	4.796	-0.178	0.155
H	1.370	4.700	-0.146

**H**	3.857	4.530	-0.074
**H**	0.881	-2.154	0.139
**F**	3.472	-2.422	0.218
**O**	0.541	-3.357	0.182
**H**	1.426	-3.761	0.222

Table S62 Frequencies (cm^{-1}) of hydrogen abstraction transition state between hydroxyl radical and β-fluoronaphtalene molecule, calculated at the M06-2X/cc-pVDZ level of theory.

-1510	66	81	124	191	224	285	310
367	406	424	477	493	550	579	626
629	735	768	793	800	820	822	877
909	916	946	993	1016	1020	1051	1147
1157	1174	1212	1267	1276	1337	1409	1416
1439	1480	1514	1568	1663	1677	1704	3202
3205	3212	3219	3224	3231	3780		

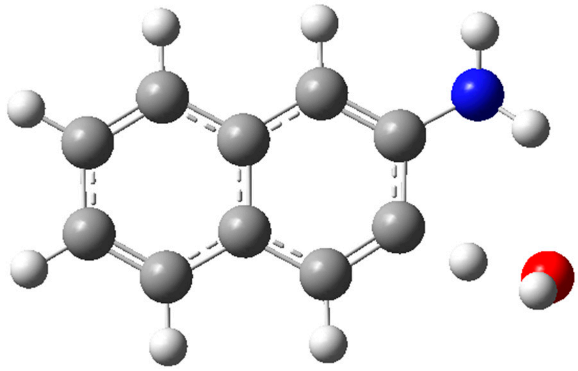

Figure S32 Visualization of hydrogen abstraction transition state between hydroxyl radical and β-aminonaphtalene molecule, calculated

at the M06-2X/cc-pVDZ level of theory.

Table S63 Geometry (Å) of hydrogen abstraction transition state between hydroxyl radical and β-aminonaphtalene molecule, calculated at the M06-2X/cc-pVDZ level of theory

Atom	x	y	z
**C**	2.063	-0.385	0.080
**C**	0.980	0.461	0.092
**C**	1.155	1.872	0.026
**C**	2.484	2.384	-0.051
**C**	3.584	1.487	-0.063
**C**	3.381	0.130	0.002
**H**	-0.031	0.061	0.151
**C**	0.049	2.761	0.039
**C**	2.662	3.798	-0.109
**H**	4.593	1.896	-0.123
**C**	1.567	4.606	-0.117

**C**	0.230	4.131	-0.027
**H**	3.669	4.212	-0.157
**H**	1.909	-1.462	0.129
**H**	4.229	-0.553	-0.008
**H**	1.682	5.808	-0.191
**H**	-0.960	2.351	0.098
**N**	-0.812	5.041	-0.073
**H**	-1.702	4.728	0.285
**H**	-0.567	5.994	0.162
**O**	1.534	7.046	0.201
**H**	1.923	6.967	1.089

Table S64 Frequencies (cm^{-1}) of hydrogen abstraction transition state between hydroxyl radical and β-aminonaphtalene molecule, calculated at the M06-2X/cc-pVDZ level of theory.

-1187	76	105	122	196	244	282	288
349	388	412	430	472	487	517	545
572	626	679	732	762	771	793	826
851	897	911	924	959	985	1014	1050
1108	1150	1158	1188	1200	1228	1278	1302
1328	1380	1422	1438	1490	1516	1560	1630
1652	1680	1698	3190	3197	3200	3204	3216
3230	3582	3709	3788				

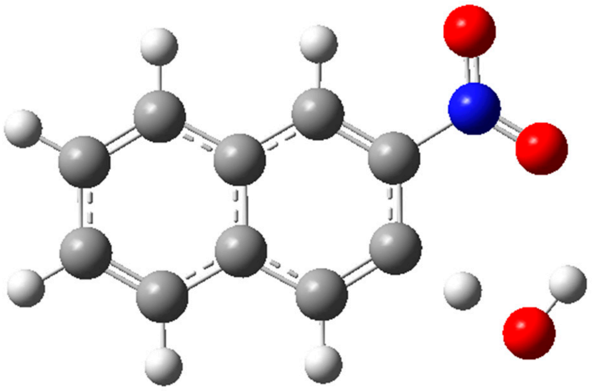

Figure S33 Visualization of hydrogen abstraction transition state between hydroxyl radical and β-nitronaphtalene molecule, calculated at the M06-2X/cc-pVDZ level of theory.

Table S65 Geometry (Å) of hydrogen abstraction transition state between hydroxyl radical and β-nitronaphtalene molecule, calculated at the M06-2X/cc-pVDZ level of theory

Atom	x	y	z
**C**	-4.584	0.306	1.674
**C**	-5.247	1.448	1.303
**C**	-6.368	1.378	0.430
**C**	-6.795	0.108	-0.056
**C**	-6.089	-1.056	0.345

**C**	-5.010	-0.958	1.189
**H**	-6.761	3.528	0.392
**H**	-3.727	0.364	2.342
**H**	-4.927	2.422	1.670
**C**	-7.067	2.547	0.035
**C**	-7.919	0.040	-0.931
**H**	-6.416	-2.026	-0.027
**H**	-4.473	-1.856	1.492
**C**	-8.568	1.184	-1.291
**C**	-8.144	2.438	-0.809
**H**	-8.252	-0.926	-1.308
**H**	-9.535	1.038	-2.055
**O**	-10.491	0.891	-2.811
**H**	-10.719	1.835	-2.872
**O**	-9.806	3.559	-1.953
**O**	-8.447	4.721	-0.759
**N**	-8.852	3.667	-1.203

Table S66 Frequencies (cm^{-1}) of hydrogen abstraction transition state between hydroxyl radical and β-nitronaphtalene molecule, calculated at the M06-2X/cc-pVDZ level of theory.

-1626	45	64	96	137	198	205	256
310	337	366	388	405	495	509	540
589	612	616	655	753	765	790	796
807	822	837	890	913	945	956	999
1003	1031	1052	1137	1161	1163	1222	1240
1280	1321	1397	1408	1448	1468	1478	1482
1563	1652	1662	1698	1716	3206	3210	3212
3222	3233	3237	3779				

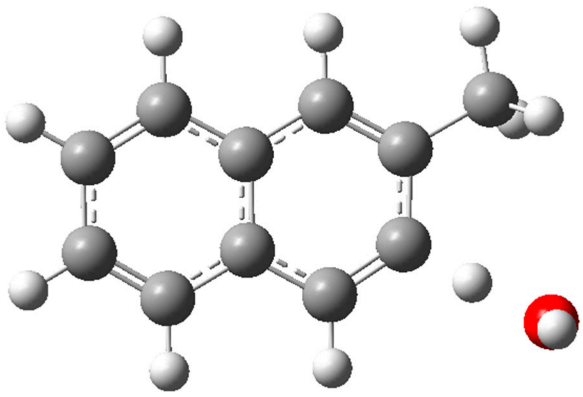

Figure S34 Visualization of hydrogen abstraction transition state between hydroxyl radical and β-methylnaphtalene molecule, calculated at the M06-2X/cc-pVDZ level of theory.

Table S67 Geometry (Å) of hydrogen abstraction transition state between hydroxyl radical and β-methylnaphtalene molecule, calculated at the M06-2X/cc-pVDZ level of theory

Atom	x	y	z
C	-7.830	-0.697	-0.632
C	-6.797	0.202	-0.516
C	-7.054	1.570	-0.231
C	-8.403	1.994	-0.068
C	-9.452	1.045	-0.193
C	-9.172	-0.272	-0.469
H	-4.972	2.177	-0.234
H	-7.620	-1.743	-0.850
H	-5.764	-0.124	-0.641
C	-6.000	2.520	-0.107
C	-8.656	3.368	0.215
H	-10.481	1.379	-0.065
H	-9.981	-0.994	-0.563
C	-7.602	4.225	0.333
C	-6.245	3.846	0.168
H	-9.682	3.714	0.343
H	-7.822	5.376	0.602
C	-5.151	4.869	0.303
H	-5.268	5.661	-0.447
H	-5.194	5.351	1.288
H	-4.166	4.407	0.177
O	-8.062	6.668	0.543
H	-8.272	6.734	-0.405

Table S68 Frequencies (cm^{-1}) of hydrogen abstraction transition state between hydroxyl radical and β-methylnaphtalene molecule, calculated at the M06-2X/cc-pVDZ level of theory.

**-1192**	77	123	128	200	284	296	306
**357**	415	428	488	493	547	572	626
**628**	656	731	756	767	793	812	861
**908**	913	928	967	988	1016	1050	1146
**1158**	1169	1184	1209	1252	1278	1322	1352
**1408**	1436	1452	1494	1532	1567	1659	1681
**1702**	3199	3204	3209	3216	3218	3230	3744
**3780**							

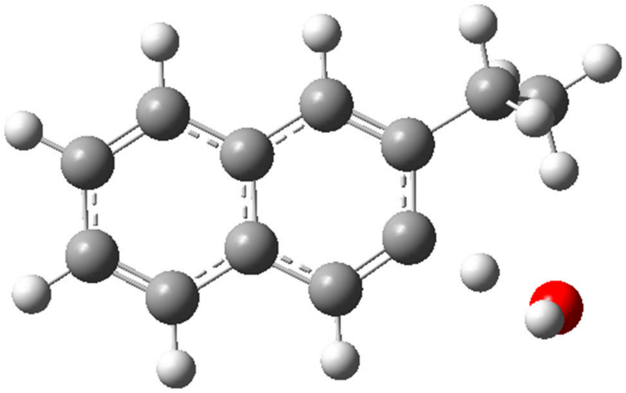

Figure S35 Visualization of hydrogen abstraction transition state between hydroxyl radical and β-ethylnaphtalene molecule, calculated at the M06-2X/cc-pVDZ level of theory.

Table S69 Geometry (Å) of hydrogen abstraction transition state between hydroxyl radical and β-ethylnaphtalene molecule, calculated at the M06-2X/cc-pVDZ level of theory

Atom	x	y	z
C	1.739	3.681	0.012
C	1.065	2.488	-0.086
C	1.772	1.256	-0.066
C	3.191	1.267	0.056
C	3.858	2.518	0.155
C	3.151	3.695	0.133
H	0.019	-0.029	-0.249
H	-0.021	2.469	-0.180
C	1.105	0.000	-0.161
C	3.902	0.034	0.078
H	4.944	2.527	0.248
C	3.257	-1.179	-0.017
C	1.846	-1.145	-0.149
H	4.989	0.060	0.174
H	1.190	4.622	-0.005
H	3.674	4.647	0.209
H	1.282	-2.199	-0.254
C	3.988	-2.497	-0.029
H	5.037	-2.326	0.244
H	3.551	-3.157	0.733
C	3.903	-3.184	-1.395
H	4.323	-2.540	-2.177
H	4.459	-4.129	-1.386
H	2.860	-3.405	-1.648
O	0.686	-3.351	-0.010
H	0.467	-3.205	0.927

Table S70 Frequencies (cm^{-1}) of hydrogen abstraction transition state between hydroxyl radical and β-ethylnaphtalene molecule, calculated at the M06-2X/cc-pVDZ level of theory.

-1063	66	73	81	100	129	193	202
209	290	339	372	406	451	473	496
549	577	625	702	704	767	779	783
797	839	870	899	913	930	956	993
1000	1020	1052	1080	1092	1116	1153	1159
1187	1224	1241	1275	1282	1287	1336	1368
1388	1416	1436	1462	1476	1478	1483	1497
1552	1653	1676	1704	3065	3072	3118	3147
3153	3185	3198	3202	3206	3216	3229	3787

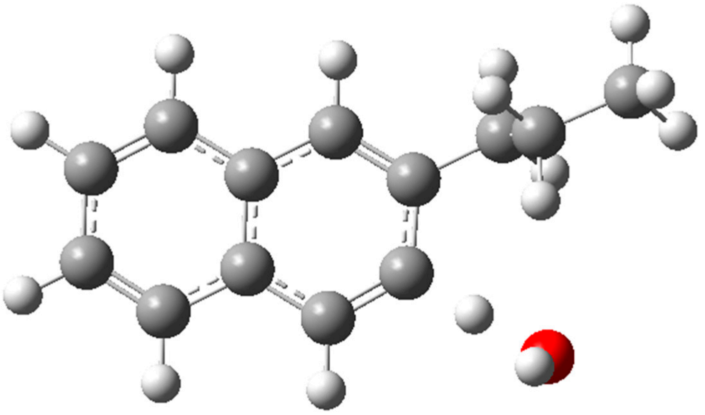

Figure S36 Visualization of hydrogen abstraction transition state between hydroxyl radical and β-propylnaphtalene molecule, calculated at the M06-2X/cc-pVDZ level of theory.

Table S71 Geometry (Å) of hydrogen abstraction transition state between hydroxyl radical and β-propylnaphtalene molecule, calculated at the M06-2X/cc-pVDZ level of theory

Atom	x	y	z
C	-1.239	2.152	-0.026
C	-2.418	1.440	-0.003
C	-2.437	0.017	-0.002
C	-1.207	-0.700	-0.027
C	0.013	0.037	-0.052
C	-0.038	1.400	-0.061
H	-4.597	-0.168	0.041
H	-3.370	1.975	0.014
C	-3.655	-0.716	0.022
C	-1.228	-2.120	-0.028
H	0.964	-0.494	-0.079
H	0.990	2.017	-0.123
C	-2.422	-2.800	-0.003
C	-3.648	-2.089	0.022
H	-0.282	-2.660	-0.050
H	-2.430	-3.889	-0.004

**H**	-4.588	-2.639	0.041
**C**	-1.196	3.656	0.023
**H**	-0.406	4.019	-0.649
**H**	-2.152	4.062	-0.334
**C**	-0.919	4.178	1.437
**H**	0.018	3.734	1.799
**H**	-1.716	3.829	2.108
**C**	-0.819	5.699	1.474
**H**	-0.631	6.062	2.491
**H**	0.001	6.045	0.831
**H**	-1.749	6.160	1.115
**O**	2.084	2.705	0.143
**H**	2.170	2.477	1.085

Table S72 Frequencies (cm^{-1}) of hydrogen abstraction transition state between hydroxyl radical and β-propylnaphtalene molecule, calculated at the M06-2X/cc-pVDZ level of theory.

-1065	50	54	70	73	93	155	174
200	241	267	295	322	374	416	444
495	511	559	575	632	674	729	756
765	777	797	834	866	873	895	914
922	932	979	992	1018	1052	1071	1092
1112	1117	1153	1159	1186	1221	1233	1252
1272	1281	1296	1310	1364	1373	1393	1415
1437	1461	1469	1476	1480	1482	1495	1552
1653	1674	1704	3059	3061	3071	3098	3120
3136	3143	3181	3197	3200	3204	3216	3229
3788							

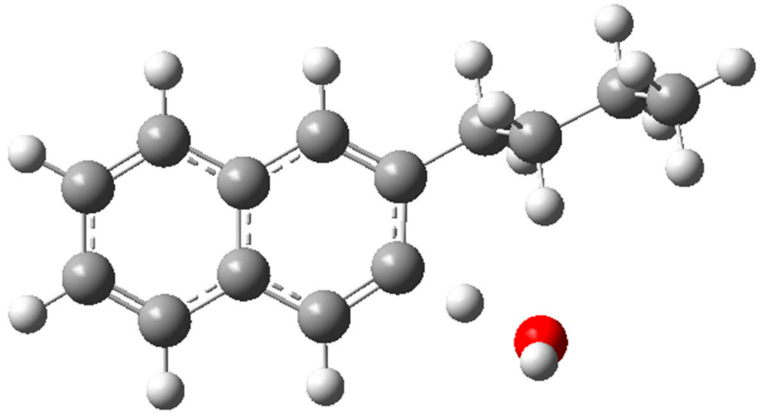

Figure S37 Visualization of hydrogen abstraction transition state between hydroxyl radical and β-butylnaphtalene molecule, calculated at the M06-2X/cc-pVDZ level of theory.

Table S73 Geometry (Å) of hydrogen abstraction transition state between hydroxyl radical and β-butylnaphtalene molecule, calculated at the M06-2X/cc-pVDZ level of theory

Atom	x	y	z
C	-1.237	2.151	-0.026
C	-2.416	1.440	0.005
C	-2.435	0.017	0.002
C	-1.206	-0.701	-0.037
C	0.014	0.035	-0.070
C	-0.037	1.398	-0.074
H	-4.595	-0.166	0.064
H	-3.367	1.975	0.032
C	-3.654	-0.715	0.035
C	-1.229	-2.121	-0.042
H	0.965	-0.496	-0.107
H	0.991	2.016	-0.141
C	-2.422	-2.800	-0.009
C	-3.648	-2.089	0.031
H	-0.283	-2.662	-0.074
H	-2.432	-3.889	-0.013
H	-4.588	-2.638	0.057
C	-1.192	3.655	0.028
H	-0.407	4.019	-0.648
H	-2.150	4.062	-0.320
C	-0.904	4.170	1.442
H	0.036	3.725	1.800
H	-1.698	3.824	2.121
C	-0.797	5.690	1.497
H	0.006	6.014	0.819
H	-1.729	6.131	1.113
C	-0.525	6.207	2.906
H	0.417	5.797	3.294
H	-0.452	7.301	2.926
H	-1.329	5.909	3.592
O	2.085	2.706	0.122
H	2.178	2.472	1.061

Table S74 Frequencies (cm^{-1}) of hydrogen abstraction transition state between hydroxyl radical and β-butylnaphtalene molecule, calculated at the M06-2X/cc-pVDZ level of theory.

-1053	37	50	60	70	88	119	144
156	201	237	240	268	298	375	394
416	455	497	510	558	575	632	679
718	754	766	778	783	797	842	869
903	906	920	928	937	977	993	1019
1044	1052	1088	1096	1114	1128	1154	1160
1187	1218	1225	1247	1257	1274	1285	1299
1315	1344	1366	1388	1395	1416	1439	1458

1467	1474	1476	1481	1486	1495	1553	1654
1675	1705	3048	3056	3058	3068	3082	3100
3121	3132	3141	3182	3196	3200	3205	3215
3227	3787						

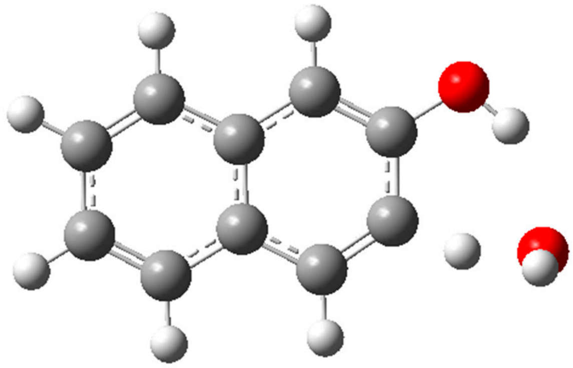

Figure S38 Visualization of hydrogen abstraction transition state between hydroxyl radical and β-hydroxynaphtalene molecule, calculated at the M06-2X/cc-pVDZ level of theory.

Table S75 Geometry (Å) of hydrogen abstraction transition state between hydroxyl radical and β-hydroxynaphtalene molecule, calculated at the M06-2X/cc-pVDZ level of theory

Atom	x	y	z
C	-3.168	0.475	-0.054
C	-1.884	0.960	-0.070
C	-0.769	0.075	-0.046
C	-1.020	-1.328	-0.003
C	-2.359	-1.802	0.012
C	-3.412	-0.922	-0.012
H	0.759	1.634	-0.089
H	-4.010	1.166	-0.073
H	-1.700	2.034	-0.103
C	0.564	0.563	-0.060
C	0.091	-2.221	0.024
H	-2.535	-2.877	0.043
H	-4.436	-1.292	0.000
C	1.350	-1.702	-0.011
C	1.626	-0.312	-0.035
H	-0.081	-3.296	0.059
H	2.337	-2.405	-0.043
O	3.585	-2.669	0.260
H	3.474	-2.845	1.211
O	2.898	0.149	-0.042
H	3.500	-0.612	0.007

Table S76 Frequencies (cm^{-1}) of hydrogen abstraction transition state between hydroxyl radical and β-hydroxynaphtalene molecule, calculated at the M06-2X/cc-pVDZ level of theory.

-1192	77	123	128	200	284	296	306
357	415	428	488	493	547	572	626
628	656	731	756	767	793	812	861
908	913	928	967	988	1016	1050	1146
1158	1169	1184	1209	1252	1278	1322	1352
1408	1436	1452	1494	1532	1567	1659	1681
1702	3199	3204	3209	3216	3218	3230	3744
3780							

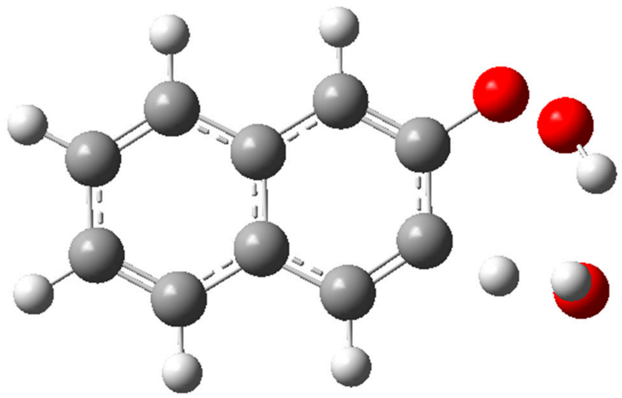

Figure S39 Visualization of hydrogen abstraction transition state between hydroxyl radical and β-peroxynaphtalene molecule, calculated at the M06-2X/cc-pVDZ level of theory.

Table S77 Geometry (Å) of hydrogen abstraction transition state between hydroxyl radical and β-peroxynaphtalene molecule, calculated at the M06-2X/cc-pVDZ level of theory

Atom	x	y	z
C	3.607	0.729	0.177
C	2.434	1.440	0.092
C	1.191	0.767	-0.045
C	1.168	-0.656	-0.093
C	2.398	-1.363	-0.002
C	3.587	-0.687	0.129
H	-0.038	2.571	-0.070
H	2.442	2.529	0.127
C	-0.042	1.483	-0.127
C	-0.072	-1.338	-0.243
H	2.381	-2.452	-0.041
C	-1.242	-0.629	-0.318
H	-0.105	-2.425	-0.299
H	4.556	1.251	0.282
H	4.522	-1.240	0.197
O	-2.437	-1.305	-0.361
O	-3.219	-0.815	-1.449
H	-3.695	-0.073	-1.032

**C**	-1.202	0.785	-0.266
**H**	-2.266	1.360	-0.327
**O**	-3.511	1.646	-0.060
**H**	-3.500	1.512	0.904

Table S78 Frequencies (cm^{-1}) of hydrogen abstraction transition state between hydroxyl radical and β-peroxynaphtalene molecule, calculated at the M06-2X/cc-pVDZ level of theory.

-1222	60	83	111	181	197	225	271
319	355	361	411	439	492	510	562
580	586	623	705	741	772	778	797
831	871	902	918	933	965	993	1015
1020	1053	1116	1152	1159	1181	1214	1263
1281	1328	1361	1416	1437	1481	1492	1501
1558	1658	1671	1702	3202	3206	3211	3219
3220	3230	3680	3781				

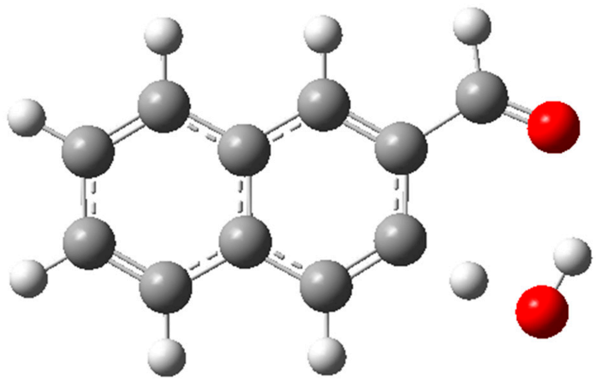

Figure S40 Visualization of hydrogen abstraction transition state between hydroxyl radical and β-naphtaldehyde molecule, calculated at the M06-2X/cc-pVDZ level of theory.

Table S79 Geometry (Å) of hydrogen abstraction transition state between hydroxyl radical and β-naphtaldehyde molecule, calculated at the M06-2X/cc-pVDZ level of theory

Atom	x	y	z
**C**	2.418	1.409	-0.001
**C**	2.404	-0.008	-0.002
**C**	1.242	2.120	0.000
**C**	1.211	-0.687	-0.001
**C**	-0.022	0.021	0.000
**C**	-0.007	1.446	0.001
**C**	-1.246	2.157	0.002
**H**	-1.237	3.247	0.003
**C**	-2.418	1.464	0.003
**C**	-2.460	0.047	0.002
**C**	-1.271	-0.653	0.001
**H**	1.252	3.209	0.001

**H**	1.192	-1.777	-0.002
**H**	-1.287	-1.745	0.000
**O**	-4.837	-0.227	0.004
**C**	-3.729	-0.715	0.003
**H**	-3.468	2.123	0.004
**H**	-3.605	-1.820	0.002
**H**	3.372	1.936	-0.001
**H**	3.345	-0.556	-0.003
**O**	-4.596	2.631	0.004
**H**	-5.086	1.787	0.004

Table S80 Frequencies (cm^{-1}) of hydrogen abstraction transition state between hydroxyl radical and β-naphtaldehyde molecule, calculated at the M06-2X/cc-pVDZ level of theory.

-1556	69	76	135	167	210	222	301
350	373	397	405	444	498	532	580
618	638	647	767	771	802	802	829
832	886	900	940	957	1000	1015	1029
1043	1053	1157	1160	1188	1226	1271	1284
1327	1397	1416	1419	1448	1480	1496	1558
1652	1671	1702	1817	2984	3188	3205	3209
3211	3221	3232	3706				

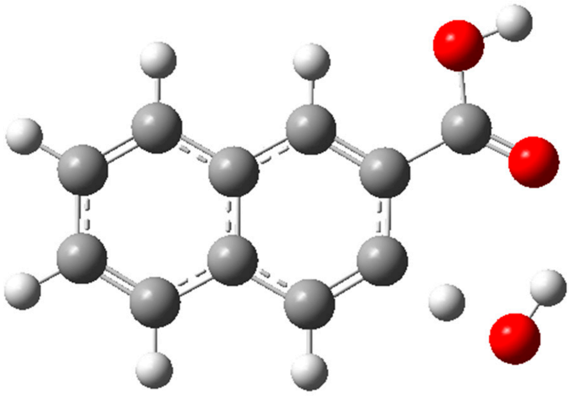

Figure S41 Visualization of hydrogen abstraction transition state between hydroxyl radical and β-naphtalenic acid molecule, calculated at the M06-2X/cc-pVDZ level of theory.

Table S81 Geometry (Å) of hydrogen abstraction transition state between hydroxyl radical and β-naphtalenic aicd molecule, calculated at the M06-2X/cc-pVDZ level of theory

Atom	x	y	z
**C**	-2.405	1.441	0.000
**C**	-2.415	0.023	0.000
**C**	-1.214	2.124	0.000
**C**	-1.237	-0.684	0.000
**C**	0.010	-0.004	0.000
**C**	0.022	1.420	0.000

C	1.264	2.105	0.000
H	1.273	3.194	0.000
C	2.456	1.410	0.000
C	2.419	-0.006	0.000
C	1.251	-0.708	0.000
H	-1.198	3.213	0.000
H	-1.243	-1.773	0.000
H	1.253	-1.797	0.000
H	3.459	-0.675	0.000
C	3.762	2.121	0.000
O	4.849	1.588	0.000
O	3.634	3.462	0.000
H	4.531	3.826	0.000
H	-3.348	1.986	0.000
H	-3.367	-0.506	0.000
O	4.556	-1.252	0.000
H	5.088	-0.434	0.000

Table S82 Frequencies (cm^{-1}) of hydrogen abstraction transition state between hydroxyl radical and β-naphtalenic acid molecule, calculated at the M06-2X/cc-pVDZ level of theory.

-1572	61	69	95	140	194	208	249
335	365	378	394	437	496	506	513
579	594	610	641	643	734	758	800
801	802	816	839	894	898	950	957
990	1002	1030	1053	1141	1159	1160	1209
1228	1259	1285	1330	1391	1404	1421	1445
1478	1501	1559	1650	1670	1703	1832	3203
3207	3211	3219	3227	3230	3730	3824	

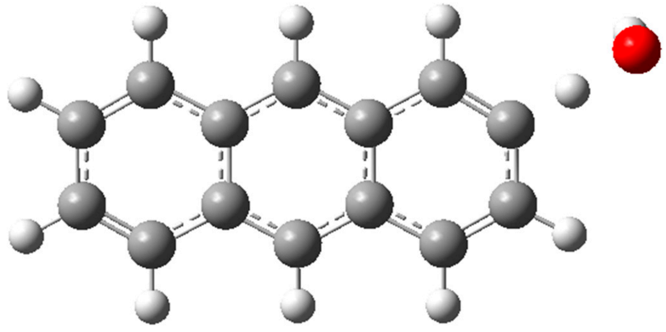

Figure S42 Visualization of hydrogen abstraction transition state between hydroxyl radical and antracene molecule, calculated at the M06-2X/cc-pVDZ level of theory.

Table S83 Geometry (Å) of hydrogen abstraction transition state between hydroxyl radical and antracene molecule, calculated at the M06-2X/cc-pVDZ level of theory

Atom	x	y	z

**C**	1.225	3.498	0.039
**C**	1.239	2.133	0.030
**C**	0.016	1.387	0.008
**C**	-1.230	2.102	-0.005
**C**	-1.202	3.535	0.004
**C**	-0.015	4.210	0.026
**C**	0.003	-0.012	-0.002
**C**	-2.431	1.386	-0.028
**C**	-2.443	-0.012	-0.037
**C**	-1.197	-0.729	-0.024
**C**	-1.218	-2.163	-0.032
**H**	-0.268	-2.698	-0.023
**C**	-2.399	-2.851	-0.055
**C**	-3.616	-2.116	-0.076
**C**	-3.670	-0.759	-0.056
**H**	0.949	-0.554	0.008
**H**	2.160	4.055	0.056
**H**	2.182	1.587	0.040
**H**	-2.149	4.074	-0.006
**H**	-0.006	5.299	0.033
**H**	-3.377	1.929	-0.039
**H**	-4.621	-0.228	-0.061
**H**	-4.651	-2.733	-0.121
**H**	-2.418	-3.939	-0.066
**O**	-5.745	-3.372	0.185
**H**	-5.710	-3.277	1.152

Table S84 Frequencies (cm^{-1}) of hydrogen abstraction transition state between hydroxyl radical and antracene molecule, calculated at the M06-2X/cc-pVDZ level of theory.

-1168	52	55	105	131	142	236	245
274	352	392	398	451	485	493	522
553	597	621	634	664	760	763	776
792	798	827	848	866	894	908	926
929	945	994	1003	1015	1036	1044	1097
1133	1150	1166	1180	1197	1266	1276	1287
1303	1332	1368	1381	1452	1466	1480	1492
1522	1612	1642	1653	1696	1712	3194	3196
3199	3202	3203	3209	3217	3228	3229	3792

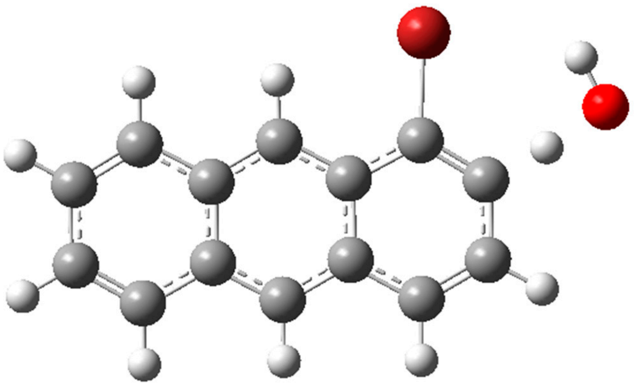

Figure S43 Visualization of hydrogen abstraction transition state between hydroxyl radical and α-bromoantracene molecule, calculated at the M06-2X/cc-pVDZ level of theory.

Table S85 Geometry (Å) of hydrogen abstraction transition state between hydroxyl radical and α-bromoantracene molecule, calculated at the M06-2X/cc-pVDZ level of theory

Atom	x	y	z
C	4.852	1.450	0.000
C	3.671	2.135	0.000
C	2.421	1.437	0.000
C	2.432	0.002	0.000
C	3.690	-0.681	0.000
C	4.862	0.020	0.000
C	1.199	2.120	0.000
C	1.214	-0.685	0.000
C	-0.010	-0.010	0.000
C	-0.016	1.431	0.000
C	-1.303	2.078	0.000
C	-2.444	1.347	0.000
C	-2.444	-0.074	0.000
C	-1.248	-0.731	0.000
H	1.204	3.208	0.000
H	5.798	1.990	0.000
H	3.660	3.225	0.000
H	3.693	-1.771	0.000
H	5.815	-0.507	0.000
H	1.216	-1.775	0.000
H	-1.216	-1.820	0.000
H	-3.390	-0.613	0.000
H	-3.536	1.891	0.000
Br	-1.413	3.977	0.000
O	-4.599	2.582	0.000
H	-4.187	3.464	0.000

Table S86 Frequencies (cm-1) of hydrogen abstraction transition state between hydroxyl radical and α-bromoantracene molecule, calculated at the M06-2X/cc-pVDZ level of theory.

-1452	46	73	102	108	133	175	221
259	263	288	303	380	411	425	488
490	505	562	564	586	625	668	678
737	768	792	792	808	820	874	880
884	920	928	936	944	999	1016	1019
1039	1128	1146	1153	1171	1186	1221	1283
1292	1309	1350	1366	1394	1441	1454	1480
1495	1519	1604	1637	1654	1693	1712	3197
3200	3204	3206	3218	3226	3230	3233	3771

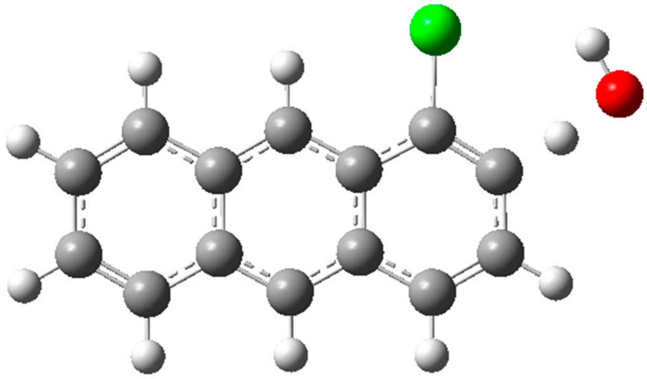

Figure S44 Visualization of hydrogen abstraction transition state between hydroxyl radical and α-chloroantracene molecule, calculated at the M06-2X/cc-pVDZ level of theory.

Table S87 Geometry (Å) of hydrogen abstraction transition state between hydroxyl radical and α-chloroantracene molecule, calculated at the M06-2X/cc-pVDZ level of theory

Atom	x	y	z
C	4.864	1.444	0.001
C	3.687	2.136	0.001
C	2.432	1.445	0.001
C	2.434	0.010	0.001
C	3.690	-0.680	0.001
C	4.865	0.014	0.001
C	1.214	2.135	0.001
C	1.214	-0.672	0.000
C	-0.007	0.009	0.000
C	-0.002	1.449	0.000
C	-1.283	2.107	0.000
C	-2.431	1.386	0.000
C	-2.442	-0.034	0.000
C	-1.251	-0.702	0.000
H	1.221	3.223	0.001
H	5.812	1.978	0.002
H	3.682	3.225	0.001

H	3.686	-1.770	0.001
H	5.815	-0.519	0.001
H	1.211	-1.763	0.000
H	-1.229	-1.791	0.000
H	-3.392	-0.566	-0.001
H	-3.514	1.957	-0.001
Cl	-1.354	3.853	0.000
O	-4.532	2.703	-0.001
H	-4.075	3.563	-0.001

Table S88 Frequencies (cm^{-1}) of hydrogen abstraction transition state between hydroxyl radical and α-chloroantracene molecule, calculated at the M06-2X/cc-pVDZ level of theory.

-1461	48	80	102	106	150	171	194
264	289	290	338	412	417	442	489
490	507	568	569	591	625	666	694
733	766	791	798	810	821	872	878
884	923	924	942	954	997	1011	1018
1037	1132	1149	1153	1173	1186	1222	1288
1292	1313	1353	1365	1398	1441	1456	1480
1497	1518	1606	1639	1656	1696	1714	3199
3203	3207	3210	3220	3228	3231	3234	3775

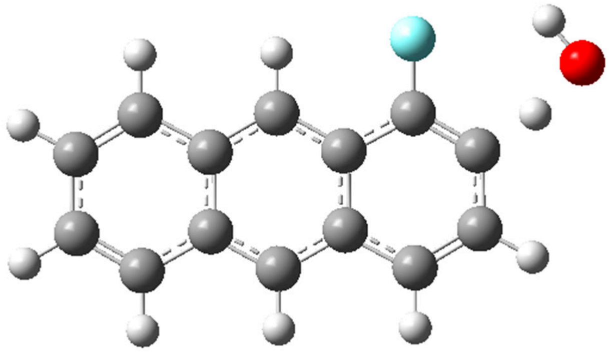

Figure S45 Visualization of hydrogen abstraction transition state between hydroxyl radical and α-fluoroantracene molecule, calculated at the M06-2X/cc-pVDZ level of theory.

Table S89 Geometry (Å) of hydrogen abstraction transition state between hydroxyl radical and α-fluoroantracene molecule, calculated at the M06-2X/cc-pVDZ level of theory

Atom	x	y	z
C	4.828	1.515	-0.001
C	3.639	2.187	-0.001
C	2.397	1.475	0.000
C	2.422	0.038	0.000
C	3.691	-0.628	0.000
C	4.854	0.086	-0.001
C	1.167	2.141	0.000

**C**	1.217	-0.672	0.000
**C**	-0.018	-0.016	0.001
**C**	-0.029	1.421	0.000
**C**	-1.308	2.058	0.000
**C**	-2.460	1.350	0.001
**C**	-2.456	-0.072	0.001
**C**	-1.260	-0.733	0.001
**H**	1.139	3.230	-0.001
**H**	5.768	2.066	-0.001
**H**	3.615	3.276	-0.001
**H**	3.707	-1.718	0.000
**H**	5.813	-0.431	-0.001
**H**	1.240	-1.762	0.001
**H**	-1.234	-1.822	0.001
**H**	-3.399	-0.615	0.001
**H**	-3.523	1.975	0.001
**F**	-1.338	3.409	0.000
**O**	-4.397	2.867	0.001
**H**	-3.814	3.647	0.000

Table S90 Frequencies (cm^{-1}) of hydrogen abstraction transition state between hydroxyl radical and α-fluoroantracene molecule, calculated at the M06-2X/cc-pVDZ level of theory.

-1500	52	74	99	114	193	193	227
269	298	313	356	418	437	489	503
505	520	579	580	610	624	654	723
740	765	789	808	824	825	866	872
897	924	927	940	995	999	1017	1036
1054	1140	1152	1161	1181	1216	1223	1284
1291	1347	1366	1373	1423	1442	1467	1479
1505	1525	1614	1642	1664	1713	1722	3196
3199	3203	3208	3219	3220	3231	3233	3779

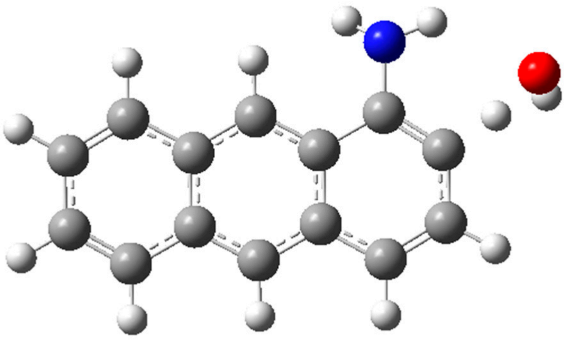

Figure S46 Visualization of hydrogen abstraction transition state between hydroxyl radical and α-aminoantracene molecule, calculated at the M06-2X/cc-pVDZ level of theory.

Table S91 Geometry (Å) of hydrogen abstraction transition state between hydroxyl radical and α-aminoantracene molecule, calculated at the M06-2X/cc-pVDZ level of theory

Atom	x	y	z
C	-1.378	-1.469	-0.044
C	-0.474	-0.406	-0.046
C	-0.968	0.944	0.016
C	-2.346	1.153	0.103
C	-3.255	0.089	0.124
H	-1.024	-2.495	-0.142
C	0.960	-0.619	-0.098
C	-0.050	2.046	-0.019
H	-2.724	2.175	0.153
C	1.294	1.818	-0.122
C	1.766	0.486	-0.199
H	-0.444	3.061	0.014
H	2.960	0.252	-0.286
H	1.998	2.647	-0.174
N	1.479	-1.901	-0.114
H	2.481	-1.934	0.044
H	0.966	-2.596	0.409
O	4.060	-0.264	0.162
H	4.068	0.108	1.059
C	-2.760	-1.254	0.040
C	-3.696	-2.336	0.037
C	-5.039	-2.099	0.123
H	-3.316	-3.355	-0.032
C	-5.532	-0.761	0.214
H	-5.743	-2.929	0.122
C	-4.669	0.298	0.213
H	-6.606	-0.591	0.282
H	-5.042	1.320	0.278

Table S92 Frequencies (cm^{-1}) of hydrogen abstraction transition state between hydroxyl radical and α-aminoantracene molecule, calculated at the M06-2X/cc-pVDZ level of theory.

-1231	58	90	100	122	175	210	243
259	283	320	352	388	413	428	481
489	510	524	562	582	606	626	656
670	740	750	767	778	788	813	818
866	872	911	916	937	975	996	1016
1022	1037	1114	1142	1156	1169	1183	1193
1238	1274	1292	1297	1353	1367	1381	1428
1447	1462	1477	1506	1522	1603	1635	1642
1660	1693	1710	3195	3199	3203	3208	3209
3218	3225	3230	3562	3691	3801		

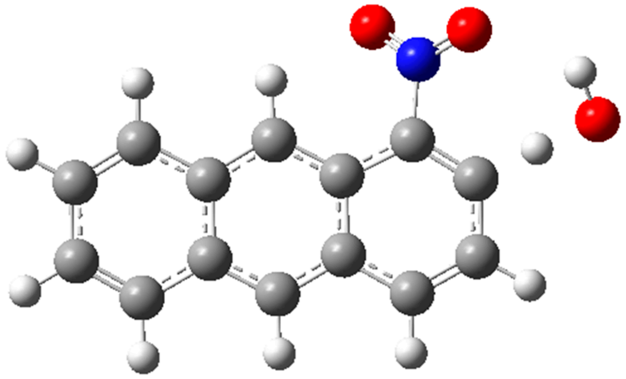

Figure S47 Visualization of hydrogen abstraction transition state between hydroxyl radical and α-nitroantracene molecule, calculated at the M06-2X/cc-pVDZ level of theory.

Table S93 Geometry (Å) of hydrogen abstraction transition state between hydroxyl radical and α-nitroantracene molecule, calculated at the M06-2X/cc-pVDZ level of theory

Atom	x	y	z
C	4.862	1.400	-0.233
C	3.691	2.101	-0.174
C	2.434	1.418	-0.108
C	2.429	-0.016	-0.105
C	3.677	-0.716	-0.167
C	4.855	-0.030	-0.228
C	1.227	2.125	-0.044
C	1.204	-0.684	-0.049
C	-0.007	0.012	0.007
C	0.001	1.454	0.019
C	-1.295	2.089	0.051
C	-2.442	1.360	0.058
C	-2.447	-0.055	0.063
C	-1.246	-0.705	0.042
H	1.261	3.209	-0.034
H	5.812	1.929	-0.283
H	3.693	3.190	-0.175
H	3.666	-1.806	-0.163
H	5.801	-0.568	-0.275
H	1.187	-1.774	-0.056
H	-1.210	-1.795	0.045
H	-3.392	-0.593	0.086
H	-3.561	1.891	0.099
N	-1.424	3.555	0.095
O	-2.375	4.059	-0.477
O	-0.585	4.183	0.709
O	-4.678	2.401	0.008
H	-4.382	3.257	-0.353

Table S94 Frequencies (cm^{-1}) of hydrogen abstraction transition state between hydroxyl radical and α-nitroantracene molecule, calculated at the M06-2X/cc-pVDZ level of theory.

-1631	44	53	69	108	140	167	183
247	269	280	314	327	350	392	427
440	486	502	512	561	577	605	626
672	685	740	762	774	791	792	803
830	855	875	882	901	927	932	941
999	1012	1013	1019	1037	1142	1155	1157
1177	1189	1230	1291	1299	1320	1351	1370
1390	1441	1460	1470	1482	1493	1519	1606
1638	1651	1682	1710	1714	3201	3203	3208
3208	3221	3231	3237	3260	3769		

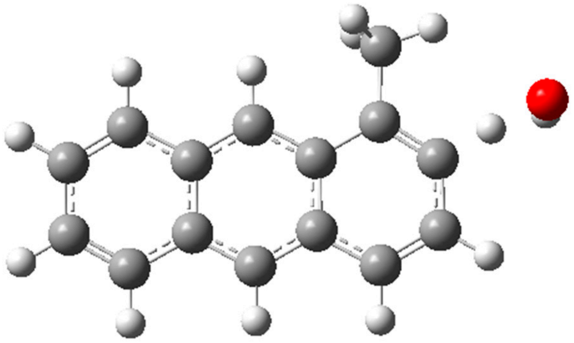

Figure S48 Visualization of hydrogen abstraction transition state between hydroxyl radical and α-methylantracene molecule, calculated at the M06-2X/cc-pVDZ level of theory.

Table S95 Geometry (Å) of hydrogen abstraction transition state between hydroxyl radical and α-methylantracene molecule, calculated at the M06-2X/cc-pVDZ level of theory

Atom	x	y	z
C	4.875	1.397	0.022
C	3.705	2.102	0.003
C	2.443	1.425	-0.003
C	2.429	-0.009	0.010
C	3.676	-0.712	0.030
C	4.860	-0.032	0.036
C	1.229	2.125	-0.023
C	1.199	-0.676	0.003
C	-0.012	0.020	-0.016
C	0.000	1.459	-0.030
C	-1.256	2.179	-0.044
C	-2.398	1.437	-0.063
C	-2.442	0.016	-0.042
C	-1.266	-0.676	-0.022
H	1.258	3.214	-0.034
H	5.830	1.921	0.027
H	3.713	3.191	-0.008

**H**	3.660	-1.802	0.040
**H**	5.804	-0.576	0.052
**H**	1.185	-1.767	0.013
**H**	-1.261	-1.765	-0.014
**H**	-3.401	-0.498	-0.053
**H**	-3.447	2.022	-0.104
**C**	-1.281	3.682	-0.049
**H**	-0.779	4.084	0.842
**H**	-0.758	4.077	-0.930
**H**	-2.314	4.042	-0.064
**O**	-4.559	2.652	0.210
**H**	-4.520	2.545	1.176

Table S96 Frequencies (cm^{-1}) of hydrogen abstraction transition state between hydroxyl radical and α-methylantracene molecule, calculated at the M06-2X/cc-pVDZ level of theory.

-1080	54	79	95	115	170	195	207
239	258	287	322	363	409	422	472
488	518	534	559	564	611	627	663
734	760	770	788	806	819	829	870
877	914	923	936	994	995	1000	1016
1036	1051	1064	1084	1145	1156	1159	1181
1198	1229	1275	1288	1304	1342	1366	1385
1404	1440	1457	1472	1478	1483	1498	1522
1618	1644	1658	1695	1711	3057	3121	3171
3190	3197	3200	3201	3213	3217	3226	3229
3790							

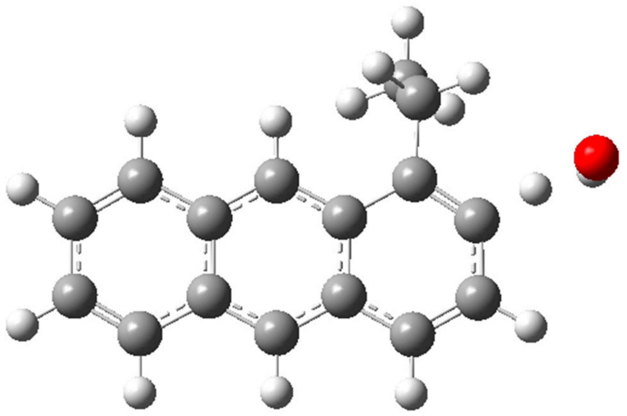

Figure S49 Visualization of hydrogen abstraction transition state between hydroxyl radical and α-ethylantracene molecule, calculated at the M06-2X/cc-pVDZ level of theory.

Table S97 Geometry (Å) of hydrogen abstraction transition state between hydroxyl radical and α-ethylantracene molecule, calculated at the M06-2X/cc-pVDZ level of theory

Atom	x	y	z

**C**	-2.420	-0.026	0.058
**C**	-1.240	-0.712	0.036
**C**	0.012	-0.016	0.014
**C**	0.003	1.418	0.016
**C**	-1.255	2.102	0.040
**C**	-2.428	1.404	0.061
**C**	1.235	-0.698	-0.010
**C**	1.222	2.103	-0.007
**C**	2.444	1.425	-0.030
**C**	2.456	-0.015	-0.032
**C**	3.727	-0.711	-0.055
**C**	4.853	0.053	-0.099
**C**	4.874	1.474	-0.085
**C**	3.684	2.143	-0.054
**H**	1.218	-1.786	-0.010
**H**	-3.366	-0.565	0.074
**H**	-1.231	-1.802	0.033
**H**	-1.256	3.192	0.041
**H**	-3.380	1.932	0.079
**H**	1.221	3.194	-0.009
**H**	3.658	3.233	-0.053
**H**	5.823	2.006	-0.111
**C**	3.803	-2.217	-0.002
**H**	3.068	-2.650	-0.693
**H**	4.795	-2.522	-0.352
**C**	3.578	-2.757	1.416
**H**	2.607	-2.441	1.817
**H**	3.619	-3.852	1.423
**H**	4.357	-2.380	2.090
**H**	5.912	-0.515	-0.146
**O**	7.022	-1.145	0.176
**H**	6.960	-1.070	1.143

Table S98 Frequencies (cm^{-1}) of hydrogen abstraction transition state between hydroxyl radical and α-ethylantracene molecule, calculated at the M06-2X/cc-pVDZ level of theory.

-1089	49	69	75	92	108	116	152
188	229	233	272	312	334	386	420
429	471	487	516	529	561	587	624
638	665	726	759	776	786	788	805
820	825	870	883	916	919	933	960
992	995	1014	1029	1036	1080	1083	1092
1147	1158	1161	1183	1199	1229	1270	1282
1291	1306	1339	1351	1369	1384	1392	1442
1458	1471	1472	1479	1490	1496	1520	1617
1641	1655	1690	1711	3064	3082	3130	3146
3151	3194	3198	3203	3203	3216	3217	3227

3228	3790

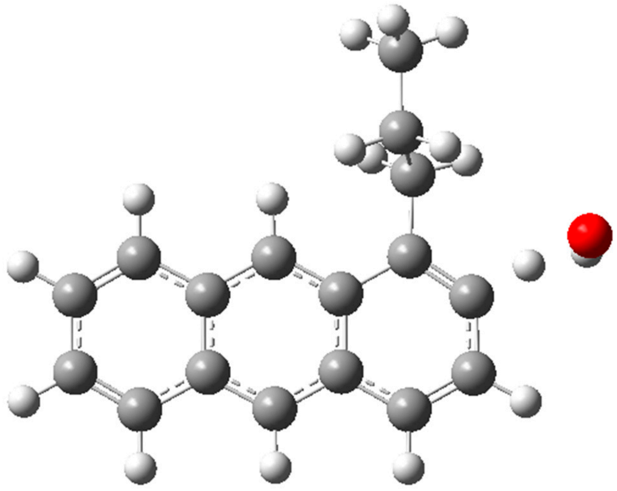

Figure S50 Visualization of hydrogen abstraction transition state between hydroxyl radical and α-propylantracene molecule, calculated at the M06-2X/cc-pVDZ level of theory.

Table S99 Geometry (Å) of hydrogen abstraction transition state between hydroxyl radical and α-propylantracene molecule, calculated at the M06-2X/cc-pVDZ level of theory

Atom	x	y	z
C	1.208	2.046	-0.069
C	2.433	1.451	-0.036
C	2.464	0.002	-0.017
C	1.223	-0.727	-0.012
C	-0.019	-0.011	-0.034
C	-0.033	1.353	-0.060
C	3.665	-0.716	0.006
C	1.245	-2.125	0.018
C	2.447	-2.839	0.042
C	3.685	-2.115	0.036
C	4.913	-2.852	0.059
H	5.853	-2.300	0.054
C	4.908	-4.218	0.087
C	3.673	-4.938	0.094
C	2.482	-4.271	0.072
H	4.617	-0.187	-0.002
H	1.171	3.245	-0.117
H	-0.950	-0.578	-0.034
H	-0.968	1.910	-0.082
H	0.301	-2.671	0.024
H	5.848	-4.768	0.105
H	3.690	-6.027	0.116
H	1.539	-4.816	0.076
C	3.702	2.263	-0.072
H	4.429	1.858	0.646

**H**	3.469	3.287	0.245
**C**	4.326	2.313	-1.473
**H**	4.479	1.293	-1.849
**H**	3.609	2.793	-2.152
**C**	5.646	3.078	-1.475
**H**	6.379	2.594	-0.817
**H**	6.076	3.127	-2.483
**H**	5.499	4.106	-1.118
**O**	1.120	4.529	0.185
**H**	1.007	4.448	1.147

Table S100 Frequencies (cm^{-1}) of hydrogen abstraction transition state between hydroxyl radical and α-propylantracene molecule, calculated at the M06-2X/cc-pVDZ level of theory.

-1043	47	50	65	76	100	111	125
139	211	226	250	270	296	303	342
380	423	426	476	489	526	547	563
603	622	641	664	737	742	761	780
788	812	817	831	865	872	898	908
920	924	935	989	996	1015	1024	1038
1072	1084	1102	1115	1147	1156	1162	1183
1193	1223	1251	1274	1288	1297	1305	1314
1346	1368	1376	1392	1395	1444	1459	1470
1476	1478	1482	1492	1496	1522	1619	1642
1656	1691	1713	3057	3063	3074	3102	3125
3136	3144	3191	3197	3200	3201	3212	3217
3226	3229	3788					

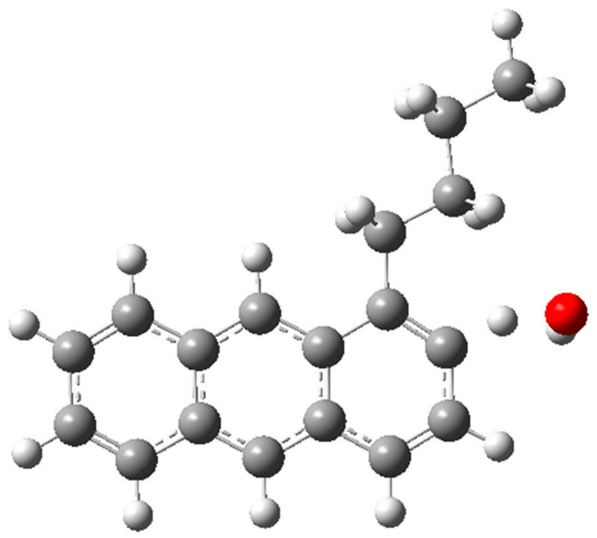

Figure S51 Visualization of hydrogen abstraction transition state between hydroxyl radical and α-butylantracene molecule, calculated at the M06-2X/cc-pVDZ level of theory.

Table S101 Geometry (Å) of hydrogen abstraction transition state between hydroxyl radical and α-butylantracene molecule, calculated at the M06-2X/cc-pVDZ level of theory

Atom	x	y	z
C	1.239	2.080	-0.025
C	2.464	1.484	-0.003
C	2.482	0.030	-0.005
C	1.241	-0.699	-0.016
C	0.001	0.020	-0.032
C	-0.002	1.383	-0.035
C	3.680	-0.692	0.006
C	1.259	-2.096	-0.013
C	2.459	-2.814	0.000
C	3.698	-2.093	0.010
C	4.924	-2.832	0.022
H	5.865	-2.281	0.029
C	4.917	-4.198	0.024
C	3.681	-4.916	0.014
C	2.492	-4.246	0.003
H	4.634	-0.168	0.013
H	1.115	3.288	-0.049
H	-0.933	-0.542	-0.045
H	-0.932	1.949	-0.057
H	0.313	-2.638	-0.022
H	5.856	-4.750	0.033
H	3.696	-6.005	0.016
H	1.547	-4.789	-0.005
C	3.775	2.233	0.004
H	4.353	1.909	0.883
H	4.356	1.907	-0.873
C	3.684	3.753	0.001
H	3.144	4.098	0.892
H	3.095	4.094	-0.862
C	5.065	4.402	-0.038
H	5.598	4.064	-0.939
H	5.654	4.055	0.823
C	4.989	5.926	-0.028
H	4.483	6.284	0.878
H	5.988	6.377	-0.061
H	4.421	6.292	-0.893
O	0.592	4.460	0.184
H	0.395	4.349	1.129

Table S102 Frequencies (cm^{-1}) of hydrogen abstraction transition state between hydroxyl radical and α-butylantracene molecule, calculated at the M06-2X/cc-pVDZ level of theory.

-1308	29	50	68	73	91	107	115
122	158	190	204	246	259	267	284

302	334	409	411	425	488	499	523
552	562	591	597	630	674	729	733
762	763	782	792	804	818	825	871
875	914	920	924	932	945	995	998
1000	1017	1036	1061	1083	1096	1116	1128
1144	1150	1159	1182	1196	1220	1234	1257
1289	1295	1307	1316	1320	1358	1367	1384
1395	1403	1418	1450	1458	1463	1470	1472
1477	1481	1486	1497	1522	1620	1644	1654
1687	1712	3036	3049	3059	3069	3071	3082
3118	3134	3141	3193	3198	3202	3203	3217
3224	3227	3229	3793				

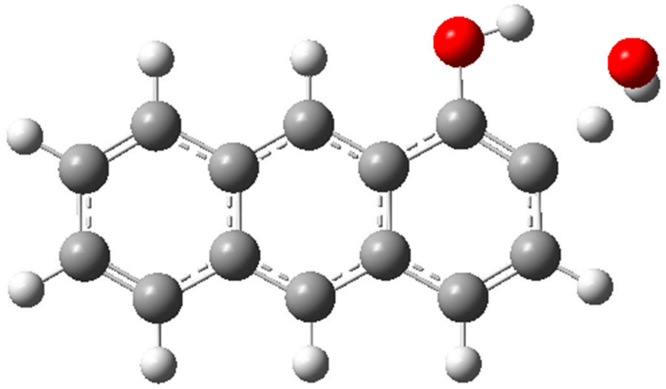

Figure S52 Visualization of hydrogen abstraction transition state between hydroxyl radical and α-hydroxyantracene molecule, calculated at the M06-2X/cc-pVDZ level of theory.

Table S103 Geometry (Å) of hydrogen abstraction transition state between hydroxyl radical and α-hydroxyantracene molecule, calculated at the M06-2X/cc-pVDZ level of theory

Atom	x	y	z
C	4.818	1.513	0.012
C	3.627	2.182	-0.011
C	2.387	1.468	-0.011
C	2.413	0.033	0.011
C	3.681	-0.631	0.036
C	4.844	0.085	0.036
C	1.154	2.132	-0.038
C	1.205	-0.677	0.010
C	-0.029	-0.023	-0.014
C	-0.042	1.414	-0.043
C	-1.318	2.084	-0.053
C	-2.459	1.331	-0.108
C	-2.463	-0.085	-0.035
C	-1.268	-0.748	-0.002
H	1.122	3.220	-0.057
H	5.757	2.065	0.013
H	3.601	3.271	-0.030

**H**	3.699	-1.721	0.054
**H**	5.803	-0.431	0.055
**H**	1.229	-1.767	0.030
**H**	-1.239	-1.837	0.012
**H**	-3.404	-0.630	-0.051
**H**	-3.481	2.004	-0.189
**O**	-1.317	3.431	-0.019
**H**	-2.244	3.724	0.030
**O**	-4.137	3.050	0.197
**H**	-4.303	2.820	1.126

Table S104 Frequencies (cm^{-1}) of hydrogen abstraction transition state between hydroxyl radical and α-hydroxyantracene molecule, calculated at the M06-2X/cc-pVDZ level of theory.

-1293	61	97	115	127	186	218	265
278	316	321	357	414	423	490	502
514	525	566	588	625	635	658	692
728	746	754	771	790	814	819	866
876	912	926	944	977	997	1017	1037
1050	1127	1144	1161	1180	1183	1220	1250
1287	1287	1318	1357	1372	1398	1447	1459
1477	1487	1518	1546	1609	1642	1651	1696
1711	3196	3199	3204	3209	3218	3223	3229
3230	3684	3796					

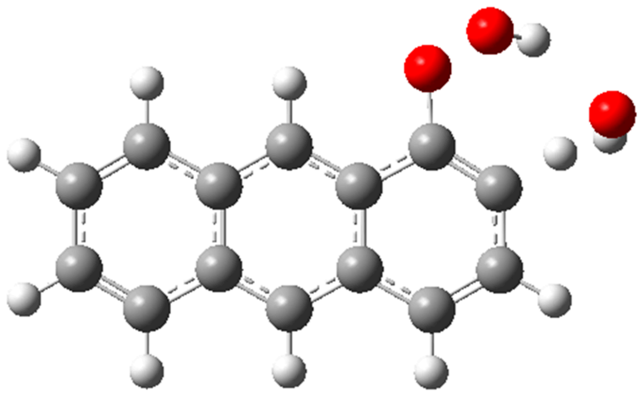

Figure S53 Visualization of hydrogen abstraction transition state between hydroxyl radical and α-peroxyantracene molecule, calculated at the M06-2X/cc-pVDZ level of theory.

Table S105 Geometry (Å) of hydrogen abstraction transition state between hydroxyl radical and α-peroxyantracene molecule, calculated at the M06-2X/cc-pVDZ level of theory

Atom	x	y	z
**C**	3.472	0.592	-0.015
**C**	3.229	-0.816	-0.024
**C**	2.431	1.476	0.008
**C**	1.950	-1.297	-0.011

**C**	0.833	-0.403	0.012
**C**	1.077	1.011	0.023
**C**	-0.008	1.895	0.055
**H**	0.179	2.969	0.070
**C**	-1.327	1.433	0.069
**C**	-0.486	-0.875	0.028
**H**	2.614	2.550	0.016
**H**	1.759	-2.370	-0.017
**H**	-0.676	-1.946	0.026
**C**	-1.561	0.016	0.052
**C**	-2.927	-0.445	0.047
**C**	-3.943	0.460	0.114
**C**	-3.721	1.863	0.121
**C**	-2.440	2.337	0.106
**H**	-4.570	2.543	0.163
**H**	-2.247	3.409	0.138
**H**	-5.101	0.049	0.061
**O**	-3.083	-1.796	-0.073
**O**	-4.274	-2.249	0.541
**H**	-4.973	-1.970	-0.082
**O**	-6.086	-0.466	-0.564
**H**	-5.960	-0.090	-1.451
**H**	4.072	-1.504	-0.042
**H**	4.498	0.956	-0.027

Table S106 Frequencies (cm^{-1}) of hydrogen abstraction transition state between hydroxyl radical and α-peroxyantracene molecule, calculated at the M06-2X/cc-pVDZ level of theory.

-1405	51	60	94	116	142	194	202
255	270	301	311	339	350	417	422
484	490	517	544	562	577	607	624
639	681	743	764	778	790	811	816
825	868	875	913	926	942	984	994
998	1018	1037	1084	1131	1148	1164	1179
1185	1227	1244	1290	1292	1345	1369	1387
1393	1447	1466	1479	1501	1508	1521	1611
1642	1653	1695	1712	3199	3202	3206	3209
3220	3227	3230	3230	3650	3795		

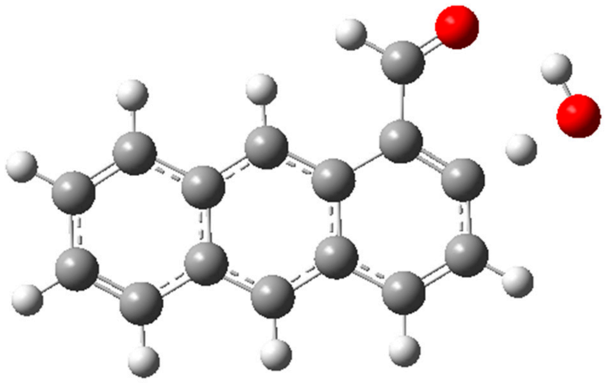

Figure S54 Visualization of hydrogen abstraction transition state between hydroxyl radical and α-antraldehyde molecule, calculated at the M06-2X/cc-pVDZ level of theory.

Table S107 Geometry (Å) of hydrogen abstraction transition state between hydroxyl radical and α-antraldehyde molecule, calculated at the M06-2X/cc-pVDZ level of theory

Atom	x	y	z
C	4.895	1.366	-0.005
C	3.729	2.077	-0.004
C	2.464	1.405	-0.002
C	2.447	-0.028	-0.002
C	3.690	-0.739	-0.003
C	4.876	-0.064	-0.005
C	1.257	2.116	-0.001
C	1.214	-0.685	0.000
C	0.008	0.022	0.001
C	0.018	1.463	0.001
C	-1.260	2.152	0.002
C	-2.406	1.405	0.004
C	-2.432	-0.008	0.004
C	-1.240	-0.679	0.003
H	1.320	3.201	-0.001
H	5.852	1.887	-0.007
H	3.743	3.166	-0.004
H	3.669	-1.829	-0.002
H	5.818	-0.610	-0.006
H	1.189	-1.776	0.001
H	-1.221	-1.769	0.003
H	-3.383	-0.536	0.006
H	-3.519	1.940	0.005
C	-1.343	3.635	0.002
O	-2.379	4.266	0.003
H	-0.385	4.186	0.001
O	-4.611	2.531	0.006
H	-4.222	3.426	0.005

Table S108 Frequencies (cm^{-1}) of hydrogen abstraction transition state between hydroxyl radical and α-antraldehyde molecule, calculated at the M06-2X/cc-pVDZ level of theory.

-1572	51	68	96	118	125	183	211
264	287	292	340	351	406	437	442
483	488	522	542	566	600	621	653
680	749	766	768	786	810	824	835
872	888	906	925	927	942	997	1020
1021	1023	1037	1043	1144	1154	1161	1185
1202	1228	1294	1294	1331	1358	1370	1401
1436	1448	1471	1485	1491	1521	1607	1638
1647	1685	1714	1807	3037	3198	3203	3204
3206	3221	3232	3232	3234	3720		

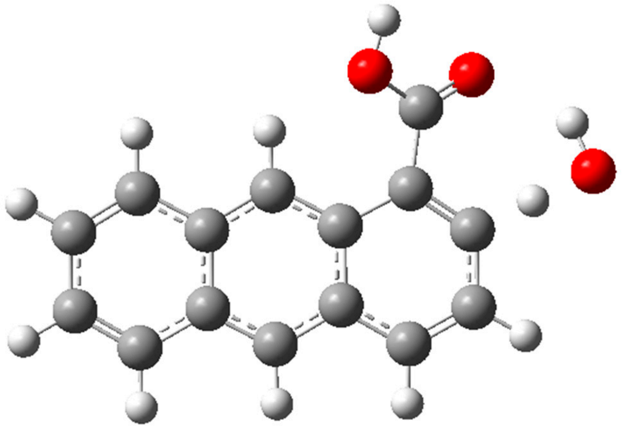

Figure S55 Visualization of hydrogen abstraction transition state between hydroxyl radical and α-antracenic acid molecule, calculated at the M06-2X/cc-pVDZ level of theory.

Table S109 Geometry (Å) of hydrogen abstraction transition state between hydroxyl radical and α-antracenic acid molecule, calculated at the M06-2X/cc-pVDZ level of theory

Atom	x	y	z
C	4.901	1.334	0.115
C	3.737	2.048	0.111
C	2.470	1.383	0.047
C	2.449	-0.048	-0.010
C	3.688	-0.765	-0.004
C	4.877	-0.095	0.056
C	1.268	2.103	0.039
C	1.213	-0.696	-0.059
C	0.010	0.017	-0.060
C	0.026	1.460	-0.025
C	-1.257	2.143	-0.016
C	-2.398	1.392	-0.011
C	-2.426	-0.022	-0.049
C	-1.235	-0.689	-0.082
H	1.322	3.186	0.080

**H**	5.858	1.849	0.163
**H**	3.753	3.137	0.155
**H**	3.663	-1.853	-0.048
**H**	5.816	-0.645	0.060
**H**	1.180	-1.786	-0.089
**H**	-1.211	-1.778	-0.117
**H**	-3.379	-0.547	-0.055
**H**	-3.513	1.917	0.002
**C**	-1.439	3.625	-0.024
**O**	-2.418	4.197	0.401
**O**	-0.430	4.313	-0.592
**H**	-0.692	5.246	-0.566
**O**	-4.631	2.448	0.146
**H**	-4.280	3.314	0.427

Table S110 Frequencies (cm^{-1}) of hydrogen abstraction transition state between hydroxyl radical and α-antracenic acid molecule, calculated at the M06-2X/cc-pVDZ level of theory.

-1573	39	52	64	109	134	164	189
250	271	276	325	343	370	399	419
436	484	497	514	556	564	583	624
628	654	678	742	749	763	787	792
802	814	833	874	887	905	929	932
943	997	997	1008	1018	1037	1133	1150
1155	1168	1186	1224	1237	1292	1297	1320
1356	1368	1393	1414	1446	1454	1483	1491
1520	1613	1638	1650	1685	1712	1820	3196
3200	3203	3204	3218	3229	3234	3270	3738
3804							

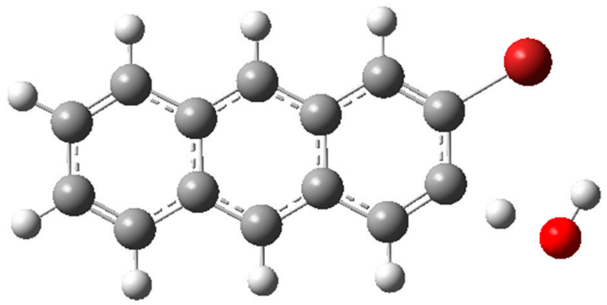

Figure S56 Visualization of hydrogen abstraction transition state between hydroxyl radical and β-bromoantracene molecule, calculated at the M06-2X/cc-pVDZ level of theory.

Table S111 Geometry (Å) of hydrogen abstraction transition state between hydroxyl radical and β-bromoantracene molecule, calculated at the M06-2X/cc-pVDZ level of theory

Atom	x	y	z
**C**	-5.905	5.975	-2.834

**C**	-4.970	1.310	-0.002
**C**	-3.661	1.697	-0.002
**C**	-2.610	0.723	0.000
**C**	-2.959	-0.670	0.000
**C**	-4.344	-1.034	0.000
**C**	-5.317	-0.077	-0.001
**C**	-1.261	1.093	0.000
**C**	-1.942	-1.631	0.001
**C**	-0.595	-1.261	0.002
**C**	-0.245	0.133	0.001
**C**	1.138	0.510	0.002
**H**	1.405	1.566	0.001
**C**	2.104	-0.452	0.003
**C**	0.458	-2.237	0.003
**H**	-0.997	2.151	0.000
**H**	-5.761	2.058	-0.003
**H**	-3.393	2.753	-0.002
**H**	-4.605	-2.092	0.000
**H**	-6.368	-0.364	-0.002
**H**	-2.206	-2.690	0.002
**H**	0.208	-3.297	0.003
**Br**	3.939	0.031	0.003
**C**	1.751	-1.828	0.003
**H**	2.624	-2.686	0.004
**O**	3.628	-3.451	0.005

Table S112 Frequencies (cm^{-1}) of hydrogen abstraction transition state between hydroxyl radical and β-bromoantracene molecule, calculated at the M06-2X/cc-pVDZ level of theory.

-1475	50	59	101	127	136	167	210
236	274	294	316	364	391	426	486
495	521	536	559	587	631	648	719
757	764	780	795	801	808	852	866
896	910	918	944	946	960	998	1021
1038	1115	1142	1151	1180	1197	1248	1284
1289	1300	1357	1366	1390	1450	1471	1477
1481	1520	1610	1640	1645	1686	1711	3197
3199	3200	3205	3211	3218	3221	3229	3767

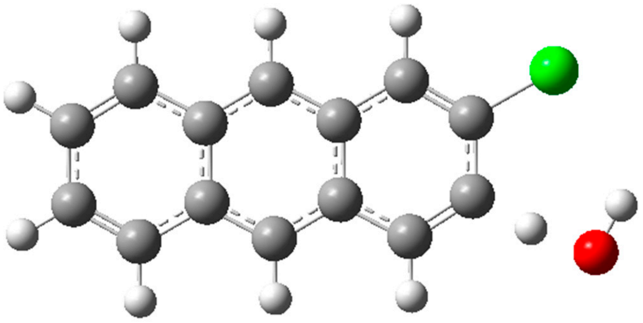

Figure S57 Visualization of hydrogen abstraction transition state between hydroxyl radical and β-chloroantracene molecule, calculated at the M06-2X/cc-pVDZ level of theory.

Table S113 Geometry (Å) of hydrogen abstraction transition state between hydroxyl radical and β-chloroantracene molecule, calculated at the M06-2X/cc-pVDZ level of theory

Atom	x	y	z
C	0.053	-0.084	-0.056
C	-1.146	-0.736	-0.024
C	-2.377	-0.003	0.001
C	-2.326	1.432	-0.007
C	-1.048	2.078	-0.041
C	0.103	1.344	-0.064
C	-3.619	-0.646	0.034
C	-3.521	2.160	0.018
C	-4.761	1.517	0.050
C	-4.813	0.081	0.059
C	-6.087	-0.576	0.092
H	-6.133	-1.664	0.099
C	-7.231	0.164	0.116
C	-7.173	1.584	0.107
C	-5.994	2.254	0.076
H	-3.656	-1.736	0.041
H	0.983	-0.651	-0.075
H	-1.188	-1.825	-0.017
H	-1.014	3.167	-0.047
H	1.070	1.845	-0.090
H	-3.484	3.250	0.011
H	-5.968	3.343	0.070
Cl	-8.793	-0.615	0.157
H	-8.214	2.231	0.128
O	-9.372	2.725	0.153
H	-9.882	1.896	0.170

Table S114 Frequencies (cm^{-1}) of hydrogen abstraction transition state between hydroxyl radical and β-chloroantracene molecule, calculated at the M06-2X/cc-pVDZ level of theory.

-1473	48	65	98	120	166	168	176

273	283	318	322	387	393	432	486
494	537	542	562	592	632	660	730
756	762	780	795	797	810	853	865
894	912	916	944	958	962	998	1019
1036	1123	1141	1151	1179	1194	1246	1283
1288	1300	1357	1366	1390	1448	1476	1478
1482	1519	1612	1642	1649	1690	1713	3198
3199	3201	3205	3213	3219	3220	3230	3776

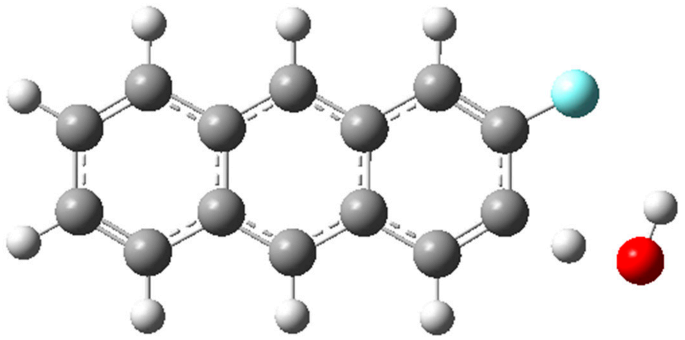

Figure S58 Visualization of hydrogen abstraction transition state between hydroxyl radical and β-fluoroantracene molecule, calculated at the M06-2X/cc-pVDZ level of theory.

Table S115 Geometry (Å) of hydrogen abstraction transition state between hydroxyl radical and β-fluoroantracene molecule, calculated at the M06-2X/cc-pVDZ level of theory

Atom	x	y	z
C	0.046	-0.105	-0.043
C	-1.160	-0.744	-0.001
C	-2.383	0.002	0.014
C	-2.318	1.436	-0.017
C	-1.033	2.068	-0.061
C	0.111	1.323	-0.074
C	-3.632	-0.629	0.057
C	-3.505	2.174	-0.003
C	-4.752	1.544	0.040
C	-4.819	0.108	0.072
C	-6.100	-0.536	0.116
H	-6.176	-1.622	0.140
C	-7.220	0.230	0.128
C	-7.160	1.645	0.097
C	-5.975	2.301	0.055
H	-3.678	-1.718	0.081
H	0.970	-0.681	-0.054
H	-1.212	-1.832	0.023
H	-0.988	3.157	-0.085
H	1.083	1.813	-0.108
H	-3.458	3.264	-0.027

H	-5.930	3.389	0.032
H	-8.232	2.255	0.111
F	-8.446	-0.337	0.169
O	-9.444	2.563	0.135
H	-9.826	1.668	0.163

Table S116 Frequencies (cm^{-1}) of hydrogen abstraction transition state between hydroxyl radical and β-fluoroantracene molecule, calculated at the M06-2X/cc-pVDZ level of theory.

-1510	50	79	80	128	194	204	219
277	319	335	346	397	429	463	486
493	542	555	582	607	633	688	754
762	776	787	794	813	815	864	864
888	913	914	941	955	996	1017	1022
1036	1139	1149	1173	1185	1219	1247	1283
1301	1309	1363	1374	1410	1451	1481	1485
1510	1530	1622	1648	1662	1706	1714	3198
3199	3201	3205	3214	3219	3227	3230	3778

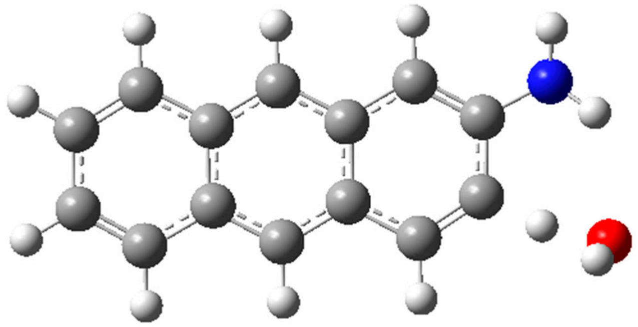

Figure S59 Visualization of hydrogen abstraction transition state between hydroxyl radical and β-aminoantracene molecule, calculated at the M06-2X/cc-pVDZ level of theory.

Table S117 Geometry (Å) of hydrogen abstraction transition state between hydroxyl radical and β-aminoantracene molecule, calculated at the M06-2X/cc-pVDZ level of theory

Atom	x	y	z
C	0.377	1.427	0.006
C	1.611	0.767	-0.033
C	1.617	-0.671	-0.077
C	0.413	-1.377	-0.082
C	-0.818	-0.712	-0.042
H	0.356	2.517	0.038
C	2.849	1.481	-0.027
C	2.887	-1.344	-0.107
H	0.432	-2.467	-0.116
C	4.023	-0.610	-0.125

**C**	4.055	0.822	-0.070
**H**	2.915	-2.433	-0.124
**H**	5.108	-1.145	-0.175
**H**	2.827	2.571	0.003
**N**	5.284	1.452	-0.128
**H**	5.321	2.411	0.185
**H**	6.082	0.884	0.129
**O**	6.312	-1.443	0.234
**H**	6.093	-1.759	1.127
**C**	-0.833	0.723	0.002
**C**	-2.099	1.392	0.041
**C**	-3.267	0.684	0.036
**H**	-2.111	2.482	0.074
**C**	-2.063	-1.419	-0.046
**C**	-3.250	-0.745	-0.009
**H**	-2.044	-2.508	-0.081
**H**	-4.222	1.206	0.065
**H**	-4.192	-1.291	-0.012

Table S118 Frequencies (cm^{-1}) of hydrogen abstraction transition state between hydroxyl radical and β-aminoantracene molecule, calculated at the M06-2X/cc-pVDZ level of theory.

-1195	55	80	99	135	188	208	236
249	325	332	340	390	404	435	451
481	488	506	526	561	589	628	646
688	748	760	772	789	793	803	849
864	871	892	911	928	942	961	992
1014	1036	1107	1144	1152	1176	1190	1199
1250	1265	1284	1300	1326	1353	1368	1388
1450	1479	1492	1512	1526	1614	1631	1642
1664	1699	1712	3192	3192	3194	3197	3201
3206	3216	3228	3585	3714	3789		

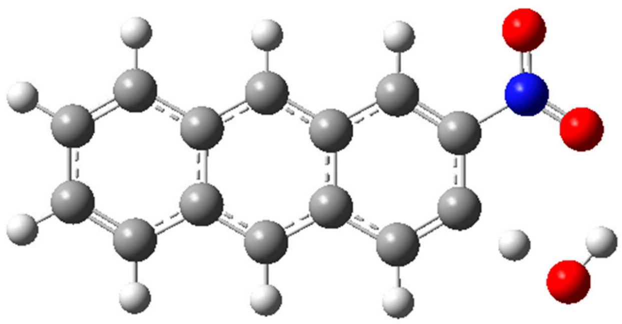

Figure S60 Visualization of hydrogen abstraction transition state between hydroxyl radical and β-nitroantracene molecule, calculated at the M06-2X/cc-pVDZ level of theory.

Table S119 Geometry (Å) of hydrogen abstraction transition state between hydroxyl radical and β-nitroantracene molecule, calculated at the M06-2X/cc-pVDZ level of theory

Atom	x	y	z
C	4.451	-0.838	0.002
C	3.248	-1.484	0.002
C	2.022	-0.745	0.002
C	2.082	0.691	0.001
C	3.363	1.331	0.001
C	4.510	0.591	0.002
C	0.776	-1.384	0.002
C	0.892	1.425	0.000
C	-0.352	0.785	0.000
C	-0.412	-0.651	0.001
C	-1.699	-1.291	0.001
H	-1.757	-2.378	0.002
C	-2.829	-0.541	0.001
C	-2.765	0.878	0.000
C	-1.568	1.534	-0.001
H	0.734	-2.473	0.002
H	5.378	-1.409	0.003
H	3.201	-2.572	0.003
H	3.403	2.420	0.000
H	0.933	2.515	0.000
H	-3.895	-1.172	0.002
H	-1.550	2.622	-0.002
O	-4.949	-1.807	0.002
H	-5.534	-1.029	0.002
H	5.480	1.085	0.002
N	-4.001	1.677	-0.001
O	-5.063	1.079	0.002
O	-3.896	2.886	-0.005

Table S120 Frequencies (cm^{-1}) of hydrogen abstraction transition state between hydroxyl radical and β-nitroantracene molecule, calculated at the M06-2X/cc-pVDZ level of theory.

-1618	42	51	62	114	128	169	174
254	264	300	310	321	363	397	418
486	497	497	526	547	583	586	632
654	722	755	757	767	773	784	795
817	846	871	874	907	914	925	957
966	998	1006	1020	1036	1133	1147	1152
1180	1195	1247	1283	1297	1302	1359	1370
1393	1451	1463	1476	1481	1484	1524	1617
1642	1649	1682	1711	1714	3200	3201	3202
3206	3212	3220	3231	3236	3780		

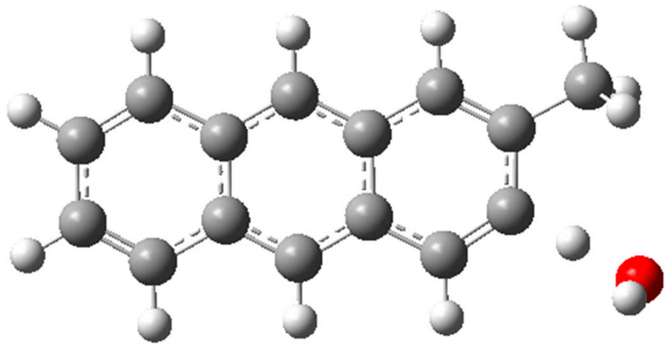

Figure S61 Visualization of hydrogen abstraction transition state between hydroxyl radical and β-methylantracene molecule, calculated at the M06-2X/cc-pVDZ level of theory.

Table S121 Geometry (Å) of hydrogen abstraction transition state between hydroxyl radical and β-methylantracene molecule, calculated at the M06-2X/cc-pVDZ level of theory

Atom	x	y	z
C	4.464	-0.840	-0.019
C	3.263	-1.488	0.019
C	2.034	-0.753	0.018
C	2.085	0.681	-0.024
C	3.365	1.323	-0.063
C	4.516	0.588	-0.060
C	0.789	-1.392	0.056
C	0.891	1.412	-0.025
C	-0.353	0.776	0.014
C	-0.401	-0.660	0.054
C	-1.688	-1.298	0.090
H	-1.747	-2.385	0.117
C	-2.805	-0.529	0.100
C	-2.801	0.900	0.049
C	-1.581	1.518	0.012
H	0.747	-2.481	0.089
H	5.393	-1.409	-0.018
H	3.219	-2.577	0.051
H	3.401	2.411	-0.094
H	5.484	1.086	-0.090
H	0.933	2.501	-0.055
H	-3.877	-1.070	0.158
H	-1.526	2.607	-0.018
C	-4.104	1.650	0.052
H	-4.692	1.397	0.943
H	-4.710	1.373	-0.820
H	-3.933	2.731	0.035
O	-5.023	-1.641	-0.138
H	-4.867	-1.800	-1.085

Table S122 Frequencies (cm^{-1}) of hydrogen abstraction transition state between hydroxyl radical and β-methylantracene molecule, calculated at the M06-2X/cc-pVDZ level of theory.

-1100	50	67	75	94	134	147	191
194	254	310	326	346	391	430	442
487	497	518	557	586	619	634	671
758	762	767	785	794	814	861	867
878	896	910	936	943	972	993	1015
1024	1037	1056	1098	1143	1152	1177	1191
1204	1264	1268	1284	1312	1320	1365	1376
1400	1441	1460	1463	1480	1488	1492	1519
1612	1642	1660	1705	1712	3065	3132	3161
3188	3193	3195	3198	3202	3206	3217	3229
3788							

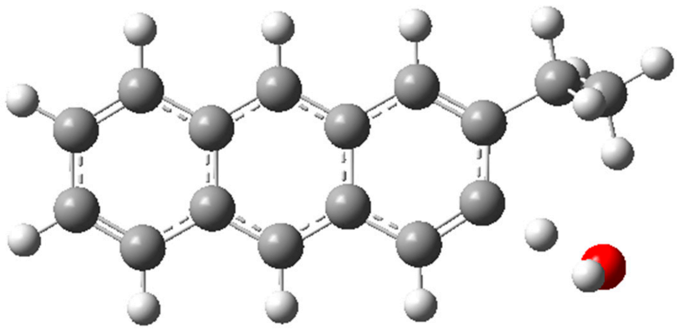

Figure S62 Visualization of hydrogen abstraction transition state between hydroxyl radical and β-ethylantracene molecule, calculated at the M06-2X/cc-pVDZ level of theory.

Table S123 Geometry (Å) of hydrogen abstraction transition state between hydroxyl radical and β-ethylantracene molecule, calculated at the M06-2X/cc-pVDZ level of theory

Atom	x	y	z
C	-3.632	-6.231	-0.119
C	-3.609	-4.865	-0.102
C	-2.368	-4.153	-0.040
C	-1.144	-4.903	0.005
C	-1.210	-6.333	-0.014
C	-2.413	-6.977	-0.074
C	-2.318	-2.754	-0.022
C	0.075	-4.218	0.066
C	0.122	-2.822	0.084
C	-1.101	-2.069	0.039
C	-1.041	-0.636	0.059
H	-1.978	-0.078	0.026
C	0.148	0.040	0.118
C	1.341	-0.748	0.149
C	1.364	-2.103	0.152

**H**	-3.249	-2.187	-0.056
**H**	-4.581	-6.762	-0.166
**H**	-4.537	-4.294	-0.136
**H**	-0.279	-6.898	0.019
**H**	-2.452	-8.065	-0.089
**H**	1.006	-4.786	0.099
**H**	2.302	-2.656	0.197
**H**	2.384	-0.153	0.184
**C**	0.244	1.543	0.105
**H**	-0.760	1.966	0.230
**H**	0.852	1.870	0.959
**C**	0.880	2.064	-1.188
**H**	0.921	3.159	-1.183
**H**	1.904	1.685	-1.293
**H**	0.298	1.740	-2.060
**O**	3.456	0.501	0.584
**H**	3.438	0.253	1.524

Table S124 Frequencies (cm^{-1}) of hydrogen abstraction transition state between hydroxyl radical and β-ethylantracene molecule, calculated at the M06-2X/cc-pVDZ level of theory.

-1080	50	64	75	81	121	128	152
180	235	243	260	318	336	375	413
440	444	488	497	537	558	584	630
654	688	757	761	764	778	789	794
814	865	868	885	899	910	941	945
959	996	996	1016	1036	1079	1092	1112
1144	1153	1178	1192	1204	1253	1270	1282
1286	1311	1324	1338	1368	1380	1388	1447
1461	1474	1476	1478	1486	1494	1519	1612
1642	1660	1704	1712	3064	3069	3118	3147
3153	3188	3194	3196	3198	3203	3208	3217
3228	3785						

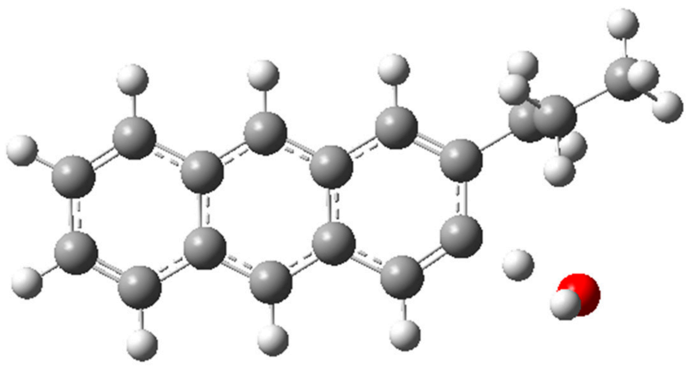

Figure S63 Visualization of hydrogen abstraction transition state between hydroxyl radical and β-propylantracene molecule, calculated at the M06-2X/cc-pVDZ level of theory.

Table S125 Geometry (Å) of hydrogen abstraction transition state between hydroxyl radical and β-propylantracene molecule, calculated at the M06-2X/cc-pVDZ level of theory

Atom	x	y	z
C	-1.235	2.153	-0.039
C	-2.415	1.459	-0.020
C	-2.454	0.026	-0.021
C	-1.219	-0.709	-0.046
C	0.013	0.029	-0.067
C	-0.030	1.385	-0.073
C	-3.661	-0.678	0.001
C	-1.245	-2.105	-0.050
C	-2.455	-2.808	-0.027
C	-3.691	-2.077	-0.001
C	-4.923	-2.808	0.022
H	-5.859	-2.251	0.042
C	-4.925	-4.174	0.019
C	-3.695	-4.901	-0.007
C	-2.501	-4.240	-0.030
H	-4.602	-0.125	0.019
H	-3.361	2.003	-0.005
H	0.960	-0.509	-0.092
H	1.002	1.996	-0.133
H	-0.306	-2.659	-0.072
H	-5.868	-4.719	0.037
H	-3.718	-5.990	-0.009
H	-1.560	-4.791	-0.050
C	-1.168	3.656	0.011
H	-0.384	4.008	-0.674
H	-2.124	4.076	-0.330
C	-0.860	4.171	1.421
H	0.074	3.711	1.770
H	-1.654	3.837	2.102
C	-0.731	5.690	1.456
H	-0.524	6.050	2.471
H	0.086	6.022	0.803
H	-1.657	6.168	1.109
O	2.106	2.666	0.135
H	2.202	2.415	1.070

Table S126 Frequencies (cm^{-1}) of hydrogen abstraction transition state between hydroxyl radical and β-propylantracene molecule, calculated at the M06-2X/cc-pVDZ level of theory.

-1079	43	50	58	62	96	120	138
168	198	224	248	260	270	321	352
374	395	431	484	493	509	525	560
606	624	642	704	729	758	763	774
787	794	818	859	863	875	886	898

910	913	941	945	982	994	1015	1037
1070	1092	1105	1116	1144	1151	1177	1190
1203	1239	1252	1267	1283	1290	1309	1311
1324	1366	1372	1380	1393	1448	1461	1468
1476	1478	1482	1484	1491	1518	1612	1642
1658	1702	1711	3059	3060	3070	3099	3119
3136	3144	3185	3192	3194	3197	3201	3206
3216	3228	3788					

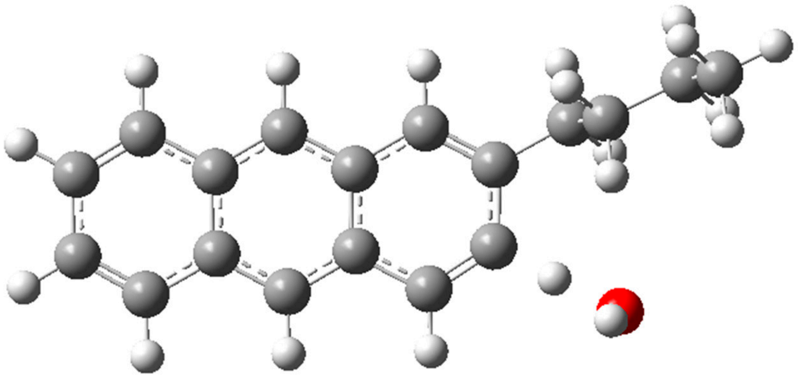

Figure S64 Visualization of hydrogen abstraction transition state between hydroxyl radical and β-butylantracene molecule, calculated at the M06-2X/cc-pVDZ level of theory.

Table S127 Geometry (Å) of hydrogen abstraction transition state between hydroxyl radical and β-butylantracene molecule, calculated at the M06-2X/cc-pVDZ level of theory

Atom	x	y	z
C	-1.240	2.155	-0.038
C	-2.420	1.460	-0.015
C	-2.457	0.026	-0.018
C	-1.222	-0.707	-0.048
C	0.010	0.031	-0.074
C	-0.035	1.387	-0.078
C	-3.665	-0.679	0.006
C	-1.247	-2.104	-0.053
C	-2.457	-2.808	-0.026
C	-3.693	-2.078	0.003
C	-4.924	-2.811	0.029
H	-5.861	-2.254	0.052
C	-4.925	-4.176	0.026
C	-3.694	-4.902	-0.004
C	-2.500	-4.240	-0.030
H	-4.605	-0.127	0.028
H	-3.366	2.003	0.004
H	0.957	-0.507	-0.103
H	0.997	1.998	-0.141
H	-0.307	-2.657	-0.078

**H**	-5.867	-4.722	0.046
**H**	-3.716	-5.991	-0.007
**H**	-1.560	-4.790	-0.053
**C**	-1.172	3.657	0.014
**H**	-0.398	4.010	-0.681
**H**	-2.133	4.078	-0.312
**C**	-0.843	4.168	1.421
**H**	0.093	3.703	1.760
**H**	-1.631	3.842	2.115
**C**	-0.700	5.686	1.468
**H**	0.098	5.988	0.775
**H**	-1.628	6.148	1.101
**C**	-0.388	6.199	2.871
**H**	0.551	5.767	3.242
**H**	-0.288	7.291	2.886
**H**	-1.186	5.922	3.573
**O**	2.105	2.663	0.123
**H**	2.207	2.404	1.055

Table S128 Frequencies (cm^{-1}) of hydrogen abstraction transition state between hydroxyl radical and β-butylantracene molecule, calculated at the M06-2X/cc-pVDZ level of theory.

-1074	29	41	48	55	82	99	124
136	172	195	199	237	241	257	308
327	370	388	418	432	484	493	507
530	559	606	626	642	703	716	758
763	774	779	791	793	819	863	869
881	896	910	915	926	941	945	981
995	1014	1036	1043	1088	1096	1108	1128
1144	1152	1177	1190	1204	1222	1253	1256
1266	1284	1295	1309	1314	1324	1343	1367
1378	1388	1394	1449	1457	1466	1473	1476
1477	1484	1484	1491	1518	1611	1642	1659
1702	1711	3046	3054	3057	3066	3080	3098
3119	3131	3140	3185	3193	3195	3198	3202
3207	3216	3228	3788				

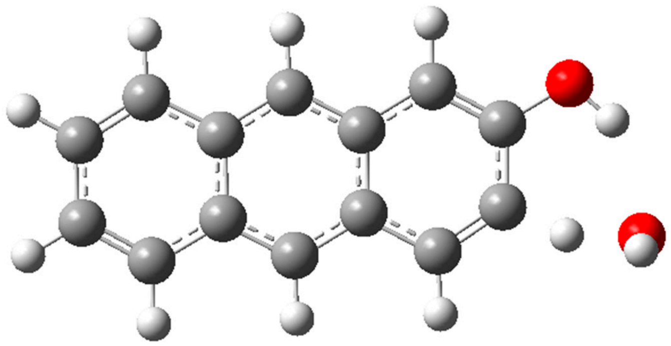

Figure S65 Visualization of hydrogen abstraction transition state between hydroxyl radical and β-hydroxyantracene molecule, calculated at the M06-2X/cc-pVDZ level of theory.

Table S129 Geometry (Å) of hydrogen abstraction transition state between hydroxyl radical and β-hydroxyantracene molecule, calculated at the M06-2X/cc-pVDZ level of theory

Atom	x	y	z
C	0.048	-0.071	0.040
C	-1.149	-0.728	0.030
C	-2.385	-0.003	0.004
C	-2.338	1.432	-0.011
C	-1.063	2.085	0.000
C	0.093	1.358	0.025
C	-3.623	-0.654	-0.007
C	-3.538	2.151	-0.038
C	-4.773	1.498	-0.048
C	-4.827	0.060	-0.033
C	-6.093	-0.605	-0.041
H	-6.140	-1.693	-0.028
C	-7.256	0.115	-0.066
C	-7.175	1.540	-0.099
C	-6.009	2.230	-0.070
H	-3.651	-1.744	0.004
H	0.980	-0.634	0.060
H	-1.184	-1.817	0.042
H	-1.035	3.174	-0.012
H	1.057	1.863	0.033
H	-3.509	3.241	-0.051
H	-5.988	3.319	-0.076
H	-8.250	2.096	-0.169
O	-8.460	-0.504	-0.072
H	-9.155	0.173	-0.063
O	-9.528	2.200	0.106
H	-9.461	2.429	1.049

Table S130 Frequencies (cm-1) of hydrogen abstraction transition state between hydroxyl radical and β-hydroxyantracene molecule, calculated at the M06-2X/cc-pVDZ level of theory.

-1192	55	84	114	136	194	215	254
293	327	339	344	393	433	474	483
490	526	559	588	612	627	638	690
745	761	761	782	793	796	858	860
881	894	912	936	941	970	993	1013
1036	1138	1149	1170	1176	1187	1236	1245
1271	1284	1306	1346	1368	1377	1434	1453
1483	1495	1521	1534	1621	1646	1664	1704
1714	3194	3198	3199	3203	3211	3218	3219
3229	3747	3779					

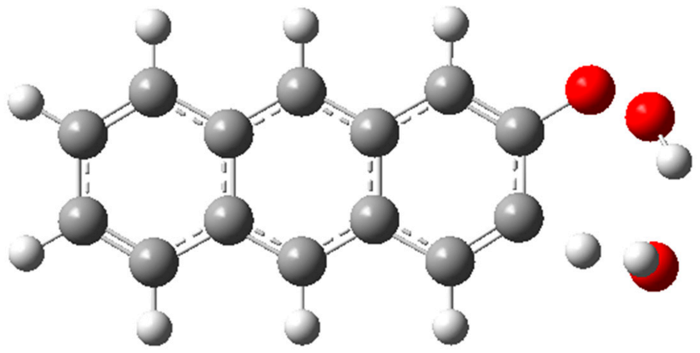

Figure S66 Visualization of hydrogen abstraction transition state between hydroxyl radical and β-peroxyantracene molecule, calculated at the M06-2X/cc-pVDZ level of theory.

Table S131 Geometry (Å) of hydrogen abstraction transition state between hydroxyl radical and β-peroxyantracene molecule, calculated at the M06-2X/cc-pVDZ level of theory

Atom	x	y	z
C	0.035	-0.042	0.039
C	-1.152	-0.717	0.053
C	-2.397	-0.009	0.020
C	-2.373	1.425	-0.028
C	-1.108	2.096	-0.041
C	0.058	1.386	-0.009
C	-3.628	-0.677	0.035
C	-3.584	2.128	-0.062
C	-4.809	1.460	-0.048
C	-4.836	0.023	0.003
C	-6.098	-0.658	0.003
H	-6.138	-1.746	0.021
C	-7.261	0.052	-0.021
C	-7.216	1.477	-0.067
C	-6.057	2.176	-0.069
H	-3.643	-1.767	0.071

**H**	0.975	-0.591	0.064
**H**	-1.173	-1.806	0.089
**H**	-1.095	3.185	-0.078
**H**	1.015	1.905	-0.020
**H**	-3.568	3.218	-0.100
**H**	-6.049	3.266	-0.078
**H**	-8.284	2.048	-0.076
**O**	-8.462	-0.606	0.086
**O**	-9.316	-0.194	-0.983
**H**	-9.761	0.580	-0.590
**O**	-9.506	2.353	0.268
**H**	-9.424	2.269	1.234

Table S132 Frequencies (cm^{-1}) of hydrogen abstraction transition state between hydroxyl radical and β-peroxyantracene molecule, calculated at the M06-2X/cc-pVDZ level of theory.

-1217	47	69	78	130	154	189	198
248	269	286	324	338	374	415	434
477	488	497	541	560	575	588	632
682	694	749	762	777	790	795	807
859	866	887	902	911	940	944	967
995	1010	1016	1038	1112	1142	1151	1176
1185	1220	1255	1281	1289	1311	1351	1366
1377	1450	1478	1484	1489	1500	1522	1618
1646	1658	1699	1713	3196	3199	3200	3204
3211	3218	3221	3230	3679	3781		

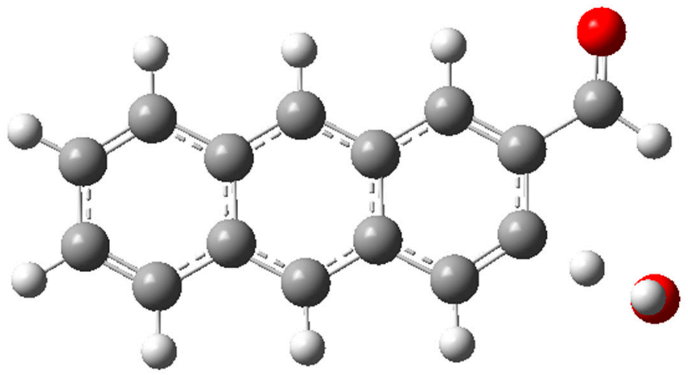

Figure S67 Visualization of hydrogen abstraction transition state between hydroxyl radical and β-antraldehyde molecule, calculated at the M06-2X/cc-pVDZ level of theory.

Table S133 Geometry (Å) of hydrogen abstraction transition state between hydroxyl radical and β-antraldehyde molecule, calculated at the M06-2X/cc-pVDZ level of theory

Atom	x	y	z
**C**	4.454	-0.833	-0.017
**C**	3.254	-1.483	0.020

**C**	2.025	-0.749	0.018
**C**	2.078	0.687	-0.023
**C**	3.358	1.331	-0.061
**C**	4.508	0.595	-0.058
**C**	0.782	-1.391	0.056
**C**	0.886	1.416	-0.024
**C**	-0.359	0.776	0.013
**C**	-0.410	-0.662	0.054
**C**	-1.694	-1.309	0.087
**H**	-1.745	-2.397	0.111
**C**	-2.814	-0.544	0.096
**C**	-2.793	0.879	0.048
**C**	-1.579	1.517	0.010
**H**	0.744	-2.480	0.088
**H**	5.384	-1.401	-0.015
**H**	3.212	-2.571	0.051
**H**	3.393	2.419	-0.092
**H**	0.923	2.506	-0.055
**H**	-3.896	-1.094	0.150
**H**	-1.559	2.607	-0.018
**O**	-5.034	-1.642	-0.124
**H**	-4.919	-1.772	-1.082
**H**	5.476	1.093	-0.087
**C**	-4.061	1.659	0.054

Table S134 Frequencies (cm^{-1}) of hydrogen abstraction transition state between hydroxyl radical and β-antraldehyde molecule, calculated at the M06-2X/cc-pVDZ level of theory.

-1100	50	67	75	94	134	147	191
194	254	310	326	346	391	430	442
487	497	518	557	586	619	634	671
758	762	767	785	794	814	861	867
878	896	910	936	943	972	993	1015
1024	1037	1056	1098	1143	1152	1177	1191
1204	1264	1268	1284	1312	1320	1365	1376
1400	1441	1460	1463	1480	1488	1492	1519
1612	1642	1660	1705	1712	3065	3132	3161
3188	3193	3195	3198	3202	3206	3217	3229

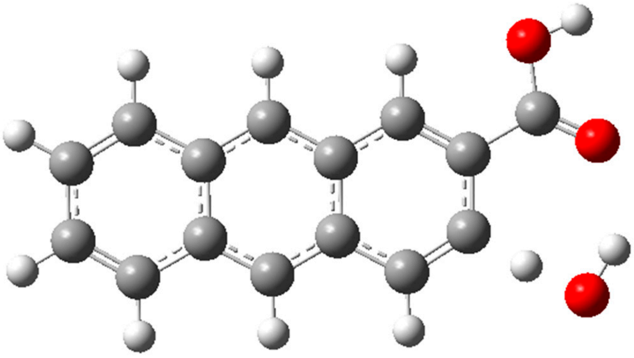

Figure S68 Visualization of hydrogen abstraction transition state between hydroxyl radical and β-antracenic acid molecule, calculated at the M06-2X/cc-pVDZ level of theory.

Table S135 Geometry (Å) of hydrogen abstraction transition state between hydroxyl radical and β-antracenic acid molecule, calculated at the M06-2X/cc-pVDZ level of theory

Atom	x	y	z
C	0.056	-0.072	-0.048
C	-1.137	-0.736	-0.054
C	-2.375	-0.017	-0.022
C	-2.343	1.419	0.017
C	-1.071	2.078	0.022
C	0.088	1.357	-0.009
C	-3.610	-0.672	-0.027
C	-3.545	2.136	0.049
C	-4.778	1.480	0.043
C	-4.811	0.044	0.004
C	-6.075	-0.624	-0.001
H	-6.098	-1.712	-0.031
C	-7.249	0.085	0.030
C	-7.192	1.511	0.068
C	-6.024	2.197	0.075
H	-3.638	-1.762	-0.057
H	0.992	-0.626	-0.073
H	-1.167	-1.825	-0.084
H	-1.048	3.167	0.051
H	1.049	1.869	-0.005
H	-3.518	3.225	0.078
H	-6.013	3.286	0.105
H	-8.225	2.190	0.097
C	-8.564	-0.607	0.025
O	-9.642	-0.057	0.051
O	-8.457	-1.949	-0.013
H	-9.360	-2.298	-0.014
O	-9.318	2.778	0.124
H	-9.858	1.965	0.108

Table S136 Frequencies (cm^{-1}) of hydrogen abstraction transition state between hydroxyl radical and β-antracenic acid molecule, calculated at the M06-2X/cc-pVDZ level of theory.

-1569	49	61	65	112	132	166	175
252	265	302	304	354	390	411	437
482	487	497	524	542	566	576	624
631	642	694	761	763	768	781	784
796	813	834	857	871	909	913	928
960	967	997	998	1019	1036	1135	1142
1151	1179	1196	1220	1256	1284	1297	1304
1360	1367	1395	1410	1453	1476	1482	1496
1522	1610	1640	1654	1693	1710	1830	3196
3199	3200	3204	3210	3218	3224	3230	3726
3824							

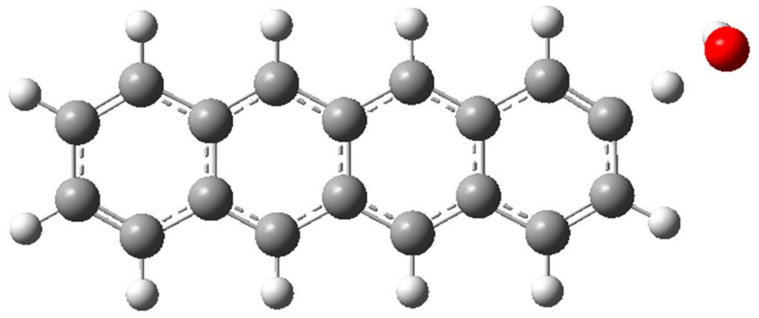

Figure S69 Visualization of hydrogen abstraction transition state between hydroxyl radical and tetracene molecule, calculated at the M06-2X/cc-pVDZ level of theory.

Table S137 Geometry (Å) of hydrogen abstraction transition state between hydroxyl radical and tetracene molecule, calculated at the M06-2X/cc-pVDZ level of theory

Atom	x	y	z
C	-3.651	0.744	-0.004
C	-2.472	1.425	0.000
C	-1.216	0.725	0.000
C	-1.229	-0.720	-0.004
C	-2.499	-1.396	-0.009
C	-3.664	-0.692	-0.009
C	-0.001	1.397	0.005
C	-0.027	-1.415	-0.004
C	1.213	-0.742	0.001
C	1.226	0.701	0.005
C	2.466	1.374	0.009
H	2.476	2.465	0.012
C	3.669	0.680	0.009
C	2.440	-1.439	0.000
H	0.009	2.488	0.008
H	-4.596	1.285	-0.005

**H**	-2.458	2.515	0.003
**H**	-2.506	-2.485	-0.013
**H**	-4.620	-1.215	-0.013
**H**	-0.038	-2.506	-0.008
**H**	2.431	-2.530	-0.005
**C**	3.653	-0.765	0.005
**C**	4.913	-1.467	0.009
**H**	4.917	-2.557	0.013
**C**	6.110	0.672	0.012
**C**	4.935	1.364	0.015
**H**	7.069	1.186	0.011
**H**	4.937	2.454	0.017
**C**	6.065	-0.756	-0.001
**H**	7.111	-1.354	-0.026
**O**	8.199	-1.994	0.302
**H**	8.077	-2.014	1.267

Table S138 Frequencies (cm^{-1}) of hydrogen abstraction transition state between hydroxyl radical and tetracene molecule, calculated at the M06-2X/cc-pVDZ level of theory.

-1170	41	50	71	101	135	160	163
198	273	298	304	327	385	422	447
476	485	499	503	536	560	587	621
636	638	740	758	762	771	775	777
788	808	828	858	874	883	900	905
922	934	944	948	996	1003	1014	1026
1035	1099	1119	1153	1154	1173	1186	1212
1221	1276	1279	1285	1320	1324	1340	1347
1389	1440	1452	1470	1488	1490	1502	1589
1619	1626	1638	1683	1696	1717	3193	3194
3195	3196	3198	3202	3202	3209	3217	3229
3230	3792						

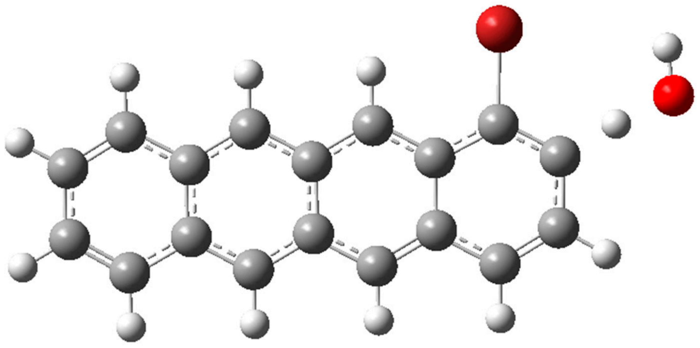

Figure S70 Visualization of hydrogen abstraction transition state between hydroxyl radical and α-bromotetracene molecule, calculated at the M06-2X/cc-pVDZ level of theory.

Table S139 Geometry (Å) of hydrogen abstraction transition state between hydroxyl radical and α-bromotetracene molecule, calculated at the M06-2X/cc-pVDZ level of theory

Atom	x	y	z
C	-3.112	-1.675	-0.001
C	-2.217	-0.597	-0.001
C	-2.734	0.748	-0.001
C	-4.115	0.952	-0.002
C	-5.011	-0.128	-0.002
C	-0.815	-0.801	0.000
C	-1.810	1.828	-0.001
C	-0.443	1.621	-0.001
C	0.079	0.266	0.000
C	1.503	0.130	0.001
C	2.397	1.193	0.001
C	1.827	2.503	0.000
C	0.474	2.721	0.000
H	-0.434	-1.821	0.000
H	-2.721	-2.693	0.000
H	-4.507	1.970	-0.002
H	-2.194	2.849	-0.002
H	0.066	3.732	-0.001
H	2.509	3.354	0.000
H	3.682	1.020	0.001
Br	2.254	-1.622	0.001
O	4.957	0.934	0.002
H	5.211	0.002	0.002
C	-4.498	-1.470	-0.001
C	-5.419	-2.562	-0.001
C	-6.768	-2.335	-0.002
H	-5.026	-3.578	-0.001
C	-7.277	-1.003	-0.003
H	-7.464	-3.173	-0.002
C	-6.426	0.068	-0.003
H	-8.354	-0.843	-0.003
H	-6.813	1.087	-0.003

Table S140 Frequencies (cm^{-1}) of hydrogen abstraction transition state between hydroxyl radical and α-bromotetracene molecule, calculated at the M06-2X/cc-pVDZ level of theory.

-4465	30	50	53	70	105	149	162
197	224	235	268	287	320	335	346
397	403	469	473	493	510	518	533
548	580	586	632	641	676	748	766
782	789	791	796	818	856	872	901
910	911	924	953	960	963	1000	1024
1033	1041	1119	1153	1156	1158	1184	1195
1226	1250	1286	1294	1303	1323	1328	1367

1383	1433	1440	1450	1461	1470	1506	1520
1557	1587	1617	1629	1637	1658	1701	3193
3199	3200	3203	3206	3209	3210	3224	3227
3234	3857						

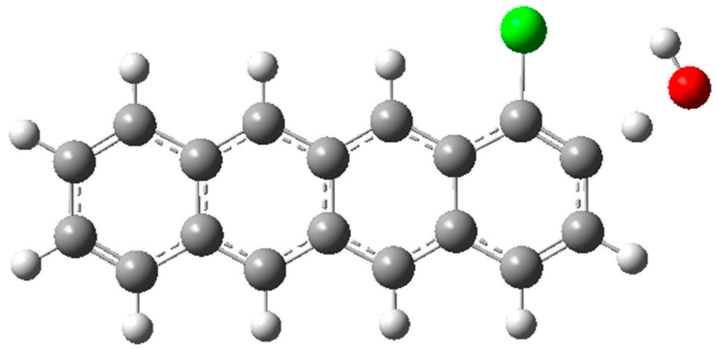

Figure S71 Visualization of hydrogen abstraction transition state between hydroxyl radical and α-chlorotetracene acid molecule, calculated at the M06-2X/cc-pVDZ level of theory.

Table S141 Geometry (Å) of hydrogen abstraction transition state between hydroxyl radical and α-chlorotetracene molecule, calculated at the M06-2X/cc-pVDZ level of theory

Atom	x	y	z
C	-2.921	-1.615	-0.001
C	-1.901	-0.642	-0.001
C	-2.265	0.753	-0.001
C	-3.630	1.107	-0.002
C	-4.627	0.141	-0.002
C	-0.535	-1.000	0.000
C	-1.241	1.723	-0.001
C	0.101	1.368	-0.001
C	0.460	-0.035	0.000
C	1.872	-0.346	0.001
C	2.798	0.639	0.001
C	2.450	2.023	0.000
C	1.133	2.370	-0.001
H	-0.269	-2.055	0.001
H	-2.647	-2.671	0.000
H	-3.904	2.163	-0.002
H	-1.511	2.779	-0.002
H	0.838	3.418	-0.001
H	3.236	2.774	0.000
H	3.989	0.361	0.001
Cl	2.379	-2.019	0.002
O	5.163	-0.102	0.002
H	4.938	-1.050	0.002
C	-4.263	-1.257	-0.001
C	-5.312	-2.241	-0.001

**C**	-6.621	-1.866	-0.002
**H**	-5.034	-3.295	-0.001
**C**	-6.982	-0.476	-0.002
**H**	-7.406	-2.620	-0.002
**C**	-6.022	0.489	-0.002
**H**	-8.036	-0.201	-0.003
**H**	-6.294	1.545	-0.003

Table S142 Frequencies (cm^{-1}) of hydrogen abstraction transition state between hydroxyl radical and α-chlorotetracene molecule, calculated at the M06-2X/cc-pVDZ level of theory.

-1453	41	62	74	91	141	143	166
186	219	229	292	297	332	360	403
405	432	480	481	494	520	534	562
571	583	631	638	689	720	759	762
769	776	790	804	810	844	861	870
891	907	911	916	944	947	963	997
1008	1018	1028	1116	1146	1155	1161	1175
1196	1221	1253	1285	1292	1323	1332	1348
1370	1399	1436	1454	1461	1486	1493	1501
1590	1614	1629	1641	1686	1697	1717	3192
3194	3195	3198	3201	3205	3217	3227	3229
3232	3779						

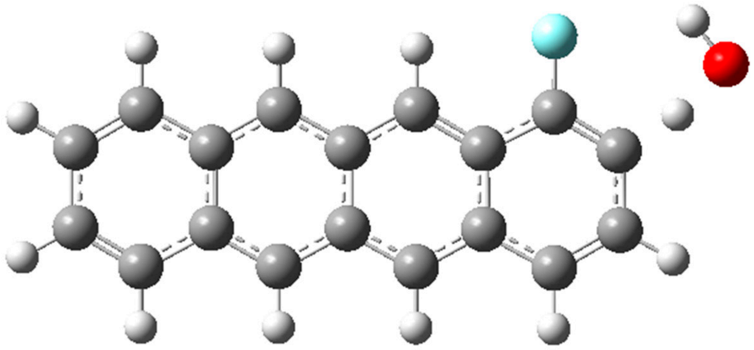

Figure S72 Visualization of hydrogen abstraction transition state between hydroxyl radical and α-fluorotetracene molecule, calculated at the M06-2X/cc-pVDZ level of theory.

Table S143 Geometry (Å) of hydrogen abstraction transition state between hydroxyl radical and α-fluorotetracene molecule, calculated at the M06-2X/cc-pVDZ level of theory

Atom	x	y	z
**C**	-4.859	1.411	-0.005
**C**	-3.691	2.110	-0.004
**C**	-2.425	1.430	-0.003
**C**	-2.416	-0.015	-0.003
**C**	-3.675	-0.709	-0.004

**C**	-4.851	-0.024	-0.006
**C**	-1.219	2.120	-0.002
**C**	-1.203	-0.690	-0.002
**C**	0.026	0.000	0.000
**C**	0.018	1.444	0.000
**C**	1.247	2.138	0.001
**H**	1.237	3.230	0.001
**C**	2.464	1.469	0.002
**C**	1.262	-0.679	0.001
**H**	-1.226	3.212	-0.001
**H**	-5.814	1.939	-0.006
**H**	-3.693	3.202	-0.004
**H**	-3.664	-1.800	-0.005
**H**	-5.800	-0.562	-0.007
**H**	-1.196	-1.783	-0.002
**H**	1.279	-1.769	0.001
**C**	2.457	0.024	0.002
**C**	3.732	-0.631	0.003
**C**	4.891	0.060	0.005
**C**	4.905	1.486	0.005
**C**	3.723	2.166	0.004
**H**	5.857	2.016	0.006
**H**	3.713	3.257	0.004
**H**	5.951	-0.607	0.006
**F**	3.736	-1.979	0.003
**O**	6.703	-1.568	0.006
**H**	6.026	-2.272	0.006

Table S144 Frequencies (cm^{-1}) of hydrogen abstraction transition state between hydroxyl radical and α-fluorotetracene molecule, calculated at the M06-2X/cc-pVDZ level of theory.

-1496	43	62	70	83	146	150	179
186	244	246	294	305	333	393	409
417	482	484	496	511	514	557	560
588	605	624	635	713	728	761	762
768	775	790	817	828	858	862	864
892	910	915	924	946	948	998	999
1018	1030	1064	1124	1153	1156	1176	1200
1212	1224	1262	1291	1306	1326	1349	1364
1382	1424	1440	1454	1474	1488	1498	1507
1594	1621	1628	1650	1689	1712	1728	3195
3197	3199	3199	3203	3209	3217	3221	3228
3232	3781						

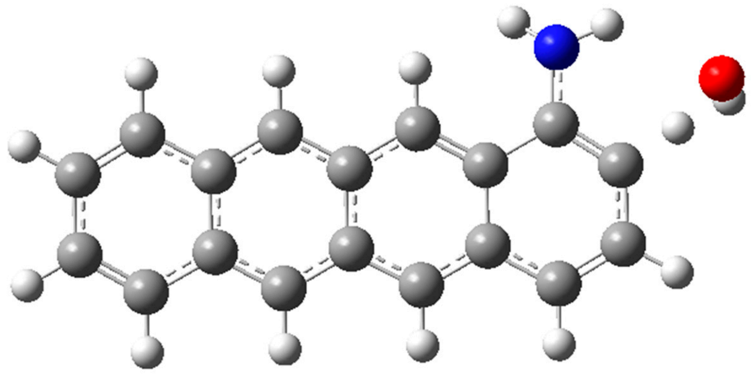

Figure S73 Visualization of hydrogen abstraction transition state between hydroxyl radical and α-aminotetracene molecule, calculated at the M06-2X/cc-pVDZ level of theory.

Table S145 Geometry (Å) of hydrogen abstraction transition state between hydroxyl radical and α-aminotetracene molecule, calculated at the M06-2X/cc-pVDZ level of theory

Atom	x	y	z
C	-1.372	-1.461	-0.046
C	-0.474	-0.407	-0.045
C	-0.965	0.954	0.021
C	-2.332	1.171	0.112
C	-3.259	0.105	0.133
H	-1.022	-2.488	-0.150
C	0.965	-0.621	-0.098
C	-0.035	2.051	-0.017
H	-2.705	2.194	0.163
C	1.303	1.816	-0.135
C	1.773	0.478	-0.224
H	-0.423	3.068	0.018
H	2.969	0.233	-0.312
H	2.010	2.641	-0.198
N	1.479	-1.903	-0.087
H	2.481	-1.938	0.070
H	0.957	-2.592	0.435
O	4.050	-0.291	0.161
H	4.075	0.116	1.043
C	-2.767	-1.246	0.043
C	-3.687	-2.313	0.040
H	-3.314	-3.335	-0.034
C	-5.549	-0.736	0.227
C	-4.648	0.323	0.225
H	-5.022	1.345	0.293
C	-5.056	-2.089	0.130
C	-6.004	-3.170	0.130
C	-7.341	-2.926	0.221
H	-5.628	-4.190	0.057
C	-7.831	-1.581	0.317

**H**	-8.050	-3.752	0.220
**C**	-6.968	-0.527	0.320
**H**	-8.904	-1.409	0.388
**H**	-7.338	0.496	0.393

Table S146 Frequencies (cm^{-1}) of hydrogen abstraction transition state between hydroxyl radical and α-aminotetracene molecule, calculated at the M06-2X/cc-pVDZ level of theory.

-1259	44	68	84	96	141	156	176
199	233	258	285	308	320	363	383
412	414	481	485	492	497	509	548
568	587	599	624	635	663	731	744
756	761	768	778	778	790	810	850
861	881	888	907	918	940	943	978
996	1015	1023	1030	1123	1127	1155	1166
1176	1198	1205	1222	1266	1286	1295	1318
1325	1353	1374	1389	1434	1445	1456	1470
1486	1502	1503	1586	1610	1629	1630	1651
1681	1694	1715	3194	3197	3197	3199	3204
3206	3207	3217	3224	3228	3558	3691	3809

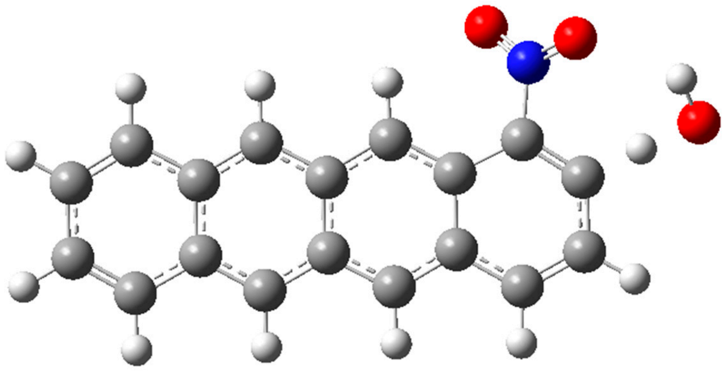

Figure S74 Visualization of hydrogen abstraction transition state between hydroxyl radical and α-nitrotetracene molecule, calculated at the M06-2X/cc-pVDZ level of theory.

Table S147 Geometry (Å) of hydrogen abstraction transition state between hydroxyl radical and α-nitrotetracene molecule, calculated at the M06-2X/cc-pVDZ level of theory

Atom	x	y	z
**O**	4.886	-2.618	-0.286
**O**	3.029	-2.786	0.776
**N**	3.896	-2.138	0.231
**C**	2.449	-0.043	0.160
**C**	2.453	1.409	0.137
**C**	3.754	-0.672	0.204
**C**	3.696	2.128	0.173
**C**	1.232	-0.711	0.106
**C**	1.252	2.101	0.069

**C**	4.896	0.061	0.225
**C**	4.894	1.481	0.212
**C**	0.010	-0.007	0.029
**C**	0.013	1.433	0.014
**H**	3.656	3.219	0.163
**H**	1.200	-1.797	0.129
**H**	1.268	3.193	0.051
**H**	6.033	-0.470	0.314
**H**	5.841	2.018	0.233
**C**	-1.223	-0.687	-0.029
**H**	-1.226	-1.779	-0.016
**C**	-2.428	0.001	-0.102
**C**	-2.423	1.446	-0.117
**C**	-1.213	2.125	-0.059
**H**	-1.207	3.218	-0.070
**C**	-3.691	-0.682	-0.162
**H**	-3.689	-1.774	-0.150
**C**	-4.859	0.013	-0.233
**H**	-5.810	-0.517	-0.278
**C**	-4.854	1.448	-0.248
**H**	-5.802	1.984	-0.305
**C**	-3.681	2.138	-0.192
**H**	-3.673	3.229	-0.203
**O**	7.103	-0.871	-0.079
**H**	6.743	-1.650	-0.548

Table S148 Frequencies (cm^{-1}) of hydrogen abstraction transition state between hydroxyl radical and α-nitrotetracene molecule, calculated at the M06-2X/cc-pVDZ level of theory.

-1631	37	52	54	73	114	136	163
168	209	222	278	285	304	327	338
362	389	414	444	478	488	494	521
529	557	575	606	633	646	683	723
751	760	766	775	784	790	795	823
837	862	870	880	895	911	911	930
943	949	998	1012	1019	1019	1028	1122
1155	1155	1171	1177	1198	1222	1260	1292
1299	1324	1335	1350	1363	1394	1436	1455
1461	1471	1485	1492	1500	1588	1614	1629
1637	1677	1686	1710	1720	3194	3196	3197
3199	3203	3207	3219	3231	3239	3259	3768

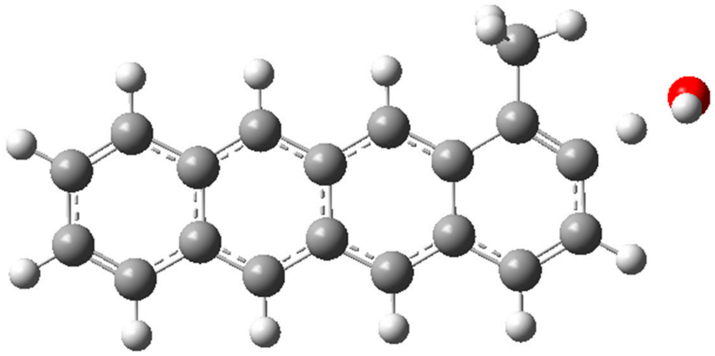

Figure S75 Visualization of hydrogen abstraction transition state between hydroxyl radical and α-methyltetracene molecule, calculated at the M06-2X/cc-pVDZ level of theory.

Table S149 Geometry (Å) of hydrogen abstraction transition state between hydroxyl radical and α-methyltetracene molecule, calculated at the M06-2X/cc-pVDZ level of theory

Atom	x	y	z
C	-4.884	1.396	0.046
C	-3.716	2.096	0.030
C	-2.449	1.416	0.018
C	-2.440	-0.028	0.022
C	-3.698	-0.724	0.039
C	-4.875	-0.040	0.050
C	-1.244	2.108	0.002
C	-1.226	-0.704	0.011
C	0.001	-0.009	-0.006
C	-0.008	1.432	-0.010
C	1.223	2.127	-0.027
H	1.199	3.216	-0.031
C	2.439	1.458	-0.039
C	2.446	0.010	-0.035
C	1.243	-0.681	-0.018
H	-1.252	3.199	-0.002
H	-5.837	1.922	0.055
H	-1.218	-1.794	0.014
C	3.704	-0.688	-0.047
H	-3.687	-1.814	0.042
H	-5.821	-0.578	0.063
H	1.254	-1.772	-0.016
H	3.696	-1.778	-0.046
C	4.878	-0.001	-0.066
C	4.839	1.427	-0.078
H	5.892	2.006	-0.116
C	3.704	2.172	-0.050
H	5.837	-0.516	-0.083
H	-3.720	3.185	0.026
C	3.734	3.675	-0.042
H	3.210	4.077	-0.919

**H**	3.233	4.070	0.852
**H**	4.768	4.032	-0.056
**O**	7.006	2.626	0.207
**H**	6.960	2.520	1.172

Table S150 Frequencies (cm^{-1}) of hydrogen abstraction transition state between hydroxyl radical and α-methyltetracene molecule, calculated at the M06-2X/cc-pVDZ level of theory.

-1085	41	68	74	81	150	154	158
173	218	250	257	290	303	328	378
401	427	477	482	492	496	523	550
559	578	597	633	636	719	749	760
770	776	780	792	813	829	843	861
888	896	910	916	945	946	996	997
1000	1017	1030	1051	1068	1081	1129	1155
1159	1176	1198	1201	1224	1264	1284	1293
1313	1327	1347	1362	1389	1403	1440	1453
1462	1471	1483	1487	1495	1504	1593	1628
1629	1645	1687	1695	1715	3057	3121	3170
3194	3195	3196	3198	3202	3202	3216	3217
3227	3228	3790					

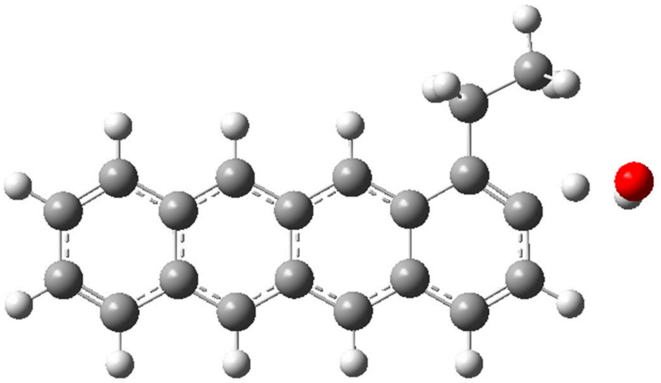

Figure S76 Visualization of hydrogen abstraction transition state between hydroxyl radical and α-ethyltetracene molecule, calculated at the M06-2X/cc-pVDZ level of theory.

Table S151 Geometry (Å) of hydrogen abstraction transition state between hydroxyl radical and α-ethyltetracene molecule, calculated at the M06-2X/cc-pVDZ level of theory

Atom	x	y	z
**C**	-4.831	1.398	-0.177
**C**	-3.704	2.158	-0.183
**C**	-2.432	1.446	-0.122
**C**	-2.428	0.000	-0.045
**C**	-3.679	-0.709	-0.039
**C**	-4.854	-0.028	-0.103
**C**	-1.216	2.117	-0.130
**C**	-1.222	-0.684	0.019

**C**	0.017	-0.009	0.012
**C**	0.017	1.429	-0.065
**C**	1.250	2.112	-0.073
**H**	1.253	3.201	-0.132
**C**	2.458	1.429	-0.009
**C**	2.456	-0.014	0.068
**C**	1.246	-0.696	0.077
**H**	-1.188	3.203	-0.189
**H**	-5.938	1.896	-0.247
**H**	-1.231	-1.774	0.076
**C**	3.718	-0.701	0.134
**H**	-3.664	-1.797	0.012
**H**	-5.811	-0.546	-0.111
**H**	1.244	-1.785	0.135
**H**	3.712	-1.789	0.192
**C**	4.891	-0.010	0.125
**C**	4.893	1.423	0.048
**H**	5.844	1.955	0.041
**C**	3.722	2.115	-0.017
**H**	3.720	3.203	-0.075
**H**	5.840	-0.542	0.175
**C**	-3.693	3.665	-0.266
**H**	-3.139	4.051	0.603
**H**	-3.100	3.947	-1.148
**C**	-5.058	4.337	-0.339
**H**	-5.654	4.135	0.558
**H**	-4.930	5.421	-0.430
**H**	-5.634	3.985	-1.202
**O**	-7.215	2.039	-0.033
**H**	-7.230	1.837	0.918

Table S152 Frequencies (cm^{-1}) of hydrogen abstraction transition state between hydroxyl radical and α-ethyltetracene molecule, calculated at the M06-2X/cc-pVDZ level of theory.

-1321	39	51	76	100	107	137	156
163	185	219	225	275	288	308	314
330	366	401	412	481	486	493	511
532	542	556	572	606	633	640	715
742	756	761	776	777	792	795	812
819	838	862	890	898	912	917	943
946	976	996	998	1016	1021	1029	1076
1097	1107	1128	1144	1155	1174	1193	1197
1221	1256	1285	1291	1296	1322	1331	1357
1372	1386	1402	1415	1449	1454	1472	1473
1480	1484	1486	1496	1503	1593	1629	1632
1641	1684	1693	1715	3048	3076	3084	3154
3160	3190	3192	3192	3196	3200	3200	3216

3224	3226	3228	3791

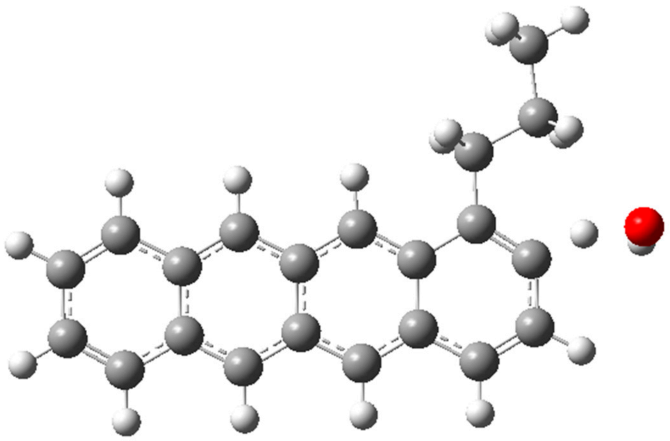

Figure S77 Visualization of hydrogen abstraction transition state between hydroxyl radical and α-propyltetracene molecule, calculated at the M06-2X/cc-pVDZ level of theory.

Table S153 Geometry (Å) of hydrogen abstraction transition state between hydroxyl radical and α-propyltetracene molecule, calculated at the M06-2X/cc-pVDZ level of theory

Atom	x	y	z
C	-1.194	2.134	-0.028
C	-2.401	1.510	-0.024
C	-2.477	0.074	-0.014
C	-1.253	-0.700	-0.010
C	0.034	-0.012	-0.007
C	-0.001	1.347	-0.024
C	-3.703	-0.575	-0.011
C	-1.340	-2.086	-0.005
C	-2.585	-2.756	-0.001
C	-3.800	-1.982	-0.004
C	-5.041	-2.651	-0.001
H	-5.960	-2.063	-0.003
C	-5.116	-4.038	0.006
C	-3.898	-4.815	0.008
C	-2.671	-4.163	0.005
H	-4.619	0.018	-0.015
H	-1.114	3.219	-0.047
H	-0.438	-2.694	-0.003
C	-3.997	-6.249	0.015
H	1.015	2.012	-0.045
H	-1.752	-4.751	0.007
H	-3.077	-6.832	0.017
C	-5.212	-6.865	0.018
C	-6.422	-6.093	0.015
H	-7.382	-6.608	0.017
C	-6.375	-4.732	0.009

**H**	-7.292	-4.143	0.007
**H**	-5.274	-7.952	0.022
**H**	-3.328	2.083	-0.034
**C**	1.299	-0.833	-0.005
**H**	1.278	-1.497	0.873
**H**	1.273	-1.498	-0.882
**C**	2.611	-0.058	-0.011
**H**	2.668	0.581	0.879
**H**	2.639	0.619	-0.874
**C**	3.810	-1.000	-0.050
**H**	4.752	-0.439	-0.051
**H**	3.814	-1.668	0.822
**H**	3.789	-1.625	-0.952
**O**	1.817	3.012	0.200
**H**	1.619	3.132	1.145

Table S154 Frequencies (cm^{-1}) of hydrogen abstraction transition state between hydroxyl radical and α-propyltetracene molecule, calculated at the M06-2X/cc-pVDZ level of theory.

-1307	38	44	77	84	103	116	123
146	162	181	185	230	250	252	293
303	324	329	364	402	418	481	490
492	509	533	554	558	575	618	633
641	721	741	757	760	764	777	782
791	808	821	836	860	875	893	898
912	914	928	943	946	989	997	1014
1018	1028	1077	1079	1110	1121	1136	1148
1157	1176	1193	1200	1224	1250	1260	1277
1296	1313	1322	1325	1344	1360	1379	1393
1403	1418	1448	1453	1464	1469	1476	1478
1484	1487	1496	1504	1594	1629	1632	1641
1683	1691	1718	3036	3056	3072	3083	3115
3138	3143	3195	3196	3197	3200	3204	3206
3219	3225	3229	3230	3791			

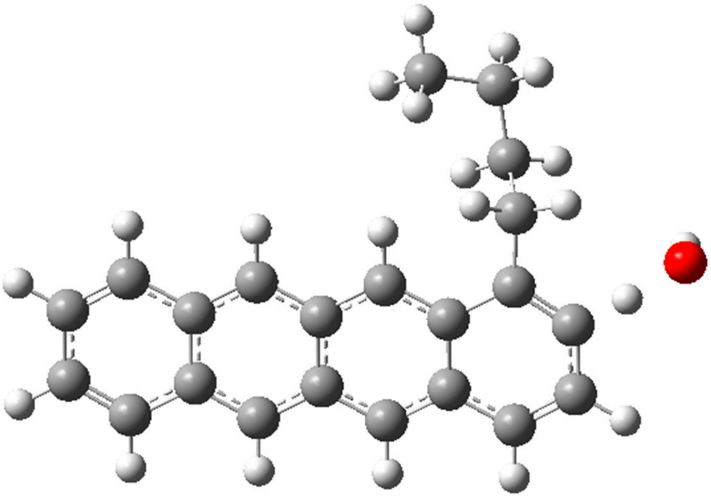

Figure S78 Visualization of hydrogen abstraction transition state between hydroxyl radical and α-butyltetracene molecule, calculated at the M06-2X/cc-pVDZ level of theory.

Table S155 Geometry (Å) of hydrogen abstraction transition state between hydroxyl radical and α-butyltetracene molecule, calculated at the M06-2X/cc-pVDZ level of theory

Atom	x	y	z
C	-1.240	2.146	-0.039
C	-2.443	1.507	-0.033
C	-2.510	0.070	-0.037
C	-1.283	-0.699	-0.042
C	-0.010	0.004	-0.039
C	-0.048	1.362	-0.057
C	-3.732	-0.588	-0.038
C	-1.358	-2.086	-0.043
C	-2.598	-2.763	-0.044
C	-3.818	-1.996	-0.041
C	-5.055	-2.673	-0.043
H	-5.978	-2.092	-0.041
C	-5.121	-4.060	-0.046
C	-3.898	-4.830	-0.048
C	-2.675	-4.171	-0.046
H	-4.653	-0.002	-0.038
H	-1.176	3.232	-0.044
H	-0.448	-2.685	-0.041
C	-3.989	-6.265	-0.051
H	1.002	1.948	-0.081
H	-1.752	-4.753	-0.049
H	-3.065	-6.843	-0.053
C	-5.199	-6.888	-0.052
C	-6.414	-6.123	-0.050
H	-7.371	-6.644	-0.051
C	-6.375	-4.762	-0.047
H	-7.296	-4.179	-0.046
H	-5.255	-7.976	-0.055

**H**	-3.374	2.073	-0.031
**C**	1.291	-0.753	0.009
**H**	1.254	-1.584	-0.707
**H**	2.099	-0.085	-0.312
**C**	1.609	-1.273	1.419
**H**	0.722	-1.760	1.848
**H**	1.828	-0.406	2.057
**C**	2.786	-2.249	1.445
**H**	3.094	-2.407	2.487
**H**	3.645	-1.797	0.929
**C**	2.454	-3.601	0.815
**H**	2.198	-3.504	-0.248
**H**	3.304	-4.290	0.884
**H**	1.602	-4.065	1.330
**O**	2.127	2.528	0.280
**H**	2.012	2.486	1.245

Table S156 Frequencies (cm^{-1}) of hydrogen abstraction transition state between hydroxyl radical and α-butyltetracene molecule, calculated at the M06-2X/cc-pVDZ level of theory.

-1097	22	37	51	61	70	78	111
127	146	160	162	196	226	243	271
289	306	321	328	384	411	423	428
477	482	494	505	530	555	570	595
615	634	648	726	739	753	759	771
776	780	788	791	812	831	852	861
884	896	901	909	918	941	942	950
978	992	997	1017	1027	1034	1076	1098
1107	1120	1130	1155	1159	1174	1194	1197
1215	1223	1258	1267	1292	1293	1306	1314
1321	1328	1348	1353	1362	1383	1391	1396
1438	1452	1462	1466	1468	1480	1483	1485
1490	1493	1501	1591	1626	1627	1642	1684
1693	1716	3054	3057	3060	3083	3098	3102
3130	3135	3143	3192	3194	3196	3199	3203
3203	3210	3217	3226	3229	3792		

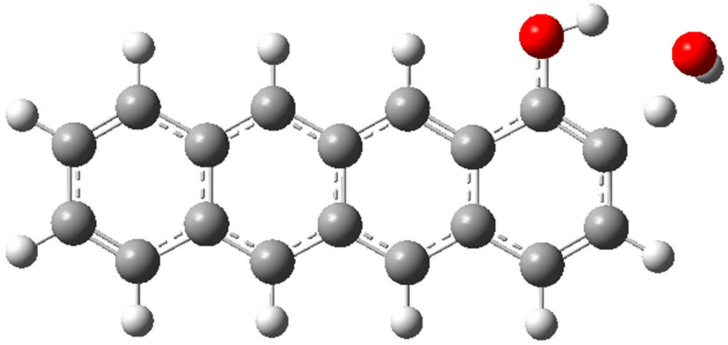

Figure S79 Visualization of hydrogen abstraction transition state between hydroxyl radical and α-hydroxytetracene molecule, calculated at the M06-2X/cc-pVDZ level of theory.

Table S157 Geometry (Å) of hydrogen abstraction transition state between hydroxyl radical and α-hydroxytetracene molecule, calculated at the M06-2X/cc-pVDZ level of theory

Atom	x	y	z
C	-4.845	1.396	0.039
C	-3.678	2.099	0.041
C	-2.410	1.424	0.019
C	-2.398	-0.020	-0.006
C	-3.654	-0.719	-0.006
C	-4.833	-0.038	0.015
C	-1.206	2.118	0.020
C	-1.181	-0.691	-0.027
C	0.044	0.004	-0.025
C	0.033	1.447	-0.001
C	1.263	2.143	0.000
H	1.250	3.235	0.020
C	2.480	1.478	-0.022
C	1.285	-0.671	-0.050
H	-1.216	3.210	0.039
H	-5.801	1.921	0.056
H	-3.683	3.190	0.059
H	-3.639	-1.810	-0.025
H	-5.779	-0.579	0.014
H	-1.169	-1.784	-0.046
H	1.310	-1.761	-0.069
C	2.477	0.033	-0.053
C	3.750	-0.655	-0.057
C	4.898	0.084	-0.120
C	4.917	1.505	-0.044
C	3.735	2.184	-0.008
H	5.868	2.039	-0.063
H	3.721	3.275	0.006
H	5.915	-0.626	-0.196
O	3.731	-1.996	-0.011
H	4.666	-2.283	0.050

**O**	6.476	-1.685	0.237
**H**	6.573	-1.432	1.175

Table S158 Frequencies (cm^{-1}) of hydrogen abstraction transition state between hydroxyl radical and α-hydroxytetracene molecule, calculated at the M06-2X/cc-pVDZ level of theory.

-1359	47	70	90	100	145	163	183
213	256	286	301	319	326	373	409
411	482	489	496	508	518	547	567
594	618	628	635	710	732	742	751
761	764	776	778	793	812	847	863
885	892	917	917	947	956	978	997
1016	1031	1056	1125	1137	1154	1176	1188
1205	1220	1254	1267	1290	1296	1321	1338
1355	1374	1404	1441	1456	1468	1486	1492
1503	1544	1586	1614	1629	1632	1677	1692
1714	3195	3197	3198	3199	3203	3206	3217
3223	3225	3228	3626	3808			

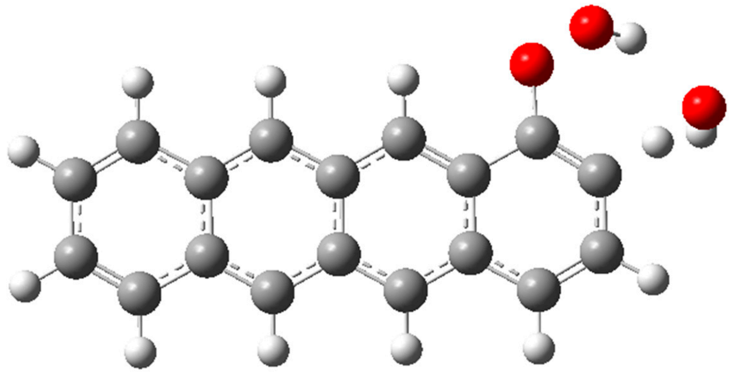

Figure S80 Visualization of hydrogen abstraction transition state between hydroxyl radical and α-peroxytetracene molecule, calculated at the M06-2X/cc-pVDZ level of theory.

Table S159 Geometry (Å) of hydrogen abstraction transition state between hydroxyl radical and α-peroxytetracene molecule, calculated at the M06-2X/cc-pVDZ level of theory

Atom	x	y	z
**C**	-4.864	1.440	0.193
**C**	-3.688	2.126	0.207
**C**	-2.431	1.438	0.105
**C**	-2.440	-0.001	-0.014
**C**	-3.705	-0.683	-0.024
**C**	-4.873	0.010	0.075
**C**	-1.218	2.116	0.116
**C**	-1.234	-0.685	-0.112
**C**	0.000	-0.006	-0.099
**C**	0.010	1.431	0.016
**C**	1.249	2.110	0.020

**H**	1.254	3.199	0.099
**C**	2.454	1.430	-0.077
**C**	1.229	-0.696	-0.201
**H**	-1.212	3.205	0.204
**H**	-5.812	1.974	0.271
**H**	-3.678	3.214	0.296
**H**	-3.706	-1.771	-0.114
**H**	-5.827	-0.519	0.065
**H**	-1.238	-1.774	-0.202
**H**	1.231	-1.782	-0.293
**C**	2.433	-0.011	-0.186
**C**	3.701	-0.705	-0.258
**C**	4.857	0.011	-0.302
**C**	4.887	1.431	-0.184
**C**	3.717	2.121	-0.082
**H**	5.845	1.951	-0.206
**H**	3.717	3.210	-0.019
**H**	5.937	-0.615	-0.308
**O**	3.609	-2.063	-0.239
**O**	4.692	-2.688	-0.901
**H**	5.443	-2.541	-0.284
**O**	6.737	-1.363	0.300
**H**	6.604	-1.043	1.212

Table S160 Frequencies (cm^{-1}) of hydrogen abstraction transition state between hydroxyl radical and α-peroxytetracene molecule, calculated at the M06-2X/cc-pVDZ level of theory.

-1451	42	55	78	83	115	152	174
189	214	234	276	291	313	322	335
374	400	412	481	489	494	505	534
550	561	576	605	619	633	659	734
758	762	764	776	789	790	810	820
836	862	889	889	913	917	942	949
985	997	997	1016	1029	1088	1124	1138
1154	1174	1184	1197	1218	1242	1265	1288
1305	1324	1347	1362	1390	1409	1441	1455
1470	1486	1498	1503	1514	1589	1620	1629
1636	1681	1693	1716	3192	3194	3195	3197
3201	3206	3217	3224	3225	3229	3623	3806

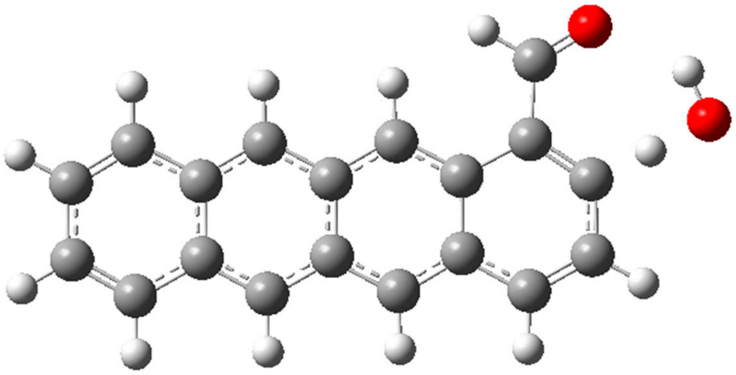

Figure S81 Visualization of hydrogen abstraction transition state between hydroxyl radical and α-tetraldehyde molecule, calculated at the M06-2X/cc-pVDZ level of theory.

Table S161 Geometry (Å) of hydrogen abstraction transition state between hydroxyl radical and α-tetraldehyde molecule, calculated at the M06-2X/cc-pVDZ level of theory

Atom	x	y	z
C	-4.859	1.418	-0.026
C	-3.692	2.118	-0.014
C	-2.425	1.438	-0.006
C	-2.415	-0.007	-0.011
C	-3.673	-0.702	-0.023
C	-4.850	-0.017	-0.031
C	-1.220	2.128	0.005
C	-1.202	-0.683	-0.004
C	0.027	0.008	0.009
C	0.016	1.448	0.013
C	1.248	2.130	0.019
H	1.251	3.222	0.013
C	2.459	1.450	0.027
C	1.264	-0.676	0.017
H	-1.224	3.220	0.007
H	-5.814	1.945	-0.032
H	-3.693	3.209	-0.011
H	-3.662	-1.793	-0.027
H	-5.798	-0.556	-0.040
H	-1.197	-1.775	-0.007
H	1.226	-1.764	0.004
C	2.478	0.001	0.033
C	3.777	-0.659	0.045
C	4.906	0.109	0.036
C	4.899	1.528	0.018
C	3.699	2.175	0.020
H	5.841	2.075	0.008
H	3.656	3.266	0.013
H	6.047	-0.402	0.089
C	3.894	-2.138	0.079
O	4.932	-2.751	-0.050

**H**	2.954	-2.704	0.244
**O**	7.100	-0.966	-0.144
**H**	6.690	-1.845	-0.273

Table S162 Frequencies (cm^{-1}) of hydrogen abstraction transition state between hydroxyl radical and α-tetraldehyde molecule, calculated at the M06-2X/cc-pVDZ level of theory.

-1570	42	54	79	96	103	142	173
180	223	237	294	296	319	330	385
399	423	437	476	479	491	534	534
542	573	578	632	644	657	738	759
763	767	773	777	793	818	838	853
861	886	905	910	916	921	946	947
998	1019	1021	1028	1029	1045	1124	1152
1156	1175	1197	1200	1222	1260	1294	1297
1324	1339	1355	1373	1401	1435	1443	1456
1478	1484	1489	1501	1588	1611	1629	1635
1680	1690	1716	1807	3041	3192	3194	3195
3198	3199	3202	3217	3229	3232	3234	3720

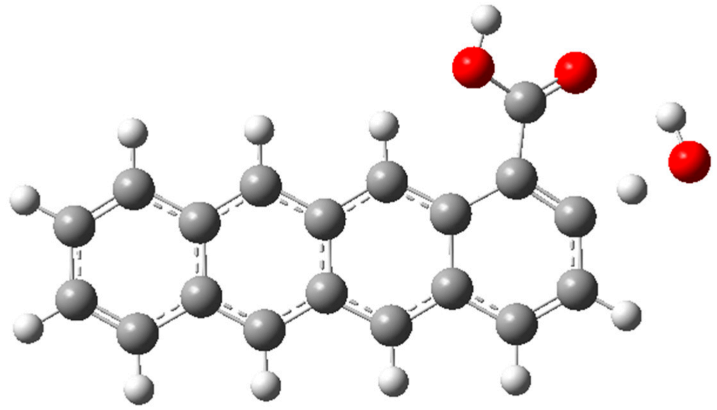

Figure S82 Visualization of hydrogen abstraction transition state between hydroxyl radical and α-tetracenic acid molecule, calculated at the M06-2X/cc-pVDZ level of theory.

Table S163 Geometry (Å) of hydrogen abstraction transition state between hydroxyl radical and α-tetracenic acid molecule, calculated at the M06-2X/cc-pVDZ level of theory

Atom	x	y	z
**C**	-4.881	1.473	0.061
**C**	-3.705	2.158	0.029
**C**	-2.447	1.462	0.017
**C**	-2.455	0.017	0.040
**C**	-3.722	-0.661	0.073
**C**	-4.890	0.038	0.084
**C**	-1.233	2.136	-0.016
**C**	-1.251	-0.674	0.028
**C**	-0.013	0.000	-0.006

**C**	-0.007	1.440	-0.027
**C**	1.237	2.099	-0.052
**H**	1.259	3.191	-0.057
**C**	2.436	1.400	-0.062
**C**	1.211	-0.706	-0.020
**H**	-1.223	3.229	-0.032
**H**	-5.829	2.012	0.070
**H**	-3.693	3.249	0.011
**H**	-3.724	-1.752	0.091
**H**	-5.844	-0.488	0.110
**H**	-1.257	-1.767	0.045
**H**	1.168	-1.791	-0.006
**C**	2.437	-0.053	-0.055
**C**	3.736	-0.717	-0.056
**C**	4.866	0.048	-0.042
**C**	4.873	1.468	-0.046
**C**	3.679	2.120	-0.063
**H**	5.822	2.002	-0.036
**H**	3.640	3.212	-0.071
**H**	6.006	-0.461	-0.088
**C**	3.946	-2.196	-0.058
**O**	4.962	-2.745	0.308
**O**	2.916	-2.913	-0.536
**H**	3.205	-3.841	-0.496
**O**	7.082	-0.917	0.272
**H**	6.698	-1.759	0.591

Table S164 Frequencies (cm^{-1}) of hydrogen abstraction transition state between hydroxyl radical and α-tetracenic acid molecule, calculated at the M06-2X/cc-pVDZ level of theory.

-1570	35	48	52	76	108	133	166
175	209	221	280	288	315	324	362
377	399	419	430	478	487	496	525
531	543	569	591	630	633	647	655
734	736	760	764	771	777	791	794
809	829	854	864	888	902	914	916
928	944	953	998	1004	1018	1018	1027
1119	1143	1155	1164	1174	1197	1222	1231
1262	1292	1298	1323	1333	1350	1373	1397
1413	1440	1457	1460	1484	1490	1500	1590
1618	1628	1638	1683	1690	1718	1819	3194
3195	3197	3200	3203	3204	3219	3230	3236
3270	3741	3805					

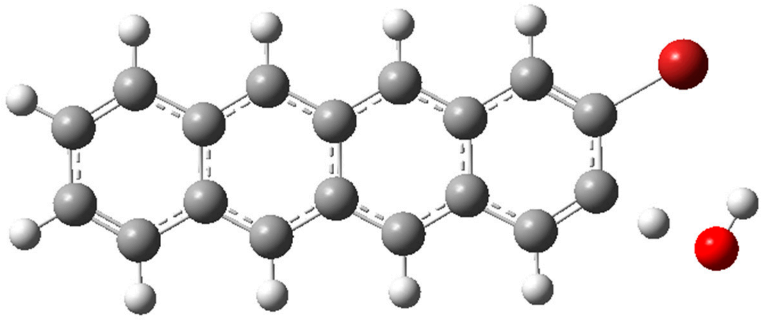

Figure S83 Visualization of hydrogen abstraction transition state between hydroxyl radical and β-bromotetracene molecule, calculated at the M06-2X/cc-pVDZ level of theory.

Table S165 Geometry (Å) of hydrogen abstraction transition state between hydroxyl radical and β-bromotetracene molecule, calculated at the M06-2X/cc-pVDZ level of theory

Atom	x	y	z
C	-4.156	-0.797	0.002
C	-2.898	-1.385	0.012
C	-1.722	-0.607	0.015
C	-1.834	0.832	0.007
C	-3.116	1.419	-0.004
C	-4.268	0.644	-0.007
C	-0.439	-1.195	0.026
C	-0.657	1.611	0.010
C	0.598	1.021	0.021
C	0.712	-0.419	0.029
C	2.019	-1.020	0.040
H	2.107	-2.106	0.046
C	3.128	-0.233	0.042
C	1.806	1.808	0.024
H	-0.355	-2.282	0.032
H	-2.814	-2.473	0.018
H	-3.201	2.507	-0.010
H	-0.740	2.698	0.004
H	1.737	2.895	0.018
Br	4.860	-1.009	0.057
C	-5.580	1.231	-0.018
H	-5.662	2.318	-0.024
C	-6.695	0.450	-0.020
H	-7.684	0.906	-0.028
C	-6.584	-0.982	-0.012
H	-7.490	-1.586	-0.014
C	-5.361	-1.581	-0.002
H	-5.273	-2.667	0.005
C	3.009	1.188	0.034
H	4.014	1.887	0.037
O	5.132	2.474	0.040

**H**	5.705	1.686	0.047

Table S166 Frequencies (cm^{-1}) of hydrogen abstraction transition state between hydroxyl radical and β-bromotetracene molecule, calculated at the M06-2X/cc-pVDZ level of theory.

-1478	35	47	91	92	116	118	178
193	217	224	239	299	330	345	351
388	456	460	477	487	501	526	539
569	604	637	646	664	732	761	771
774	781	789	801	802	806	859	872
889	892	910	920	934	947	947	958
998	1018	1028	1106	1122	1154	1174	1185
1213	1222	1258	1279	1290	1321	1322	1343
1371	1391	1438	1453	1472	1480	1488	1502
1590	1618	1627	1633	1677	1689	1716	3194
3196	3197	3199	3199	3203	3210	3217	3220
3229	3769						

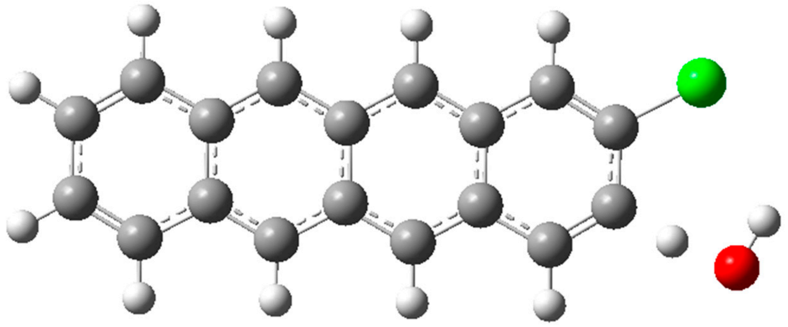

Figure S84 Visualization of hydrogen abstraction transition state between hydroxyl radical and β-chlorotetracene acid molecule, calculated at the M06-2X/cc-pVDZ level of theory.

Table S167 Geometry (Å) of hydrogen abstraction transition state between hydroxyl radical and β-chlorotetracene molecule, calculated at the M06-2X/cc-pVDZ level of theory

Atom	x	y	z
**C**	-3.582	-0.733	-0.003
**C**	-2.357	-1.389	-0.014
**C**	-1.140	-0.676	-0.014
**C**	-1.174	0.766	-0.004
**C**	-2.422	1.423	0.007
**C**	-3.615	0.711	0.007
**C**	0.108	-1.334	-0.025
**C**	0.044	1.480	-0.005
**C**	1.265	0.823	-0.015
**C**	1.300	-0.622	-0.025
**C**	2.573	-1.293	-0.036
**H**	2.607	-2.381	-0.044

**C**	3.722	-0.566	-0.036
**C**	2.514	1.544	-0.016
**H**	0.133	-2.424	-0.032
**H**	-2.333	-2.480	-0.021
**H**	-2.447	2.513	0.014
**H**	0.020	2.570	0.003
**H**	2.501	2.633	-0.008
**C**	-4.893	1.370	0.018
**H**	-4.914	2.460	0.025
**C**	-6.049	0.651	0.018
**H**	-7.011	1.161	0.026
**C**	-6.016	-0.784	0.008
**H**	-6.954	-1.338	0.008
**C**	-4.828	-1.450	-0.003
**H**	-4.800	-2.539	-0.010
**C**	3.681	0.860	-0.026
**H**	4.731	1.493	-0.026
**O**	5.896	1.972	-0.029
**H**	6.395	1.136	-0.037
**Cl**	5.277	-1.358	-0.049

Table S168 Frequencies (cm^{-1}) of hydrogen abstraction transition state between hydroxyl radical and β-chlorotetracene molecule, calculated at the M06-2X/cc-pVDZ level of theory.

-1472	35	52	90	90	121	136	153
198	227	228	267	302	345	354	381
389	464	470	477	487	500	530	542
576	610	638	652	680	731	759	773
775	782	791	796	806	809	859	872
890	892	912	920	935	952	961	963
998	1018	1027	1115	1124	1155	1175	1188
1214	1225	1261	1282	1294	1322	1325	1344
1374	1393	1441	1453	1478	1481	1489	1503
1591	1622	1628	1637	1682	1692	1718	3196
3198	3200	3201	3201	3204	3213	3219	3221
3230	3776						

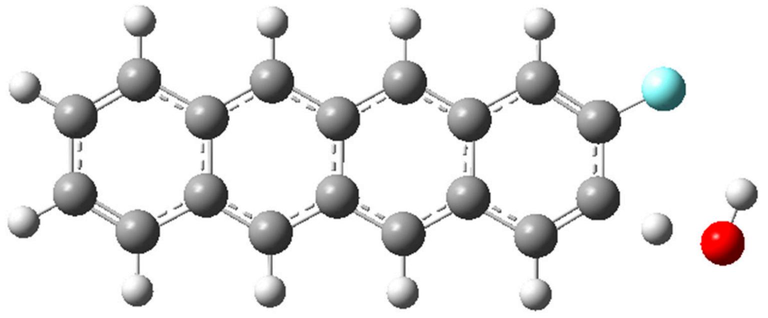

Figure S85 Visualization of hydrogen abstraction transition state between hydroxyl radical and β-fluorotetracene molecule, calculated at the M06-2X/cc-pVDZ level of theory.

Table S169 Geometry (Å) of hydrogen abstraction transition state between hydroxyl radical and β-fluorotetracene molecule, calculated at the M06-2X/cc-pVDZ level of theory

Atom	x	y	z
C	-3.670	0.675	-0.001
C	-2.508	1.385	-0.001
C	-1.235	0.716	0.000
C	-1.213	-0.729	-0.001
C	-2.465	-1.436	-0.001
C	-3.648	-0.761	-0.002
C	-0.037	1.417	0.000
C	0.006	-1.395	0.000
C	1.229	-0.693	0.000
C	1.206	0.750	0.001
C	2.430	1.453	0.001
H	2.415	2.543	0.002
C	3.646	0.786	0.002
C	2.473	-1.360	0.001
H	-0.053	2.508	0.001
H	-4.628	1.192	-0.001
H	-2.521	2.474	0.000
H	-2.446	-2.525	-0.002
H	-4.590	-1.307	-0.002
H	0.021	-2.486	-0.001
H	2.487	-2.450	0.000
C	3.671	-0.661	0.001
C	4.939	-1.341	0.002
C	6.078	-0.608	0.002
H	4.985	-2.429	0.001
C	4.897	1.505	0.003
H	4.885	2.594	0.003
F	7.289	-1.206	0.003
C	6.059	0.814	0.003
H	7.149	1.390	0.003
O	8.371	1.661	0.004
H	8.725	0.754	0.004

Table S170 Frequencies (cm^{-1}) of hydrogen abstraction transition state between hydroxyl radical and β-fluorotetracene molecule, calculated at the M06-2X/cc-pVDZ level of theory.

-1510	36	60	77	95	134	151	186
226	245	268	274	310	368	371	394
420	478	482	487	495	500	538	547
596	615	638	652	730	744	759	772
777	782	789	810	816	830	859	869
892	892	915	917	932	949	958	998

1017	1019	1028	1122	1156	1170	1176	1196
1215	1251	1266	1283	1296	1323	1333	1348
1385	1406	1449	1453	1485	1489	1503	1516
1595	1628	1637	1646	1688	1708	1721	3196
3198	3200	3201	3201	3205	3215	3219	3228
3230	3778						

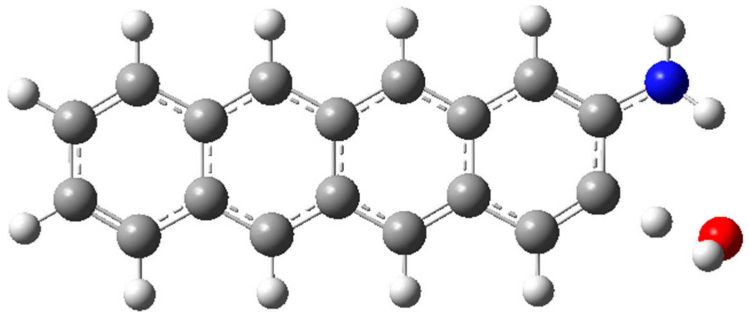

Figure S86 Visualization of hydrogen abstraction transition state between hydroxyl radical and β-aminotetracene molecule, calculated at the M06-2X/cc-pVDZ level of theory.

Table S171 Geometry (Å) of hydrogen abstraction transition state between hydroxyl radical and β-aminotetracene molecule, calculated at the M06-2X/cc-pVDZ level of theory

Atom	x	y	z
C	0.382	1.433	0.006
C	1.605	0.774	-0.030
C	1.611	-0.673	-0.074
C	0.418	-1.377	-0.078
C	-0.827	-0.712	-0.040
H	0.362	2.523	0.038
C	2.852	1.485	-0.023
C	2.886	-1.347	-0.100
H	0.437	-2.467	-0.113
C	4.019	-0.614	-0.121
C	4.052	0.823	-0.066
H	2.913	-2.436	-0.113
H	5.104	-1.150	-0.167
H	2.832	2.575	0.004
N	5.286	1.443	-0.128
H	5.330	2.405	0.173
H	6.080	0.871	0.130
O	6.306	-1.451	0.246
H	6.085	-1.765	1.140
C	-0.842	0.730	0.003
C	-2.086	1.394	0.040
C	-3.286	0.693	0.035
H	-2.104	2.484	0.072

C	-2.048	-1.415	-0.044
C	-4.558	1.362	0.073
C	-3.268	-0.750	-0.008
H	-2.032	-2.505	-0.078
C	-5.721	0.654	0.068
H	-4.570	2.451	0.105
C	-4.521	-1.456	-0.012
C	-5.703	-0.781	0.025
H	-6.678	1.173	0.096
H	-4.502	-2.545	-0.044
H	-6.646	-1.326	0.021

Table S172 Frequencies (cm^{-1}) of hydrogen abstraction transition state between hydroxyl radical and β-aminotetracene molecule, calculated at the M06-2X/cc-pVDZ level of theory.

-1205	38	64	88	103	133	157	186
230	239	265	270	303	356	369	389
403	427	470	474	481	491	498	503
540	553	598	628	639	672	726	752
756	758	770	778	789	809	834	842
858	872	888	901	916	917	938	947
958	994	1014	1028	1106	1130	1154	1174
1186	1197	1209	1217	1277	1279	1288	1301
1324	1332	1350	1364	1396	1444	1452	1487
1491	1504	1514	1592	1624	1627	1634	1650
1686	1702	1719	3192	3193	3194	3194	3196
3198	3202	3208	3217	3228	3586	3715	3790

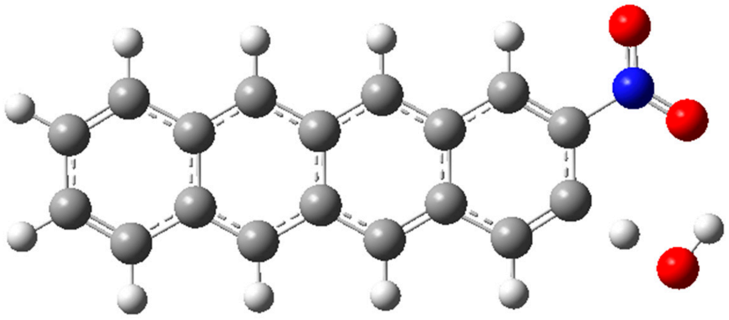

Figure S87 Visualization of hydrogen abstraction transition state between hydroxyl radical and β-nitrotetracene molecule, calculated at the M06-2X/cc-pVDZ level of theory.

Table S173 Geometry (Å) of hydrogen abstraction transition state between hydroxyl radical and β-nitrotetracene molecule, calculated at the M06-2X/cc-pVDZ level of theory

Atom	x	y	z
C	-3.648	0.719	-0.001
C	-2.475	1.410	0.000

**C**	-1.214	0.721	0.000
**C**	-1.217	-0.724	-0.001
**C**	-2.479	-1.412	-0.002
**C**	-3.650	-0.717	-0.002
**C**	-0.004	1.405	0.001
**C**	-0.009	-1.409	-0.001
**C**	1.224	-0.724	0.000
**C**	1.227	0.720	0.001
**C**	2.462	1.406	0.001
**H**	2.463	2.497	0.002
**C**	3.667	0.722	0.002
**C**	2.455	-1.411	0.000
**H**	-0.005	2.495	0.001
**H**	-4.597	1.252	-0.001
**H**	-2.471	2.500	0.000
**H**	-2.477	-2.501	-0.002
**H**	-4.601	-1.247	-0.002
**H**	-0.009	-2.500	-0.001
**H**	2.457	-2.501	0.000
**C**	3.663	-0.724	0.001
**C**	4.912	-1.425	0.001
**C**	6.080	-0.725	0.002
**H**	4.936	-2.513	0.000
**C**	4.936	1.408	0.002
**H**	4.953	2.497	0.003
**N**	7.345	-1.474	0.002
**O**	7.289	-2.686	0.001
**O**	8.382	-0.834	0.003
**C**	6.089	0.702	0.002
**H**	7.130	1.371	0.003
**O**	8.162	2.044	0.004
**H**	8.775	1.287	0.004

Table S174 Frequencies (cm^{-1}) of hydrogen abstraction transition state between hydroxyl radical and β-nitrotetracene molecule, calculated at the M06-2X/cc-pVDZ level of theory.

-1612	34	45	50	94	96	122	155
183	211	226	261	281	326	329	350
359	393	446	451	478	488	502	523
533	556	566	612	638	652	673	732
758	760	766	772	774	788	791	808
813	862	866	880	892	902	916	932
939	955	971	999	1005	1019	1028	1123
1136	1155	1175	1187	1215	1229	1263	1281
1292	1324	1331	1347	1374	1398	1442	1453
1460	1480	1484	1489	1505	1594	1627	1628
1637	1677	1684	1712	1719	3197	3199	3201

3201	3203	3205	3212	3220	3231	3235	3779

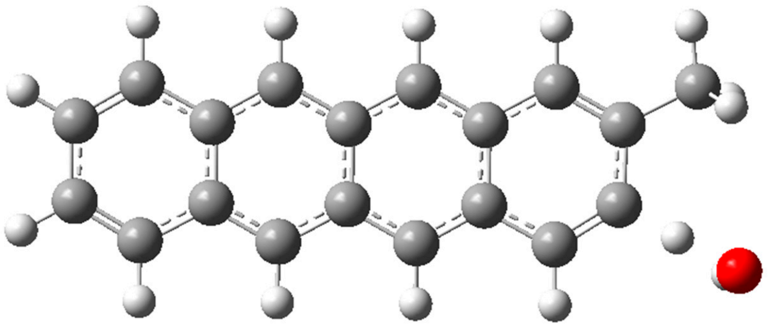

Figure S88 Visualization of hydrogen abstraction transition state between hydroxyl radical and β-methyltetracene molecule, calculated at the M06-2X/cc-pVDZ level of theory.

Table S175 Geometry (Å) of hydrogen abstraction transition state between hydroxyl radical and β-methyltetracene molecule, calculated at the M06-2X/cc-pVDZ level of theory

Atom	x	y	z
C	-3.665	0.720	-0.009
C	-2.492	1.411	-0.033
C	-1.229	0.724	-0.014
C	-1.229	-0.720	0.032
C	-2.491	-1.407	0.056
C	-3.664	-0.715	0.037
C	-0.020	1.407	-0.038
C	-0.020	-1.403	0.051
C	1.214	-0.720	0.027
C	1.212	0.722	-0.019
C	2.448	1.406	-0.044
H	2.451	2.496	-0.080
C	3.652	0.719	-0.025
C	2.448	-1.405	0.045
H	-0.020	2.497	-0.073
H	-4.615	1.251	-0.024
H	-2.489	2.501	-0.068
H	-2.488	-2.496	0.091
H	-4.614	-1.247	0.056
H	-0.021	-2.494	0.085
H	2.446	-2.495	0.079
C	3.657	-0.725	0.021
C	4.918	-1.420	0.040
C	6.111	-0.758	0.016
H	4.904	-2.511	0.072
C	4.922	1.403	-0.044
H	4.942	2.492	-0.072
C	7.442	-1.454	0.030

**H**	7.315	-2.541	0.052
**H**	8.028	-1.181	-0.857
**H**	8.029	-1.146	0.905
**C**	6.062	0.676	-0.039
**H**	7.116	1.255	-0.084
**O**	8.232	1.870	0.235
**H**	8.048	2.034	1.175

Table S176 Frequencies (cm^{-1}) of hydrogen abstraction transition state between hydroxyl radical and β-methyltetracene molecule, calculated at the M06-2X/cc-pVDZ level of theory.

-1112	38	58	63	91	111	126	146
162	186	237	253	276	301	352	357
388	430	466	477	482	489	497	536
551	586	622	638	656	736	739	758
763	774	778	789	814	833	856	860
880	894	907	914	920	944	947	968
995	1015	1024	1029	1056	1099	1126	1155
1174	1187	1195	1214	1234	1277	1280	1288
1313	1325	1338	1349	1388	1401	1430	1451
1459	1465	1488	1489	1492	1505	1591	1624
1628	1644	1687	1706	1720	3065	3131	3162
3190	3194	3195	3196	3197	3199	3203	3207
3218	3229	3789					

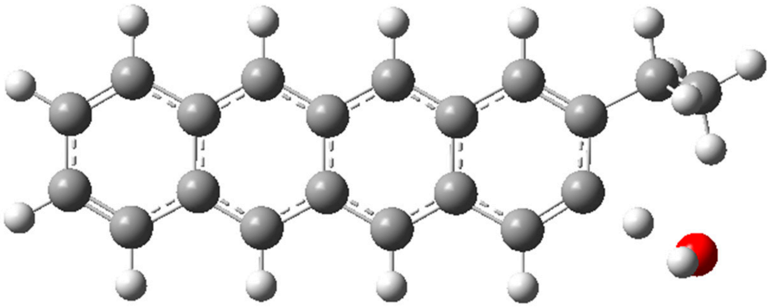

Figure S89 Visualization of hydrogen abstraction transition state between hydroxyl radical and β-ethyltetracene molecule, calculated at the M06-2X/cc-pVDZ level of theory.

Table S177 Geometry (Å) of hydrogen abstraction transition state between hydroxyl radical and β-ethyltetracene molecule, calculated at the M06-2X/cc-pVDZ level of theory

Atom	x	y	z
**C**	4.928	1.291	0.125
**C**	3.754	1.987	0.091
**C**	2.474	1.327	0.069
**C**	2.429	-0.117	0.087
**C**	3.677	-0.837	0.133

**C**	4.838	-0.142	0.131
**C**	1.285	2.041	0.032
**C**	1.206	-0.770	0.068
**C**	-0.010	-0.052	0.030
**C**	0.032	1.391	0.012
**C**	-1.181	2.109	-0.026
**H**	-1.152	3.199	-0.039
**C**	-2.409	1.458	-0.046
**C**	-2.450	0.015	-0.028
**C**	-1.260	-0.702	0.010
**H**	1.314	3.132	0.018
**H**	1.178	-1.860	0.080
**C**	-3.731	-0.638	-0.049
**H**	3.665	-1.926	0.160
**H**	5.874	-0.751	0.147
**H**	-1.291	-1.792	0.023
**H**	-3.758	-1.727	-0.036
**C**	-4.883	0.086	-0.085
**C**	-4.843	1.521	-0.103
**H**	-5.777	2.080	-0.132
**C**	-3.652	2.181	-0.084
**H**	-3.618	3.270	-0.097
**H**	-5.848	-0.419	-0.101
**C**	6.281	1.950	0.111
**H**	6.153	3.031	0.251
**H**	6.872	1.573	0.958
**C**	7.041	1.671	-1.190
**H**	8.014	2.176	-1.182
**H**	7.217	0.595	-1.310
**H**	6.467	2.029	-2.053
**H**	3.772	3.078	0.077
**O**	6.975	-1.363	0.534
**H**	6.751	-1.493	1.471

Table S178 Frequencies (cm^{-1}) of hydrogen abstraction transition state between hydroxyl radical and β-ethyltetracene molecule, calculated at the M06-2X/cc-pVDZ level of theory.

-1090	36	54	68	77	94	115	128
144	172	202	211	261	271	275	324
361	376	407	430	469	477	485	496
507	533	553	601	629	640	682	732
738	760	763	774	777	780	789	814
834	859	864	882	894	911	913	922
946	950	958	992	997	1016	1028	1078
1091	1112	1128	1155	1175	1188	1196	1215
1232	1259	1281	1286	1290	1316	1324	1338
1342	1353	1386	1391	1435	1452	1461	1474

1478	1484	1488	1492	1504	1591	1624	1627
1644	1688	1704	1718	3064	3068	3118	3147
3153	3189	3194	3195	3196	3197	3199	3202
3207	3217	3228	3785				

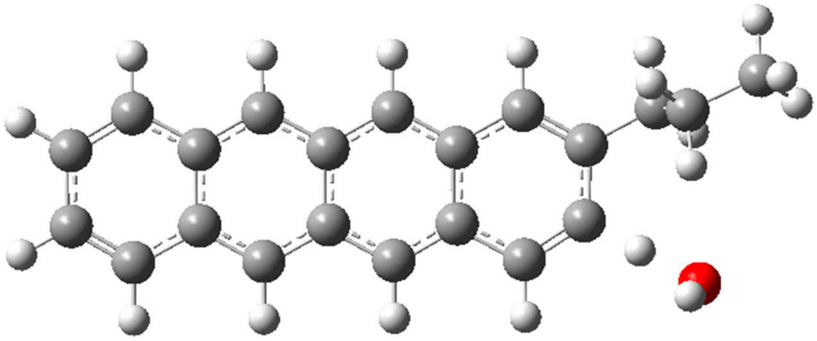

Figure S90 Visualization of hydrogen abstraction transition state between hydroxyl radical and β-propyltetracene molecule, calculated at the M06-2X/cc-pVDZ level of theory.

Table S179 Geometry (Å) of hydrogen abstraction transition state between hydroxyl radical and β-propyltetracene molecule, calculated at the M06-2X/cc-pVDZ level of theory

Atom	x	y	z
C	1.221	2.038	-0.049
C	2.420	1.410	-0.041
C	2.458	-0.031	-0.028
C	1.202	-0.747	-0.013
C	-0.028	0.001	-0.011
C	-0.047	1.366	-0.021
C	3.653	-0.734	-0.031
C	1.214	-2.134	0.000
C	2.423	-2.863	0.000
C	3.675	-2.145	-0.016
C	4.883	-2.873	-0.016
H	5.829	-2.330	-0.028
C	4.892	-4.262	-0.002
C	3.640	-4.981	0.014
C	2.444	-4.273	0.015
H	4.598	-0.189	-0.045
H	1.199	3.237	-0.103
H	0.269	-2.679	0.011
C	3.672	-6.419	0.029
H	-0.968	-0.553	-0.003
H	1.499	-4.817	0.026
H	2.726	-6.959	0.041
C	4.857	-7.091	0.029
C	6.102	-6.376	0.013
H	7.036	-6.935	0.013
C	6.118	-5.014	-0.002

**H**	7.061	-4.469	-0.014
**H**	4.868	-8.180	0.041
**H**	3.355	1.969	-0.059
**C**	-1.317	2.171	0.029
**H**	-1.237	3.020	-0.664
**H**	-2.159	1.547	-0.300
**C**	-1.601	2.708	1.437
**H**	-1.710	1.858	2.125
**H**	-0.733	3.287	1.777
**C**	-2.849	3.584	1.470
**H**	-3.730	3.022	1.130
**H**	-3.052	3.951	2.483
**H**	-2.726	4.453	0.811
**O**	1.175	4.528	0.168
**H**	1.451	4.484	1.099

Table S180 Frequencies (cm^{-1}) of hydrogen abstraction transition state between hydroxyl radical and β-propyltetracene molecule, calculated at the M06-2X/cc-pVDZ level of theory.

-1080	33	45	50	56	82	100	110
147	159	169	196	238	248	256	272
297	332	360	373	391	454	473	480
489	496	500	551	571	590	630	640
679	728	736	756	759	765	774	778
789	813	834	858	863	869	882	895
904	914	917	923	946	951	978	996
1015	1027	1070	1091	1104	1116	1125	1152
1172	1184	1192	1212	1228	1242	1257	1276
1285	1292	1306	1319	1323	1338	1347	1372
1388	1393	1435	1452	1461	1468	1477	1482
1482	1487	1490	1502	1590	1622	1626	1642
1686	1703	1718	3058	3060	3070	3099	3119
3136	3144	3186	3193	3195	3195	3197	3198
3202	3207	3218	3229	3787			

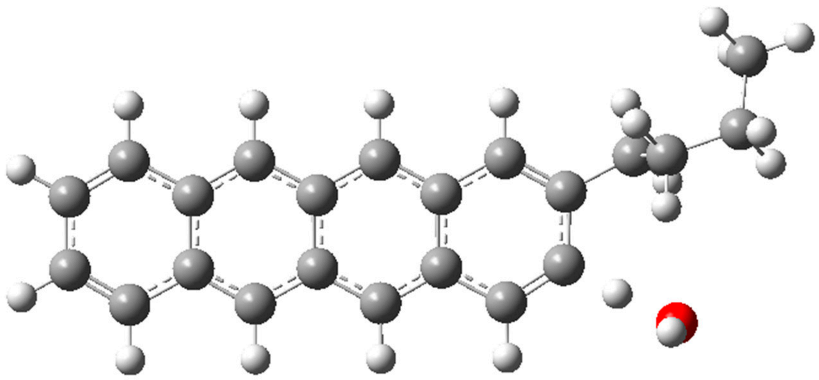

Figure S91 Visualization of hydrogen abstraction transition state between hydroxyl radical and β-butyltetracene molecule, calculated at the M06-2X/cc-pVDZ level of theory.

Table S181 Geometry (Å) of hydrogen abstraction transition state between hydroxyl radical and β-butyltetracene molecule, calculated at the M06-2X/cc-pVDZ level of theory

Atom	x	y	z
C	1.222	2.032	-0.044
C	2.422	1.407	-0.045
C	2.462	-0.035	-0.027
C	1.207	-0.751	0.002
C	-0.024	-0.006	0.011
C	-0.045	1.359	-0.005
C	3.658	-0.736	-0.039
C	1.221	-2.139	0.020
C	2.431	-2.867	0.012
C	3.682	-2.148	-0.019
C	4.890	-2.874	-0.029
H	5.835	-2.330	-0.053
C	4.901	-4.263	-0.010
C	3.650	-4.983	0.022
C	2.454	-4.277	0.031
H	4.601	-0.190	-0.064
H	1.195	3.232	-0.103
H	0.277	-2.685	0.042
C	3.684	-6.421	0.042
H	-0.964	-0.561	0.028
H	1.509	-4.822	0.055
H	2.738	-6.961	0.066
C	4.869	-7.092	0.031
C	6.113	-6.376	0.000
H	7.048	-6.933	-0.008
C	6.127	-5.014	-0.020
H	7.070	-4.468	-0.044
H	4.882	-8.180	0.047
H	3.357	1.966	-0.072
C	-1.315	2.163	0.052
H	-2.151	1.538	-0.282
H	-1.235	3.018	-0.635
C	-1.587	2.692	1.467
H	-1.796	1.839	2.129
H	-0.674	3.172	1.845
C	-2.740	3.693	1.520
H	-2.847	4.052	2.553
H	-2.476	4.568	0.908
C	-4.071	3.113	1.046
H	-4.887	3.830	1.192
H	-4.042	2.860	-0.021
H	-4.319	2.200	1.605

**O**	1.132	4.524	0.157
**H**	1.380	4.493	1.097

Table S182 Frequencies (cm^{-1}) of hydrogen abstraction transition state between hydroxyl radical and β-butyltetracene molecule, calculated at the M06-2X/cc-pVDZ level of theory.

-1081	27	34	44	52	78	85	105
125	148	155	167	196	207	251	263
284	295	313	357	372	391	425	458
474	480	488	496	503	551	572	590
630	640	679	727	737	758	760	766
774	778	786	789	819	837	858	863
880	895	897	913	916	923	936	947
951	974	992	996	1015	1029	1092	1107
1115	1118	1129	1153	1174	1187	1194	1212
1214	1233	1254	1265	1278	1286	1295	1313
1318	1324	1340	1350	1358	1378	1389	1397
1437	1453	1455	1469	1474	1481	1484	1488
1488	1490	1504	1591	1623	1628	1643	1688
1702	1717	3050	3057	3060	3067	3098	3102
3128	3134	3145	3184	3192	3193	3194	3195
3197	3201	3206	3216	3229	3789		

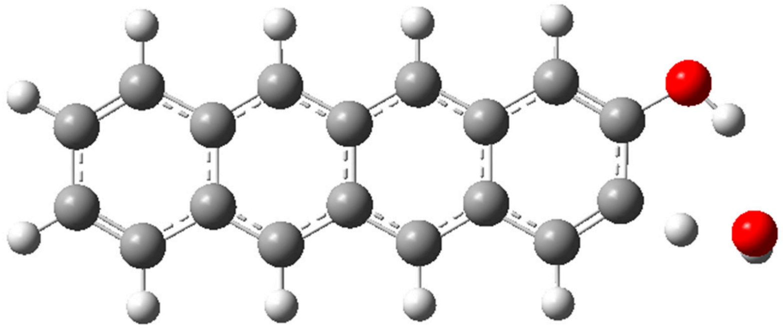

Figure S92 Visualization of hydrogen abstraction transition state between hydroxyl radical and β-hydroxytetracene molecule, calculated at the M06-2X/cc-pVDZ level of theory.

Table S183 Geometry (Å) of hydrogen abstraction transition state between hydroxyl radical and β-hydroxytetracene molecule, calculated at the M06-2X/cc-pVDZ level of theory

Atom	x	y	z
**C**	-3.679	0.678	-0.019
**C**	-2.517	1.387	-0.020
**C**	-1.244	0.717	-0.010
**C**	-1.222	-0.727	0.001
**C**	-2.475	-1.433	0.001
**C**	-3.657	-0.758	-0.008
**C**	-0.045	1.418	-0.011

**C**	-0.003	-1.394	0.010
**C**	1.221	-0.693	0.009
**C**	1.197	0.749	-0.001
**C**	2.421	1.450	-0.004
**H**	2.409	2.541	-0.014
**C**	3.636	0.780	0.006
**C**	2.465	-1.362	0.018
**H**	-0.060	2.508	-0.019
**H**	-4.637	1.195	-0.026
**H**	-2.529	2.477	-0.028
**H**	-2.456	-2.523	0.009
**H**	-4.599	-1.304	-0.007
**H**	0.011	-2.484	0.018
**H**	2.477	-2.452	0.026
**C**	3.668	-0.667	0.017
**C**	4.932	-1.348	0.031
**C**	6.101	-0.644	0.028
**H**	4.964	-2.436	0.039
**C**	4.888	1.494	0.012
**H**	4.883	2.584	0.014
**O**	7.299	-1.272	0.039
**H**	8.000	-0.602	0.064
**C**	6.040	0.789	0.000
**H**	7.124	1.329	-0.049
**O**	8.398	1.416	0.255
**H**	8.314	1.642	1.198

Table S184 Frequencies (cm^{-1}) of hydrogen abstraction transition state between hydroxyl radical and β-hydroxytetracene molecule, calculated at the M06-2X/cc-pVDZ level of theory.

-1195	38	67	95	108	137	166	188
241	267	274	287	308	364	375	396
423	474	480	486	498	502	538	549
592	614	627	639	662	725	744	751
758	772	777	788	795	835	856	864
876	887	910	917	920	944	949	966
996	1015	1027	1120	1155	1170	1174	1181
1203	1214	1248	1272	1282	1288	1304	1324
1350	1358	1387	1438	1448	1453	1489	1492
1506	1524	1594	1627	1636	1648	1687	1708
1720	3194	3196	3197	3199	3200	3203	3212
3218	3220	3229	3746	3779			

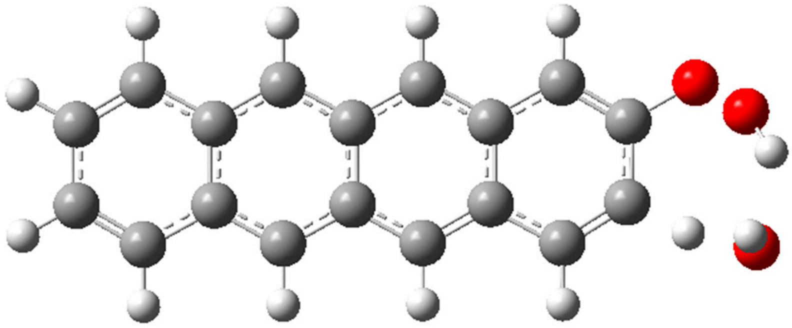

Figure S93 Visualization of hydrogen abstraction transition state between hydroxyl radical and β -peroxytetracene molecule, calculated at the M06-2X/cc-pVDZ level of theory.

Table S185 Geometry (Å) of hydrogen abstraction transition state between hydroxyl radical and β-peroxytetracene molecule, calculated at the M06-2X/cc-pVDZ level of theory

Atom	x	y	z
C	-2.396	-1.393	-0.089
C	-1.168	-0.702	-0.054
C	-1.179	0.737	0.036
C	-2.414	1.414	0.086
C	-3.620	0.723	0.051
C	0.071	-1.379	-0.105
C	0.053	1.429	0.073
C	1.261	0.752	0.024
C	1.275	-0.692	-0.069
C	2.537	-1.382	-0.107
H	2.568	-2.469	-0.156
C	3.702	-0.682	-0.077
C	3.670	0.748	0.011
C	2.522	1.457	0.045
H	0.076	-2.467	-0.173
H	-2.391	-2.482	-0.157
H	-2.421	2.502	0.155
H	0.048	2.517	0.143
H	2.523	2.545	0.080
H	4.745	1.307	0.022
O	4.899	-1.340	-0.219
O	5.765	-0.965	0.853
H	6.214	-0.184	0.478
O	5.963	1.611	-0.335
H	5.868	1.547	-1.301
C	-3.610	-0.718	-0.039
C	-4.868	-1.413	-0.074
C	-6.045	-0.730	-0.024
H	-4.858	-2.501	-0.142
C	-4.886	1.402	0.102
C	-6.054	0.703	0.066

H	-6.992	-1.267	-0.051
H	-4.889	2.489	0.170
H	-7.008	1.227	0.105

Table S186 Frequencies (cm^{-1}) of hydrogen abstraction transition state between hydroxyl radical and β-peroxytetracene molecule, calculated at the M06-2X/cc-pVDZ level of theory.

-1222	34	59	63	98	121	144	177
185	218	246	264	273	291	334	370
380	409	430	470	480	489	499	524
532	552	572	614	635	643	689	726
758	760	763	772	779	789	812	829
857	867	882	892	913	916	922	946
951	964	996	1007	1016	1028	1110	1125
1156	1174	1177	1198	1214	1247	1272	1285
1296	1312	1326	1349	1360	1389	1442	1453
1484	1488	1489	1497	1504	1593	1628	1632
1643	1687	1700	1719	3194	3196	3197	3200
3200	3203	3211	3218	3221	3229	3677	3781

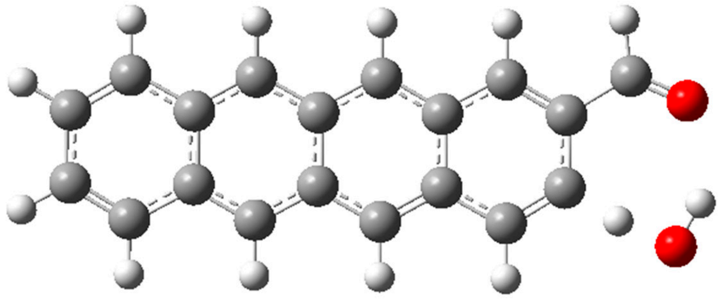

Figure S94 Visualization of hydrogen abstraction transition state between hydroxyl radical and β-tetraldehyde molecule, calculated at the M06-2X/cc-pVDZ level of theory.

Table S187 Geometry (Å) of hydrogen abstraction transition state between hydroxyl radical and β-tetraldehyde molecule, calculated at the M06-2X/cc-pVDZ level of theory

Atom	x	y	z
C	-3.654	0.695	-0.001
C	-2.486	1.395	0.001
C	-1.220	0.715	0.001
C	-1.212	-0.729	-0.002
C	-2.469	-1.426	-0.003
C	-3.646	-0.741	-0.003
C	-0.015	1.408	0.002
C	0.002	-1.405	-0.002
C	1.229	-0.710	-0.001

**C**	1.221	0.733	0.002
**C**	2.452	1.428	0.003
**H**	2.444	2.519	0.005
**C**	3.662	0.754	0.003
**C**	2.467	-1.387	-0.001
**H**	-0.024	2.499	0.004
**H**	-4.608	1.221	0.000
**H**	-2.490	2.485	0.003
**H**	-2.459	-2.516	-0.005
**H**	-4.593	-1.278	-0.004
**H**	0.009	-2.496	-0.004
**H**	2.476	-2.478	-0.003
**C**	3.669	-0.692	0.000
**C**	4.930	-1.373	0.000
**C**	6.114	-0.684	0.001
**H**	4.939	-2.465	-0.002
**C**	4.927	1.452	0.004
**H**	4.930	2.542	0.006
**C**	7.375	-1.454	0.000
**O**	8.487	-0.972	0.001
**H**	7.243	-2.557	-0.001
**C**	6.081	0.750	0.004
**H**	7.138	1.395	0.005
**O**	8.274	1.889	0.007
**H**	8.753	1.038	0.005

Table S188 Frequencies (cm^{-1}) of hydrogen abstraction transition state between hydroxyl radical and β-tetraldehyde molecule, calculated at the M06-2X/cc-pVDZ level of theory.

-1548	34	54	92	97	104	170	170
191	230	264	265	296	322	361	362
389	446	453	479	479	488	499	529
545	596	610	637	646	665	741	759
766	776	779	782	791	815	821	835
862	878	892	898	915	928	937	955
963	998	1017	1018	1029	1042	1127	1156
1176	1190	1199	1219	1244	1270	1284	1296
1325	1340	1349	1383	1401	1418	1447	1455
1484	1489	1493	1506	1592	1623	1628	1641
1684	1691	1719	1812	2984	3188	3198	3199
3200	3202	3203	3206	3211	3220	3231	3706

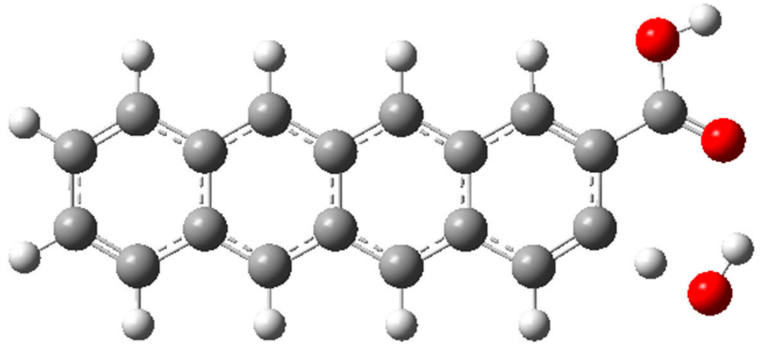

Figure S95 Visualization of hydrogen abstraction transition state between hydroxyl radical and β -tetracenic acid molecule, calculated at the M06-2X/cc-pVDZ level of theory.

Table S189 Geometry (Å) of hydrogen abstraction transition state between hydroxyl radical and β-tetracenic acid molecule, calculated at the M06-2X/cc-pVDZ level of theory

Atom	x	y	z
C	-3.653	0.718	-0.001
C	-2.480	1.410	0.000
C	-1.218	0.721	0.000
C	-1.220	-0.724	-0.001
C	-2.483	-1.412	-0.001
C	-3.655	-0.718	-0.001
C	-0.009	1.405	0.001
C	-0.012	-1.408	-0.001
C	1.221	-0.722	0.000
C	1.223	0.721	0.001
C	2.458	1.407	0.002
H	2.459	2.498	0.002
C	3.664	0.723	0.002
C	2.453	-1.408	0.000
H	-0.010	2.496	0.001
H	-4.602	1.251	-0.001
H	-2.476	2.500	0.001
H	-2.480	-2.501	-0.002
H	-4.605	-1.249	-0.002
H	-0.012	-2.499	-0.001
H	2.455	-2.499	-0.001
C	3.661	-0.722	0.001
C	4.913	-1.421	0.001
C	6.102	-0.742	0.001
H	4.909	-2.510	0.000
C	4.934	1.407	0.002
H	4.952	2.496	0.003
C	7.398	-1.466	0.001
O	8.490	-0.942	0.002
O	7.260	-2.806	0.000

**H**	8.155	-3.175	0.001
**C**	6.081	0.691	0.002
**H**	7.131	1.342	0.003
**O**	8.240	1.902	0.003
**H**	8.758	1.075	0.004

Table S190 Frequencies (cm^{-1}) of hydrogen abstraction transition state between hydroxyl radical and β-tetracenic acid molecule, calculated at the M06-2X/cc-pVDZ level of theory.

-1560	35	50	62	94	94	122	164
185	199	222	262	290	315	343	355
386	432	433	454	477	486	497	518
530	531	562	608	619	637	640	659
740	741	759	776	776	777	781	791
808	826	829	862	880	891	902	913
932	939	957	969	997	997	1017	1027
1122	1131	1154	1174	1186	1212	1216	1236
1269	1281	1292	1324	1330	1345	1376	1395
1409	1445	1454	1480	1488	1498	1504	1590
1619	1626	1640	1684	1692	1717	1828	3195
3197	3197	3200	3200	3203	3210	3218	3224
3230	3730	3824					
